# Cross-modal contrastive learning decodes developmental regulatory features through chromatin potential analysis

**DOI:** 10.1093/gigascience/giaf053

**Published:** 2025-10-17

**Authors:** Yueyuxiao Yang, Chenxi Xie, Qiushun He, Meng Yang

**Affiliations:** MGI Tech Co., Ltd., Shenzhen 518083, China; MGI Tech Co., Ltd., Shenzhen 518083, China; MGI Tech Co., Ltd., Shenzhen 518083, China; MGI Tech Co., Ltd., Shenzhen 518083, China

**Keywords:** contrastive learning, multimodality, developmental regulation, chromatin potential analysis

## Abstract

**Background:**

Emerging large-scale multimodal single-cell data jointly measure chromatin accessibility and transcription in the same cell, thus reconciling matched data paves an integrated route for comprehensive regulatory analysis.

**Findings:**

Here, we introduce Attune, a cross-modal contrastive learning framework to align paired gene expression and accessibility information. Systematic benchmarking shows Attune’s superior performance for omics integration and gene expression prediction. We further introduce transformer-based cross-modal attention over fine-tuned gene and peak embeddings to infer regulatory interaction and discover significant differential signals of cell subtypes. Applied to a hair follicle maturation dataset, Attune reveals chromatin potential for the bifunctional transcription factor Gli3 at the gene level. In addition, the paired representations determine transmitted states across neonatal and mature cell types of cortical neuron differentiation at the cell level. Taken together, Attune offers an approach for regulatory inference across omics layers and enables more advanced omics analyses.

**Conclusions:**

Attune offers a versatile framework for integrating gene expression and chromatin accessibility, enabling the inference of regulatory mechanisms and the prediction of gene expression from cross-modal data.

## Background

Gene transcription is a dynamic process that drives the precise differentiation of cell lineages [1]. This complex process is orchestrated by coordinated regulation of chromatin accessibility around key regulatory elements, such as promoters and enhancers, which creates a permissive landscape for the binding of transcription factors and cofactors, thus initiating transcription [2].

Recent advancements in multimodal sequencing technologies, such as 10x Multiome, SHARE-seq [3], SNARE-seq [4], and scCAT-seq [5], have enabled the simultaneous measurement of multiple layers of a single cell, including chromatin and transcriptional status. By capitalizing on these data, it becomes conceivable to refine cell identity and reconstruct the causal sequence of the regulatory network [6] that underlies cellular differentiation. Moreover, by integrating information from multiple modalities within a joint embedded space and accounting for temporal dynamics, we can unearth the intricate relationships and dependencies that permeate across these modalities. Despite the existence of various techniques for integrating multimodal single-cell data, such as Seurat V4 [7], MultiVI [8], and BABEL [9], they are predominantly focused on integration or prediction tasks, resulting in a pressing need for approaches to elucidate the regulation of cell differentiation. GLUE [10] employs a guidance graph to explicitly model *cis*-regulatory interactions between feature spaces, while MIRA utilizes a combination of topic modeling and regulatory potential modeling to capture key regulators at lineage branch points [11]. Nevertheless, the pool of available methods specifically designed for this purpose remains limited.

Multimodal deep learning offers a panoramic understanding of data, wielding substantial power. For example, Vision-Language (VL) pretraining has demonstrated remarkable efficacy in various VL downstream tasks [12], as evidenced by the success of CLIP [13] and ALBEF [14]. These models utilize a contrastive learning module to pretrain the encoder and subsequently fine-tune it with a transformer-based decoder. In the context of single-cell multimodal data, the deployment of multimodal deep learning algorithms becomes profoundly advantageous. Drawing inspiration from the exceptional representation capabilities of contrastive learning for multimodal data [13, 15], as well as the impressive performance of transformer models in uncovering latent interactions [14, 16], we introduce Attune: a cross-modal contrastive learning pretraining model designed to capture the interactions between different modalities. By maximizing the agreement between modalities within a cell on the hypersphere [17], Attune places representations of distinct modalities into a shared feature space, enabling the modeling of interactions between peaks and genes.

Using Attune, we apply learned cell embeddings to a range of downstream tasks and achieve superior performance in cross-modal prediction tasks. By employing a transformer-based decoder, we can construct gene–peak interaction networks and interrogate the regulations underlying key developmental processes, such as the maturation of transit-amplifying cells in the hair follicle dataset and the active transition region during neuron differentiation in the cortex dataset. Comprehensive benchmarking and regulation analysis demonstrate the power of Attune in learning comprehensive and informative representations of omics-specific features and reconstructing regulatory interactions.

## Results

### Attune achieves exceptional overall performance of integration

**Figure 1:**
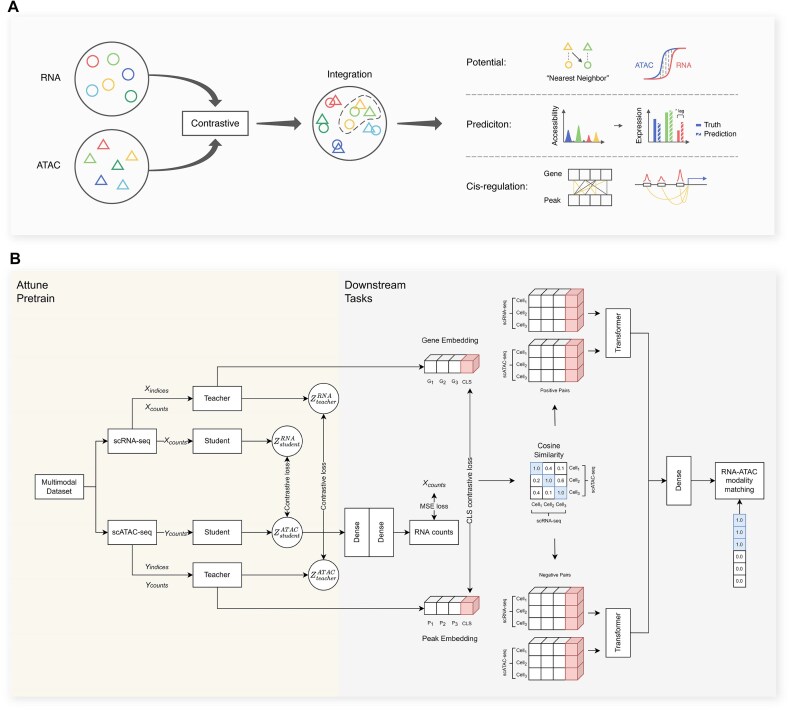
The overall framework of Attune. (A) Schematic of Attune and downstream tasks. Each cell has 2 modalities from scRNA-seq and scATAC-seq (the color indicates different cells and the shapes indicate different modalities), and each cell’s 2 modalities are positive pairs to be pulled together while other cells are pushed away via contrastive learning. Learned cell embeddings after integration are fed into downstream tasks, including cross-modality prediction, differentiation analysis, and inference of gene–peak interaction. (B) Overview of Attune model and transformer-based decoder architecture. Attune consists of 2 asymmetric teacher–student networks. We propose cross-modal contrastive loss to integrate the cell embeddings of matched RNA–ATAC pairs into a common space. We fine-tune Attune via a transformer-based decoder for recovering regulatory events, which is used to learn multimodal interactions between peak and gene. We extract gene embedding and peak embedding from teacher networks. The green block denotes each gene or peak, and the yellow block denotes CLS token. A CLS contrastive loss is applied to shorten the distance between the matched RNA–ATAC pairs globally. The cosine similarity between cells in-batch is obtained by CLS contrastive learning. The cell pair with highest similarity is taken as positive pairs (matched), and cells with low similarity are randomly selected as negative pairs (unmatched). Then the positive pairs and negative pairs are input into the transformer in turn. The output of the positive pairs and negative pairs in the transformer is concatenated and fed into the dense layer, followed by softmax for 2-class prediction (positive pairs or negative pairs). Then the cross-entropy with one-hot label (1 for positive pairs) for RNA–ATAC modality matching is calculated.

Attune employs a cross-modal contrastive learning approach to integrate single-cell RNA sequencing (scRNA-seq) and single-cell ATAC sequencing (scATAC-seq) data, effectively preserving biological consistency across both modalities. The architecture consists of the Attune pretrained model and 2 downstream modules: the cross-modal prediction module and the transformer-based peak–gene interaction module. To learn cell embeddings from scRNA-seq and scATAC-seq data, Attune utilizes 2 asymmetric teacher–student networks, which are trained through cross-modal contrastive learning. These learned cell embeddings can be fine-tuned for various downstream tasks, including cross-modal prediction, peak–gene interaction recovery, and differentiation analysis, as depicted in Fig. 1.

To demonstrate the performance of Attune, we benchmark it against other multimodal integration methods on matched scRNA-seq and scATAC-seq datasets, such as 10x Multiome and SHARE-seq, with several established metrics [10, 18], most of which have been widely embraced and validated in previous single-cell integration tasks, such as graph connectivity (GC) in the NeurIPS 2021 competition [19] and average silhouette width (ASW) in scJoint [20] (see Methods in detail).

**Figure 2:**
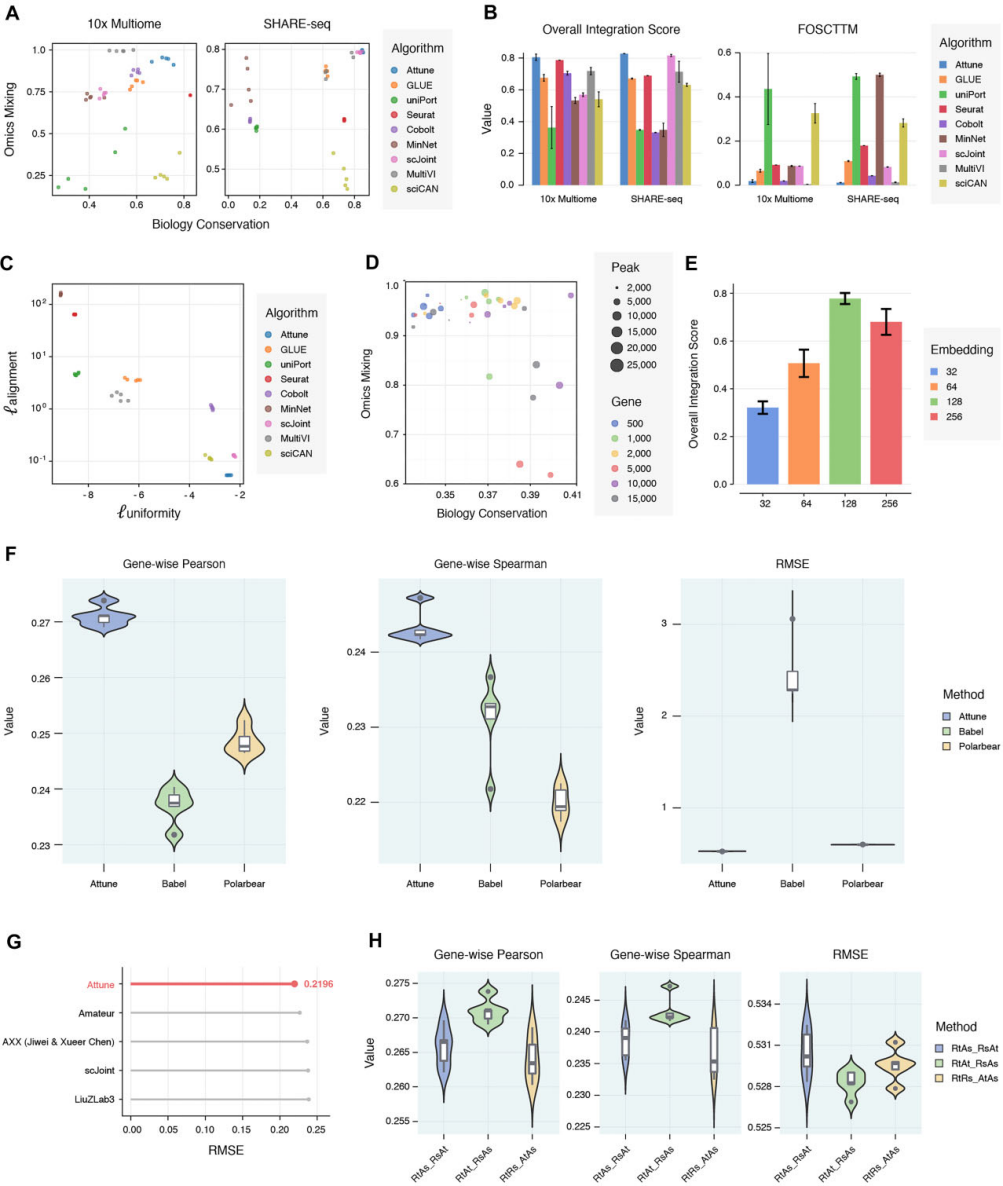
Comprehensive benchmarks of integration and cross-modal prediction performance. (A) Biology conservation score versus omics integration score for different methods (repeated 5 times with different random seeds) on the 10x Multiome dataset (*n* = 11,909 cells, left) and SHARE-seq dataset (*n* = 32,231 cells, right). (B) Comparison of overall integration score on the 10x Multiome dataset and SHARE-seq dataset on the left and comparison of FOSCTTM on the right. Error bars represent the 95% confidence interval. (C) Alignment–uniformity plot for Attune, GLUE, LIGER, Seurat, Cobolt, MinNet, scJoint, and MultiVI on the 10x Multiome dataset. There are 5 replicates (represented by dots) for each method. (D) Integration performance of Attune under different numbers of gene and peak settings on the 10x Multiome dataset. The size of the dot indicates the number of peaks, and the color indicates the number of genes. (E) Ablation study of Attune feature embeddings under different numbers of dimension settings. The bar shows the overall integration score for 10x Multiome datasets (repeated 5 times with different random seeds). Error bars represent the 95% confidence interval. (F) Comparing the performance of modality predictions (5-fold cross-validation) in terms of gene-wise Pearson correlation coefficient, gene-wise Spearman correlation coefficient, and RMSE on the 10x Multiome dataset. Comparison is made with Babel and Polarbear. (G) Performance comparison of modality prediction against the top winners in the NeurIPS 2021 competition. (H) Ablation study on contrast between different modalities or networks; RtAs_RsAt represents 1 set of contrast learning between RNA teacher network and ATAC student network and another set of contrast learning between RNA student network and ATAC teacher network. The violin plots show the modality prediction performance for 10x Multiome datasets (5-fold cross-validation). In the boxplots included in violin plots: center line, median; box, interquartile range (IQR; the range between the 25th and 75th percentiles); whiskers, 1.5 × IQR; dots, outliers.

As shown in Fig. 2A (left) (refer to Supplementary Table S2 for details), Attune emerges as the clear winner, situated in the top-right corner of the 10x Multiome dataset, signifying that it strikes a balance between omics mixing and biological fidelity. MultiVI, on the other hand, demonstrates higher omics mixing but sacrifices biological meaning. Moreover, Attune outperforms other methods on the SHARE-seq dataset (Fig. 2A [right], refer to Supplementary Table S3 for details), as evaluated by 3 separate metrics (see Supplementary Fig. S1). Upon probing Attune’s integration scores on distinct datasets, we observe that it obtains the highest overall integration scores (mean 0.806 and 0.829 for 10x Multiome and SHARE-seq data, respectively) in Fig. 2B (left). The UMAP visualization of the cell embeddings for the 10x Multiome and SHARE-seq datasets is presented in Supplementary Figs. 2 and 3, respectively. We also quantify the alignment performance between modalities using the fraction of samples closer than the true match (FOSCTTM) on both datasets, as depicted in Fig. 2B (right). The lowest FOSCTTM scores suggest that Attune effectively matches different modalities from a cell.

To further evaluate the performance of Attune’s feature distributions on the output unit hypersphere, we measure the alignment between cross-modal positive pairs and the uniformity of the entire representation space. This assessment allows us to determine the quality of learned embeddings. Compared to other integration techniques’ cell embeddings (Fig. 2C and Supplementary Table S4), Attune achieves the best alignment (the lower the better), indicating that the feature distribution between RNA–ATAC pairs is more consistent in the high-dimensional space. Other components regarding feature selection settings and hyperparameter choice are presented in Fig. 2D and Fig. 2E, respectively (refer to Supplementary Tables S5 and S6 for more information) to substantiate the rationality under the current settings, thus enhancing the validity of the study.

The cells’ embedding after integration establishes the cell labels and ontologies. Through multimodal reference building and mapping of query cells, it demonstrates that the embedding of cells retains their original biological characteristics [21, 22]. We assess Attune’s ability to map query cells, including previously unseen cell types, onto reference embeddings. We utilize 80% of the cells from the 10x Multiome dataset to construct the reference embeddings, while the remaining 20% serves as the query cells with unseen cell types. Specifically, we first train the reference cells to obtain the reference embeddings. Next, we directly infer the query cells using pretrained model weights to generate the query embeddings. Finally, we concatenate the reference embeddings and query embeddings along the sample dimension and visualize them using UMAP (refer to Supplementary Fig. S9). In Supplementary Fig. S9a, we deliberately exclude all CD14 monocyte cells (CD14 Mono) from the reference. Despite never encountering CD14 monocyte cells during training, Attune accurately localizes them between CD16 monocyte cells (CD16 Mono) and conventional dendritic cells (CDCs), with query cells positioned in proximity to their most similar reference cells. Similarly, Supplementary Fig. S9b and c demonstrate comparable outcomes. These findings emphatically highlight Attune’s prowess in integrating multimodal data and acquiring biologically meaningful embeddings.

The essence of Attune lies in its cross-modal contrastive learning module. Taking inspiration from the pioneering work of Concerto [15], we regard Attune’s multimodal contrastive learning framework as an indivisible entity. However, the efficacy of integration can be influenced by different internal comparison objects. To establish the intrinsic soundness of our module design, we conduct an ablation experiment on the cross-modal contrastive learning module. Since the module employs 2 asymmetric teacher–student networks, we compare 3 different designs. The first design involves comparing the RNA student network with the ATAC student network and, correspondingly, contrasting the RNA teacher network with the ATAC teacher network, as proposed in this study. The second one compares the RNA student network with the RNA teacher network, while simultaneously comparing the ATAC student network with the ATAC teacher network. Finally, the third design involves comparing the RNA student network with the ATAC teacher network and, conversely, contrasting the RNA teacher network with the ATAC student network. We assess the performance disparities among these 3 comparison methods in the context of multimodal integration (Supplementary Table S7) and discover that the first comparison method yields the most exceptional outcomes. This observation suggests that the improved performance of Attune stems not from a mere amalgamation of network components, but rather from the ingenious architectural design.

### Internal relation is well captured by Attune, resulting in outstanding performance in cross-modal prediction

In cross-modal prediction, our objective is to predict all feature values for each cell in scRNA-seq using scATAC-seq, and this requires algorithms to learn the complex regulatory interactions between layers of genetic information. To assess the performance of Attune, we compare it against state-of-the-art cross-modal prediction methods such as BABEL [9] and Polarbear [23], using the 10x Multiome dataset (PBMC10k, *n* = 11,909). We randomly divide cells into training (*n* = 9,527) and testing (*n* = 2,382) sets (bootstrapping 5 times) and evaluate the method using gene-wise Pearson correlation and gene-wise Spearman’s correlation. Our findings show that Attune outperforms other methods with the highest Spearman’s correlation coefficient (0.243), the highest Pearson correlation coefficient (0.271), and the lowest root mean square error (RMSE) of 0.528. Figure 2F provides a visual representation of our results, while Supplementary Table S8 contains detailed information.

To further verify our findings, we compare the performance of Attune with the top 5 winners from the modality prediction task (ATAC-GEX subtask) using the official settings, datasets, and guidelines from the multimodal single-cell data integration competition of NeurIPS 2021 [19]. As illustrated in Fig. 2G and Supplementary Table S9, Attune outperforms all other methods with the lowest RMSE.

We posit that the efficacy of cross-modal prediction may be significantly influenced by the integration of pretraining. To investigate this hypothesis, we design a comparative experiment to demonstrate the benefits derived from fine-tuning Attune’s pretrained model through the utilization of a multilayer perceptron (MLP). Our investigation entails an examination of 3 distinct settings: an MLP network with pretraining, an MLP network without pretraining (*de novo* training), and classical regression methods such as LASSO (Supplementary Table S10). Additionally, we conduct a comprehensive ablation study to elucidate the impact of Attune’s structure on the performance of cross-modal prediction, as presented in Fig. 2H and Supplementary Table S11. These results prominently underscore the significant impact of Attune’s pretrained model on the robustness of fine-tuning and its potential for downstream tasks.

### Transformer’s cross-attention mechanism enables revealing regulatory interaction of genes via fine-tuning Attune

Various metrics are utilized to infer regulatory interactions by quantifying the relationship between modalities. For instance, Cao and Gao [10] utilized cosine similarity of different feature embeddings, while Trevino et al. [24] and Ma et al. [3] used correlation metrics to evaluate the relationship between genes and peaks. In this work, we leverage the intrinsic property of the transformer (i.e., the ability of cross-attention to discover inner connections) to quantify associations between genes and peaks by utilizing attention weight.

**Figure 3:**
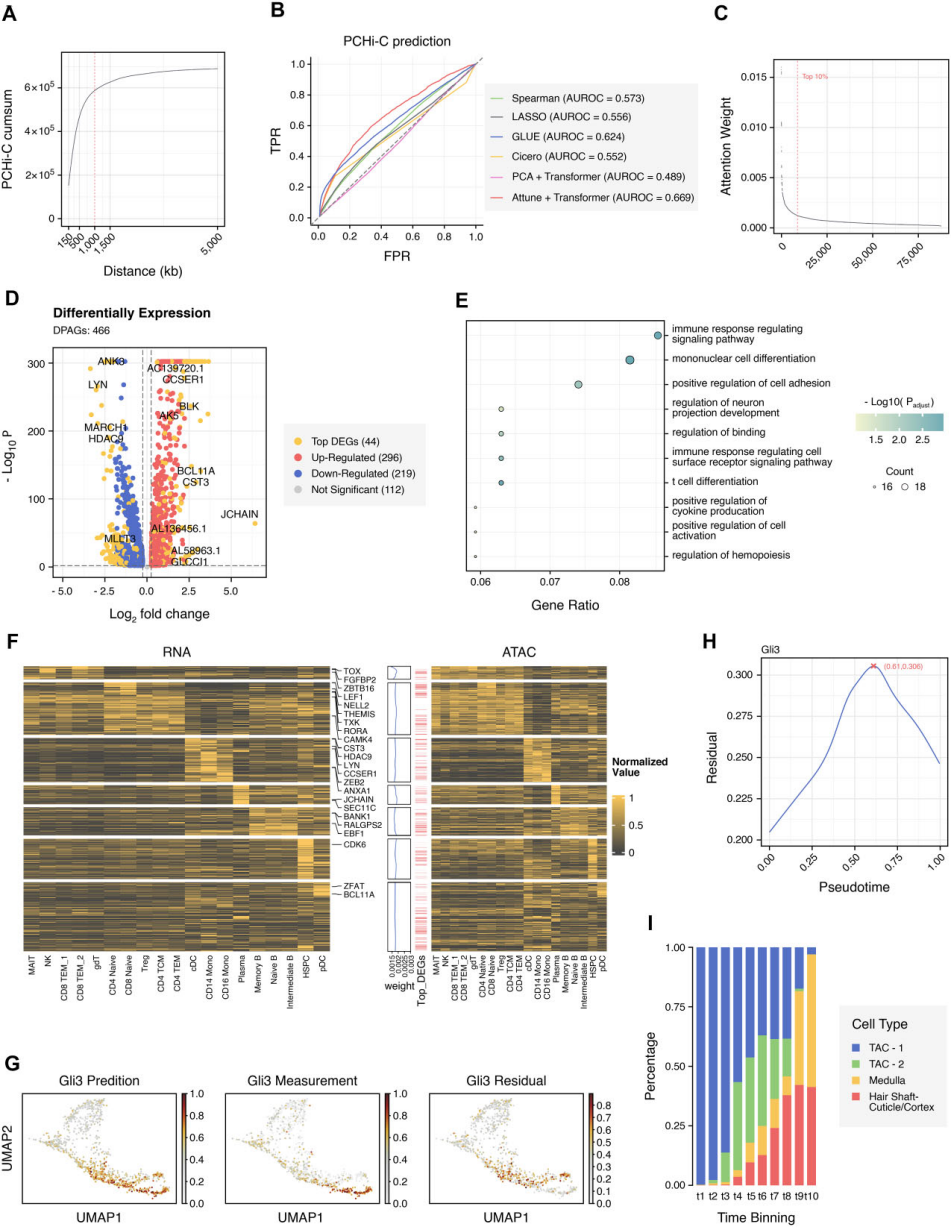
Regulatory interaction analysis of Attune on the 10x Multiome dataset. (A) Distribution of distance between peaks and promoters in the PCHi-C dataset. (B) Comparison of AUROC among 6 methods, including Attune + Transformer, PCA + Transformer, GLUE, Spearman, LASSO, and Cicero, on the PCHi-C dataset. The threshold for peak-promoter distance is set to 1,200 kb. (C) Attention weight is in descending order, and the cutoff of the top 10% is labeled on the 10x Multiome dataset. (D) Differential expression of 466 DPAGs in the 10x Multiome dataset, with some top DEGs highlighted. (E) Enrichment analysis of biological process within differentially expressed genes. (F) Heatmap of gene expression or accessibility with cell types. Each row represents a gene–peak pair extracted by attention weight. (G) UMAP visualization of prediction, measurement, and the residual value of Gli3 gene on the SHARE-seq dataset. (H) Trend of Gli3’s residual from the SHARE-seq dataset along the pseudotime. The maximum residual value is highlighted. (I) Composition of cell types in 10 pseudo-samples.

To demonstrate the efficacy of the transformer in discovering regulatory interactions, we employ a matched scRNA-seq and scATAC-seq multiome dataset from 10x Genomics, consisting of approximately 11,000 human peripheral blood mononuclear cells (PBMCs). Previous studies have suggested that regulatory elements, such as enhancers and silencers, may be distributed away from promoters up to several Mbps [25, 26]. Based on these observations and the statistical analysis of the dataset from Javierre et al. [27] (see Fig. 3A), we mask genes and peaks whose distances are greater than 1.2 Mbps (see Supplementary Fig. S4a and Methods) for comprehensive prediction and evaluation [28, 29]. We utilize a Promoter capture Hi-C (PCHi-C) dataset of human PBMCs that profiles distal promoter-interacting regions as a validated resource [27]. As illustrated in Fig. 3B, Attune + Transformer (i.e., training the Transformer model by fine-tuning Attune’s pretrained model) outperforms other methods, including Cicero [30], LASSO, and GLUE [10], in regulatory prediction, indicating that cross-attention learned by Transformer (with Attune pretraining) captures promoter-interacting regions effectively.

To further substantiate the benefits of Attune’s pretraining, we introduce an ablation experiment. This experiment incorporates the principal component analysis (PCA) + Transformer configuration, where the Transformer is trained solely on the gene embeddings and peak embeddings derived from the first 10 principal components (PCs) extracted via PCA, without the utilization of Attune pretraining. These comparisons in Fig. 3B highlight the role of Attune pretraining in facilitating the Transformer’s effective capture of promoter-interacting regions.

To elucidate the biological signals captured by the attention mechanism, we select gene–peak pairs with the top 10% of attention weight using an inflection point (“elbow”) when ranking gene–peak pairs by attention weight (Fig. 3C). A total of 8,744 gene–peak pairs remain under this cutoff, including 5,447 peaks and 646 genes (full list in Supplementary Table S13). We define 466 genes linking at least 10 peaks among them as dense peak-associated genes (DPAGs). Key regulatory events may occur within DPAGs and their associated peaks [3]. Most of the DPAGs express differentially (354 versus 466, see Supplementary Table S13 for details) and are enriched in immune response regulating signaling (*P*-adjust = 0.001), mononuclear cell differentiation (*P*-adjust = 0.001), and positive regulation of cell adhesion (*P*-adjust = 0.005), as shown in Fig. 3D and 3E. DPAGs include cell markers of plasma (JCHAIN, SEC11C), HSPC (CDK6), pDC (BCL11A, ZFAT), B naive or B memory cells (BANK1, EBF1), and so on, and clear separation of cell types is observed in Fig. 3F and Supplementary Fig. S4b from both modalities. Similar results of the SHARE-seq dataset are displayed in Supplementary Fig. S5.

### Attune enables chromatin potential discovery and illuminates the priming of lineage

Attune outperforms other methods in predicting cell modalities, but some genes exhibit low Pearson correlation coefficients. Based on the delay between chromatin accessibility and transcription [2, 3, 31], we propose that chromatin potential, which refers to the latent information underlying chromatin accessibility or transcriptional delay, may account for the inaccurate prediction of certain genes, particularly during lineage development. To investigate this hypothesis, we calculate the residuals between predicted and measured gene expressions on the mouse skin SHARE-seq dataset, which represents chromatin potential, as hair follicles remain in the cell cycle, even in adulthood. Supplementary Fig. S7a and b illustrate that residuals display diverse patterns of gene expression during cell differentiation, evident by their trends along pseudotime (see Methods). Some genes, including Hexb, Arl15, Styx, and Atp8b1, exhibit consistency between predicted and measured expressions, whereas Gli3 displays conspicuous residuals (Fig. 3G, Supplementary Fig. S7c). Notably, the high residuals of Gli3 emerge before cell-type transition, known as lineage commitment [3], as indicated by the low-dimensional projection of cell type and pseudotime in Supplementary Fig. S6a–b and the change in residual in Fig. 3H, pointing to a delay between modalities.

As an example, Gli3 serves a critical function in the Hedgehog pathway (Hh) and regulates hair follicle cycles in embryonic and adult skin [32, 33]. In the canonical Hh pathway, Gli3 primarily acts as a repressor to maintain pathway activity balance [34, 35]. Regulon analysis by SCENIC [36] confirms its role in transcriptional inhibition, with network importance scores for refined Gli3 regulon target genes listed in Supplementary Table S14, such as Basp1 (14.03), Sema4a (5.21), and Myh14 (3.36). Given its diverse functions and multiple targets during development, we speculate that the lag of Gli3 contributes to lineage differentiation.

#### Regulation of Gli3 orderly shift with the maturity of cells

**Figure 4:**
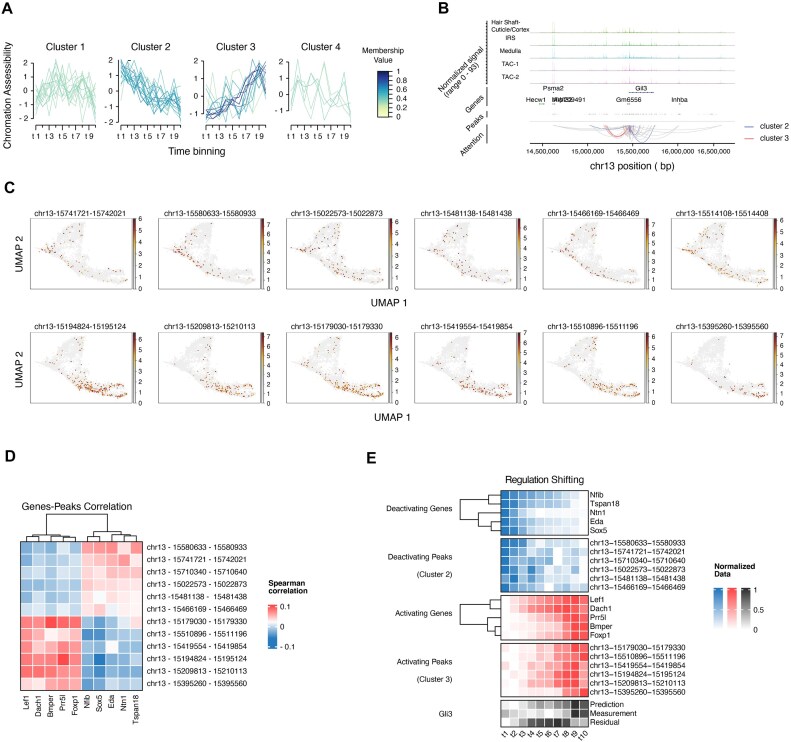
Regulatory mechanism of hair follicle maturation. (A) Four clusters of Gli3 peaks by soft cluster. (B) Link plot of peaks with high attention weight to Gli3. Peaks of clusters 2/3 are colored. (C) Chromatin-accessible state of 12 peaks within cluster 2 (top) or cluster 3 (bottom). (D) Spearman’s correlation between peaks in cluster 2 or cluster 3 and genes. (E) Similar transit pattern between peaks of 2 clusters and genes. Each row shows the normalized expression or accessibility score of a gene or peak.

In a previous study, Gli3 was identified as a highly connected transcriptional repressor with limited description [3]. In this study, we aim to elucidate the lagging mechanism of Gli3 and provide a comprehensive understanding of its role in hair follicle maturity. We utilize a prediction subtask to generate expression from chromatin state signal and find that the transition of chromatin accessibility may account for the delay of Gli3. After being filtered by attention weight, 49 peaks of Gli3 are soft clustered (see Fig. 3I and Methods) [37, 38]. As depicted in Fig. 4A and Supplementary Table S15, we determine 4 clusters, with clusters 2 and 3 having a higher number of peaks with high membership value (>0.5). Cluster 2 shows a downward trend in peak accessibility, while cluster 3 demonstrates an opposite fluctuation, suggesting that the peaks around Gli3 change in accessibility in a coordinated manner instead of independently opening or closing. To reduce noise, we further analyze 12 peaks from clusters 2 and 3 for their relationship with Gli3.

The majority of the 12 peaks are situated within 500 kb of Gli3’s transcriptional start site (as depicted in Fig. 4B) and are found to be accessible during the early or late differentiation stage (as shown in Fig. 4C, either at the top or bottom, respectively, and Supplementary Fig. S6c). To investigate the genes that impact accessibility of these 12 peaks, we perform Spearman correlation analysis between the peaks and genes to discover cluster-associated genes (see Methods). Our analysis reveals that Eda (*P* = 6.01 × 10^−4^), Nfib (*P* = 2.20×10^−4^), Sox5 (*P =* 6.67 × 10^−4^), Ntn1 (*P =* 4.38 × 10^−4^), and Tspan18 (*P* = 4.79 × 10^−4^) are the top 5 correlated genes for cluster 2, while Lef1 (*P =* 1.52 × 10^−4^), Prr5l (*P =* 1.43 × 10^−4^), Foxp1 (*P* = 4.72 × 10^−5^), Bmper (*P =* 1.64 × 10^−4^), and Dach1 (*P =* 2.85 × 10^−4^) are the top 5 correlated genes for cluster 3 (see Fig. 4D and Supplementary Table S16 for details). Notably, Nfib, Sox5, Lef1, Foxp1, and Dach1 are transcription factors. Our findings suggest that Gli3 may undergo a regulatory shift, and the peaks may either activate or deactivate in clusters 2 and 3, respectively, as shown in Fig. 4E, which corresponds to the observed time lag in Gli3 expression.

#### Cross-talk of multiple pathways commits cell differentiation

**Figure 5:**
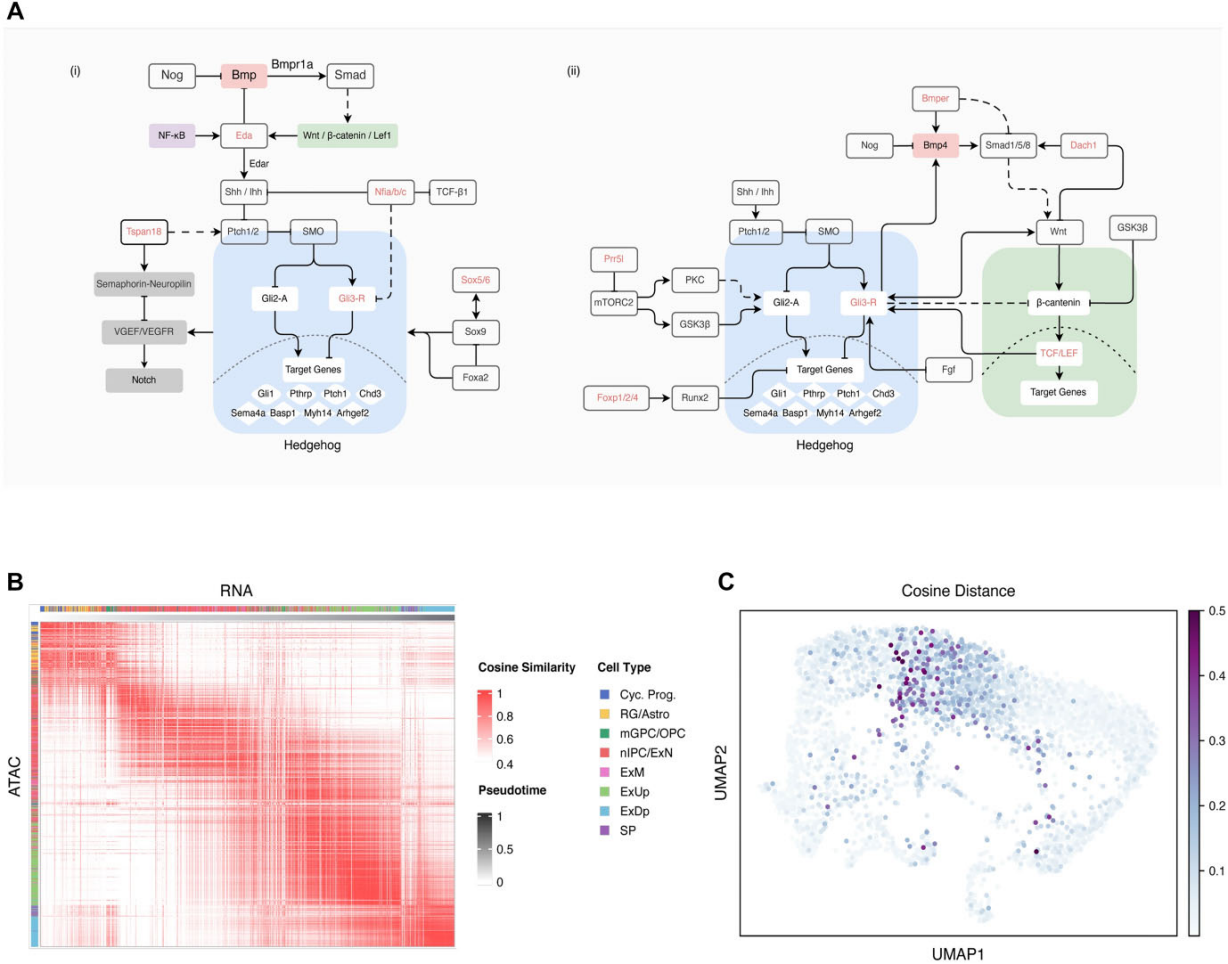
State transition of neonatal neurons in the human cortex. (A) Blueprint of Hh-centric multiple pathways involved in hair follicle development. Genes associated with peaks in cluster 2 or cluster 3 are highlighted in red. The background of pathways such as NF, Wnt, Bmp, and Hh are colored. (B) Cosine similarity between each cell from 2 modalities on the human cortex dataset is calculated. Cells are arranged chronologically. (C) UMAP visualization of cosine distance from matched cells between RNA and ATAC modalities.

Similar to other developmental processes [39–41], the differentiation of hair follicle stem cells is a highly regulated process that involves the interaction of multiple pathways, including Wnt, Bmp, Notch, and Hh, among others. These pathways act as positive or negative feedback loops, as described in previous studies [42, 43]. Using matched multimodal data, we employ Attune to recover a complex regulatory network by a chain rule from residual to peak and to other genes. Based on the discovery of cluster-associated genes, targets, and literature review, we propose a model for hair follicle maturity (see Fig. 5A).

During the early stages, when transient-amplifying cells predominate (peaks in cluster 2 and their associated genes), cell proliferation continues while the function of Gli3 is hindered directly or indirectly. Eda promotes the Hh pathway via the Wnt-Eda-Shh cascade [39, 44–46]. Sox9, which regulates GLI expression, can be enhanced by Sox5/6 [47–49], while Nfib and Nfia enable DNA-binding transcription activator activity and share many targets, including Gli3, Sox3, and Cdh2 [50–52]. These signals decrease as cell specification becomes more pronounced.

At t3–t4 time points, the expression of Lef1 and Dach1 increases (peaks in cluster 3 and their associated genes). TCF/LEF restricts Shh activity by binding to the enhancer of Gli3. Motif analysis also identifies peaks (chr13:15510896–15511196 and chr13:15395260–15395560) containing the LEF binding motif, which is consistent with previous research. Along with the Wnt pathway, Bmp [53–55], Foxp1–Runx2 [56], and Prr5l–mTORC2 [57–59] are implicated in the maintenance of the Hh pathway and Gli3. The downregulation of target genes, such as Basp1 [60, 61] and Chd3 [62], occurs upon inactivation of Hh, forming a feedback loop. In conclusion, the pattern shift of the chromatin state, initiated from chromatin potential, sheds new light on lineage priming and provides a basis for further developmental studies.

#### Embeddings facilitate discovery of key factors among active differentiating cells

The Attune algorithm not only ensures reliability for downstream tasks but also preserves biological signals in the high-dimensional space of cell embeddings. We delve into the embeddings between modalities in the fetal human cortex dataset (Supplementary Fig. S8a–b) and calculate cosine similarity for each pair of cells from RNA and ATAC embeddings, as depicted in Fig. 5B. Higher cosine similarity values are enriched in the diagonal, with the exception of the boundary between newborn neurons (nIPC/ExN), maturing neurons (ExM), and excitatory neurons in the upper layer (ExUp). The overall upward shift of blocks indicates the lag of RNA modality. These findings are supported by the observation of more cells with high cosine distance (1 – cosine similarity) at the junction of ExN, ExM, and ExUp regions in Fig. 5C and Supplementary Fig. S8c.

To investigate accessibility or expression events, we group cells with a cosine distance value above 0.1 (Supplementary Fig. S8d) and conduct differential expression analysis. Our analysis reveals overexpression of genes such as CNTNAP2, DCC, SLIT2, and KCND2 in this group (*P*-adjust < 0.01 and log fold change > 0.25). Notably, CNTNAP2 or DCC knockout models have been associated with abnormalities in neuronal migration [63, 64], while products of KCNH8, SLC24A2, and KCND2 involved in ion transportation have been shown to impact neuronal excitability and maturing [65].

## Discussion

Attune builds upon the principles of cross-modal contrastive learning and proposes a novel approach to tackle the problem of learning robust cell representations from multimodal data on a unit hypersphere. To this end, Attune leverages 2 teacher–student frameworks, achieving impressive performance in integration benchmarks without compromising biological signals. Drawing inspiration from the pretraining/fine-tuning paradigm, Attune’s embeddings can be effortlessly adapted to diverse downstream tasks via fine-tuning, as validated through cross-modal prediction and regulatory interaction inference tasks.

By juxtaposing cells from disparate modalities in a common space, Attune enables the detection of regulatory events. Among the 3 datasets of peripheral blood, skin, and cortex examined, Attune uncovers the relationships between chromatin accessibility and transcription features, disentangles the intricate network of lineage priming regulations, and identifies transcriptionally active cells. By expanding regulatory sequence along both feature spaces using chromatin potential and cross-modal attention, Attune provides fresh insights into the maturation of hair follicles, which entail multiple pathways. Furthermore, Attune pinpoints the local inconsistencies in embeddings in the human cortex dataset and posits the occurrence of swift transcriptional activities in nascent neurons.

An upsurge in experimental protocols combining dual-modalities, trio-modalities, and other modalities highlights the inevitability and indispensability of matched sequencing [6, 66]. Such a surge poses a challenge in scaling methods to suit increased modalities and cells. Attune rises to this challenge by employing a lightweight model framework and an optimized input structure, which facilitates the easy scaling of Attune to support millions of cell atlases. By combining contrastive loss between specified modalities, Attune can handle additional modalities of data and explore intermodal connections with greater flexibility. Additionally, Attune’s embeddings present a unique opportunity for portraying differentiation trajectories, which complements existing methods for trajectory analysis [67].

While Attune has demonstrated its potential across multiple scenarios, its performance verification with limited data remains insufficient. Acknowledging this limitation, we aim to augment the validated dataset in subsequent stages to enhance model robustness and validate its applicability across diverse multiple modalities.

In conclusion, Attune constitutes a potent paradigm for analyzing matched multimodal single-cell data, enabling exploration of intermodal relations and unearthing the mechanisms driving complex biological phenomena at a single-cell resolution.

## Methods

### Input data and preprocessing

The Attune model takes expression (gene count) and accessibility (peak count) matrices from matched multimodal scRNA-seq and scATAC-seq as input data. For scRNA-seq data, genes expressed in fewer than 5% of cells were filtered out. We used SCANPY [68] to normalize each cell count to 10,000 read counts before the logarithm. Additionally, sex chromosome genes were removed, and 2,000 highly variable genes (HVGs) were selected based on the experiments depicted in Fig. 2D, providing a reasonable compromise. This choice allows us to achieve satisfactory performance while managing the computational cost and ensuring the overall stability and efficiency of the model. For scATAC-seq data, peaks accessed in fewer than 5% of cells were filtered out. Peaks from sex chromosomes were also filtered out. A complete list of all data used in the study is provided in Supplementary Table S1.

### Input encoding scheme

The normalized expression and accessibility matrices were encoded in the TensorFlow Record (TF-record) format. The scRNA-seq data were encapsulated in 1 TF-record file, with “gene index” and “gene count” fields, while the scATAC-seq data were encapsulated in another file, with “peak index” and “peak count” fields.

### Overview of model architecture

As illustrated in Fig. 1B, the input of Attune is a multimodal dataset, also called joint profiling data, which contains information from 2 modalities, and the cells of the 2 modalities are paired. Attune is designed based on joint profiling data, which considers the pairing information. We consider the cells in paired scRNA-seq and scATAC-seq as positive pairs (*m* and ${{m}^ + }$ is a pair of positive samples).

The overarching model architecture encompasses the Attune pretraining model, which comprises 2 asymmetric teacher–student networks, along with 2 modules dedicated to downstream tasks: the cross-modal prediction module and the transformer-based peak–gene interaction module. Attune leverages separate teacher–student networks to learn cell representations from scRNA-seq and scATAC-seq, respectively, through cross-modal contrastive learning. The teacher network, designed to be more complex, employs a hierarchical attention mechanism [69], while the student network uses a simpler dense operation. The representation of cells in 2 modalities (scRNA-seq and scATAC-seq) can be learned by maximizing the consistency between positive pairs in the embedding space. The representations from both modalities are then projected into a common space.

We adapted the pretrained Attune model to 3 downstream tasks: inference of gene–peak interaction and cross-modal prediction and differentiation analysis. Reconstructing regulatory events requires the model to reconstruct the correspondence between accessible chromatin and gene expression and ascertain which chromatin regions are responsible for the change in gene expression across cells. To accomplish this, we utilized a transformer-based decoder that captures multimodal cross-attention, thereby establishing the link between peaks and genes. For cross-modal prediction, Attune was fine-tuned via an MLP to predict gene expression and further identify temporal differences in expression accessibility and transcription for differentiation analysis.

### Teacher network

For 2 kinds of single-cell data (scRNA-seq and scATAC-seq), we designed 2 teacher networks (RNA teacher network and ATAC teacher network) to learn fine-grained representations, respectively. The RNA teacher network accepts ${{X}_{\textit{indices}}}\epsilon {{\mathbb{R}}^G}$ and ${{X}_{\textit{counts}}}\epsilon {{\mathbb{R}}^G}$ as input, where G denotes the number of genes. ${{X}_{\textit{indices}}}$ represents gene indices, with a dimension of (N × G), and ${{X}_{\textit{counts}}}$ represents the value of gene expression, with a dimension of (N × G). N is the number of cells. For ATAC, teacher network accepts an input of ${{Y}_{\textit{indices}}}\epsilon {{\mathbb{R}}^P}$ and ${{Y}_{\textit{counts}}}\epsilon {{\mathbb{R}}^P}$, where P denotes the number of peaks. ${{Y}_{\textit{indices}}}$ represents peak indices, with a dimension of (N × P), and ${{Y}_{\textit{counts}}}$ represents the value of peak counts, with a dimension of (N × P). Each gene within a cell is represented by $i \epsilon {{\mathbb{Z}}^G}$, and each peak is represented by $j\epsilon {{\mathbb{Z}}^P}$.

In the teacher network, the embedding layer maps discrete inputs such as genes and peaks to continuous vector space. The input of the embedding layer of the RNA teacher network is each row of ${{X}_{\textit{indices}}}$, that is, an integer sequence ${{x}_1}$, ${{x}_2}$, …, ${{x}_G}$, where each ${{x}_i}\ $ represents the index of a gene. The purpose of the embedding layer is to map these integers into a dense vector $gene\ \textit{embe}{{d}_i}\in {{\mathbb{R}}^d}$ (a ${\mathrm{d}}$-dimensional embedding vector corresponding to ${{x}_i}$). The embedding layer can be represented by a matrix ${{E}_{RNA}}\in {{\mathbb{R}}^{G \times d}}$, where G is the number of genes, and ${\mathrm{d}}$ is the dimension of the embedding. ${{E}_{RNA}}[ {{{x}_i}} ]$ represents the extraction of the ${{x}_i}$ row (i.e., the embedding vector corresponding to the gene ${\mathrm{i}}$) from the matrix ${{E}_{RNA}}$ through a table lookup operation (eq. 1). Then, the RNA expression of gene ${\mathrm{i}}$ ($coun{{t}_i}\in \mathbb{R}$) from ${{X}_{\textit{counts}}}$ is element-wise cross-multiplied with its embedding vector ($gene\ \textit{embe}{{d}_i}$) to obtain the weighted embedding vector $gene\ \textit{hidde}{{n}_i}\in {{\mathbb{R}}^d}$ (eq. 2). For scATAC-seq data, we use ${{y}_j}$ to represent the index of a peak in ${{Y}_{\textit{indices}}}$, and the embedding layer of the ATAC teacher network is represented by a matrix ${{E}_{\textit{ATAC}}}\in {{\mathbb{R}}^{P \times d}}$, where P is the number of peaks. The embedding vector ${\mathrm{\ }}\textit{peak}\ \textit{embe}{{d}_j}$ of peak ${\mathrm{j}}$ is obtained by a table lookup operation (eq. 3). The peak count of peak ${\mathrm{j}}$ ($coun{{t}_j}\epsilon\mathbb{R}$) from ${{Y}_{\textit{counts}}}$ is element-wise cross-multiplied with its embedding vector ($peak\ \textit{embe}{{d}_j}$) to obtain the weighted embedding vector $peak\ \textit{hidde}{{n}_j}\in {{\mathbb{R}}^d}$ (eq. 4).


(1) \begin{eqnarray*}
\textit{gene}\ \textit{embe}{{d}_i} = {{E}_{RNA}}\left[ {{{x}_i}} \right]
\end{eqnarray*}

(2) \begin{eqnarray*}
\textit{gene}\ \textit{hidde}{{n}_i} = \textit{gene}\ \textit{embe}{{d}_i} \times \textit{coun}{{t}_i}
\end{eqnarray*}

(3) \begin{eqnarray*}
\textit{peak}\ \textit{embe}{{d}_j} = {{E}_{\textit{ATAC}}}\left[ {{{y}_j}} \right]
\end{eqnarray*}

(4) \begin{eqnarray*}
\textit{peak}\ \textit{hidde}{{n}_j} = \textit{peak}\ \textit{embe}{{d}_j} \times \textit{coun}{{t}_j}
\end{eqnarray*}

Then we use the attention mechanism to aggregate gene or peak embeddings. The input ${\mathrm{\ }}\textit{gene}{\mathrm{\ }}\textit{hidde}{{n}_i}$ is passed through a multilayer perceptron with 1 hidden layer and a nonlinear tanh transformation. A cellular context vector $u\epsilon{{\mathbb{R}}^d}$ then applies the dot product to $gene{\mathrm{\ }}\textit{hidde}{{n}_i}$, using the softmax operation to obtain $gene{\mathrm{\ }}\textit{attentio}{{n}_i}\in {{\mathbb{R}}^d}$ (eq. 5). The cell context vector *u* serves as an intermediate variable in computing hierarchical attention, which is a weighted value on 128-dimensional embeddings. Aggregation is then applied to the genes’ vectors $gene{\mathrm{\ }}\textit{hidde}{{n}_i}$ through weighted summation by $gene{\mathrm{\ }}\textit{attentio}{{n}_i}$, to obtain aggregated vectors, $RNA{\mathrm{\ }}\textit{hidden}$ (eq. 6), with N*d dimension. The same process is applied to scATAC-seq data, as shown in eqs. (7) and (8).


(5) \begin{eqnarray*}
\textit{gene}{\mathrm{\ }}\textit{attentio}{{n}_i} = {\mathrm{softmax}}\left( {\tanh \left( {\textit{gene}{\mathrm{\ }}\textit{hidde}{{n}_i}} \right) \cdot u} \right)
\end{eqnarray*}

(6) \begin{eqnarray*}
RNA{\mathrm{\ }}\textit{hidden} = {\mathrm{\ }}\mathop \sum \limits_i \left( {\textit{gene}{\mathrm{\ }}\textit{attentio}{{n}_i} \times \textit{gene}{\mathrm{\ }}\textit{hidde}{{n}_i}} \right)
\end{eqnarray*}

(7) \begin{eqnarray*}
\textit{peak}{\mathrm{\ }}\textit{attentio}{{n}_j} = {\mathrm{softmax}}\left( {\tanh \left( {\textit{peak}{\mathrm{\ }}\textit{hidde}{{n}_j}} \right) \cdot u} \right)
\end{eqnarray*}

(8) \begin{eqnarray*}
\textit{ATAC}{\mathrm{\ }}\textit{hidden} = {\mathrm{\ }}\mathop \sum \limits_j \left( {\textit{peak}{\mathrm{\ }}\textit{attentio}{{n}_j} \times \textit{peak}{\mathrm{\ }}\textit{hidde}{{n}_j}} \right)
\end{eqnarray*}

We apply the attention mechanism output to feed into a batch normalization layer, followed by a dropout layer. Then a dense layer with ReLU activation projects to the final output of the RNA teacher network, $Z_{\textit{teacher}}^{RNA}\in {{\mathbb{R}}^d}$ (eq. 9), and the final output of the ATAC teacher network, $Z_{\textit{teacher}}^{\textit{ATAC}}\in {{\mathbb{R}}^d}$ (eq. 10).


(9) \begin{eqnarray*}
Z_{\textit{teacher}}^{RNA} = \textit{Dense}\left( {RNA{\mathrm{\ }}\textit{hidden}} \right)
\end{eqnarray*}

(10) \begin{eqnarray*}
Z_{\textit{teacher}}^{\textit{ATAC}} = \textit{Dense}\left( {\textit{ATAC}{\mathrm{\ }}\textit{hidden}} \right)
\end{eqnarray*}

### Student network

We also designed 2 student networks (RNA student network and ATAC student network) to learn coarse-grained representations, respectively. The student network accepts only ${{X}_{\textit{counts}}}\in {{\mathbb{R}}^G}$ or ${{Y}_{\textit{counts}}}\epsilon {{\mathbb{R}}^P}$, then passes a batch normalization layer, followed by a dropout layer and a dense layer, with ReLU activation projects to the final output of the RNA student network $Z_{\textit{student}}^{RNA}\in {{\mathbb{R}}^d}$ (eq. 11). The final output of the ATAC student network is $Z_{\textit{student}}^{\textit{ATAC}}\epsilon{{\mathbb{R}}^d}$ (eq. 12).


(11) \begin{eqnarray*}
Z_{\textit{student}}^{RNA} = \textit{Dense}\left( {{{x}_{\textit{counts}}}} \right)
\end{eqnarray*}

(12) \begin{eqnarray*}
Z_{\textit{student}}^{\textit{ATAC}} = \textit{Dense}\left( {{{y}_{\textit{counts}}}} \right)
\end{eqnarray*}

### Cross-modal contrastive loss

Contrast learning is implemented by the explicit comparison of the *d*-dimensional embedding (where d = 128 by default) of a cell on 2 modalities on a unit hypersphere. Positive sample pairs are created by taking 2 modal representations of a cell and pulling them together, while negative samples are created by taking different cells and widening the distance between them. Four different embeddings—$Z_{\textit{student}}^{\textit{ATAC}}$, $Z_{\textit{student}}^{RNA}$, $Z_{\textit{teacher}}^{\textit{ATAC}}$, $Z_{\textit{teacher}}^{RNA}$—are obtained using 2 independent asymmetric teacher–student networks.

Assume the embedding is obtained by the teacher network, and the cosine similarity with L2 regularization of the 2 given embeddings (the embedding under the same network structure) is defined by eqs. (13) and (14). The positive pair is $cel{{l}_m}$ (whose embedding is $z_{\textit{teache}{{r}_m}}^{RNA}\in Z_{\textit{teacher}}^{RNA}$) and $cel{{l}_{{{m}^ + }}}$ (whose embedding is $z_{\textit{teache}{{r}_{{{m}^ + }}}}^{\textit{ATAC}}\epsilon Z_{\textit{teacher}}^{\textit{ATAC}}$). The NT-Xent loss represents the normalized temperature-scaled cross-entropy loss, as formalized by eq. (15), where *m* and ${{m}^ + }$ is a pair of positive samples. We randomly sample a mini-batch of N cells and compute a NT-Xent loss on pairs of cross-modal examples derived from the mini-batch, resulting in 2 N data points. Given a positive pair, the other 2(N − 1) cross-modal examples within a mini-batch are treated as negative examples. The calculation process of NT-Xent loss for the embedding obtained by the student network is the same; see eqs. (16)–(18). For the full pretraining objective of the Attune model, see eq. (19).


(13) \begin{eqnarray*}
{{s}_{\alpha ,\beta }} = sim\left( {{{z}_{\textit{teache}{{r}_\alpha }}},{{z}_{\textit{teache}{{r}_\beta }}}} \right)
\end{eqnarray*}

(14) \begin{eqnarray*}
s_{\alpha ,\beta }^ + = sim\left( {{{z}_{\textit{teache}{{r}_\beta }}},{{z}_{\textit{teache}{{r}_\alpha }}}} \right)
\end{eqnarray*}

where $sim( {{{h}_1},{{h}_2}} )$ is defined as


(15) \begin{eqnarray*}
sim\left( {{{h}_1},{{h}_2}} \right) &=& \frac{{h_1^T{{h}_2}}}{{\tau \|{{h}_1}\|\|{{h}_2}\|}}\\
{{\mathcal{L}}_{\textit{teacher}}} &=& \frac{1}{{2N}}\mathop \sum \limits_{m = 1}^N \left[ {\ell \left( {m,{{m}^ + }} \right) + \ell \left( {{{m}^ + },m} \right)} \right]
\end{eqnarray*}

where ${\ell }( {m,{{m}^ + }} )$ is defined as


\begin{eqnarray*}
\ell (m,{m}^{+}) = - log \frac{\text{exp}({s}_{m,{m}^{+}})}
{\sum_{k = 1}^{2N} {{II}}_{[{k \neq m}]}[\exp ({s}_{k,m}) + {\mathrm{exp}}\left({s}_{k,{{m}^{+}}}\right)]}
\end{eqnarray*}

where $\ell ( {{{m}^ + },m} )$ is defined as


\begin{eqnarray*}
\ell \left( {{{m}^ + },m} \right) = \ - log\frac{{{\mathrm{exp}}\left( {s_{m,{{m}^ + }}^ + } \right)}}{{\mathop \sum \nolimits_{k = 1}^{2N} {{II}}_{[k \neq {m}^{+}]}\left[ {\exp \left( {s_{k,{{m}^ + }}^ + } \right) + {\mathrm{exp}}\left( {s_{k,m}^ + } \right)} \right]}}
\end{eqnarray*}

(16) \begin{eqnarray*}
{{s}_{\alpha ,\beta }} = sim\left( {{{z}_{\textit{studen}{{t}_\alpha }}},{{z}_{\textit{studen}{{t}_\beta }}}} \right)
\end{eqnarray*}

(17) \begin{eqnarray*}
s_{\alpha ,\beta }^ + = sim\left( {{{z}_{\textit{studen}{{t}_\beta }}},{{z}_{\textit{studen}{{t}_\alpha }}}} \right)
\end{eqnarray*}

(18) \begin{eqnarray*}
{{\mathcal{L}}_{\textit{student}}} = \frac{1}{{2N}}\mathop \sum \limits_{m = 1}^N \left[ {\ell \left( {m,{{m}^ + }} \right) + \ell \left( {{{m}^ + },m} \right)} \right]
\end{eqnarray*}

(19) \begin{eqnarray*}
{{\mathcal{L}}_{\textit{pretrain}}} = \frac{{{{\mathcal{L}}_{\textit{teacher}}} + \ {{\mathcal{L}}_{\textit{student}}}}}{2}
\end{eqnarray*}

where τ is the adjustable temperature coefficient, which can be used to scale the degree of pushing apart negative samples.

### Joint representation and UMAP visualization

Contrast learning can integrate cells from different modalities together. Meanwhile, to join the cell embeddings of 2 modalities together, we concatenate $Z_{\textit{teacher}}^{\textit{ATAC}}$ to $Z_{\textit{teacher}}^{RNA}$ to get the joint embedding matrix ${{Z}_{\textit{joint}}}\in {{\mathbb{R}}^d}$, with 2N*d dimension (eq. 20). Cell embeddings are visualized by UMAP using SCANPY.


(20) \begin{eqnarray*}
{{Z}_{\textit{joint}}} = {\mathrm{\ }}\textit{Concatenate}\left( {Z_{\textit{teacher}}^{RNA},Z_{\textit{teacher}}^{\textit{ATAC}}} \right)
\end{eqnarray*}

#### Inference of gene–peak interaction

To recover gene–peak interaction, Attune employs contrastive learning as a pretraining procedure, followed by a transformer decoder to model the relationship between peaks and genes. The transformer is a deep neural network structure for sequence modeling. The self-attention mechanism establishes attention connections between each token in a sequence, so the embedding of each token contains implicit context. Meanwhile, the cross-attention mechanism establishes attention connections between tokens in 2 sequences, enabling the model to extract the dependency between tokens in the 2 sequences. Both self-attention and cross-attention are adopted in our model. Self-attention captures intramodality interaction, such as gene–gene relationships, while cross-attention models intermodality interaction, which is the peak–gene relationship.

The input sequences of the ransformer are $gene{\mathrm{\ }}\textit{hidde}{{n}_i}$ (N*G*d dimension) and $peak{\mathrm{\ }}\textit{hidde}{{n}_j}$ (N*P*d dimension). First, a Classify (CLS) token is inserted at the beginning of a input sequence. It can also be understood as a weighted average of each token in a sequence. The embedding of the CLS token is denoted as $gene{\mathrm{\ }}\textit{hidde}{{n}_{CLS}}\epsilon {{\mathbb{R}}^d}$ and $peak{\mathrm{\ }}\textit{hidde}{{n}_{CLS}}\epsilon {{\mathbb{R}}^d}$. Then, self-attention is applied to $gene{\mathrm{\ }}\textit{hidde}{{n}_i}$:

Step 1: $gene{\mathrm{\ }}\textit{hidde}{{n}_i}$ is fed into a 3-multilayer perceptron to get vectors $Q\epsilon {{\mathbb{R}}^d}$, $K\epsilon {{\mathbb{R}}^d}$, $V{\mathrm{\ }}\epsilon {{\mathbb{R}}^d}$.
Step 2: *Q* applies the dot product to *K*, using the softmax operation to obtain $Attention{\mathrm{\ }}\textit{weigh}{{t}_i}$, $Attention{\mathrm{\ }}\textit{weigh}{{t}_i}\epsilon {{\mathbb{R}}^{G + 1}}$, with a N*(G+1)*(G+1) dimension. $self{\mathrm{\ }}\textit{gene}{\mathrm{\ }}\textit{hidde}{{n}_i}\epsilon {{\mathbb{R}}^d}$ is defined as (21) with a N*(G+1) *d dimension.
  (21) \begin{eqnarray*}
\textit{self}\ \textit{gene}\ \textit{hidde}{{n}_i} = \textit{softmax}\left( {\frac{{Q{{K}^T}}}{{\sqrt d }}} \right)V
\end{eqnarray*}

Finally, the cross-attention mechanism is applied to $peak{\mathrm{\ }}\textit{hidde}{{n}_j}$ and $self{\mathrm{\ }}\textit{gene}{\mathrm{\ }}\textit{hidde}{{n}_i}$:

$cross{\mathrm{\ }}\textit{gene}{\mathrm{\ }}\textit{hidde}{{n}_i}\epsilon {{\mathbb{R}}^d}$ is defined as (22) with a dimension of N*(G+1) *d.


(22) \begin{eqnarray*}
\textit{cross}\ \textit{gene}\ \textit{hidde}{{n}_i} = \textit{softmax}\left( {\frac{{Q{{K}^T} + \textit{Attention}\ \textit{mask}}}{{\sqrt d }}} \right)V
\end{eqnarray*}

where *Q* is $self{\mathrm{\ }}\textit{gene}{\mathrm{\ }}\textit{hidde}{{n}_i}$ and *K, V* are $peak{\mathrm{\ }}\textit{hidde}{{n}_j}$. $Attention\ \textit{mask}$ (with N*(G+1) *(P+1) dimension) is defined in the “Attention mask” section. Cross-attention weight is defined as (23):


(23) \begin{eqnarray*}
\textit{Cross}\ \textit{attention}\ \textit{weight} = \textit{softmax}\left( {\frac{{Q{{K}^T} + \textit{Attention}\ \textit{mask}}}{{\sqrt d }}} \right)
\end{eqnarray*}

### Training objectives

We train the transformer with 2 objective functions: contrastive loss between $gene{\mathrm{\ }}\textit{hidde}{{n}_{CLS}}$ and $peak{\mathrm{\ }}\textit{hidde}{{n}_{CLS}}$, with RNA–ATAC modality matching loss. $gene{\mathrm{\ }}\textit{hidde}{{n}_{CLS}}$ and $peak{\mathrm{\ }}\textit{hidde}{{n}_{CLS}}$ are CLS tokens in different modalities with a N*1*d dimension. They learn the weighted average embedding representing the entire genes or peaks. The purpose of comparing the 2 embeddings is to shorten the distance between the matched RNA–ATAC pairs globally. The calculation process of the contrastive loss of CLS tokens is described in eqs. (24)–(26).


(24) \begin{eqnarray*}
{{s}_{\alpha ,\beta }} = sim\left( {\textit{gene}\ \textit{hidde}{{n}_{CL{{S}_\alpha }}},\textit{peak}\ \textit{hidde}{{n}_{CL{{S}_\beta }}}} \right)
\end{eqnarray*}

(25) \begin{eqnarray*}
s_{\alpha ,\beta }^ + = sim\left( {\textit{peak}\ \textit{hidde}{{n}_{CL{{S}_\beta }}},\textit{gene}\ \textit{hidde}{{n}_{CL{{S}_\alpha }}}} \right)
\end{eqnarray*}

where $sim( {{{h}_1},{{h}_2}} )$ is defined as


\begin{eqnarray*}
sim\left( {{{h}_1},{{h}_2}} \right) = \frac{{h_1^T{{h}_2}}}{{\tau \|{{h}_1}\|\|{{h}_2}\|}}
\end{eqnarray*}

(26) \begin{eqnarray*}
{{\mathcal{L}}_{CLS}} = \frac{1}{{2N}}\mathop \sum \limits_{m = 1}^N \left[ {\ell \left( {m,{{m}^ + }} \right) + \ell \left( {{{m}^ + },m} \right)} \right]
\end{eqnarray*}

where $\ell ( {m,{{m}^ + }} )$ is defined as


\begin{eqnarray*}
\ell \left( {m,{{m}^ + }} \right) = \ - log\frac{{{\mathrm{exp}}\left( {{{s}_{m,{{m}^ + }}}} \right)}}{{\mathop \sum \nolimits_{k = 1}^{2N} {{{II}}_{\left[ {k \neq m} \right]}}\left[ {\exp \left( {{{s}_{k,m}}} \right) + {\mathrm{exp}}\left( {{{s}_{k,{{m}^ + }}}} \right)} \right]}}
\end{eqnarray*}

where $\ell ( {{{m}^ + },m} )$ is defined as


\begin{eqnarray*}
\ell \left( {{{m}^ + },m} \right) = \ - log\frac{{{\mathrm{exp}}\left( {s_{m,{{m}^ + }}^ + } \right)}}{{\mathop \sum \nolimits_{k = 1}^{2N} {{{II}}_{\left[ {k \neq {{m}^ + }} \right]}}\left[ {\exp \left( {s_{k,{{m}^ + }}^ + } \right) + {\mathrm{exp}}\left( {s_{k,m}^ + } \right)} \right]}}
\end{eqnarray*}

where τ is the adjustable temperature coefficient, which can be used to scale the degree of pushing apart negative samples.

For the RNA–ATAC modality matching loss, the transformer is given a batch of matched RNA–ATAC pairs (positive pairs) or mismatched RNA–ATAC pairs (negative pairs). The network’s goal is to identify whether a given pair is positive or negative. We feed the transformer with $gene{\mathrm{\ }}\textit{hidde}{{n}_i}$ and $peak{\mathrm{\ }}\textit{hidde}{{n}_j}$ pairs, and the probability of a positive pair and the probability of a negative pair are set equal. A classifier is added on top of the CLS token’s embedding $cross{\mathrm{\ }}\textit{gene}{\mathrm{\ }}\textit{hidde}{{n}_{CLS}}\epsilon {{\mathbb{R}}^d}$ to predict a binary label $gt$, where $gt$ is a 2-dimensional one-hot vector representing the ground-truth label. ${{p}^{\textit{match}}}$ is the probability of prediction. RNA–ATAC modality matching loss is defined in eq. (27), and the full training objective of the transformer is presented in eq. (28).


(27) \begin{eqnarray*}
{{\mathcal{L}}_{\textit{match}}} = {{{\mathbb{E}}}_{\left( {RNA,\textit{ATAC}} \right)\sim D}}CE\left( {{{p}^{\textit{match}}}\left( {RNA,\textit{ATAC}} \right),gt} \right)
\end{eqnarray*}

(28) \begin{eqnarray*}
{{\mathcal{L}}_{\textit{interaction}}} = {{\mathcal{L}}_{CLS}} + \ {{\mathcal{L}}_{\textit{match}}}
\end{eqnarray*}

### Global cross-attention weight extraction

$Cross\ \textit{attention}\ \textit{weight}$ contains 2 global CLS tokens’ attention weight, $CLS{\mathrm{\ }}\textit{Attention}{\mathrm{\ }}\textit{weight}_{}^{RNA}$ (with N*(P+1) dimension) and $CLS{\mathrm{\ }}\textit{Attention}{\mathrm{\ }}\textit{weight}_{}^{\textit{ATAC}}$ (with N*(G+1) dimension). $Global{\mathrm{\ }}\textit{Attention}{\mathrm{\ }}\textit{weight}$ matrix (with G*P dimension) is defined as the dot product of $CLS{\mathrm{\ }}\textit{Attention}{\mathrm{\ }}\textit{weight}_{}^{RNA}$ and $CLS{\mathrm{\ }}\textit{Attention}{\mathrm{\ }}\textit{weight}_{}^{\textit{ATAC}}$.

### Modality prediction network

For the modality prediction task, a modality prediction network is fine-tuned on the Attune pretrained model to predict the RNA expression level. The network uses a relatively simple regression model, a multilayer perceptron. The input of the network is $Z_{\textit{student}}^{\textit{ATAC}}\epsilon {{\mathbb{R}}^d}$, the number of hidden layer units is set to 1,000, and the number of units in the last layer is set to G (the number of genes). The output of the network is the predicted value of gene counts, $X_{\textit{counts}}^{\textit{pred}}\epsilon {{\mathbb{R}}^G}$. The loss function of modality prediction network is defined as eq. (29).


(29) \begin{eqnarray*}
{{\mathcal{L}}_{\textit{prediction}}} = MSE\left( {{{X}_{\textit{counts}}},X_{\textit{counts}}^{\textit{pred}}} \right)
\end{eqnarray*}

### Hyperparameter tuning

The learning rate in contrastive pretraining varies from 1 × 10^−4^ to 1 × 10^−6^ using Adam optimizer training for 20 epochs. For the transformer, it trains for 5 epochs, whereas for the modality prediction network, it trains for 40 epochs. The temperature coefficient in NT-Xent loss is set to 0.1, the mini-batch size is set to 32, and the dimension of the embedding is 128. Comparative experiments are detailed in Supplementary Tables S5 and S6.

### Metrics

Integration endeavors are assessed through an array of metrics, including mean average precision (MAP), cell-type ASW, neighbor consistency (NC), Seurat alignment score (SAS), Batch ASW, graph connectivity (GC), biology conservation, omics mixing, overall integration score, and FOSCTTM.

*The mean average precision (MAP)* furnishes a measure of the congruity between cell types in neighboring cells, thereby quantifying the accuracy of clustering outcomes with respect to cell-type assignments [10].

*Cell-type ASW*. To evaluate the integration outputs pertaining to cell types, cell-type ASW affords an assessment of the silhouette of cell-type labels, suitably scaled to a value between 0 and 1 [18].

*Batch ASW* evaluates the integration among multimodalities by computing cell modality labels, also scaled between 0 and 1 [18].

*NC* measures the degree of intercellular neighbor retention after integrating multimodal data, ranging from 0 to 1, where higher values indicate better preservation [70].

*SAS* calculates the alignment score to assess how well 2 or more modalities have been aligned, with values ranging from 0 to 1, where higher values indicate better integration among modalities [71].

*GC* evaluates the proximity of cells with the same identity across different modalities in the embedding. The GC ranges from 0 to 1, with higher values indicating better integration [10, 19].

*FOSCTTM* measures the accuracy of modal alignment at the single-cell level in paired cells, with a range from 0 to 1, where lower values indicate higher accuracy. Studies like GLUE [10] and MMD-MA [72] have utilized FOSCTTM to assess performance.

*Biology conservation* is evaluated through MAP, cell-type ASW, and NC, which collectively assess the biological conservation of integration. These metrics are min-max scaled, and their average is calculated as a single metric for biological conservation, as per eq. (30) [10].

*Omics mixing* is evaluated using SAS, batch ASW, and GC, which collectively assess the mixing performance of multimodalities. These metrics are also min-max scaled, and their average is calculated as a single metric for omics mixing, as per eq. (31) [10].

*The overall integration score* is computed as an overall weighted average of omics mixing and bio-conservation scores, as per eq. (32) [10, 19].


(30) \begin{eqnarray*}
\textit{Biology}\ \textit{conservation} = \ \frac{{\textit{scale}\left( {MAP} \right) + \textit{scale}\left( {\textit{Cell}\ \textit{type}\ ASW} \right) + \textit{scale}\left( {NC} \right)}}{3}\\
\end{eqnarray*}

(31) \begin{eqnarray*}
\textit{Omics}\ \textit{mixing} = \ \frac{{\textit{scale}\left( {SAS} \right) + \textit{scale}\left( {\textit{Omics}\ \textit{layerASW}} \right) + \textit{scale}\left( {GC} \right)}}{3}\\
\end{eqnarray*}

(32) \begin{eqnarray*}
\textit{Overall}\ \textit{integratin}\ \textit{score} = \ 0.6 \times \textit{Biology}\ \textit{conservation} + 0.4 \times \textit{Omics}\ \textit{mixing}\\\end{eqnarray*}

Various metrics are employed to evaluate the proposed solution in modality prediction. These metrics include the RMSE, gene-wise Pearson correlation coefficient, and gene-wise Spearman correlation coefficient. The RMSE serves to appraise the precision of RNA expression prediction across individual cells [19], while gene-wise Pearson or Spearman correlation coefficients gauge the average per-gene correlation in Polarbear [23] and BABEL [9].

### Evaluation on inference of regulatory interaction

PCHi-C enables identification of long-range interactions between gene promoters and regulatory elements such as enhancers and other potential regulatory elements. Promoter interactomes are highly cell type specific, and interacted regions quantitatively contribute to gene expression [27, 73]. To demonstrate the potential of cross-modal association, we utilize the PCHi-C dataset of human primary hematopoietic cells. With the aim of consistency, only common cell types in the 10x Multiome and PCHi-C datasets, including T cells, B cells, and monocytes, are considered for the comparison of different methods. Coordinates of interactions from PCHi-C binding matrix whose CHiCAGO interaction scores pass a cutoff of 5 in at least one cell type are lifted over [74] to Genome Reference Consortium Human Build 38 and ordered based on the distance between the midpoint of baited regions and other ends. The distance statistics of interactions are depicted in Fig. 3A. Following the guidelines established in GLUE [10], we generate a truth set of peak–gene pairs supported by PCHi-C. These rules consider the proximity (within 1 kb) of the gene promoter to a bait fragment and the peak’s proximity (within 1 kb) to the other-end fragment, along with significant interaction identified in PCHi-C. By considering the distance statistics of the PCHi-C data and controlling for noise introduced by abundant distal regions, we explore a range of gene–peak distances from 150 to 1,500 kb (Supplementary Fig. S4a) and determine 1,200 kb as the threshold for all subsequent experiments.

Specifically, during the calculation of the transformer module, we focus on the relationship between the gene promoter and peaks within a 1.2-Mb region surrounding it. By masking the peaks outside this region, we encourage the transformer module to prioritize cross-modal attention within the adjacent regions of the gene. Attention weights are calculated for each gene–peak pair within 1,200 kb, and the “sklearn.metrics.roc_auc_score” function is utilized to assess whether the attention weights can reflect the promoter interactome.

### Differential expression analysis and gene ontology enrichment

We use the “FindAllMakers” function of the Seurat (RRID:SCR_007322) [7] package to identify differentially expressed genes (DEGs) within each cell type (one versus others, *P*-adjust < 0.01, log fold change > 0.25). The top 10 and bottom 10 genes are chosen as top DEGs. Collection of gene sets (GO:BP in C5 category) from the Molecular Signatures Database (MSigDB) [75] is used for overpresentation analysis by clusterProfiler (RRID:SCR_016884) [76]. We keep an ontology with a smaller *P* value when the geneID is repeated.

### Pseudotime inference

We focus on the differentiation of transient-amplifying cells and select cell types, including transit-amplifying cells, inner root sheath, medulla, and hair shaft, from the SHARE-seq dataset of mouse skin, leading to 6k cells. Ten topics are then determined by cisTopic [77] using chromatin accessibility data. The default parameters are used, except for burin = 120 and iterations = 150 in the “runModels” functions. The *z* score is then computed by the “modelMatSelection” function as an input of Palantir [78] for generating diffusion maps and pseudotime with n_components = 10 of the “run_diffusion_maps” function.

### Residual of modalities

The residual of each gene is calculated from normalized predicted RNA counts based on ATAC minus normalized measured RNA counts. We evaluate trends with pseudotime using a generalized additive model and filter them based on standard deviation. We use the “argrelextrema” function in the “scikit-learn” package to define the gene expression pattern.

### Motif and regulon analysis

The JASPAR CORE Vertebrata 2022 database (RRID:SCR_003030) [79] is selected for motif matching, which contains 841 motifs. We set the *P* cutoff to 5 × 10^−5^ for filtering motifs. To illustrate the regulatory network, we use SCENIC (RRID:SCR_017247) [36], a workflow that exploits coexpression between genes and transcription factors, to analyze regulons in the SHARE-seq dataset. All modules are kept in the step of regulon prediction (add a parameter “-a”) because of the known negative effect of Gli3. The area under the curve threshold is 0.05.

### Soft cluster of chromatin accessibility data

Chromatin accessibility data at the cellular level are extremely sparse, resulting in dramatic fluctuations even within the same cell type, while it is coarse at the cell type level. To mitigate this issue, we aggregate cells into pseudo-bulk samples by dividing cells into 10 groups along pseudotime before soft clustering [38]. The cell-type composition of each group is shown in Fig. 3I. Mean values are calculated and standardized for each pseudo-bulk sample. To estimate the optimized number of cluster centroids c, we perform soft clustering with a range of cluster numbers from 2 to 20, and 4 is determined as cluster number by the centroid distance plot. We extract alpha cores of each cluster using acore = 0.5, which preserves 18 peaks while discarding cluster 1 and cluster 4. To increase the concentration of members, we ultimately select 12 peaks from cluster 2 and cluster 3.

### Co-occurrence of genes and peaks

To identify the latent interacting genes of each cluster, we first calculate Spearman’s correlation between each peak in clusters and genes and then average the values of each gene for each cluster. With the purpose of eliminating contingency, 50 peaks for each peak are selected as the background based on GC content and coverage using the “getBackgroundPeaks” function of ChromVAR (RRID:SCR_026570) [80] package. Wilcoxon rank-sum test is performed to examine the difference.

### Running benchmarks

GLUE (v0.3.2) [10], uniPort (v1.1.2) [81], Cobolt [82] (v1.0.1), MinNet [83], scJoint [20], MultiVI (v0.19.0) [8], and sciCAN [84] were conducted using Python (v3.6). We followed the tutorials for each method: GLUE [85], uniPort [86], Cobolt [87], scJoint [88], MultiVI [89], and sciCAN [90]. We conducted Seurat V3 [91] using the R (v4.1.2) and the tutorial at [92].

All the methods were used the default settings and data preprocessing steps as recommended. Notably, scJoint, Seurat V3, sciCAN, and MinNet require converting peak counts into gene activity scores [93].

## Availability of Source Code and Requirements

Project name: Attune

Project homepage: https://github.com/melobio/Attune

Operating system: Platform independent

Programming language: Python

Other requirements: Python 3.6 or higher, Tensorflow 2.5.0

License: GPL-3.0 License

RRID:SCR_026494

bio.tools ID: attune

An archival copy of the code is available via Software Heritage [94].

## Supplementary Material

giaf053_Supplemental_Files

giaf053_Authors_Response_To_Reviewer_Comments_Original_Submission

giaf053_Authors_Response_To_Reviewer_Comments_Revision_1

giaf053_GIGA-D-24-00345_Original_Submission

giaf053_GIGA-D-24-00345_Revision_1

giaf053_GIGA-D-24-00345_Revision_2

giaf053_Reviewer_1_Report_Original_Submission Yang Xu -- 10/15/2024

giaf053_Reviewer_1_Report_Revision_1 Yang Xu -- 1/23/2025

giaf053_Reviewer_2_Report_Original_Submission Yingxin Lin -- 10/18/2024

giaf053_Reviewer_2_Report_Revision_1 Yingxin Lin -- 2/6/2025

## Data Availability

All datasets utilized in this study are publicly accessible: The scRNA-seq and scATAC-seq data for PBMC are available from 10x Genomics [95]. The SHARE-seq dataset, which includes data from mouse skin in the late anagen stage, can be accessed via NCBI GEO (accession GSE140203). The NeurIPS dataset, comprising data from human bone marrow, is publicly available on GEO (accession GSE194122). The Greenleaf 2021 dataset, derived from human brain cortex, is provided through GEO (accession GSE162170). Data utilized in this study are also available via Figshare (Attune 10x multiome dataset [96]; SHARE-seq TAC dataset [97]). A complete list of all datasets and additional information can be found in Supplementary Table S1. Additional supporting data are available via the *GigaScience* database, GigaDB [98]. DOME-ML (Data, Optimization, Model and Evaluation in Machine Learning) annotations are available via the DOME registry (accession q1loj0zi07) [99].

## Additional Files

**Supplementary Table S1**. Summary of the dataset used.

**Supplementary Table S2**. Performance evaluation of multimodal integration on the 10x Multiome dataset.

**Supplementary Table S3**. Performance evaluation of multimodal integration on the SHARE-seq dataset.

**Supplementary Table S4**. Performance evaluation of the quality of learned embeddings on the 10x Multiome dataset.

**Supplementary Table S5**. Evaluation of the rationality of feature number selection.

**Supplementary Table S6**. Evaluation of the rationality of hyperparameter selection.

**Supplementary Table S7**. Ablation study on contrast between different modalities or networks in multimodal integration.

**Supplementary Table S8**. Performance evaluation of cross-modal prediction on the 10x Multiome dataset.

**Supplementary Table S9**. Performance evaluation of cross-modal prediction on competition of NeurIPS 2021.

**Supplementary Table S10**. Evaluating the impact of Attune pretraining on cross-modal prediction.

**Supplementary Table S11**. Ablation study on contrast between different modalities or networks in the cross-modal prediction.

**Supplementary Table S12**. All attention weight between gene and peak of 10x PBMC Multiome dataset.

**Supplementary Table S13**. Differential expression of 466 dense associated genes.

**Supplementary Table S14**. Network importance scores for target genes of refined Gli3 regulons.

**Supplementary Table S15**. Membership values of 4 clusters are calculated from Gli3 peaks.

**Supplementary Table S16**. Correlation between genes and cluster 2 or 3.

**Supplementary Fig. S1**. Metrics of evaluating integration performance.

**Supplementary Fig. S2**. UMAP visualization of the cell embeddings in the 10x Multiome dataset aligned with different integration methods.

**Supplementary Fig. S3**. UMAP visualization of the cell embeddings in the SHARE-seq dataset aligned with different integration methods.

**Supplementary Fig. S4**. (a) Benchmark of AUC score in different distance and algorithm settings. (b) Marker genes from Fig. 2D are visualized in RNA modality.

**Supplementary Fig. S5**. (a) Attention weight is in descending order and the elbow is tipped in the top 5% on the SHARE-seq dataset. (b) Expression difference of 246 DPAGs. Some top DEGs are highlighted. (c) Heatmap of gene expression or accessibility with cell types. Each row represents a gene–peak pair extracted by attention weight.

**Supplementary Fig. S6**. (a) UMAP of SHARE-seq dataset colored by cell type. (b) UMAP of SHARE-seq dataset colored by pseudotime. (c) Abundances of 12 peaks from clusters 2 and 3 are plotted in different cell types.

**Supplementary Fig. S7**. The pattern of Residual. (a) Top 100 and (b) Bottom 100 genes selected for visualization based on variance. (c) Residuals of some highly expressed and low expressed genes.

**Supplementary Fig. S8**. (a) UMAP of human fetal cortex dataset colored by cell type. (b) UMAP of human fetal cortex dataset colored by pseudotime. (c) Distribution of cosine distance among cell type. (d) Cells with cosine distance above 0.1 are highlighted in the embedding.

**Supplementary Fig. S9**. Illustration of Attune’s ability to project unseen cell types onto a reference on the 10x Multiomics dataset. (a) CD14 Mono, (b) CD4 naive, and (c) CD4 TCM.

## Abbreviations

ASW: average silhouette width; CDC: conventional dendritic cell; DEG: differentially expressed gene; DPAG: dense peak-associated gene; FOSCTTM: fraction of samples closer than the true match; GC: graph connectivity; Hh: Hedgehog pathway; HVG: highly variable gene; IQR: interquartile range; MAP: mean average precision; MLP: multilayer perceptron; NC: neighbor consistency; PBMC: peripheral blood mononuclear cell; PC: principal component; PCA: principal component analysis; PCHi-C: promoter capture Hi-C; RMSE: root mean square error; SAS: Seurat alignment score; scATAC-seq: single-cell ATAC sequencing; scRNA-seq: single-cell RNA sequencing; VL: Vision-Language.
