# Supplementary material for: Cross-modal contrastive learning decodes developmental regulatory features through chromatin potential analysis
Source: Gigascience. 2025 Oct 17;14:giaf053. doi: 10.1093/gigascience/giaf053 (PMC12532322; doi:10.1093/gigascience/giaf053)
Supplement: giaf053_Supplemental_Files [file giaf053_supplemental_files.zip › Supplementary_File.pdf]

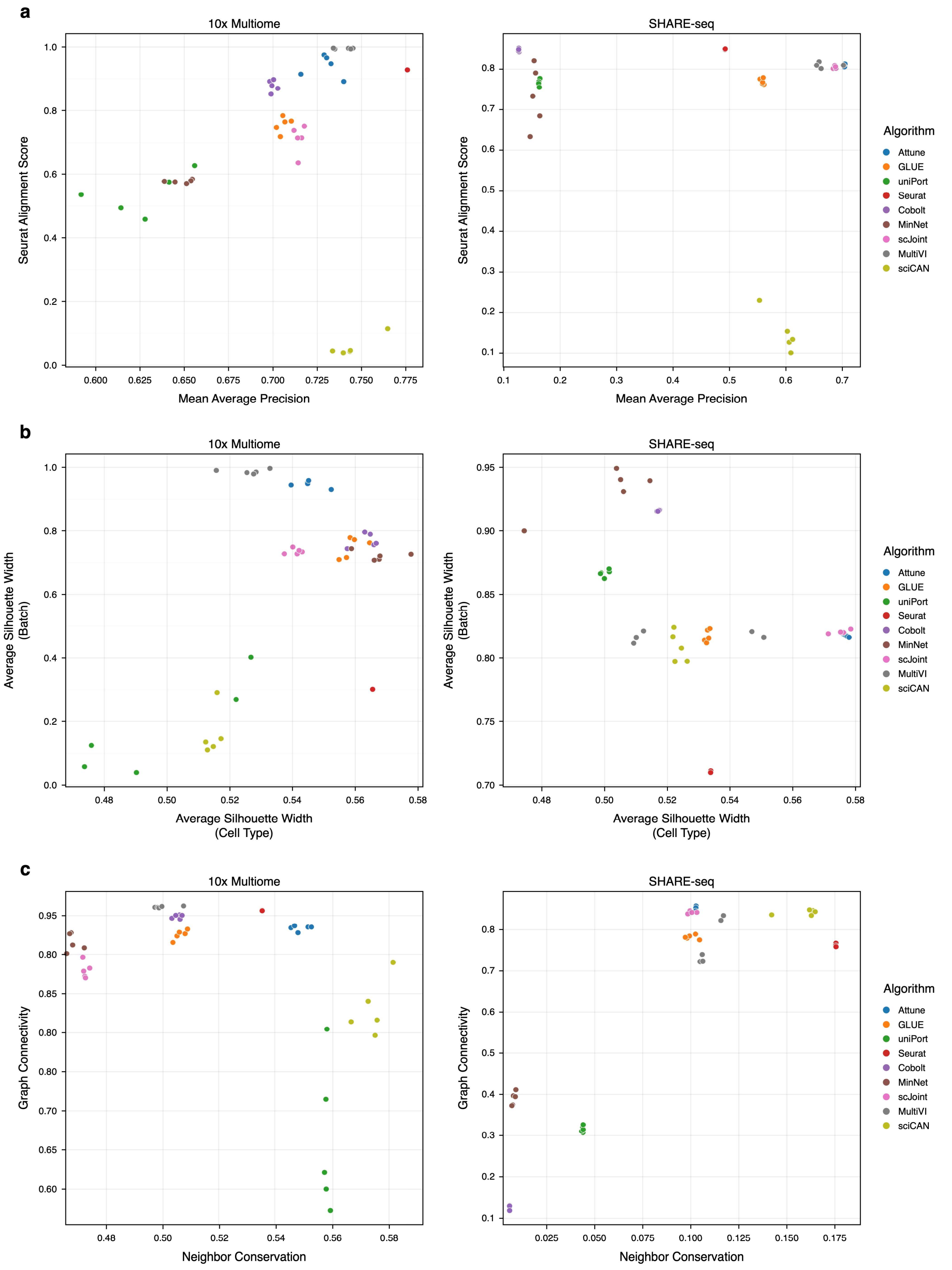

Supplementary Figure 1. Metrics for evaluating integration performance. (a) Mean average precision vs. Seurat alignment score. (b) Cell type vs. Batch average silhouette width. (c) Neighbor conservation vs. Graph connectivity.

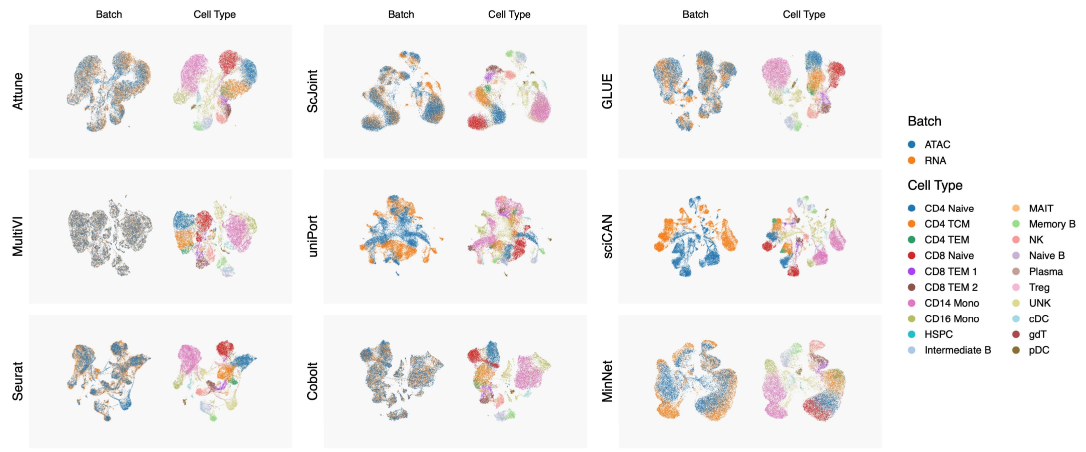

Supplementary Figure 2. UMAP visualization of the cell embeddings in the 10X Multiome dataset aligned with different integration methods.

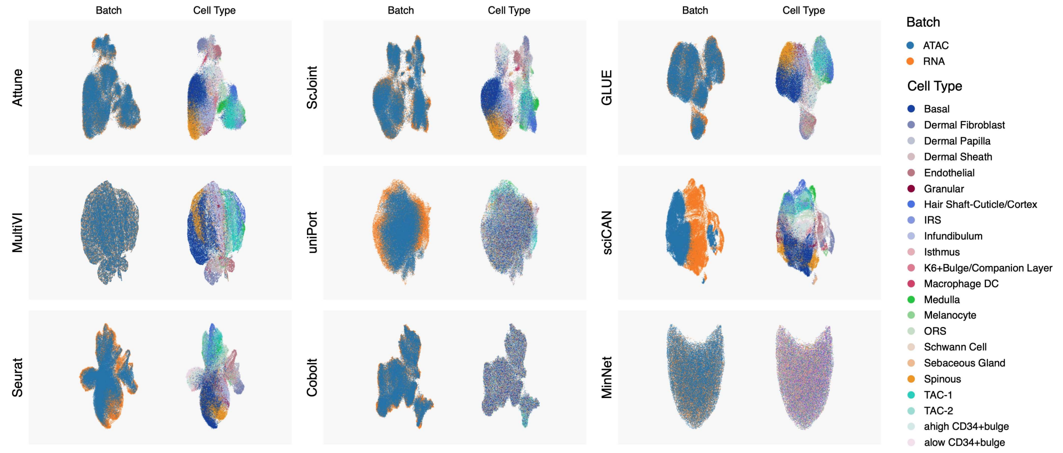

Supplementary Figure 3. UMAP visualization of the cell embeddings in the SHARE-seq dataset aligned with different integration methods.

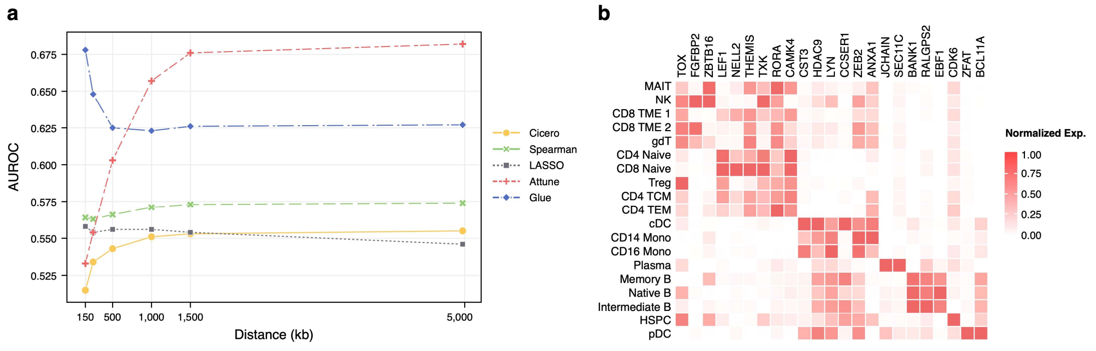

Supplementary Figure 4. (a) Benchmark of AUC score in different distance and algorithm settings. (b) Marker genes from Figure 2D are visualized in RNA modality.

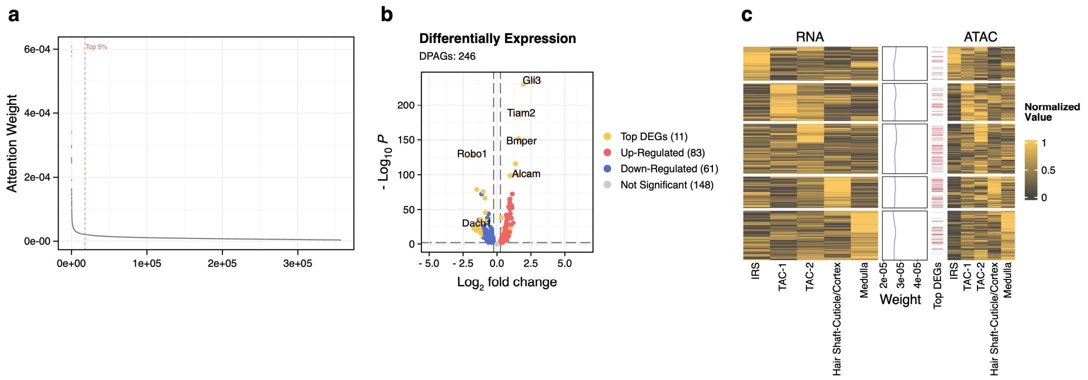

Supplementary Figure 5. (a) Attention weight is in descending order and the elbow is tipped in the Top 5% on the SHARE-seq dataset. (b) Expression difference of 246 DPAGs. Some Top DEGs are highlighted. (c) Heatmap of gene expression or accessibility with cell types. Each row represents a gene-peak pair extracted by attention weight.

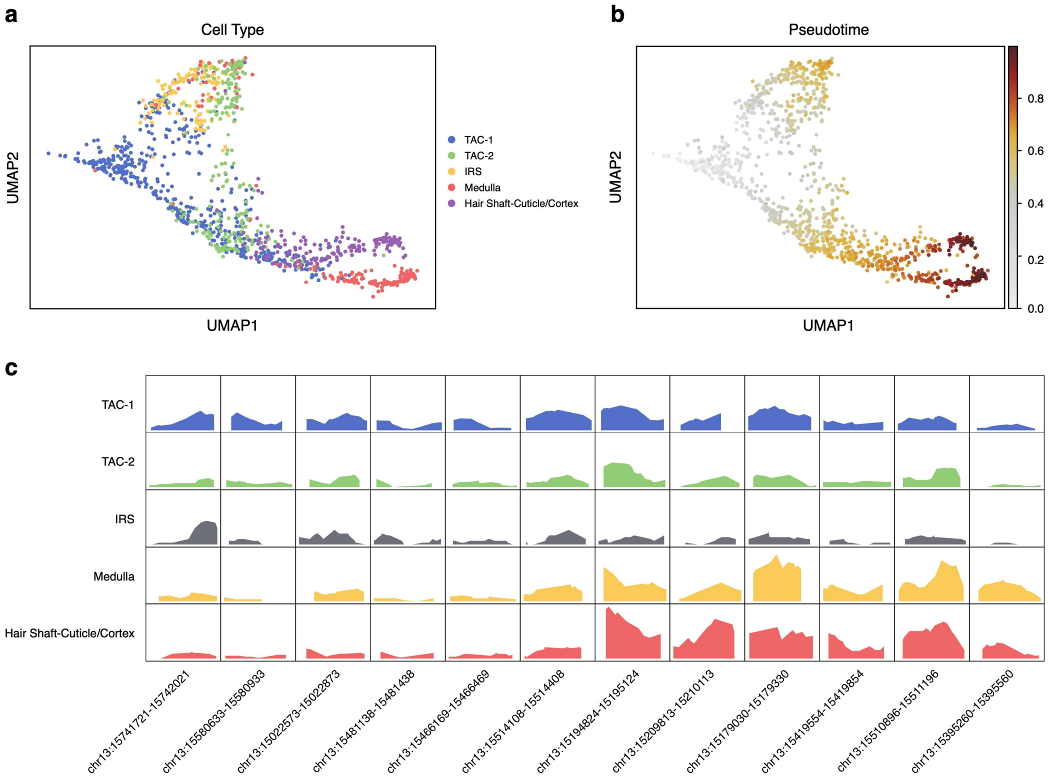

Supplementary Figure 6. (a) UMAP of SHARE-seq dataset colored by cell type. (b) UMAP of SHARE-seq dataset colored by pseudotime. (c) Abundances of 12 peaks from cluster2 and 3 are plotted in different cell types.

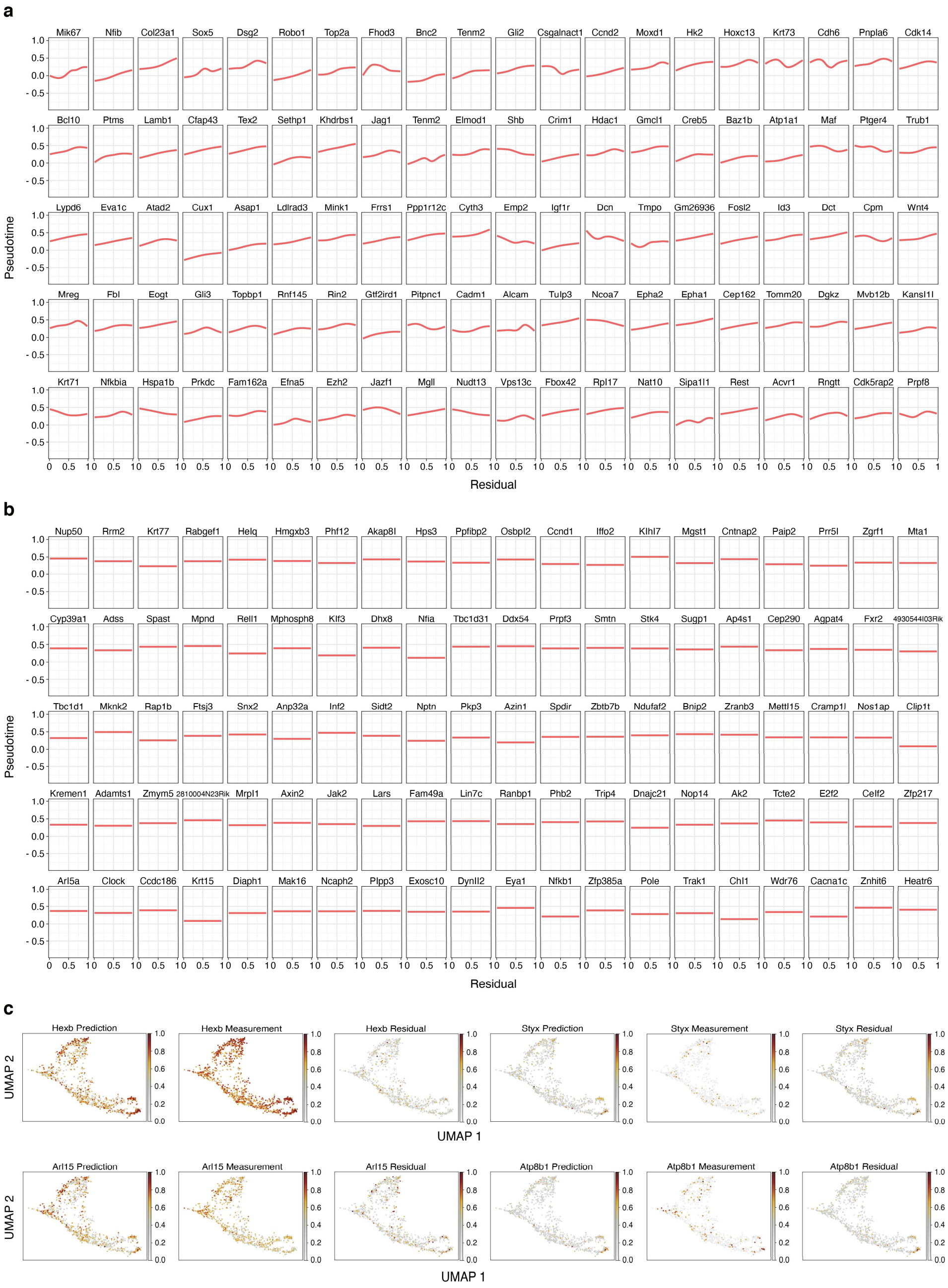

Supplementary Figure 7. The pattern of Residual. (a) Top 100 and (b) Bottom 100 genes selected for visualization based on variance. (c) Residuals of some highly expressed and low expressed genes.

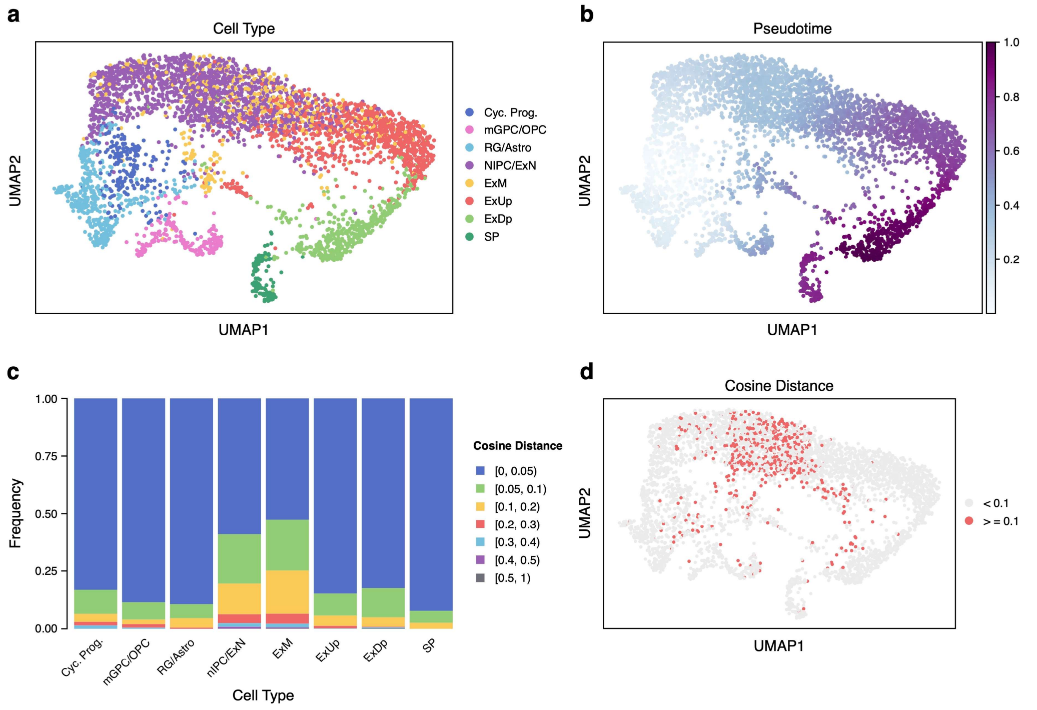

Supplementary Figure 8. (a) UMAP of human fetal cortex dataset colored by cell type. (b) UMAP of human fetal cortex dataset colored by pseudotime. (c) Distribution of cosine distance among cell type. (d) Cells with cosine distance above 0.1 are highlighted in the embedding.

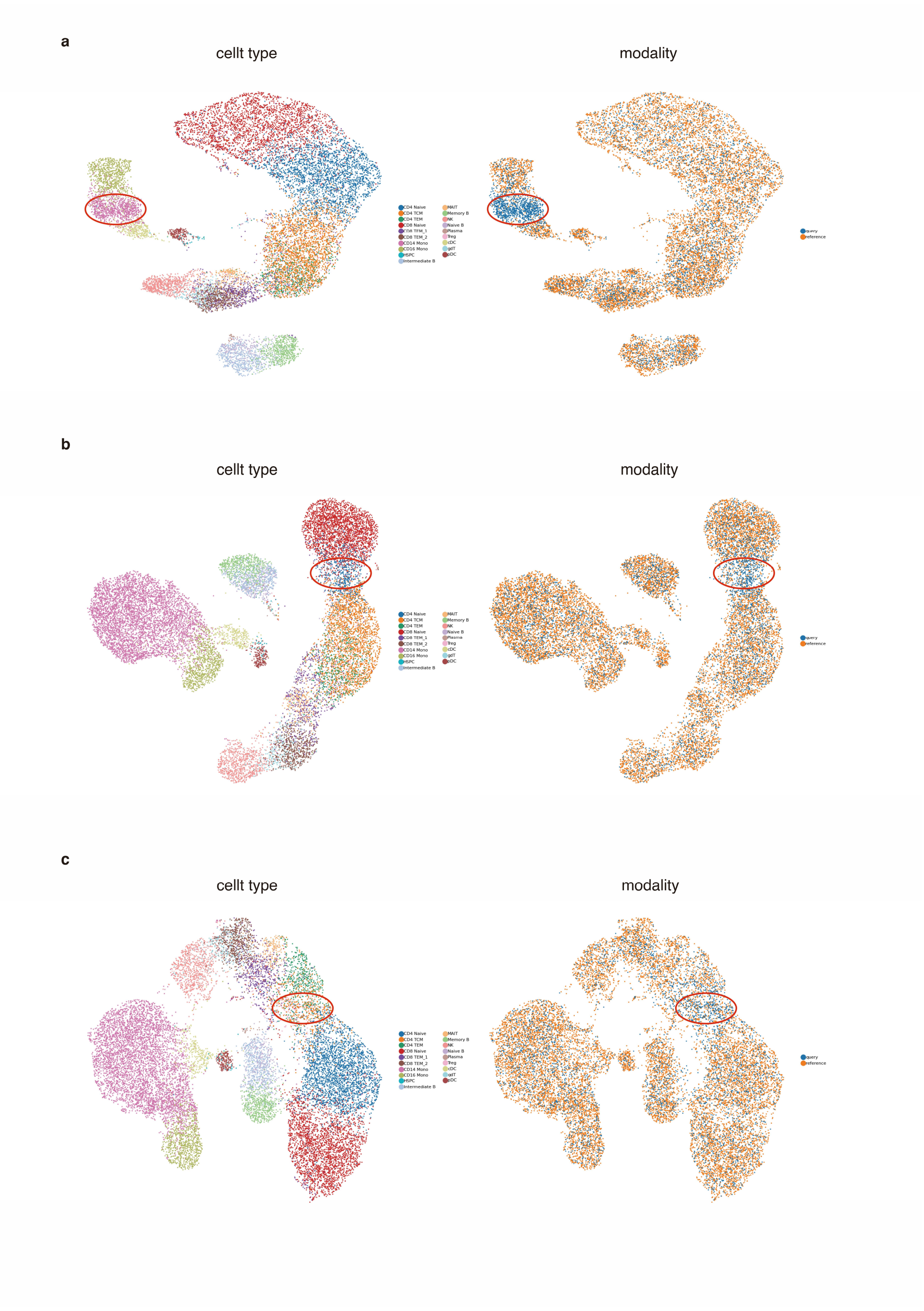

Supplementary Figure 9. Illustration of Attune's ability to project unseen cell types onto a reference on the 10x Multiomics dataset. (a) CD14 Mono (b) CD4 Naïve (c) CD4 TCM.
