# Supplementary material for: Cross-modal contrastive learning decodes developmental regulatory features through chromatin potential analysis
Source: Gigascience. 2025 Oct 17;14:giaf053. doi: 10.1093/gigascience/giaf053 (PMC12532322; doi:10.1093/gigascience/giaf053)
Supplement: giaf053_GIGA-D-24-00345_Original_Submission [file giaf053_giga-d-24-00345_original_submission.pdf]

GigaScience

Cross-modal contrastive learning discovers chromatin potential regulating gene expression of single cell atlas

--Manuscript Draft--

**Manuscript Number:**	GIGA-D-24-00345
**Full Title:**	Cross-modal contrastive learning discovers chromatin potential regulating gene expression of single cell atlas
**Article Type:**	Technical Note
**Funding Information:**	
**Abstract:**	Emerging large-scale multimodal single-cell data jointly measures chromatin accessibility and transcription in the same cell, thus reconciling matched data paves integrated route for comprehensive regulatory analysis. Here, we introduce Attune, a cross-modal contrastive learning framework to align paired gene expression and accessibility information. Systematic benchmarking shows Attune's superior performance for omics integration and gene expression prediction. We further introduce Transformer-based cross-modal attention over fine-tuned gene and peak embeddings to infer regulatory interaction and discover significant differential signals of cell subtypes. Applied to hair follicle maturation dataset, Attune reveals chromatin potential for bifunctional transcription factor Gli3 at the gene level. In addition, the paired representations determine transmitted states across neonatal and mature cell types of cortical neuron differentiation at the cell level. Taken together, Attune features a promising paradigm for regulatory inference across omics layers and allows for extending more complex omics analysis.
**Corresponding Author:**	MENG YANG<br>BGI-Shenzhen: BGI Group<br>Shenzhen, CHINA
**Corresponding Author Secondary Information:**	
**Corresponding Author's Institution:**	BGI-Shenzhen: BGI Group
**Corresponding Author's Secondary Institution:**	
**First Author:**	Yueyuxiao Yang
**First Author Secondary Information:**	
**Order of Authors:**	Yueyuxiao Yang
	Chenxi Xie
	Qiushun He
	MENG YANG
**Order of Authors Secondary Information:**	
**Additional Information:**	
**Question**	**Response**
Are you submitting this manuscript to a special series or article collection?	No
**Experimental design and statistics**<br><br>Full details of the experimental design and statistical methods used should be given in the Methods section, as detailed in our	Yes

Minimum Standards Reporting Checklist. Information essential to interpreting the data presented should be made available in the figure legends.<br><br>Have you included all the information requested in your manuscript?	
**Resources**<br><br>A description of all resources used, including antibodies, cell lines, animals and software tools, with enough information to allow them to be uniquely identified, should be included in the Methods section. Authors are strongly encouraged to cite Research Resource Identifiers (RRIDs) for antibodies, model organisms and tools, where possible.<br><br>Have you included the information requested as detailed in our Minimum Standards Reporting Checklist?	Yes
**Availability of data and materials**<br><br>All datasets and code on which the conclusions of the paper rely must be either included in your submission or deposited in publicly available repositories (where available and ethically appropriate), referencing such data using a unique identifier in the references and in the "Availability of Data and Materials" section of your manuscript.<br><br>Have you have met the above requirement as detailed in our Minimum Standards Reporting Checklist?	Yes

Click here to access/download;Manuscript;Manuscript.docx

Cross-modal contrastive learning discovers chromatin potential regulating gene expression of single cell atlas

Yueyuxiao Yang[1], Chenxi Xie[1], Qiushun He[1], Meng Yang[1]*

[1]MGI, BGI-Shenzhen, Shenzhen 518083, China.
*Correspondence to: yangmeng1@mgi-tech.com

Abstract

Emerging large-scale multimodal single-cell data jointly measures chromatin accessibility and transcription in the same cell, thus reconciling matched data paves integrated route for comprehensive regulatory analysis. Here, we introduce Attune, a cross-modal contrastive learning framework to align paired gene expression and accessibility information. Systematic benchmarking shows Attune's superior performance for omics integration and gene expression prediction. We further introduce Transformer-based cross-modal attention over fine-tuned gene and peak embeddings to infer regulatory interaction and discover significant differential signals of cell subtypes. Applied to hair follicle maturation dataset, Attune reveals chromatin potential for bifunctional transcription factor Gli3 at the gene level. In addition, the paired representations determine transmitted states across neonatal and mature cell types of cortical neuron differentiation at the cell level. Taken together, Attune features a promising paradigm for regulatory inference across omics layers and allows for extending more complex omics analysis.

Keywords

Single-cell multi-omics, Contrastive learning, Transformer, Chromatin potential, Regulatory analysis

Introduction

Gene transcription is a dynamic process that drives the precise differentiation of cell lineages[1]. This complex process is orchestrated by coordinated regulation of chromatin accessibility around key regulatory elements, such as promoters and enhancers, which creates a permissive landscape for the binding of transcription factors and co-factors, thus initiating transcription[2].

Recent advancements in multimodal sequencing technologies, such as 10x Multiome, SHARE-seq[3], SNARE-seq[4] and scCAT-seq[5], have enabled the simultaneous measurement of multiple layers of a single cell, including chromatin and transcriptional status. By capitalizing on these data, it becomes conceivable to refine cell identity and reconstruct the causal sequence of the regulatory network[6] that underlies cellular differentiation. Moreover, by integrating information from multiple modalities within a joint embedded space and accounting for temporal dynamics, we can unearth the intricate relationships and dependencies that permeate across these modalities. Despite the existence of various techniques for integrating multimodal single-cell data, such as Seurat

V4[7], MultiVI[8], BABEL[9], they are predominantly focused on integration or prediction tasks, resulting in a pressing need for approaches to elucidate the regulation of cell differentiation. GLUE[10] employs a guidance graph to explicitly model cis-regulatory interactions between feature spaces, while MIRA utilizes a combination of topic modeling and regulatory potential modeling to capture key regulators at lineage branch points[11]. Nevertheless, the pool of available methods specifically designed for this purpose remains limited.

Multimodal deep learning, a formidable technique within the realm of computer science, offers a panoramic understanding of data, wielding substantial power. For example, Vision-Language (VL) pre-training has demonstrated remarkable efficacy in various VL downstream tasks[12], as evidenced by the success of CLIP[13] and ALBEF[14]. These models utilize a contrastive learning module to pretrain the encoder and subsequently finetune it with a transformer-based decoder. In the context of single-cell multi-modal data, the deployment of multimodal deep learning algorithms becomes profoundly advantageous. Drawing inspiration from the exceptional representation capabilities of contrastive learning for multimodal data[13,15], as well as the impressive performance of transformer models in uncovering latent interactions[14,16], we introduce Attune: a cross-modal contrastive learning pre-training model designed to capture the interactions between different modalities. By maximizing the agreement between modalities within a cell on the hypersphere[17], Attune places representations of distinct modalities into a shared feature space, enabling the modeling of interactions between peaks and genes.

Using Attune, we apply learned cell embeddings to a range of downstream tasks and achieve superior performance in cross-modal prediction tasks. By employing a transformer-based decoder, we can construct gene-peak interaction networks and interrogate the regulations underlying key developmental processes, such as the maturation of transit-amplifying cells in the hair follicle dataset and the active transition region during neuron differentiation in the cortex dataset. Comprehensive benchmarking and regulation analysis demonstrate the power of Attune in learning comprehensive and informative representations of omics-specific features and reconstructing regulatory interactions.

Results

Attune achieves exceptional overall performance of integration

Attune employs cross-modal contrastive learning approach to integrate scRNA-seq and scATAC-seq data effectively, while maintaining a high degree of biological conservation.

The learned cell embeddings from Attune can be fine-tuned for downstream tasks such as cross-modal prediction, recovery of peak-gene interaction, and differentiation analysis, as illustrated in Figure 1a. To demonstrate the performance of Attune, we benchmark it against other multimodal integration methods on matched scRNA-seq and scATAC-seq datasets, such as 10x Multiome and SHARE-seq, with several established metrics[10,18], most of which have been widely embraced and validated in previous single-cell integration tasks, such as graph connectivity (GC) in the NeurIPS 2021 competition[19] and average silhouette width (ASW) in scJoint[20] (see Methods in detail).

As shown in Figure 2a-left (refer to Table S2 for details), Attune emerges as the clear winner, situated in the top-right corner of the 10x Multiome dataset, signifying that it strikes a balance between omics mixing and biological fidelity. MultiVI, on the other hand, demonstrates higher omics mixing but sacrifices biological meaning. Moreover, Attune outperforms other methods on the SHARE-seq dataset (Figure 2a-right, refer to Table S3 for details), as evaluated by three separate metrics (see Supplementary Figure 1). Upon probing Attune's integration scores on distinct datasets, we observe that it obtains the highest overall integration scores (mean 0.806 and 0.829 for 10x Multiome and SHARE-seq data, respectively) in Figure 2b-left. The UMAP visualization of the cell embeddings for the 10x Multiome and SHARE-seq datasets is presented in Supplementary Figure 2 and Supplementary Figure 3, respectively. We also quantify the alignment performance between modalities using the Fraction Of Samples Closer Than the True Match (FOSCTTM) on both datasets, as depicted in Figure 2b-right. The lowest FOSCTTM scores suggest that Attune effectively matches different modalities from a cell.

To further evaluate the performance of Attune's feature distributions on the output unit hypersphere, we measure the alignment between cross-modal positive pairs and the uniformity of the entire representation space. This assessment allows us to determine the quality of learned embeddings. Compared to other integration techniques' cell embeddings (Figure 2c and Table S4), Attune achieves the best alignment (the lower the better), indicating that the feature distribution between RNA-ATAC pairs is more consistent in the high dimensional space. Other components regarding feature selection settings and hyperparameter choice are presented in Figure 2d and Figure 2e respectively (refer to Table S5 and Table S6 for more information) to substantiate the rationality under the current settings, thus enhancing the validity of the study.

The cells' embedding after integration establishes the cell labels and ontologies. Through multimodal reference building and mapping of query cells, it demonstrates that the embedding of cells retains their original biological characteristics[21,22]. We assess Attune's

ability to map query cells, including previously unseen cell types, onto reference embeddings. We utilize 80% of the cells from the 10x Multiome dataset to construct the reference embeddings, while the remaining 20% serves as the query cells with unseen cell types. Specifically, we first train the reference cells to obtain the reference embeddings. Next, we directly infer the query cells using pre-trained model weights to generate the query embeddings. Finally, we concatenate the reference embeddings and query embeddings along the sample dimension and visualize them using UMAP (refer to Supplementary Figure 9). In Supplementary Figure 9a, we deliberately exclude all CD14 monocyte cells (CD14 Mono) from the reference. Despite never encountering CD14 monocyte cells during training, Attune accurately localizes them between CD16 monocyte cells (CD16 Mono) and conventional dendritic cells (CDC), with query cells positioned in proximity to their most similar reference cells. Similarly, Supplementary Figures 9b and 9c demonstrate comparable outcomes. These findings emphatically highlight Attune's prowess in integrating multimodal data and acquiring biologically meaningful embeddings.

The essence of Attune lies in its cross-modal contrastive learning module. Taking inspiration from the pioneering work of Concerto[15], we regard Attune's multimodal contrastive learning framework as an indivisible entity. However, the efficacy of integration can be influenced by different internal comparison objects. To establish the intrinsic soundness of our module design, we conduct an ablation experiment on the cross-modal contrastive learning module. Since the module employs two asymmetric teacher-student networks, we compare three different designs. The first design involves comparing the RNA student network with the ATAC student network, and correspondingly, contrasting the RNA teacher network with the ATAC teacher network, as proposed in this study. The second one compares the RNA student network with the RNA teacher network, while simultaneously compares the ATAC student network with the ATAC teacher network. Finally, the third design involves comparing the RNA student network with the ATAC teacher network, and conversely, contrasting the RNA teacher network with the ATAC student network. We assess the performance disparities among these three comparison methods in the context of multimodal integration (Table S7) and discover that the first comparison method yields the most exceptional outcomes. This observation suggests that the improved performance of Attune stems not from a mere amalgamation of network components, but rather from the ingenious architectural design.

Internal relation is well captured by Attune resulting in outstanding

performance in cross-modal prediction

In cross-modal prediction, our objective is to predict all feature values for each cell in scRNA-seq using scATAC-seq, and this requires algorithms to learn the complex regulatory interactions between layers of genetic information. To assess the performance of Attune, we compare it against state-of-the-art cross-modal prediction methods such as BABEL[9] and Polarbear[23], using the 10x Multiome dataset (PBMC10k, n=11,909). We randomly divide cells into training (n=9527) and testing (n=2382) sets (bootstrapping five times), and evaluate the method using gene-wise Pearson correlation and gene-wise Spearman's correlation. Our findings show that Attune outperforms other methods with the highest Spearman's correlation coefficient (0.243), the highest Pearson correlation coefficient (0.271), and the lowest Root Mean Square Error (RMSE) of 0.528. Figure 2f provides a visual representation of our results, while Table S8 contains detailed information.

To further verify our findings, we compare the performance of Attune with the top five winners from the modality prediction task (ATAC-GEX subtask) using the official settings, datasets, and guidelines from the multimodal single-cell data integration competition of NeurIPS 2021[19]. As illustrated in Figure 2g and Table S9, Attune outperforms all other methods with the lowest RMSE.

We posit that the efficacy of cross-modal prediction may be significantly influenced by the integration of pretraining. To investigate this hypothesis, we design a comparative experiment to demonstrate the benefits derived from fine-tuning Attune's pretrained model through the utilization of a multilayer perceptron (MLP). Our investigation entails an examination of three distinct settings: an MLP network with pretraining, an MLP network without pretraining (de novo training), and classical regression methods such as LASSO (Table S10). Additionally, we conduct a comprehensive ablation study to elucidate the impact of Attune's structure on the performance of cross-modal prediction, as presented in Figure 2h and Table S11. These results prominently underscore the significant impact of Attune's pretrained model on the robustness of fine-tuning and its potential for downstream tasks.

Transformer's cross-attention mechanism enables revealing regulatory interaction of genes via fine-tuning Attune

Various metrics are utilized to infer regulatory interactions by quantifying the relationship between modalities. For instance, Cao et al.[10] utilized cosine similarity of different feature

embeddings, while Trevino et al.[24] and Sai Ma et al.[3] used correlation metrics to evaluate the relationship between genes and peaks. In this work, we leverage the intrinsic property of transformer, i.e., the ability of cross attention to discover inner connections, to quantify associations between genes and peaks by utilizing attention weight.

To demonstrate the efficacy of Transformer in discovering regulatory interactions, we employ a matched scRNA-seq and scATAC-seq Multiome dataset from 10x Genomics, consisting of approximately 11,000 human peripheral blood mononuclear cells (PBMC). Previous studies have suggested that regulatory elements, such as enhancers and silencers, may be distributed away from promoters up to several Mbps[25,26]. Based on these observations and the statistical analysis of the dataset from Javierre, B. M.[27] (see Figure 3a), we mask genes and peaks whose distances are greater than 1.2 Mbps (see Supplementary Figure 4a and Methods) for comprehensive prediction and evaluation[28,29]. We utilize a Promoter capture Hi-C (PCHi-C) dataset of human PBMC that profiles distal promoter-interacting regions as a validated resource[27]. As illustrated in Figure 3b, Attune+Transformer, i.e. training the Transformer model by fine-tuning Attune's pre-trained model, outperforms other methods, including Cicero[30], LASSO, and GLUE[10], in regulatory prediction, indicating that cross attention learned by Transformer (with Attune pretraining) captures promoter-interacting regions effectively.

To further substantiate the benefits of Attune's pre-training, we introduce an ablation experiment. This experiment incorporates the PCA+Transformer configuration, where the Transformer is trained solely on the gene embeddings and peak embeddings derived from the first 10 Principal Components (PCs) extracted via Principal Component Analysis (PCA), without the utilization of Attune pre-training. These comparisons in Figure 3b highlight the role of Attune pre-training in facilitating the Transformer's effective capture of promoter-interacting regions.

To elucidate the biological signals captured by the attention mechanism, we select gene-peak pairs with the top 10% of attention weight using an inflection point ("elbow") when ranking gene-peak pairs by attention weight (Figure 3c). A total of 8,744 gene-peak pairs remains under this cutoff, including 5,447 peaks and 646 genes (full list in Table S12). We define 466 genes linking at least 10 peaks among them as DPAGs (dense peak-associated genes). Key regulatory events may occur within DPAGs and their associated peaks[3]. Most of the DPAGs express differentially (354 versus 466, see Table S13 for details) and are enriched in immune response regulating signaling (p.adjust=0.001), mononuclear cell differentiation (p.adjust=0.001), and positive regulation of cell adhesion (p.adjust=0.005), as shown in Figure 3d and 3e. DPAGs include cell markers of plasma (JCHAIN, SEC11C),

HSPC (CDK6), pDC (BCL11A, ZFAT), B naive or B memory cells (BANK1, EBF1), etc., and clear separation of cell types is observed in Figure 3f and Supplementary Figure 4b from both modalities. Similar results of the SHARE-seq dataset are displayed in Supplementary Figure 5.

Attune enables chromatin potential discovery and illuminates the priming of lineage

Attune outperforms other methods in predicting cell modalities, but some genes exhibit low Pearson correlation coefficients. Based on the delay between chromatin accessibility and transcription[2,3,31], we propose that chromatin potential, which refers to the latent information underlying chromatin accessibility or transcriptional delay, may account for the inaccurate prediction of certain genes, particularly during lineage development. To investigate this hypothesis, we calculate the residuals between predicted and measured gene expressions on the mouse skin SHARE-seq dataset, which represents chromatin potential, as hair follicles remain cell cycle even in adulthood. Supplementary Figure 7a and 7b illustrate that residuals display diverse patterns of gene expression during cell differentiation, evident by their trends along pseudotime (see Methods). Some genes, including Hexb, Arl15, Styx, and Atp8b1, exhibit consistency between predicted and measured expressions, whereas Gli3 displays conspicuous residuals (Figure 3g, Supplementary Figure 7c). Notably, the high residuals of Gli3 emerge before cell type transition, known as lineage commitment[3], as indicated by the low-dimensional projection of cell type and pseudotime in Supplementary Figure 6a-6b and the change in residual in Figure 3h, pointing to a delay between modalities.

As an example, Gli3 serves a critical function in the Hedgehog pathway (Hh) and regulates hair follicle cycles in embryonic and adult skin[32,33]. In the canonical Hh pathway, Gli3 primarily acts as a repressor to maintain pathway activity balance[34,35]. Regulon analysis by SCENIC[36] confirms its role in transcriptional inhibition, with network importance scores for refined Gli3 regulon target genes listed in Table S14, such as Basp1 (14.03), Sema4a (5.21), and Myh14 (3.36). Given its diverse functions and multiple targets during development, we speculate that the lag of Gli3 contributes to lineage differentiation.

Regulation of Gli3 orderly shift with the maturity of cells

In a previous study, Gli3 was identified as a highly connected transcriptional repressor with limited description[3]. In this study, we aim to elucidate the lagging mechanism of Gli3 and

provide a comprehensive understanding of its role in hair follicle maturity. We utilize a prediction subtask to generate expression from chromatin state signal and find that the transition of chromatin accessibility may account for the delay of Gli3. After filtered by attention weight, 49 peaks of Gli3 are soft clustered (see Figure 3i and Method)[37,38]. As depicted in Figure 4a and Table S15, we determine four clusters, with clusters 2 and 3 having a higher number of peaks with high membership value (>0.5). Cluster 2 shows a downward trend in peak accessibility, while cluster 3 demonstrates an opposite fluctuation, suggesting that the peaks around Gli3 change in accessibility in a coordinated manner instead of independently opening or closing. To reduce noise, we further analyze 12 peaks from clusters 2 and 3 for their relationship with Gli3.

The majority of the 12 peaks are situated within 500 kb of Gli3's transcriptional start site (as depicted in Figure 4b) and are found to be accessible during the early or late differentiation stage (as shown in Figure 4c, either at the top or bottom, respectively, and Supplementary Figure 6c). To investigate the genes that impact accessibility of these 12 peaks, we perform Spearman correlation analysis between the peaks and genes to discover cluster-associated genes (see Methods). Our analysis reveals that Eda ($p = 6.01 \times 10^{-4}$), Nfib ($p = 2.20 \times 10^{-4}$), Sox5 ($p = 6.67 \times 10^{-4}$), Ntn1 ($p = 4.38 \times 10^{-4}$), and Tspan18 ($p = 4.79 \times 10^{-4}$) are the top five correlated genes for cluster 2, while Lef1 ($p = 1.52 \times 10^{-4}$), Prr5l ($p = 1.43 \times 10^{-4}$), Foxp1 ($p = 4.72 \times 10^{-5}$), Bmper ($p = 1.64 \times 10^{-4}$), and Dach1 ($p = 2.85 \times 10^{-4}$) are the top five correlated genes for cluster 3 (see Figure 4d and Table S16 for details). Notably, Nfib, Sox5, Lef1, Foxp1, and Dach1 are transcription factors. Our findings suggest that Gli3 may undergo a regulatory shift, and the peaks may either activate or deactivate in clusters 2 and 3, respectively, as shown in Figure 4e, which corresponds to the observed time lag in Gli3 expression.

Cross-talk of multiple pathways commits cell differentiation

Similar to other developmental processes[39-41], the differentiation of hair follicle stem cells is a highly regulated process that involves the interaction of multiple pathways, including Wnt, Bmp, Notch, and Hh, among others. These pathways act as positive or negative feedback loops, as described in previous studies[42,43]. Using matched multimodal data, we employ Attune to recover a complex regulatory network by a chain rule from residual to peak and to other genes. Based on the discovery of cluster-associated genes, targets, and literature review, we propose a model for hair follicle maturity (see Figure 5a).

During the early stages, when transient-amplifying cells predominate (peaks in cluster2 and their associated genes), cell proliferation continues while the function of Gli3 is

hindered directly or indirectly. Eda promotes the Hh pathway via the Wnt-Eda-Shh cascade [44]. Sox9, which regulates GLI expression, can be enhanced by Sox5/6[45-47], while Nfib and Nfia enable DNA-binding transcription activator activity and share many targets, including Gli3, Sox3, and Cdh2[48-50]. These signals decrease as cell specification becomes more pronounced.

At t3-t4 time points, the expression of Lef1 and Dach1 increases (peaks in cluster3 and their associated genes). TCF/LEF restricts Shh activity by binding to the enhancer of Gli3. Motif analysis also identifies peaks (chr13:15510896-15511196 and chr13:15395260-15395560) containing the LEF binding motif, which is consistent with previous research. Along with the Wnt pathway, Bmp[51-53], Foxp1-Runx2[54], and Prr5l-mTORC2[55-57] are implicated in the maintenance of the Hh pathway and Gli3. The down-regulation of target genes, such as Basp1[58,59] and Chd3[60], occurs upon inactivation of Hh, forming a feedback loop. In conclusion, the pattern shift of the chromatin state, initiated from chromatin potential, sheds new light on lineage priming and provides a basis for further developmental studies.

Embeddings facilitate discovery of key factors among active differentiating cells

The Attune algorithm not only ensures reliability for downstream tasks but also preserves biological signals in the high-dimensional space of cell embeddings. We delve into the embeddings between modalities in the fetal human cortex dataset (Supplementary Figure 8a-8b) and calculate cosine similarity for each pair of cells from RNA and ATAC embeddings, as depicted in Figure 5b. Higher cosine similarity values are enriched in the diagonal, with the exception of the boundary between newborn neurons (nIPC/ExN), maturing neurons (ExM), and excitatory neurons in the upper layer (ExUp). The overall upward shift of blocks indicates the lag of RNA modality. These findings are supported by the observation of more cells with high cosine distance (1 - cosine similarity) at the junction of ExN, ExM, and ExUp regions in Figure 5c and Supplementary Figure 8c.

To investigate accessibility or expression events, we group cells with a cosine distance value above 0.1 (Supplementary Figure 8d) and conduct differential expression analysis. Our analysis reveals overexpression of genes such as CNTNAP2, DCC, SLIT2, and KCND2 in this group (p.adjust<0.01 and log fold change>0.25). Notably, CNTNAP2 or DCC knock-out models have been associated with abnormalities in neuronal migration[61,62], while products of KCNH8, SLC24A2, and KCND2 involved in ion transportation have been shown to impact neuronal excitability and maturing[63].

Discussion

Attune builds upon the principles of cross-modal contrastive learning and proposes a novel approach to tackle the problem of learning robust cell representations from multimodal data on a unit hypersphere. To this end, Attune leverages two teacher-student frameworks, achieving impressive performance in integration benchmarks without compromising biological signals. Drawing inspiration from the pretraining-finetuning paradigm, Attune's embeddings can be effortlessly adapted to diverse downstream tasks via fine-tuning, as validated through cross-modal prediction and regulatory interaction inference tasks.

By juxtaposing cells from disparate modalities in a common space, Attune enables the detection of regulatory events. Among the three datasets of peripheral blood, skin, and cortex examined, Attune uncovers the relationships between chromatin accessibility and transcription features, disentangles the intricate network of lineage priming regulations, and identifies transcriptionally active cells. By expanding regulatory sequence along both feature spaces using chromatin potential and cross-modal attention, Attune provides fresh insights into the maturation of hair follicles, which entail multiple pathways. Furthermore, Attune pinpoints the local inconsistencies in embeddings in the human cortex dataset and posits the occurrence of swift transcriptional activities in nascent neurons.

An upsurge in experimental protocols combining dual-modalities, trio-modalities, and other modalities highlights the inevitability and indispensability of matched sequencing[6,64]. Such a surge poses a challenge in scaling methods to suit increased modalities and cells. Attune rises to this challenge by employing a lightweight model framework and an optimized input structure, which facilitates the easy scaling of Attune to support millions of cell atlases. By combining contrastive loss between specified modalities, Attune can handle additional modalities of data and explore intermodal connections with greater flexibility. Additionally, Attune's embeddings present a unique opportunity for portraying differentiation trajectories, which complements existing methods for trajectory analysis[65].

While Attune has demonstrated its potential across multiple scenarios, its performance verification with limited data remains insufficient. Acknowledging this limitation, we aim to augment the validated dataset in subsequent stages to enhance model robustness and validate its applicability across diverse multiple modalities.

In conclusion, Attune constitutes a potent paradigm for analyzing matched multimodal single-cell data, enabling exploration of inter-modal relations and unearthing the mechanisms driving complex biological phenomena at a single-cell resolution.

Methods

Input data & preprocessing

The Attune model takes expression (gene count) and accessibility (peak count) matrices from matched multimodal scRNA-seq and scATAC-seq as input data. For scRNA-seq data, genes expressed in fewer than 5% of cells were filtered out. We used SCANPY[66] to normalize each cell count to 10,000 read counts before the logarithm. Additionally, sex chromosome genes were removed, and 2000 highly variable genes (HVGs) were selected

based on the experiments depicted in Figure 2d providing a reasonable compromise. This choice allows us to achieve satisfactory performance while managing the computational cost and ensuring the overall stability and efficiency of the model. For scATAC-seq data, peaks accessed in fewer than 5% of cells were filtered out. And peaks from sex chromosomes were also filtered out. A complete list of all data used in the study is provided in Table S1.

Input encoding scheme

The normalized expression and accessibility matrices were encoded in the TensorFlow Record (TF-record) format. The scRNA-seq data was encapsulated in one TF-record file, with 'gene index' and 'gene count' fields, while the scATAC-seq data was encapsulated in another file, with 'peak index' and 'peak count' fields.

Overview of model architecture

As illustrated in Figure 1b, the overarching model architecture encompasses the Attune pre-training model, which comprises two asymmetric teacher-student networks, along with two modules dedicated to downstream tasks: the cross-modal prediction module and the transformer-based peak-gene interaction module. Attune leverages separate teacher-student networks to learn cell representations from scRNA-seq and scATAC-seq respectively, through cross-modal contrastive learning. The teacher network, designed to be more complex, employs a hierarchical attention mechanism [67], while the student network uses a simpler dense operation. Positive pairs of embeddings from the two modalities of a cell are considered as positive pairs, and RNA and ATAC representations are learned by maximizing their agreement. The representations from both modalities are then projected into a common space.

We adapted the pre-trained Attune model to three downstream tasks: inference of gene-peak interaction and cross-modal prediction and differentiation analysis. Reconstructing of regulatory events requires the model to reconstruct the correspondence between accessible chromatin and gene expression and ascertain which chromatin regions are responsible for the change in gene expression across cells. To accomplish this, we utilized a transformer-based decoder that captures multimodal cross attention, thereby establishing the link between peaks and genes. For cross-modal prediction, Attune was fine-tuned via a multi-layer perceptron (MLP) to predict gene expression and further identify temporal differences in expression accessibility and transcription for differentiation analysis.

Teacher network

For two kinds of single-cell data (scRNA-seq and scATAC-seq), we designed two teacher networks (RNA teacher network and ATAC teacher network) to learn fine-grained representations respectively. The RNA teacher network accepts $X_{indices} \epsilon \mathbb{R}^G$ and $X_{counts} \epsilon \mathbb{R}^G$ as input, where G denotes the number of genes. $X_{indices}$ represents gene indices and X_{counts} represents the value of gene counts. For ATAC teacher network accepts input of $Y_{indices} \epsilon \mathbb{R}^P$ and $Y_{counts} \epsilon \mathbb{R}^P$, where P denotes the number of peaks. $Y_{indices}$ represents peak indices and Y_{counts} represents the value of peak counts. Each gene within a cell is represented by $i \epsilon \mathbb{R}^G$ and each peak is represented by $j \epsilon \mathbb{R}^P$. Both teacher networks use an Embedding layer to convert the input sequences into gene and peak embeddings.

First, $x_{indices}$ is embedded into a d-dimensional vector space $gene\ \ emb$, $gene\ \ emb_i \in \mathbb{R}^d$ (equation 1), where d is set to 128 as the default. $X_{indices}$ is a matrix with N*G dimension (N is the number of cells), where $x_{indices}$ is one of the vectors in $X_{indices}$. The cross product of $gene\ emb$ and x_{counts} outputs the weighted hidden vector $gene\ \ hidden_i$, $gene\ \ hidden_i \in \mathbb{R}^d$ (equation 2). The same process is applied to scATAC-seq data, as shown in equation (3-4).

$$gene\ \ emb = Embedding(x_{indices}) \tag{1.}$$

$$gene\ \ hidden_i = gene\ \ emb \times\ x_{counts} \tag{2}$$

$$peak\ \ emb = Embedding(y_{indices}) \tag{3}$$

$$peak\ \ hidden_j = peak\ \ emb \times\ y_{counts} \tag{4}$$

Then we use the attention mechanism to aggregate gene or peak embeddings. The input $gene\ \ hidden_i$ is passed through a multilayer perceptron with one hidden layer and a nonlinear tanh transformation. A cellular context vector $u \in \mathbb{R}^d$ then applies the dot product to $gene\ \ hidden_i$, using the softmax operation to obtain $gene\ \ attention_i \in \mathbb{R}^d$ (equation 5). The cell context vector u serves as an intermediate variable in computing hierarchical attention, which is a weighted value on 128-dimensional embeddings. Aggregation is then applied to the genes' vectors $gene\ \ hidden_i$ through weighted summation by $gene\ \ attention_i$, to obtain aggregated vectors, $RNA\ \ hidden$ (equation 6), with N*d dimension. The same process is applied to scATAC-seq data, as shown in equation (7-8).

$$gene\ \ attention_i = \mathrm{softmax}(\tanh(gene\ \ hidden_i) \cdot u) \tag{5}$$

$$RNA\ \ hidden = \sum_i (gene\ \ attention_i \times gene\ \ hidden_i) \tag{6}$$

$$peak\ \ attention_j = \mathrm{softmax}\big(\tanh\big(peak\ \ hidden_j\big) \cdot u\big) \tag{7}$$

$$ATAC\ \ hidden = \sum_j \big(peak\ \ attention_j \times peak\ \ hidden_j\big) \tag{8}$$

We apply the attention mechanism output to fed into a batch normalization layer followed by a dropout layer. Then a dense layer with ReLU activation projects to the final output of RNA teacher network, $Z_{teacher}^{RNA} \in \mathbb{R}^d$ (equation 9) and the final output of ATAC teacher network, $Z_{teacher}^{ATAC} \in \mathbb{R}^d$ (equation 10).

$$Z_{teacher}^{RNA} = Dense(RNA\ \ hidden) \tag{9}$$

$$Z_{teacher}^{ATAC} = Dense(ATAC\ \ hidden) \tag{10}$$

Student network

We also designed two student networks (RNA student network and ATAC student network) to learn coarse-grained representations respectively. The student network accepts only $X_{counts} \epsilon \mathbb{R}^{G}$ or $Y_{counts} \epsilon \mathbb{R}^{P}$, then passing a batch normalization layer followed by a dropout layer and a dense layer with ReLU activation projects to the final output of the RNA student network $Z_{student}^{RNA} \in \mathbb{R}^{d}$ (equation 11). The final output of the ATAC student network is $Z_{student}^{ATAC} \in \mathbb{R}^{d}$ (equation 12).

$$Z_{student}^{RNA} = Dense(x_{counts}) \tag{11}$$

$$Z_{student}^{ATAC} = Dense(y_{counts}) \tag{12}$$

Cross-modal contrastive loss

Contrast learning is implemented by the explicit comparison of the d-dimensional embedding (where d = 128 by default) of a cell on two modalities on a unit hypersphere. Positive sample pairs are created by taking two modal representations of a cell and pulling them together, while negative samples are created by taking different cells and widening the distance between them. Four different embeddings: $Z_{student}^{ATAC}$, $Z_{student}^{RNA}$, $Z_{teacher}^{ATAC}$, $Z_{teacher}^{RNA}$ are obtained using two independent asymmetric teacher-student networks.

Assume the embedding is obtained by the teacher network and the cosine similarity with L2 regularization of the two given embeddings (the embedding under the same network structure) is defined by equations (13-14). The positive pair as $cell_m$ (whose embedding is $z_{teacher_m}^{RNA} \in Z_{teacher}^{RNA}$) and $cell_{m^+}$(whose embedding is $z_{teacher_{m^+}}^{ATAC} \in Z_{teacher}^{ATAC}$). The NT-Xent loss represents the normalized temperature-scaled cross-entropy loss, as formalized by equation (15), where m and m^+ is a pair of positive samples. We randomly sample a min-batch of N cells and compute NT-Xent loss on pairs of cross-modal examples derived from the mini-batch, resulting in 2N data points. Given a positive pair, the other 2(N-1) cross-modal examples within a mini-batch are treated as negative examples. The calculation process of NT-Xent loss for the embedding obtained by the student network is the same, see equation (16-18). The full pretraining objective of Attune model sees equation (19).

$$s_{\alpha,\beta} = sim\left(z_{teacher_\alpha}, z_{teacher_\beta}\right) \tag{13}$$

$$s_{\alpha,\beta}^{+} = sim\left(z_{teacher_\beta}, z_{teacher_\alpha}\right) \tag{14}$$

where $sim(h_1, h_2)$ is defined as:

$$sim(h_1, h_2) = \frac{h_1^T h_2}{\tau \|h_1\| \|h_2\|}$$

$$\mathcal{L}_{teacher} = \frac{1}{2N}\sum_{m=1}^{N}[\ell(m,m^{+}) + \ell(m^{+},m)] \tag{15}$$

where $\ell(m, m^{+})$ is defined as:

$$\ell(m,m^{+}) = -log\frac{\exp(s_{m,m^{+}})}{\sum_{k=1}^{2N}\mathbb{1}_{[k\neq m]}[\exp(s_{k,m}) + \exp(s_{k,m^{+}})]}$$

where $\ell(m^{+}, m)$ is defined as:

$$\ell(m^{+},m) = -log\frac{\exp(s^{+}_{m,m^{+}})}{\sum_{k=1}^{2N}\mathbb{1}_{[k\neq m^{+}]}[\exp(s^{+}_{k,m^{+}}) + \exp(s^{+}_{k,m})]}$$

$$s_{\alpha,\beta} = sim\left(z_{student_{\alpha}}, z_{student_{\beta}}\right) \tag{16}$$

$$s^{+}_{\alpha,\beta} = sim\left(z_{student_{\beta}}, z_{student_{\alpha}}\right) \tag{17}$$

$$\mathcal{L}_{student} = \frac{1}{2N}\sum_{m=1}^{N}[\ell(m,m^{+}) + \ell(m^{+},m)] \tag{18}$$

$$\mathcal{L}_{pretrain} = \frac{\mathcal{L}_{teacher} + \mathcal{L}_{student}}{2} \tag{19}$$

where τ is the adjustable temperature coefficient, which can be used to scale the degree of pushing apart negative samples.

Joint representation and UMAP visualization

Contrast learning can integrate cells from different modalities together. Meanwhile, to join the cell embeddings of two modalities together, we concatenate $Z^{ATAC}_{teacher}$ to $Z^{RNA}_{teacher}$ to get the joint embedding matrix $Z_{joint} \in \mathbb{R}^{d}$, with 2N* d dimension (equation 20). Cell embeddings are visualized by UMAP using SCANPY.

$$Z_{joint} = Concatenate(Z^{RNA}_{teacher}, Z^{ATAC}_{teacher}) \tag{20}$$

Inference of gene-peak interaction

To recover gene-peak interaction, Attune employs contrastive learning as a pretraining procedure followed by a transformer decoder to model the relationship between peaks and genes. The transformer is a deep neural network structure for sequence modeling. The self-attention mechanism establishes attention connections between each token in a sequence, so the embedding of each token contains implicit context. Meanwhile, the cross-attention mechanism establishes attention connections between tokens in two sequences, enabling the model to extract the dependency between tokens in the two sequences. Both self-attention and cross-attention are adopted in our model. Self-attention captures intra-modality interaction, such as gene-gene relationships, while cross-attention models inter-modality interaction, which is the peak-gene relationship.

The input sequences of transformer are $gene\ hidden_i$ (N*G* d dimension) and $peak\ hidden_j$ (N*P*d dimension). Firstly, a CLS token is inserted at the beginning of a input sequence. It can also be understood as a weighted average of each token in a sequence. The embedding of CLS token is denoted as $gene\ hidden_{CLS} \epsilon \mathbb{R}^d$ and $peak\ hidden_{CLS} \epsilon \mathbb{R}^d$. Then, self-Attention is applied to $gene\ hidden_i$:

Step1: $gene\ hidden_i$ is fed into three multilayer perceptron to get vectors $Q \epsilon \mathbb{R}^d$, $K \epsilon \mathbb{R}^d$, $V\ \epsilon \mathbb{R}^d$.

Step2: Q applies the dot product to K, using the softmax operation to obtain $Attention\ weight_i$, $Attention\ weight_i \epsilon \mathbb{R}^{G+1}$, with N*(G+1)*(G+1) dimension. $self\ gene\ hidden_i \epsilon \mathbb{R}^d$ is defined as (21) with a N*(G+1) *d dimension.

$$self\ gene\ hidden_i = softmax\left(\frac{QK^T}{\sqrt{d}}\right)V \qquad (21)$$

Finally, Cross-Attention mechanism is applied to $peak\ hidden_j$ and $self\ gene\ hidden_i$: $cross\ gene\ hidden_i \epsilon \mathbb{R}^d$ is defined as (22) with a dimension of N*(G+1) *d.

$$cross\ gene\ hidden_i = softmax\left(\frac{QK^T + Attention\ mask}{\sqrt{d}}\right)V \qquad (22)$$

where Q is $self\ gene\ hidden_i$ and K, V are $peak\ hidden_j$. $Attention\ mask$ (with N*(G+1) *(P+1) dimension) is defined in *Attention mask* section. Cross attention weight is defined as (23):

$$Cross\ attention\ weight = softmax\left(\frac{QK^T + Attention\ mask}{\sqrt{d}}\right) \qquad (23)$$

Training objectives

We train transformer with two objectives function: contrastive loss between $gene\ hidden_{CLS}$ and $peak\ hidden_{CLS}$, RNA-ATAC modality matching loss. $gene\ hidden_{CLS}$ and $peak\ hidden_{CLS}$ are CLS tokens in different modalities with N*1*d dimension. They learn the weighted average embedding representing the entire genes or peaks. The purpose of comparing the two embeddings is to shorten the distance between the matched RNA-ATAC pairs globally. The calculation process of contrastive loss of CLS tokens, is described as equation (24-26).

$$s_{\alpha,\beta} = sim\left(gene\ hidden_{CLS_\alpha}, peak\ hidden_{CLS_\beta}\right) \qquad (24)$$

$$s_{\alpha,\beta}^{+} = sim\left(peak\ hidden_{CLS_\beta}, gene\ hidden_{CLS_\alpha}\right) \qquad (25)$$

$$\mathcal{L}_{CLS} = \frac{1}{2N}\sum_{m=1}^{N}\left[\ell(m, m^+) + \ell(m^+, m)\right] \qquad (26)$$

For the RNA-ATAC modality matching loss, the transformer is given a batch of matched RNA-ATAC pairs (positive pairs) or mismatched RNA-ATAC pairs (negative pairs). The network's goal is to identify whether a given pair is positive or negative. We feed the transformer with $gene\ hidden_i$ and $peak\ hidden_j$ pairs, and the probability of positive

and the probability of negative pair are set equal. A classifier is added on top of the CLS token's embedding $cross\ gene\ hidden_{CLS} \epsilon \mathbb{R}^d$ to predict a binary label gt, where gt is a 2-dimensional one-hot vector representing the ground-truth label. p^{match} is the probability of prediction. RNA-ATAC modality matching loss is defined in equation (27) and the full training objective of the transformer is presented in equation (28).

$$\mathcal{L}_{match} = \mathbb{E}_{(RNA,ATAC)\sim D} CE(p^{match}(RNA, ATAC), gt) \quad (27)$$

$$\mathcal{L}_{interaction} = \mathcal{L}_{CLS} + \mathcal{L}_{match} \quad (28)$$

Global cross-attention weight extraction

$Cross\ attention\ weight$ contains 2 global CLS tokens' attention weight, $CLS\ Attention\ weight^{RNA}$ (with N*(P+1) dimension) and $CLS\ Attention\ weight^{ATAC}$ (with N*(G+1) dimension). $Global\ Attention\ weight$ matrix (with G*P dimension) is defined as the dot product of $CLS\ Attention\ weight^{RNA}$ and $CLS\ Attention\ weight^{ATAC}$.

Modality prediction network

For the modality prediction task, a modality prediction network is finetuned on the Attune pretrained model to predict the RNA expression level. The network uses a relatively simple regression model, a multilayer perceptron. The input of the network is $Z^{ATAC}_{student} \epsilon \mathbb{R}^d$ and the number of hidden layer units is set to 1000, while the number of units in the last layer is set to G (the number of genes). The output of the network is the predicted value of gene counts, $X^{pred}_{counts} \epsilon \mathbb{R}^G$. The loss function of modality prediction network is defined as equation (29).

$$\mathcal{L}_{prediction} = MSE\left(X_{counts}, X^{pred}_{counts}\right) \quad (29)$$

Hyperparameter tuning

The learning rate in contrastive pretraining varies from 1×10^{-4} to 1×10^{-6} using Adam optimizer training for 20 epochs. For transformer, it trains for 5 epochs, whereas for the modality prediction network, it trains for 40 epochs. The temperature coefficient in NT-Xent loss is set to 0.1, the mini-batch size is set to 32, and the dimension of the embedding is 128. Comparative experiments are detailed in Table S5 and S6.

Metrics

Integration endeavors are assessed through an array of metrics, including mean average precision (MAP), cell type ASW, neighbor consistency (NC), Seurat alignment score (SAS), Batch ASW, graph connectivity (GC), biology conservation, omics mixing, overall integration score and FOSCTTM.

The mean average precision (MAP) furnishes a measure of the congruity between cell types in neighboring cells, thereby quantifying the accuracy of clustering outcomes with respect to cell type assignments[10].

Cell type ASW. To evaluate the integration outputs pertaining to cell types, cell type ASW

affords an assessment of the silhouette of cell type labels, suitably scaled to a value between 0 and 1[18].

Batch ASW evaluates the integration among multimodalities by computing cell modality labels, also scaled between 0 and 1[18].

Neighbor consistency (NC) measures the degree of intercellular neighbor retention after integrating multimodal data, ranging from 0 to 1, where higher values indicate better preservation[68].

Seurat alignment score (SAS) calculates the alignment score to assess how well two or more modalities have been aligned, with values ranging from 0 to 1, where higher values indicate better integration among modalities[69].

Graph connectivity (GC) evaluates the proximity of cells with the same identity across different modalities in the embedding. The GC ranges from 0 to 1, with higher values indicating better integration[10,19].

Fraction Of Samples Closer Than the True Match (FOSCTTM) measures the accuracy of modal alignment at the single-cell level in paired cells, with a range from 0 to 1, where lower values indicate higher accuracy. Studies like GLUE[10] and MMD-MA[70] have utilized FOSCTTM to assess performance.

Biology conservation is evaluated through MAP, cell type ASW, and NC, which collectively assess the biological conservation of integration. These metrics are min-max scaled, and their average is calculated as a single metric for biological conservation, as per equation (30)[10].

Omics mixing is evaluated using SAS, Batch ASW, and GC, which collectively assess the mixing performance of multi-modalities. These metrics are also min-max scaled, and their average is calculated as a single metric for omics mixing, as per equation (31)[10].

The overall integration score is computed as an overall weighted average of omics mixing and bio-conservation scores, as per equation (32)[10,19].

$$Biology\ \ conservation = \frac{scale(MAP) + scale(Cell\ \ type\ \ ASW) + scale(NC)}{3} \tag{30}$$

$$Omics\ \ mixing = \frac{scale(SAS) + scale(Omics\ \ layerASW) + scale(GC)}{3} \tag{31}$$

$$Overall\ \ integratin\ \ score = \ 0.6 \times Biology\ \ conservation + 0.4 \times Omics\ \ mixing \tag{32}$$

Various metrics are employed to evaluate the proposed solution in modality prediction. These metrics include the root-mean-square-error (RMSE), gene-wise Pearson correlation coefficient, and gene-wise Spearman correlation coefficient. The RMSE serves to appraise the precision of RNA expression prediction across individual cells[19], while gene-wise Pearson or Spearman correlation coefficients gauge the average per-gene correlation in

Polarbear[23] and BABEL[9].

Evaluation on inference of regulatory interaction

Promoter Capture Hi-C (PCHi-C) enables identification of long-range interactions between gene promoters and regulatory elements such as enhancers and other potential regulatory elements. Promoter interactomes are highly cell type specific and interacted regions quantitatively contribute to gene expression[27,71]. To demonstrate the potential of cross-modal association, we utilize the PCHi-C dataset of human primary hematopoietic cells. With the aim of consistency, only common cell types in the 10x Multiome and PCHi-C datasets, including T cells, B cells and monocytes, are considered for the comparison of different methods. Coordinates of interactions from PCHi-C binding matrix whose CHiCAGO interaction scores pass a cutoff of 5 in at least one cell type are lifted over[72] to Genome Reference Consortium Human Build 38 and ordered based on the distance between the midpoint of baited regions and other ends. The distance statistics of interactions are depicted in Figure 3a. Following the guidelines established in GLUE[10], we generate a truth set of peak-gene pairs supported by PCHi-C. These rules consider the proximity (within 1kb) of the gene promoter to a bait fragment and the peak's proximity (within 1kb) to the other-end fragment, along with significant interaction identified in PCHi-C. By considering the distance statistics of the PCHi-C data and controlling for noise introduced by abundant distal regions, we explore a range of gene-peak distances from 150kb to 1500kb (Supplementary Figure 4a) and determine 1200kb as the threshold for all subsequent experiments.

Specifically, during the calculation of the transformer module, we focus on the relationship between the gene promoter and peaks within a 1.2Mb region surrounding it. By masking the peaks outside this region, we encourage the transformer module to prioritize cross-modal attention within the adjacent regions of the gene. Attention weights are calculated for each gene-peak pair within 1200kb, and the "sklearn.metrics.roc_auc_score" function is utilized to assess whether the attention weights can reflect the promoter interactome.

Differential expression analysis and gene ontology enrichment

We use the "FindAllMakers" function of the Seurat[7] package to identify differentially expressed genes (DEGs) within each cell type (one versus others, p.adjust<0.01, log fold change>0.25). The top 10 and bottom 10 genes are chosen as top DEGs. Collection of gene sets (GO:BP in C5 category) from the Molecular Signatures Database (MSigDB)[73] is used for over-presentation analysis by clusterProfiler[74]. We keep an ontology with smaller pvalue when the geneID is repeated.

Pseudotime inference

We focus on the differentiation of transient amplifying cells and select cell types including

transit-amplifying cells (TAC), inner root sheath (IRS), medulla, and hair shaft from the SHARE-seq dataset of mouse skin, leading to 6k cells. 10 topics are then determined by cisTopic[75] using chromatin accessibility data. The default parameters are used, except for burin=120 and iterations=150 in the "runModels" functions. Z score is then computed by the "modelMatSelection" function as input of Palantir[76] for generating diffusion maps and pseudotime with n_components=10 of the "run_diffusion_maps" function.

Residual of modalities

The residual of each gene is calculated from normalized predicted RNA counts based on ATAC minus normalized measured RNA counts. We evaluate trends with pseudotime using a generalized additive model (GAM) and filter them based on standard deviation. We use the "argrelextrema" function in the "scikit-learn" package to define gene expression pattern.

Motif and regulon analysis

JASPAR CORE Vertebrata 2022 database[77] is selected for motif matching which contains 841 motifs. We set p.cutoff to 5×10^{-5} for filtering motifs. To illustrate the regulatory network, we use SCENIC[36], a workflow that exploits co-expression between genes and transcription factors, to analyze regulons in the SHARE-seq dataset. All modules are kept in the step of regulon prediction (add a parameter "-a") because of the known negative effect of Gli3. The AUC threshold is 0.05.

Soft cluster of chromatin accessibility data

Chromatin accessibility data at the cellular level is extremely sparse, resulting in dramatic fluctuations even within the same cell type, while it is coarse at the cell type level. To mitigate this issue, we aggregate cells into pseudo-bulk samples by dividing cells into 10 groups along pseudotime before soft clustering[38]. The cell-type composition of each group is shown in Figure 3i. Mean values are calculated and standardized for each pseudo-bulk sample. To estimate the optimized number of cluster centroids c, we perform soft clustering with a range of cluster numbers from 2 to 20. And 4 is determined as cluster number by the centroid distance plot. We extract alpha cores of each cluster using acore=0.5, which preserves 18 peaks while discarding discards cluster 1 and cluster 4. To increase the concentration of members, we ultimately select 12 peaks from cluster 2 and cluster 3.

Co-occurrence of genes and peaks

To identify the latent interacting genes of each cluster, we first calculate Spearman's

correlation between each peak in clusters and genes, and then average the values of each gene for each cluster. With the purpose of eliminating contingency, 50 peaks for each peak are selected as the background based on GC content and coverage using the "getBackgroundPeaks" function of ChromVAR[78] package. Wilcoxon rank sum test is performed to examine the difference.

Running benchmarks

GLUE (v0.3.2)[10], uniPort (v1.1.2)[79], Cobolt[80] (v1.0.1), MinNet[81], scJoint[20], MultiVI (v0.19.0)[8], sciCAN[82] were conducted using the Python (v3.6). We followed the tutorials for each method: GLUE (https://scglue.readthedocs.io/en/latest/tutorials.html), uniPort (https://uniport.readthedocs.io), Cobolt (https://github.com/epurdom/cobolt/blob/master/docs/tutorial.ipynb), scJoint (https://github.com/SydneyBioX/scJoint/tree/main/tutorial), MultiVI (https://docs.scvi-tools.org/en/stable/tutorials/notebooks/MultiVI_tutorial.html), sciCAN (https://github.com/rpmccordlab/sciCAN). We conducted Seurat V3[83] using the R (v4.1.2) and the tutorial at https://satijalab.org/seurat/articles/atacseq_integration_vignette.html.

All the methods were used the default settings and data preprocessing steps as recommended. Notably, scJoint, Seurat V3, sciCAN and MinNet require converting peak counts into gene activity scores (https://stuartlab.org/signac/articles/pbmc_vignette.html#create-a-gene-activity-matrix).

Key Points

- The study introduces 'Attune', a novel model that leverages contrastive learning for the effective integration of scRNA-seq and scATAC-seq data. Attune demonstrates enhanced performance in integration and downstream cross-modal predictive tasks, showing clear advancements over current approaches.
- Employing a transformer-based architecture, Attune effectively deciphers complex regulatory interactions among genomic features, thereby enriching our understanding of cellular differentiation and lineage specification.
- Attune further explores the concept of chromatin potential, illustrating the impact of temporal delays between chromatin accessibility and gene expression on cell fate decisions, particularly during critical stages of lineage commitment.

Author contributions statement

M.Y. conceived the problem and designed the study. Y.Y. and Q.S. performed bioinformatics analysis. C.X. performed algorithm design and deep learning experiments. Y.Y. and Q.S. and C.X. wrote the manuscript.

Code availability

Attune is written in Python using the TensorFlow library. The source code is available on Github at https://github.com/melobio/Attune.

Author Biographies

Yueyuxiao Yang receives the MS degree from Nation Institute of Applied Sciences (INSA), Lyon, France. Before joining INSA, he got his BSc degree at Harbin Institute of Technology (HIT). His current research interests include deep learning and single cell multi-omics.

References

1. Nimmo, R.A., May, G.E., and Enver, T. (2015). Primed and ready: understanding lineage commitment through single cell analysis. Trends in Cell Biology *25*, 459-467. https://doi.org/10.1016/j.tcb.2015.04.004.
2. Li, B., Carey, M., and Workman, J.L. (2007). The Role of Chromatin during Transcription. Cell *128*, 707-719. 10.1016/j.cell.2007.01.015.
3. Ma, S., Zhang, B., LaFave, L.M., Earl, A.S., Chiang, Z., Hu, Y., Ding, J., Brack, A., Kartha, V.K., Tay, T., et al. (2020). Chromatin Potential Identified by Shared Single-Cell Profiling of RNA and Chromatin. Cell *183*, 1103-1116.e1120. 10.1016/j.cell.2020.09.056.
4. Chen, S., Lake, B.B., and Zhang, K. (2019). High-throughput sequencing of the transcriptome and chromatin accessibility in the same cell. Nature Biotechnology *37*, 1452-1457. 10.1038/s41587-019-0290-0.
5. Liu, L., Liu, C., Quintero, A., Wu, L., Yuan, Y., Wang, M., Cheng, M., Leng, L., Xu, L., Dong, G., et al. (2019). Deconvolution of single-cell multi-omics layers reveals regulatory heterogeneity. Nature Communications *10*, 470. 10.1038/s41467-018-08205-7.
6. Zhu, C., Preissl, S., and Ren, B. (2020). Single-cell multimodal omics: the power of many. Nature Methods *17*, 11-14. 10.1038/s41592-019-0691-5.
7. Hao, Y., Hao, S., Andersen-Nissen, E., Mauck, W.M., III, Zheng, S., Butler, A., Lee, M.J., Wilk, A.J., Darby, C., Zager, M., et al. (2021). Integrated analysis of multimodal single-cell data. Cell *184*, 3573-3587.e3529. 10.1016/j.cell.2021.04.048.
8. Ashuach, T., Gabitto, M.I., Jordan, M.I., and Yosef, N. (2021). MultiVI: deep generative model for the integration of multi-modal data. bioRxiv, 2021.2008.2020.457057. 10.1101/2021.08.20.457057.
9. Wu, K.E., Yost, K.E., Chang, H.Y., and Zou, J. (2021). BABEL enables cross-modality translation between multiomic profiles at single-cell resolution. Proceedings of the National Academy of Sciences *118*, e2023070118. 10.1073/pnas.2023070118.
10. Cao, Z.-J., and Gao, G. (2022). Multi-omics single-cell data integration and regulatory inference with graph-linked embedding. Nature Biotechnology *40*, 1458-1466. 10.1038/s41587-022-01284-4.
11. Lynch, A.W., Theodoris, C.V., Long, H.W., Brown, M., Liu, X.S., and Meyer, C.A. (2022). MIRA: joint regulatory modeling of multimodal expression and chromatin accessibility in single cells. Nature Methods *19*, 1097-1108. 10.1038/s41592-022-01595-z.
12. Dou, Z.-Y., Xu, Y., Gan, Z., Wang, J., Wang, S., Wang, L., Zhu, C., Zhang, P., Yuan, L., and Peng, N. An empirical study of training end-to-end vision-and-language transformers. 2022. pp. 18166-18176.
13. Radford, A., Kim, J.W., Hallacy, C., Ramesh, A., Goh, G., Agarwal, S., Sastry, G., Askell, A., Mishkin, P., and Clark, J. Learning transferable visual models from natural language supervision. 2021. (PMLR), pp. 8748-8763.
14. Li, J., Selvaraju, R., Gotmare, A., Joty, S., Xiong, C., and Hoi, S.C.H. (2021). Align before fuse: Vision and language representation learning with momentum distillation. Advances in neural information processing systems *34*, 9694-9705.
15. Yang, M., Yang, Y., Xie, C., Ni, M., Liu, J., Yang, H., Mu, F., and Wang, J. (2022). Contrastive learning enables rapid mapping to multimodal single-cell atlas of multimillion scale.

Nature Machine Intelligence *4*, 696-709. 10.1038/s42256-022-00518-z.

16. Vaswani, A., Shazeer, N., Parmar, N., Uszkoreit, J., Jones, L., Gomez, A.N., Kaiser, Ł., and Polosukhin, I. (2017). Attention is all you need. Advances in neural information processing systems *30*.
17. Wang, T., and Isola, P. Understanding contrastive representation learning through alignment and uniformity on the hypersphere. 2020. (PMLR), pp. 9929-9939.
18. Luecken, M.D., Büttner, M., Chaichoompu, K., Danese, A., Interlandi, M., Müller, M.F., Strobl, D.C., Zappia, L., Dugas, M., and Colomé-Tatché, M. (2022). Benchmarking atlas-level data integration in single-cell genomics. Nature methods *19*, 41-50.
19. Luecken, M.D., Burkhardt, D.B., Cannoodt, R., Lance, C., Agrawal, A., Aliee, H., Chen, A.T., Deconinck, L., Detweiler, A.M., and Granados, A.A. A sandbox for prediction and integration of dna, rna, and proteins in single cells. 2021.
20. Lin, Y., Wu, T.-Y., Wan, S., Yang, J.Y.H., Wong, W.H., and Wang, Y.X.R. (2022). scJoint integrates atlas-scale single-cell RNA-seq and ATAC-seq data with transfer learning. Nature Biotechnology *40*, 703-710. 10.1038/s41587-021-01161-6.
21. Lotfollahi, M., Litinetskaya, A., and Theis, F.J. (2022). Multigrate: single-cell multi-omic data integration. BioRxiv, 2022-2003.
22. Hao, Y., Stuart, T., Kowalski, M.H., Choudhary, S., Hoffman, P., Hartman, A., Srivastava, A., Molla, G., Madad, S., and Fernandez-Granda, C. (2023). Dictionary learning for integrative, multimodal and scalable single-cell analysis. Nature Biotechnology, 1-12.
23. Zhang, R., Meng-Papaxanthos, L., Vert, J.-p., and Noble, W.S. (2022). Multimodal Single-Cell Translation and Alignment with Semi-Supervised Learning. Journal of Computational Biology *29*, 1198-1212.
24. Trevino, A.E., Müller, F., Andersen, J., Sundaram, L., Kathiria, A., Shcherbina, A., Farh, K., Chang, H.Y., Pașca, A.M., Kundaje, A., et al. (2021). Chromatin and gene-regulatory dynamics of the developing human cerebral cortex at single-cell resolution. Cell *184*, 5053-5069.e5023. 10.1016/j.cell.2021.07.039.
25. Dekker, J., and Heard, E. (2015). Structural and functional diversity of Topologically Associating Domains. FEBS Letters *589*, 2877-2884. https://doi.org/10.1016/j.febslet.2015.08.044.
26. Dixon, J.R., Selvaraj, S., Yue, F., Kim, A., Li, Y., Shen, Y., Hu, M., Liu, J.S., and Ren, B. (2012). Topological domains in mammalian genomes identified by analysis of chromatin interactions. Nature *485*, 376-380. 10.1038/nature11082.
27. Javierre, B.M., Burren, O.S., Wilder, S.P., Kreuzhuber, R., Hill, S.M., Sewitz, S., Cairns, J., Wingett, S.W., Várnai, C., Thiecke, M.J., et al. (2016). Lineage-Specific Genome Architecture Links Enhancers and Non-coding Disease Variants to Target Gene Promoters. Cell *167*, 1369-1384.e1319. 10.1016/j.cell.2016.09.037.
28. Krivega, I., and Dean, A. (2012). Enhancer and promoter interactions-long distance calls. Current Opinion in Genetics & Development *22*, 79-85. 10.1016/j.gde.2011.11.001.
29. van Arensbergen, J., van Steensel, B., and Bussemaker, H.J. (2014). In search of the determinants of enhancer-promoter interaction specificity. Trends in Cell Biology *24*, 695-702. 10.1016/j.tcb.2014.07.004.
30. Pliner, H.A., Packer, J.S., McFaline-Figueroa, J.L., Cusanovich, D.A., Daza, R.M., Aghamirzaie, D., Srivatsan, S., Qiu, X., Jackson, D., Minkina, A., et al. (2018). Cicero Predicts cis-

Regulatory DNA Interactions from Single-Cell Chromatin Accessibility Data. Molecular Cell *71*, 858-871.e858. 10.1016/j.molcel.2018.06.044.
31. Li, C., Virgilio, M.C., Collins, K.L., and Welch, J.D. (2022). Multi-omic single-cell velocity models epigenome–transcriptome interactions and improves cell fate prediction. Nature Biotechnology. 10.1038/s41587-022-01476-y.
32. Brownell, I. Guevara e, Bai CB, Loomis CA, Joyner AL (2011) Nerve-derived sonic hedgehog defines a niche for hair follicle stem cells capable of becoming epidermal stem cells. Cell Stem Cell *8*, 552-565.
33. Mill, P., Mo, R., Fu, H., Grachtchouk, M., Kim, P.C.W., Dlugosz, A.A., and Hui, C.-c. (2003). Sonic hedgehog-dependent activation of Gli2 is essential for embryonic hair follicle development. Genes & development *17*, 282-294.
34. Matissek, S.J., and Elsawa, S.F. (2020). GLI3: a mediator of genetic diseases, development and cancer. Cell Communication and Signaling *18*, 1-20.
35. Chandramouli, A., Hatsell, S.J., Pinderhughes, A., Koetz, L., and Cowin, P. (2013). Gli activity is critical at multiple stages of embryonic mammary and nipple development. PLoS One *8*, e79845.
36. Aibar, S., González-Blas, C.B., Moerman, T., Huynh-Thu, V.A., Imrichova, H., Hulselmans, G., Rambow, F., Marine, J.-C., Geurts, P., Aerts, J., et al. (2017). SCENIC: single-cell regulatory network inference and clustering. Nature Methods *14*, 1083-1086. 10.1038/nmeth.4463.
37. Futschik, M.E., and Carlisle, B. (2005). NOISE-ROBUST SOFT CLUSTERING OF GENE EXPRESSION TIME-COURSE DATA. Journal of Bioinformatics and Computational Biology *03*, 965-988. 10.1142/S0219720005001375.
38. Kumar, L., and Futschik, M.E. (2007). Mfuzz: a software package for soft clustering of microarray data. Bioinformation *2*, 5.
39. Cui, C.-Y., Yin, M., Sima, J., Childress, V., Michel, M., Piao, Y., and Schlessinger, D. (2014). Involvement of Wnt, Eda and Shh at defined stages of sweat gland development. Development *141*, 3752-3760.
40. Avilés, E.C., Wilson, N.H., and Stoeckli, E.T. (2013). Sonic hedgehog and Wnt: antagonists in morphogenesis but collaborators in axon guidance. Frontiers in cellular neuroscience *7*, 86.
41. Dave, R.K., Ellis, T., Toumpas, M.C., Robson, J.P., Julian, E., Adolphe, C., Bartlett, P.F., Cooper, H.M., Reynolds, B.A., and Wainwright, B.J. (2011). Sonic hedgehog and notch signaling can cooperate to regulate neurogenic divisions of neocortical progenitors. PloS one *6*, e14680.
42. Adam, R.C., Yang, H., Ge, Y., Lien, W.-H., Wang, P., Zhao, Y., Polak, L., Levorse, J., Baksh, S.C., and Zheng, D. (2018). Temporal layering of signaling effectors drives chromatin remodeling during hair follicle stem cell lineage progression. Cell stem cell *22*, 398-413.
43. Hu, X.-M., Li, Z.-X., Zhang, D.-Y., Yang, Y.-C., Fu, S.-a., Zhang, Z.-Q., Yang, R.-H., and Xiong, K. (2021). A systematic summary of survival and death signalling during the life of hair follicle stem cells. Stem cell research & therapy *12*, 1-29.
44. (!!! INVALID CITATION !!! 37,42-44).
45. Lefebvre, V. (2019). Roles and regulation of SOX transcription factors in skeletogenesis. Current topics in developmental biology *133*, 171-193.

46. Tan, Z., Niu, B., Tsang, K.Y., Melhado, I.G., Ohba, S., He, X., Huang, Y., Wang, C., McMahon, A.P., and Jauch, R. (2018). Synergistic co-regulation and competition by a SOX9-GLI-FOXA phasic transcriptional network coordinate chondrocyte differentiation transitions. PLoS genetics *14*, e1007346.
47. Liu, C.-F., and Lefebvre, V. (2015). The transcription factors SOX9 and SOX5/SOX6 cooperate genome-wide through super-enhancers to drive chondrogenesis. Nucleic acids research *43*, 8183-8203.
48. Bunt, J., Osinski, J.M., Lim, J.W.C., Vidovic, D., Ye, Y., Zalucki, O., O'Connor, T.R., Harris, L., Gronostajski, R.M., and Richards, L.J. (2017). Combined allelic dosage of Nfia and Nfib regulates cortical development. Brain and Neuroscience Advances *1*, 2398212817739433.
49. Pjanic, M., Pjanic, P., Schmid, C., Ambrosini, G., Gaussin, A., Plasari, G., Mazza, C., Bucher, P., and Mermod, N. (2011). Nuclear factor I revealed as family of promoter binding transcription activators. BMC genomics *12*, 1-10.
50. Fraser, J., Essebier, A., Brown, A.S., Davila, R.A., Harkins, D., Zalucki, O., Shapiro, L.P., Penzes, P., Wainwright, B.J., and Scott, M.P. (2020). Common regulatory targets of NFIA, NFIX and NFIB during postnatal cerebellar development. The Cerebellum *19*, 89-101.
51. Moser, M., Binder, O., Wu, Y., Aitsebaomo, J., Ren, R., Bode, C., Bautch, V.L., Conlon, F.L., and Patterson, C. (2003). BMPER, a novel endothelial cell precursor-derived protein, antagonizes bone morphogenetic protein signaling and endothelial cell differentiation. Molecular and cellular biology *23*, 5664-5679.
52. McGarvey, A.C., Rybtsov, S., Souilhol, C., Tamagno, S., Rice, R., Hills, D., Godwin, D., Rice, D., Tomlinson, S.R., and Medvinsky, A. (2017). A molecular roadmap of the AGM region reveals BMPER as a novel regulator of HSC maturation. Journal of Experimental Medicine *214*, 3731-3751.
53. Kuschel, S., Rüther, U., and Theil, T. (2003). A disrupted balance between Bmp/Wnt and Fgf signaling underlies the ventralization of the Gli3 mutant telencephalon. Developmental biology *260*, 484-495.
54. Zhao, H., Zhou, W., Yao, Z., Wan, Y., Cao, J., Zhang, L., Zhao, J., Li, H., Zhou, R., and Li, B. (2015). Foxp1/2/4 regulate endochondral ossification as a suppresser complex. Developmental biology *398*, 242-254.
55. Gan, X., Wang, J., Wang, C., Sommer, E., Kozasa, T., Srinivasula, S., Alessi, D., Offermanns, S., Simon, M.I., and Wu, D. (2012). PRR5L degradation promotes mTORC2-mediated PKC-δ phosphorylation and cell migration downstream of Gα12. Nature cell biology *14*, 686-696.
56. Riobo, N.A., Haines, G.M., and Emerson Jr, C.P. (2006). Protein kinase C-δ and mitogen-activated protein/extracellular signal–regulated kinase-1 control GLI activation in Hedgehog signaling. Cancer research *66*, 839-845.
57. Maiti, S., Mondal, S., Satyavarapu, E.M., and Mandal, C. (2017). mTORC2 regulates hedgehog pathway activity by promoting stability to Gli2 protein and its nuclear translocation. Cell Death & Disease *8*, e2926-e2926.
58. Khajavi, M., Zhou, Y., Schiffer, A.J., Bazinet, L., Birsner, A.E., Zon, L., and D'Amato, R.J. (2021). Identification of Basp1 as a novel angiogenesis-regulating gene by multi-model system studies. The FASEB Journal *35*, e21404.
59. Gao, Y., Banik, D.D., Muna, M.M., Roberts, S.G.E., and Medler, K.F. (2019). The WT1–BASP1

complex is required to maintain the differentiated state of taste receptor cells. Life science alliance *2*.

60. Wu, J., Zhu, P., Lu, T., Du, Y., Wang, Y., He, L., Ye, B., Liu, B., Yang, L., and Wang, J. (2019). The long non-coding RNA LncHDAC2 drives the self-renewal of liver cancer stem cells via activation of Hedgehog signaling. Journal of hepatology *70*, 918-929.
61. Peñagarikano, O., Abrahams, Brett S., Herman, Edward I., Winden, Kellen D., Gdalyahu, A., Dong, H., Sonnenblick, Lisa I., Gruver, R., Almajano, J., Bragin, A., et al. (2011). Absence of CNTNAP2 Leads to Epilepsy, Neuronal Migration Abnormalities, and Core Autism-Related Deficits. Cell *147*, 235-246. https://doi.org/10.1016/j.cell.2011.08.040.
62. Junge, H.J., Yung, A.R., Goodrich, L.V., and Chen, Z. (2016). Netrin1/DCC signaling promotes neuronal migration in the dorsal spinal cord. Neural Development *11*, 19. 10.1186/s13064-016-0074-x.
63. Bando, Y., Ishibashi, M., Yamagishi, S., Fukuda, A., and Sato, K. (2022). Orchestration of Ion Channels and Transporters in Neocortical Development and Neurological Disorders. Frontiers in Neuroscience *16*.
64. Wen, L., Li, G., Huang, T., Geng, W., Pei, H., Yang, J., Zhu, M., Zhang, P., Hou, R., Tian, G., et al. (2022). Single-cell technologies: From research to application. The Innovation *3*, 100342. https://doi.org/10.1016/j.xinn.2022.100342.
65. Saelens, W., Cannoodt, R., Todorov, H., and Saeys, Y. (2019). A comparison of single-cell trajectory inference methods. Nature Biotechnology *37*, 547-554. 10.1038/s41587-019-0071-9.
66. Wolf, F.A., Angerer, P., and Theis, F.J. (2018). SCANPY: large-scale single-cell gene expression data analysis. Genome Biology *19*, 15. 10.1186/s13059-017-1382-0.
67. Yang, Z., Yang, D., Dyer, C., He, X., Smola, A., and Hovy, E. Hierarchical attention networks for document classification. 2016. pp. 1480-1489.
68. Xu, C., Lopez, R., Mehlman, E., Regier, J., Jordan, M.I., and Yosef, N. (2021). Probabilistic harmonization and annotation of single-cell transcriptomics data with deep generative models. Molecular systems biology *17*, e9620.
69. Butler, A., Hoffman, P., Smibert, P., Papalexi, E., and Satija, R. (2018). Integrating single-cell transcriptomic data across different conditions, technologies, and species. Nature biotechnology *36*, 411-420.
70. Singh, R., Demetci, P., Bonora, G., Ramani, V., Lee, C., Fang, H., Duan, Z., Deng, X., Shendure, J., and Disteche, C. Unsupervised manifold alignment for single-cell multi-omics data. 2020. pp. 1-10.
71. Schoenfelder, S., Javierre, B.-M., Furlan-Magaril, M., Wingett, S.W., and Fraser, P. (2018). Promoter capture Hi-C: high-resolution, genome-wide profiling of promoter interactions. JoVE (Journal of Visualized Experiments), e57320.
72. Hinrichs, A.S., Karolchik, D., Baertsch, R., Barber, G.P., Bejerano, G., Clawson, H., Diekhans, M., Furey, T.S., Harte, R.A., Hsu, F., et al. (2006). The UCSC Genome Browser Database: update 2006. Nucleic Acids Research *34*, D590-D598. 10.1093/nar/gkj144.
73. Subramanian, A., Tamayo, P., Mootha, V.K., Mukherjee, S., Ebert, B.L., Gillette, M.A., Paulovich, A., Pomeroy, S.L., Golub, T.R., Lander, E.S., and Mesirov, J.P. (2005). Gene set enrichment analysis: A knowledge-based approach for interpreting genome-wide expression profiles. Proceedings of the National Academy of Sciences *102*, 15545-15550.

10.1073/pnas.0506580102.
74. Wu, T., Hu, E., Xu, S., Chen, M., Guo, P., Dai, Z., Feng, T., Zhou, L., Tang, W., Zhan, L., et al. (2021). clusterProfiler 4.0: A universal enrichment tool for interpreting omics data. The Innovation *2*. 10.1016/j.xinn.2021.100141.
75. Bravo González-Blas, C., Minnoye, L., Papasokrati, D., Aibar, S., Hulselmans, G., Christiaens, V., Davie, K., Wouters, J., and Aerts, S. (2019). cisTopic: cis-regulatory topic modeling on single-cell ATAC-seq data. Nature Methods *16*, 397-400. 10.1038/s41592-019-0367-1.
76. Setty, M., Kiseliovas, V., Levine, J., Gayoso, A., Mazutis, L., and Pe'er, D. (2019). Characterization of cell fate probabilities in single-cell data with Palantir. Nature Biotechnology *37*, 451-460. 10.1038/s41587-019-0068-4.
77. Castro-Mondragon, J.A., Riudavets-Puig, R., Rauluseviciute, I., Berhanu Lemma, R., Turchi, L., Blanc-Mathieu, R., Lucas, J., Boddie, P., Khan, A., Manosalva Pérez, N., et al. (2022). JASPAR 2022: the 9th release of the open-access database of transcription factor binding profiles. Nucleic Acids Research *50*, D165-D173. 10.1093/nar/gkab1113.
78. Schep, A.N., Wu, B., Buenrostro, J.D., and Greenleaf, W.J. (2017). chromVAR: inferring transcription-factor-associated accessibility from single-cell epigenomic data. Nature Methods *14*, 975-978. 10.1038/nmeth.4401.
79. Cao, K., Gong, Q., Hong, Y., and Wan, L. (2022). A unified computational framework for single-cell data integration with optimal transport. Nature Communications *13*, 1-15.
80. Gong, B., Zhou, Y., and Purdom, E. (2021). Cobolt: Joint analysis of multimodal single-cell sequencing data. bioRxiv, 2021.2004.2003.438329. 10.1101/2021.04.03.438329.
81. Liu, C., Wang, L., and Liu, Z. (2022). Single-cell Multi-omics Integration for Unpaired Data by a Siamese Network with Graph-based Contrastive Loss. bioRxiv, 2022.2006.2007.495170. 10.1101/2022.06.07.495170.
82. Xu, Y., Begoli, E., and McCord, R.P. (2021). sciCAN: Single-cell chromatin accessibility and gene expression data integration via Cycle-consistent Adversarial Network. bioRxiv.
83. Stuart, T., Butler, A., Hoffman, P., Hafemeister, C., Papalexi, E., Mauck, W.M., Hao, Y., Stoeckius, M., Smibert, P., and Satija, R. (2019). Comprehensive Integration of Single-Cell Data. Cell *177*, 1888-1902.e1821. 10.1016/j.cell.2019.05.031.

Figures

Figure 1. **The overall framework of Attune. (a)** Schematic of Attune and downstream tasks. Each cell has two modalities from scRNA-seq and scATAC-seq (the color indicates different cells and the shape indicate different modalities) and each cell's two modalities are positive pairs to be pulled together while other cells are pushed away via contrastive learning. Learned cell embeddings after integration are fed into downstream tasks including cross-modality prediction, differentiation analysis and inference of gene-peak interaction.

(b) Overview of Attune model and transformer-based decoder architecture. Attune consists of two asymmetric teacher-student networks. We propose cross-modal contrastive loss to integrate the cell embeddings of matched RNA-ATAC pairs into a common space. We finetune Attune via a transformer-based decoder for recovering regulatory events, which is used to learn multimodal interactions between peak and gene. We extract gene embedding and peak embedding from teacher networks and the green block denotes each gene or peak and the yellow block denotes CLS token. A CLS contrastive loss is applied to shorten the distance between the matched RNA-ATAC pairs globally. The Cosine similarity between cells in-batch is obtained by CLS contrastive learning and the cells pair with highest similarity is taken as positive pairs (matched) and cells with low similarity is randomly selected as negative pairs (unmatched). Then input the positive pairs and negative pairs into transformer in turn. Concatenate the output of the positive pairs and negative pairs in the transformer and feed it into dense layer followed by softmax for two-class prediction (positive pairs or negative pairs). Then calculate the cross-entropy with one-hot label (1 for positive pairs) for RNA-ATAC modality matching.

Figure 2. **Comprehensive benchmarks of integration and cross-modal prediction performance. (a)** Biology conservation score versus omics integration score for different methods (repeated five times with different random seeds) on 10x Multiome dataset (n=11,909 cells, left) and SHARE-seq dataset (n=32,231 cells, right). **(b)** Comparison of overall integration score on the 10x Multiome dataset and SHARE-seq dataset on the left and comparison of FOSCTTM on the right. Error bars represent the 95% confidence interval. **(c)** Alignment–uniformity plot for Attune, GLUE, LIGER, Seurat, Cobolt, MinNet, scJoint and MultiVI on 10x Multiome dataset. There are five replicates (represented by dots) for each method. **(d)** Integration performance of Attune under different numbers of gene and peak settings on 10x Multiome dataset. The size of the dot indicates the number of peaks, and the color indicates the number of genes. **(e)** Ablation study of Attune feature

embeddings under different numbers of dimension settings. The bar shows the overall integration score for 10x Multiome datasets (repeated five times with different random seeds). Error bars represent the 95% confidence interval. **(f)** Comparing the performance of modality predictions (fivefold cross-validation) in terms of gene-wise Pearson correlation coefficient, gene-wise Spearman correlation coefficient and RMSE on the 10x Multiome dataset. Comparison is made with Babel and Polarbear. **(g)** Performance comparison of modality prediction against the top winners in the NeurIPS 2021 competition. **(h)** Ablation study on contrast between different modalities or networks; RtAs_RsAt represents one set of contrast learning between RNA teacher network and ATAC student network and another set of contrast learning between RNA student network and ATAC teacher network. The violin plots show the modality prediction performance for 10x Multiome datasets (fivefold cross-validation). In the boxplots included in violin plots: center line, median; box, interquartile range (IQR; the range between the 25th and 75th percentiles); whiskers, 1.5 × IQR; dots, outliers.

Figure 3. **Regulatory interaction analysis of Attune on 10x Multiome dataset. (a)** Distribution of distance between peaks and promoters in the PCHi-C dataset. **(b)** Comparison of AUROC among six methods, including Attune + Transformer, PCA + Transformer, GLUE, Spearman, LASSO, and Cicero, on the PCHi-C dataset. The threshold for peak-promoter distance is set to 1200kb. **(c)** Attention weight is in descending order and the cutoff of the top 10% is labeled on the 10x Multiome dataset. **(d)** Differential expression of 466 DPAGs in the 10x Multiome dataset, with some top DEGs highlighted. **(e)** Enrichment analysis of biological process within differentially expressed genes. **(f)** Heatmap of gene expression or accessibility with cell types. Each row represents a gene-peak pair extracted by attention weight. **(g)** UMAP visualization of prediction, measurement, and the residual value of Gli3 gene on SHARE-seq dataset. **(h)** Trend of Gli3's residual from SHARE-seq dataset along the pseudotime. The maximum residual value is highlighted. **(i)** Composition of cell types in ten pseudo samples.

Figure 4. **Regulatory mechanism of hair follicle maturation. (a)** Four clusters of Gli3 peaks by soft cluster. **(b)** Link plot of peaks with high attention weight to Gli3. Peaks of cluster 2/3 are colored. **(c)** Chromatin-accessible state of 12 peaks within cluster 2 (top) or cluster 3 (bottom). **(d)** Spearman's correlation between peaks in cluster 2 or cluster 3 and genes. **(e)** Similar transit pattern between peaks of two clusters and genes. Each row shows the normalized expression or accessibility score of a gene or peak.

Figure 5. **State transition of neonatal neurons in the human cortex. (a)** Blueprint of Hh-centric multiple pathways involved in hair follicle development. Genes associated with peaks in cluster 2 or cluster 3 are highlighted in red. The background of pathways such as NF, Wnt, Bmp and Hh are colored. **(b)** Cosine similarity between each cell from two modalities on the human cortex dataset is calculated. Cells are arranged chronologically. **(c)** UMAP visualization of cosine distance from matched cells between RNA and ATAC modalities.

Supplementary Data

Supplementary information is available for this paper in an additional Supplementary File, including 9 Supplementary Figures and 16 Supplementary Tables.

Click here to access/download;Figure;Figure1.png

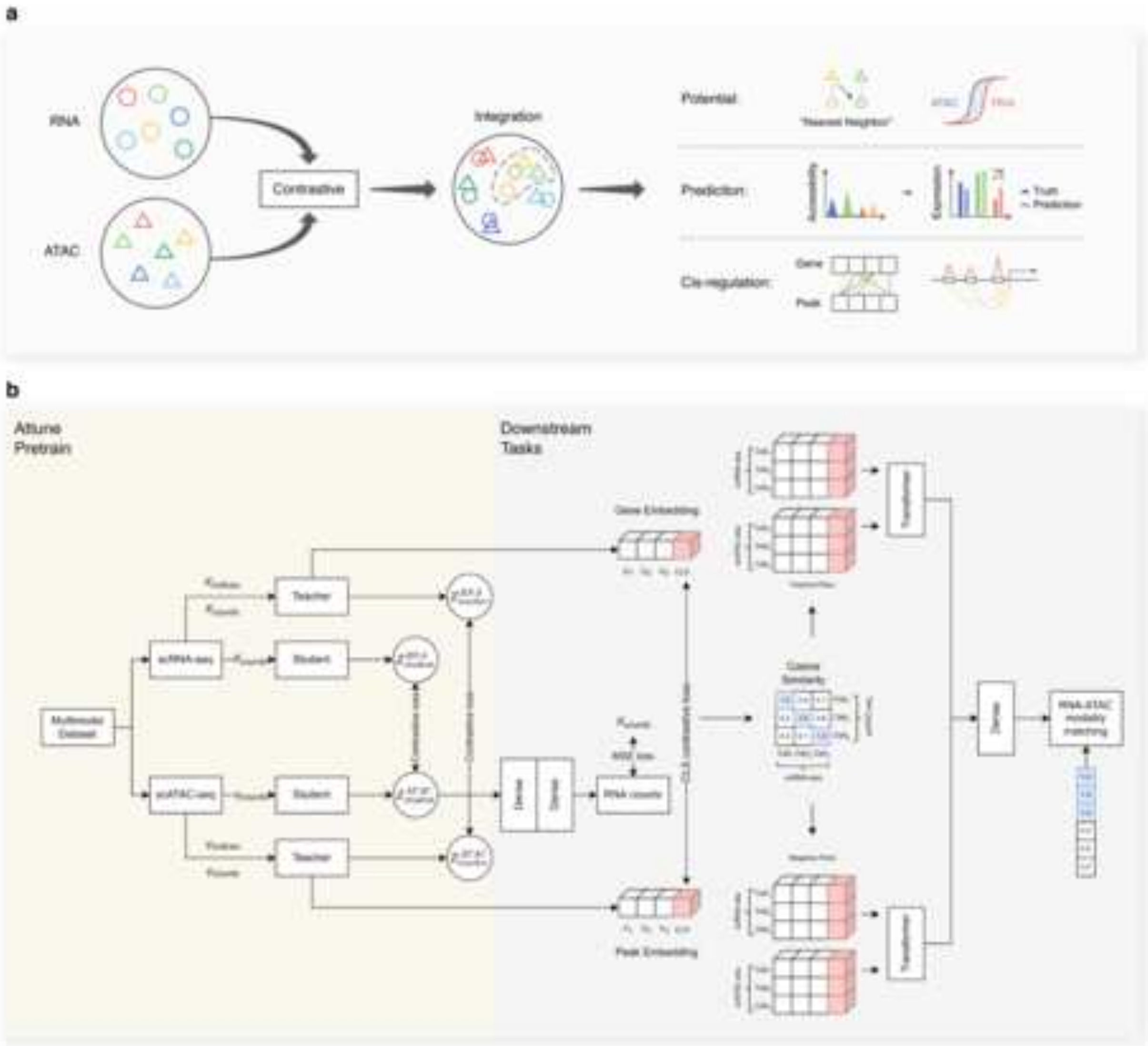

Figure2

Click here to access/download;Figure;Figure2.png

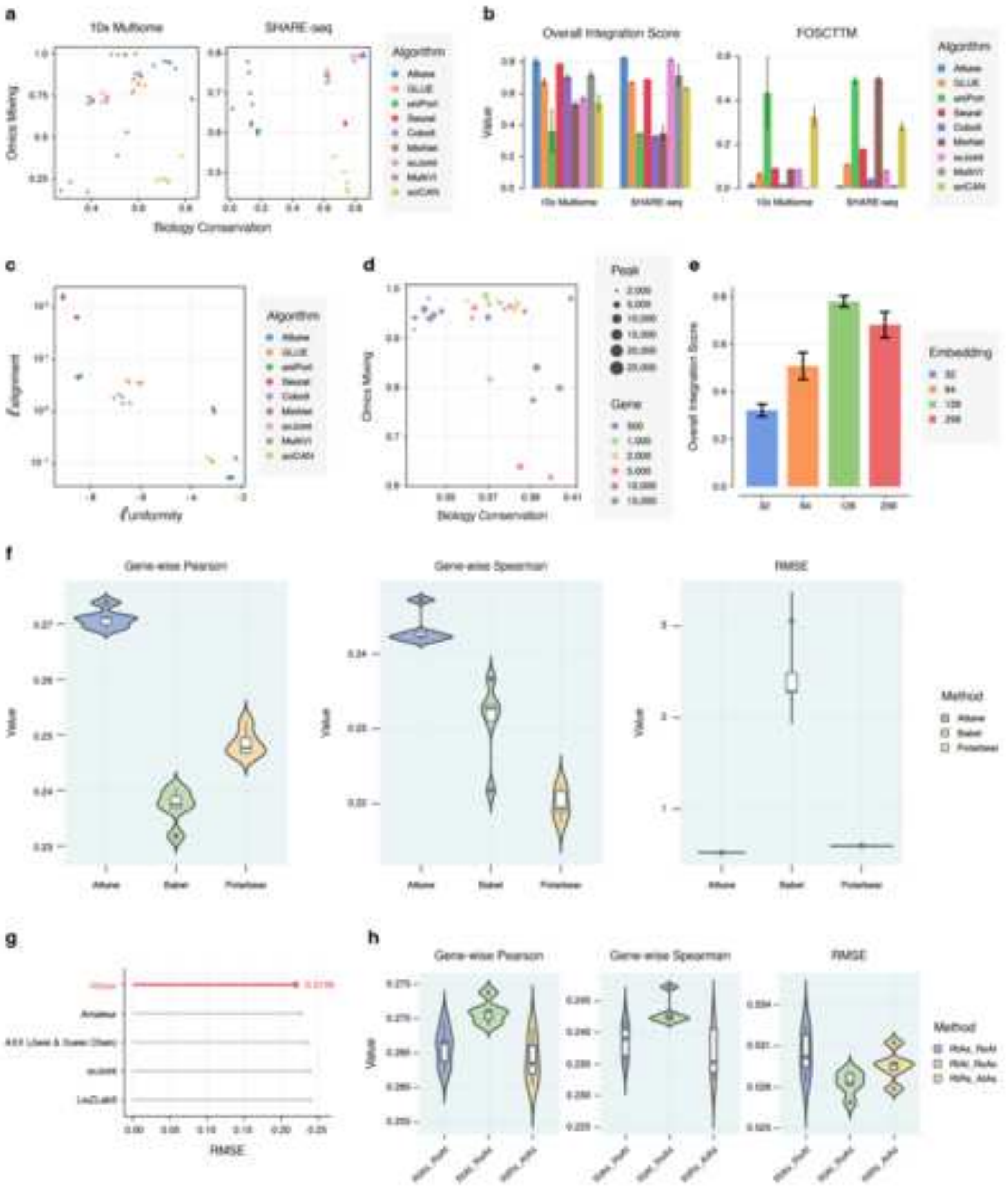

Figure3

Click here to access/download;Figure;Figure3.png

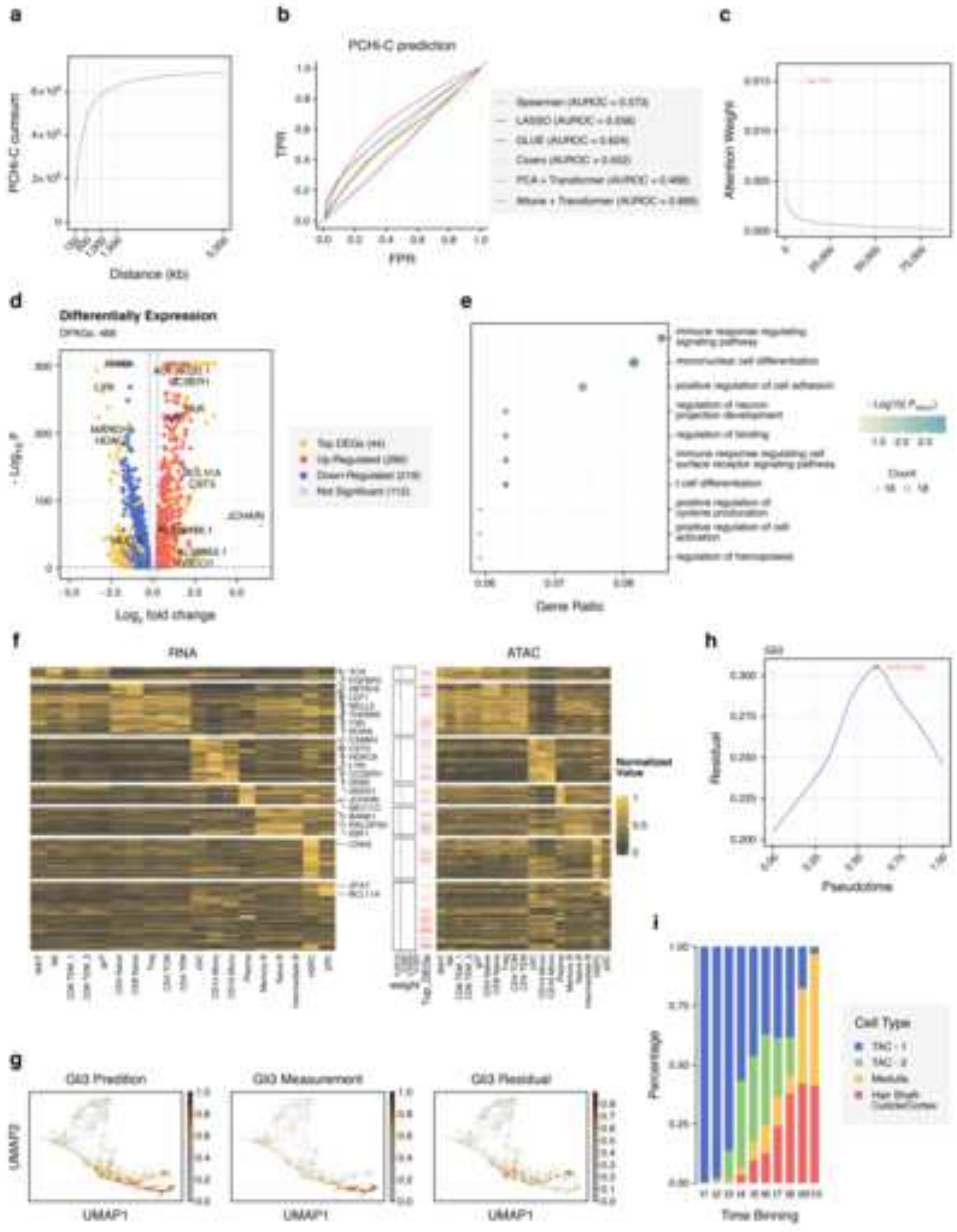

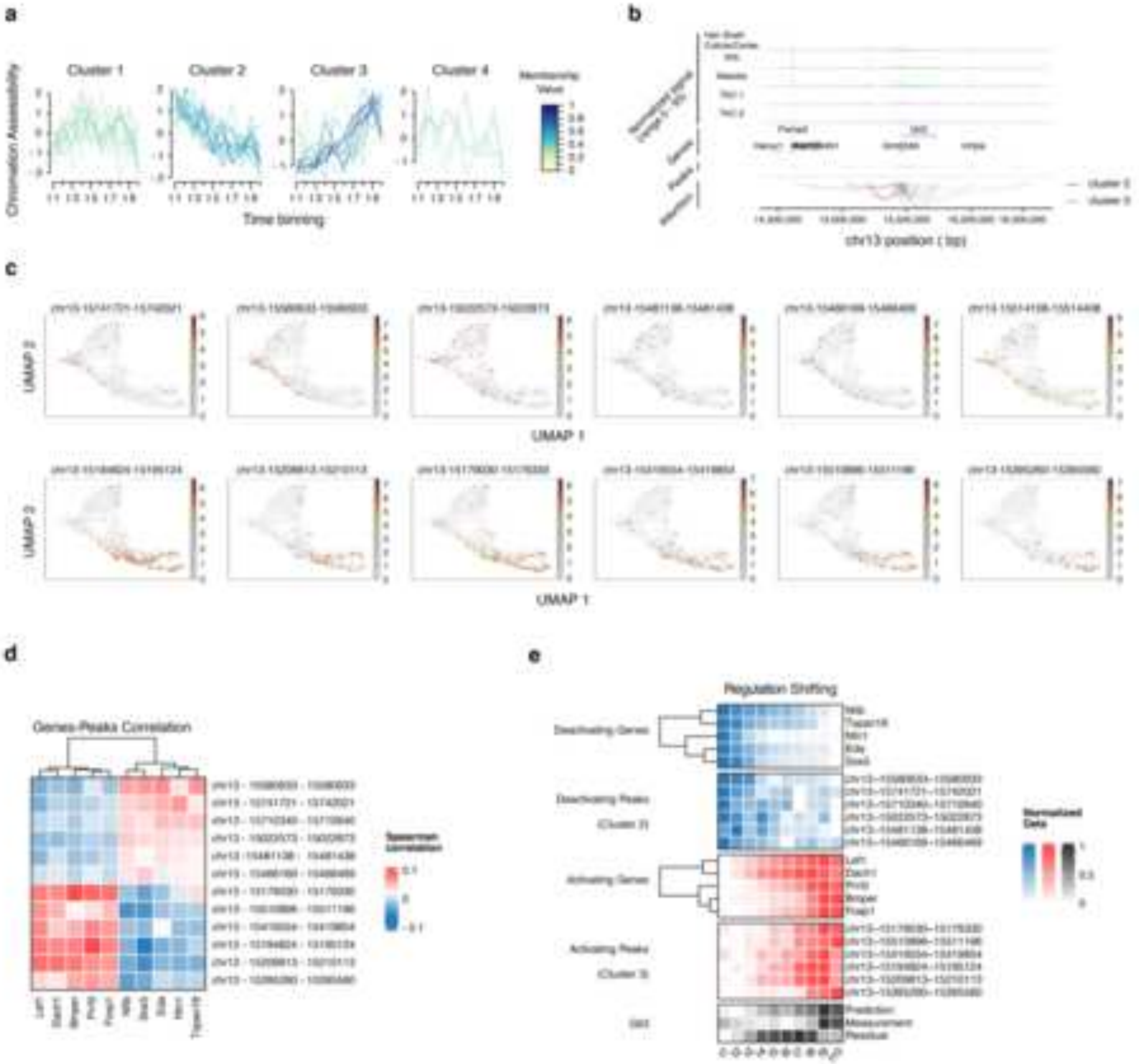
a
Cluster 1
Cluster 2
Cluster 3
Cluster 4
Chromatin Accessibility
Time binning
b
chr13 position (bp)
c
UMAP 2
UMAP 1
d
Genes-Peaks Correlation
e
Regulation Shifting
Deactivating Genes
Deactivating Peaks
Activating Genes
Activating Peaks
Prediction
Measurement
Residual

Figure5

Click here to access/download;Figure;Figure5_new.png

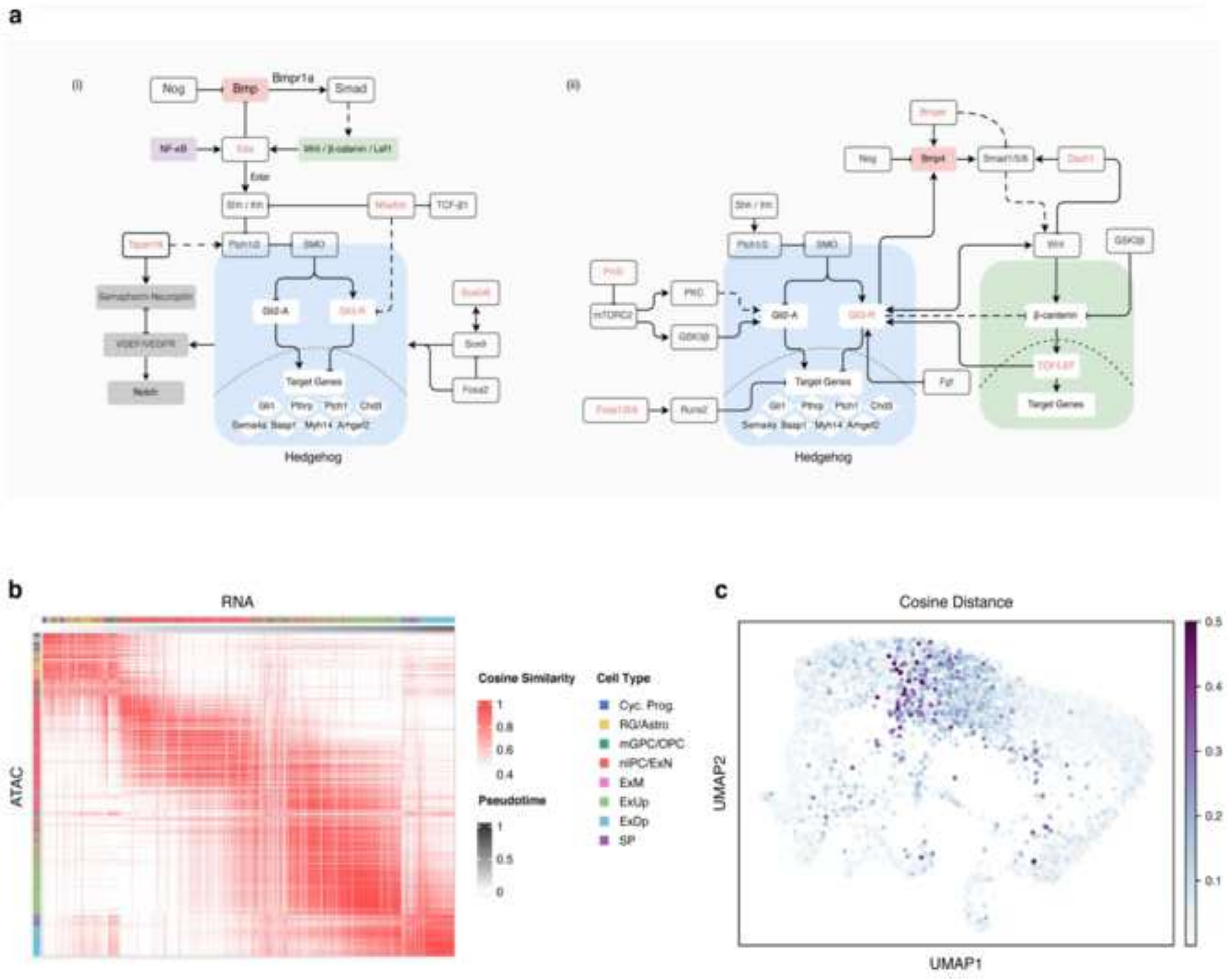

Click here to access/download
Supplementary Material
Supplementary_File.pdf

Supplementary Table

Click here to access/download
Supplementary Material
Supplementary_Table.xlsx

Personal Cover

Click here to access/download;Personal Cover;cover_letter_240522.docx

Dear Editor:

We are delighted to submit our manuscript, entitled "Cross-modal contrastive learning discovers chromatin potential regulating gene expression of single cell atlas" along with Supplementary Files containing 8 Supplementary Figures and 14 Supplementary Tables. We believe that our work has great potential as a candidate for publication in your esteemed journal.

Our manuscript introduces a cutting-edge computational framework called Attune, which utilizes cross-modal contrastive learning to advance the field of multi-modal single-cell data modeling and regulatory network discovery.

Our key innovations include as below,

1. Attune is a self-supervised learning framework that aligns paired gene expression and accessibility information through multi-view contrastive operations. It is fully data-driven, without requiring any prior knowledge or labels. Our framework's multi-view teacher-student configuration balances the advantages of complex and simple networks, capturing semantically rich features while maintaining generalizability. This configuration is well-suited for single-cell data analysis since each cell is unique, yet similarities are also shared between cells.

2. Attune's cell embeddings comprise information from different measurements, which can be flexibly adapted to various downstream tasks via pre-training and fine-tuning. Our framework outperforms competing methods in multiple tasks, such as data integration, cross-modal prediction, and cis-regulation discovery.

3. Moreover, Attune presents a novel strategy for inferring regulation in multi-modal single-cell data by implementing an attention mechanism. Current methods for regulation inference can be classified into four categories depending on the approach to deducing regulation: correlation (DORCs), co-expression (SCENIC+), distance (GLUE), and topic model (MIRA). The attention operation equips Attune with robust interpretability, effectively addressing the limitations of current deep learning methods in single-cell analysis. Leveraging cross-attention, Attune uncovers the cis-regulatory elements of genes and cell-type-specific factors that determine the cellular state.

4. We have also observed that certain cells' different modalities do not correspond, particularly cells in the differentiation process. Attune can capture this inconsistency and uncover chromatin potential along lineage priming. With the cross-attention mechanism, Attune unveils connections between peaks and genes and reconstructs a regulatory network that confirms the biological mechanism of chromatin potential. In the context of the mouse hair follicle dataset, Attune portrays a multi-pathway developmental landscape, beginning with the time delay of a dual-form transcription factor Gli3. Furthermore, by leveraging fine-tuned embeddings, Attune depicts the transition states between neonatal

and maturing neurons in the human cortex.

Our team specializes in integrating AI techniques into single-cell analysis, evidenced by our established publication record. We have recently contributed to Nature Machine Intelligence (doi.org/10.1038/s42256-022-00518-z) and iScience (10.1016/j.isci.2024.109635). These publications highlight our innovative approaches using contrastive learning for single-cell analysis and applying deep learning to investigate RNA velocity in single cells. We believe that our manuscript fits perfectly with the scope of your journal and adds significant value to the recent surge in using AI in multi-modal single-cell data discovery.

We eagerly look forward to your response and the opportunity to share our findings with your readership.

Best wishes,

Yang Meng
On behalf of all authors
MGI, BGI-Shenzhen
