# Supplementary material for: Cross-modal contrastive learning decodes developmental regulatory features through chromatin potential analysis
Source: Gigascience. 2025 Oct 17;14:giaf053. doi: 10.1093/gigascience/giaf053 (PMC12532322; doi:10.1093/gigascience/giaf053)
Supplement: giaf053_GIGA-D-24-00345_Revision_1 [file giaf053_giga-d-24-00345_revision_1.pdf]

GigaScience

Cross-modal contrastive learning discovers chromatin potential regulating gene expression of single cell atlas

--Manuscript Draft--

**Manuscript Number:**	GIGA-D-24-00345R1
**Full Title:**	Cross-modal contrastive learning discovers chromatin potential regulating gene expression of single cell atlas
**Article Type:**	Research
**Funding Information:**	
**Abstract:**	Emerging large-scale multimodal single-cell data jointly measures chromatin accessibility and transcription in the same cell, thus reconciling matched data paves integrated route for comprehensive regulatory analysis. Here, we introduce Attune, a cross-modal contrastive learning framework to align paired gene expression and accessibility information. Systematic benchmarking shows Attune's superior performance for omics integration and gene expression prediction. We further introduce Transformer-based cross-modal attention over fine-tuned gene and peak embeddings to infer regulatory interaction and discover significant differential signals of cell subtypes. Applied to hair follicle maturation dataset, Attune reveals chromatin potential for bifunctional transcription factor Gli3 at the gene level. In addition, the paired representations determine transmitted states across neonatal and mature cell types of cortical neuron differentiation at the cell level. Taken together, Attune features a promising paradigm for regulatory inference across omics layers and allows for extending more complex omics analysis.
**Corresponding Author:**	MENG YANG<br>BGI-Shenzhen: BGI Group<br>Shenzhen, CHINA
**Corresponding Author Secondary Information:**	
**Corresponding Author's Institution:**	BGI-Shenzhen: BGI Group
**Corresponding Author's Secondary Institution:**	
**First Author:**	Yueyuxiao Yang
**First Author Secondary Information:**	
**Order of Authors:**	Yueyuxiao Yang
	Chenxi Xie
	Qiushun He
	MENG YANG
**Order of Authors Secondary Information:**	
**Response to Reviewers:**	We have included the figures, tables, and formulas in a separate document titled "Response.docx" (attached to the Personal cover) to address the reviewer's comments. The reason for using a separate file is that the website's input box does not support the inclusion of figures. By providing a standalone document, we aim to make it easier for the reviewer to access and evaluate our responses. While we have conducted several additional experiments based on the reviewer's suggestions, we are unsure if they fully meet the intended requirements. Therefore, we have refrained from making extensive changes to the original manuscript to avoid potential confusion if further revisions are necessary. Should we receive additional feedback, we are prepared to integrate and update the results accordingly to ensure the manuscript's quality.

Powered by Editorial Manager® and ProduXion Manager® from Aries Systems Corporation

We sincerely appreciate the time and effort the reviewers have dedicated to reviewing our manuscript titled “”. We acknowledge the significance of addressing your concerns to ensure the clarity and comprehensibility of our work.

We are grateful for the valuable insights provided by Reviewer #1 in the feedback. And we will provide detailed explanations and clarifications for the raised comments, as we strive to address them effectively.

Reviewer#1 comments 1. As for model architecture presented in Figure 1b, the author included a one-way translation from ATAC counts to RNA counts. MSE loss was applied to minimize the reconstruction of translation from ATAC to RNA. What is the rationale behind such one-way translation? Why not translation from RNA counts to ATAC counts, or explicitly having bidirectional translation between these two modalities?

Response to Reviewer#1 comments 1:

Our approach was inspired by the multimodal single-cell data integration task presented at the NeurIPS 2021 competition, specifically Task 1: Modality Prediction [1]. This task focuses on predicting one modality from another, with performance evaluated using root mean squared error (RMSE). The biological rationale underlying this task is that genetic information flows from DNA to RNA to proteins. Chromatin accessibility (ATAC data) governs gene expression (RNA data), which in turn drives protein synthesis. By aligning with this biological hierarchy, our focus was to predict RNA (GEX) data from ATAC data, reflecting how chromatin accessibility regulates transcription.
In the context of modality prediction, previous works such as BABEL [2], MultiVI [3], and Polarbear [4] have demonstrated the feasibility of both RNA-to-ATAC and ATAC-to-RNA predictions. Building on this foundation, we chose to prioritize the ATAC-to-RNA direction for its biological relevance. However, to extend the model’s capability, we incorporated a reverse flow (RNA to ATAC) into Attune’s cross-modal prediction task (see Response Figure 1). We then benchmarked Attune against nine state-of-the-art algorithms, including LS_Lab, MultiVI, scVAEIT, LIGER, Seurat, BABEL, scMOG, scMoGNN, and CMAE, as described in a recent study on single-cell multi-omics prediction and integration [5].
To evaluate Attune, we utilized 11 single-cell RNA + ATAC datasets from a range of sequencing platforms, including SNARE-seq, SHARE-seq, ISSAAC-seq, 10x Multiome, and DOGMA-seq. We tested the model under two scenarios: intra-dataset (training and testing on the same dataset, see Response Figures 2A-2C) and inter-dataset (training and testing on different datasets, see Response Figures 2D-2F). Evaluation metrics included cell-cell PCC, peak-peak PCC, cell-cell CMD, peak-peak CMD, RMSE, AUROC, and Ranking Index (RI), providing a comprehensive assessment of prediction accuracy and generalizability. Specifically:
RMSE quantifies the deviation between predicted and actual values.
CMD measures differences in correlation matrices, indicating how well the model captures relationships between features.
PCC assesses chromatin accessibility abundance correlation.
AUROC evaluates the effectiveness of predicting chromatin accessibility.
RI summarizes overall algorithm performance.
A lower CMD and RMSE indicate better performance, while higher PCC and AUROC suggest more accurate predictions.
In the intra-dataset scenario, although Attune did not outperform LS_Lab or scVAEIT in all metrics, it achieved results that were consistently above or near the median across most benchmarks. Overall, Attune ranked in the second tier among the algorithms, comparable to MultiVI and slightly outperforming Seurat (see Response Figure 3). In the inter-dataset scenario, Attune demonstrated strong generalizability alongside LS_Lab. Both models achieved cell-cell PCC, peak-peak PCC, and AUROC values above the median, while their CMD and RMSE values were below the median, indicating robust cross-dataset performance. As a result, Attune ranked in the top tier, comparable to LS_Lab (see Response Figure 4). When considering performance across both intra- and inter-dataset scenarios (see Response Figure 5), Attune exhibited robustness and adaptability. While LS_Lab achieved the best overall

Powered by Editorial Manager® and ProduXion Manager® from Aries Systems Corporation

performance, Attune was comparable to MultiVI and scVAEIT, highlighting its effectiveness and versatility.
The datasets and evaluation pipeline used in this study are consistent with the benchmarking article by Hu et al. [5]. The datasets were obtained from https://mailustceducn-my.sharepoint.com/:f:/g/personal/hyl2016_mail_ustc_edu_cn/EgYFP7tlTKBBuAhkdtrIOg4B1Eyo-_iBx1VKBWSK0r-9rA?e=gmhocx, and the evaluation pipeline was implemented as described in the benchmarking repository https://github.com/QuKunLab/MultiomeBenchmarking/blob/main/code/Prediction/RNA_ATAC/Matrics.py.
We hope this explanation clarifies the rationale for our design and the comprehensive steps we took to evaluate Attune.

[1] Luecken, M.D., Burkhardt, D.B., Cannoodt, R., Lance, C., Agrawal, A., Aliee, H., Chen, A.T., Deconinck, L., Detweiler, A.M., and Granados, A.A. A sandbox for prediction and integration of dna, rna, and proteins in single cells. 2021.
[2] Wu K E, Yost K E, Chang H Y, et al. BABEL enables cross-modality translation between multiomic profiles at single-cell resolution[J]. Proceedings of the National Academy of Sciences, 2021, 118(15): e2023070118.
[3] Ashuach T, Gabitto M I, Koodli R V, et al. MultiVI: deep generative model for the integration of multimodal data[J]. Nature Methods, 2023, 20(8): 1222-1231.
[4] Zhang R, Meng-Papaxanthos L, Vert J, et al. Multimodal single-cell translation and alignment with semi-supervised learning[J]. Journal of Computational Biology, 2022, 29(11): 1198-1212.
[5] Hu Y, Wan S, Luo Y, et al. Benchmarking algorithms for single-cell multi-omics prediction and integration[J]. Nature Methods, 2024: 1-13.

Reviewer#1 comments 2. The gene-peak cross-attention seems to be a very big computation, not to mention the computation needed for the peak-peak self-attention (It is not clear to me if peak-peak self-attention is included in the model. If not, why peak-peak attention is unnecessary?). It is unclear what kind of computational power is enough to do the transformer fine-tuning. Meanwhile, it is also not clear why the author didn't combine transformer fine-tuning with pretraining as one end-to-end training? What are the benefits of dividing the model into two parts?

Response to Reviewer#1 comments 2:

Thank you for your insightful comments. We greatly appreciate the opportunity to further clarify the design and computational approach of our model.
Our methodology consists of two main components: pre-training the Attune model and performing downstream tasks. During the pre-training phase, the expression matrices of both ATAC-seq and RNA-seq data are input into the pre-training component. This phase employs unsupervised cross-modal contrastive learning, which is critical for setting up the model for subsequent tasks. The goal is to establish shared high-dimensional representations for cells across the two modalities (ATAC and RNA).
For tasks involving gene-peak interactions, the model generates embedding matrices for genes and peaks (with dimensions ×× for genes and ×× for peaks, where represents the number of cells, represents the number of genes, represents the number of peaks, and is the embedding dimension). These matrices are then passed through a Transformer model that uses self-attention and cross-attention mechanisms to derive a global cross-attention weight matrix (×), which captures the gene-peak relationships. The self-attention mechanism operates on the gene embedding matrices (××) to model interactions between genes. The cross-attention mechanism operates between the peak embedding matrices (××) and the output of the gene self-attention mechanism (××) to capture the interaction between genes and peaks.
(1) Why not perform self-attention on peaks?
Due to hardware limitations (we are using a Quadro RTX 6000 with 24GB of memory), we are unable to perform self-attention on the peak embedding matrices. The number of peaks in typical datasets ranges from tens of thousands to hundreds of thousands, and the corresponding embedding matrices are typically very sparse. Performing self-attention on such sparse matrices would be computationally expensive and inefficient. Our testing has shown that performing self-attention on genes (with approximately 2,000 genes) and cross-attention between genes and peaks (with around 28,708 peaks) consumes approximately 24GB of memory on an RTX 6000 (batch size = 2)

and 76GB on an A100 (batch size = 12). These memory requirements indicate that, with our current hardware setup, performing self-attention on peaks is infeasible due to their large number and sparsity.
In our cross-attention mechanism, we use the gene embedding matrices, after self-attention, as the Query and directly use the peak embedding matrices as the Key and Value. When investigating regulatory interactions between genes, we aim to identify which peaks regulate each gene. It is therefore reasonable to use the gene embeddings as the Query, as they already capture associations between genes. Each gene in the Query computes the similarity (via vector dot product) with each peak in the Key. If a gene is highly correlated with a peak, the corresponding peak in the Value will be selected. This peak is then weighted by the similarity score, and the gene embedding matrix is updated accordingly, based on the relationship between the gene and the peak.
(2) Why not combine pre-training and fine-tuning into a single end-to-end process?
The decision to separate pre-training and fine-tuning is a deliberate design choice to address computational constraints while ensuring effective model training. By pre-training Attune using cross-modal contrastive learning, we ensure that the gene and peak representations share a high-dimensional latent space. During the downstream task, we freeze the weights of the Attune model and only fine-tune the Transformer. This separation prevents the contrastive learning process from disrupting the learned gene-peak associations during fine-tuning, thus ensuring stability in the shared space and improving the accuracy of downstream predictions. If we were to combine pre-training and fine-tuning into a single end-to-end training process, the backpropagation through both Attune and the Transformer might cause the learned gene-peak associations to shift, leading to potential instability. The contrastive learning process in Attune would update the distances between cell representations in the shared latent space, which could inadvertently affect the pre-established gene-peak associations. By separating these stages, we maintain a more stable learning process for the downstream task.
In summary, the decision to exclude self-attention on peaks and to split the pre-training and fine-tuning stages was driven by both computational constraints and the need for a stable and efficient learning process. We hope this explanation clarifies the rationale behind our model architecture and design choices.

Reviewer#1 comments 3. It is not clear how benchmarking was done. Both 10X multiome and SHARE-seq are joint profiling data. It is necessary for the author to provide more details about how other methods were run under author's hands, especially how the author handled data preprocessing for each method. Meanwhile, each cell has profiles of two modalities. Attune clearly takes advantage of knowing such correspondence. However, other methods primarily use shared features for integration, which will not take a full advantage of joint profiling. Seurat V3 was included in benchmarking. This version of Seurat also uses shared features. The author will need to consider adding Seurat V5 in benchmarking, which uses information of joint profiling for integration. Another existing contrastive learning SMILE was also proposed to integrate RNA-seq and ATAC-seq with joint-profiling data. Meanwhile, the author previously developed another contrastive learning integration method Concerto. It should also be included in benchmarking. The author may need to include more clear justifications on how Attune is improved from their previous work Concerto.

Response to Reviewer#1 comments 3:

Thank you for your insightful comments regarding the benchmarking methodology and the comparison with existing methods. We agree that it is important to clarify the distinction between multimodal integration and cross-modal alignment, as these are fundamentally different tasks with distinct objectives.
(1)Multimodal Integration: In our approach, Attune is designed for multimodal integration, where the goal is to combine data from paired modalities into a shared feature space. The model takes joint profiling data (where each cell contains profiles of both RNA and ATAC modalities) and learns a joint embedding that represents both modalities together. This approach aligns with the concept of joint embedding as outlined in the NIPS 2021 competition [1], which focuses on integrating features from different modalities into a unified representation. Attune leverages the correspondence between modalities within paired cells to optimize the integration, ensuring that both modalities are represented in a coherent and shared space.

(2)Cross-modal Alignment: In contrast, cross-modal alignment methods aim to establish correspondences between unpaired cells from different modalities. Historically, most single-cell methods were designed to analyze one modality at a time, but recent advances have introduced methods for measuring multiple modalities within the same cells. Methods such as GLUE, uniPort, Seurat V3, Cobolt, MinNet, scJoint, MultiVI, and sciCAN focus on aligning unpaired cells by mapping data from different modalities into a shared space using similarity metrics. These methods can also be applied to joint profiling data, but they do not fully exploit the pairing information. For example, scJoint is designed for unpaired data, but it can still be used with paired data, treating the RNA and ATAC components as separate datasets [2]. Similarly, GLUE has been benchmarked against unpaired methods like MMD-MA, LIGER, and Seurat V3 using paired data such as 10X Multiome and SHARE-seq [3].

Data Processing. In our benchmarking experiments, we followed the standard preprocessing procedures for multimodal data integration. Although the data is joint profiling, we treated the RNA and ATAC parts as separate datasets for the purpose of comparison with other algorithms. For scRNA-seq data, we removed genes expressed in fewer than 5% of cells and normalized the counts to 10,000 per cell using SCANPY. Additionally, sex chromosome genes were excluded, and the top 2000 highly variable genes (HVGs) were selected. For scATAC-seq data, we filtered out peaks detected in fewer than 5% of cells and removed sex chromosome peaks. These preprocessing steps ensured that the datasets were comparable while balancing computational efficiency and model performance. Methods like MultiVI and GLUE do not require the same number of features between modalities and can directly handle RNA and ATAC data. However, methods such as scJoint and sciCAN require common features between the modalities, so we first identified overlapping genes between RNA and ATAC datasets before applying the algorithms. After training, we computed integration metrics such as neighbor consistency, Seurat alignment score, and Fraction of Samples Closer Than the True Match (FOSCTTM).

Benchmarking with Multimodal Integration Methods. The key advantage of Attune lies in its ability to explicitly use the pairing information between modalities. This allows the model to treat the two modalities of the same cell as positive pairs, which results in a more precise alignment. Through contrastive learning, the model brings RNA and ATAC data closer in a high-dimensional space, yielding better performance in metrics like FOSCTTM compared to methods that rely solely on shared features. Unlike other methods that aim to create a single unified representation, Attune aligns RNA and ATAC data in a shared feature space while maintaining the identity of each modality, allowing for more accurate comparisons and analyses of the interactions between the modalities.

Concerto [4] supports multimodal integration. It is a simple element-wise summation of the output of the teacher network or student network for each modality (the embedding of cell in each modality). The contrastive loss (NT-Xent loss [5]) is calculated on the summed cell embeddings (from teacher network and student network, respectively). Concert can generate unified cell embeddings. In the case of two modalities (RNA and ATAC), we illustrate the corresponding operation according to equation (1) and (2) and (3), where the term z_teacher^RNA∈R^d denotes cell embedding of RNA output by teacher network and z_teacher^ATAC∈R^d denotes cell embedding of ATAC output by teacher network and z_student^RNA∈R^d denotes cell embedding of RNA output by student network and z_student^ATAC∈R^d denotes cell embedding of ATAC output by student network. Add denotes add along the dimension of embedding and Contrastive denotes NT-Xent loss and d denotes the dimension of cell embeddings.

The improvement of Attune over Concerto is that it does not need to sum the cell embeddings of the two modalities before calculating the contrastive loss, but directly calculates the contrastive loss for the cell embeddings of each modality (from the teacher network and the student network respectively). The above calculation process in shown in equation (4) and (5) and (6).

When using UMAP to visualize cell embeddings, Concerto performs UMAP on z_teacher^multi to obtain unified cell embeddings （N×d） that combines the two modalities (RNA and ATAC), while Attune concatenates z_teacher^RNA and z_teacher^ATAC to get joint cell embeddings （2N×d）, which maps the cell embeddings of two modalities onto the same two-dimensional space. N represents the number of cells.

In response to the reviewer’s suggestion, we have included SMILE [6], Seurat V5 [7], and Concerto in the benchmarking process. These methods use joint profiling data for integration and are compared with Attune using several metrics: mean average

Powered by Editorial Manager® and ProduXion Manager® from Aries Systems Corporation

precision (MAP), cell type adjusted silhouette width (ASW), neighbor consistency (NC), Seurat alignment score (SAS), batch adjusted silhouette width (Batch ASW), graph connectivity (GC), biology conservation, omics mixing, overall integration score, and FOSCTTM (see Methods for details). Since Seurat V5 maps scATAC-seq datasets onto scRNA-seq datasets when integrating different modalities, it only outputs cell representations of single modality and Concerto generates unified cell embeddings. Therefore, it is impossible to calculate the metrics that require the cell representation of each modality, such as Batch ASW, NC, SAS and FOSCTTM.
In the 10X Multiome dataset (Response Table 1), SMILE outperforms Attune in GC, but Attune shows superior results in most other metrics. Seurat V5 excels in MAP and cell type ASW but is outperformed by Attune in GC. Concerto performs similarly to Attune in MAP but lags behind in terms of robustness in cell type ASW and GC. UMAP visualizations for SMILE, Seurat V5, and Concerto are shown in Response Figures 6-9.
In the SHARE-seq dataset (Response Table 2), SMILE surpasses Attune in Batch ASW and GC but falls short in MAP and cell type ASW. Therefore, it is close to Attune in Omics mixing, but inferior to Attune in Biology conservation. Seurat V5 and Concerto also perform worse than Attune in MAP, cell type ASW, and GC. The UMAP visualizations for SMILE, Seurat V5, and Concerto are shown in Response Figures 10-13. These results were repeated five times with different random seeds to ensure robustness.

Response Table 1. Metrics for evaluating integration performance in 10X Multiome dataset
foldalgorithmmapBatch ASWCell type ASWGCNCSASFOSCTTMBiology conservationOmics mixingOverall score
1SMILE0.6950.8580.5150.9890.3660.5890.0080.5250.8120.640
2SMILE0.6920.8490.5200.9870.3670.5810.0070.5260.8060.638
3SMILE0.6880.8550.5100.9800.3610.5820.0080.5200.8060.634
4SMILE0.6890.8500.5130.9850.3650.5800.0070.5220.8050.635
5SMILE0.6910.8520.5150.9810.3650.5840.0080.5240.8060.636
1Attune0.7160.9510.5400.9340.2110.9150.0180.6640.9290.770
2Attune0.7290.9540.5450.9370.2120.9750.0230.7080.9550.807
3Attune0.7400.9430.5520.9280.2130.8920.0120.7560.9110.818
4Attune0.7300.9530.5450.9350.2170.9660.0250.7250.9500.815
5Attune0.7330.9580.5450.9350.2180.9470.0110.7340.9470.819
1Seurat V50.8110.5500.854
2Seurat V50.8090.5480.850
3Seurat V50.8100.5490.852
4Seurat V50.8100.5500.853
5Seurat V50.8090.5490.851
1Concerto0.7420.5220.905
2Concerto0.7340.5120.893
3Concerto0.7310.5110.892
4Concerto0.7250.5100.883
5Concerto0.7210.5070.881

Response Table 2. Metrics for evaluating integration performance in 10X SHARE-seq dataset
foldalgorithmmapBatch ASWCell type ASWGCNCSASFOSCTTMBiology conservationOmics mixingOverall score
1SMILE0.6300.9150.5100.8810.3890.5620.0150.5100.7860.620
2SMILE0.6310.9200.5110.8800.3880.5600.0140.510.7870.621
3SMILE0.6290.9160.5090.8820.3900.5610.0150.5090.7860.620
4SMILE0.6300.9150.5120.8810.3900.5630.0140.5110.7860.621
5SMILE0.6290.9140.5090.8790.3880.5600.0150.5090.7840.619
1Attune0.7010.8130.5400.8510.1070.8100.0120.8470.7970.827
2Attune0.7020.8130.5400.8580.1070.8090.0110.8490.7990.829
3Attune0.7030.8120.5410.8530.1070.8070.0110.8510.7950.828
4Attune0.7040.8110.5410.8530.1070.8060.0100.8530.7930.829
5Attune0.7050.8100.5420.8460.1070.8130.0100.8550.7920.830
1Seurat V50.6020.4830.570
2Seurat V50.6020.4840.572
3Seurat V50.6030.4820.572
4Seurat V50.6010.4810.571

Powered by Editorial Manager® and ProduXion Manager® from Aries Systems Corporation

5Seurat V50.6020.4820.573
1Concerto0.4670.4970.703
2Concerto0.4470.4940.684
3Concerto0.4830.4990.714
4Concerto0.4970.5020.733
5Concerto0.4370.4910.660

[1] Luecken, M.D., Burkhardt, D.B., Cannoodt, R., Lance, C., Agrawal, A., Aliee, H., Chen, A.T., Deconinck, L., Detweiler, A.M., and Granados, A.A. A sandbox for prediction and integration of dna, rna, and proteins in single cells. 2021.
[2] Lin Y, Wu T Y, Wan S, et al. scJoint integrates atlas-scale single-cell RNA-seq and ATAC-seq data with transfer learning[J]. Nature biotechnology, 2022, 40(5): 703-710.
[3] Cao Z J, Gao G. Multi-omics single-cell data integration and regulatory inference with graph-linked embedding[J]. Nature Biotechnology, 2022, 40(10): 1458-1466.
[4] Yang M, Yang Y, Xie C, et al. Contrastive learning enables rapid mapping to multimodal single-cell atlas of multimillion scale[J]. Nature Machine Intelligence, 2022, 4(8): 696-709.
[5] Chen T, Kornblith S, Norouzi M, et al. A simple framework for contrastive learning of visual representations[C]//International conference on machine learning. PMLR, 2020: 1597-1607.
[6] Xu Y, Das P, McCord R P. SMILE: mutual information learning for integration of single-cell omics data[J]. Bioinformatics, 2022, 38(2): 476-486.
[7] Hao Y, Stuart T, Kowalski M H, et al. Dictionary learning for integrative, multimodal and scalable single-cell analysis[J]. Nature biotechnology, 2024, 42(2): 293-304.

Reviewer#1 comments 4. The author included a PCA + Transformer as one of comparisons in the task of regulatory prediction. It is questionable if PCA is a proper way to learn the peak embedding. Second, only the top 10 PCs don't justify if all major variations are captured in 10 PCs. I am interested in knowing if NMF-based approach would perform much better than PCA. For example, the author can combine the cisTopic and Transformer.

Response to Reviewer#1 comments 4:

Thank you for your insightful feedback and thoughtful suggestions. We greatly appreciate your comments regarding the use of PCA for learning peak embeddings and the potential advantages of alternative methods such as NMF.
In response to your concern, we performed additional experiments to evaluate the performance of NMF-based methods. Specifically, we compared PCA with 10 components to NMF with both 10 and 20 components. However, as shown in Response Figure 14, the results did not demonstrate a significant improvement over the PCA approach. This suggests that, while NMF may have some potential advantages, the performance gain over PCA was not particularly substantial in our study. We acknowledge that further exploration, including experimentation with different configurations and larger datasets, could provide a more comprehensive assessment of NMF's capabilities in this context.
Regarding cisTopic, we recognize that it is primarily designed for scATAC-seq data and may not be directly applicable to scRNA-seq. In our study, we opted to use PCA as the dimensionality reduction technique for both scRNA-seq and scATAC-seq data to maintain consistency across the two modalities. However, we are uncertain whether the reviewer would prefer us to apply PCA specifically to scRNA-seq and use cisTopic for the scATAC-seq data. If this alternative approach is preferred, we are happy to explore it further and present the corresponding results.
We hope this clarifies our approach and addresses your concerns. Should you require additional information or further analysis, we are more than willing to provide it. Thank you once again for your valuable input.

Reviewer#1 comments 5. It is surprising that all methods perform poorly in predicting distal interactions (Figure 3b). Even though the author showed that Attune outperforms other existing methods, the prediction results in Figure 3b doesn't justify that Attune can accurately identify distal promoter interactions (The false positive rate is so high). The author needs a clear explanation on such issue and adds additional benchmark data for this task.

Powered by Editorial Manager® and ProduXion Manager® from Aries Systems Corporation

Response to Reviewer#1 comments 5:

Thank you for your insightful comments regarding the performance of our model and the challenges associated with predicting distal promoter interactions. We agree that Figure 3b highlights notable limitations, particularly in accurately identifying distal interactions, and we provide additional context and detailed interpretations below.
The importance of distal interactions in benchmarking. Distal promoter interactions are biologically significant and essential to understanding gene regulation, even though they are challenging to predict. In our study, we included interactions up to 1.2 Mbps based on the statistical distribution of interaction distances in the dataset from Javierre et al. (2016) [1]. Studies such as Laverré et al. (2022) further emphasize the critical role of long-range interactions in gene expression and chromatin organization [2]. However, most existing methods primarily focus on short-range interactions (e.g., GLUE ≤150 kb, Cicero ≤500 kb). By intentionally including distal interactions in our benchmarks, we aim to highlight their biological importance and encourage improvements in predictive models for these relationships. Ignoring distal interactions could risk overlooking key regulatory mechanisms and limit the development of predictive methods for studying long-range regulation.
Performance trends and challenges of distal predictions. From the PCHi-C dataset results, we observe that true regulatory interactions decrease in frequency as distance increases (see Supplementary Figure 4a), which likely reflects a combination of biological and dataset-specific constraints. Nonetheless, most methods demonstrate higher differentiation capacity for short-range interactions (e.g., 25-50 kb and 50-75 kb bins), as shown in Response Figure 15. The boxplots illustrate that differentiation between true positive (TP) and true negative (TN) interactions diminishes at longer distances for most methods, reflecting the added complexity of long-range regulation. Nonetheless, Attune demonstrates relatively stronger differentiation capability in distal bins compared to other methods, indicating it may capture certain features of long-range regulation. Additionally, Calculating FDR uniformly across methods presents challenges due to the differing scoring systems used by each method (e.g., Spearman's correlation for association strength, Cicero's co-accessibility score for chromatin accessibility relationships). As these scores vary in scale and interpretation, defining fair and consistent thresholds across methods is challenging due to differing scoring systems. Instead of relying on thresholds, we visualized score distributions across distance bins to provide a more intuitive and method-agnostic comparison.
Inclusion of the eQTL v10 dataset. In response to your suggestion, we expanded our analysis to include the eQTL v10 dataset, which evaluates significant gene-peak pairs from whole blood samples. This dataset complements the PCHi-C data and allows us to assess Attune's generalizability across broader biological contexts. Since Attune was trained on PBMC data (primarily T cells, B cells, and monocytes), the inclusion of whole blood data helps evaluate its performance in a bulk context. However, Attune does not perform as well on the eQTL dataset, where GLUE achieves higher AUROC scores, likely due to its use of prior knowledge-based guidance graphs that incorporate additional regulatory context. For Attune, we hypothesize that incorporating such biological priors (e.g., enhancer-promoter interaction maps or tissue-specific chromatin features) might improve its performance in tasks involving distal interactions. Additionally, we hypothesize that Attune's predictions may be sensitive to cell-type composition, which could impact its robustness in datasets without cell-type annotations, such as eQTL v10. This remains an aspect requiring further investigation.
Our analysis underscores the importance of including distal interactions in benchmarking studies despite the challenges associated with their prediction. While these challenges are shared across all methods, our results show that Attune demonstrates stronger differentiation capacity in distal bins compared to other approaches. However, this study also highlights the need for future improvements, including integrating biological priors and leveraging single-cell datasets with detailed cell-type annotations to enhance robustness and performance.
We appreciate the reviewer's comments, which prompted us to expand our analysis and address the challenges of distal interaction prediction more comprehensively. If additional details or analyses are required, we would be happy to provide them. Thank you again for your thoughtful feedback.

[1] Javierre, Biola M., et al. "Lineage-specific genome architecture links enhancers and non-coding disease variants to target gene promoters." Cell 167.5 (2016): 1369-1384.
[2] Laverré, Alexandre, Eric Tannier, and Anamaria Necsulea. "Long-range

Powered by Editorial Manager® and ProduXion Manager® from Aries Systems Corporation

promoter–enhancer contacts are conserved during evolution and contribute to gene expression robustness." Genome Research 32.2 (2022): 280-296.

Reviewer#1 comments 6. It is not obvious that the chromatin regions presented in Figure 4c explain well the bifurcation of lineage commitment in mouse skin data. First, what are key peaks that drive the lineage commitment into the upper branch (IRS and TAC2). Second, these peaks seem to be not differentiable in the lower branch. Say, what drives the bifurcation into Medulla and Hair shaft-cuticle.

Response to Reviewer#1 comments 6:

Thank you for your insightful comments. To clarify the analysis presented in Figure 4c, our initial hypothesis was that Gli3 plays an important role in lineage commitment, based on the inconsistencies observed between two modalities. We then investigated the chromatin accessibility peaks associated with Gli3. Since differentiation is a time-dependent process, we performed pseudotime analysis to explore the relationship between peak accessibility and cell maturation. During this analysis, IRS cells were excluded because, as noted in both Ma’s study and our results, IRS consistently occupies an intermediate position along the pseudotime trajectory, whereas hair shaft-cuticle/cortex and medulla cells are found at the differentiation endpoint [1, 2]. Given that IRS, medulla, and hair shaft-cuticle/cortex represent distinct differentiation fates, we focused our pseudotime analysis on the outer sheath differentiation pattern and excluded IRS cells to avoid confounding the results. This decision was documented in the GitHub code provided with the manuscript. However, as you rightly pointed out, this rationale was not sufficiently explained in the main text, leading to potential misinterpretation.
The primary objective of this experiment was to investigate how Gli3 contributes to overall hair follicle development (on a broader scale) rather than its specific role in determining the bifurcation into medulla or hair shaft. At the current stage, our analysis does not resolve how individual Gli3-associated peaks might guide differentiation toward specific lineages. This limitation likely requires more intricate network analyses, integrating multi-gene and multi-pathway interactions, to comprehensively address the underlying mechanisms.
We will revise the manuscript to explicitly clarify these points and provide additional context to avoid similar ambiguities. If additional clarifications or analyses are needed, we are happy to provide further details. Thank you again for your thoughtful comments and for helping us improve the clarity and rigor of our work.

[1] Ma, Sai, et al. "Chromatin potential identified by shared single-cell profiling of RNA and chromatin." Cell 183.4 (2020): 1103-1116.
[2] Abe, Yoshinori, and Nobuyuki Tanaka. "Roles of the hedgehog signaling pathway in epidermal and hair follicle development, homeostasis, and cancer." Journal of developmental biology 5.4 (2017): 12.

Reviewer#1 comments 7. The author made a claim that "The overall upward shift of blocks indicates the lag of RNA modality", backed up by presentation of Figure 5c and Supplementary Figure 8c. These two figures are not straightforward to make such conclusion. It is suggested for the author to provide a more quantitative visualization or statistical examination to illustrate such transition.

Response to Reviewer#1 comments 7:

We greatly appreciate the reviewer’s insightful suggestion to enhance the quantitative rigor of our analysis supporting the claim that "the overall upward shift of blocks indicates the lag of RNA modality." In the original analysis, we used embedding techniques to explore the alignment between RNA and ATAC modalities, primarily to assess whether any cell types exhibit poor modality alignment. This misalignment could suggest a lag in RNA modality. Although the upward shift in the heatmap (Figure 5b), the clustering patterns in the UMAP (Figure 5c), and the distribution of cosine distances (Supplementary Figure 8c) indicate a potential RNA modality lag in certain cell types, we acknowledge that these visual representations alone may not provide sufficient statistical validation.
To address this, we conducted additional statistical analyses to provide a more robust quantitative comparison of modality mismatch across cell types. Specifically, we

calculated the distribution of cosine distances (1 - cosine similarity) for each cell type and visualized these results using a boxplot (Response Figure 17). Statistical comparisons using the Wilcoxon rank-sum test revealed significant differences in modality mismatch between nIPC/ExN and other cell types (p =1.68e-117), as well as between ExM and other cell types (p=7.85e-65). These findings quantitatively confirm that nIPC/ExN and ExM exhibit elevated modality mismatch compared to other cell types, thus directly supporting the claim of RNA modality lag in these regions.
We sincerely thank the reviewer for their valuable feedback, which has contributed significantly to improving the clarity and rigor of our analysis. Should further clarifications or additional analyses be required, we would be happy to provide them.

We sincerely thank Reviewer#2 for the thoughtful and constructive feedback on our manuscript. Your suggestions have been invaluable, and we have revised the manuscript to address your concerns. Below, we respond to each comment in detail.

Reviewer#2 comments 1. The notations and descriptions in the Methods section in the whole manuscript are quite confusing, making it difficult to follow. A lot of current presentations do not adhere to standard mathematical notation practices. For example:
a. x_indices and x_counts are defined multiple times with different dimensions.
b. In the line "outputs the weighted hidden vector h, h ∈ R (equation 2)." it is unclear how an observed gene expression variable like x_counts can "output" a hidden vector.
c. Equations 1-8 would benefit from clearer mathematical notations to denote gene embedding, gene hidden states, and use math notations to denote Embedding() as a function, ensuring clarity.
d. The meaning of certain operations, such as those in equations 2 and 4, is unclear—are these referring to element-wise multiplication or vector multiplications?
e. Additionally, variables such as m and m+ are defined only after they are first introduced, which disrupts the logical flow.
f. l() in 26 is not defined.
These are some of the examples. Completely rewriting of the whole method section is probably needed to improve clarity.

Response to Reviewer#2 comments 1:

We would like to sincerely thank Reviewer#2 for the detailed and constructive feedback. Your valuable comments have highlighted several areas that required attention, and we have thoroughly reviewed and revised the Methods section accordingly. Below, we respond to each of the specific points you raised:

On the multiple definitions of X_indices and X_counts with inconsistent dimensions: Our model uses two input modalities: single-cell ATAC-seq (scATAC-seq) and single-cell RNA-seq (scRNA-seq). For scRNA-seq, the data is organized into a matrix with 10,000 rows (representing individual cells) and 2,000 columns (representing genes). Each column corresponds to a unique gene index ranging from 1 to 2,000, where "gene 1" is in the first column with an index of 1. In the manuscript, “i" denotes the index of a gene within the matrix, and “G” represents the total number of genes, which in this case is 2,000. The matrix “X_indices” contains the gene indices and has dimensions of (N x G), where N is the number of cells and G is the number of genes. Each row of “X_indices” contains the sequence [1, 2, 3, ..., 2000]. “X_counts” refers to the gene expression matrix, which has the same dimensions as “X_indices” (N x G). We have now unified the definitions and descriptions of “X_indices” and “X_counts” for consistency and clarity.

b-d.

Modify “Teacher network” section in Methods.

For two kinds of single-cell data (scRNA-seq and scATAC-seq), we designed two teacher networks (RNA teacher network and ATAC teacher network) to learn fine-grained representations respectively. The RNA teacher network accepts X_indices R^G and X_counts R^G as input, where G denotes the number of genes. X_indices represents gene indices, which dimension is (N×G) and X_counts represents the value of gene expression, which dimension is (N×G). N is the number of cells. For ATAC

Powered by Editorial Manager® and ProduXion Manager® from Aries Systems Corporation

teacher network accepts input of Y_indices R^P and Y_counts R^P, where P denotes the number of peaks. Y_indices represents peak indices, which dimension is (N×P) and Y_counts represents the value of peak counts, which dimension is (N×P). Each gene within a cell is represented by iZ^G and each peak is represented by jZ^P.
In the teacher network, the embedding layer maps discrete inputs such as genes, peaks, etc. to continuous vector space. The input of embedding layer of RNA teacher network is each row of X_indices, that is, an integer sequence x_1, x_2, …, x_G, where each x_i represents the index of a gene. The purpose of the embedding layer is to map these integers into a dense vector 〖gene embed〗_i∈R^d (a d-dimensional embedding vector corresponding to x_i). The embedding layer can be represented by a matrix E_RNA∈R^(G×d), where G is the number of genes, and d is the dimension of the embedding. E_RNA [x_i] represents the extraction of the x_i row (i.e. the embedding vector corresponding to the gene i) from the matrix E_RNA through a table lookup operation (equation 1). Then the RNA expression of gene i (〖count〗_i∈R) from X_counts is element-wise cross-multiplied with its embedding vector (〖gene embed〗_i) to obtain the weighted embedding vector 〖gene hidden〗_i∈R^d (equation 2). For scATAC-seq data, we use y_j to represent the index of a peak in Y_indices and the embedding layer of ATAC teacher network is represented by a matrix E_ATAC∈R^(P×d), where P is the number of peaks. The embedding vector 〖peak embed〗_j of peak j is obtained by table lookup operation (equation 3). The peak counts of peak j (〖count〗_j∈R) from Y_counts is element-wise cross-multiplied with its embedding vector (〖peak embed〗_j) to obtain the weighted embedding vector 〖peak hidden〗_j∈R^d (equation 4).

e. Definition of variables such as m and m+ introduced only after they are first used:

Modify “Overview of model architecture” section in Methods.

As illustrated in Figure 1b, the input of Attune is a multimodal dataset, also called joint profiling data, which contains information from two modalities, and the cells of the two modalities are paired. Attune is designed based on joint profiling data, which considers the pairing information. We consider the cells in paired scRNA-seq and scATAC-seq as positive pairs (m and m^+ is a pair of positive samples).
The overarching model architecture encompasses the Attune pre-training model, which comprises two asymmetric teacher-student networks, along with two modules dedicated to downstream tasks: the cross-modal prediction module and the transformer-based peak-gene interaction module. Attune leverages separate teacher-student networks to learn cell embeddings from scRNA-seq and scATAC-seq respectively, through cross-modal contrastive learning. The teacher network, designed to be more complex, employs a hierarchical attention mechanism 67, while the student network uses a simpler dense operation.
Learn the representation of cells in two modalities (scRNA-seq and scATAC-seq) by maximizing the consistency between positive pairs in the embedding space. The representations from both modalities are then projected into a common space.

f. Definition of the function I() in equation 26:

Modify “Training objectives” section in Methods.

We train transformer with two objectives function: contrastive loss between gene 〖hidden〗_CLS and peak 〖hidden〗_CLS, RNA-ATAC modality matching loss. gene 〖hidden〗_CLS and peak 〖hidden〗_CLS are CLS tokens in different modalities with N*1*d dimension. They learn the weighted average embedding representing the entire genes or peaks. The purpose of comparing the two embeddings is to shorten the distance between the matched RNA-ATAC pairs globally. The calculation process of contrastive loss of CLS tokens, is described as equation (24-26).

We sincerely hope that the revisions we have made effectively address your concerns. Regarding your suggestion to rewrite the entire Methods section, we have carefully reviewed and reorganized it to enhance clarity. We deeply appreciate your insightful feedback, which has significantly contributed to improving the overall quality of the manuscript.

Reviewer#2 comments 2. Given that Attune uses paired information in the loss

function, is it fair to compare this with other unpaired methods in the data integration performance, such as scJoint? A note should be made for the unpaired methods to clarify.

Response to Reviewer#2 comments 2:

We thank Reviewer#2 for raising this important point. Initially, we selected scJoint and other unpaired methods for comparison due to the limited availability of methods specifically designed for paired data integration at the time. However, we acknowledge that comparing a paired method like Attune with unpaired methods such as scJoint and GLUE may introduce fairness concerns, as these unpaired methods, while capable of handling paired data, do not fully leverage pairing labels.
In response to this concern, and as detailed in our reply to Reviewer#1's Comment 3, we have now included additional benchmarking with other paired data methods such as Seurat V5, SMILE, and Concerto. The updated results show that even when compared to these paired methods, Attune maintains competitive performance, further supporting its efficacy in multimodal integration. Since the detailed benchmarking results are already provided in the response to Reviewer#1’s Comment 3, we do not repeat the results here, but we encourage the reviewer to refer to that section for further details. We hope this addresses the fairness concern raised by Reviewer#2.

Reviewer#2 comments 3. Methods and evaluation metrics that are mentioned in the recent benchmarking paper in single-cell multi-omics prediction and integration (Hu et al.) should be considered in this manuscript to improve the evaluation and benchmarking.
Hu, Y., Wan, S., Luo, Y. et al. Benchmarking algorithms for single-cell multi-omics prediction and integration. Nat Methods (2024).

Response to Reviewer#2 comments 3:

We thank Reviewer#2 for the valuable suggestion. In response, we have indeed benchmarked our method against the recent benchmarking work by Hu et al. (2024) on single-cell multi-omics prediction and integration [1]. A detailed discussion on predicting chromatin accessibility from scRNA-seq data is provided in our response to Reviewer#1’s Comment 1.
Regarding the integration of RNA expression and chromatin accessibility (referred to as vertical integration), we benchmarked Attune alongside several algorithms highlighted in the benchmarking paper by Hu et al., including scAI, MOJITOO, MultiVI, Seurat, scVAEIT, MOFA+, Multigrate, scMVP, MIRA, DeepMAPS, SCOIT, and Schema. These comparisons were performed across 11 RNA + ATAC datasets (see Response Figure 18-20), which are joint profiling data derived from various technologies such as SNARE-seq, SHARE-seq, ISSAAC-seq, 10x Multiome, and DOGMA-seq (Response Figure 21). For the evaluation of vertical integration, we used multiple metrics, including ARI (Adjusted Rand Index), NMI (Normalized Mutual Information), cASW (cell-type labels average silhouette width), cLISI (cell-type separation LISI), and BVC (biological variation conservation). ARI and NMI were used to assess the consistency between cell-type labels and clustering results obtained through the Leiden algorithm. A higher cASW value indicates better accuracy in cell-type separation, while higher cLISI values suggest more effective cell-type separation, indicating better preservation of biological variation. The BVC metric provides a comprehensive performance evaluation, aggregating several metrics (ARI, NMI, cASW, and cLISI) into a single score.
In terms of NMI and ARI, MOJITOO achieved the highest scores, followed by Attune and scAI (Response Figure 18). For the cASW and cLISI metrics, Attune outperformed the other algorithms (Response Figure 19). These results indicate that, while Attune’s performance in cell clustering was slightly lower than that of MOJITOO, it demonstrated superior cell-cell similarity representation, which is a critical factor for accurate integration. Overall, Attune, scAI, and MOJITOO performed the best in terms of vertical integration, as shown in Response Figure 19. We would like to emphasize that the datasets and evaluation pipeline used in our vertical integration benchmarks are consistent with those described in the benchmarking paper by Hu et al. (2024). The data can be accessed at https://mailustceducn-my.sharepoint.com/:f:/g/personal/hyl2016_mail_ustc_edu_cn/EgYFP7tlTKBBuAhkdtrIOg4B1Eyo-_iBx1VKBWSK0r-9rA?e=gmhocx, and the evaluation pipeline is available at

Powered by Editorial Manager® and ProduXion Manager® from Aries Systems Corporation

	https://github.com/QuKunLab/MultiomeBenchmarking/blob/main/code/Integration/compare/count_metrics_ATAC.ipynb.<br>We hope these additional details address your concerns and strengthen our manuscript.<br><br>[1] Hu Y, Wan S, Luo Y, et al. Benchmarking algorithms for single-c...
Additional Information:	
Question	Response
Are you submitting this manuscript to a special series or article collection?	No
**Experimental design and statistics**<br><br>Full details of the experimental design and statistical methods used should be given in the Methods section, as detailed in our Minimum Standards Reporting Checklist. Information essential to interpreting the data presented should be made available in the figure legends.<br><br>Have you included all the information requested in your manuscript?	Yes
**Resources**<br><br>A description of all resources used, including antibodies, cell lines, animals and software tools, with enough information to allow them to be uniquely identified, should be included in the Methods section. Authors are strongly encouraged to cite Research Resource Identifiers (RRIDs) for antibodies, model organisms and tools, where possible.<br><br>Have you included the information requested as detailed in our Minimum Standards Reporting Checklist?	Yes
**Availability of data and materials**<br><br>All datasets and code on which the conclusions of the paper rely must be either included in your submission or deposited in publicly available repositories (where available and ethically	Yes

appropriate), referencing such data using a unique identifier in the references and in the “Availability of Data and Materials” section of your manuscript.<br><br>Have you have met the above requirement as detailed in our Minimum Standards Reporting Checklist?	

Revised manuscript

Click here to access/download;Manuscript;Manuscript - 241230.docx

Cross-modal contrastive learning discovers chromatin potential regulating gene expression of single cell atlas

Yueyuxiao Yang[1], Chenxi Xie[1], Qiushun He[1], Meng Yang[1]*

[1]MGI, Shenzhen 518083, China.

*Correspondence to: yangmeng1@mgi-tech.com

Abstract

Background

Emerging large-scale multimodal single-cell data jointly measures chromatin accessibility and transcription in the same cell, thus reconciling matched data paves integrated route for comprehensive regulatory analysis.

Results

Here, we introduce Attune, a cross-modal contrastive learning framework to align paired gene expression and accessibility information. Systematic benchmarking shows Attune's superior performance for omics integration and gene expression prediction. We further introduce Transformer-based cross-modal attention over fine-tuned gene and peak embeddings to infer regulatory interaction and discover significant differential signals of cell subtypes. Applied to hair follicle maturation dataset, Attune reveals chromatin potential for bifunctional transcription factor Gli3 at the gene level. In addition, the paired representations determine transmitted states across neonatal and mature cell types of cortical neuron differentiation at the cell level. Taken together, Attune features a promising paradigm for regulatory inference across omics layers and allows for extending more complex omics analysis.

Conclusions

Attune offers a versatile framework for integrating gene expression and chromatin accessibility, enabling the inference of regulatory mechanisms and the prediction of gene expression from cross-modal data.

Background

Gene transcription is a dynamic process that drives the precise differentiation of cell lineages[1]. This complex process is orchestrated by coordinated regulation of chromatin accessibility around key regulatory elements, such as promoters and enhancers, which creates a permissive landscape for the binding of transcription factors and co-factors, thus initiating transcription[2].

Recent advancements in multimodal sequencing technologies, such as 10x Multiome, SHARE-seq[3], SNARE-seq[4] and scCAT-seq[5], have enabled the simultaneous measurement of multiple layers of a single cell, including chromatin and transcriptional status. By capitalizing on these data, it becomes conceivable to refine cell identity and reconstruct the causal sequence of the regulatory network[6] that underlies cellular differentiation. Moreover, by integrating information from multiple modalities within a joint embedded space and accounting for temporal dynamics, we can unearth the intricate relationships and dependencies that permeate across these modalities. Despite the existence of various techniques for integrating multimodal single-cell data, such as Seurat V4[7], MultiVI[8], BABEL[9], they are predominantly focused on integration or prediction tasks, resulting in a pressing need for approaches to elucidate the regulation of cell differentiation. GLUE[10] employs a guidance graph to explicitly model cis-regulatory interactions between feature spaces, while MIRA utilizes a combination of topic modeling and regulatory potential modeling to capture key regulators at lineage branch points[11]. Nevertheless, the pool of available methods specifically designed for this purpose remains limited.

Multimodal deep learning, a formidable technique within the realm of computer science, offers a panoramic understanding of data, wielding substantial power. For example, Vision-Language (VL) pre-training has demonstrated remarkable efficacy in various VL downstream tasks[12], as evidenced by the success of CLIP[13] and ALBEF[14]. These models utilize a contrastive learning module to pretrain the encoder and subsequently finetune it with a transformer-based decoder. In the context of single-cell multi-modal data, the deployment of multimodal deep learning algorithms becomes profoundly advantageous. Drawing inspiration from the exceptional representation capabilities of contrastive learning for multimodal data[13,15], as well as the impressive performance of transformer models in uncovering latent interactions[14,16], we introduce Attune: a cross-modal contrastive learning pre-training model designed to capture the interactions between different modalities. By maximizing the agreement between modalities within a cell on the hypersphere[17], Attune places representations of distinct modalities into a shared feature space, enabling the modeling of interactions between peaks and genes.

Using Attune, we apply learned cell embeddings to a range of downstream tasks and achieve superior performance in cross-modal prediction tasks. By employing a transformer-based decoder, we can construct gene-peak interaction networks and interrogate the regulations underlying key developmental processes, such as the maturation of transit-amplifying cells in the hair follicle dataset and the active transition region during neuron differentiation in the cortex dataset. Comprehensive benchmarking and regulation analysis demonstrate the power of Attune in learning comprehensive and informative representations of omics-specific features and reconstructing regulatory interactions.

Methods

Input data & preprocessing

The Attune model takes expression (gene count) and accessibility (peak count) matrices from matched multimodal scRNA-seq and scATAC-seq as input data. For scRNA-seq data, genes expressed in fewer than 5% of cells were filtered out. We used SCANPY[66] to normalize each cell count to 10,000 read counts before the logarithm. Additionally, sex chromosome genes were removed, and 2000 highly variable genes (HVGs) were selected based on the experiments depicted in Figure 2d providing a reasonable compromise. This choice allows us to achieve satisfactory performance while managing the computational cost and ensuring the overall stability and efficiency of the model. For scATAC-seq data, peaks accessed in fewer than 5% of cells were filtered out. And peaks from sex chromosomes were also filtered out. A complete list of all data used in the study is provided in Table S1.

Input encoding scheme

The normalized expression and accessibility matrices were encoded in the TensorFlow Record (TF-record) format. The scRNA-seq data was encapsulated in one TF-record file, with 'gene index' and 'gene count' fields, while the scATAC-seq data was encapsulated in another file, with 'peak index' and 'peak count' fields.

Overview of model architecture

As illustrated in Figure 1b, the input of Attune is a multimodal dataset, also called joint profiling data, which contains information from two modalities, and the cells of the two modalities are paired. Attune is designed based on joint profiling data, which considers the pairing information. We consider the cells in paired scRNA-seq and scATAC-seq as positive pairs (m and m^+ is a pair of positive samples).

The overarching model architecture encompasses the Attune pre-training model, which comprises two asymmetric teacher-student networks, along with two modules dedicated to downstream tasks: the cross-modal prediction module and the transformer-based peak-gene interaction module. Attune leverages separate teacher-student networks to learn cell representations from scRNA-seq and scATAC-seq respectively, through cross-modal contrastive learning. The teacher network, designed to be more complex, employs a hierarchical attention mechanism [67], while the student network uses a simpler dense operation. Learn the representation of cells in two modalities (scRNA-seq and scATAC-seq) by maximizing the consistency between positive pairs in the embedding space. The representations from both modalities are then projected into a common space.

We adapted the pre-trained Attune model to three downstream tasks: inference of gene-peak interaction and cross-modal prediction and differentiation analysis. Reconstructing of regulatory events requires the model to reconstruct the correspondence between accessible chromatin and gene expression and ascertain which chromatin regions are responsible for the change in gene expression across cells. To accomplish this, we utilized a transformer-based decoder that captures multimodal cross attention, thereby establishing the link between peaks and genes. For cross-modal prediction, Attune was fine-tuned via a multi-layer perceptron (MLP) to predict gene expression and further identify temporal differences in expression accessibility and transcription for differentiation analysis.

Teacher network

For two kinds of single-cell data (scRNA-seq and scATAC-seq), we designed two teacher networks (RNA teacher network and ATAC teacher network) to learn fine-grained representations respectively. The RNA teacher network accepts $X_{indices} \epsilon \mathbb{R}^G$ and $X_{counts} \epsilon \mathbb{R}^G$ as input, where G denotes the number of genes. $X_{indices}$ represents gene indices, which dimension is (N×G) and X_{counts} represents the value of gene expression, which dimension is (N×G). N is the number of cells. For ATAC teacher network accepts input of $Y_{indices} \epsilon \mathbb{R}^P$ and $Y_{counts} \epsilon \mathbb{R}^P$, where P denotes the number of peaks. $Y_{indices}$ represents peak indices, which dimension is (N×P) and Y_{counts} represents the value of peak counts, which dimension is (N×P). Each gene within a cell is represented by $i \epsilon \mathbb{Z}^G$ and each peak is represented by $j \epsilon \mathbb{Z}^P$.

In the teacher network, the embedding layer maps discrete inputs such as genes, peaks, etc. to continuous vector space. The input of embedding layer of RNA teacher network is each row of $X_{indices}$, that is, an integer sequence x_1, x_2, …, x_G, where each x_i represents the index of a gene. The purpose of the embedding layer is to map these integers into a dense vector $gene\ embed_i \in \mathbb{R}^d$ (a d-dimensional embedding vector corresponding to x_i). The embedding layer can be represented by a matrix $E_{RNA} \in \mathbb{R}^{G \times d}$, where G is the number of genes, and d is the dimension of the embedding. $E_{RNA}[x_i]$ represents the extraction of the x_i row (i.e. the embedding vector corresponding to the

gene i) from the matrix E_{RNA} through a table lookup operation (equation 1). Then the RNA expression of gene i ($count_i \in \mathbb{R}$) from X_{counts} is element-wise cross-multiplied with its embedding vector ($gene\ \ embed_i$) to obtain the weighted embedding vector $gene\ \ hidden_i \in \mathbb{R}^d$ (equation 2). For scATAC-seq data, we use y_j to represent the index of a peak in $Y_{indices}$ and the embedding layer of ATAC teacher network is represented by a matrix $E_{ATAC} \in \mathbb{R}^{P \times d}$, where P is the number of peaks. The embedding vector $peak\ \ embed_j$ of peak j is obtained by table lookup operation (equation 3). The peak counts of peak j ($count_j \in \mathbb{R}$) from Y_{counts} is element-wise cross-multiplied with its embedding vector ($peak\ \ embed_j$) to obtain the weighted embedding vector $peak\ \ hidden_j \in \mathbb{R}^d$ (equation 4).

$$gene\ \ embed_i = E_{RNA}[x_i] \qquad (1)$$

$$gene\ \ hidden_i = gene\ \ embed_i \times count_i \qquad (2)$$

$$peak\ \ embed_j = E_{ATAC}[y_j] \qquad (3)$$

$$peak\ \ hidden_j = peak\ \ embed_j \times count_j \qquad (4)$$

Then we use the attention mechanism to aggregate gene or peak embeddings. The input $gene\ \ hidden_i$ is passed through a multilayer perceptron with one hidden layer and a nonlinear tanh transformation. A cellular context vector $u \in \mathbb{R}^d$ then applies the dot product to $gene\ \ hidden_i$, using the softmax operation to obtain $gene\ \ attention_i \in \mathbb{R}^d$ (equation 5). The cell context vector u serves as an intermediate variable in computing hierarchical attention, which is a weighted value on 128-dimensional embeddings. Aggregation is then applied to the genes' vectors $gene\ \ hidden_i$ through weighted summation by $gene\ \ attention_i$, to obtain aggregated vectors, $RNA\ \ hidden$ (equation 6), with N*d dimension. The same process is applied to scATAC-seq data, as shown in equation (7-8).

$$gene\ \ attention_i = \mathrm{softmax}(\tanh(gene\ \ hidden_i) \cdot u) \qquad (5)$$

$$RNA\ \ hidden = \sum_i (gene\ \ attention_i \times gene\ \ hidden_i) \qquad (6)$$

$$peak\ \ attention_j = \mathrm{softmax}(\tanh(peak\ \ hidden_j) \cdot u) \qquad (7)$$

$$ATAC\ \ hidden = \sum_j (peak\ \ attention_j \times peak\ \ hidden_j) \qquad (8)$$

We apply the attention mechanism output to fed into a batch normalization layer followed by a dropout layer. Then a dense layer with ReLU activation projects to the final output of RNA teacher network, $Z_{teacher}^{RNA} \in \mathbb{R}^d$ (equation 9) and the final output of ATAC teacher network, $Z_{teacher}^{ATAC} \in \mathbb{R}^d$ (equation 10).

$$Z_{teacher}^{RNA} = Dense(RNA\ \ hidden) \qquad (9)$$

$$Z_{teacher}^{ATAC} = Dense(ATAC\ \ hidden) \qquad (10)$$

Student network

We also designed two student networks (RNA student network and ATAC student network) to learn coarse-grained representations respectively. The student network accepts only

$X_{counts} \epsilon \mathbb{R}^G$ or $Y_{counts} \epsilon \mathbb{R}^P$, then passing a batch normalization layer followed by a dropout layer and a dense layer with ReLU activation projects to the final output of the RNA student network $Z_{student}^{RNA} \in \mathbb{R}^d$ (equation 11). The final output of the ATAC student network is $Z_{student}^{ATAC} \in \mathbb{R}^d$ (equation 12).

$$Z_{student}^{RNA} = Dense(x_{counts}) \quad (11)$$

$$Z_{student}^{ATAC} = Dense(y_{counts}) \quad (12)$$

Cross-modal contrastive loss

Contrast learning is implemented by the explicit comparison of the d-dimensional embedding (where d = 128 by default) of a cell on two modalities on a unit hypersphere. Positive sample pairs are created by taking two modal representations of a cell and pulling them together, while negative samples are created by taking different cells and widening the distance between them. Four different embeddings: $Z_{student}^{ATAC}$, $Z_{student}^{RNA}$, $Z_{teacher}^{ATAC}$, $Z_{teacher}^{RNA}$ are obtained using two independent asymmetric teacher-student networks. Assume the embedding is obtained by the teacher network and the cosine similarity with L2 regularization of the two given embeddings (the embedding under the same network structure) is defined by equations (13-14). The positive pair as $cell_m$ (whose embedding is $z_{teacher_m}^{RNA} \in Z_{teacher}^{RNA}$) and $cell_{m^+}$(whose embedding is $z_{teacher_{m^+}}^{ATAC} \in Z_{teacher}^{ATAC}$). The NT-Xent loss represents the normalized temperature-scaled cross-entropy loss, as formalized by equation (15), where m and m^+ is a pair of positive samples. We randomly sample a min-batch of N cells and compute NT-Xent loss on pairs of cross-modal examples derived from the mini-batch, resulting in 2N data points. Given a positive pair, the other 2(N-1) cross-modal examples within a mini-batch are treated as negative examples. The calculation process of NT-Xent loss for the embedding obtained by the student network is the same, see equation (16-18). The full pretraining objective of Attune model sees equation (19).

$$s_{\alpha,\beta} = sim\left(z_{teacher_\alpha}, z_{teacher_\beta}\right) \quad (13)$$

$$s_{\alpha,\beta}^{+} = sim\left(z_{teacher_\beta}, z_{teacher_\alpha}\right) \quad (14)$$

where $sim(h_1, h_2)$ is defined as:

$$sim(h_1, h_2) = \frac{h_1^T h_2}{\tau \|h_1\| \|h_2\|}$$

$$\mathcal{L}_{teacher} = \frac{1}{2N} \sum_{m=1}^{N} [\ell(m, m^+) + \ell(m^+, m)] \quad (15)$$

where $\ell(m, m^+)$ is defined as:

$$\ell(m, m^+) = -log \frac{\exp(s_{m,m^+})}{\sum_{k=1}^{2N} \mathbb{1}_{[k \neq m]} [\exp(s_{k,m}) + \exp(s_{k,m^+})]}$$

where $\ell(m^+, m)$ is defined as:

$$\ell(m^+, m) = \quad -log\frac{\exp(s^+_{m,m^+})}{\sum_{k=1}^{2N}\mathbb{1}_{[k\neq m^+]}\left[\exp\left(s^+_{k,m^+}\right)+\exp(s^+_{k,m})\right]}$$

$$s_{\alpha,\beta} = sim\left(z_{student_\alpha}, z_{student_\beta}\right) \tag{16}$$

$$s^+_{\alpha,\beta} = sim\left(z_{student_\beta}, z_{student_\alpha}\right) \tag{17}$$

$$\mathcal{L}_{student} = \frac{1}{2N}\sum_{m=1}^{N}\left[\ell(m, m^+) + \ell(m^+, m)\right] \tag{18}$$

$$\mathcal{L}_{pretrain} = \frac{\mathcal{L}_{teacher} + \mathcal{L}_{student}}{2} \tag{19}$$

where τ is the adjustable temperature coefficient, which can be used to scale the degree of pushing apart negative samples.

Joint representation and UMAP visualization

Contrast learning can integrate cells from different modalities together. Meanwhile, to join the cell embeddings of two modalities together, we concatenate $Z^{ATAC}_{teacher}$ to $Z^{RNA}_{teacher}$ to get the joint embedding matrix $Z_{joint} \in \mathbb{R}^d$, with 2N* d dimension (equation 20). Cell embeddings are visualized by UMAP using SCANPY.

$$Z_{joint} = \quad Concatenate\left(Z^{RNA}_{teacher}, Z^{ATAC}_{teacher}\right) \tag{20}$$

Inference of gene-peak interaction

To recover gene-peak interaction, Attune employs contrastive learning as a pretraining procedure followed by a transformer decoder to model the relationship between peaks and genes. The transformer is a deep neural network structure for sequence modeling. The self-attention mechanism establishes attention connections between each token in a sequence, so the embedding of each token contains implicit context. Meanwhile, the cross-attention mechanism establishes attention connections between tokens in two sequences, enabling the model to extract the dependency between tokens in the two sequences. Both self-attention and cross-attention are adopted in our model. Self-attention captures intra-modality interaction, such as gene-gene relationships, while cross-attention models inter-modality interaction, which is the peak-gene relationship.

The input sequences of transformer are $gene\ hidden_i$ (N*G* d dimension) and $peak\ hidden_j$ (N*P*d dimension). Firstly, a CLS token is inserted at the beginning of a input sequence. It can also be understood as a weighted average of each token in a sequence. The embedding of CLS token is denoted as $gene\ hidden_{CLS} \epsilon \mathbb{R}^d$ and $peak\ hidden_{CLS} \epsilon \mathbb{R}^d$. Then, self-Attention is applied to $gene\ hidden_i$:

Step1: $gene\ hidden_i$ is fed into three multilayer perceptron to get vectors $Q \epsilon \mathbb{R}^d$, $K \epsilon \mathbb{R}^d$,

$V \epsilon \mathbb{R}^{d}$.

Step2: Q applies the dot product to K, using the softmax operation to obtain $Attention\ weight_i$, $Attention\ weight_i \epsilon \mathbb{R}^{G+1}$, with N*(G+1)*(G+1) dimension. $self\ \ gene\ \ hidden_i \epsilon \mathbb{R}^{d}$ is defined as (21) with a N*(G+1) *d dimension.

$$self\ \ gene\ \ hidden_i = softmax\left(\frac{QK^T}{\sqrt{d}}\right)V \tag{21}$$

Finally, Cross-Attention mechanism is applied to $peak\ \ hidden_j$ and $self\ \ gene\ \ hidden_i$: $cross\ \ gene\ \ hidden_i \epsilon \mathbb{R}^{d}$ is defined as (22) with a dimension of N*(G+1) *d.

$$cross\ \ gene\ \ hidden_i = softmax\left(\frac{QK^T + Attention\ \ mask}{\sqrt{d}}\right)V \tag{22}$$

where Q is $self\ \ gene\ \ hidden_i$ and K, V are $peak\ \ hidden_j$. $Attention\ \ mask$ (with N*(G+1) *(P+1) dimension) is defined in *Attention mask* section. Cross attention weight is defined as (23):

$$Cross\ \ attention\ \ weight = softmax\left(\frac{QK^T + Attention\ \ mask}{\sqrt{d}}\right) \tag{23}$$

Training objectives

We train transformer with two objectives function: contrastive loss between $gene\ \ hidden_{CLS}$ and $peak\ \ hidden_{CLS}$, RNA-ATAC modality matching loss. $gene\ \ hidden_{CLS}$ and $peak\ \ hidden_{CLS}$ are CLS tokens in different modalities with N*1*d dimension. They learn the weighted average embedding representing the entire genes or peaks. The purpose of comparing the two embeddings is to shorten the distance between the matched RNA-ATAC pairs globally. The calculation process of contrastive loss of CLS tokens, is described as equation (24-26).

$$s_{\alpha,\beta} = sim\left(gene\ \ hidden_{CLS_\alpha}, peak\ \ hidden_{CLS_\beta}\right) \tag{24}$$

$$s^{+}_{\alpha,\beta} = sim\left(peak\ \ hidden_{CLS_\beta}, gene\ \ hidden_{CLS_\alpha}\right) \tag{25}$$

where $sim(h_1, h_2)$ is defined as:

$$sim(h_1, h_2) = \frac{h_1^T h_2}{\tau \|h_1\| \|h_2\|}$$

$$\mathcal{L}_{CLS} = \frac{1}{2N}\sum_{m=1}^{N}[\ell(m, m^{+}) + \ell(m^{+}, m)] \tag{26}$$

where $\ell(m, m^{+})$ is defined as:

$$\ell(m, m^{+}) = \ -log\frac{\exp(s_{m,m^{+}})}{\sum_{k=1}^{2N} \mathbb{1}_{[k \neq m]}\left[\exp\left(s_{k,m}\right) + \exp(s_{k,m^{+}})\right]}$$

where $\ell(m^{+}, m)$ is defined as:

$$\ell(m^{+}, m) = \ -log\frac{\exp(s^{+}_{m,m^{+}})}{\sum_{k=1}^{2N} \mathbb{1}_{[k \neq m^{+}]}\left[\exp\left(s^{+}_{k,m^{+}}\right) + \exp(s^{+}_{k,m})\right]}$$

where τ is the adjustable temperature coefficient, which can be used to scale the degree

of pushing apart negative samples.

For the RNA-ATAC modality matching loss, the transformer is given a batch of matched RNA-ATAC pairs (positive pairs) or mismatched RNA-ATAC pairs (negative pairs). The network's goal is to identify whether a given pair is positive or negative. We feed the transformer with $gene\ hidden_i$ and $peak\ hidden_j$ pairs, and the probability of positive and the probability of negative pair are set equal. A classifier is added on top of the CLS token's embedding $cross\ gene\ hidden_{CLS} \epsilon \mathbb{R}^d$ to predict a binary label gt, where gt is a 2-dimensional one-hot vector representing the ground-truth label. p^{match} is the probability of prediction. RNA-ATAC modality matching loss is defined in equation (27) and the full training objective of the transformer is presented in equation (28).

$$\mathcal{L}_{match} = \mathbb{E}_{(RNA,ATAC)\sim D} CE(p^{match}(RNA, ATAC), gt) \tag{27}$$

$$\mathcal{L}_{interaction} = \mathcal{L}_{CLS} + \mathcal{L}_{match} \tag{28}$$

Global cross-attention weight extraction

$Cross\ attention\ weight$ contains 2 global CLS tokens' attention weight, $CLS\ Attention\ weight^{RNA}$ (with N*(P+1) dimension) and $CLS\ Attention\ weight^{ATAC}$ (with N*(G+1) dimension). $Global\ Attention\ weight$ matrix (with G*P dimension) is defined as the dot product of $CLS\ Attention\ weight^{RNA}$ and $CLS\ Attention\ weight^{ATAC}$.

Modality prediction network

For the modality prediction task, a modality prediction network is finetuned on the Attune pretrained model to predict the RNA expression level. The network uses a relatively simple regression model, a multilayer perceptron. The input of the network is $Z_{student}^{ATAC} \epsilon \mathbb{R}^d$ and the number of hidden layer units is set to 1000, while the number of units in the last layer is set to G (the number of genes). The output of the network is the predicted value of gene counts, $X_{counts}^{pred} \epsilon \mathbb{R}^G$. The loss function of modality prediction network is defined as equation (29).

$$\mathcal{L}_{prediction} = MSE\left(X_{counts}, X_{counts}^{pred}\right) \tag{29}$$

Hyperparameter tuning

The learning rate in contrastive pretraining varies from 1×10^{-4} to 1×10^{-6} using Adam optimizer training for 20 epochs. For transformer, it trains for 5 epochs, whereas for the modality prediction network, it trains for 40 epochs. The temperature coefficient in NT-Xent loss is set to 0.1, the mini-batch size is set to 32, and the dimension of the embedding is 128. Comparative experiments are detailed in Table S5 and S6.

Metrics

Integration endeavors are assessed through an array of metrics, including mean average precision (MAP), cell type ASW, neighbor consistency (NC), Seurat alignment score (SAS),

Batch ASW, graph connectivity (GC), biology conservation, omics mixing, overall integration score and FOSCTTM.

The mean average precision (MAP) furnishes a measure of the congruity between cell types in neighboring cells, thereby quantifying the accuracy of clustering outcomes with respect to cell type assignments[10].

Cell type ASW. To evaluate the integration outputs pertaining to cell types, cell type ASW affords an assessment of the silhouette of cell type labels, suitably scaled to a value between 0 and 1[18].

Batch ASW evaluates the integration among multimodalities by computing cell modality labels, also scaled between 0 and 1[18].

Neighbor consistency (NC) measures the degree of intercellular neighbor retention after integrating multimodal data, ranging from 0 to 1, where higher values indicate better preservation[68].

Seurat alignment score (SAS) calculates the alignment score to assess how well two or more modalities have been aligned, with values ranging from 0 to 1, where higher values indicate better integration among modalities[69].

Graph connectivity (GC) evaluates the proximity of cells with the same identity across different modalities in the embedding. The GC ranges from 0 to 1, with higher values indicating better integration[10,19].

Fraction Of Samples Closer Than the True Match (FOSCTTM) measures the accuracy of modal alignment at the single-cell level in paired cells, with a range from 0 to 1, where lower values indicate higher accuracy. Studies like GLUE[10] and MMD-MA[70] have utilized FOSCTTM to assess performance.

Biology conservation is evaluated through MAP, cell type ASW, and NC, which collectively assess the biological conservation of integration. These metrics are min-max scaled, and their average is calculated as a single metric for biological conservation, as per equation (30)[10].

Omics mixing is evaluated using SAS, Batch ASW, and GC, which collectively assess the mixing performance of multi-modalities. These metrics are also min-max scaled, and their average is calculated as a single metric for omics mixing, as per equation (31)[10].

The overall integration score is computed as an overall weighted average of omics mixing and bio-conservation scores, as per equation (32)[10,19].

$$Biology\ \ conservation = \frac{scale(MAP) + scale(Cell\ \ type\ \ ASW) + scale(NC)}{3} \tag{30}$$

$$Omics\ \ mixing = \frac{scale(SAS) + scale(Omics\ \ layerASW) + scale(GC)}{3} \tag{31}$$

$$Overall\ \ integratin\ \ score = \ 0.6 \times Biology\ \ conservation + 0.4 \times Omics\ \ mixing \quad (32)$$

Various metrics are employed to evaluate the proposed solution in modality prediction. These metrics include the root-mean-square-error (RMSE), gene-wise Pearson correlation coefficient, and gene-wise Spearman correlation coefficient. The RMSE serves to appraise the precision of RNA expression prediction across individual cells[19], while gene-wise Pearson or Spearman correlation coefficients gauge the average per-gene correlation in Polarbear[23] and BABEL[9].

Evaluation on inference of regulatory interaction

Promoter Capture Hi-C (PCHi-C) enables identification of long-range interactions between gene promoters and regulatory elements such as enhancers and other potential regulatory elements. Promoter interactomes are highly cell type specific and interacted regions quantitatively contribute to gene expression[27,71]. To demonstrate the potential of cross-modal association, we utilize the PCHi-C dataset of human primary hematopoietic cells. With the aim of consistency, only common cell types in the 10x Multiome and PCHi-C datasets, including T cells, B cells and monocytes, are considered for the comparison of different methods. Coordinates of interactions from PCHi-C binding matrix whose CHiCAGO interaction scores pass a cutoff of 5 in at least one cell type are lifted over[72] to Genome Reference Consortium Human Build 38 and ordered based on the distance between the midpoint of baited regions and other ends. The distance statistics of interactions are depicted in Figure 3a. Following the guidelines established in GLUE[10], we generate a truth set of peak-gene pairs supported by PCHi-C. These rules consider the proximity (within 1kb) of the gene promoter to a bait fragment and the peak's proximity (within 1kb) to the other-end fragment, along with significant interaction identified in PCHi-C. By considering the distance statistics of the PCHi-C data and controlling for noise introduced by abundant distal regions, we explore a range of gene-peak distances from 150kb to 1500kb (Supplementary Figure 4a) and determine 1200kb as the threshold for all subsequent experiments.

Specifically, during the calculation of the transformer module, we focus on the relationship between the gene promoter and peaks within a 1.2Mb region surrounding it. By masking the peaks outside this region, we encourage the transformer module to prioritize cross-modal attention within the adjacent regions of the gene. Attention weights are calculated for each gene-peak pair within 1200kb, and the “sklearn.metrics.roc_auc_score” function is utilized to assess whether the attention weights can reflect the promoter interactome.

Differential expression analysis and gene ontology enrichment

We use the “FindAllMakers” function of the Seurat[7] package to identify differentially expressed genes (DEGs) within each cell type (one versus others, p.adjust<0.01, log fold change>0.25). The top 10 and bottom 10 genes are chosen as top DEGs. Collection of

gene sets (GO:BP in C5 category) from the Molecular Signatures Database (MSigDB)[73] is used for over-presentation analysis by clusterProfiler[74]. We keep an ontology with smaller pvalue when the geneID is repeated.

Pseudotime inference

We focus on the differentiation of transient amplifying cells and select cell types including transit-amplifying cells (TAC), inner root sheath (IRS), medulla, and hair shaft from the SHARE-seq dataset of mouse skin, leading to 6k cells. 10 topics are then determined by cisTopic[75] using chromatin accessibility data. The default parameters are used, except for burin=120 and iterations=150 in the "runModels" functions. Z score is then computed by the "modelMatSelection" function as input of Palantir[76] for generating diffusion maps and pseudotime with n_components=10 of the "run_diffusion_maps" function.

Residual of modalities

The residual of each gene is calculated from normalized predicted RNA counts based on ATAC minus normalized measured RNA counts. We evaluate trends with pseudotime using a generalized additive model (GAM) and filter them based on standard deviation. We use the "argrelextrema" function in the "scikit-learn" package to define gene expression pattern.

Motif and regulon analysis

JASPAR CORE Vertebrata 2022 database[77] is selected for motif matching which contains 841 motifs. We set p.cutoff to 5×10^{-5} for filtering motifs. To illustrate the regulatory network, we use SCENIC[36], a workflow that exploits co-expression between genes and transcription factors, to analyze regulons in the SHARE-seq dataset. All modules are kept in the step of regulon prediction (add a parameter "-a") because of the known negative effect of Gli3. The AUC threshold is 0.05.

Soft cluster of chromatin accessibility data

Chromatin accessibility data at the cellular level is extremely sparse, resulting in dramatic fluctuations even within the same cell type, while it is coarse at the cell type level. To mitigate this issue, we aggregate cells into pseudo-bulk samples by dividing cells into 10 groups along pseudotime before soft clustering[38]. The cell-type composition of each group is shown in Figure 3i. Mean values are calculated and standardized for each pseudo-bulk sample. To estimate the optimized number of cluster centroids c, we perform soft clustering with a range of cluster numbers from 2 to 20. And 4 is determined as cluster number by

the centroid distance plot. We extract alpha cores of each cluster using acore=0.5, which preserves 18 peaks while discarding discards cluster 1 and cluster 4. To increase the concentration of members, we ultimately select 12 peaks from cluster 2 and cluster 3.

Co-occurrence of genes and peaks

To identify the latent interacting genes of each cluster, we first calculate Spearman's correlation between each peak in clusters and genes, and then average the values of each gene for each cluster. With the purpose of eliminating contingency, 50 peaks for each peak are selected as the background based on GC content and coverage using the "getBackgroundPeaks" function of ChromVAR[78] package. Wilcoxon rank sum test is performed to examine the difference.

Running benchmarks

GLUE (v0.3.2)[10], uniPort (v1.1.2)[79], Cobolt[80] (v1.0.1), MinNet[81], scJoint[20], MultiVI (v0.19.0)[8], sciCAN[82] were conducted using the Python (v3.6). We followed the tutorials for each method: GLUE (https://scglue.readthedocs.io/en/latest/tutorials.html), uniPort (https://uniport.readthedocs.io), Cobolt (https://github.com/epurdom/cobolt/blob/master/docs/tutorial.ipynb), scJoint (https://github.com/SydneyBioX/scJoint/tree/main/tutorial), MultiVI (https://docs.scvi-tools.org/en/stable/tutorials/notebooks/MultiVI_tutorial.html), sciCAN (https://github.com/rpmccordlab/sciCAN). We conducted Seurat V3[83] using the R (v4.1.2) and the tutorial at https://satijalab.org/seurat/articles/atacseq_integration_vignette.html. All the methods were used the default settings and data preprocessing steps as recommended. Notably, scJoint, Seurat V3, sciCAN and MinNet require converting peak counts into gene activity scores (https://stuartlab.org/signac/articles/pbmc_vignette.html#create-a-gene-activity-matrix).

Results

Attune achieves exceptional overall performance of integration

Attune employs a cross-modal contrastive learning approach to integrate scRNA-seq and scATAC-seq data, effectively preserving biological consistency across both modalities. The architecture consists of the Attune pre-trained model and two downstream modules: the cross-modal prediction module and the Transformer-based peak-gene interaction module. To learn cell embeddings from scRNA-seq and scATAC-seq data, Attune utilizes two asymmetric teacher-student networks, which are trained through cross-modal contrastive learning. These learned cell embeddings can be fine-tuned for various downstream tasks,

including cross-modal prediction, peak-gene interaction recovery, and differentiation analysis, as depicted in Figures 1a and 1b.

To demonstrate the performance of Attune, we benchmark it against other multimodal integration methods on matched scRNA-seq and scATAC-seq datasets, such as 10x Multiome and SHARE-seq, with several established metrics[10,18], most of which have been widely embraced and validated in previous single-cell integration tasks, such as graph connectivity (GC) in the NeurIPS 2021 competition[19] and average silhouette width (ASW) in scJoint[20] (see Methods in detail).

As shown in Figure 2a-left (refer to Table S2 for details), Attune emerges as the clear winner, situated in the top-right corner of the 10x Multiome dataset, signifying that it strikes a balance between omics mixing and biological fidelity. MultiVI, on the other hand, demonstrates higher omics mixing but sacrifices biological meaning. Moreover, Attune outperforms other methods on the SHARE-seq dataset (Figure 2a-right, refer to Table S3 for details), as evaluated by three separate metrics (see Supplementary Figure 1). Upon probing Attune's integration scores on distinct datasets, we observe that it obtains the highest overall integration scores (mean 0.806 and 0.829 for 10x Multiome and SHARE-seq data, respectively) in Figure 2b-left. The UMAP visualization of the cell embeddings for the 10x Multiome and SHARE-seq datasets is presented in Supplementary Figure 2 and Supplementary Figure 3, respectively. We also quantify the alignment performance between modalities using the Fraction Of Samples Closer Than the True Match (FOSCTTM) on both datasets, as depicted in Figure 2b-right. The lowest FOSCTTM scores suggest that Attune effectively matches different modalities from a cell.

To further evaluate the performance of Attune's feature distributions on the output unit hypersphere, we measure the alignment between cross-modal positive pairs and the uniformity of the entire representation space. This assessment allows us to determine the quality of learned embeddings. Compared to other integration techniques' cell embeddings (Figure 2c and Table S4), Attune achieves the best alignment (the lower the better), indicating that the feature distribution between RNA-ATAC pairs is more consistent in the high dimensional space. Other components regarding feature selection settings and hyperparameter choice are presented in Figure 2d and Figure 2e respectively (refer to Table S5 and Table S6 for more information) to substantiate the rationality under the current settings, thus enhancing the validity of the study.

The cells' embedding after integration establishes the cell labels and ontologies. Through multimodal reference building and mapping of query cells, it demonstrates that the embedding of cells retains their original biological characteristics[21,22]. We assess Attune's ability to map query cells, including previously unseen cell types, onto reference

embeddings. We utilize 80% of the cells from the 10x Multiome dataset to construct the reference embeddings, while the remaining 20% serves as the query cells with unseen cell types. Specifically, we first train the reference cells to obtain the reference embeddings. Next, we directly infer the query cells using pre-trained model weights to generate the query embeddings. Finally, we concatenate the reference embeddings and query embeddings along the sample dimension and visualize them using UMAP (refer to Supplementary Figure 9). In Supplementary Figure 9a, we deliberately exclude all CD14 monocyte cells (CD14 Mono) from the reference. Despite never encountering CD14 monocyte cells during training, Attune accurately localizes them between CD16 monocyte cells (CD16 Mono) and conventional dendritic cells (CDC), with query cells positioned in proximity to their most similar reference cells. Similarly, Supplementary Figures 9b and 9c demonstrate comparable outcomes. These findings emphatically highlight Attune's prowess in integrating multimodal data and acquiring biologically meaningful embeddings.

The essence of Attune lies in its cross-modal contrastive learning module. Taking inspiration from the pioneering work of Concerto[15], we regard Attune's multimodal contrastive learning framework as an indivisible entity. However, the efficacy of integration can be influenced by different internal comparison objects. To establish the intrinsic soundness of our module design, we conduct an ablation experiment on the cross-modal contrastive learning module. Since the module employs two asymmetric teacher-student networks, we compare three different designs. The first design involves comparing the RNA student network with the ATAC student network, and correspondingly, contrasting the RNA teacher network with the ATAC teacher network, as proposed in this study. The second one compares the RNA student network with the RNA teacher network, while simultaneously compares the ATAC student network with the ATAC teacher network. Finally, the third design involves comparing the RNA student network with the ATAC teacher network, and conversely, contrasting the RNA teacher network with the ATAC student network. We assess the performance disparities among these three comparison methods in the context of multimodal integration (Table S7) and discover that the first comparison method yields the most exceptional outcomes. This observation suggests that the improved performance of Attune stems not from a mere amalgamation of network components, but rather from the ingenious architectural design.

Internal relation is well captured by Attune resulting in outstanding performance in cross-modal prediction

In cross-modal prediction, our objective is to predict all feature values for each cell in scRNA-seq using scATAC-seq, and this requires algorithms to learn the complex regulatory interactions between layers of genetic information. To assess the performance of Attune, we compare it against state-of-the-art cross-modal prediction methods such as BABEL[9] and Polarbear[23], using the 10x Multiome dataset (PBMC10k, n=11,909). We randomly divide cells into training (n=9527) and testing (n=2382) sets (bootstrapping five times), and evaluate the method using gene-wise Pearson correlation and gene-wise Spearman's correlation. Our findings show that Attune outperforms other methods with the highest Spearman's correlation coefficient (0.243), the highest Pearson correlation coefficient (0.271), and the lowest Root Mean Square Error (RMSE) of 0.528. Figure 2f provides a visual representation of our results, while Table S8 contains detailed information.

To further verify our findings, we compare the performance of Attune with the top five winners from the modality prediction task (ATAC-GEX subtask) using the official settings, datasets, and guidelines from the multimodal single-cell data integration competition of NeurIPS 2021[19]. As illustrated in Figure 2g and Table S9, Attune outperforms all other methods with the lowest RMSE.

We posit that the efficacy of cross-modal prediction may be significantly influenced by the integration of pretraining. To investigate this hypothesis, we design a comparative experiment to demonstrate the benefits derived from fine-tuning Attune's pretrained model through the utilization of a multilayer perceptron (MLP). Our investigation entails an examination of three distinct settings: an MLP network with pretraining, an MLP network without pretraining (de novo training), and classical regression methods such as LASSO (Table S10). Additionally, we conduct a comprehensive ablation study to elucidate the impact of Attune's structure on the performance of cross-modal prediction, as presented in Figure 2h and Table S11. These results prominently underscore the significant impact of Attune's pretrained model on the robustness of fine-tuning and its potential for downstream tasks.

Transformer's cross-attention mechanism enables revealing regulatory interaction of genes via fine-tuning Attune

Various metrics are utilized to infer regulatory interactions by quantifying the relationship between modalities. For instance, Cao et al.[10] utilized cosine similarity of different feature embeddings, while Trevino et al.[24] and Sai Ma et al.[3] used correlation metrics to evaluate the relationship between genes and peaks. In this work, we leverage the intrinsic property

of transformer, i.e., the ability of cross attention to discover inner connections, to quantify associations between genes and peaks by utilizing attention weight.

To demonstrate the efficacy of Transformer in discovering regulatory interactions, we employ a matched scRNA-seq and scATAC-seq Multiome dataset from 10x Genomics, consisting of approximately 11,000 human peripheral blood mononuclear cells (PBMC). Previous studies have suggested that regulatory elements, such as enhancers and silencers, may be distributed away from promoters up to several Mbps[25,26]. Based on these observations and the statistical analysis of the dataset from Javierre, B. M.[27] (see Figure 3a), we mask genes and peaks whose distances are greater than 1.2 Mbps (see Supplementary Figure 4a and Methods) for comprehensive prediction and evaluation[28,29]. We utilize a Promoter capture Hi-C (PCHi-C) dataset of human PBMC that profiles distal promoter-interacting regions as a validated resource[27]. As illustrated in Figure 3b, Attune+Transformer, i.e. training the Transformer model by fine-tuning Attune's pre-trained model, outperforms other methods, including Cicero[30], LASSO, and GLUE[10], in regulatory prediction, indicating that cross attention learned by Transformer (with Attune pretraining) captures promoter-interacting regions effectively.

To further substantiate the benefits of Attune's pre-training, we introduce an ablation experiment. This experiment incorporates the PCA+Transformer configuration, where the Transformer is trained solely on the gene embeddings and peak embeddings derived from the first 10 Principal Components (PCs) extracted via Principal Component Analysis (PCA), without the utilization of Attune pre-training. These comparisons in Figure 3b highlight the role of Attune pre-training in facilitating the Transformer's effective capture of promoter-interacting regions.

To elucidate the biological signals captured by the attention mechanism, we select gene-peak pairs with the top 10% of attention weight using an inflection point ("elbow") when ranking gene-peak pairs by attention weight (Figure 3c). A total of 8,744 gene-peak pairs remains under this cutoff, including 5,447 peaks and 646 genes (full list in Table S12). We define 466 genes linking at least 10 peaks among them as DPAGs (dense peak-associated genes). Key regulatory events may occur within DPAGs and their associated peaks[3]. Most of the DPAGs express differentially (354 versus 466, see Table S13 for details) and are enriched in immune response regulating signaling (p.adjust=0.001), mononuclear cell differentiation (p.adjust=0.001), and positive regulation of cell adhesion (p.adjust=0.005), as shown in Figure 3d and 3e. DPAGs include cell markers of plasma (JCHAIN, SEC11C), HSPC (CDK6), pDC (BCL11A, ZFAT), B naive or B memory cells (BANK1, EBF1), etc., and clear separation of cell types is observed in Figure 3f and Supplementary Figure 4b

from both modalities. Similar results of the SHARE-seq dataset are displayed in Supplementary Figure 5.

Attune enables chromatin potential discovery and illuminates the priming of lineage

Attune outperforms other methods in predicting cell modalities, but some genes exhibit low Pearson correlation coefficients. Based on the delay between chromatin accessibility and transcription[2,3,31], we propose that chromatin potential, which refers to the latent information underlying chromatin accessibility or transcriptional delay, may account for the inaccurate prediction of certain genes, particularly during lineage development. To investigate this hypothesis, we calculate the residuals between predicted and measured gene expressions on the mouse skin SHARE-seq dataset, which represents chromatin potential, as hair follicles remain cell cycle even in adulthood. Supplementary Figure 7a and 7b illustrate that residuals display diverse patterns of gene expression during cell differentiation, evident by their trends along pseudotime (see Methods). Some genes, including Hexb, Arl15, Styx, and Atp8b1, exhibit consistency between predicted and measured expressions, whereas Gli3 displays conspicuous residuals (Figure 3g, Supplementary Figure 7c). Notably, the high residuals of Gli3 emerge before cell type transition, known as lineage commitment[3], as indicated by the low-dimensional projection of cell type and pseudotime in Supplementary Figure 6a-6b and the change in residual in Figure 3h, pointing to a delay between modalities.

As an example, Gli3 serves a critical function in the Hedgehog pathway (Hh) and regulates hair follicle cycles in embryonic and adult skin[32,33]. In the canonical Hh pathway, Gli3 primarily acts as a repressor to maintain pathway activity balance[34,35]. Regulon analysis by SCENIC[36] confirms its role in transcriptional inhibition, with network importance scores for refined Gli3 regulon target genes listed in Table S14, such as Basp1 (14.03), Sema4a (5.21), and Myh14 (3.36). Given its diverse functions and multiple targets during development, we speculate that the lag of Gli3 contributes to lineage differentiation.

Regulation of Gli3 orderly shift with the maturity of cells

In a previous study, Gli3 was identified as a highly connected transcriptional repressor with limited description[3]. In this study, we aim to elucidate the lagging mechanism of Gli3 and provide a comprehensive understanding of its role in hair follicle maturity. We utilize a prediction subtask to generate expression from chromatin state signal and find that the

transition of chromatin accessibility may account for the delay of Gli3. After filtered by attention weight, 49 peaks of Gli3 are soft clustered (see Figure 3i and Method)[37,38]. As depicted in Figure 4a and Table S15, we determine four clusters, with clusters 2 and 3 having a higher number of peaks with high membership value (>0.5). Cluster 2 shows a downward trend in peak accessibility, while cluster 3 demonstrates an opposite fluctuation, suggesting that the peaks around Gli3 change in accessibility in a coordinated manner instead of independently opening or closing. To reduce noise, we further analyze 12 peaks from clusters 2 and 3 for their relationship with Gli3.

The majority of the 12 peaks are situated within 500 kb of Gli3's transcriptional start site (as depicted in Figure 4b) and are found to be accessible during the early or late differentiation stage (as shown in Figure 4c, either at the top or bottom, respectively, and Supplementary Figure 6c). To investigate the genes that impact accessibility of these 12 peaks, we perform Spearman correlation analysis between the peaks and genes to discover cluster-associated genes (see Methods). Our analysis reveals that Eda ($p = 6.01 \times 10^{-4}$), Nfib ($p = 2.20 \times 10^{-4}$), Sox5 ($p = 6.67 \times 10^{-4}$), Ntn1 ($p = 4.38 \times 10^{-4}$), and Tspan18 ($p = 4.79 \times 10^{-4}$) are the top five correlated genes for cluster 2, while Lef1 ($p = 1.52 \times 10^{-4}$), Prr5l ($p = 1.43 \times 10^{-4}$), Foxp1 ($p = 4.72 \times 10^{-5}$), Bmper ($p = 1.64 \times 10^{-4}$), and Dach1 ($p = 2.85 \times 10^{-4}$) are the top five correlated genes for cluster 3 (see Figure 4d and Table S16 for details). Notably, Nfib, Sox5, Lef1, Foxp1, and Dach1 are transcription factors. Our findings suggest that Gli3 may undergo a regulatory shift, and the peaks may either activate or deactivate in clusters 2 and 3, respectively, as shown in Figure 4e, which corresponds to the observed time lag in Gli3 expression.

Cross-talk of multiple pathways commits cell differentiation

Similar to other developmental processes[39-41], the differentiation of hair follicle stem cells is a highly regulated process that involves the interaction of multiple pathways, including Wnt, Bmp, Notch, and Hh, among others. These pathways act as positive or negative feedback loops, as described in previous studies[42,43]. Using matched multimodal data, we employ Attune to recover a complex regulatory network by a chain rule from residual to peak and to other genes. Based on the discovery of cluster-associated genes, targets, and literature review, we propose a model for hair follicle maturity (see Figure 5a).

During the early stages, when transient-amplifying cells predominate (peaks in cluster2 and their associated genes), cell proliferation continues while the function of Gli3 is hindered directly or indirectly. Eda promotes the Hh pathway via the Wnt-Eda-Shh cascade [44]. Sox9, which regulates GLI expression, can be enhanced by Sox5/6[45-47], while Nfib and

Nfia enable DNA-binding transcription activator activity and share many targets, including Gli3, Sox3, and Cdh2[48-50]. These signals decrease as cell specification becomes more pronounced.

At t3-t4 time points, the expression of Lef1 and Dach1 increases (peaks in cluster3 and their associated genes). TCF/LEF restricts Shh activity by binding to the enhancer of Gli3. Motif analysis also identifies peaks (chr13:15510896-15511196 and chr13:15395260-15395560) containing the LEF binding motif, which is consistent with previous research. Along with the Wnt pathway, Bmp[51-53], Foxp1-Runx2[54], and Prr5l-mTORC2[55-57] are implicated in the maintenance of the Hh pathway and Gli3. The down-regulation of target genes, such as Basp1[58,59] and Chd3[60], occurs upon inactivation of Hh, forming a feedback loop. In conclusion, the pattern shift of the chromatin state, initiated from chromatin potential, sheds new light on lineage priming and provides a basis for further developmental studies.

Embeddings facilitate discovery of key factors among active differentiating cells

The Attune algorithm not only ensures reliability for downstream tasks but also preserves biological signals in the high-dimensional space of cell embeddings. We delve into the embeddings between modalities in the fetal human cortex dataset (Supplementary Figure 8a-8b) and calculate cosine similarity for each pair of cells from RNA and ATAC embeddings, as depicted in Figure 5b. Higher cosine similarity values are enriched in the diagonal, with the exception of the boundary between newborn neurons (nIPC/ExN), maturing neurons (ExM), and excitatory neurons in the upper layer (ExUp). The overall upward shift of blocks indicates the lag of RNA modality. These findings are supported by the observation of more cells with high cosine distance (1 - cosine similarity) at the junction of ExN, ExM, and ExUp regions in Figure 5c and Supplementary Figure 8c.

To investigate accessibility or expression events, we group cells with a cosine distance value above 0.1 (Supplementary Figure 8d) and conduct differential expression analysis. Our analysis reveals overexpression of genes such as CNTNAP2, DCC, SLIT2, and KCND2 in this group (p.adjust<0.01 and log fold change>0.25). Notably, CNTNAP2 or DCC knock-out models have been associated with abnormalities in neuronal migration[61,62], while products of KCNH8, SLC24A2, and KCND2 involved in ion transportation have been shown to impact neuronal excitability and maturing[63].

Discussion

Attune builds upon the principles of cross-modal contrastive learning and proposes a novel approach to tackle the problem of learning robust cell representations from multimodal data on a unit hypersphere. To this end, Attune leverages two teacher-student frameworks, achieving impressive performance in integration benchmarks without compromising biological signals. Drawing inspiration from the pretraining-finetuning paradigm, Attune's embeddings can be effortlessly adapted to diverse downstream tasks via fine-tuning, as validated through cross-modal prediction and regulatory interaction inference tasks.

By juxtaposing cells from disparate modalities in a common space, Attune enables the detection of regulatory events. Among the three datasets of peripheral blood, skin, and cortex examined, Attune uncovers the relationships between chromatin accessibility and transcription features, disentangles the intricate network of lineage priming regulations, and identifies transcriptionally active cells. By expanding regulatory sequence along both feature spaces using chromatin potential and cross-modal attention, Attune provides fresh insights into the maturation of hair follicles, which entail multiple pathways. Furthermore, Attune pinpoints the local inconsistencies in embeddings in the human cortex dataset and posits the occurrence of swift transcriptional activities in nascent neurons.

An upsurge in experimental protocols combining dual-modalities, trio-modalities, and other modalities highlights the inevitability and indispensability of matched sequencing[6,64]. Such a surge poses a challenge in scaling methods to suit increased modalities and cells. Attune rises to this challenge by employing a lightweight model framework and an optimized input structure, which facilitates the easy scaling of Attune to support millions of cell atlases. By combining contrastive loss between specified modalities, Attune can handle additional modalities of data and explore intermodal connections with greater flexibility. Additionally, Attune's embeddings present a unique opportunity for portraying differentiation trajectories, which complements existing methods for trajectory analysis[65].

While Attune has demonstrated its potential across multiple scenarios, its performance verification with limited data remains insufficient. Acknowledging this limitation, we aim to augment the validated dataset in subsequent stages to enhance model robustness and validate its applicability across diverse multiple modalities.

In conclusion, Attune constitutes a potent paradigm for analyzing matched multimodal single-cell data, enabling exploration of inter-modal relations and unearthing the mechanisms driving complex biological phenomena at a single-cell resolution.

Availability of Source Code and Requirements

Project name: Attune

Project homepage: https://github.com/melobio/Attune

Operating system: Platform independent

Programming language: Python

Other requirements: Python 3.6 or higher, Tensorflow 2.5.0

License: GPL-3.0 License

Additional Files

Supplementary Table S1. Summary of the dataset used.

Supplementary Table S2. Performance evaluation of multimodal integration on 10X Multiome dataset.

Supplementary Table S3. Performance evaluation of multimodal integration on SHARE-seq dataset.

Supplementary Table S4. Performance evaluation of the quality of learned embeddings on 10X Multiome dataset.

Supplementary Table S5. Evaluation of the rationality of feature number selection.

Supplementary Table S6. Evaluation of the rationality of hyperparameter selection.

Supplementary Table S7. Ablation study on contrast between different modalities or networks in multimodal integration.

Supplementary Table S8. Performance evaluation of cross-modal prediction on 10X Multiome dataset.

Supplementary Table S9. Performance evaluation of cross-modal prediction on competition of NeurIPS 2021.

Supplementary Table S10. Evaluating the impact of Attune pretraining on cross-modal prediction.

Supplementary Table S11. Ablation study on contrast between different modalities or networks in cross-modal prediction.

Supplementary Table S12. All attention weight between gene and peak of 10x PBMC Multiome dataset.

Supplementary Table S13. Differential expression of 466 dense associated genes.

Supplementary Table S14. Network importance scores for target genes of refined Gli3 regulons.

Supplementary Table S15. Membership values of four clusters are calculated from Gli3 peaks.

Supplementary Table S16. Correlation between genes and cluster 2 or 3.

Supplementary Fig. S1. Metrics of evaluating integration performance.

Supplementary Fig. S2. UMAP visualization of the cell embeddings in the 10X Multiome dataset aligned with different integration methods.

Supplementary Fig. S3. UMAP visualization of the cell embeddings in the SHARE-seq dataset aligned with different integration methods.

Supplementary Fig. S4. (a) Benchmark of AUC score in different distance and algorithm settings. (b) Marker genes from Figure 2D are visualized in RNA modality.

Supplementary Fig. S5. (a) Attention weight is in descending order and the elbow is tipped in the Top 5% on the SHARE-seq dataset. (b) Expression difference of 246 DPAGs. Some Top DEGs are highlighted. (c) Heatmap of gene expression or accessibility with cell types. Each row represents a gene-peak pair extracted by attention weight.

Supplementary Fig. S6. (a) UMAP of SHARE-seq dataset colored by cell type. (b) UMAP of SHARE-seq dataset colored by pseudotime. (c) Abundances of 12 peaks from cluster2 and 3 are plotted in different cell types.

Supplementary Fig. S7. The pattern of Residual. (a) Top 100 and (b) Bottom 100 genes selected for visualization based on variance. (c) Residuals of some highly expressed and low expressed genes.

Supplementary Fig. S8. (a) UMAP of human fetal cortex dataset colored by cell type. (b) UMAP of human fetal cortex dataset colored by pseudotime. (c) Distribution of cosine distance among cell type. (d) Cells with cosine distance above 0.1 are highlighted in the embedding.

Supplementary Fig. S9. Illustration of Attune's ability to project unseen cell types onto a reference on the 10x Multiomics dataset. (a) CD14 Mono (b) CD4 Naïve (c) CD4 TCM.

Author contributions

M.Y. conceived the problem and designed the study. Y.Y. and Q.S. performed bioinformatics analysis. C.X. performed algorithm design and deep learning experiments. Y.Y. and Q.S. and C.X. wrote the manuscript.

Data Availability

All datasets utilized in this study are publicly accessible. The scRNA-seq and scATAC-seq data for PBMC are available from 10X Genomics (https://support.10xgenomics.com/single-cell-multiome-atac-gex/datasets/1.0.0/pbmc_granulocyte_sorted_10k). The SHARE-seq dataset, which includes data from mouse skin in the late anagen stage, can be accessed via NCBI GEO (accession code: GSE140203). The NeurIPS dataset, comprising data from human bone marrow, is publicly available on GEO (accession code: GSE194122). Additionally, the Greenleaf 2021 dataset, derived from human brain cortex, is provided through GEO (accession code: GSE162170). A complete list of all datasets and additional information can be found in Table S1.

Competing interests

The authors declare that they have no competing interests.

References

1. Nimmo, R.A., May, G.E., and Enver, T. (2015). Primed and ready: understanding lineage commitment through single cell analysis. Trends in Cell Biology *25*, 459-467. https://doi.org/10.1016/j.tcb.2015.04.004.
2. Li, B., Carey, M., and Workman, J.L. (2007). The Role of Chromatin during Transcription. Cell *128*, 707-719. 10.1016/j.cell.2007.01.015.
3. Ma, S., Zhang, B., LaFave, L.M., Earl, A.S., Chiang, Z., Hu, Y., Ding, J., Brack, A., Kartha, V.K., Tay, T., et al. (2020). Chromatin Potential Identified by Shared Single-Cell Profiling of RNA and Chromatin. Cell *183*, 1103-1116.e1120. 10.1016/j.cell.2020.09.056.
4. Chen, S., Lake, B.B., and Zhang, K. (2019). High-throughput sequencing of the transcriptome and chromatin accessibility in the same cell. Nature Biotechnology *37*, 1452-1457. 10.1038/s41587-019-0290-0.
5. Liu, L., Liu, C., Quintero, A., Wu, L., Yuan, Y., Wang, M., Cheng, M., Leng, L., Xu, L., Dong, G., et al. (2019). Deconvolution of single-cell multi-omics layers reveals regulatory heterogeneity. Nature Communications *10*, 470. 10.1038/s41467-018-08205-7.
6. Zhu, C., Preissl, S., and Ren, B. (2020). Single-cell multimodal omics: the power of many. Nature Methods *17*, 11-14. 10.1038/s41592-019-0691-5.
7. Hao, Y., Hao, S., Andersen-Nissen, E., Mauck, W.M., III, Zheng, S., Butler, A., Lee, M.J., Wilk, A.J., Darby, C., Zager, M., et al. (2021). Integrated analysis of multimodal single-cell data. Cell *184*, 3573-3587.e3529. 10.1016/j.cell.2021.04.048.
8. Ashuach, T., Gabitto, M.I., Jordan, M.I., and Yosef, N. (2021). MultiVI: deep generative model for the integration of multi-modal data. bioRxiv, 2021.2008.2020.457057. 10.1101/2021.08.20.457057.
9. Wu, K.E., Yost, K.E., Chang, H.Y., and Zou, J. (2021). BABEL enables cross-modality translation between multiomic profiles at single-cell resolution. Proceedings of the National Academy of Sciences *118*, e2023070118. 10.1073/pnas.2023070118.
10. Cao, Z.-J., and Gao, G. (2022). Multi-omics single-cell data integration and regulatory inference with graph-linked embedding. Nature Biotechnology *40*, 1458-1466. 10.1038/s41587-022-01284-4.
11. Lynch, A.W., Theodoris, C.V., Long, H.W., Brown, M., Liu, X.S., and Meyer, C.A. (2022). MIRA: joint regulatory modeling of multimodal expression and chromatin accessibility in single cells. Nature Methods *19*, 1097-1108. 10.1038/s41592-022-01595-z.
12. Dou, Z.-Y., Xu, Y., Gan, Z., Wang, J., Wang, S., Wang, L., Zhu, C., Zhang, P., Yuan, L., and Peng, N. An empirical study of training end-to-end vision-and-language transformers. 2022. pp. 18166-18176.
13. Radford, A., Kim, J.W., Hallacy, C., Ramesh, A., Goh, G., Agarwal, S., Sastry, G., Askell, A., Mishkin, P., and Clark, J. Learning transferable visual models from natural language supervision. 2021. (PMLR), pp. 8748-8763.
14. Li, J., Selvaraju, R., Gotmare, A., Joty, S., Xiong, C., and Hoi, S.C.H. (2021). Align before fuse: Vision and language representation learning with momentum distillation. Advances in neural information processing systems *34*, 9694-9705.
15. Yang, M., Yang, Y., Xie, C., Ni, M., Liu, J., Yang, H., Mu, F., and Wang, J. (2022). Contrastive

learning enables rapid mapping to multimodal single-cell atlas of multimillion scale. Nature Machine Intelligence *4*, 696-709. 10.1038/s42256-022-00518-z.
16. Vaswani, A., Shazeer, N., Parmar, N., Uszkoreit, J., Jones, L., Gomez, A.N., Kaiser, Ł., and Polosukhin, I. (2017). Attention is all you need. Advances in neural information processing systems *30*.
17. Wang, T., and Isola, P. Understanding contrastive representation learning through alignment and uniformity on the hypersphere. 2020. (PMLR), pp. 9929-9939.
18. Luecken, M.D., Büttner, M., Chaichoompu, K., Danese, A., Interlandi, M., Müller, M.F., Strobl, D.C., Zappia, L., Dugas, M., and Colomé-Tatché, M. (2022). Benchmarking atlas-level data integration in single-cell genomics. Nature methods *19*, 41-50.
19. Luecken, M.D., Burkhardt, D.B., Cannoodt, R., Lance, C., Agrawal, A., Aliee, H., Chen, A.T., Deconinck, L., Detweiler, A.M., and Granados, A.A. A sandbox for prediction and integration of dna, rna, and proteins in single cells. 2021.
20. Lin, Y., Wu, T.-Y., Wan, S., Yang, J.Y.H., Wong, W.H., and Wang, Y.X.R. (2022). scJoint integrates atlas-scale single-cell RNA-seq and ATAC-seq data with transfer learning. Nature Biotechnology *40*, 703-710. 10.1038/s41587-021-01161-6.
21. Lotfollahi, M., Litinetskaya, A., and Theis, F.J. (2022). Multigrate: single-cell multi-omic data integration. BioRxiv, 2022-2003.
22. Hao, Y., Stuart, T., Kowalski, M.H., Choudhary, S., Hoffman, P., Hartman, A., Srivastava, A., Molla, G., Madad, S., and Fernandez-Granda, C. (2023). Dictionary learning for integrative, multimodal and scalable single-cell analysis. Nature Biotechnology, 1-12.
23. Zhang, R., Meng-Papaxanthos, L., Vert, J.-p., and Noble, W.S. (2022). Multimodal Single-Cell Translation and Alignment with Semi-Supervised Learning. Journal of Computational Biology *29*, 1198-1212.
24. Trevino, A.E., Müller, F., Andersen, J., Sundaram, L., Kathiria, A., Shcherbina, A., Farh, K., Chang, H.Y., Pașca, A.M., Kundaje, A., et al. (2021). Chromatin and gene-regulatory dynamics of the developing human cerebral cortex at single-cell resolution. Cell *184*, 5053-5069.e5023. 10.1016/j.cell.2021.07.039.
25. Dekker, J., and Heard, E. (2015). Structural and functional diversity of Topologically Associating Domains. FEBS Letters *589*, 2877-2884. https://doi.org/10.1016/j.febslet.2015.08.044.
26. Dixon, J.R., Selvaraj, S., Yue, F., Kim, A., Li, Y., Shen, Y., Hu, M., Liu, J.S., and Ren, B. (2012). Topological domains in mammalian genomes identified by analysis of chromatin interactions. Nature *485*, 376-380. 10.1038/nature11082.
27. Javierre, B.M., Burren, O.S., Wilder, S.P., Kreuzhuber, R., Hill, S.M., Sewitz, S., Cairns, J., Wingett, S.W., Várnai, C., Thiecke, M.J., et al. (2016). Lineage-Specific Genome Architecture Links Enhancers and Non-coding Disease Variants to Target Gene Promoters. Cell *167*, 1369-1384.e1319. 10.1016/j.cell.2016.09.037.
28. Krivega, I., and Dean, A. (2012). Enhancer and promoter interactions-long distance calls. Current Opinion in Genetics & Development *22*, 79-85. 10.1016/j.gde.2011.11.001.
29. van Arensbergen, J., van Steensel, B., and Bussemaker, H.J. (2014). In search of the determinants of enhancer-promoter interaction specificity. Trends in Cell Biology *24*, 695-702. 10.1016/j.tcb.2014.07.004.
30. Pliner, H.A., Packer, J.S., McFaline-Figueroa, J.L., Cusanovich, D.A., Daza, R.M., Aghamirzaie,

D., Srivatsan, S., Qiu, X., Jackson, D., Minkina, A., et al. (2018). Cicero Predicts cis-Regulatory DNA Interactions from Single-Cell Chromatin Accessibility Data. Molecular Cell *71*, 858-871.e858. 10.1016/j.molcel.2018.06.044.

31. Li, C., Virgilio, M.C., Collins, K.L., and Welch, J.D. (2022). Multi-omic single-cell velocity models epigenome–transcriptome interactions and improves cell fate prediction. Nature Biotechnology. 10.1038/s41587-022-01476-y.
32. Brownell, I. Guevara e, Bai CB, Loomis CA, Joyner AL (2011) Nerve-derived sonic hedgehog defines a niche for hair follicle stem cells capable of becoming epidermal stem cells. Cell Stem Cell *8*, 552-565.
33. Mill, P., Mo, R., Fu, H., Grachtchouk, M., Kim, P.C.W., Dlugosz, A.A., and Hui, C.-c. (2003). Sonic hedgehog-dependent activation of Gli2 is essential for embryonic hair follicle development. Genes & development *17*, 282-294.
34. Matissek, S.J., and Elsawa, S.F. (2020). GLI3: a mediator of genetic diseases, development and cancer. Cell Communication and Signaling *18*, 1-20.
35. Chandramouli, A., Hatsell, S.J., Pinderhughes, A., Koetz, L., and Cowin, P. (2013). Gli activity is critical at multiple stages of embryonic mammary and nipple development. PLoS One *8*, e79845.
36. Aibar, S., González-Blas, C.B., Moerman, T., Huynh-Thu, V.A., Imrichova, H., Hulselmans, G., Rambow, F., Marine, J.-C., Geurts, P., Aerts, J., et al. (2017). SCENIC: single-cell regulatory network inference and clustering. Nature Methods *14*, 1083-1086. 10.1038/nmeth.4463.
37. Futschik, M.E., and Carlisle, B. (2005). NOISE-ROBUST SOFT CLUSTERING OF GENE EXPRESSION TIME-COURSE DATA. Journal of Bioinformatics and Computational Biology *03*, 965-988. 10.1142/S0219720005001375.
38. Kumar, L., and Futschik, M.E. (2007). Mfuzz: a software package for soft clustering of microarray data. Bioinformation *2*, 5.
39. Cui, C.-Y., Yin, M., Sima, J., Childress, V., Michel, M., Piao, Y., and Schlessinger, D. (2014). Involvement of Wnt, Eda and Shh at defined stages of sweat gland development. Development *141*, 3752-3760.
40. Avilés, E.C., Wilson, N.H., and Stoeckli, E.T. (2013). Sonic hedgehog and Wnt: antagonists in morphogenesis but collaborators in axon guidance. Frontiers in cellular neuroscience *7*, 86.
41. Dave, R.K., Ellis, T., Toumpas, M.C., Robson, J.P., Julian, E., Adolphe, C., Bartlett, P.F., Cooper, H.M., Reynolds, B.A., and Wainwright, B.J. (2011). Sonic hedgehog and notch signaling can cooperate to regulate neurogenic divisions of neocortical progenitors. PloS one *6*, e14680.
42. Adam, R.C., Yang, H., Ge, Y., Lien, W.-H., Wang, P., Zhao, Y., Polak, L., Levorse, J., Baksh, S.C., and Zheng, D. (2018). Temporal layering of signaling effectors drives chromatin remodeling during hair follicle stem cell lineage progression. Cell stem cell *22*, 398-413.
43. Hu, X.-M., Li, Z.-X., Zhang, D.-Y., Yang, Y.-C., Fu, S.-a., Zhang, Z.-Q., Yang, R.-H., and Xiong, K. (2021). A systematic summary of survival and death signalling during the life of hair follicle stem cells. Stem cell research & therapy *12*, 1-29.
44. (!!! INVALID CITATION !!! 37,42-44).
45. Lefebvre, V. (2019). Roles and regulation of SOX transcription factors in skeletogenesis.

Current topics in developmental biology *133*, 171-193.

46. Tan, Z., Niu, B., Tsang, K.Y., Melhado, I.G., Ohba, S., He, X., Huang, Y., Wang, C., McMahon, A.P., and Jauch, R. (2018). Synergistic co-regulation and competition by a SOX9-GLI-FOXA phasic transcriptional network coordinate chondrocyte differentiation transitions. PLoS genetics *14*, e1007346.
47. Liu, C.-F., and Lefebvre, V. (2015). The transcription factors SOX9 and SOX5/SOX6 cooperate genome-wide through super-enhancers to drive chondrogenesis. Nucleic acids research *43*, 8183-8203.
48. Bunt, J., Osinski, J.M., Lim, J.W.C., Vidovic, D., Ye, Y., Zalucki, O., O'Connor, T.R., Harris, L., Gronostajski, R.M., and Richards, L.J. (2017). Combined allelic dosage of Nfia and Nfib regulates cortical development. Brain and Neuroscience Advances *1*, 2398212817739433.
49. Pjanic, M., Pjanic, P., Schmid, C., Ambrosini, G., Gaussin, A., Plasari, G., Mazza, C., Bucher, P., and Mermod, N. (2011). Nuclear factor I revealed as family of promoter binding transcription activators. BMC genomics *12*, 1-10.
50. Fraser, J., Essebier, A., Brown, A.S., Davila, R.A., Harkins, D., Zalucki, O., Shapiro, L.P., Penzes, P., Wainwright, B.J., and Scott, M.P. (2020). Common regulatory targets of NFIA, NFIX and NFIB during postnatal cerebellar development. The Cerebellum *19*, 89-101.
51. Moser, M., Binder, O., Wu, Y., Aitsebaomo, J., Ren, R., Bode, C., Bautch, V.L., Conlon, F.L., and Patterson, C. (2003). BMPER, a novel endothelial cell precursor-derived protein, antagonizes bone morphogenetic protein signaling and endothelial cell differentiation. Molecular and cellular biology *23*, 5664-5679.
52. McGarvey, A.C., Rybtsov, S., Souilhol, C., Tamagno, S., Rice, R., Hills, D., Godwin, D., Rice, D., Tomlinson, S.R., and Medvinsky, A. (2017). A molecular roadmap of the AGM region reveals BMPER as a novel regulator of HSC maturation. Journal of Experimental Medicine *214*, 3731-3751.
53. Kuschel, S., Rüther, U., and Theil, T. (2003). A disrupted balance between Bmp/Wnt and Fgf signaling underlies the ventralization of the Gli3 mutant telencephalon. Developmental biology *260*, 484-495.
54. Zhao, H., Zhou, W., Yao, Z., Wan, Y., Cao, J., Zhang, L., Zhao, J., Li, H., Zhou, R., and Li, B. (2015). Foxp1/2/4 regulate endochondral ossification as a suppresser complex. Developmental biology *398*, 242-254.
55. Gan, X., Wang, J., Wang, C., Sommer, E., Kozasa, T., Srinivasula, S., Alessi, D., Offermanns, S., Simon, M.I., and Wu, D. (2012). PRR5L degradation promotes mTORC2-mediated PKC-δ phosphorylation and cell migration downstream of Gα12. Nature cell biology *14*, 686-696.
56. Riobo, N.A., Haines, G.M., and Emerson Jr, C.P. (2006). Protein kinase C-δ and mitogen-activated protein/extracellular signal–regulated kinase-1 control GLI activation in Hedgehog signaling. Cancer research *66*, 839-845.
57. Maiti, S., Mondal, S., Satyavarapu, E.M., and Mandal, C. (2017). mTORC2 regulates hedgehog pathway activity by promoting stability to Gli2 protein and its nuclear translocation. Cell Death & Disease *8*, e2926-e2926.
58. Khajavi, M., Zhou, Y., Schiffer, A.J., Bazinet, L., Birsner, A.E., Zon, L., and D'Amato, R.J. (2021). Identification of Basp1 as a novel angiogenesis-regulating gene by multi-model system studies. The FASEB Journal *35*, e21404.

59. Gao, Y., Banik, D.D., Muna, M.M., Roberts, S.G.E., and Medler, K.F. (2019). The WT1–BASP1 complex is required to maintain the differentiated state of taste receptor cells. Life science alliance *2*.
60. Wu, J., Zhu, P., Lu, T., Du, Y., Wang, Y., He, L., Ye, B., Liu, B., Yang, L., and Wang, J. (2019). The long non-coding RNA LncHDAC2 drives the self-renewal of liver cancer stem cells via activation of Hedgehog signaling. Journal of hepatology *70*, 918-929.
61. Peñagarikano, O., Abrahams, Brett S., Herman, Edward I., Winden, Kellen D., Gdalyahu, A., Dong, H., Sonnenblick, Lisa I., Gruver, R., Almajano, J., Bragin, A., et al. (2011). Absence of CNTNAP2 Leads to Epilepsy, Neuronal Migration Abnormalities, and Core Autism-Related Deficits. Cell *147*, 235-246. https://doi.org/10.1016/j.cell.2011.08.040.
62. Junge, H.J., Yung, A.R., Goodrich, L.V., and Chen, Z. (2016). Netrin1/DCC signaling promotes neuronal migration in the dorsal spinal cord. Neural Development *11*, 19. 10.1186/s13064-016-0074-x.
63. Bando, Y., Ishibashi, M., Yamagishi, S., Fukuda, A., and Sato, K. (2022). Orchestration of Ion Channels and Transporters in Neocortical Development and Neurological Disorders. Frontiers in Neuroscience *16*.
64. Wen, L., Li, G., Huang, T., Geng, W., Pei, H., Yang, J., Zhu, M., Zhang, P., Hou, R., Tian, G., et al. (2022). Single-cell technologies: From research to application. The Innovation *3*, 100342. https://doi.org/10.1016/j.xinn.2022.100342.
65. Saelens, W., Cannoodt, R., Todorov, H., and Saeys, Y. (2019). A comparison of single-cell trajectory inference methods. Nature Biotechnology *37*, 547-554. 10.1038/s41587-019-0071-9.
66. Wolf, F.A., Angerer, P., and Theis, F.J. (2018). SCANPY: large-scale single-cell gene expression data analysis. Genome Biology *19*, 15. 10.1186/s13059-017-1382-0.
67. Yang, Z., Yang, D., Dyer, C., He, X., Smola, A., and Hovy, E. Hierarchical attention networks for document classification. 2016. pp. 1480-1489.
68. Xu, C., Lopez, R., Mehlman, E., Regier, J., Jordan, M.I., and Yosef, N. (2021). Probabilistic harmonization and annotation of single-cell transcriptomics data with deep generative models. Molecular systems biology *17*, e9620.
69. Butler, A., Hoffman, P., Smibert, P., Papalexi, E., and Satija, R. (2018). Integrating single-cell transcriptomic data across different conditions, technologies, and species. Nature biotechnology *36*, 411-420.
70. Singh, R., Demetci, P., Bonora, G., Ramani, V., Lee, C., Fang, H., Duan, Z., Deng, X., Shendure, J., and Disteche, C. Unsupervised manifold alignment for single-cell multi-omics data. 2020. pp. 1-10.
71. Schoenfelder, S., Javierre, B.-M., Furlan-Magaril, M., Wingett, S.W., and Fraser, P. (2018). Promoter capture Hi-C: high-resolution, genome-wide profiling of promoter interactions. JoVE (Journal of Visualized Experiments), e57320.
72. Hinrichs, A.S., Karolchik, D., Baertsch, R., Barber, G.P., Bejerano, G., Clawson, H., Diekhans, M., Furey, T.S., Harte, R.A., Hsu, F., et al. (2006). The UCSC Genome Browser Database: update 2006. Nucleic Acids Research *34*, D590-D598. 10.1093/nar/gkj144.
73. Subramanian, A., Tamayo, P., Mootha, V.K., Mukherjee, S., Ebert, B.L., Gillette, M.A., Paulovich, A., Pomeroy, S.L., Golub, T.R., Lander, E.S., and Mesirov, J.P. (2005). Gene set enrichment analysis: A knowledge-based approach for interpreting genome-wide

expression profiles. Proceedings of the National Academy of Sciences *102*, 15545-15550. 10.1073/pnas.0506580102.

74. Wu, T., Hu, E., Xu, S., Chen, M., Guo, P., Dai, Z., Feng, T., Zhou, L., Tang, W., Zhan, L., et al. (2021). clusterProfiler 4.0: A universal enrichment tool for interpreting omics data. The Innovation *2*. 10.1016/j.xinn.2021.100141.

75. Bravo González-Blas, C., Minnoye, L., Papasokrati, D., Aibar, S., Hulselmans, G., Christiaens, V., Davie, K., Wouters, J., and Aerts, S. (2019). cisTopic: cis-regulatory topic modeling on single-cell ATAC-seq data. Nature Methods *16*, 397-400. 10.1038/s41592-019-0367-1.

76. Setty, M., Kiseliovas, V., Levine, J., Gayoso, A., Mazutis, L., and Pe'er, D. (2019). Characterization of cell fate probabilities in single-cell data with Palantir. Nature Biotechnology *37*, 451-460. 10.1038/s41587-019-0068-4.

77. Castro-Mondragon, J.A., Riudavets-Puig, R., Rauluseviciute, I., Berhanu Lemma, R., Turchi, L., Blanc-Mathieu, R., Lucas, J., Boddie, P., Khan, A., Manosalva Pérez, N., et al. (2022). JASPAR 2022: the 9th release of the open-access database of transcription factor binding profiles. Nucleic Acids Research *50*, D165-D173. 10.1093/nar/gkab1113.

78. Schep, A.N., Wu, B., Buenrostro, J.D., and Greenleaf, W.J. (2017). chromVAR: inferring transcription-factor-associated accessibility from single-cell epigenomic data. Nature Methods *14*, 975-978. 10.1038/nmeth.4401.

79. Cao, K., Gong, Q., Hong, Y., and Wan, L. (2022). A unified computational framework for single-cell data integration with optimal transport. Nature Communications *13*, 1-15.

80. Gong, B., Zhou, Y., and Purdom, E. (2021). Cobolt: Joint analysis of multimodal single-cell sequencing data. bioRxiv, 2021.2004.2003.438329. 10.1101/2021.04.03.438329.

81. Liu, C., Wang, L., and Liu, Z. (2022). Single-cell Multi-omics Integration for Unpaired Data by a Siamese Network with Graph-based Contrastive Loss. bioRxiv, 2022.2006.2007.495170. 10.1101/2022.06.07.495170.

82. Xu, Y., Begoli, E., and McCord, R.P. (2021). sciCAN: Single-cell chromatin accessibility and gene expression data integration via Cycle-consistent Adversarial Network. bioRxiv.

83. Stuart, T., Butler, A., Hoffman, P., Hafemeister, C., Papalexi, E., Mauck, W.M., Hao, Y., Stoeckius, M., Smibert, P., and Satija, R. (2019). Comprehensive Integration of Single-Cell Data. Cell *177*, 1888-1902.e1821. 10.1016/j.cell.2019.05.031.

Figures

Figure 1. **The overall framework of Attune. (a)** Schematic of Attune and downstream tasks. Each cell has two modalities from scRNA-seq and scATAC-seq (the color indicates different cells and the shape indicate different modalities) and each cell's two modalities are positive pairs to be pulled together while other cells are pushed away via contrastive learning. Learned cell embeddings after integration are fed into downstream tasks including cross-modality prediction, differentiation analysis and inference of gene-peak interaction.

(b) Overview of Attune model and transformer-based decoder architecture. Attune consists of two asymmetric teacher-student networks. We propose cross-modal contrastive loss to integrate the cell embeddings of matched RNA-ATAC pairs into a common space. We finetune Attune via a transformer-based decoder for recovering regulatory events, which is used to learn multimodal interactions between peak and gene. We extract gene embedding and peak embedding from teacher networks and the green block denotes each gene or peak and the yellow block denotes CLS token. A CLS contrastive loss is applied to shorten the distance between the matched RNA-ATAC pairs globally. The Cosine similarity between cells in-batch is obtained by CLS contrastive learning and the cells pair with highest similarity is taken as positive pairs (matched) and cells with low similarity is randomly selected as negative pairs (unmatched). Then input the positive pairs and negative pairs into transformer in turn. Concatenate the output of the positive pairs and negative pairs in the transformer and feed it into dense layer followed by softmax for two-class prediction (positive pairs or negative pairs). Then calculate the cross-entropy with one-hot label (1 for positive pairs) for RNA-ATAC modality matching.

Figure 2. **Comprehensive benchmarks of integration and cross-modal prediction performance. (a)** Biology conservation score versus omics integration score for different methods (repeated five times with different random seeds) on 10x Multiome dataset (n=11,909 cells, left) and SHARE-seq dataset (n=32,231 cells, right). **(b)** Comparison of overall integration score on the 10x Multiome dataset and SHARE-seq dataset on the left and comparison of FOSCTTM on the right. Error bars represent the 95% confidence interval. **(c)** Alignment–uniformity plot for Attune, GLUE, LIGER, Seurat, Cobolt, MinNet, scJoint and MultiVI on 10x Multiome dataset. There are five replicates (represented by dots) for each method. **(d)** Integration performance of Attune under different numbers of gene and peak settings on 10x Multiome dataset. The size of the dot indicates the number of peaks, and the color indicates the number of genes. **(e)** Ablation study of Attune feature

embeddings under different numbers of dimension settings. The bar shows the overall integration score for 10x Multiome datasets (repeated five times with different random seeds). Error bars represent the 95% confidence interval. **(f)** Comparing the performance of modality predictions (fivefold cross-validation) in terms of gene-wise Pearson correlation coefficient, gene-wise Spearman correlation coefficient and RMSE on the 10x Multiome dataset. Comparison is made with Babel and Polarbear. **(g)** Performance comparison of modality prediction against the top winners in the NeurIPS 2021 competition. **(h)** Ablation study on contrast between different modalities or networks; RtAs_RsAt represents one set of contrast learning between RNA teacher network and ATAC student network and another set of contrast learning between RNA student network and ATAC teacher network. The violin plots show the modality prediction performance for 10x Multiome datasets (fivefold cross-validation). In the boxplots included in violin plots: center line, median; box, interquartile range (IQR; the range between the 25th and 75th percentiles); whiskers, 1.5 × IQR; dots, outliers.

Figure 3. **Regulatory interaction analysis of Attune on 10x Multiome dataset. (a)** Distribution of distance between peaks and promoters in the PCHi-C dataset. **(b)** Comparison of AUROC among six methods, including Attune + Transformer, PCA + Transformer, GLUE, Spearman, LASSO, and Cicero, on the PCHi-C dataset. The threshold for peak-promoter distance is set to 1200kb. **(c)** Attention weight is in descending order and the cutoff of the top 10% is labeled on the 10x Multiome dataset. **(d)** Differential expression of 466 DPAGs in the 10x Multiome dataset, with some top DEGs highlighted. **(e)** Enrichment analysis of biological process within differentially expressed genes. **(f)** Heatmap of gene expression or accessibility with cell types. Each row represents a gene-peak pair extracted by attention weight. **(g)** UMAP visualization of prediction, measurement, and the residual value of Gli3 gene on SHARE-seq dataset. **(h)** Trend of Gli3's residual from SHARE-seq dataset along the pseudotime. The maximum residual value is highlighted. **(i)** Composition of cell types in ten pseudo samples.

Figure 4. **Regulatory mechanism of hair follicle maturation. (a)** Four clusters of Gli3 peaks by soft cluster. **(b)** Link plot of peaks with high attention weight to Gli3. Peaks of cluster 2/3 are colored. **(c)** Chromatin-accessible state of 12 peaks within cluster 2 (top) or cluster 3 (bottom). **(d)** Spearman's correlation between peaks in cluster 2 or cluster 3 and genes. **(e)** Similar transit pattern between peaks of two clusters and genes. Each row shows the normalized expression or accessibility score of a gene or peak.

Figure 5. **State transition of neonatal neurons in the human cortex. (a)** Blueprint of Hh-centric multiple pathways involved in hair follicle development. Genes associated with peaks in cluster 2 or cluster 3 are highlighted in red. The background of pathways such as NF, Wnt, Bmp and Hh are colored. **(b)** Cosine similarity between each cell from two modalities on the human cortex dataset is calculated. Cells are arranged chronologically. **(c)** UMAP visualization of cosine distance from matched cells between RNA and ATAC modalities.

 Click here to access/download;Table;Supplementary_Table.xlsx

dataset	cells (filter)	organ/tissue	download
PBMC 10k	11,909	PBMC	https://support.10xgenomics.com/single-cell-multiome-atac-gex/datasets/1.0.0/pbmc_granulocyte_sorted_10k
SHARE-seq	32,231	skin late anagen （mouse）	https://www.ncbi.nlm.nih.gov/geo/query/acc.cgi?acc=GSE140203
NeurIPS	42,492	bone marrow	GSE194122
Greenleaf 2021	4,733	brain cortex	GSE162170

article

Chromatin potential identified by shared single-cell profiling of RNA and chromatin.

A sandbox for prediction and integration of DNA, RNA, and proteins in single cells

Chromatin and gene-regulatory dynamics of the developing human cerebral cortex at single-cell resolution

fold	algorithm	dataset	mean_average_precision	Average silhouette width (batch)
0	Attune	10X Multiomis (11909 cells)	0.715699252	0.950580955
1	Attune	10X Multiomis (11909 cells)	0.728872708	0.953992724
2	Attune	10X Multiomis (11909 cells)	0.739889412	0.94317323
3	Attune	10X Multiomis (11909 cells)	0.730246558	0.952854753
4	Attune	10X Multiomis (11909 cells)	0.732744958	0.957969844
0	GLUE	10X Multiomis (11909 cells)	0.710354404	0.854486346
1	GLUE	10X Multiomis (11909 cells)	0.706715393	0.863063991
2	GLUE	10X Multiomis (11909 cells)	0.704176993	0.826774478
3	GLUE	10X Multiomis (11909 cells)	0.70197674	0.830168903
4	GLUE	10X Multiomis (11909 cells)	0.705537845	0.859724343
0	uniPort	10X Multiomis (11909 cells)	0.655791917	0.66494894
1	uniPort	10X Multiomis (11909 cells)	0.614235679	0.51865828
2	uniPort	10X Multiomis (11909 cells)	0.641337587	0.595125377
3	uniPort	10X Multiomis (11909 cells)	0.591730644	0.48354286
4	uniPort	10X Multiomis (11909 cells)	0.627806753	0.473863691
0	Seurat	10X Multiomis (11909 cells)	0.775889	0.612347
1	Seurat	10X Multiomis (11909 cells)	0.775685	0.612226
2	Seurat	10X Multiomis (11909 cells)	0.775918	0.612153
3	Seurat	10X Multiomis (11909 cells)	0.775944	0.612453
4	Seurat	10X Multiomis (11909 cells)	0.775831	0.611914
0	Cobolt	10X Multiomis (11909 cells)	0.698212306	0.872220993
1	Cobolt	10X Multiomis (11909 cells)	0.70268918	0.851240635
2	Cobolt	10X Multiomis (11909 cells)	0.698915555	0.8448264
3	Cobolt	10X Multiomis (11909 cells)	0.700310116	0.868741214
4	Cobolt	10X Multiomis (11909 cells)	0.699390792	0.853699565
0	MinNet	10X Multiomis (11909 cells)	0.654403648	0.844810367
1	MinNet	10X Multiomis (11909 cells)	0.653700792	0.835619565
2	MinNet	10X Multiomis (11909 cells)	0.638721231	0.827216565
3	MinNet	10X Multiomis (11909 cells)	0.644724416	0.832748893
4	MinNet	10X Multiomis (11909 cells)	0.651204449	0.82571957
0	scJoint	10X Multiomis (11909 cells)	0.716132315	0.836206794
1	scJoint	10X Multiomis (11909 cells)	0.717660907	0.839528084
2	scJoint	10X Multiomis (11909 cells)	0.713938056	0.841825604
3	scJoint	10X Multiomis (11909 cells)	0.711823744	0.847566426
4	scJoint	10X Multiomis (11909 cells)	0.714244158	0.836271644
0	MultiVI	10X Multiomis (11909 cells)	0.734876481	0.971069694
1	MultiVI	10X Multiomis (11909 cells)	0.734075594	0.97470057
2	MultiVI	10X Multiomis (11909 cells)	0.745116985	0.978001595
3	MultiVI	10X Multiomis (11909 cells)	0.742567282	0.971963108
4	MultiVI	10X Multiomis (11909 cells)	0.743889789	0.96882391
0	sciCAN	10X Multiomis (11909 cells)	0.733585615	0.516801715
1	sciCAN	10X Multiomis (11909 cells)	0.739636665	0.511185408
2	sciCAN	10X Multiomis (11909 cells)	0.743406546	0.529781699
3	sciCAN	10X Multiomis (11909 cells)	0.743672036	0.524292469
4	sciCAN	10X Multiomis (11909 cells)	0.764743391	0.606484234

Average silhouette width (cell type)	graph_connectivity	neighbor_conservation	seurat_alignment_score
0.539540384	0.93446245	0.211078	0.914609503
0.54469981	0.936969125	0.212365	0.97522516
0.552365862	0.92812328	0.213556	0.891521861
0.544816639	0.93546325	0.217124	0.966178733
0.545132156	0.935456733	0.218323	0.947538911
0.56460537	0.926826584	0.173642465	0.766396222
0.558356252	0.932757271	0.174523653	0.763897935
0.554822516	0.915577341	0.169343927	0.717839978
0.557212908	0.923921817	0.170817504	0.746571162
0.559735034	0.928725161	0.17160848	0.783688772
0.526763016	0.804729593	0.223810468	0.626941279
0.475881124	0.621422855	0.222932006	0.493674017
0.522023192	0.715059231	0.223440557	0.575092208
0.473701473	0.573031559	0.225001321	0.535373995
0.490237314	0.60031135	0.223553277	0.458125731
0.565527	0.957658	0.20085847	0.928327
0.565448	0.95619	0.200844264	0.928272
0.565573	0.957182	0.200970694	0.928055
0.565643	0.956325	0.200790573	0.928503
0.565535	0.956502	0.200929377	0.928231
0.563039735	0.946855931	0.169000428	0.891881079
0.565973394	0.951334033	0.171740724	0.869537974
0.557477526	0.945634405	0.171857522	0.851662573
0.564797401	0.950597082	0.17257875	0.897561762
0.566664159	0.950623258	0.170370041	0.878682954
0.558792822	0.928066292	0.133337891	0.584315866
0.577794159	0.908663258	0.1380041	0.579315295
0.567624384	0.901337033	0.131757533	0.577474435
0.567895506	0.912337506	0.133877643	0.575705558
0.566080001	0.926795931	0.132857555	0.570586967
0.541469961	0.872481027	0.138211068	0.71397065
0.543057568	0.870456361	0.138444313	0.750587967
0.542118408	0.882899837	0.139917105	0.713695454
0.540100019	0.896691315	0.13746356	0.737376435
0.537405796	0.879019794	0.137775619	0.635700985
0.525465	0.960912436	0.163012666	0.993482791
0.515694387	0.960833417	0.163995178	0.996274973
0.532789897	0.962648026	0.173104753	0.996089392
0.528379327	0.960232133	0.164421897	0.995840304
0.527599966	0.962025928	0.165446728	0.993841604
0.514762215	0.814283891	0.232345794	0.044732781
0.512896264	0.840500268	0.238369645	0.038943077
0.517187703	0.796689493	0.240852328	0.043311993
0.512317998	0.816483417	0.241523535	0.046178619
0.515947238	0.890165428	0.247169005	0.114583914

foscttm	biology conservation	omics mixing	Overall integration score
0.017866916	0.664249645	0.929320572	0.770278016
0.023431551	0.708325997	0.954826744	0.806926295
0.011923121	0.756249343	0.910960311	0.81813373
0.024615942	0.724931114	0.949636109	0.814813112
0.010526495	0.733925301	0.946522406	0.818964143
0.056320952	0.626721189	0.807644142	0.69909037
0.061847301	0.602670085	0.817519711	0.688609935
0.069743357	0.571800697	0.762790203	0.648196499
0.063453659	0.579730051	0.78217754	0.660709046
0.072718289	0.596534905	0.818752883	0.685422096
0.224017801	0.551705051	0.529307068	0.542745858
0.527558624	0.311034241	0.229351481	0.278361137
0.306075636	0.509303399	0.388369991	0.460930036
0.591969929	0.26930826	0.179252066	0.233285782
0.534543792	0.383357869	0.169294195	0.297732399
0.091492	0.82686281	0.730303124	0.788238936
0.09162	0.826199664	0.728948033	0.787299012
0.091496	0.827386717	0.729672907	0.788301193
0.091499	0.827137695	0.729294055	0.788000239
0.091502	0.826988273	0.728994394	0.787790722
0.018383992	0.586329311	0.880198714	0.703877072
0.019334743	0.611739145	0.862378173	0.711994756
0.018474227	0.578042028	0.847036807	0.685639939
0.017689055	0.606088788	0.883076572	0.716883902
0.018998267	0.604023915	0.866580096	0.709046388
0.084794144	0.390456804	0.73890851	0.529837487
0.089794144	0.463509506	0.714490338	0.563901839
0.087411849	0.385796201	0.702025455	0.512287903
0.084091767	0.403650486	0.714478864	0.527981837
0.085636236	0.406616102	0.720418664	0.532137127
0.086131909	0.460756822	0.730808824	0.568777623
0.0857538	0.469280417	0.744022438	0.579177225
0.086300949	0.463790226	0.743341864	0.575610881
0.085910762	0.446414584	0.767182336	0.574721685
0.085892136	0.443067968	0.709193184	0.549518054
0.003085138	0.515054429	0.992959583	0.706216491
0.003212105	0.485154751	0.996264905	0.689598813
0.003031255	0.586189042	0.999935383	0.751687578
0.003517377	0.54237354	0.993789138	0.722939779
0.003246292	0.545230819	0.992552257	0.724159394
0.318637878	0.678694454	0.236807865	0.501939819
0.368938031	0.701066704	0.253507693	0.5220431
0.316206176	0.728801181	0.229842709	0.529217792
0.367186973	0.715626043	0.244145927	0.527033997
0.2615055	0.781681735	0.385346796	0.623147759

fold	algorithm	dataset	mean_average_precision	Average silhouette width (cell type)	Average silhouette width (batch)
0	Attune	SHARE-seq	0.700503821	0.540143441	0.813250124
1	Attune	SHARE-seq	0.701558815	0.540536985	0.812617123
2	Attune	SHARE-seq	0.702686828	0.54095947	0.811814725
3	Attune	SHARE-seq	0.703766793	0.541391455	0.811021447
4	Attune	SHARE-seq	0.704645388	0.541762486	0.810315847
0	GLUE	SHARE-seq	0.561111737	0.50452668	0.816608453
1	GLUE	SHARE-seq	0.55428103	0.503710011	0.807886863
2	GLUE	SHARE-seq	0.558594212	0.504250078	0.805636549
3	GLUE	SHARE-seq	0.558991697	0.504829154	0.809639299
4	GLUE	SHARE-seq	0.559939252	0.505114703	0.817935729
0	uniPort	SHARE-seq	0.163973846	0.476491107	0.86670053
1	uniPort	SHARE-seq	0.161983345	0.478651188	0.867574751
2	uniPort	SHARE-seq	0.16119803	0.478579322	0.870128274
3	uniPort	SHARE-seq	0.161670712	0.477358013	0.861711264
4	uniPort	SHARE-seq	0.162853271	0.476336412	0.865957201
0	Seurat	SHARE-seq	0.492227153	0.505292071	0.694095075
1	Seurat	SHARE-seq	0.493015888	0.505325851	0.693091631
2	Seurat	SHARE-seq	0.492684339	0.505365004	0.693689227
3	Seurat	SHARE-seq	0.492254652	0.505365572	0.694175661
4	Seurat	SHARE-seq	0.492577208	0.50530346	0.692621768
0	Cobolt	SHARE-seq	0.126967955	0.491194342	0.919983923
1	Cobolt	SHARE-seq	0.126630572	0.491923968	0.921099842
2	Cobolt	SHARE-seq	0.125941492	0.491741425	0.920791805
3	Cobolt	SHARE-seq	0.125941492	0.491741425	0.920791805
4	Cobolt	SHARE-seq	0.126299977	0.49143934	0.92005831
0	MinNet	SHARE-seq	0.151289623	0.456259929	0.90310359
1	MinNet	SHARE-seq	0.153622058	0.480556196	0.957647026
2	MinNet	SHARE-seq	0.146684919	0.48934857	0.946947455
3	MinNet	SHARE-seq	0.163738093	0.482384188	0.937516272
4	MinNet	SHARE-seq	0.156352604	0.481624577	0.947921813
0	scJoint	SHARE-seq	0.685662338	0.536288328	0.813352013
1	scJoint	SHARE-seq	0.68383071	0.539928585	0.814432168
2	scJoint	SHARE-seq	0.686678751	0.540302094	0.814481223
3	scJoint	SHARE-seq	0.688810924	0.542210817	0.817528868
4	scJoint	SHARE-seq	0.687938309	0.539540138	0.814856195
0	MultiVI	SHARE-seq	0.658794353	0.487673123	0.815787153
1	MultiVI	SHARE-seq	0.654525256	0.485742581	0.8101808
2	MultiVI	SHARE-seq	0.701351145	0.516192274	0.815275397
3	MultiVI	SHARE-seq	0.70215241	0.51930875	0.810303407
4	MultiVI	SHARE-seq	0.66255027	0.485082293	0.805249579
0	sciCAN	SHARE-seq	0.608825559	0.499169683	0.789557099
1	sciCAN	SHARE-seq	0.606024601	0.497639547	0.800970316
2	sciCAN	SHARE-seq	0.611913511	0.495937721	0.789408922
3	sciCAN	SHARE-seq	0.602698643	0.495377491	0.81079632
4	sciCAN	SHARE-seq	0.553156243	0.495650109	0.819048703

graph_connectivity	neighbor_conservation	seurat_alignment_score	foscttm
0.851057914	0.107465274	0.809841309	0.012032774
0.858072286	0.107401702	0.808762891	0.011458796
0.853274856	0.107341886	0.807434766	0.010927234
0.853709479	0.107281487	0.805726514	0.010450458
0.846011273	0.107205681	0.813027246	0.010052169
0.77934308	0.102771348	0.76200319	0.110629896
0.784497844	0.104017673	0.775601153	0.10787848
0.775116944	0.109277855	0.76426073	0.105463391
0.789208013	0.107120461	0.767416919	0.111203577
0.781341619	0.10182567	0.779142931	0.108680776
0.306936335	0.048544213	0.777082442	0.481773245
0.319933312	0.048394669	0.769063055	0.482565013
0.310575102	0.047930969	0.763526664	0.491753515
0.314703845	0.048651231	0.76472855	0.512390725
0.326495592	0.048647553	0.755073826	0.500785185
0.762716206	0.18019184	0.84922287	0.17888408
0.761397277	0.180204388	0.849131379	0.179018104
0.767198831	0.180213782	0.84856863	0.179248804
0.761368617	0.180105454	0.848205264	0.179726115
0.758282363	0.180244591	0.849925982	0.178902058
0.127547808	0.01040219	0.842686479	0.042933941
0.131189956	0.010395602	0.851623275	0.041899675
0.129715142	0.010365822	0.845319218	0.041560404
0.129715142	0.010365822	0.845319218	0.041560404
0.118823305	0.010391691	0.848359817	0.04226729
0.374789126	0.011971294	0.732720935	0.511673155
0.396704217	0.01234045	0.820827039	0.50308594
0.372757869	0.011601522	0.63334363	0.49541324
0.411413553	0.013664909	0.684402851	0.504290015
0.394515306	0.01335504	0.790599817	0.49254413
0.846413557	0.104223634	0.805877016	0.082324544
0.842151251	0.107842403	0.801615292	0.080487798
0.838807418	0.103144827	0.809110796	0.082459949
0.84241778	0.107822663	0.802074574	0.081040717
0.842293106	0.10519862	0.806159523	0.082841588
0.722442013	0.109550911	0.818209774	0.01414992
0.739414579	0.110623368	0.80957123	0.013241166
0.834679516	0.121670117	0.809675246	0.010299091
0.822829083	0.12040744	0.809653961	0.01101054
0.723285919	0.110914124	0.801641305	0.013412454
0.847031168	0.168237592	0.101129564	0.255789352
0.844101057	0.169397468	0.127078293	0.280872834
0.834934866	0.167460495	0.134085125	0.283475354
0.848637957	0.166470236	0.154072672	0.28333202
0.836458723	0.14675747	0.230099868	0.308249402

biology conservation	omics mixing	Overall integration score
0.846790413	0.796665603	0.826740489
0.848799585	0.798553307	0.828701074
0.850970425	0.794791008	0.828498658
0.853149288	0.793230521	0.829181781
0.854945539	0.79211451	0.829813128
0.619161912	0.747305317	0.670419274
0.614505752	0.744699697	0.66658333
0.629406066	0.732602587	0.670684675
0.627647577	0.745392618	0.674745594
0.618911428	0.757488496	0.674342255
0.175279866	0.60399368	0.346765391
0.182217096	0.607391839	0.352286993
0.180576181	0.603924819	0.349915636
0.177525271	0.595733898	0.344808722
0.174237251	0.602103019	0.345383558
0.734365951	0.624456735	0.690402265
0.734975889	0.62255931	0.690009257
0.734955192	0.625676953	0.691243896
0.734497336	0.623498481	0.690097794
0.734715258	0.620916729	0.689195846
0.136144674	0.619260862	0.329391149
0.138767039	0.62627597	0.333770611
0.13760376	0.622423574	0.331531685
0.13760376	0.622423574	0.331531685
0.136689466	0.617940297	0.329189798
0.017750749	0.660671445	0.274919027
0.11404397	0.778287201	0.379741263
0.142696709	0.670761159	0.353922489
0.129558925	0.699007385	0.357338309
0.121750954	0.751642914	0.373707738
0.816930192	0.792938826	0.807333646
0.837093449	0.790482621	0.818449118
0.83096495	0.792365702	0.815525251
0.848774233	0.794701641	0.827145196
0.832765369	0.79309823	0.816898514
0.623368637	0.745579454	0.672252964
0.615526999	0.742344349	0.666253939
0.782263955	0.791753965	0.786059959
0.792334146	0.780147576	0.787459518
0.618159204	0.725347511	0.661034527
0.754326436	0.450274418	0.632705629
0.749054826	0.474833268	0.639366203
0.742046146	0.459271009	0.628936092
0.732622639	0.501227178	0.640064455
0.666463423	0.539882466	0.61583104

algorithm	align	uniform
Attune	0.053840429	-2.533678532
Attune	0.054178644	-2.495167732
Attune	0.054326255	-2.452186346
Attune	0.054320544	-2.405549765
Attune	0.054261476	-2.352644444
GLUE	3.573233128	-5.954609871
GLUE	3.946593761	-6.55628109
GLUE	3.461676836	-6.115733147
GLUE	3.642579317	-6.456144333
GLUE	3.590011597	-6.041229248
uniPort	4.762979507	-8.403865814
uniPort	4.660891533	-8.538525581
uniPort	4.982663155	-8.381621361
uniPort	4.386288643	-8.483123779
uniPort	4.426053047	-8.514008522
Seurat	63.95615768	-8.562609673
Seurat	64.04239655	-8.508541107
Seurat	63.67448807	-8.522782326
Seurat	63.71570969	-8.532381058
Seurat	63.89039993	-8.564231873
Cobolt	1.185844541	-3.150137424
Cobolt	1.012006164	-3.068443298
Cobolt	0.944249928	-3.079703331
Cobolt	1.10590148	-3.106876612
Cobolt	1.050275445	-3.114895105
MinNet	153.900116	-9.09206295
MinNet	157.8298645	-9.083189672
MinNet	148.1457214	-9.100876554
MinNet	167.6159058	-9.088761345
MinNet	156.007117	-9.088960151
scJoint	0.121046998	-2.188551664
scJoint	0.131353483	-2.22865963
scJoint	0.129614368	-2.257395029
scJoint	0.131866261	-2.260996819
scJoint	0.125707358	-2.232083559
MultiVI	2.085311413	-6.869775772
MultiVI	1.784704924	-7.071695328
MultiVI	1.888138056	-6.712666988
MultiVI	1.434597731	-6.424882889
MultiVI	1.419558167	-6.72172451
sciCAN	0.107095167	-3.126694202
sciCAN	0.110953093	-3.158628941
sciCAN	0.116046928	-3.200929165
sciCAN	0.112709604	-3.21176815
sciCAN	0.131893486	-3.377581596

gene	peak	map	asw	asw_batch	gc	nc
0.5	2	0.55297912	0.505416012	0.922532678	0.907910746	0.119220695
0.5	5	0.564954373	0.510162615	0.928308845	0.916633657	0.127170908
0.5	10	0.573637536	0.513549264	0.945458889	0.922086762	0.137858397
0.5	15	0.580741579	0.519074792	0.931212068	0.911811637	0.133928221
0.5	20	0.594649157	0.519952027	0.94525528	0.942935229	0.147737639
0.5	25	0.589378214	0.516435977	0.945778668	0.927170425	0.144797687
1	2	0.607227507	0.512680615	0.916690826	0.894342173	0.147630032
1	5	0.629911875	0.522512471	0.935263753	0.909976429	0.155035466
1	10	0.652896019	0.531481065	0.927199244	0.909317953	0.161521906
1	15	0.658963593	0.528769257	0.93620187	0.923322014	0.165754021
1	20	0.675513155	0.550075181	0.866093814	0.94909692	0.191593621
1	25	0.662219335	0.530056886	0.946348488	0.926215156	0.163038354
2	2	0.629294213	0.519769244	0.908391774	0.887189205	0.156074104
2	5	0.626565548	0.520938193	0.957562804	0.866754059	0.149798528
2	10	0.676734491	0.538321432	0.958908081	0.927474236	0.171493448
2	15	0.678957948	0.532552987	0.937515736	0.920168378	0.176802678
2	20	0.691058904	0.536792528	0.935329318	0.872546209	0.181450218
2	25	0.731147806	0.55530803	0.939409481	0.930935399	0.202164017
5	2	0.602045098	0.509148316	0.936844051	0.868567645	0.140751509
5	5	0.625763414	0.51071797	0.961326122	0.852928858	0.150958664
5	10	0.65269494	0.521416552	0.934367776	0.871466103	0.173572351
5	15	0.711945458	0.542070076	0.709525704	0.909162493	0.205110316
5	20	0.683364269	0.534626421	0.943213165	0.896540249	0.184672135
5	25	0.676167619	0.511144442	0.703007281	0.895906584	0.180370079
10	2	0.605786139	0.508448499	0.907245278	0.843767727	0.142293303
10	5	0.650672795	0.520709956	0.917206764	0.850656895	0.15220708
10	10	0.664568862	0.540179331	0.925853372	0.863987528	0.161682138
10	15	0.723065489	0.569928356	0.923560143	0.919437954	0.206394215
10	20	0.68234349	0.522498589	0.928131878	0.840340812	0.16837309
10	25	0.704671059	0.540969696	0.814708829	0.90862982	0.183953771
15	2	0.586312186	0.502735721	0.918875694	0.814477481	0.126527346
15	5	0.60405861	0.512228936	0.950597405	0.798196749	0.134117363
15	10	0.685118941	0.536326922	0.933359802	0.845913432	0.163850967
15	15	0.675155713	0.53900544	0.822017848	0.911295317	0.174826901
15	20	0.654827553	0.516617091	0.959806919	0.872826654	0.164589435
15	25	0.695018466	0.553700451	0.8637411	0.926203449	0.191332393

sas	foscttm	biology conservation	omics mixing	Overall integration score
0.847176925	0.063434387	0.340238811	0.962663869	0.589208834
0.89651773	0.043010361	0.341503893	0.981914765	0.597668242
0.82824589	0.03117182	0.334930457	0.941974145	0.577747932
0.843391654	0.027568512	0.347830923	0.95517245	0.590767534
0.804098492	0.020715765	0.342364801	0.940031906	0.581431643
0.866807182	0.01763645	0.339674656	0.959391575	0.587561423
0.876891886	0.04575725	0.357426472	0.973063424	0.603681253
0.908291101	0.027158475	0.359874765	0.977673211	0.606994143
0.885501731	0.021836825	0.374976608	0.974062702	0.614611045
0.878717414	0.019355671	0.370444631	0.969556965	0.610089565
0.616927719	0.025823121	0.370691936	0.817306711	0.549337846
0.93688046	0.019630579	0.368606454	0.987403304	0.616125194
0.806236658	0.040319532	0.370816426	0.945343264	0.600627161
0.980363655	0.019404861	0.340293076	0.945253963	0.582277431
0.947135117	0.016452051	0.369169766	0.981709421	0.614185628
0.889361478	0.019679187	0.375921674	0.97129815	0.614072265
0.908322162	0.016157948	0.38244461	0.960298557	0.613586189
0.968363822	0.035994398	0.383767522	0.971120342	0.61870865
0.922963717	0.022055416	0.347401151	0.965600004	0.594680692
0.979160931	0.014566412	0.335893815	0.942016225	0.578342779
0.864393527	0.020680378	0.362325518	0.941782018	0.594108118
0.302048743	0.052417012	0.399494493	0.618044152	0.486914356
0.963213499	0.01231677	0.363348889	0.962890611	0.603165578
0.315882805	0.095346092	0.385059275	0.639933077	0.487008796
0.802960625	0.028855409	0.361525106	0.926896411	0.587673628
0.89115171	0.028930682	0.377764248	0.959649254	0.610518251
0.934437951	0.01829589	0.380190785	0.965907729	0.614477562
0.888264378	0.015921835	0.409112862	0.981678831	0.638139249
0.950793145	0.014276024	0.369833695	0.943289797	0.599216136
0.567228303	0.067743784	0.403735901	0.799762248	0.56214644
0.860788532	0.025081233	0.351694309	0.931643965	0.583674171
0.978398	0.017056458	0.334822642	0.917878078	0.568044816
0.917453599	0.018906195	0.387149087	0.955230069	0.61438148
0.50275347	0.061608745	0.391285099	0.774681754	0.544643761
0.980188673	0.013912784	0.344231879	0.947791445	0.585655705
0.639031016	0.026953267	0.39283723	0.841407651	0.572265398

fold	embed dim	mean_average_precision	Average silhouette width (cell type)	Average silhouette width (batch)
0	128	0.706188389	0.548575226	0.89252156
1	128	0.707323328	0.548303116	0.906170368
2	128	0.704612414	0.546103332	0.927480817
3	128	0.700556458	0.540824808	0.930000007
4	128	0.700016951	0.541446324	0.926071942
0	64	0.675137859	0.53012953	0.924337864
1	64	0.671839083	0.528695885	0.93178463
2	64	0.678808587	0.538759813	0.941469193
3	64	0.676188697	0.53493445	0.908232033
4	64	0.669981614	0.533029366	0.908285737
0	32	0.648769102	0.538593061	0.932272613
1	32	0.656486555	0.538394295	0.917565227
2	32	0.642499213	0.534144871	0.937769711
3	32	0.635729961	0.533075042	0.938174605
4	32	0.640626517	0.531704746	0.942454636
0	256	0.709095403	0.535671353	0.890350342
1	256	0.696261395	0.521700894	0.942803383
2	256	0.696675129	0.527923314	0.959396362
3	256	0.703513632	0.542145379	0.922052264
4	256	0.687291565	0.521689001	0.974942386

graph_connectivity	neighbor_conservation	seurat_alignment_score	foscttm
0.893635484	0.203001321	0.899153452	0.028634694
0.904891019	0.201629858	0.880169313	0.026483238
0.906338459	0.2001626	0.884549082	0.019334751
0.899175497	0.204139277	0.892321079	0.016486849
0.907007346	0.205659515	0.811474199	0.020567782
0.889975361	0.172447925	0.901045373	0.020935249
0.899763008	0.172631384	0.905011362	0.018159392
0.894441495	0.171185457	0.880513912	0.029760408
0.872829331	0.175170998	0.785880973	0.032627409
0.868378871	0.174474248	0.84019839	0.02442595
0.795039128	0.146009546	0.936351368	0.030428206
0.797894236	0.150957365	0.911140666	0.033795033
0.79234619	0.147508239	0.927093658	0.029497725
0.802738995	0.145995085	0.959655712	0.026533095
0.802779453	0.145895603	0.973315498	0.025032339
0.914438041	0.210667661	0.705237576	0.026277953
0.913518915	0.200340561	0.878980637	0.009899106
0.928179841	0.206393057	0.963570963	0.005887243
0.915425373	0.191495867	0.606395103	0.03640305
0.920766183	0.198412012	0.986341714	0.005441476

biology conservation	omics mixing	Overall integration score
0.947339165	0.513959401	0.77398726
0.942064228	0.578708055	0.796721759
0.894923654	0.670075906	0.804984555
0.83151781	0.669243446	0.766608064
0.844595613	0.602055916	0.747579735
0.420338378	0.632008876	0.505006577
0.388520411	0.688850852	0.508652587
0.537516804	0.692461779	0.599494794
0.49869747	0.42543251	0.469391486
0.443291069	0.462376333	0.450925174
0.269404488	0.46127832	0.346154021
0.327466906	0.388212772	0.351765252
0.193481803	0.468209169	0.303372749
0.141675213	0.523875621	0.294555376
0.146421762	0.552824237	0.308982752
0.840018783	0.386327311	0.658542194
0.555357387	0.743189608	0.630490275
0.665529924	0.91876404	0.76682357
0.79625979	0.426954917	0.64853784
0.504531029	0.981807017	0.695441424

method	mean_av	Average silhouette width (cell type)	Average silhouette width (batch)	graph_co	neighbor_	seurat_al	foscttm	biology c
RtRs_AtA	0.612	0.501	0.673	0.575	0.169	0.035	0.512	0.746
RtAs_RsA	0.648	0.507	0.584	0.576	0.185	0.028	0.481	0.828
RtAt_RsA	0.696	0.534	0.948	0.934	0.188	0.931	0.012	0.906

omics mi:	Overall integration score
0.582	0.681
0.56	0.721
0.891	0.9

fold	method	data	gene-wise pearson	gene-wise spearman
1	Attune	10X Multiomis (11909 cells)	0.271090238	0.242903922
2	Attune	10X Multiomis (11909 cells)	0.273824178	0.247215429
3	Attune	10X Multiomis (11909 cells)	0.271090238	0.242260487
4	Attune	10X Multiomis (11909 cells)	0.269055564	0.24172355
5	Attune	10X Multiomis (11909 cells)	0.269894374	0.24230947
1	Babel	10X Multiomis (11909 cells)	0.238933224	0.231096099
2	Babel	10X Multiomis (11909 cells)	0.240366417	0.236666302
3	Babel	10X Multiomis (11909 cells)	0.236870107	0.232715446
4	Babel	10X Multiomis (11909 cells)	0.237460062	0.233148242
5	Babel	10X Multiomis (11909 cells)	0.231782766	0.221775233
1	Polarbear	10X Multiomis (11909 cells)	0.246805469	0.219425662
2	Polarbear	10X Multiomis (11909 cells)	0.249427211	0.222515346
3	Polarbear	10X Multiomis (11909 cells)	0.252360613	0.221630709
4	Polarbear	10X Multiomis (11909 cells)	0.247649537	0.21891009
5	Polarbear	10X Multiomis (11909 cells)	0.246502016	0.217417622

cell-wise pearson	cell-wise spearman	RMSE
0.666248086	0.416821048	0.528193
0.669694704	0.421254485	0.5268862
0.667194198	0.416142513	0.529023
0.664145703	0.412877439	0.5290738
0.665983824	0.41390667	0.52823395
0.950884821	0.42062725	3.057968
0.952266705	0.423499432	2.2468605
0.953766459	0.419150537	2.2855103
0.953226997	0.419480285	2.4865284
0.948524744	0.41741065	2.2773008
0.684754519	0.410022739	0.600261838
0.68901334	0.414471032	0.602313093
0.689434701	0.412794781	0.599803005
0.686681526	0.411686344	0.600477124
0.686940267	0.410910207	0.599708679

method	RMSE
Attune	0.2196
Amateur	0.2266
AXX (Jiwei Liu & Xueer Chen)	0.2366
scJoint	0.2377
LiuZLab3	0.2386

fold	method	gene-wis	gene-wis	RMSE
1	MLP(+)	0.276	0.247	0.527
2	MLP(+)	0.276	0.246	0.528
3	MLP(+)	0.275	0.247	0.528
4	MLP(+)	0.273	0.246	0.53
5	MLP(+)	0.273	0.245	0.531
1	MLP(-)	0.266	0.242	0.531
2	MLP(-)	0.258	0.231	0.533
3	MLP(-)	0.256	0.232	0.533
4	MLP(-)	0.253	0.23	0.54
5	MLP(-)	0.25	0.229	0.535
1	LASSO	0.206	0.211	0.55
2	LASSO	0.202	0.207	0.564
3	LASSO	0.2	0.198	0.569
4	LASSO	0.197	0.195	0.571
5	LASSO	0.199	0.196	0.57

+: with Attune pretrained

-: without Attune pretrained

LASSO: sklearn.linear_model Lasso (alpha=0.1, max_iter=100000)

fold	method	gene-wise pearson	gene-wise spearman	RMSE
1	RtRs_AtAs	0.268634883	0.240664315	0.527862669
1	RtAs_RsAt	0.266784727	0.239044185	0.532484227
1	RtAt_RsAs	0.271090238	0.242903922	0.528193
2	RtRs_AtAs	0.266125089	0.240616145	0.529241377
2	RtAs_RsAt	0.266520779	0.24052851	0.528346907
2	RtAt_RsAs	0.273824178	0.247215429	0.5268862
3	RtRs_AtAs	0.261927835	0.23367682	0.529702891
3	RtAs_RsAt	0.269653606	0.241845971	0.531787594
3	RtAt_RsAs	0.271090238	0.242260487	0.529023
4	RtRs_AtAs	0.260289054	0.232475572	0.531219515
4	RtAs_RsAt	0.262130546	0.23550083	0.530191509
4	RtAt_RsAs	0.269055564	0.24172355	0.5290738
5	RtRs_AtAs	0.26343837	0.235330541	0.529776271
5	RtAs_RsAt	0.263778006	0.236305457	0.529456625
5	RtAt_RsAs	0.269894374	0.24230947	0.52823395

gene	peak	weight
GBE1	chr3-81760210-81762316	0.01602161
LINC00662	chr19-27792905-27795090	0.01576839
NEGR1	chr1-72281087-72285474	0.015559921
CNTLN	chr9-17134549-17135840	0.015556715
PEX2	chr8-76998902-77001317	0.015513564
AC006504.5	chr19-27792905-27795090	0.015484083
ZNF804A	chr2-184596626-184600488	0.015387084
SLC16A7	chr12-59595133-59598335	0.015302409
CCDC59	chr12-82685581-82688529	0.010532711
CCDC59	chr12-82357590-82359560	0.010514989
C15orf41	chr15-36578867-36580271	0.010493986
C15orf41	chr15-35545210-35547024	0.010480937
PLEKHA5	chr12-19129166-19131196	0.010443674
PLEKHA5	chr12-19438815-19441371	0.010442254
LINC01684	chr21-23968474-23969677	0.010436174
LINC01684	chr21-25456372-25458052	0.010424158
GUF1	chr4-44677602-44679547	0.010418901
GUF1	chr4-44725568-44727280	0.010402501
LIN7A	chr12-79927735-79929270	0.010400771
LIN7A	chr12-79933441-79936623	0.010398692
PDGFC	chr4-155953025-155954533	0.0103972
SVIP	chr11-22828833-22830430	0.010391895
METTL25	chr12-82685581-82688529	0.010386489
SVIP	chr11-22624614-22626791	0.010385498
PDGFC	chr4-156970536-156972421	0.010384252
METTL25	chr12-82357590-82359560	0.010369014
TMTC2	chr12-82685581-82688529	0.010352049
TMTC2	chr12-82357590-82359560	0.010334628
CHRM3-AS2	chr1-239501518-239502483	0.010330932
CHRM3-AS2	chr1-239717259-239720699	0.010327214
AC114763.1	chr2-137968727-137969855	0.010322117
AC114763.1	chr2-138500721-138505594	0.010301826
KHDRBS2	chr6-63352024-63353548	0.010284029
TENT5A	chr6-81750868-81754718	0.010280313
KHDRBS2	chr6-62285408-62286740	0.010265652
TENT5A	chr6-82246424-82248890	0.010261145
IKZF2	chr2-213150016-213152894	0.010248645
LINC02328	chr14-85599791-85600661	0.010243915
IKZF2	chr2-213283646-213285064	0.010238545
LINC02328	chr14-85933734-85935467	0.010230485
LINC02316	chr14-85599791-85600661	0.010213815
HNMT	chr2-137968727-137969855	0.01020805
LINC02100	chr5-18744316-18747489	0.010203317
LINC02316	chr14-85933734-85935467	0.010200426
LINC02100	chr5-18697547-18699009	0.010196785
HNMT	chr2-138500721-138505594	0.01018798
LARGE1	chr22-33919437-33922207	0.010161709
LARGE1	chr22-32723350-32724221	0.010154694
TBC1D32	chr6-121333914-121335761	0.008060379

TBC1D32	chr6-122398808-122400963	0.00805885
TBC1D32	chr6-122469313-122472681	0.008057247
ARL13B	chr3-93973099-93974327	0.007710051
ARL13B	chr3-93979357-93980961	0.007703515
LINC01473	chr2-186484157-186487669	0.007698027
ARL13B	chr3-94062038-94064925	0.007689661
LINC01473	chr2-186589301-186591530	0.007679122
LINC01473	chr2-187168699-187170144	0.007665082
AC078881.1	chr4-176319136-176321696	0.007575621
AC078881.1	chr4-177307940-177310566	0.007575511
AC078881.1	chr4-177440759-177443486	0.007574842
WDR49	chr3-168094586-168096760	0.006211595
NBEA	chr13-34941574-34944030	0.006204987
WDR49	chr3-167750082-167750970	0.006202317
NBEA	chr13-33817449-33819062	0.006197772
WDR49	chr3-167733889-167736332	0.006196979
WDR49	chr3-167379737-167380687	0.006192545
NBEA	chr13-33786534-33787739	0.006189488
NBEA	chr13-33743631-33745171	0.00618305
PKIA	chr8-78444743-78445828	0.006176538
PKIA	chr8-78515438-78517334	0.006167715
PKIA	chr8-78665050-78667018	0.006162926
PKIA	chr8-78804190-78805806	0.006156961
NAALADL2	chr3-174439600-174441843	0.006128894
NAALADL2	chr3-174336708-174338269	0.006121242
NAALADL2	chr3-174330850-174332884	0.00611779
NAALADL2	chr3-173395013-173396183	0.006114584
DPYD	chr1-97847965-97850414	0.006093971
DPYD	chr1-98870882-98872253	0.006093198
DPYD	chr1-98053462-98054605	0.006087855
DPYD	chr1-97919472-97921991	0.006086405
FHIT	chr3-60080313-60082335	0.006033916
FHIT	chr3-62318549-62319675	0.006033043
FHIT	chr3-61008144-61009603	0.006028887
LANCL1	chr2-210475552-210477797	0.006028455
FHIT	chr3-61249672-61252152	0.006026872
LANCL1	chr2-209423571-209424481	0.006021152
LANCL1	chr2-210169525-210172483	0.006020525
LANCL1	chr2-209999838-210003831	0.006015997
ARL14EP	chr11-31368862-31370453	0.005259148
ARL14EP	chr11-30919226-30920158	0.00525823
ARL14EP	chr11-30486962-30487815	0.005246268
ARL14EP	chr11-31508850-31510621	0.005243887
ARL14EP	chr11-30322541-30324214	0.005233801
CPNE8	chr12-38316031-38317551	0.00522684
CPNE8	chr12-38904423-38907283	0.005226174
LRRC40	chr1-71080098-71082132	0.005224469
LRRC40	chr1-70410185-70413124	0.005220748
SMYD2	chr1-213987329-213989351	0.005220448
CPNE8	chr12-39441798-39444184	0.005216823

SMYD2	chr1-214631833-214632688	0.00521447
LRRC40	chr1-70353415-70356587	0.005213097
SMYD2	chr1-214279933-214282962	0.005212649
SMYD2	chr1-214602481-214604376	0.005206985
SMYD2	chr1-214427603-214428552	0.005206001
CPNE8	chr12-39617890-39622900	0.005205724
LRRC40	chr1-70219717-70222355	0.005205197
CPNE8	chr12-40104805-40108131	0.005200494
DACH1	chr13-72780703-72783177	0.005200097
DACH1	chr13-73058286-73061021	0.005198108
LRRC40	chr1-70204724-70206517	0.005197536
DACH1	chr13-72726532-72728700	0.005195545
MARCKS	chr6-113629908-113633146	0.005189539
DACH1	chr13-71864479-71867591	0.005185879
MARCKS	chr6-113634178-113635638	0.005184657
MARCKS	chr6-113854439-113860529	0.005183303
MARCKS	chr6-113809456-113810428	0.005182522
MARCKS	chr6-113969735-113972183	0.005181739
DACH1	chr13-71290207-71292160	0.005177846
VCAN-AS1	chr5-83471006-83474938	0.005173834
VCAN-AS1	chr5-83368092-83369612	0.005157154
GALNT3	chr2-165952545-165954696	0.005154409
GALNT3	chr2-165793071-165795528	0.005150356
VCAN-AS1	chr5-83312328-83313705	0.005141331
PAM	chr5-102865392-102866664	0.005139973
GALNT3	chr2-165293463-165294357	0.005139033
VCAN-AS1	chr5-83076564-83078352	0.005138686
PAM	chr5-102754123-102756603	0.005137619
VCAN-AS1	chr5-83306386-83307448	0.00513434
PAM	chr5-103119134-103121360	0.00513274
GALNT3	chr2-164840372-164842439	0.005126854
PAM	chr5-102294901-102297436	0.005125214
PAM	chr5-103258064-103260223	0.00512253
GALNT3	chr2-164722396-164723363	0.005121653
MMS22L	chr6-96499497-96500444	0.005121631
MMS22L	chr6-97282232-97283917	0.005119481
COBLL1	chr2-165952545-165954696	0.005110395
MMS22L	chr6-96520694-96522930	0.005110064
UBA6-AS1	chr4-68373388-68374074	0.005108509
MMS22L	chr6-96923261-96925571	0.005106587
COBLL1	chr2-165793071-165795528	0.005106372
TOGARAM1	chr14-45083257-45085352	0.005106366
TOGARAM1	chr14-45133510-45136677	0.005104605
MMS22L	chr6-96896928-96899280	0.005103351
FBN2	chr5-129093930-129095778	0.005102471
UBA6-AS1	chr4-67544088-67546379	0.00510185
TOGARAM1	chr14-44960210-44964450	0.005101815
LRRC7	chr1-70410185-70413124	0.005100445
UBA6-AS1	chr4-68345768-68351257	0.005097602
MCPH1-AS1	chr8-6717913-6718841	0.005097177

TOGARAM1	chr14-44896412-44898341	0.005096876
UBA6-AS1	chr4-67558307-67559177	0.005095837
COBLL1	chr2-165293463-165294357	0.005095148
TOGARAM1	chr14-45249725-45255289	0.005095127
MCPH1-AS1	chr8-6747979-6749000	0.005094061
UBA6-AS1	chr4-67699557-67702063	0.005093029
LRRC7	chr1-70353415-70356587	0.005092974
MCPH1-AS1	chr8-6707492-6709950	0.005092184
CD36	chr7-80918332-80919727	0.005089471
FBN2	chr5-128537218-128539090	0.005085953
LRRC7	chr1-70219717-70222355	0.005085256
COBLL1	chr2-164840372-164842439	0.005083073
MCPH1-AS1	chr8-6690005-6691027	0.005082748
FBN2	chr5-127516968-127518638	0.005081398
COBLL1	chr2-164722396-164723363	0.005077919
LRRC7	chr1-70204724-70206517	0.005077769
FBN2	chr5-128082022-128085243	0.005077331
FBN2	chr5-128196652-128198484	0.005076977
MCPH1-AS1	chr8-6405394-6407730	0.005076138
CD36	chr7-80721489-80723180	0.005071379
LRRC7	chr1-68496260-68497749	0.00506757
MAT2B	chr5-163502802-163507291	0.005064563
CD36	chr7-80637997-80639005	0.005063574
CD36	chr7-80442982-80443925	0.005063366
CD36	chr7-80134292-80136033	0.005062786
MAT2B	chr5-163458853-163462830	0.005060677
MAT2B	chr5-163914784-163917341	0.00505731
MAT2B	chr5-163436511-163439301	0.005046532
MAT2B	chr5-163943287-163944187	0.005043632
MCPH1	chr8-6717913-6718841	0.005019438
MCPH1	chr8-6747979-6749000	0.005016372
MCPH1	chr8-6707492-6709950	0.005014521
MCPH1	chr8-6690005-6691027	0.005005227
MCPH1	chr8-6405394-6407730	0.004998715
ANGPT2	chr8-6717913-6718841	0.004828401
ANGPT2	chr8-6747979-6749000	0.004825454
ANGPT2	chr8-6707492-6709950	0.004823668
ANGPT2	chr8-6690005-6691027	0.004814731
ANGPT2	chr8-6405394-6407730	0.004808469
STXBP4	chr17-55419029-55422709	0.004557483
STXBP4	chr17-55576084-55578177	0.004555527
STXBP4	chr17-55263524-55266540	0.00455408
STXBP4	chr17-55750542-55751743	0.004551983
STXBP4	chr17-55237152-55239276	0.004547053
STXBP4	chr17-54967519-54969686	0.0045426
ZNF248	chr10-38401661-38404057	0.004538212
EIF2AK3	chr2-88690858-88692877	0.004534508
ZNF248	chr10-38355790-38356688	0.004531725
ZNF248	chr10-38093449-38095618	0.004526237
ZNF248	chr10-37856678-37858720	0.004526185

ZNF248	chr10-37975128-37977496	0.00452604
EIF2AK3	chr2-88626170-88629719	0.004525611
ZNF248	chr10-38009437-38012042	0.004524906
EIF2AK3	chr2-88599566-88600668	0.004516816
EIF2AK3	chr2-88169750-88171316	0.004512046
EIF2AK3	chr2-88054128-88056846	0.004510989
EIF2AK3	chr2-88015405-88017801	0.00450998
AC106818.2	chr5-94617787-94619538	0.004509693
AC106818.2	chr5-94432362-94434176	0.004509058
AC106818.2	chr5-94110725-94112475	0.004500193
WARS2	chr1-119564430-119566186	0.004493737
WARS2	chr1-119139296-119141675	0.004493086
WARS2	chr1-119645730-119648799	0.004488214
AC106818.2	chr5-93620394-93623304	0.004484091
WARS2	chr1-119987043-119989225	0.004480767
WARS2	chr1-120068879-120069874	0.004474627
SEMA3C	chr7-81845621-81847104	0.004471682
WARS2	chr1-120175677-120177294	0.004469667
AC106818.2	chr5-93583842-93585789	0.004467291
TMX4	chr20-8434328-8436042	0.004466687
FAM3C	chr7-120949757-120952271	0.004461956
TMX4	chr20-8427434-8428483	0.004457334
FAM3C	chr7-122143294-122144866	0.004456955
AC106818.2	chr5-93578321-93582136	0.004456915
FAM3C	chr7-120987526-120990124	0.004455832
FAM3C	chr7-121437946-121438961	0.004451774
SEMA3C	chr7-80918332-80919727	0.004451569
TMX4	chr20-8399825-8400776	0.004451001
FAM3C	chr7-120995014-120998359	0.004449487
FAM3C	chr7-121395363-121396963	0.00444808
TMX4	chr20-8393444-8394895	0.004445922
TMX4	chr20-8131589-8133640	0.004438907
IL7	chr8-78444743-78445828	0.004437957
WDFY3	chr4-84965636-84967654	0.004437191
WDFY3	chr4-84582081-84584425	0.004437068
WDFY3	chr4-85701619-85702533	0.004436114
SEMA3C	chr7-80721489-80723180	0.004435746
WDFY3	chr4-85474578-85475822	0.004434755
WDFY3	chr4-84496341-84499794	0.004434583
WDFY3	chr4-85681027-85681777	0.004433324
IL7	chr8-78515438-78517334	0.004431619
TMX4	chr20-8017389-8021516	0.004429421
SEMA3C	chr7-80637997-80639005	0.004428919
SEMA3C	chr7-80442982-80443925	0.004428736
SEMA3C	chr7-80134292-80136033	0.004428229
IL7	chr8-78665050-78667018	0.004428173
MDFIC	chr7-114051710-114052584	0.004427456
IL7	chr8-78804190-78805806	0.004423886
MDFIC	chr7-114084416-114087608	0.004422093
IL7	chr8-79764936-79769241	0.004417846

IL7	chr8-79783335-79784544	0.004413667
MDFIC	chr7-114920818-114924736	0.004410454
TSHZ3	chr19-32421579-32422558	0.00440752
TSHZ3	chr19-32403602-32406699	0.004403287
PLCB1	chr20-8434328-8436042	0.00440247
TSHZ3	chr19-31213780-31214784	0.004396332
TSHZ3	chr19-32344852-32346632	0.004396169
MDFIC	chr7-114930846-114932255	0.004395691
WARS2-AS1	chr1-119564430-119566186	0.004394854
WARS2-AS1	chr1-119139296-119141675	0.004394219
PLCB1	chr20-8427434-8428483	0.00439325
TSHZ3	chr19-31339292-31341021	0.00439142
TSHZ3	chr19-31348468-31352221	0.004391107
WARS2-AS1	chr1-119645730-119648799	0.004389455
PLCB1	chr20-8399825-8400776	0.004387008
MDFIC	chr7-115008382-115010181	0.004385074
MDFIC	chr7-115410360-115411648	0.004384019
WARS2-AS1	chr1-119987043-119989225	0.004382174
PLCB1	chr20-8393444-8394895	0.004382
LRRK2	chr12-39441798-39444184	0.004379649
WARS2-AS1	chr1-120068879-120069874	0.004376169
PLCB1	chr20-8131589-8133640	0.004375085
LRRK2	chr12-40223581-40227289	0.004373887
IGKC	chr2-88690858-88692877	0.004373622
LRRK2	chr12-40208256-40209541	0.004372734
WARS2-AS1	chr1-120175677-120177294	0.004371319
LRRK2	chr12-39617890-39622900	0.004370335
TMEM117	chr12-44874887-44877228	0.00436998
TMEM117	chr12-44846908-44849095	0.004368397
LRRK2	chr12-40158215-40159278	0.004368142
CPED1	chr7-120949757-120952271	0.004366646
LRRK2	chr12-40104805-40108131	0.004365943
PLCB1	chr20-8017389-8021516	0.004365735
IGKC	chr2-88626170-88629719	0.004365039
AUTS2	chr7-70659262-70661084	0.004362271
CPED1	chr7-122143294-122144866	0.004361751
CPED1	chr7-120987526-120990124	0.004360653
SHTN1	chr10-117174636-117176364	0.004360309
SHTN1	chr10-117004218-117005560	0.004360255
TMEM117	chr12-44827621-44828613	0.004360136
AUTS2	chr7-70693367-70696141	0.004358116
AUTS2	chr7-70647703-70648898	0.004357914
CPED1	chr7-121437946-121438961	0.004356681
IGKC	chr2-88599566-88600668	0.004356556
SHTN1	chr10-116947071-116948892	0.004355862
SHTN1	chr10-117373223-117376854	0.004355326
CPED1	chr7-120995014-120998359	0.00435444
CPED1	chr7-121395363-121396963	0.004353066
TMEM117	chr12-43758076-43760654	0.004352528
IGKC	chr2-88169750-88171316	0.004351958

SHTN1	chr10-116741702-116743249	0.004351315
TMEM117	chr12-43835259-43837035	0.00435121
IGKC	chr2-88054128-88056846	0.004350937
IGKC	chr2-88015405-88017801	0.004349964
SHTN1	chr10-118045775-118047713	0.00434915
TMEM117	chr12-43805268-43807822	0.004348183
AUTS2	chr7-69824087-69825436	0.004346537
AUTS2	chr7-69596896-69600496	0.004334885
AUTS2	chr7-69256400-69257257	0.004329867
TPK1	chr7-144812385-144813490	0.004328704
TPK1	chr7-143901245-143902861	0.004326475
TPK1	chr7-144817944-144819177	0.004324321
TPK1	chr7-143836128-143837663	0.004318994
TPK1	chr7-144834071-144836850	0.004316934
TPK1	chr7-144841896-144843091	0.004311386
SGCE	chr7-95608191-95609264	0.004298004
SGCE	chr7-95595997-95597224	0.004283236
SGCE	chr7-94003447-94005197	0.00428322
SGCE	chr7-94509151-94511242	0.004271434
SGCE	chr7-95434317-95435703	0.004269835
SGCE	chr7-94655180-94659044	0.004265285
AL049828.1	chr14-39102008-39104385	0.004232721
AL049828.1	chr14-39113674-39115515	0.00422825
AL049828.1	chr14-39169386-39171234	0.004227399
AL049828.1	chr14-39173869-39176286	0.004226006
AL049828.1	chr14-39265701-39268889	0.004222616
AL049828.1	chr14-39430396-39433917	0.004219559
QDPR	chr4-17336377-17337306	0.003939761
QDPR	chr4-17511322-17512676	0.003931856
QDPR	chr4-17808399-17811785	0.003924541
QDPR	chr4-18020074-18023234	0.00392449
QDPR	chr4-17549013-17550146	0.00392406
TAF4B	chr18-26547564-26550292	0.003921443
QDPR	chr4-17612874-17615642	0.003921395
TAF4B	chr18-26225271-26228111	0.003921302
QDPR	chr4-17576006-17580983	0.00392018
TAF4B	chr18-26656001-26657460	0.003914896
TAF4B	chr18-26215154-26216644	0.003912142
TAF4B	chr18-26190206-26191652	0.003897552
SPATA6	chr1-47313185-47315437	0.003897253
DCUN1D4	chr4-52721580-52723271	0.003894663
KIAA1328	chr18-35808775-35810999	0.003893739
DCUN1D4	chr4-52711230-52713852	0.00389265
SPATA6	chr1-47332973-47335194	0.003891473
DCUN1D4	chr4-52861470-52862952	0.00389067
KIAA1328	chr18-36075632-36076619	0.003888769
KIAA1328	chr18-35969980-35973844	0.003888494
COL19A1	chr6-69814842-69817025	0.003888308
KIAA1328	chr18-36128076-36131500	0.003888139
KIAA1328	chr18-36066258-36068131	0.003887715

COL19A1	chr6-69866205-69868165	0.00388743
COL19A1	chr6-70955457-70956858	0.00388645
KIAA1328	chr18-36186568-36188116	0.003884825
DCUN1D4	chr4-52657901-52660406	0.003883691
TAF4B	chr18-26089179-26091761	0.003883506
COL19A1	chr6-70412458-70414725	0.003882547
SPATA6	chr1-47432082-47435365	0.003882519
COL19A1	chr6-70666599-70669202	0.003881599
KIAA1328	chr18-36827595-36830287	0.003881455
COL19A1	chr6-69795355-69797749	0.00388133
COL19A1	chr6-70566263-70567698	0.003879645
SPATA6	chr1-48775648-48777807	0.003877919
ATP10A	chr15-24954630-24957129	0.00387676
NRG1	chr8-30655667-30659145	0.003876325
SLX4IP	chr20-10034541-10036083	0.003875167
SPATA6	chr1-47436166-47439852	0.003874198
TAF4B	chr18-25349325-25352717	0.003873592
ATP10A	chr15-25437682-25439985	0.003873166
SPATA6	chr1-48469915-48473084	0.003873129
DCUN1D4	chr4-52051028-52052970	0.003872709
SNRPB2	chr20-17609386-17618285	0.003872539
SNRPB2	chr20-17679797-17683356	0.003871765
SLX4IP	chr20-10433054-10436152	0.003871747
SLX4IP	chr20-9397703-9398506	0.003871222
SPATA6	chr1-48244936-48245747	0.003870759
DCUN1D4	chr4-51841781-51845440	0.003866784
DCUN1D4	chr4-52037631-52038877	0.003866158
SNRPB2	chr20-17568086-17571777	0.00386523
ATP10A	chr15-25810402-25812898	0.003863625
NRG1	chr8-30721888-30724685	0.003862749
SLX4IP	chr20-10488180-10489178	0.003862658
PTPRM	chr18-8703707-8707587	0.0038573
PTPRM	chr18-8755231-8756215	0.003857232
SNRPB2	chr20-17530442-17531825	0.003856514
NRG1	chr8-32574764-32575691	0.003853543
CCDC50	chr3-190231353-190232454	0.003853281
SLX4IP	chr20-10504183-10505781	0.003853202
CCDC50	chr3-190512855-190515642	0.003852976
ATP10A	chr15-25845252-25847747	0.003852731
SNRPB2	chr20-17503581-17507647	0.003851754
TNFSF13B	chr13-108367973-108369110	0.003851685
TNFSF13B	chr13-109389658-109390568	0.003851523
SNRPB2	chr20-16572851-16575823	0.003851487
TCF4	chr18-55320948-55322914	0.003851406
SNRPB2	chr20-16729302-16731029	0.003851269
PTPRM	chr18-8623381-8624430	0.003849955
CCDC50	chr3-191328603-191330615	0.003849456
NRG1	chr8-30725479-30728586	0.003849034
SLX4IP	chr20-10671307-10675234	0.003848308
SLX4IP	chr20-10511220-10512193	0.003848143

NRG1	chr8-31032169-31034808	0.003848017
CCDC50	chr3-190565990-190567125	0.003847771
PLXDC2	chr10-20128540-20129366	0.003847089
CCDC50	chr3-191245731-191246861	0.003845715
TNFSF13B	chr13-108342123-108343387	0.003845381
ATP10A	chr15-25848841-25851453	0.003844289
CCDC50	chr3-190585551-190587807	0.003842787
CCDC50	chr3-190616457-190620134	0.003842186
NRG1	chr8-30811779-30813375	0.003842001
TCF4	chr18-56650111-56653548	0.003841865
TCF4	chr18-55477222-55478334	0.003841761
NRG1	chr8-30743299-30744993	0.003841311
PLXDC2	chr10-20001997-20005131	0.003839859
PTPRM	chr18-8607909-8611260	0.00383912
ATP10A	chr15-25858839-25863999	0.003838343
CBLB	chr3-106176355-106177447	0.003837474
PLXDC2	chr10-19823762-19827378	0.003836705
CD2AP	chr6-47476551-47479364	0.003835268
TNFSF13B	chr13-108268438-108270857	0.003835239
PLXDC2	chr10-19815338-19818725	0.003834826
TCF4	chr18-56637327-56639632	0.003833935
CD2AP	chr6-46376363-46377330	0.003833725
ATP10A	chr15-26080855-26083304	0.003832073
PLXDC2	chr10-18657978-18661103	0.003831204
PTPRM	chr18-8603422-8604752	0.003830688
CBLB	chr3-105882343-105883261	0.003830663
TCF4	chr18-55587123-55590503	0.003830401
PTPRM	chr18-6413657-6415410	0.003829355
TNFSF13B	chr13-108214304-108216158	0.003828933
PTPRM	chr18-7566405-7568752	0.003827962
TNFSF13B	chr13-108266196-108267824	0.003827239
PLXDC2	chr10-18650439-18652959	0.003826041
TCF4	chr18-56257136-56258797	0.003825943
TNFSF13B	chr13-108217563-108220453	0.003825509
TCEA1	chr8-54152661-54153872	0.003825509
CD2AP	chr6-46652428-46653718	0.003825499
CBLB	chr3-105866486-105870115	0.003824786
TCF4	chr18-55778545-55781183	0.003823984
CD2AP	chr6-47413711-47415356	0.003823817
PLXDC2	chr10-18627337-18628199	0.00382256
CBLB	chr3-105827297-105831313	0.003821251
TCEA1	chr8-52939443-52942030	0.003820192
CBLB	chr3-105764352-105765473	0.003818328
HMGB2	chr4-172785982-172787758	0.003816973
CD2AP	chr6-46770683-46773807	0.003816206
VCAN	chr5-83471006-83474938	0.003815417
TCEA1	chr8-53657120-53658583	0.003814077
CBLB	chr3-105752368-105754731	0.003813965
CD2AP	chr6-47308729-47310324	0.003813686
HMGB2	chr4-173167779-173170521	0.003813619

HMGB2	chr4-173331932-173335820	0.003812893
TCEA1	chr8-54134520-54135997	0.003811577
CD2AP	chr6-47241931-47243237	0.003810876
HMGB2	chr4-173369095-173373124	0.003810288
IFI44	chr1-77681420-77684246	0.003809259
CBLB	chr3-105365587-105370254	0.00380872
TCEA1	chr8-53842427-53845396	0.003806257
VCAN	chr5-82277158-82279373	0.003803295
VCAN	chr5-83368092-83369612	0.003803118
HMGB2	chr4-173414745-173417490	0.003803049
TCEA1	chr8-54100798-54103264	0.003802151
IFI44	chr1-77758745-77760343	0.003801947
TCEA1	chr8-54020285-54023209	0.003800758
VCAN	chr5-82356743-82357918	0.003797257
IFI44	chr1-77779018-77780406	0.003796433
HMGB2	chr4-174281887-174284902	0.003792731
VCAN	chr5-83312328-83313705	0.003791449
VCAN	chr5-83076564-83078352	0.003789497
IFI44	chr1-77975075-77980661	0.003788918
VCAN	chr5-83306386-83307448	0.003786293
LRIG1	chr3-66220039-66221725	0.003784172
HMGB2	chr4-174520571-174523622	0.003783647
LRIG1	chr3-66301898-66305121	0.003780275
ABCD2	chr12-38904423-38907283	0.003778873
IFI44	chr1-78003227-78006330	0.003778823
SLC2A13	chr12-38904423-38907283	0.003778557
ABCD2	chr12-39441798-39444184	0.00377211
SLC2A13	chr12-39441798-39444184	0.003771795
LRIG1	chr3-66393150-66394736	0.003771257
IFI44	chr1-78619344-78621165	0.003769764
ABCD2	chr12-40223581-40227289	0.003767145
SLC2A13	chr12-40223581-40227289	0.003766831
IFI44	chr1-78649318-78651879	0.003766209
ABCD2	chr12-40208256-40209541	0.00376615
SLC2A13	chr12-40208256-40209541	0.003765838
ABCD2	chr12-39617890-39622900	0.003764084
SLC2A13	chr12-39617890-39622900	0.003763771
ABCD2	chr12-40158215-40159278	0.003762197
SLC2A13	chr12-40158215-40159278	0.003761884
LRIG1	chr3-66499161-66501973	0.003761166
ABCD2	chr12-40104805-40108131	0.003760302
SLC2A13	chr12-40104805-40108131	0.003759991
LRIG1	chr3-66971762-66972810	0.003753305
ALCAM	chr3-106176355-106177447	0.003751216
LRIG1	chr3-66997414-67000743	0.003748091
ALCAM	chr3-105882343-105883261	0.00374456
LRIG1	chr3-67652870-67657527	0.003744065
ALCAM	chr3-105866486-105870115	0.00373881
ALCAM	chr3-105827297-105831313	0.003735354
ALCAM	chr3-105764352-105765473	0.003732499

ALCAM	chr3-105752368-105754731	0.003728235
ALCAM	chr3-105365587-105370254	0.003723108
SH3YL1	chr2-460498-461608	0.003555479
SH3YL1	chr2-675385-678665	0.003552849
SH3YL1	chr2-437402-438884	0.003550799
SH3YL1	chr2-429274-431156	0.003543494
SH3YL1	chr2-392791-394885	0.003538522
SH3YL1	chr2-175566-177257	0.003538249
SH3YL1	chr2-45223-47726	0.003537776
SH3YL1	chr2-263037-265826	0.003537495
DMAC1	chr9-7973838-7977945	0.00351648
DMAC1	chr9-7935184-7936768	0.003512164
DMAC1	chr9-7798770-7800303	0.003510178
ARHGAP24	chr4-86593683-86595605	0.003510175
DMAC1	chr9-7626377-7627343	0.003509431
DMAC1	chr9-6756470-6760015	0.003506825
ARHGAP24	chr4-86359643-86360708	0.00350566
ARHGAP24	chr4-84965636-84967654	0.003501458
ARHGAP24	chr4-84582081-84584425	0.003501362
DMAC1	chr9-6750051-6751442	0.003500932
ARHGAP24	chr4-85701619-85702533	0.00350061
ARHGAP24	chr4-85474578-85475822	0.003499537
ARHGAP24	chr4-84496341-84499794	0.003499404
ARHGAP24	chr4-85681027-85681777	0.003498406
DMAC1	chr9-6715366-6716953	0.00349398
DMAC1	chr9-6702560-6705889	0.003490336
IDNK	chr9-83977364-83982018	0.003473924
IDNK	chr9-83964038-83967869	0.003471877
IDNK	chr9-83955874-83957734	0.003468756
ELP4	chr11-31368862-31370453	0.003466057
ELP4	chr11-30919226-30920158	0.003465454
IDNK	chr9-83919981-83922662	0.003465176
SLC8A1	chr2-40482225-40483958	0.003464822
NCK1	chr3-136191335-136197947	0.003464271
NCK1	chr3-136249545-136251850	0.003463174
IDNK	chr9-83816774-83818598	0.003459972
NCK1	chr3-135965282-135966563	0.003459935
ELP4	chr11-30486962-30487815	0.003457571
AC131571.1	chr11-31368862-31370453	0.003456008
ELP4	chr11-31508850-31510621	0.003456
NCK1	chr3-136712824-136714790	0.003455538
AC131571.1	chr11-30919226-30920158	0.003455407
SLC8A1	chr2-40479715-40480825	0.003454553
IDNK	chr9-83705095-83709343	0.003452609
NIFK-AS1	chr2-121197103-121198402	0.003451451
SLC8A1	chr2-39491769-39493332	0.003450308
ELP4	chr11-30322541-30324214	0.003449354
SLC8A1	chr2-39435199-39438467	0.00344857
FRMD3	chr9-83977364-83982018	0.003448441
SLC8A1	chr2-39778271-39781440	0.003448236

FRMD3	chr9-83964038-83967869	0.003446406
AC131571.1	chr11-31508850-31510621	0.003445978
IDNK	chr9-83622342-83624716	0.003445301
SLC8A1	chr2-40471156-40472122	0.003444984
NCK1	chr3-136749101-136754272	0.003444675
SLC8A1	chr2-40449892-40452652	0.003443644
FRMD3	chr9-83955874-83957734	0.003443308
IDNK	chr9-83472260-83474101	0.003442259
SLC8A1	chr2-40455306-40456251	0.003441209
FRMD3	chr9-83919981-83922662	0.003439754
NIFK-AS1	chr2-121286831-121287924	0.003438341
ELP4	chr11-31809892-31813434	0.003438265
PLA2G4A	chr1-186374191-186376433	0.003436716
PLA2G4A	chr1-186617840-186619089	0.003436137
NCK1	chr3-136818236-136820512	0.003435863
FRMD3	chr9-83816774-83818598	0.003434588
RASGRP3	chr2-32355757-32358764	0.003434271
PLA2G4A	chr1-185693689-185694676	0.003432982
NCK1	chr3-137013759-137014700	0.003432874
RASGRP3	chr2-32276948-32279033	0.003432788
NCK1	chr3-136860537-136863478	0.003432231
IMMP1L	chr11-31368862-31370453	0.003432049
AC092546.1	chr4-13482967-13485803	0.003431819
UBE2E2	chr3-23805180-23807735	0.003431608
NIFK-AS1	chr2-121735491-121739353	0.003431599
IMMP1L	chr11-30919226-30920158	0.003431449
RASGRP3	chr2-32484587-32485642	0.003431402
PLA2G4A	chr1-186654648-186656496	0.003431165
UBE2E2	chr3-23808518-23812247	0.003430475
NIFK-AS1	chr2-121754806-121756446	0.003430222
SOX4	chr6-22062651-22063665	0.003429152
PLA2G4A	chr1-185672705-185674714	0.003429048
RASGRP3	chr2-32264227-32266101	0.003428895
AC131571.1	chr11-31809892-31813434	0.003428295
PLA2G4A	chr1-186848685-186850321	0.003427988
SOX4	chr6-21986977-21988148	0.003427287
FRMD3	chr9-83705095-83709343	0.003427283
NIFK-AS1	chr2-121698562-121701281	0.003427218
RASGRP3	chr2-32261849-32262783	0.00342672
UBE2E2	chr3-23748327-23749360	0.003426418
IFI44L	chr1-77430672-77432282	0.003426297
RASGRP3	chr2-32626967-32628731	0.00342597
PLA2G4A	chr1-186679902-186681289	0.003425784
SOX4	chr6-21855530-21857569	0.003425747
NIFK-AS1	chr2-121529668-121533174	0.003425493
UBE2E2	chr3-23915477-23921801	0.003425009
PLA2G4A	chr1-186828456-186830588	0.003424384
SOX4	chr6-20399555-20405156	0.003424008
IMMP1L	chr11-30486962-30487815	0.003423641
AC092546.1	chr4-13541058-13545340	0.003422627

SOX4	chr6-21593026-21597372	0.003422429
IMMP1L	chr11-31508850-31510621	0.00342209
RASGRP3	chr2-33475329-33478581	0.003421799
RASGRP3	chr2-33598282-33600293	0.003421234
NIFK-AS1	chr2-121648099-121651074	0.003420955
UBE2E2	chr3-23943541-23947064	0.003420419
FRMD3	chr9-83622342-83624716	0.003420031
ELP4	chr11-32090535-32092278	0.003420029
SOX4	chr6-20528094-20529542	0.003419251
NIFK-AS1	chr2-121596174-121597302	0.003419103
SOX4	chr6-21586967-21589826	0.003418368
AC092546.1	chr4-13626541-13628595	0.00341806
DPH6	chr15-36578867-36580271	0.003417723
UBE2E2	chr3-23220957-23221899	0.003417698
FRMD3	chr9-83472260-83474101	0.003417011
AC092546.1	chr4-13654010-13654914	0.003416904
SOX4	chr6-20533842-20535368	0.003416725
IMMP1L	chr11-30322541-30324214	0.003415507
IFI44L	chr1-77681420-77684246	0.003415295
STX7	chr6-131627431-131629202	0.003414894
AC092546.1	chr4-14125052-14126109	0.003414836
STX7	chr6-132510684-132514451	0.003413602
DPH6	chr15-35545210-35547024	0.003413474
DPH6	chr15-34986817-34989244	0.003412843
STX7	chr6-132713293-132714592	0.003412099
GKAP1	chr9-83977364-83982018	0.003411336
DPH6	chr15-34968805-34970512	0.003411217
UBE2E2	chr3-23202181-23204329	0.003410455
AC131571.1	chr11-32090535-32092278	0.003410113
GKAP1	chr9-83964038-83967869	0.003409325
NIFK	chr2-121197103-121198402	0.003409011
AC092546.1	chr4-14856049-14856971	0.003408771
IFI44L	chr1-77758745-77760343	0.003408739
ELP4	chr11-32583068-32585332	0.0034074
UBE2E2	chr3-23071379-23072331	0.003407387
STX7	chr6-132733437-132735401	0.003407238
GKAP1	chr9-83955874-83957734	0.003406258
DPH6	chr15-34582301-34584667	0.003406073
IMMP1L	chr11-31809892-31813434	0.00340453
IFI44L	chr1-77779018-77780406	0.003403795
GKAP1	chr9-83919981-83922662	0.003402742
TBC1D4	chr13-74286710-74289103	0.003402618
TBC1D4	chr13-74289861-74290961	0.003400913
AC092546.1	chr4-14877062-14878024	0.003399936
RFC3	chr13-34941574-34944030	0.003399753
STX7	chr6-132762539-132764074	0.003399442
DPH6	chr15-34514055-34515555	0.003399316
CEP290	chr12-88035037-88036315	0.003398981
TBC1D4	chr13-75325701-75327718	0.003398648
GKAP1	chr9-83816774-83818598	0.003397632

LMO7	chr13-75325701-75327718	0.003397551
AC131571.1	chr11-32583068-32585332	0.00339752
DPH6	chr15-34365117-34368781	0.003397335
IFI44L	chr1-77975075-77980661	0.003397054
TBC1D4	chr13-75480687-75482950	0.00339643
NIFK	chr2-121286831-121287924	0.003396062
RFC3	chr13-33817449-33819062	0.003395801
DPH6	chr15-34436190-34438274	0.003395465
LMO7	chr13-75480687-75482950	0.003395333
STX7	chr6-132817674-132821274	0.003394037
CEP290	chr12-88141404-88143171	0.003393381
AC092546.1	chr4-15000965-15005549	0.00339266
THOC7	chr3-63933284-63935296	0.003392636
STX7	chr6-132797828-132799772	0.00339256
TBC1D4	chr13-75537246-75538485	0.003392545
AC131571.1	chr11-32829116-32830811	0.003391885
LMO7	chr13-75537246-75538485	0.003391449
RFC3	chr13-33786534-33787739	0.003391261
STX7	chr6-132811763-132817060	0.00339064
GKAP1	chr9-83705095-83709343	0.003390405
AC131571.1	chr11-32891967-32894779	0.003390157
THOC7	chr3-64030426-64031273	0.003390156
THOC7	chr3-63937384-63938306	0.003390048
THOC7	chr3-63910049-63913608	0.003389804
NIFK	chr2-121735491-121739353	0.003389401
NIFK	chr2-121754806-121756446	0.003388042
IFI44L	chr1-78003227-78006330	0.003388003
RFC3	chr13-33743631-33745171	0.003387732
IMMP1L	chr11-32090535-32092278	0.003386471
THOC7	chr3-64021880-64024177	0.003385659
THOC7	chr3-63967352-63971248	0.003385358
TBC1D4	chr13-75548774-75550866	0.003385315
NIFK	chr2-121698562-121701281	0.003385072
RFC3	chr13-33729775-33731071	0.003384286
LMO7	chr13-75548774-75550866	0.003384222
NIFK	chr2-121529668-121533174	0.003383372
THOC7	chr3-63975596-63980257	0.003383255
GKAP1	chr9-83622342-83624716	0.003383229
THOC7	chr3-63862711-63865257	0.003382448
CEP290	chr12-89155823-89157452	0.003381403
GKAP1	chr9-83472260-83474101	0.003380241
IFI44L	chr1-78619344-78621165	0.003379881
RFC3	chr13-33540610-33544456	0.003379342
NIFK	chr2-121648099-121651074	0.003378886
NIFK	chr2-121596174-121597302	0.003377058
IFI44L	chr1-78649318-78651879	0.003376698
KIF21A	chr12-38316031-38317551	0.003376661
KIF21A	chr12-38904423-38907283	0.003376233
TBC1D4	chr13-75635042-75637417	0.003375838
LMO7	chr13-75635042-75637417	0.003374747

IMMP1L	chr11-32583068-32585332	0.003373965
RFC3	chr13-33352755-33353960	0.00337335
KIF21A	chr12-39441798-39444184	0.003370192
RFC3	chr13-32736564-32738064	0.003369523
TBC1D4	chr13-75760091-75763168	0.003367931
CEP290	chr12-89160318-89164509	0.003367843
ATP5PF	chr21-26168356-26171441	0.003367078
LMO7	chr13-75760091-75763168	0.003366842
LMO7	chr13-76799439-76800163	0.003366402
KIF21A	chr12-40223581-40227289	0.003365754
PRICKLE1	chr12-42468572-42470126	0.003364933
KIF21A	chr12-40208256-40209541	0.003364867
LMO7	chr13-76744909-76746300	0.003363998
ATP5PF	chr21-25967185-25968449	0.003363488
KIF21A	chr12-39617890-39622900	0.003363024
PRICKLE1	chr12-41931646-41933129	0.003362834
PRICKLE1	chr12-42324849-42327432	0.003362625
PRICKLE1	chr12-42482647-42484687	0.003362312
ATP5PF	chr21-25733501-25736674	0.003361879
KIF21A	chr12-40158215-40159278	0.003361333
ATP5PF	chr21-25638644-25640633	0.003361193
KIF21A	chr12-40104805-40108131	0.003359644
ATP5PF	chr21-25606856-25608940	0.003358878
PRICKLE1	chr12-42237117-42239350	0.003357928
PRICKLE1	chr12-42097753-42098734	0.003357807
CEP290	chr12-89165843-89167381	0.003357122
PRICKLE1	chr12-43758076-43760654	0.00335682
PRICKLE1	chr12-42143326-42145943	0.003355515
ATP5PF	chr21-25572695-25574322	0.003353928
CEP290	chr12-89225219-89226079	0.003349845
ATP5PF	chr21-25561345-25564836	0.003348608
ATP5PF	chr21-25456372-25458052	0.003346855
CEP290	chr12-89243674-89245793	0.003343492
CEP290	chr12-89333691-89334393	0.0033358
TAFA2	chr12-62190852-62193097	0.003327111
TAFA2	chr12-62214815-62216329	0.003325769
TAFA2	chr12-62258870-62262612	0.003320412
FER	chr5-108379818-108383773	0.003319414
FER	chr5-109688322-109693187	0.003317867
FER	chr5-109656488-109657600	0.003315263
FER	chr5-109856859-109858211	0.00331507
TAFA2	chr12-62263854-62265965	0.003312065
FER	chr5-108727077-108729662	0.003310235
FER	chr5-109407935-109412077	0.003309771
FER	chr5-109921328-109922885	0.003309247
FER	chr5-108747441-108750521	0.003306777
TAFA2	chr12-62434522-62436317	0.003304273
TAFA2	chr12-62933798-62935422	0.003302877
TAFA2	chr12-62601740-62605279	0.003301082
TAFA2	chr12-62465204-62468071	0.003300484

C4orf33	chr4-128552760-128553761	0.003255663
C4orf33	chr4-128286157-128289987	0.003254992
C4orf33	chr4-128809135-128813086	0.003252257
C4orf33	chr4-129326807-129328641	0.003251676
C4orf33	chr4-128060175-128062770	0.003251239
RFX3-AS1	chr9-2843000-2845147	0.003248441
C4orf33	chr4-127963426-127966639	0.003247436
C4orf33	chr4-128916471-128918299	0.00324723
C4orf33	chr4-129095586-129096772	0.003246847
C4orf33	chr4-129091353-129094826	0.003244685
RFX3-AS1	chr9-3397672-3398966	0.003242374
RFX3-AS1	chr9-4661239-4668290	0.003236178
RFX3-AS1	chr9-3507371-3509170	0.003236148
RFX3-AS1	chr9-4678482-4681465	0.003235372
RFX3-AS1	chr9-4596020-4597742	0.003234664
RFX3-AS1	chr9-4296824-4300401	0.00323242
RFX3-AS1	chr9-3524097-3529230	0.003232385
BCKDHB	chr6-79536504-79538189	0.003228823
BCKDHB	chr6-79233306-79235291	0.003227762
BCKDHB	chr6-79544186-79545555	0.003223512
BCKDHB	chr6-79075954-79079414	0.003222157
BCKDHB	chr6-79777554-79778641	0.003214662
DOK6	chr18-68714167-68715780	0.003210147
DOK6	chr18-68721448-68723280	0.003209774
BCKDHB	chr6-79869500-79870233	0.003207271
APBA2	chr15-28094151-28097393	0.003205387
DOK6	chr18-69895446-69899404	0.003204558
BCKDHB	chr6-79946261-79948126	0.003204133
BCKDHB	chr6-80003391-80005248	0.003204003
BCKDHB	chr6-80105972-80107430	0.003203085
APBA2	chr15-28936857-28938856	0.003202577
APBA2	chr15-28941411-28943496	0.003199545
APBA2	chr15-28890196-28891618	0.003199318
APBA2	chr15-28737427-28739368	0.003197817
DOK6	chr18-69934769-69936618	0.003195967
APBA2	chr15-28884919-28887917	0.003194499
APBA2	chr15-28831752-28833018	0.003193436
APBA2	chr15-28947606-28949534	0.003190028
DOK6	chr18-69944423-69949731	0.003188477
DOK6	chr18-70288401-70289973	0.003187195
DOK6	chr18-70205078-70206684	0.003186624
DOK6	chr18-69954591-69960134	0.003185584
DOK6	chr18-70421698-70422757	0.003182823
AC012447.1	chr2-120343255-120347209	0.003180801
APBA2	chr15-29268546-29271080	0.00317885
RPS12	chr6-131627431-131629202	0.003176677
RPS12	chr6-132510684-132514451	0.003175476
RPS12	chr6-132713293-132714592	0.003174078
AC012447.1	chr2-121197103-121198402	0.003173751
ST8SIA4	chr5-100534552-100536404	0.003170079

RPS12	chr6-132733437-132735401	0.003169557
ST8SIA4	chr5-100666980-100668002	0.003166217
ST8SIA4	chr5-100706612-100707420	0.00316549
ST8SIA4	chr5-100777456-100778384	0.003164725
RPS12	chr6-132762539-132764074	0.003162303
EIPR1	chr2-2613121-2614118	0.003161937
RPS12	chr6-133951338-133954643	0.003161855
AC012447.1	chr2-121286831-121287924	0.003161697
ST8SIA4	chr5-100779621-100781011	0.003160456
RPS12	chr6-132817674-132821274	0.003157278
RNF125	chr18-32040309-32041569	0.003156396
RNF125	chr18-32017467-32021002	0.003156236
RPS12	chr6-132797828-132799772	0.003155901
AC012447.1	chr2-121735491-121739353	0.003155493
MEF2C-AS1	chr5-88824777-88830172	0.003155319
SMC6	chr2-18559430-18561381	0.003155076
AC012447.1	chr2-121754806-121756446	0.003154226
RPS12	chr6-132811763-132817060	0.003154117
MEF2C-AS1	chr5-88679848-88681013	0.003154009
ST8SIA4	chr5-100840271-100841928	0.003152463
AC012447.1	chr2-121698562-121701281	0.003151464
SCLT1	chr4-128552760-128553761	0.003150874
RNF125	chr18-31941817-31944663	0.003150865
MEF2C-AS1	chr5-88881430-88884878	0.003150791
SCLT1	chr4-128286157-128289987	0.003150226
EIPR1	chr2-2646056-2647555	0.003150186
SMC6	chr2-17752062-17755099	0.003149946
AC012447.1	chr2-121529668-121533174	0.003149882
RNF125	chr18-32048383-32051485	0.003149404
MEF2C-AS1	chr5-88674246-88676428	0.003148967
SCLT1	chr4-128809135-128813086	0.003147576
ST8SIA4	chr5-100898704-100905766	0.003147376
SCLT1	chr4-129326807-129328641	0.003147015
SCLT1	chr4-128060175-128062770	0.003146595
RNF125	chr18-31658412-31659635	0.00314616
RNF125	chr18-31684070-31686209	0.003145951
AC012447.1	chr2-121648099-121651074	0.003145706
ST8SIA4	chr5-100852771-100853660	0.003144714
MEF2C-AS1	chr5-88268099-88269966	0.003144684
MEF2C-AS1	chr5-88140730-88142716	0.00314429
AC012447.1	chr2-121596174-121597302	0.003144005
SMC6	chr2-16608537-16609526	0.003143565
SCLT1	chr4-127963426-127966639	0.003142912
SMC6	chr2-16624913-16627467	0.00314277
SCLT1	chr4-128916471-128918299	0.00314271
MEF2C-AS1	chr5-89293523-89294462	0.003142605
SMC6	chr2-16861085-16862996	0.003142493
ST8SIA4	chr5-100889006-100891289	0.003142364
SCLT1	chr4-129095586-129096772	0.003142341
EIPR1	chr2-3222477-3223633	0.003141142

SCLT1	chr4-129091353-129094826	0.003140247
RNF125	chr18-32084559-32087655	0.003138451
SMC6	chr2-16630081-16632126	0.003137847
EIPR1	chr2-3242398-3243508	0.003137335
EIPR1	chr2-3376992-3380739	0.003136862
IL1RAP	chr3-190231353-190232454	0.003135604
EIPR1	chr2-3518116-3520718	0.003135368
IL1RAP	chr3-190512855-190515642	0.003135355
MEF2C-AS1	chr5-89302594-89303633	0.003135244
SMC6	chr2-16662479-16667328	0.003134886
GASK1B	chr4-158816961-158818833	0.003133997
IL1RAP	chr3-191328603-191330615	0.003132493
SMC6	chr2-16649338-16651397	0.003132436
GASK1B	chr4-158935859-158936834	0.003132347
MEF2C-AS1	chr5-89396263-89397584	0.003131786
IL1RAP	chr3-190565990-190567125	0.00313112
GASK1B	chr4-158809300-158812504	0.003131099
SMC6	chr2-16653069-16660704	0.003130878
IL1RAP	chr3-189355301-189356465	0.003130437
EIPR1	chr2-3557414-3559624	0.003130417
IL1RAP	chr3-191245731-191246861	0.003129447
RNF125	chr18-32091135-32099671	0.003129249
GASK1B	chr4-159101098-159106324	0.003128768
ST6GALNAC3	chr1-77218646-77220059	0.003127263
IL1RAP	chr3-190585551-190587807	0.003127062
IL1RAP	chr3-190616457-190620134	0.003126574
GASK1B	chr4-159177533-159179934	0.003126083
RNF125	chr18-32470048-32471718	0.003125383
GASK1B	chr4-158766199-158770757	0.003123903
EIPR1	chr2-3574447-3576528	0.003123475
IL1RAP	chr3-189323804-189325452	0.00312255
ST6GALNAC3	chr1-76294343-76295489	0.00312071
EIPR1	chr2-3650403-3652557	0.003118611
ST6GALNAC3	chr1-76153899-76155279	0.003118357
ST6GALNAC3	chr1-76073982-76076615	0.0031173
ST6GALNAC3	chr1-76267444-76268498	0.003116421
ST6GALNAC3	chr1-76270199-76271253	0.003116372
GASK1B	chr4-158722248-158724526	0.003116116
XRCC4	chr5-83471006-83474938	0.003115323
GASK1B	chr4-158208836-158210793	0.003113974
GASK1B	chr4-158670698-158673254	0.003112228
CWC27	chr5-64766945-64769461	0.003111503
ST6GALNAC3	chr1-75784268-75787959	0.003110513
CWC27	chr5-65924438-65928648	0.003109639
TTC39B	chr9-15551918-15554680	0.003109286
TTC39B	chr9-15509868-15513815	0.003108346
CD226	chr18-69895446-69899404	0.003106391
XRCC4	chr5-82173934-82175010	0.003106185
XRCC4	chr5-82277158-82279373	0.003105426
XRCC4	chr5-83368092-83369612	0.00310528

CWC27	chr5-65034362-65038290	0.003105097
CWC27	chr5-65480101-65484168	0.003104958
XRCC4	chr5-81970699-81973287	0.003104708
CWC27	chr5-65562138-65564136	0.003104311
CWC27	chr5-65102346-65104067	0.003103945
TTC39B	chr9-14692743-14693960	0.003103276
TTC39B	chr9-15421599-15423996	0.003103066
TTC39B	chr9-14322064-14323199	0.003102271
CWC27	chr5-65845028-65846033	0.003101303
CWC27	chr5-65623492-65625669	0.003101245
XRCC4	chr5-82356743-82357918	0.003100495
AASDHPPT	chr11-106021127-106023277	0.003100483
TTC39B	chr9-14991983-14994537	0.003100348
ST6GALNAC3	chr1-75745155-75745851	0.003100135
AASDHPPT	chr11-106076315-106079157	0.003099715
MEF2C	chr5-88824777-88830172	0.003098907
CWC27	chr5-65719115-65723523	0.003098895
CD226	chr18-69934769-69936618	0.00309806
MEF2C	chr5-88679848-88681013	0.003097622
AASDHPPT	chr11-105181100-105182190	0.00309709
TTC39B	chr9-15304530-15308146	0.003096895
TET2	chr4-106314691-106318088	0.003096142
TTC39B	chr9-15293172-15297689	0.003095908
XRCC4	chr5-83312328-83313705	0.003095752
SMAD5	chr5-135999090-136000035	0.003095335
MEF2C	chr5-88881430-88884878	0.003094458
XRCC4	chr5-83076564-83078352	0.003094158
TTC39B	chr9-15298732-15300645	0.003094017
SMAD5	chr5-136011744-136013003	0.003093801
SMAD5	chr5-135455819-135457898	0.003092932
SMAD5	chr5-136132037-136134091	0.003092883
MEF2C	chr5-88674246-88676428	0.003092669
SMAD5	chr5-136028597-136029694	0.00309184
XRCC4	chr5-83306386-83307448	0.003091542
ST6GALNAC3	chr1-75723930-75725283	0.003091275
CD226	chr18-69944423-69949731	0.003090801
CD226	chr18-70288401-70289973	0.003089561
CD226	chr18-70205078-70206684	0.003089006
MEF2C	chr5-88268099-88269966	0.003088462
MEF2C	chr5-88140730-88142716	0.003088075
AASDHPPT	chr11-105099655-105101708	0.003088071
CD226	chr18-69954591-69960134	0.003087998
TET2	chr4-105707788-105709622	0.003086464
MEF2C	chr5-89293523-89294462	0.003086419
SMAD5	chr5-135397776-135400236	0.003086182
CD226	chr18-70421698-70422757	0.003085323
APP	chr21-26965441-26967975	0.003081198
RAPGEF2	chr4-158816961-158818833	0.003079844
MEF2C	chr5-89302594-89303633	0.00307919
TET2	chr4-105469717-105474694	0.003079051

RAPGEF2	chr4-158935859-158936834	0.00307822
SMAD5	chr5-135391585-135394158	0.003078044
APP	chr21-26168356-26171441	0.003077888
RAPGEF2	chr4-158809300-158812504	0.003076994
AASDHPPT	chr11-105043635-105046202	0.003076362
MEF2C	chr5-89396263-89397584	0.003075795
CD226	chr18-70658848-70659841	0.003075576
RAPGEF2	chr4-159101098-159106324	0.003074701
APP	chr21-25967185-25968449	0.003074606
TET2	chr4-105196216-105197177	0.003074367
SULT1B1	chr4-70901550-70903543	0.003073692
APP	chr21-25733501-25736674	0.003073134
APP	chr21-25638644-25640633	0.003072505
SMAD5	chr5-135385567-135387852	0.003072086
RAPGEF2	chr4-159177533-159179934	0.003072061
SULT1B1	chr4-69830716-69832924	0.003071934
SULT1B1	chr4-70891971-70892916	0.003070902
SULT1B1	chr4-69760043-69761370	0.003070677
APP	chr21-25606856-25608940	0.003070389
TET2	chr4-105144405-105148460	0.003070284
RAPGEF2	chr4-158766199-158770757	0.003069919
AASDHPPT	chr11-104876195-104877801	0.003069594
SMAD5	chr5-135139785-135141398	0.003069129
AASDHPPT	chr11-105032436-105036161	0.003067144
SULT1B1	chr4-70687279-70689481	0.003066875
AASDHPPT	chr11-104916108-104918987	0.003065866
APP	chr21-25572695-25574322	0.003065863
ACADM	chr1-76294343-76295489	0.003065552
TET2	chr4-105057185-105062171	0.003064843
CD226	chr18-70672083-70673451	0.003063968
AASDHPPT	chr11-104967422-104969684	0.003063843
SULT1B1	chr4-70838459-70841408	0.00306359
ACADM	chr1-76153899-76155279	0.003063242
RAPGEF2	chr4-158722248-158724526	0.003062265
ACADM	chr1-76073982-76076615	0.003062202
ACADM	chr1-76267444-76268498	0.003061337
ACADM	chr1-76270199-76271253	0.003061291
APP	chr21-25561345-25564836	0.003061001
RAPGEF2	chr4-158208836-158210793	0.003060158
APP	chr21-25456372-25458052	0.003059402
SULT1B1	chr4-70703621-70706843	0.003059091
TET2	chr4-104966324-104967613	0.003058887
RAPGEF2	chr4-158670698-158673254	0.003058443
TET2	chr4-104490717-104491965	0.003057385
GLT1D1	chr12-128857052-128857968	0.003056724
SULT1B1	chr4-70733991-70736341	0.003056363
ACADM	chr1-75784268-75787959	0.003055536
TET2	chr4-104930480-104931543	0.003055535
SULT1B1	chr4-70712871-70714431	0.003054399
GLT1D1	chr12-128793786-128797923	0.0030527

GLT1D1	chr12-128782287-128783970	0.003052003
GLT1D1	chr12-128852438-128854813	0.003049746
FAR2	chr12-28189375-28192265	0.003049109
CEP162	chr6-84226911-84228623	0.003048782
GLT1D1	chr12-128806817-128807890	0.003048337
SGO1-AS1	chr3-19146522-19148702	0.003047949
SGO1-AS1	chr3-20185004-20187983	0.003046316
ACADM	chr1-75745155-75745851	0.00304534
CEP162	chr6-83430463-83431546	0.003044391
SGO1-AS1	chr3-20125650-20126955	0.003043778
CEP162	chr6-83192475-83194650	0.003042263
CEP162	chr6-83467356-83468378	0.003042162
RTTN	chr18-69895446-69899404	0.003041601
FAR2	chr12-28799271-28800632	0.003041034
SGO1-AS1	chr3-19930941-19932308	0.003040671
GLT1D1	chr12-128810013-128812198	0.003040252
CCDC6	chr10-60777818-60779450	0.003039711
GLT1D1	chr12-128843205-128844599	0.003039685
CCDC6	chr10-60731197-60734091	0.003039009
CEP162	chr6-83067132-83068621	0.003038895
CEP162	chr6-83065025-83066550	0.003038471
SGO1-AS1	chr3-20103758-20104838	0.00303714
CEP162	chr6-83867315-83868263	0.003036974
CEP162	chr6-83590067-83590969	0.003036756
ACADM	chr1-75723930-75725283	0.003036636
LRRN3	chr7-111081521-111084085	0.003035851
CCDC6	chr10-60942700-60945053	0.00303547
LRRN3	chr7-111137676-111138709	0.003035098
LRRN3	chr7-111024868-111025735	0.003034625
LRRN3	chr7-111012781-111014116	0.00303427
ACADM	chr1-74732271-74734295	0.003033513
RTTN	chr18-69934769-69936618	0.003033445
GLT1D1	chr12-128820219-128825073	0.00303338
CEP162	chr6-83859101-83861704	0.003033298
GLT1D1	chr12-128829325-128831414	0.003032867
CCDC6	chr10-60727688-60728745	0.003032198
SGO1-AS1	chr3-19945916-19948818	0.003030883
LRRN3	chr7-111561053-111563144	0.003030165
FAR2	chr12-29102704-29105214	0.003028967
VNN2	chr6-131627431-131629202	0.00302894
SGO1-AS1	chr3-20060514-20061774	0.00302838
VNN2	chr6-132510684-132514451	0.003027794
VNN2	chr6-132713293-132714592	0.003026461
RTTN	chr18-69944423-69949731	0.003026336
PPP2R2B	chr5-146446484-146448493	0.003025427
PPP2R2B	chr5-146202457-146206009	0.003025353
RTTN	chr18-70288401-70289973	0.003025119
RTTN	chr18-70205078-70206684	0.003024578
RTTN	chr18-69954591-69960134	0.003023591
SGO1-AS1	chr3-20038434-20044581	0.00302341

SGO1-AS1	chr3-20054386-20055529	0.003022485
CCDC6	chr10-60666045-60666738	0.003022391
VNN2	chr6-132733437-132735401	0.003022149
LRRN3	chr7-111986960-111988186	0.003022102
PPP2R2B	chr5-146181389-146183955	0.00302153
RTTN	chr18-70421698-70422757	0.00302097
TFEC	chr7-116952802-116954801	0.003020353
PPP2R2B	chr5-146751515-146752494	0.003019027
FAR2	chr12-29148055-29150369	0.003017874
LRRN3	chr7-112203993-112207663	0.003015939
VNN2	chr6-132762539-132764074	0.003015232
LRRN3	chr7-112080242-112082630	0.003014959
VNN2	chr6-133951338-133954643	0.003014805
CCDC6	chr10-60236621-60238919	0.00301425
FAR2	chr12-29427909-29429205	0.003013993
FAR2	chr12-29782331-29784388	0.00301347
FAR2	chr12-29380079-29382033	0.003013028
LRRN3	chr7-112083967-112085223	0.00301272
TFEC	chr7-116861308-116864260	0.003011999
FAR2	chr12-29164305-29165460	0.003011811
RTTN	chr18-70658848-70659841	0.003011429
KAT2B	chr3-19146522-19148702	0.003011326
FAR2	chr12-29241785-29243441	0.003011184
VNN2	chr6-132817674-132821274	0.003010442
CCDC6	chr10-60031489-60033243	0.003010219
KAT2B	chr3-20185004-20187983	0.003009713
VNN2	chr6-132797828-132799772	0.003009128
TFEC	chr7-116842369-116844737	0.003008827
CCDC6	chr10-59991447-59992317	0.003008739
TFEC	chr7-116524463-116526909	0.003008681
TFEC	chr7-116858880-116860038	0.003008537
PPP2R2B	chr5-146877191-146879204	0.00300826
VNN2	chr6-132811763-132817060	0.003007428
KAT2B	chr3-20125650-20126955	0.003007205
CCDC6	chr10-59903892-59908895	0.003006373
TMEM144	chr4-158816961-158818833	0.003005008
TFEC	chr7-116209499-116215965	0.003004821
KAT2B	chr3-19930941-19932308	0.003004136
TMEM144	chr4-158935859-158936834	0.003003426
TMEM144	chr4-158809300-158812504	0.003002231
KAT2B	chr3-20103758-20104838	0.003000645
RTTN	chr18-70672083-70673451	0.003000065
TMEM144	chr4-159101098-159106324	0.002999993
TFEC	chr7-116165533-116166597	0.002998046
PPP2R2B	chr5-147481383-147483181	0.002997995
TMEM144	chr4-159177533-159179934	0.002997418
TMEM144	chr4-158766199-158770757	0.00299533
KAT2B	chr3-19945916-19948818	0.002994468
TFEC	chr7-115008382-115010181	0.002993563
TFEC	chr7-115410360-115411648	0.002992845

KAT2B	chr3-20060514-20061774	0.002991991
PPP2R2B	chr5-147782120-147783148	0.002991843
PPP2R2B	chr5-147804642-147805797	0.002989532
PPP2R2B	chr5-148186090-148186994	0.002988181
TMEM144	chr4-158722248-158724526	0.002987864
KAT2B	chr3-20038434-20044581	0.002987083
KAT2B	chr3-20054386-20055529	0.002986166
TMEM144	chr4-158208836-158210793	0.002985808
TMEM144	chr4-158670698-158673254	0.002984134
MB21D2	chr3-192892749-192894199	0.002959589
MB21D2	chr3-194069782-194071725	0.002959184
MB21D2	chr3-194002946-194004161	0.002958371
MB21D2	chr3-194088174-194090214	0.002958064
MB21D2	chr3-194103333-194104900	0.002957118
MB21D2	chr3-192916343-192918574	0.002956968
MB21D2	chr3-193841839-193842978	0.002955696
MB21D2	chr3-192957044-192958300	0.002954052
MB21D2	chr3-193592517-193594238	0.002953544
L3MBTL4	chr18-5236988-5239159	0.002933958
L3MBTL4	chr18-5293869-5297766	0.002931743
L3MBTL4	chr18-5309755-5310782	0.002925549
L3MBTL4	chr18-5455514-5457814	0.002918408
L3MBTL4	chr18-5542591-5544395	0.002918335
L3MBTL4	chr18-6413657-6415410	0.002917486
L3MBTL4	chr18-5514989-5516324	0.002916916
L3MBTL4	chr18-7566405-7568752	0.002916423
L3MBTL4	chr18-5466065-5467033	0.002914459
L3MBTL4	chr18-5462074-5464974	0.00291425
FRMD4B	chr3-69738333-69740793	0.002902495
FRMD4B	chr3-69352255-69354179	0.002900817
FRMD4B	chr3-69320701-69322039	0.002895824
EPHA4	chr2-221517084-221519218	0.002889841
EPHA4	chr2-221519757-221521575	0.002889659
EPHA4	chr2-221570305-221574363	0.002889178
FRMD4B	chr3-69306174-69307041	0.002888622
FRMD4B	chr3-69077995-69089586	0.002885991
EPHA4	chr2-222304263-222306538	0.002885491
FILIP1L	chr3-99816809-99819184	0.0028854
FRMD4B	chr3-69090966-69093002	0.002884069
FRMD4B	chr3-69057873-69059354	0.002883133
FRMD4B	chr3-69197801-69200756	0.002882938
FRMD4B	chr3-69093670-69096276	0.002881674
FILIP1L	chr3-99875394-99876957	0.002881656
FILIP1L	chr3-99895215-99896820	0.002878348
EPHA4	chr2-222317959-222320676	0.002878083
FILIP1L	chr3-100708374-100711277	0.002876612
EPHA4	chr2-222707364-222709978	0.002876162
FILIP1L	chr3-100047030-100048542	0.002873341
EPHA4	chr2-222690465-222691079	0.002872069
FILIP1L	chr3-100602491-100603941	0.002871851

EPHA4	chr2-222423571-222426569	0.002869911
EPHA4	chr2-222667751-222668901	0.002866877
FILIP1L	chr3-100260001-100261885	0.002866776
EPHA4	chr2-222654957-222657243	0.002865449
FILIP1L	chr3-100491971-100493008	0.00286539
FRMD4B	chr3-69012481-69014375	0.002863453
BASP1	chr5-16464383-16467758	0.002862843
FILIP1L	chr3-100333948-100335893	0.002861626
AC027018.1	chr8-73990749-73992970	0.002861219
FILIP1L	chr3-100399756-100402521	0.00286102
AC027018.1	chr8-74051837-74052796	0.002859612
AC027018.1	chr8-73975277-73977053	0.002858345
BASP1	chr5-16615630-16618672	0.002858044
STK3	chr8-97774778-97777125	0.002857185
AC027018.1	chr8-73292669-73296208	0.002855335
AC027018.1	chr8-73745694-73748647	0.00285469
AC027018.1	chr8-73970927-73973322	0.002854622
AC027018.1	chr8-73876852-73880876	0.002853377
AC027018.1	chr8-74349971-74351063	0.002852891
STK3	chr8-98043687-98046572	0.002851893
AC027018.1	chr8-73167109-73168955	0.002851766
FAM198B-AS1	chr4-158816961-158818833	0.002850965
FAM198B-AS1	chr4-158935859-158936834	0.002849463
BASP1	chr5-16624783-16627881	0.002849362
FAM198B-AS1	chr4-158809300-158812504	0.002848327
FAM198B-AS1	chr4-159101098-159106324	0.002846207
STK3	chr8-99862034-99863260	0.002843837
AC027018.1	chr8-73008102-73010072	0.002843825
FAM198B-AS1	chr4-159177533-159179934	0.002843765
STK3	chr8-98116422-98118058	0.002843565
STK3	chr8-99892766-99894490	0.002843204
FAM198B-AS1	chr4-158766199-158770757	0.00284178
UBE3D	chr6-84226911-84228623	0.002841507
PTPRE	chr10-129035249-129037461	0.002841144
PTPRE	chr10-129057276-129058719	0.002839906
STK3	chr8-99012337-99014254	0.002839484
PTPRE	chr10-128209895-128212562	0.002839228
USP45	chr6-99602558-99605535	0.002838456
BASP1	chr5-16628835-16630374	0.002837901
USP45	chr6-99612946-99615113	0.002837565
UBE3D	chr6-83430463-83431546	0.002837416
FAM198B-AS1	chr4-156970536-156972421	0.002837336
USP45	chr6-99588477-99589690	0.002836526
UBE3D	chr6-82246424-82248890	0.002835434
UBE3D	chr6-83192475-83194650	0.002835431
UBE3D	chr6-83467356-83468378	0.002835337
PTPRE	chr10-128149405-128150725	0.002835116
FAM198B-AS1	chr4-158722248-158724526	0.002834695
STK3	chr8-98293093-98294641	0.002834012
PTPRE	chr10-127905828-127908021	0.002833984

PTPRE	chr10-128011100-128012116	0.002833601
FAM198B-AS1	chr4-158208836-158210793	0.002832746
UBE3D	chr6-83067132-83068621	0.00283229
STK3	chr8-98947207-98949580	0.002832203
PTPRE	chr10-128124353-128127673	0.002831975
UBE3D	chr6-83065025-83066550	0.002831894
PTPRE	chr10-128045032-128071717	0.002831878
FAM198B-AS1	chr4-158670698-158673254	0.002831158
PTPRE	chr10-127880373-127881329	0.002830977
USP45	chr6-99567811-99569550	0.002830616
UBE3D	chr6-83867315-83868263	0.002830502
UBE3D	chr6-83590067-83590969	0.002830299
SATB1-AS1	chr3-17739393-17743649	0.002829945
NTPCR	chr1-232628911-232631268	0.002828577
SATB1-AS1	chr3-18438290-18440840	0.002828512
SATB1-AS1	chr3-18422855-18427169	0.002828388
NTPCR	chr1-232620179-232621509	0.002828139
SATB1-AS1	chr3-18401527-18403041	0.002828023
BASP1	chr5-16935373-16937037	0.002827606
STK3	chr8-98825018-98826586	0.002827308
ZNF254	chr19-24032541-24034596	0.002827145
UBE3D	chr6-83859101-83861704	0.002827076
STK3	chr8-98939398-98946591	0.002826807
BASP1	chr5-17436074-17437385	0.002826692
SATB1-AS1	chr3-18442745-18446661	0.002826427
LARP1B	chr4-128552760-128553761	0.002826246
BASP1	chr5-17316739-17319018	0.002826087
PTPRE	chr10-127293370-127294201	0.002826057
LARP1B	chr4-128286157-128289987	0.002825663
ZNF254	chr19-24086570-24088134	0.002825314
NTPCR	chr1-232589742-232591271	0.002825055
NTPCR	chr1-231803195-231804275	0.002824278
ZNF254	chr19-23914243-23915514	0.002824174
BASP1	chr5-17255191-17256333	0.002824082
RETREG1	chr5-16464383-16467758	0.002823944
NTPCR	chr1-232804830-232806476	0.002823503
LARP1B	chr4-128809135-128813086	0.002823288
USP45	chr6-99520712-99522098	0.00282286
SATB1-AS1	chr3-18723157-18727719	0.00282271
LARP1B	chr4-128060175-128062770	0.002822407
BASP1	chr5-17118260-17119631	0.00282204
BASP1	chr5-17216048-17219434	0.002821711
SATB1-AS1	chr3-18738462-18742321	0.002819531
RETREG1	chr5-16615630-16618672	0.00281921
LARP1B	chr4-127963426-127966639	0.002819106
LARP1B	chr4-128916471-128918299	0.002818922
LARP1B	chr4-129095586-129096772	0.002818591
SATB1-AS1	chr3-18743744-18746116	0.002818329
SATB1-AS1	chr3-18757709-18758583	0.002817873
MITF	chr3-69738333-69740793	0.002817759

LARP1B	chr4-127879989-127881903	0.002817618
LARP1B	chr4-127781605-127783346	0.002817538
USP45	chr6-99514610-99516432	0.002816842
LARP1B	chr4-129091353-129094826	0.002816713
ZNF254	chr19-23799143-23800719	0.002816616
MITF	chr3-69352255-69354179	0.002816127
SATB1-AS1	chr3-19146522-19148702	0.002815156
NTPCR	chr1-232923560-232924677	0.002814575
USP45	chr6-99423093-99426515	0.002814306
USP45	chr6-99393518-99394605	0.002813495
MITF	chr3-69320701-69322039	0.002811283
USP45	chr6-98946665-98948601	0.002810855
RETREG1	chr5-16624783-16627881	0.002810646
ZNF254	chr19-23762297-23764061	0.002808187
ZNF254	chr19-23272814-23274928	0.00280757
ZNF254	chr19-23394077-23396223	0.002806958
NTPCR	chr1-232949922-232951695	0.002806308
USP45	chr6-98831094-98836179	0.002804317
MITF	chr3-69306174-69307041	0.002804291
ZNF254	chr19-23686025-23688170	0.002803949
ZNF254	chr19-23758055-23760803	0.002803441
ZNF254	chr19-23249276-23250822	0.002802886
EPB41L3	chr18-5236988-5239159	0.002802838
NTPCR	chr1-233326765-233329082	0.002802278
NTPCR	chr1-233112461-233115425	0.002802211
NTPCR	chr1-233294816-233296040	0.002802003
EPB41L3	chr18-5057675-5059596	0.002801764
MITF	chr3-69077995-69089586	0.002801736
MCTP2	chr15-93025014-93031278	0.00280139
EPB41L3	chr18-5293869-5297766	0.002800723
MITF	chr3-69090966-69093002	0.002799869
RETREG1	chr5-16628835-16630374	0.002799341
MITF	chr3-69057873-69059354	0.002798959
MITF	chr3-69197801-69200756	0.002798772
MITF	chr3-69093670-69096276	0.002797544
EPB41L3	chr18-5309755-5310782	0.002794803
MCTP2	chr15-93861948-93863753	0.002794547
AC120193.1	chr8-24271584-24272597	0.002794303
AC120193.1	chr8-23761180-23762224	0.002794286
MCTP2	chr15-93235845-93236904	0.002793314
MCTP2	chr15-93035684-93038166	0.0027928
AC120193.1	chr8-24293685-24294383	0.0027925
MCTP2	chr15-94172882-94175263	0.002790994
LINC01184	chr5-129093930-129095778	0.002790326
RETREG1	chr5-16935373-16937037	0.002789185
MCTP2	chr15-93178680-93179659	0.002788512
RETREG1	chr5-17436074-17437385	0.002788283
EPB41L3	chr18-5455514-5457814	0.002787981
EPB41L3	chr18-5542591-5544395	0.00278791
RETREG1	chr5-17316739-17319018	0.002787689

EPB41L3	chr18-6413657-6415410	0.002787098
ZNF827	chr4-145617990-145621002	0.002786792
AC120193.1	chr8-24349155-24350936	0.002786697
EPB41L3	chr18-5514989-5516324	0.002786554
MCTP2	chr15-93047305-93049340	0.002786012
MCTP2	chr15-94228949-94232932	0.002785803
RETREG1	chr5-17255191-17256333	0.00278571
MCTP2	chr15-93087765-93089531	0.002784682
ZNF827	chr4-145481434-145483201	0.002784641
ZNF827	chr4-145732640-145733906	0.002784491
EPB41L3	chr18-5466065-5467033	0.002784205
EPB41L3	chr18-5462074-5464974	0.002784007
RETREG1	chr5-17118260-17119631	0.002783696
MCTP2	chr15-94287482-94288479	0.002783665
RETREG1	chr5-17216048-17219434	0.00278337
ZNF827	chr4-145096968-145100186	0.002781718
ZNF827	chr4-145176965-145181494	0.002781328
LINC01184	chr5-128537218-128539090	0.002781294
LINC00667	chr18-5236988-5239159	0.002780602
AC120193.1	chr8-25457202-25459813	0.002780123
MITF	chr3-69012481-69014375	0.002779852
LINC00667	chr18-5057675-5059596	0.002779536
LINC01184	chr5-127516968-127518638	0.002778808
ZNF827	chr4-145935533-145939662	0.002778553
LINC00667	chr18-5293869-5297766	0.002778504
LINC01184	chr5-127228855-127230081	0.002778315
TSTD3	chr6-99602558-99605535	0.002777963
AC120193.1	chr8-24386291-24388290	0.002777631
TSTD3	chr6-99612946-99615113	0.002777093
LINC01184	chr5-128082022-128085243	0.002776584
LINC01184	chr5-128196652-128198484	0.002776386
TSTD3	chr6-99588477-99589690	0.002776076
AC120193.1	chr8-25242704-25244534	0.002775161
LINC01184	chr5-127072727-127074059	0.002772687
ZNF827	chr4-146104757-146106445	0.002772672
LINC00667	chr18-5309755-5310782	0.002772632
CDK14	chr7-90244669-90245997	0.002771267
ZNF827	chr4-146945415-146946248	0.002770805
ERCC8	chr5-60520446-60522851	0.002770338
TSTD3	chr6-99567811-99569550	0.00277029
ZNF827	chr4-146520771-146523272	0.002770217
ZNF827	chr4-146175115-146177300	0.002769934
AC120193.1	chr8-25183607-25186698	0.002769221
EXT1	chr8-118089337-118090406	0.002769125
AC120193.1	chr8-25237271-25239876	0.002768771
EXT1	chr8-118060396-118061416	0.002768175
EXT1	chr8-118099104-118100419	0.002767274
ERCC8	chr5-60538366-60540042	0.002767013
ERCC8	chr5-61407728-61409863	0.002766465
ERCC8	chr5-60540820-60542481	0.002766428

EXT1	chr8-118281701-118282624	0.002766363
EXT1	chr8-118278939-118280111	0.002766151
LINC00667	chr18-5455514-5457814	0.002765865
EXT1	chr8-118108415-118113700	0.002765848
AC120193.1	chr8-25198314-25199777	0.002765836
LINC00667	chr18-5542591-5544395	0.002765792
ERCC8	chr5-60699219-60701783	0.002765293
LINC00667	chr18-6413657-6415410	0.002764989
PDE4D	chr5-60520446-60522851	0.002764874
LINC00667	chr18-5514989-5516324	0.002764449
LINC01184	chr5-127028709-127032288	0.002764207
EXT1	chr8-118284808-118285903	0.002763494
CDK14	chr7-90345351-90348194	0.002763399
CDK14	chr7-90599800-90600822	0.002762901
TSTD3	chr6-99520712-99522098	0.0027627
EXT1	chr8-117519237-117521934	0.002762539
LINC00667	chr18-5466065-5467033	0.002762119
ERCC8	chr5-60943398-60946386	0.002761942
LINC00667	chr18-5462074-5464974	0.002761922
CDK14	chr7-90620849-90622128	0.002761867
CDK14	chr7-90594797-90597687	0.002761783
ERCC8	chr5-61329628-61337466	0.002761762
PDE4D	chr5-60538366-60540042	0.002761556
PDE4D	chr5-61407728-61409863	0.002761009
SEMA6A-AS1	chr5-115261829-115263378	0.002760993
PDE4D	chr5-60540820-60542481	0.002760973
CDK14	chr7-90402951-90404542	0.002760783
PDE4D	chr5-60699219-60701783	0.00275984
LINC01184	chr5-126972167-126973654	0.002759249
CDK14	chr7-91164836-91165966	0.002759109
LY96	chr8-73990749-73992970	0.002758518
LINC01184	chr5-126987626-126988700	0.002758279
ERCC8	chr5-61161544-61163610	0.002757767
CDK14	chr7-91325719-91327942	0.002757523
ERCC8	chr5-61319061-61321362	0.002757335
LY96	chr8-74051837-74052796	0.002756968
CDK14	chr7-91263377-91265920	0.002756862
TSTD3	chr6-99514610-99516432	0.00275681
CDK14	chr7-91300069-91300979	0.002756555
EXT1	chr8-118620881-118622656	0.002756506
PDE4D	chr5-60943398-60946386	0.002756496
FAM49A	chr2-17752062-17755099	0.002756371
PDE4D	chr5-61329628-61337466	0.002756315
LY96	chr8-73975277-73977053	0.002755746
ERCC8	chr5-61275209-61277922	0.002755659
TSTD3	chr6-99423093-99426515	0.002754328
EXT1	chr8-117439157-117440595	0.002753773
TSTD3	chr6-99393518-99394605	0.002753533
LY96	chr8-73292669-73296208	0.002752844
PDE4D	chr5-61161544-61163610	0.00275233

LY96	chr8-73745694-73748647	0.002752221
SEMA6A-AS1	chr5-115634166-115635113	0.002752185
LY96	chr8-73970927-73973322	0.002752158
PDE4D	chr5-61319061-61321362	0.002751898
SEMA6A-AS1	chr5-115295761-115297281	0.002751532
LY96	chr8-73876852-73880876	0.002750956
TSTD3	chr6-98946665-98948601	0.002750951
SEMA6A-AS1	chr5-115623717-115627125	0.002750812
FAM49A	chr2-16608537-16609526	0.002750786
LY96	chr8-74349971-74351063	0.002750487
PDE4D	chr5-61275209-61277922	0.002750224
FAM49A	chr2-16624913-16627467	0.002750091
FAM49A	chr2-16861085-16862996	0.002749848
SEMA6A-AS1	chr5-115838729-115839495	0.002749742
LY96	chr8-73167109-73168955	0.002749404
FAM49A	chr2-15591078-15592693	0.002748049
SEMA6A-AS1	chr5-116083973-116086229	0.002747099
SEMA6A-AS1	chr5-115840693-115843837	0.002746686
SEMA6A-AS1	chr5-115601456-115604251	0.002746234
FAM49A	chr2-16630081-16632126	0.002745785
FAM49A	chr2-15560352-15562391	0.002744977
TSTD3	chr6-98831094-98836179	0.002744554
SEMA6A-AS1	chr5-115484097-115485109	0.002744169
FAM49A	chr2-16662479-16667328	0.002743192
SEMA6A-AS1	chr5-115542479-115545576	0.002742626
LY96	chr8-73008102-73010072	0.002741748
FAM49A	chr2-16649338-16651397	0.00274105
GSTCD	chr4-106707723-106709027	0.002739938
FAM49A	chr2-16653069-16660704	0.002739685
NDUFAF2	chr5-60520446-60522851	0.002737343
GSTCD	chr4-106676179-106677035	0.002736585
NDUFAF2	chr5-60538366-60540042	0.002734055
NDUFAF2	chr5-61407728-61409863	0.002733513
NDUFAF2	chr5-60540820-60542481	0.002733478
NDUFAF2	chr5-60699219-60701783	0.002732357
NDUFAF2	chr5-60943398-60946386	0.002729047
GSTCD	chr4-106314691-106318088	0.002729006
NDUFAF2	chr5-61329628-61337466	0.002728867
NDUFAF2	chr5-61161544-61163610	0.002724922
NDUFAF2	chr5-61319061-61321362	0.002724493
NDUFAF2	chr5-61275209-61277922	0.002722838
GSTCD	chr4-105707788-105709622	0.002720476
GSTCD	chr4-105469717-105474694	0.002713942
ACAD11	chr3-131502057-131503767	0.002710197
GSTCD	chr4-105196216-105197177	0.00270981
GSTCD	chr4-105144405-105148460	0.00270621
ACAD11	chr3-132283054-132284188	0.00270468
GSTCD	chr4-105057185-105062171	0.002701416
ACAD11	chr3-132316874-132317941	0.002697613
GSTCD	chr4-104966324-104967613	0.002696166

GSTCD	chr4-104930480-104931543	0.002693209
ACAD11	chr3-132416451-132419400	0.002692618
ACAD11	chr3-132659042-132661510	0.002690215
ACAD11	chr3-132721328-132723610	0.002688039
ACAD11	chr3-133447747-133449152	0.002683625
SLC12A2	chr5-129093930-129095778	0.002681397
ACAD11	chr3-133490391-133493381	0.002676892
SLC12A2	chr5-128537218-128539090	0.002672715
ACAD11	chr3-133505952-133507262	0.00267042
SLC12A2	chr5-127516968-127518638	0.002670327
SLC12A2	chr5-127228855-127230081	0.002669855
ACAD11	chr3-133805109-133806722	0.002668872
SLC12A2	chr5-128082022-128085243	0.002668189
SLC12A2	chr5-128196652-128198484	0.002668
ACAD11	chr3-133661187-133662766	0.002667141
SLC12A2	chr5-127072727-127074059	0.002664445
HDAC9	chr7-17769809-17771357	0.002662506
HDAC9	chr7-17938903-17941598	0.002662334
HDAC9	chr7-17408217-17409012	0.002662244
HDAC9	chr7-17297062-17300384	0.002661104
SRPRB	chr3-132659042-132661510	0.002660683
HDAC9	chr7-18177679-18178788	0.002660368
SRPRB	chr3-132721328-132723610	0.002658532
HDAC9	chr7-17233457-17236226	0.002657818
SLC12A2	chr5-127028709-127032288	0.002656299
BMP2K	chr4-78629209-78631038	0.002654764
SRPRB	chr3-133447747-133449152	0.002654166
HDAC9	chr7-17152466-17153991	0.002652475
BMP2K	chr4-77860772-77864142	0.002651775
SLC12A2	chr5-126972167-126973654	0.002651535
BMP2K	chr4-78644646-78646490	0.002650952
HDAC9	chr7-17008992-17011076	0.002650608
SLC12A2	chr5-126987626-126988700	0.002650603
SRPRB	chr3-133490391-133493381	0.002647507
HDAC9	chr7-17142238-17143589	0.002647366
HDAC9	chr7-17041169-17041921	0.002647046
HDAC9	chr7-17122785-17123663	0.002645277
BMP2K	chr4-77852357-77855525	0.002643407
BMP2K	chr4-78656680-78658989	0.002643238
SRPRB	chr3-134852570-134853539	0.002642322
SRPRB	chr3-133505952-133507262	0.002641104
SRPRB	chr3-133927066-133928143	0.002640502
SRPRB	chr3-134249501-134251511	0.002640126
SRPRB	chr3-134484741-134488287	0.002640093
SRPRB	chr3-133805109-133806722	0.002639573
SRPRB	chr3-133661187-133662766	0.002637861
AC002460.2	chr4-148051949-148053190	0.002637605
ADAMTS6	chr5-66170914-66172368	0.002637444
AC002460.2	chr4-148055989-148057946	0.002636566
BMP2K	chr4-78749358-78750869	0.002636276

FAM241A	chr4-112636355-112638445	0.002635218
BMP2K	chr4-77826329-77828169	0.002634495
AC002460.2	chr4-147831683-147833046	0.002633063
BMP2K	chr4-78774334-78778109	0.002632813
TRPS1	chr8-116471675-116473388	0.002632588
AC002460.2	chr4-148060318-148061480	0.002632241
FAM241A	chr4-112522719-112524649	0.002632156
TRPS1	chr8-115845907-115847270	0.002632069
BMP2K	chr4-78938136-78940242	0.002631956
TRPS1	chr8-116651478-116653216	0.002630326
ADAMTS6	chr5-66142544-66145865	0.002630273
TRPS1	chr8-115665851-115669930	0.002630144
BMP2K	chr4-77817292-77823287	0.00262957
BMP2K	chr4-77800655-77801983	0.002629415
FAM241A	chr4-112514332-112518272	0.002629177
TRPS1	chr8-115427237-115428296	0.002629124
TRPS1	chr8-115449153-115452709	0.002628938
TRPS1	chr8-115647834-115650126	0.002628867
AC002460.2	chr4-148375700-148376663	0.002628666
AC002460.2	chr4-148441432-148446477	0.0026284
TRPS1	chr8-115218488-115219490	0.002628036
TRMT11	chr6-124961663-124964206	0.002627946
TRPS1	chr8-116872296-116875584	0.002627936
TRPS1	chr8-116755074-116756487	0.002626671
TRMT11	chr6-125300883-125303828	0.002625823
TRPS1	chr8-116765469-116767910	0.002624941
FAM241A	chr4-112315358-112316868	0.002624711
AC002460.2	chr4-147799796-147800623	0.00262452
ADAMTS6	chr5-64766945-64769461	0.002622539
TRMT11	chr6-124819101-124819916	0.002622521
ADAMTS6	chr5-65924438-65928648	0.002620965
FAM241A	chr4-112312178-112313331	0.002619486
ADAMTS6	chr5-65034362-65038290	0.002617138
ADAMTS6	chr5-65480101-65484168	0.002617019
ADAMTS6	chr5-65562138-65564136	0.002616473
AC002460.2	chr4-147779440-147780964	0.002616388
ADAMTS6	chr5-65102346-65104067	0.002616165
TRMT11	chr6-125746511-125750680	0.002615961
AC002460.2	chr4-147616719-147618587	0.002615798
FAM241A	chr4-112296806-112298426	0.002615688
FAM241A	chr4-112230715-112232887	0.002615056
FAM241A	chr4-112284063-112289902	0.002614628
FAM241A	chr4-112213081-112215050	0.002614028
ADAMTS6	chr5-65845028-65846033	0.002613937
ADAMTS6	chr5-65623492-65625669	0.002613889
AC002460.2	chr4-147682663-147685186	0.002613174
AC002460.2	chr4-147730774-147733641	0.002612449
ADAMTS6	chr5-65719115-65723523	0.002611907
FAM241A	chr4-112144687-112146982	0.002609703
ENOSF1	chr18-711150-713580	0.002608585

ENOSF1	chr18-810191-814984	0.002606837
ENOSF1	chr18-657221-659001	0.002606224
ENOSF1	chr18-829927-831410	0.002604713
FAM241A	chr4-112084746-112086093	0.002603575
TRMT11	chr6-125779695-125782018	0.002602583
ENOSF1	chr18-614688-616749	0.002598377
TMTC1	chr12-28799271-28800632	0.002597408
TRMT11	chr6-125789068-125792210	0.0025912
RP9	chr7-32941949-32943652	0.002590168
RP9	chr7-33039345-33041923	0.002590088
RP9	chr7-33050001-33051196	0.002589617
RP9	chr7-33061549-33063769	0.002588789
RP9	chr7-32904852-32906570	0.002588189
ENOSF1	chr18-596341-597463	0.002587533
TMTC1	chr12-29102704-29105214	0.002587101
RP9	chr7-33107296-33110254	0.002586186
TRMT11	chr6-125831529-125833127	0.002584631
CENPK	chr5-66170914-66172368	0.002583609
SYNE1	chr6-151493338-151495048	0.002583006
RP9	chr7-32888488-32893574	0.002582575
SYNE1	chr6-151451390-151453469	0.002582407
TRMT11	chr6-125918418-125919778	0.00258163
TMTC1	chr12-30850995-30852115	0.002581233
NIPAL2	chr8-97277326-97278400	0.002581162
RP9	chr7-33128657-33130627	0.002580983
SYNE1	chr6-151497739-151498928	0.002579352
TRMT11	chr6-125955908-125959437	0.002578841
ENOSF1	chr18-463760-464755	0.002578397
ENOSF1	chr18-157862-159728	0.002577731
TMTC1	chr12-29148055-29150369	0.002577626
ENOSF1	chr18-267125-269893	0.002577132
TMTC1	chr12-30753292-30755722	0.002576653
CENPK	chr5-66142544-66145865	0.002576584
NIPAL2	chr8-97637588-97640766	0.002576259
SYNE1	chr6-152309587-152310902	0.002576238
ENOSF1	chr18-322072-323753	0.00257498
SYNE1	chr6-152974968-152975950	0.002574425
TMTC1	chr12-29427909-29429205	0.002574313
ENOSF1	chr18-379466-380527	0.002574306
SYNE1	chr6-152300562-152302764	0.002574279
TRMT11	chr6-125985705-125987744	0.002574247
RP9	chr7-32846506-32847129	0.002574054
TMTC1	chr12-30694804-30696741	0.002574001
SYNE1	chr6-151690155-151691839	0.002573876
TMTC1	chr12-29782331-29784388	0.002573863
TMTC1	chr12-29380079-29382033	0.002573487
NIPAL2	chr8-97642994-97646936	0.002572504
TMTC1	chr12-29164305-29165460	0.002572449
TMTC1	chr12-29241785-29243441	0.002571914
UGGT2	chr13-95300522-95302334	0.002571622

SYNE1	chr6-152182731-152186741	0.002571149
SYNE1	chr6-152170496-152171714	0.002570512
UGGT2	chr13-95188901-95189822	0.0025702
SYNE1	chr6-152982139-152985068	0.002569495
NIPAL2	chr8-97774778-97777125	0.00256926
CENPK	chr5-64766945-64769461	0.002569003
TRMT11	chr6-126339671-126340754	0.002568984
UGGT2	chr13-95478274-95479203	0.002568825
YES1	chr18-711150-713580	0.002568235
CENPK	chr5-65924438-65928648	0.002567465
WWOX	chr16-77189963-77192235	0.002567037
WWOX	chr16-77211646-77213537	0.002566777
YES1	chr18-810191-814984	0.002566513
YES1	chr18-657221-659001	0.002565913
RP9	chr7-32494775-32496648	0.002565844
UGGT2	chr13-95479709-95481116	0.002564891
NIPAL2	chr8-98043687-98046572	0.002564502
SYNE1	chr6-153001854-153004063	0.002564432
YES1	chr18-829927-831410	0.00256442
UGGT2	chr13-95185430-95186527	0.00256437
CENPK	chr5-65034362-65038290	0.002563714
CENPK	chr5-65480101-65484168	0.002563601
CENPK	chr5-65562138-65564136	0.002563068
UGGT2	chr13-95707033-95709193	0.002562781
UGGT2	chr13-95675622-95680362	0.002562768
CENPK	chr5-65102346-65104067	0.002562761
WWOX	chr16-77235910-77236666	0.002562719
UGGT2	chr13-96052624-96053868	0.00256247
RP9	chr7-32489662-32491462	0.002561428
HDDC2	chr6-124961663-124964206	0.002560932
NR3C2	chr4-148051949-148053190	0.002560836
CENPK	chr5-65845028-65846033	0.002560582
CENPK	chr5-65623492-65625669	0.002560535
NR3C2	chr4-148055989-148057946	0.002559826
UGGT2	chr13-97109579-97110499	0.002559158
HDDC2	chr6-125300883-125303828	0.002558862
CENPK	chr5-65719115-65723523	0.002558594
BBS9	chr7-32941949-32943652	0.002558272
BBS9	chr7-33039345-33041923	0.002558192
YES1	chr18-614688-616749	0.002558187
BBS9	chr7-33050001-33051196	0.002557729
NIPAL2	chr8-98116422-98118058	0.002557013
BBS9	chr7-33061549-33063769	0.002556912
NR3C2	chr4-147831683-147833046	0.002556426
BBS9	chr7-32904852-32906570	0.002556316
WWOX	chr16-77721640-77723647	0.002555933
HDDC2	chr6-124819101-124819916	0.002555647
NR3C2	chr4-148060318-148061480	0.002555628
BBS9	chr7-33107296-33110254	0.002554343
NIPAL2	chr8-99012337-99014254	0.002553343

UGGT2	chr13-97209940-97211381	0.00255265
NR3C2	chr4-148375700-148376663	0.002552157
NR3C2	chr4-148441432-148446477	0.002551898
BBS9	chr7-32888488-32893574	0.002550772
GCNT1	chr9-76650675-76651967	0.002550278
WWOX	chr16-78098486-78100740	0.002549409
HDDC2	chr6-125746511-125750680	0.002549251
BBS9	chr7-33128657-33130627	0.002549205
NIPAL2	chr8-98293093-98294641	0.002548422
GCNT1	chr9-77176153-77179502	0.002548361
NR3C2	chr4-147799796-147800623	0.002548131
YES1	chr18-596341-597463	0.002547511
NIPAL2	chr8-98947207-98949580	0.002546796
GCNT1	chr9-76595404-76597072	0.0025464
WWOX	chr16-78742118-78743978	0.002545621
UGGT2	chr13-97221586-97227665	0.002545614
WWOX	chr16-79089508-79091768	0.002544411
WWOX	chr16-79093374-79094359	0.002543637
NIPAL2	chr8-98825018-98826586	0.002542395
BBS9	chr7-32846506-32847129	0.002542356
NIPAL2	chr8-98939398-98946591	0.002541943
NRCAM	chr7-107659699-107662163	0.002541839
WWOX	chr16-79271826-79272826	0.0025414
NRCAM	chr7-107578898-107580985	0.002541088
NR3C2	chr4-147779440-147780964	0.002540235
NR3C2	chr4-147616719-147618587	0.002539663
GCNT1	chr9-76575869-76578256	0.002538981
NRCAM	chr7-107742623-107745007	0.002538522
YES1	chr18-463760-464755	0.002538514
THAP5	chr7-107659699-107662163	0.002538225
WWOX	chr16-79278324-79279528	0.00253808
YES1	chr18-157862-159728	0.002537852
THAP5	chr7-107578898-107580985	0.002537474
YES1	chr18-267125-269893	0.002537265
NR3C2	chr4-147682663-147685186	0.002537114
NR3C2	chr4-147730774-147733641	0.002536411
HDDC2	chr6-125779695-125782018	0.002536213
WWOX	chr16-79286718-79288473	0.002536197
NRCAM	chr7-107563077-107564889	0.002535996
YES1	chr18-322072-323753	0.002535146
THAP5	chr7-107742623-107745007	0.002534911
YES1	chr18-379466-380527	0.002534485
BBS9	chr7-32494775-32496648	0.002534244
NRCAM	chr7-107874553-107876123	0.002534144
AP1AR	chr4-112636355-112638445	0.002533846
NRCAM	chr7-108454530-108457229	0.002532894
NRCAM	chr7-108521696-108527555	0.002532891
THAP5	chr7-107563077-107564889	0.002532388
GCNT1	chr9-76570841-76572130	0.002532037
NRCAM	chr7-108415131-108416967	0.002531849

NRCAM	chr7-107890108-107892515	0.00253171
NRCAM	chr7-108568108-108571687	0.002531607
AP1AR	chr4-112522719-112524649	0.002530902
THAP5	chr7-107874553-107876123	0.002530539
BBS9	chr7-32489662-32491462	0.002529882
NRCAM	chr7-107560202-107561811	0.002529535
THAP5	chr7-108454530-108457229	0.00252929
THAP5	chr7-108521696-108527555	0.002529288
THAP5	chr7-108415131-108416967	0.002528247
THAP5	chr7-107890108-107892515	0.002528107
AP1AR	chr4-112514332-112518272	0.002528038
THAP5	chr7-108568108-108571687	0.002528004
GCNT1	chr9-76458695-76460552	0.002527983
GCNT1	chr9-76403183-76403961	0.002526015
THAP5	chr7-107560202-107561811	0.002525936
HDDC2	chr6-125789068-125792210	0.00252512
AP1AR	chr4-112315358-112316868	0.002523742
GCNT1	chr9-76393349-76395940	0.002523356
AP1AR	chr4-112312178-112313331	0.002518718
HDDC2	chr6-125831529-125833127	0.002518717
GCNT1	chr9-76023426-76024759	0.002518135
HDDC2	chr6-125918418-125919778	0.002515795
AP1AR	chr4-112296806-112298426	0.002515066
AP1AR	chr4-112230715-112232887	0.00251446
AP1AR	chr4-112284063-112289902	0.002514047
AP1AR	chr4-112213081-112215050	0.00251347
HDDC2	chr6-125955908-125959437	0.002513078
GCNT1	chr9-75889549-75893547	0.002511278
AP1AR	chr4-112144687-112146982	0.002509311
HDDC2	chr6-125985705-125987744	0.002508601
BANK1	chr4-101084307-101085954	0.002505873
GCNT1	chr9-75868926-75869886	0.002505712
BANK1	chr4-101327881-101328987	0.002505618
BANK1	chr4-101018723-101021536	0.002504308
RBM43	chr2-152431872-152433753	0.002503891
RBM43	chr2-151260615-151262925	0.002503713
HDDC2	chr6-126339671-126340754	0.002503475
AP1AR	chr4-112084746-112086093	0.002503418
RBM43	chr2-152030723-152032057	0.00250332
RBM43	chr2-152042702-152043557	0.002502874
RBM43	chr2-152420658-152422275	0.002502267
BANK1	chr4-101345160-101349703	0.002501696
RBM43	chr2-152174684-152176915	0.002501317
RBM43	chr2-151826590-151829334	0.002501044
RBM43	chr2-152334833-152336823	0.002500929
RBM43	chr2-151636867-151639053	0.002497055
RBM43	chr2-151286793-151290797	0.002496939
BANK1	chr4-101527078-101527756	0.002495206
RBM43	chr2-151409292-151410920	0.002494689
BANK1	chr4-101790324-101791570	0.002489325

BANK1	chr4-102343722-102346521	0.002486517
BANK1	chr4-102390180-102391173	0.00248642
BANK1	chr4-102430525-102431878	0.002486362
IPO11	chr5-62403012-62404840	0.002486111
BANK1	chr4-102499910-102504978	0.002484014
IPO11	chr5-62305096-62308639	0.002483493
IPO11	chr5-62411687-62413573	0.002482708
BANK1	chr4-102517805-102520157	0.00247989
IPO11	chr5-62299149-62300322	0.002477148
BANK1	chr4-102598097-102599450	0.002477134
IPO11	chr5-62578251-62579110	0.002474691
IPO11	chr5-62291542-62292482	0.002470743
IPO11	chr5-62255978-62257203	0.002466464
IPO11	chr5-62223348-62224585	0.002463755
IPO11	chr5-61407728-61409863	0.002460714
IPO11	chr5-61329628-61337466	0.002456532
ACPP	chr3-131502057-131503767	0.002453626
ACPP	chr3-131386160-131387496	0.002453502
IPO11	chr5-61319061-61321362	0.002452594
IPO11	chr5-61275209-61277922	0.002451103
ACPP	chr3-132283054-132284188	0.002448633
ACPP	chr3-131380743-131382667	0.002447328
ACPP	chr3-132316874-132317941	0.002442236
ACPP	chr3-131360987-131362529	0.002438008
ACPP	chr3-132416451-132419400	0.002437713
INPP4B	chr4-143335529-143338073	0.002436894
ACPP	chr3-132659042-132661510	0.002435538
INPP4B	chr4-142354759-142357246	0.002435219
INPP4B	chr4-143358733-143360042	0.002434833
INPP4B	chr4-143183653-143187122	0.002433572
ACPP	chr3-132721328-132723610	0.002433569
INPP4B	chr4-142404226-142407894	0.00243311
INPP4B	chr4-142416500-142417469	0.002431107
ACPP	chr3-133447747-133449152	0.002429574
INPP4B	chr4-142961230-142962385	0.002428278
INPP4B	chr4-142565505-142568490	0.002428239
INPP4B	chr4-143512512-143515424	0.002427479
INPP4B	chr4-142703638-142704591	0.002425423
INPP4B	chr4-142845306-142847712	0.002425022
ACPP	chr3-133490391-133493381	0.002423478
PDE5A	chr4-118589611-118592588	0.002422348
IMMP2L	chr7-111081521-111084085	0.002421738
IMMP2L	chr7-111137676-111138709	0.002421137
IMMP2L	chr7-111024868-111025735	0.00242076
IMMP2L	chr7-111012781-111014116	0.002420476
INPP4B	chr4-143558757-143560162	0.002418653
ACPP	chr3-133505952-133507262	0.002417618
IMMP2L	chr7-111561053-111563144	0.002417202
RPS13	chr11-17206598-17210051	0.002415556
PDE5A	chr4-118684758-118686097	0.002415492

RPS13	chr11-17229983-17231485	0.002414456
RPS13	chr11-17097662-17099188	0.002412461
IMMP2L	chr7-112449260-112454443	0.002411985
IMMP2L	chr7-111986960-111988186	0.00241077
IMMP2L	chr7-112390703-112392641	0.002410024
IMMP2L	chr7-112461935-112463091	0.002409951
RPS13	chr11-17275279-17279922	0.002409107
RPS13	chr11-17075863-17078939	0.002408664
RPS13	chr11-16737588-16740675	0.002408223
PDE5A	chr4-120066012-120068424	0.002407334
PDE5A	chr4-118834844-118837107	0.002406051
IMMP2L	chr7-112203993-112207663	0.002405851
IMMP2L	chr7-112080242-112082630	0.002405071
NBPF15	chr1-145607630-145608559	0.002404822
IKBIP	chr12-98643663-98647117	0.002404617
IKBIP	chr12-99130881-99132337	0.002404408
RPS13	chr11-18105485-18106575	0.00240362
IMMP2L	chr7-112083967-112085223	0.002403285
RPS13	chr11-17285259-17287554	0.002402807
RPS13	chr11-18277478-18278491	0.00240236
RPS13	chr11-18010093-18014387	0.002402312
PDE5A	chr4-120049601-120050328	0.002400446
RPS13	chr11-17388547-17390255	0.002399807
IKBIP	chr12-98618537-98620149	0.002399756
RPS13	chr11-17351018-17354922	0.002399294
AC010275.1	chr5-111223076-111225350	0.002398229
PDE5A	chr4-118850076-118851193	0.002397602
NBPF15	chr1-145465133-145466006	0.002397003
AC010275.1	chr5-111229050-111231582	0.002396895
AC010275.1	chr5-111091658-111093327	0.002396656
TAFA1	chr3-69077995-69089586	0.002394409
AC010275.1	chr5-111232337-111234818	0.002394117
AL356599.1	chr6-146595833-146596744	0.002393671
IKBIP	chr12-98612512-98613528	0.002393072
PDE5A	chr4-119626927-119629519	0.002392959
TAFA1	chr3-69090966-69093002	0.002392814
PDE5A	chr4-119025375-119027091	0.002392788
AC010275.1	chr5-110738029-110739970	0.002392595
TAFA1	chr3-69057873-69059354	0.002392037
TAFA1	chr3-69197801-69200756	0.002391876
TNS3	chr7-48388933-48391371	0.002391598
AL356599.1	chr6-146770303-146771664	0.002391592
PDE5A	chr4-119037950-119038946	0.002391474
PDE5A	chr4-119211734-119213754	0.002391265
PDGFD	chr11-105181100-105182190	0.002391258
TNS3	chr7-48034978-48037385	0.002391224
AC010275.1	chr5-111235654-111238985	0.002391214
TAFA1	chr3-69093670-69096276	0.002390826
TNS3	chr7-48108982-48110972	0.002390525
NBPF15	chr1-144560050-144561237	0.002390467

TNS3	chr7-47991314-47992694	0.002390398
TNS3	chr7-48084672-48090628	0.002390113
NBPF15	chr1-144550484-144552665	0.002389989
AL356599.1	chr6-146543038-146547054	0.002389933
TNS3	chr7-48095486-48098841	0.002389596
IKBIP	chr12-98513706-98519617	0.002389025
PGM1	chr1-64505089-64506754	0.00238869
PDE5A	chr4-119453653-119456118	0.002388662
AC010275.1	chr5-110726123-110727383	0.002388652
IKBIP	chr12-98606409-98608085	0.002388612
PDE5A	chr4-119370219-119371717	0.002388395
NBPF15	chr1-145424620-145426122	0.00238833
IKBIP	chr12-98502309-98505176	0.00238816
IKBIP	chr12-98592310-98597734	0.002387954
GAB1	chr4-143335529-143338073	0.002387949
CCDC149	chr4-25375096-25378207	0.00238791
AC010275.1	chr5-111511291-111513553	0.002387888
NBPF15	chr1-144076469-144077929	0.002387668
NBPF15	chr1-145214316-145216426	0.002387504
NBPF15	chr1-144545759-144547380	0.002386809
PGM1	chr1-64512066-64512779	0.002386761
CCDC149	chr4-25859016-25864828	0.002386323
GAB1	chr4-142354759-142357246	0.002386311
PGM1	chr1-64469619-64471680	0.002386038
GAB1	chr4-143358733-143360042	0.002385931
TNS3	chr7-47978507-47980198	0.002385695
CCDC149	chr4-25312178-25313553	0.002385445
GAB1	chr4-143183653-143187122	0.002384692
NBPF15	chr1-144411950-144413529	0.002384561
PDGFD	chr11-105099655-105101708	0.002384295
GAB1	chr4-142404226-142407894	0.002384245
NBPF15	chr1-144523740-144524543	0.002384163
AL356599.1	chr6-146857349-146858206	0.002384067
NBPF15	chr1-145232598-145233582	0.002383725
AL356599.1	chr6-146357496-146358556	0.002383644
PGM1	chr1-64744194-64746629	0.002383537
IKBIP	chr12-98490556-98491653	0.002383425
NBPF15	chr1-145280453-145282640	0.002383304
AC010275.1	chr5-111529340-111532213	0.002382797
JCHAIN	chr4-70901550-70903543	0.002382517
GAB1	chr4-142416500-142417469	0.002382282
CCDC149	chr4-25233012-25235863	0.002381218
JCHAIN	chr4-69830716-69832924	0.002381154
CCDC149	chr4-25912823-25915129	0.00238106
JCHAIN	chr4-70992387-70997527	0.002380698
JCHAIN	chr4-70891971-70892916	0.002380353
JCHAIN	chr4-69760043-69761370	0.00238018
GAB1	chr4-142961230-142962385	0.002379505
GAB1	chr4-142565505-142568490	0.00237947
PGM1	chr1-63592669-63595052	0.002378928

AL356599.1	chr6-145963204-145965500	0.00237888
GAB1	chr4-143512512-143515424	0.002378724
TXNL1	chr18-57665645-57667487	0.002378048
TNS3	chr7-47968826-47971707	0.002377693
CCDC149	chr4-25172837-25174091	0.002377665
AL356599.1	chr6-145813621-145816286	0.002377559
JCHAIN	chr4-71003927-71006977	0.002377485
AL356599.1	chr6-145863290-145867451	0.002377352
JCHAIN	chr4-70687279-70689481	0.002377231
AL356599.1	chr6-145733927-145736937	0.002376739
GAB1	chr4-142703638-142704591	0.002376711
IKBIP	chr12-98456043-98457450	0.002376373
GAB1	chr4-142845306-142847712	0.002376317
JCHAIN	chr4-71186279-71188343	0.002375879
TAFA1	chr3-69012481-69014375	0.002375707
CCDC149	chr4-26074176-26076917	0.002375612
AC010275.1	chr5-111755852-111758533	0.002375532
PDGFD	chr11-105043635-105046202	0.002375256
CCDC149	chr4-25159298-25161852	0.002375156
AL356599.1	chr6-146876186-146877205	0.002375137
JCHAIN	chr4-70838459-70841408	0.002374685
TXNL1	chr18-57649438-57650530	0.002374364
ARHGAP10	chr4-148051949-148053190	0.00237435
AL356599.1	chr6-144659035-144660138	0.002374023
ARHGAP10	chr4-148055989-148057946	0.002373414
TAFA1	chr3-66971762-66972810	0.002373308
TXNL1	chr18-57580785-57581901	0.002373281
CCDC149	chr4-24978648-24980503	0.00237209
TXNL1	chr18-57585777-57587358	0.002371962
PDGFD	chr11-104798559-104799866	0.002371606
TXNL1	chr18-56650111-56653548	0.002371518
AC103591.3	chr1-77279844-77283411	0.002371487
TXNL1	chr18-55477222-55478334	0.002371454
PDGFD	chr11-104705067-104706814	0.00237135
AL356599.1	chr6-144653877-144654874	0.002371233
JCHAIN	chr4-70703621-70706843	0.002371199
TXNL1	chr18-57629496-57631445	0.00237101
IKBIP	chr12-98437296-98440802	0.002370735
PGM1	chr1-63522519-63524227	0.0023706
PDGFD	chr11-103108849-103110825	0.002370546
PDGFD	chr11-103035867-103036565	0.002370389
PDGFD	chr11-103090883-103092871	0.002370383
TXNL1	chr18-57621014-57623153	0.002370285
ARHGAP10	chr4-147831683-147833046	0.002370262
GAB1	chr4-143558757-143560162	0.002370074
PDGFD	chr11-104876195-104877801	0.002370031
TAFA1	chr3-66997414-67000743	0.002370012
ARHGAP10	chr4-148060318-148061480	0.002369519
TNS3	chr7-47669036-47671602	0.002369348
IKBIP	chr12-98397196-98398049	0.002369272

JCHAIN	chr4-70733991-70736341	0.002369083
AC103591.3	chr1-77218646-77220059	0.00236895
AC103591.3	chr1-77312130-77314107	0.002368528
TAFA1	chr3-68088013-68088966	0.002368261
PDGFD	chr11-105032436-105036161	0.002368138
AC010275.1	chr5-112160174-112161790	0.002368033
JCHAIN	chr4-70712871-70714431	0.002367561
TAFA1	chr3-67652870-67657527	0.002367466
PDGFD	chr11-104916108-104918987	0.002367154
CCDC149	chr4-24973693-24975842	0.002367078
TXNL1	chr18-56637327-56639632	0.002366623
ARHGAP10	chr4-148375700-148376663	0.002366303
ARHGAP10	chr4-148441432-148446477	0.002366064
PDGFD	chr11-104967422-104969684	0.00236559
TAFA1	chr3-67947774-67948779	0.002365331
TAFA1	chr3-68057164-68058427	0.002364936
DIMT1	chr5-62403012-62404840	0.002364919
PGM1	chr1-63366923-63368592	0.0023648
PGM1	chr1-62434583-62438598	0.002364686
PGM1	chr1-62783197-62785101	0.0023645
PGM1	chr1-62687218-62689056	0.002364447
TXNL1	chr18-55587123-55590503	0.002364443
AC010275.1	chr5-112417870-112420690	0.002363766
PGM1	chr1-62800206-62801529	0.002363687
TNS3	chr7-47580903-47582619	0.00236349
PGM1	chr1-63319499-63324758	0.002362983
ARHGAP10	chr4-147799796-147800623	0.002362571
DIMT1	chr5-62305096-62308639	0.002362427
TXNL1	chr18-56257136-56258797	0.00236169
DIMT1	chr5-62411687-62413573	0.002361684
CCDC149	chr4-24582451-24585754	0.002361048
TIAM2	chr6-154242959-154248515	0.002360822
TNS3	chr7-47574033-47575391	0.002360698
AC103591.3	chr1-77412060-77414280	0.002360504
TXNL1	chr18-55778545-55781183	0.002360481
TIAM2	chr6-154249334-154251463	0.002360262
TIAM2	chr6-154229515-154232740	0.002360064
TIAM2	chr6-154155978-154158008	0.002359893
TNS3	chr7-47474989-47475831	0.002359123
TIAM2	chr6-154256880-154258752	0.002358299
CCDC149	chr4-24471917-24473434	0.002356897
TIAM2	chr6-154354480-154357293	0.002356406
DIMT1	chr5-62299149-62300322	0.002356389
TIAM2	chr6-154683768-154684756	0.002355963
ARHGAP10	chr4-146945415-146946248	0.002355955
TIAM2	chr6-154675450-154676218	0.002355825
ARHGAP10	chr4-147779440-147780964	0.002355249
ARHGAP10	chr4-147616719-147618587	0.002354719
TIAM2	chr6-154732619-154735075	0.002354709
DIMT1	chr5-62578251-62579110	0.002354057

ARHGAP10	chr4-147682663-147685186	0.002352354
ITGB3BP	chr1-64505089-64506754	0.002351899
ARHGAP10	chr4-147730774-147733641	0.002351702
AC103591.3	chr1-77430672-77432282	0.002350926
TIAM2	chr6-155170995-155171991	0.002350603
DIMT1	chr5-62291542-62292482	0.002350297
ITGB3BP	chr1-64512066-64512779	0.00235
ITGB3BP	chr1-64469619-64471680	0.002349289
KYNU	chr2-142865467-142868948	0.002348045
ITGB3BP	chr1-64744194-64746629	0.002346824
KYNU	chr2-142871764-142875340	0.00234629
DIMT1	chr5-62255978-62257203	0.002346227
KYNU	chr2-142877111-142880748	0.002346081
KYNU	chr2-143034217-143034831	0.002344841
BMT2	chr7-112449260-112454443	0.002344814
TIAM2	chr6-155313520-155315847	0.00234452
DIMT1	chr5-62223348-62224585	0.002343649
BMT2	chr7-111986960-111988186	0.002343631
AC103591.3	chr1-77681420-77684246	0.002343377
BMT2	chr7-112390703-112392641	0.002342906
BMT2	chr7-112461935-112463091	0.002342836
ITGB3BP	chr1-63592669-63595052	0.002342288
KYNU	chr2-143068184-143069194	0.002341084
DIMT1	chr5-61407728-61409863	0.002340757
TIAM2	chr6-155417393-155419379	0.002339539
6-Mar	chr5-10731730-10733636	0.00233937
KYNU	chr2-143151986-143154129	0.002339131
KYNU	chr2-143255628-143259946	0.002339086
PRKN	chr6-162184157-162185062	0.00233896
AC103591.3	chr1-77758745-77760343	0.002338878
BMT2	chr7-112203993-112207663	0.00233885
6-Mar	chr5-10635159-10636192	0.002338765
BMT2	chr7-112789485-112791108	0.002338356
BMT2	chr7-112080242-112082630	0.002338091
PRKN	chr6-162726759-162728852	0.002337647
DIMT1	chr5-61329628-61337466	0.002336779
BMT2	chr7-112083967-112085223	0.002336355
KYNU	chr2-143149867-143150928	0.002336176
KYNU	chr2-143261429-143263816	0.002336093
KYNU	chr2-143110569-143111433	0.002336029
6-Mar	chr5-10759791-10763385	0.002335742
AC103591.3	chr1-77779018-77780406	0.002335488
AC026341.1	chr3-113947226-113949023	0.002334604
PRKN	chr6-162180994-162182346	0.002334506
BMT2	chr7-112937706-112941087	0.002334352
ITGB3BP	chr1-63522519-63524227	0.002334088
CCDC141	chr2-178522493-178524142	0.002333175
6-Mar	chr5-10630659-10634558	0.002333158
CCDC141	chr2-179049145-179050366	0.002333059
DIMT1	chr5-61319061-61321362	0.002333033

KYNU	chr2-143139785-143142979	0.00233297
KYNU	chr2-143128262-143129984	0.002332646
CCDC141	chr2-178477783-178481709	0.002332507
BMT2	chr7-113116984-113119291	0.002332476
CCDC141	chr2-179234680-179236086	0.002332453
CCDC141	chr2-178530097-178531778	0.002332368
CCDC141	chr2-179031522-179032721	0.002332313
KANK1	chr9-621077-622055	0.00233208
DIMT1	chr5-61275209-61277922	0.002331616
BMT2	chr7-114051710-114052584	0.002331512
PRKN	chr6-163353974-163356893	0.002331444
AC103591.3	chr1-77975075-77980661	0.002330865
BMT2	chr7-114084416-114087608	0.002328686
6-Mar	chr5-10352165-10355808	0.002328576
CCDC141	chr2-179263137-179265793	0.002328434
AC026341.1	chr3-114055718-114057746	0.002328431
ITGB3BP	chr1-63366923-63368592	0.002328379
ITGB3BP	chr1-62434583-62438598	0.002328268
CCDC141	chr2-178449071-178452354	0.002328241
KANK1	chr9-503667-505929	0.002328227
ITGB3BP	chr1-62783197-62785101	0.002328085
ITGB3BP	chr1-62687218-62689056	0.002328033
6-Mar	chr5-10440730-10443010	0.002327545
PRKN	chr6-162099514-162100492	0.002327303
ITGB3BP	chr1-62800206-62801529	0.002327285
6-Mar	chr5-10332959-10334127	0.002326651
ITGB3BP	chr1-63319499-63324758	0.002326591
HPF1	chr4-169659333-169660983	0.00232637
6-Mar	chr5-10626186-10628342	0.002325536
AC026341.1	chr3-114290592-114292019	0.00232507
AC103591.3	chr1-78003227-78006330	0.002324656
AC026341.1	chr3-114231318-114233425	0.00232463
PRKN	chr6-163404496-163407170	0.002324421
HPF1	chr4-169756793-169758626	0.002324158
6-Mar	chr5-10248823-10251798	0.00232388
6-Mar	chr5-10536537-10538161	0.002323767
HPF1	chr4-169618226-169622990	0.002323188
PRKN	chr6-163709645-163713440	0.002322533
PRKN	chr6-163667152-163668747	0.00232249
AC026341.1	chr3-114152352-114153233	0.002322383
AC026341.1	chr3-114307211-114309487	0.00232222
AC026341.1	chr3-114221139-114223781	0.002321815
CCDC141	chr2-180006177-180007993	0.002321507
CCDC141	chr2-178413055-178415319	0.00232081
PRKN	chr6-163457570-163461020	0.002320724
KANK1	chr9-468210-471518	0.002320647
PRKN	chr6-163412028-163417952	0.002320532
6-Mar	chr5-10606949-10608469	0.002320388
6-Mar	chr5-10562162-10566601	0.002320193
AC026341.1	chr3-114212642-114216815	0.002320087

PRKN	chr6-163750452-163751707	0.002319692
AC103591.3	chr1-78619344-78621165	0.002319081
HPF1	chr4-170025503-170027967	0.002318857
KANK1	chr9-112994-115191	0.002318246
AC026341.1	chr3-115099905-115100777	0.002318072
AC026341.1	chr3-115783637-115785531	0.002317387
AC103591.3	chr1-78649318-78651879	0.002316895
AC026341.1	chr3-115658020-115659828	0.002315964
PRKN	chr6-163754768-163756954	0.002315943
HPF1	chr4-169611666-169613825	0.002315892
AC026341.1	chr3-115145540-115148818	0.002315638
KANK1	chr9-296625-298278	0.002313469
CCDC141	chr2-178193869-178195311	0.00231337
KANK1	chr9-178314-179531	0.002313336
KANK1	chr9-221874-223459	0.00231314
KANK1	chr9-257512-258576	0.002312486
KANK1	chr9-214034-217085	0.002312052
KANK1	chr9-211077-212928	0.002311312
KANK1	chr9-268313-279990	0.002310538
KANK1	chr9-281336-282989	0.002310022
CCDC141	chr2-178111947-178113503	0.002309254
HPF1	chr4-169270050-169272695	0.002308523
UTP3	chr4-70901550-70903543	0.002307638
HPF1	chr4-169008705-169011192	0.002306636
HPF1	chr4-168880971-168882200	0.002306356
UTP3	chr4-69830716-69832924	0.002306318
UTP3	chr4-70992387-70997527	0.002305878
UTP3	chr4-70891971-70892916	0.002305543
UTP3	chr4-69760043-69761370	0.002305375
HPF1	chr4-169079108-169080859	0.002304995
HPF1	chr4-169210477-169211744	0.002304733
HPF1	chr4-168877592-168879773	0.002303207
UTP3	chr4-71003927-71006977	0.002302766
UTP3	chr4-70687279-70689481	0.002302521
UTP3	chr4-71186279-71188343	0.002301211
UTP3	chr4-70838459-70841408	0.002300054
HPF1	chr4-168831068-168833338	0.00229925
WDR60	chr7-157851265-157852293	0.002298222
UTP3	chr4-70703621-70706843	0.002296677
WDR60	chr7-157853520-157855176	0.002295448
UTP3	chr4-70733991-70736341	0.002294627
TOX	chr8-58559671-58564341	0.002294007
TOX	chr8-58594045-58596764	0.002293907
UTP3	chr4-70712871-70714431	0.002293154
TOX	chr8-58552380-58556761	0.002292619
TOX	chr8-58410681-58412564	0.002291568
TOX	chr8-58395158-58398370	0.002290375
TOX	chr8-58657649-58660906	0.002290048
1-Mar	chr4-163481904-163483422	0.002289171
1-Mar	chr4-164956615-164957411	0.00228917

WDR60		chr7-158587520-158588406	0.002289055
USP53		chr4-118277292-118282221	0.00228831
WDR60		chr7-158975774-158977667	0.002287112
	1-Mar	chr4-163665230-163667018	0.002287088
	1-Mar	chr4-165111857-165113663	0.002287003
TOX		chr8-58172405-58173645	0.002286992
WDR60		chr7-158855709-158857415	0.002286694
WDR60		chr7-158814264-158832092	0.002286653
	1-Mar	chr4-163493236-163496428	0.002286571
WDR60		chr7-158862882-158864757	0.002286184
USP53		chr4-118589611-118592588	0.002285847
WDR60		chr7-158807037-158813601	0.002284199
	1-Mar	chr4-163590573-163592614	0.002283671
	1-Mar	chr4-163549862-163550921	0.002283491
TOX		chr8-58800767-58802581	0.002282495
	1-Mar	chr4-163558216-163559796	0.002282076
WDR60		chr7-158695645-158696924	0.002281481
	1-Mar	chr4-165206556-165209331	0.002280657
TOX		chr8-58154947-58156225	0.002280272
TRAT1		chr3-108123890-108129274	0.002280043
WDR60		chr7-158802748-158806351	0.002279731
TRAT1		chr3-108099102-108103284	0.00227944
USP53		chr4-118684758-118686097	0.002279375
TRAT1		chr3-108130165-108134966	0.00227689
WDR60		chr7-158703745-158708062	0.002276422
WDR60		chr7-158709791-158713028	0.002276102
TOX		chr8-58881615-58882458	0.002274347
TRAT1		chr3-108087127-108094053	0.00227394
	1-Mar	chr4-165255183-165256782	0.002273215
TRAT1		chr3-108219331-108225119	0.002273085
IRF4		chr6-692193-693916	0.002272827
TOX		chr8-58145389-58146510	0.002271839
USP53		chr4-120066012-120068424	0.002271676
GALNT10		chr5-154937417-154938721	0.002271647
IRF4		chr6-710460-711996	0.002271341
TRAT1		chr3-108588499-108590983	0.002270815
GALNT10		chr5-154856437-154861261	0.002270634
USP53		chr4-118834844-118837107	0.002270467
GALNT10		chr5-154940434-154941728	0.002269819
TOX		chr8-59117154-59120394	0.002269756
TRAT1		chr3-108602226-108604233	0.002269618
IRF4		chr6-498626-500143	0.002269293
	1-Mar	chr4-165378679-165379712	0.00226868
	1-Mar	chr4-165294550-165295634	0.002268329
	1-Mar	chr4-165325463-165328505	0.002267415
TRAT1		chr3-108805963-108807000	0.002267327
IRF4		chr6-1523842-1524897	0.002267013
TRAT1		chr3-108061886-108062786	0.002265857
GALNT10		chr5-154752960-154758671	0.002265508
USP53		chr4-120049601-120050328	0.002265176

IRF4	chr6-1554222-1555529	0.002263352
AC078845.1	chr7-135661435-135663457	0.002262716
TRAT1	chr3-108820606-108822984	0.002262651
USP53	chr4-118850076-118851193	0.002262491
AC078845.1	chr7-135976215-135978062	0.002262317
IRF4	chr6-473796-475627	0.002262113
AC078845.1	chr7-135146610-135149231	0.002261305
AC078845.1	chr7-135979891-135982660	0.002261253
AC078845.1	chr7-135557297-135559248	0.002260336
AC078845.1	chr7-135150113-135152534	0.002260211
AC078845.1	chr7-134995044-134996448	0.0022597
TRAT1	chr3-107927263-107928438	0.002259458
USP53	chr4-119626927-119629519	0.002258109
AHR	chr7-17769809-17771357	0.002258072
GALNT10	chr5-154734234-154735421	0.002258021
USP53	chr4-119025375-119027091	0.002257947
AHR	chr7-17938903-17941598	0.002257926
AC078845.1	chr7-134985876-134987511	0.002257854
AHR	chr7-17408217-17409012	0.00225785
MOB3B	chr9-27598951-27602554	0.002257267
TRAT1	chr3-108833328-108835750	0.00225697
AHR	chr7-17297062-17300384	0.002256882
USP53	chr4-119037950-119038946	0.002256707
AC078845.1	chr7-135160068-135162525	0.00225661
USP53	chr4-119211734-119213754	0.002256509
AC078845.1	chr7-135508764-135511220	0.002256078
MOB3B	chr9-27700962-27702233	0.002255839
IRF4	chr6-444656-448338	0.002255393
MOB3B	chr9-27590620-27591776	0.002254171
MOB3B	chr9-26891702-26893659	0.002254157
AHR	chr7-17233457-17236226	0.002254095
USP53	chr4-119453653-119456118	0.002254055
USP53	chr4-119370219-119371717	0.002253802
TRAT1	chr3-109806476-109807656	0.00225339
AC078845.1	chr7-135166233-135172138	0.002253234
AC078845.1	chr7-135206822-135212411	0.002252918
IRF4	chr6-389507-394409	0.002252813
SPATA5	chr4-122921837-122923699	0.002252332
GALNT10	chr5-154713604-154715241	0.002251806
GALNT10	chr5-154444512-154448023	0.002250596
GALNT10	chr5-154682125-154683887	0.002250119
SPATA5	chr4-122731789-122733707	0.002250093
AHR	chr7-17152466-17153991	0.002249565
AHR	chr7-16753287-16754954	0.002249559
GALNT10	chr5-154708461-154709680	0.002249425
ATG10	chr5-81006262-81008637	0.002248909
ATG10	chr5-81299310-81302815	0.002248886
SPATA5	chr4-123396297-123400296	0.002248812
MOB3B	chr9-27588007-27590019	0.002248436
AHR	chr7-16644226-16647219	0.002248144

AHR	chr7-17008992-17011076	0.002247982
GALNT10	chr5-154384384-154385721	0.002247607
MOB3B	chr9-26946292-26948152	0.002246129
ATG10	chr5-80959822-80962376	0.002245781
AHR	chr7-17142238-17143589	0.002245232
AHR	chr7-16420130-16422147	0.002245103
AHR	chr7-17041169-17041921	0.00224496
ATG10	chr5-81506771-81507404	0.002244022
AHR	chr7-17122785-17123663	0.002243461
SPATA5	chr4-122577797-122579264	0.002243306
MOB3B	chr9-27570420-27574973	0.00224301
IRF4	chr6-234959-236899	0.002242719
MMADHC	chr2-148508911-148510078	0.002242484
ATG10	chr5-80957587-80958731	0.00224214
SPATA5	chr4-123443308-123444829	0.002241722
GALNT10	chr5-154197076-154198963	0.002240721
IRF4	chr6-188578-189465	0.002240071
IRF4	chr6-224795-226326	0.002239801
MOB3B	chr9-27563992-27566510	0.002239569
IRF4	chr6-217069-220357	0.002239518
MMADHC	chr2-148524069-148525437	0.002239487
MMADHC	chr2-148545254-148546863	0.002239356
MMADHC	chr2-148538378-148540776	0.002239148
MOB3B	chr9-26953841-26957169	0.002239071
MMADHC	chr2-148526382-148529763	0.002238374
MOB3B	chr9-27527824-27530239	0.002237676
MMADHC	chr2-148552202-148554314	0.002236262
MOB3B	chr9-27460375-27461310	0.002236164
SPATA5	chr4-122150406-122153736	0.002235665
ATG10	chr5-81749028-81752447	0.002235399
MOB3B	chr9-27334844-27336467	0.002235307
SPATA5	chr4-123599377-123600712	0.002234905
MOB3B	chr9-27351385-27354202	0.002234879
CAMK4	chr5-111223076-111225350	0.002234577
CAMK4	chr5-111229050-111231582	0.002233334
CAMK4	chr5-111091658-111093327	0.002233112
GALNT10	chr5-154189074-154192425	0.002232576
RAB30	chr11-82895237-82897023	0.002232507
EPM2A	chr6-146595833-146596744	0.002231615
RAB30	chr11-82898135-82902559	0.002231102
SPATA5	chr4-123606735-123607692	0.002230972
VPS37A	chr8-17925720-17928870	0.0022308
VPS37A	chr8-17575924-17577983	0.002230748
CAMK4	chr5-111232337-111234818	0.002230746
RAB30	chr11-82888913-82891678	0.00223067
VPS37A	chr8-18068248-18070006	0.00223056
SPATA5	chr4-121950059-121952317	0.002230547
VPS37A	chr8-17697037-17698023	0.002230442
SPATA5	chr4-123616628-123619280	0.002229971
VPS37A	chr8-17921616-17924639	0.002229882

EPM2A	chr6-146770303-146771664	0.002229679
VPS37A	chr8-17907340-17908258	0.002229666
MMADHC	chr2-148613876-148615098	0.002229611
CAMK4	chr5-110738029-110739970	0.002229328
VPS37A	chr8-17245887-17248142	0.002228993
SPATA5	chr4-121869422-121871331	0.002228727
SPATA5	chr4-121822687-121824611	0.002228464
EPM2A	chr6-146543038-146547054	0.00222813
CAMK4	chr5-111235654-111238985	0.002228043
SPATA5	chr4-121800183-121801952	0.00222771
VPS37A	chr8-18082352-18085664	0.002227644
RAB30	chr11-82919774-82920885	0.002227466
ATG10	chr5-81778255-81780437	0.002226705
GALNT10	chr5-154038048-154039675	0.002226648
CAMK4	chr5-110726123-110727383	0.002225655
VPS37A	chr8-17155506-17157684	0.002225336
THEMIS	chr6-128258989-128260596	0.002225019
CCDC112	chr5-115178954-115180856	0.002224953
CAMK4	chr5-111511291-111513553	0.002224945
CAMK4	chr5-110053671-110054756	0.002224841
THEMIS	chr6-127971333-127972754	0.002224457
CCDC112	chr5-115208919-115209993	0.002224279
RAB30	chr11-82955578-82956787	0.002224004
THEMIS	chr6-128518628-128521238	0.002223601
VPS37A	chr8-18209382-18210822	0.00222267
EPM2A	chr6-146857349-146858206	0.002222662
CCDC112	chr5-115168905-115171388	0.002222495
CCSER1	chr4-89110154-89112636	0.002222336
EPM2A	chr6-146357496-146358556	0.002222266
VPS37A	chr8-16361523-16363321	0.002222244
MMADHC	chr2-148642706-148647537	0.002222146
RAB30	chr11-82959668-82961236	0.002222093
VPS37A	chr8-17026471-17028834	0.002221871
ATG10	chr5-82173934-82175010	0.002221763
ATG10	chr5-81850399-81853360	0.002221544
ATG10	chr5-82277158-82279373	0.002221219
VPS37A	chr8-16631935-16632800	0.00222078
ATG10	chr5-81970699-81973287	0.002220706
RAB30	chr11-83061843-83064419	0.002220694
THEMIS	chr6-127950577-127952028	0.002220375
CAMK4	chr5-111529340-111532213	0.0022202
UTP25	chr1-210328278-210329861	0.002220127
UTP25	chr1-210332077-210333358	0.002220116
CCSER1	chr4-89293337-89295685	0.002220103
CCSER1	chr4-89296624-89300811	0.002220072
UTP25	chr1-209827186-209828713	0.002219219
CCSER1	chr4-89282690-89286389	0.002219145
CCDC112	chr5-115261829-115263378	0.002219103
CCSER1	chr4-89288673-89291561	0.002218876
MAF	chr16-79769534-79771173	0.002218795

MAF	chr16-80540160-80541624	0.00221853
LINC01376	chr2-19868260-19869711	0.002218359
MZT2B	chr2-130824083-130825083	0.002218024
LINC01376	chr2-19891607-19892433	0.00221799
MZT2B	chr2-131039710-131042443	0.002217952
MMADHC	chr2-149160783-149161963	0.002217917
EPM2A	chr6-145963204-145965500	0.002217827
ATG10	chr5-82356743-82357918	0.002217693
RAB30	chr11-83069255-83073879	0.002217512
MMADHC	chr2-149586187-149588472	0.002217442
CCSER1	chr4-89302923-89304260	0.00221744
MMADHC	chr2-148874564-148877463	0.002217398
MAF	chr16-80590879-80591818	0.002216908
LINC01376	chr2-19354610-19364350	0.002216829
MMADHC	chr2-148926145-148927688	0.00221667
EPM2A	chr6-145813621-145816286	0.002216595
EPM2A	chr6-145863290-145867451	0.002216402
MAF	chr16-79598130-79602898	0.002216192
MAF	chr16-80676190-80677201	0.002215953
EPM2A	chr6-145733927-145736937	0.00221583
CCSER1	chr4-89829211-89830355	0.002215655
LINC01376	chr2-19346674-19353067	0.002214979
UTP25	chr1-209805461-209806831	0.002214892
CCSER1	chr4-89331320-89332385	0.002214505
EPM2A	chr6-146876186-146877205	0.002214334
LINC01376	chr2-19899865-19903501	0.002214095
MZT2B	chr2-130796229-130799208	0.002213974
THEMIS	chr6-127934159-127935188	0.002213947
MAF	chr16-78742118-78743978	0.002213861
ZFAT	chr8-134830227-134833842	0.002213648
CCSER1	chr4-89305133-89308845	0.002213595
ZFAT	chr8-134719217-134721529	0.002213592
CCSER1	chr4-89836018-89838312	0.002213591
CAMK4	chr5-111755852-111758533	0.00221343
EPM2A	chr6-144659035-144660138	0.002213298
ATG10	chr5-83076564-83078352	0.002213161
MMADHC	chr2-149597322-149598229	0.00221308
SAMD12	chr8-118089337-118090406	0.002213032
MZT2B	chr2-131092055-131094512	0.002212916
THEMIS	chr6-127346343-127348560	0.002212827
MAF	chr16-79089508-79091768	0.002212808
LINC01376	chr2-18559430-18561381	0.002212788
ZFAT	chr8-134711504-134714545	0.002212528
SAMD12	chr8-118060396-118061416	0.002212271
MAF	chr16-79093374-79094359	0.002212134
CCSER1	chr4-89324314-89325312	0.002212073
THEMIS	chr6-127457249-127458094	0.002212054
CCDC112	chr5-115634166-115635113	0.002212026
ZFAT	chr8-134684805-134686241	0.002211593
SAMD12	chr8-118099104-118100419	0.002211552

CCDC112	chr5-115295761-115297281	0.002211501
MAF	chr16-79380778-79382210	0.00221143
RAB30	chr11-83115714-83118850	0.002211373
CCSER1	chr4-89316226-89317585	0.002211328
CCDC112	chr5-115623717-115627125	0.002210923
EPM2A	chr6-144582757-144583846	0.002210857
ZFAT	chr8-135456420-135458522	0.002210846
SAMD12	chr8-118281701-118282624	0.002210825
EPM2A	chr6-144653877-144654874	0.002210695
SAMD12	chr8-118278939-118280111	0.002210656
THEMIS	chr6-127341420-127344729	0.002210599
SAMD12	chr8-118108415-118113700	0.002210413
ZFAT	chr8-133918493-133920169	0.002210273
MAF	chr16-79271826-79272826	0.002210188
CCDC112	chr5-115838729-115839495	0.002210063
CCSER1	chr4-90126843-90129614	0.002209411
MZT2B	chr2-130726706-130730025	0.002209208
THEMIS	chr6-127514416-127517077	0.002209025
THEMIS	chr6-127918230-127919310	0.002208542
SAMD12	chr8-118284808-118285903	0.002208533
CCDC112	chr5-116083973-116086229	0.002207938
SAMD12	chr8-117519237-117521934	0.002207769
CCDC112	chr5-115840693-115843837	0.002207606
THEMIS	chr6-127302482-127304076	0.002207515
MAF	chr16-79278324-79279528	0.002207301
CCDC112	chr5-115601456-115604251	0.002207244
ZFAT	chr8-133896877-133900012	0.002207131
MAF	chr16-79328520-79329828	0.002207128
THEMIS	chr6-127900629-127901712	0.00220693
LINC01376	chr2-19989246-19990769	0.002206923
MZT2B	chr2-130390304-130392307	0.00220666
THEMIS	chr6-127265863-127268721	0.002206595
TTN	chr2-178522493-178524142	0.002206558
MZT2B	chr2-130354872-130357036	0.002206522
MZT2B	chr2-130369379-130373853	0.002206513
TTN	chr2-179049145-179050366	0.002206447
CAMK4	chr5-112160174-112161790	0.002206441
UTP25	chr1-209782958-209785634	0.002206411
TTN	chr2-178477783-178481709	0.002205927
TTN	chr2-179234680-179236086	0.002205873
TTN	chr2-178530097-178531778	0.002205794
TTN	chr2-179031522-179032721	0.002205742
MAF	chr16-79286718-79288473	0.002205665
CCDC112	chr5-115484097-115485109	0.002205584
MZT2B	chr2-131103895-131107045	0.002205573
MZT2B	chr2-130341365-130343987	0.002204407
CCDC112	chr5-115542479-115545576	0.002204344
RAB30	chr11-83155421-83165001	0.002203739
AC139720.1	chr4-141635796-141638004	0.002203077
SAMD12	chr8-118620881-118622656	0.002202949

CAMK4	chr5-112417870-112420690	0.002202465
LINC01376	chr2-20445406-20451869	0.002202375
LINC01376	chr2-20438169-20440629	0.002202092
TTN	chr2-179263137-179265793	0.002202073
TTN	chr2-178449071-178452354	0.00220189
ZFAT	chr8-133645044-133646326	0.00220184
AC139720.1	chr4-143335529-143338073	0.002201467
SAMD12	chr8-117439157-117440595	0.002200762
MZT2B	chr2-130180690-130184355	0.002200339
AC139720.1	chr4-142354759-142357246	0.002199953
AC139720.1	chr4-143358733-143360042	0.002199606
LINC01376	chr2-20048749-20053265	0.002199288
LINC01376	chr2-20429299-20431363	0.002198842
AC139720.1	chr4-143183653-143187122	0.002198465
AC139720.1	chr4-142404226-142407894	0.002198047
UTP25	chr1-209626220-209628105	0.002197964
RAB30	chr11-83172346-83174621	0.00219769
RAB30	chr11-83284954-83287239	0.002196896
MZT2B	chr2-129876876-129878550	0.002196824
MZT2B	chr2-129811762-129812769	0.002196411
AC139720.1	chr4-142416500-142417469	0.002196237
UTP25	chr1-209767396-209770408	0.002196062
ZFAT	chr8-133616248-133617332	0.002196028
UTP25	chr1-209648740-209652007	0.002196023
SAMD12	chr8-119600890-119602276	0.002195992
TTN	chr2-180006177-180007993	0.002195522
RAB30	chr11-83192647-83195814	0.00219551
LINC01934	chr2-181464898-181468348	0.0021955
LINC01376	chr2-20423733-20425923	0.0021952
TTN	chr2-178413055-178415319	0.002194862
LINC01376	chr2-20349801-20352736	0.002194825
LINC01934	chr2-181891204-181893174	0.002194651
AC139720.1	chr4-142961230-142962385	0.002193683
UTP25	chr1-209656835-209658090	0.002193656
AC139720.1	chr4-142565505-142568490	0.002193647
LINC01934	chr2-181454919-181464165	0.002193401
CAAP1	chr9-27598951-27602554	0.002193155
AC139720.1	chr4-143512512-143515424	0.002192961
C9orf72	chr9-27598951-27602554	0.002192785
ZFAT	chr8-133567280-133573555	0.002192149
ZFAT	chr8-133524466-133529928	0.002192029
CAAP1	chr9-27700962-27702233	0.002191769
CEP128	chr14-81464281-81465367	0.002191597
ZFAT	chr8-133518046-133521051	0.002191564
C9orf72	chr9-27700962-27702233	0.002191399
ZFAT	chr8-133563625-133565682	0.002191287
AC139720.1	chr4-142703638-142704591	0.002191103
SAMD12	chr8-119614274-119616021	0.002190907
AC139720.1	chr4-142845306-142847712	0.002190742
CEP128	chr14-81435365-81436987	0.002190594

CAAP1	chr9-27590620-27591776	0.002190148
CAAP1	chr9-26891702-26893659	0.002190135
UTP25	chr1-209659254-209661701	0.002189983
C9orf72	chr9-27590620-27591776	0.002189778
C9orf72	chr9-26891702-26893659	0.002189764
SAMD12	chr8-119638416-119639956	0.002189629
LINC01934	chr2-181419064-181421411	0.002189616
CEP128	chr14-81519152-81520141	0.002188475
TTN	chr2-178193869-178195311	0.002187826
UTP25	chr1-209755423-209757593	0.002187767
LINC01934	chr2-181393179-181396348	0.00218629
UTP25	chr1-209672763-209676241	0.002186007
CEP128	chr14-81415849-81417350	0.002185569
AC139720.1	chr4-143558757-143560162	0.002184986
CAAP1	chr9-27588007-27590019	0.002184577
UTP25	chr1-209747318-209749010	0.002184439
LINC01934	chr2-181310975-181314708	0.002184308
C9orf72	chr9-27588007-27590019	0.002184208
TTN	chr2-178111947-178113503	0.002183934
TTN	chr2-177753608-177754664	0.002183848
CEP128	chr14-81533043-81535979	0.002183448
LINC01934	chr2-181303834-181310073	0.00218258
HS2ST1	chr1-86787654-86789390	0.002182401
CAAP1	chr9-26946292-26948152	0.002182334
C9orf72	chr9-26946292-26948152	0.002181964
HS2ST1	chr1-86773127-86775702	0.002181914
THYN1	chr11-133927529-133929050	0.002180894
THYN1	chr11-133944560-133948898	0.002180624
THYN1	chr11-133955075-133958258	0.002179423
CAAP1	chr9-27570420-27574973	0.002179303
LINC01934	chr2-181300308-181301634	0.002179214
CEP128	chr14-81400115-81402387	0.002179169
C9orf72	chr9-27570420-27574973	0.002178935
DOCK5	chr8-24271584-24272597	0.002178913
HS2ST1	chr1-86912613-86916199	0.002178693
HS2ST1	chr1-86703497-86706485	0.002178626
HS2ST1	chr1-87869348-87870311	0.002177997
DOCK5	chr8-24293685-24294383	0.002177507
MICU3	chr8-17925720-17928870	0.002177266
MICU3	chr8-17575924-17577983	0.002177216
MICU3	chr8-18068248-18070006	0.002177029
MICU3	chr8-17697037-17698023	0.002176917
MICU3	chr8-17921616-17924639	0.00217637
MICU3	chr8-17907340-17908258	0.002176161
CAAP1	chr9-27563992-27566510	0.002175961
THYN1	chr11-133967040-133968111	0.002175906
C9orf72	chr9-27563992-27566510	0.002175593
HS2ST1	chr1-86502043-86504288	0.00217557
MICU3	chr8-17245887-17248142	0.0021755
CAAP1	chr9-26953841-26957169	0.002175477

THYN1	chr11-134223106-134226149	0.002175134
C9orf72	chr9-26953841-26957169	0.002175107
HS2ST1	chr1-86155064-86157137	0.002175027
SETBP1	chr18-45668770-45671769	0.002174992
LINC01934	chr2-180006177-180007993	0.002174715
HS2ST1	chr1-86137267-86138070	0.002174631
HS2ST1	chr1-86394697-86397520	0.002174624
CEP128	chr14-81218717-81222169	0.002174488
MICU3	chr8-18082352-18085664	0.002174184
CAAP1	chr9-27527824-27530239	0.002174121
SETBP1	chr18-45837329-45840047	0.002174062
THYN1	chr11-134068494-134070924	0.002173989
LINC01934	chr2-181164655-181166045	0.002173784
C9orf72	chr9-27527824-27530239	0.002173753
SETBP1	chr18-45686288-45689347	0.002173653
THYN1	chr11-134252489-134254373	0.002173509
HS2ST1	chr1-87423431-87424470	0.002173316
CEP128	chr14-81169562-81171070	0.002172993
DOCK5	chr8-24349155-24350936	0.002172981
CEP128	chr14-80959016-80960317	0.002172979
CEP128	chr14-81208445-81209749	0.002172813
SETBP1	chr18-45664662-45667696	0.002172801
HS2ST1	chr1-87044534-87046480	0.002172726
CAAP1	chr9-27460375-27461310	0.002172653
C9orf72	chr9-27460375-27461310	0.002172284
CEP128	chr14-80930424-80932005	0.002172191
CEP128	chr14-80954669-80956138	0.002171936
MICU3	chr8-17155506-17157684	0.00217193
CAAP1	chr9-27334844-27336467	0.00217182
SETBP1	chr18-45774133-45775932	0.002171453
C9orf72	chr9-27334844-27336467	0.002171449
CAAP1	chr9-27351385-27354202	0.002171405
SETBP1	chr18-45826651-45829497	0.002171393
THYN1	chr11-134027417-134028386	0.002171102
CEP128	chr14-80941015-80942756	0.002171047
C9orf72	chr9-27351385-27354202	0.002171034
THYN1	chr11-134275314-134277423	0.002170974
THYN1	chr11-134050235-134051232	0.002170616
THYN1	chr11-134330919-134332898	0.002169717
MICU3	chr8-18209382-18210822	0.002169328
MICU3	chr8-16361523-16363321	0.002168914
HS2ST1	chr1-87333594-87335944	0.002168878
MICU3	chr8-17026471-17028834	0.00216855
HS2ST1	chr1-87327708-87332519	0.002168503
PIP5K1B	chr9-68844041-68845463	0.002168502
LINC01934	chr2-180979019-180982127	0.002168471
DOCK5	chr8-26265659-26267654	0.002168299
LINC01934	chr2-181140057-181143030	0.002168286
DOCK5	chr8-25457202-25459813	0.002167853
THYN1	chr11-134032515-134035194	0.002167657

THYN1	chr11-134036228-134038202	0.002167653
JAKMIP2	chr5-148963383-148964321	0.002167591
MICU3	chr8-16631935-16632800	0.002167485
DOCK5	chr8-26382144-26384508	0.00216706
SETBP1	chr18-45105880-45107088	0.002167045
PIP5K1B	chr9-68974186-68976341	0.002166865
FAM114A2	chr5-154937417-154938721	0.002166402
DOCK5	chr8-26290336-26293747	0.002166299
TFB1M	chr6-154242959-154248515	0.002165963
LINC01934	chr2-181123441-181126680	0.002165938
DOCK5	chr8-24386291-24388290	0.002165909
PIP5K1B	chr9-68779060-68781168	0.002165648
TFB1M	chr6-154249334-154251463	0.002165448
FAM114A2	chr5-154856437-154861261	0.002165436
TFB1M	chr6-154229515-154232740	0.002165266
TFB1M	chr6-154155978-154158008	0.002165109
DOCK5	chr8-26302994-26305659	0.002165021
FAM114A2	chr5-154940434-154941728	0.002164659
DOCK5	chr8-25242704-25244534	0.002163984
TFB1M	chr6-154256880-154258752	0.002163647
SETBP1	chr18-44678796-44681554	0.002163581
SETBP1	chr18-44507426-44508908	0.002162904
JAKMIP2	chr5-146751515-146752494	0.002162595
JAKMIP2	chr5-148847601-148850064	0.002162382
PIP5K1B	chr9-69027387-69029311	0.002162305
TFB1M	chr6-154354480-154357293	0.002161909
TFB1M	chr6-154683768-154684756	0.002161502
TFB1M	chr6-154675450-154676218	0.002161376
SETBP1	chr18-44723299-44726333	0.002161313
SETBP1	chr18-45102861-45104407	0.002160688
FAM114A2	chr5-154752960-154758671	0.002160546
TFB1M	chr6-154732619-154735075	0.002160352
PIP5K1B	chr9-68704576-68707052	0.00215977
DOCK5	chr8-25183607-25186698	0.002159351
DOCK5	chr8-25237271-25239876	0.002158999
SETBP1	chr18-44729577-44731990	0.002158095
PIP5K1B	chr9-69035230-69037047	0.002158061
PIP5K1B	chr9-69173550-69175924	0.002157359
SETBP1	chr18-44758818-44760525	0.002157282
PIP5K1B	chr9-69671217-69673504	0.002156934
PIP5K1B	chr9-69168386-69169602	0.002156748
DOCK5	chr8-25198314-25199777	0.002156711
GNPTAB	chr12-101875254-101879050	0.002156674
TFB1M	chr6-155170995-155171991	0.002156584
PIP5K1B	chr9-69758793-69760657	0.002156473
PIP5K1B	chr9-69120684-69123032	0.00215632
GNPTAB	chr12-101917416-101918161	0.002155482
GNPTAB	chr12-101838492-101840373	0.002154903
JAKMIP2	chr5-146877191-146879204	0.002154884
PIP5K1B	chr9-68559719-68561004	0.002154253

FAM114A2	chr5-154734234-154735421	0.002153408
JAKMIP2	chr5-148831230-148833621	0.002153314
GNPTAB	chr12-102060848-102063398	0.002152816
PIP5K1B	chr9-68546232-68548293	0.002151669
TFB1M	chr6-155313520-155315847	0.002151002
LMO4	chr1-86787654-86789390	0.002150889
GNPTAB	chr12-101828476-101831647	0.002150468
LMO4	chr1-86773127-86775702	0.002150409
GNPTAB	chr12-102119146-102121327	0.002150121
RRAGA	chr9-19048195-19051504	0.002149019
JAKMIP2	chr5-147481383-147483181	0.00214753
FAM114A2	chr5-154713604-154715241	0.00214748
GNPTAB	chr12-101669821-101672401	0.002147309
LMO4	chr1-86912613-86916199	0.002147235
CEP78	chr9-77176153-77179502	0.002147224
LMO4	chr1-86703497-86706485	0.002147168
LMO4	chr1-87869348-87870311	0.002146548
TFB1M	chr6-155417393-155419379	0.002146432
FAM114A2	chr5-154444512-154448023	0.002146327
GNPTAB	chr12-101696556-101699170	0.002146278
FAM114A2	chr5-154682125-154683887	0.002145871
GNPTAB	chr12-101615562-101616807	0.002145757
GNPTAB	chr12-101822904-101824570	0.002145672
TFB1M	chr6-156395024-156397760	0.002145455
RRAGA	chr9-19102032-19103833	0.002145367
FAM114A2	chr5-154708461-154709680	0.002145209
RRAGA	chr9-19925872-19926618	0.002144523
LMO4	chr1-86502043-86504288	0.002144156
JAKMIP2	chr5-148825534-148830365	0.002144105
GABPB1-AS1	chr15-50064248-50067748	0.002144084
GNPTAB	chr12-101743533-101744653	0.002144008
LMO4	chr1-86155064-86157137	0.002143622
FAM114A2	chr5-154384384-154385721	0.002143476
RRAGA	chr9-19464466-19465433	0.002143352
GNPTAB	chr12-101773493-101774282	0.002143244
LMO4	chr1-86137267-86138070	0.002143232
LMO4	chr1-86394697-86397520	0.002143224
GABPB1-AS1	chr15-49619931-49622022	0.002143157
JAKMIP2	chr5-147782120-147783148	0.002143123
GNPTAB	chr12-101406922-101408830	0.00214288
RRAGA	chr9-19125600-19129245	0.002142304
CEP78	chr9-77909008-77910487	0.002142068
LMO4	chr1-87423431-87424470	0.002141937
JAKMIP2	chr5-147804642-147805797	0.002141468
LMO4	chr1-87044534-87046480	0.002141353
GNPTAB	chr12-101279278-101280859	0.002141344
RRAGA	chr9-20209265-20210451	0.002141216
GABPB1-AS1	chr15-50107463-50108572	0.002140867
JAKMIP2	chr5-148186090-148186994	0.002140501
GABPB1-AS1	chr15-49169510-49171331	0.002140239

GABPB1-AS1	chr15-49154763-49156615	0.002138959
RRAGA	chr9-19153791-19154726	0.002138863
JAKMIP2	chr5-148382979-148384989	0.002138772
JAKMIP2	chr5-148807502-148812591	0.002138368
RRAGA	chr9-19407663-19410033	0.002138185
GLCCI1	chr7-8174855-8178514	0.002137577
LMO4	chr1-87333594-87335944	0.002137563
LMO4	chr1-87327708-87332519	0.002137192
JAKMIP2	chr5-148805370-148806970	0.002137192
CEP78	chr9-77934856-77936631	0.002136923
FAM114A2	chr5-154197076-154198963	0.00213691
CEP78	chr9-78021626-78023715	0.002136514
CEP78	chr9-78296085-78298445	0.002136497
RRAGA	chr9-20242172-20244870	0.002136179
EPB41L2	chr6-131061437-131064005	0.002135687
CEP78	chr9-78029491-78033626	0.002135562
CEP78	chr9-77977034-77978665	0.002135373
CEP78	chr9-78235299-78237036	0.002135169
EPB41L2	chr6-131134946-131136824	0.002134905
CEP78	chr9-77943819-77947505	0.002134669
GABPB1-AS1	chr15-50112271-50114883	0.002134654
RRAGA	chr9-19161160-19164014	0.002134546
GLCCI1	chr7-8131575-8134870	0.002133623
EPB41L2	chr6-130575585-130577826	0.002132894
CEP78	chr9-78073724-78076019	0.002132346
RRAGA	chr9-19377248-19381267	0.002132334
CEP78	chr9-78200693-78201881	0.002131586
EPB41L2	chr6-131199399-131200763	0.002131061
RRAGA	chr9-19183778-19185045	0.002130572
RRAGA	chr9-19229689-19233160	0.002129348
CEP78	chr9-78103562-78104640	0.002129232
FAM114A2	chr5-154189074-154192425	0.002129143
GABPB1-AS1	chr15-50685589-50687695	0.002129047
GABPB1-AS1	chr15-50247560-50248736	0.002128884
GABPB1-AS1	chr15-50764162-50766613	0.002128865
CEP78	chr9-78118722-78119683	0.002128822
EPB41L2	chr6-130513331-130514533	0.00212864
GABPB1-AS1	chr15-50423239-50425495	0.002127342
GLCCI1	chr7-8124912-8127707	0.002126947
EPB41L2	chr6-131281109-131282328	0.002126699
GABPB1-AS1	chr15-50885125-50886349	0.002126585
GABPB1-AS1	chr15-50350024-50357258	0.002126436
EPB41L2	chr6-130183280-130184554	0.002125827
GABPB1-AS1	chr15-51076411-51078031	0.002125652
EPB41L2	chr6-130222692-130224222	0.002125651
EPB41L2	chr6-130131260-130133423	0.002125311
EPB41L2	chr6-130215090-130216075	0.002125141
CPLANE1	chr5-36685256-36686413	0.002124626
GABPB1-AS1	chr15-50906658-50909829	0.002124591
CPLANE1	chr5-36657318-36658409	0.002124406

EPB41L2	chr6-131627431-131629202	0.002124061
FAM114A2	chr5-154038048-154039675	0.002123491
CPLANE1	chr5-36239654-36243314	0.002122811
CPLANE1	chr5-36689042-36690938	0.002122414
EPB41L2	chr6-130027771-130029957	0.002122356
CPLANE1	chr5-36150714-36153072	0.002121748
CPLANE1	chr5-37111425-37112703	0.002121257
GLCCI1	chr7-7967547-7972585	0.002119976
CPLANE1	chr5-36874624-36879190	0.00211982
CPLANE1	chr5-37197275-37198538	0.002119165
RGS18	chr1-193104036-193105968	0.002119006
RGS18	chr1-193120863-193123527	0.002118982
CPLANE1	chr5-36722854-36725714	0.002118936
EPB41L2	chr6-130017900-130021663	0.002118025
RGS18	chr1-193057919-193061434	0.002117866
RGS18	chr1-192936119-192938035	0.002117782
RGS18	chr1-192940286-192942648	0.00211767
CPLANE1	chr5-36870321-36870942	0.002117329
RGS18	chr1-192954622-192955579	0.002117259
GLCCI1	chr7-7639330-7642328	0.002117071
GLCCI1	chr7-7566045-7570232	0.002116909
CPLANE1	chr5-36744340-36746034	0.0021167
GLCCI1	chr7-7944269-7945482	0.002115843
RGS18	chr1-192907571-192908617	0.002115765
GLCCI1	chr7-7761040-7762917	0.002115535
FBXL3	chr13-78022038-78022823	0.002114702
CPLANE1	chr5-37248435-37250143	0.002113674
GLCCI1	chr7-7257163-7260626	0.002113081
RGS18	chr1-192805355-192815423	0.002111259
FBXL3	chr13-77695870-77699587	0.00210928
HS3ST3B1	chr17-14427746-14429499	0.002108142
CPLANE1	chr5-37370124-37372191	0.002107502
GLCCI1	chr7-7251183-7252409	0.002106874
HS3ST3B1	chr17-14604514-14605818	0.002106307
RGS18	chr1-192608632-192610466	0.00210614
RGS18	chr1-192516405-192517665	0.002106035
HS3ST3B1	chr17-14404178-14405085	0.002105734
RGS18	chr1-192538606-192540116	0.002105061
FBXL3	chr13-77477962-77479031	0.002105016
RGS18	chr1-192157749-192159265	0.002104413
CPLANE1	chr5-37378619-37379862	0.002103818
RGS18	chr1-192544972-192546933	0.002103151
RGS18	chr1-192575157-192576265	0.002103041
GLCCI1	chr7-6990867-6991798	0.002102787
HS3ST3B1	chr17-14737836-14738999	0.002102006
GLCCI1	chr7-7180949-7184421	0.002101801
FBXL3	chr13-77338449-77339831	0.002100661
GLCCI1	chr7-7156664-7160242	0.002100522
HS3ST3B1	chr17-14358956-14360309	0.002099913
AC073332.1	chr7-17769809-17771357	0.002098667

AC073332.1	chr7-17938903-17941598	0.002098532
AC073332.1	chr7-17408217-17409012	0.002098461
FBXL3	chr13-76883594-76887384	0.002098229
HS3ST3B1	chr17-15237883-15239062	0.002097854
FBXL3	chr13-76879112-76880377	0.002097671
AC073332.1	chr7-17297062-17300384	0.002097562
ANXA1	chr9-72585527-72587408	0.00209713
AC073332.1	chr7-18177679-18178788	0.002096982
ANXA1	chr9-72925310-72926323	0.00209647
ANXA1	chr9-72292088-72293504	0.002095964
FBXL3	chr13-76979831-76980576	0.002095886
FBXL3	chr13-77328508-77330673	0.002095779
ANXA1	chr9-72294240-72295375	0.002095359
FBXL3	chr13-76799439-76800163	0.00209526
AC073332.1	chr7-17233457-17236226	0.002094971
ANXA1	chr9-72304222-72307260	0.002094554
ANXA1	chr9-72526814-72528257	0.002094355
FBXL3	chr13-76744909-76746300	0.002093766
HS3ST3B1	chr17-14351181-14353562	0.002093723
HS3ST3B1	chr17-14199589-14202820	0.002093591
HS3ST3B1	chr17-14196303-14198267	0.002093213
GABPB1	chr15-50064248-50067748	0.002092513
ANXA1	chr9-72351466-72357300	0.00209245
FBXL3	chr13-76990671-76993536	0.002092321
HS3ST3B1	chr17-14203328-14204891	0.00209225
ANXA1	chr9-73131707-73134282	0.002092211
NFIA	chr1-61049623-61051554	0.002091859
HS3ST3B1	chr17-14068786-14070159	0.002091841
FBXL3	chr13-77323125-77327735	0.002091702
GABPB1	chr15-49619931-49622022	0.002091607
HS3ST3B1	chr17-13930746-13932438	0.002090856
AC073332.1	chr7-17152466-17153991	0.00209076
AC073332.1	chr7-16753287-16754954	0.002090754
ANXA1	chr9-72464648-72465411	0.002090625
FBXL3	chr13-77025051-77028380	0.002090372
NFIA	chr1-61056611-61058995	0.002090314
HS3ST3B1	chr17-14296685-14305495	0.002090305
HS3ST3B1	chr17-14308681-14310444	0.002090252
ANXA1	chr9-72358377-72366566	0.002089838
NFIA	chr1-61042680-61043822	0.002089727
AC073332.1	chr7-16644226-16647219	0.00208944
GABPB1	chr15-50107463-50108572	0.002089373
AC073332.1	chr7-17008992-17011076	0.002089288
ANXA1	chr9-72443173-72444604	0.002088774
GABPB1	chr15-49169510-49171331	0.002088759
GABPB1	chr15-49154763-49156615	0.00208751
AC073332.1	chr7-17142238-17143589	0.002086731
AC073332.1	chr7-16420130-16422147	0.002086612
ANXA1	chr9-73147531-73149953	0.002086537
AC073332.1	chr7-17041169-17041921	0.002086478

NFIA	chr1-61076371-61080358	0.002085995
NFIA	chr1-59813997-59815790	0.002085621
AC073332.1	chr7-17122785-17123663	0.002085085
NFIA	chr1-59672881-59675234	0.002084663
GABPB1	chr15-50112271-50114883	0.002083309
NFIA	chr1-59696466-59697814	0.002082581
NFIA	chr1-59692434-59693934	0.002082527
ANXA1	chr9-73151141-73177008	0.002082095
NFIA	chr1-61080931-61084125	0.002081381
AL513188.1	chr6-20168536-20170291	0.00208128
ABCB1	chr7-86978451-86979563	0.002080178
ABCB1	chr7-87474016-87476363	0.002079883
ABCB1	chr7-87344669-87346331	0.00207978
ANXA1	chr9-73219827-73220842	0.002079562
ABCB1	chr7-87058726-87060328	0.00207863
NFIA	chr1-61253317-61255714	0.002078271
GABPB1	chr15-50685589-50687695	0.002077837
GABPB1	chr15-50247560-50248736	0.002077677
GABPB1	chr15-50764162-50766613	0.00207766
ABCB1	chr7-87215027-87221430	0.002077575
ABCB1	chr7-87598461-87601312	0.002077324
NFIA	chr1-61452971-61454010	0.002076423
GABPB1	chr15-50423239-50425495	0.002076171
ABCB1	chr7-87151567-87154523	0.00207615
ABCB1	chr7-87211574-87214329	0.002075671
GABPB1	chr15-50885125-50886349	0.002075434
GABPB1	chr15-50350024-50357258	0.002075286
GABPB1	chr15-51076411-51078031	0.002074522
NFIA	chr1-61724280-61725916	0.002074079
ABCB1	chr7-87627408-87629480	0.002073637
GABPB1	chr15-50906658-50909829	0.002073488
PCSK5	chr9-76650675-76651967	0.002073144
AL513188.1	chr6-20173562-20174766	0.002071205
ABCB1	chr7-87873627-87877838	0.002070703
FAM169A	chr5-75057392-75060810	0.002070471
NFIA	chr1-61741661-61743582	0.002070053
H2AFZ	chr4-99945777-99947834	0.002070012
PCSK5	chr9-76595404-76597072	0.002069992
H2AFZ	chr4-101084307-101085954	0.002069361
FAM169A	chr5-75235627-75237658	0.002069345
AL513188.1	chr6-21986977-21988148	0.002068931
H2AFZ	chr4-99948680-99951591	0.002068856
ABCB1	chr7-87933446-87935436	0.002068725
FAM169A	chr5-75049736-75054761	0.002068657
AL513188.1	chr6-20365363-20367573	0.002068483
H2AFZ	chr4-99893488-99895393	0.002068266
H2AFZ	chr4-101018723-101021536	0.00206807
AL513188.1	chr6-21855530-21857569	0.002068002
CROT	chr7-86978451-86979563	0.002067828
H2AFZ	chr4-100093313-100094090	0.002067633

CROT	chr7-87474016-87476363	0.002067535
CROT	chr7-87344669-87346331	0.002067434
AL513188.1	chr6-20399555-20405156	0.002066954
AL513188.1	chr6-20325803-20326719	0.002066774
FAM169A	chr5-74638149-74643924	0.002066316
CROT	chr7-87058726-87060328	0.002066289
FAM169A	chr5-74865313-74867266	0.002066221
ANKAR	chr2-190647285-190650812	0.002066124
ABCB1	chr7-88217630-88221368	0.002066003
AL513188.1	chr6-21593026-21597372	0.002065999
FAM169A	chr5-74766071-74768029	0.002065359
CROT	chr7-87215027-87221430	0.002065241
AK5	chr1-77279844-77283411	0.002065183
FAM169A	chr5-75318912-75321435	0.00206505
CROT	chr7-87598461-87601312	0.002064992
NFIA	chr1-61781878-61783879	0.002064927
EHBP1	chr2-61853132-61855616	0.002064181
AL513188.1	chr6-20528094-20529542	0.002064081
WRN	chr8-30141162-30142596	0.002064043
FAM169A	chr5-74630199-74632503	0.002063972
PCSK5	chr9-76575869-76578256	0.002063962
CROT	chr7-87151567-87154523	0.002063823
WRN	chr8-30154569-30158928	0.002063669
AL513188.1	chr6-21586967-21589826	0.002063548
CROT	chr7-87211574-87214329	0.002063348
AL513188.1	chr6-20205635-20207477	0.002063274
EHBP1	chr2-61793189-61794062	0.002063179
AK5	chr1-77218646-77220059	0.002062974
AL513188.1	chr6-20318823-20321114	0.002062962
ANKAR	chr2-189657543-189659405	0.002062744
AK5	chr1-77312130-77314107	0.002062607
AL513188.1	chr6-20533842-20535368	0.002062557
H2AFZ	chr4-99855086-99857790	0.00206222
WRN	chr8-30131799-30137748	0.002062022
ANKAR	chr2-189660771-189663128	0.002061952
ANKAR	chr2-190532260-190536735	0.002061479
CROT	chr7-87627408-87629480	0.002061328
EHBP1	chr2-61887324-61889945	0.002061312
ANKAR	chr2-189579539-189582222	0.002060918
AL513188.1	chr6-20211031-20213807	0.002060652
ABCB1	chr7-88278721-88279568	0.002060622
ANKAR	chr2-190406667-190410613	0.00206014
ANKAR	chr2-190359294-190360663	0.002059971
FAM169A	chr5-75335981-75338707	0.002059873
EHBP1	chr2-61469591-61472952	0.002059826
ANKAR	chr2-190525874-190527148	0.002059794
WRN	chr8-30655667-30659145	0.002059518
FAM169A	chr5-75716653-75718124	0.002059456
EHBP1	chr2-61762244-61765248	0.002059403
WRN	chr8-30111754-30116951	0.002059223

FAM169A	chr5-74537784-74539004	0.002059206
SLC17A5	chr6-73388572-73389671	0.002059167
SLC17A5	chr6-73309390-73310769	0.002059109
ANKAR	chr2-189440135-189442286	0.002058972
AK5	chr1-76294343-76295489	0.002058651
MZT2A	chr2-130824083-130825083	0.002058512
MZT2A	chr2-131039710-131042443	0.002058445
CROT	chr7-87873627-87877838	0.002058412
ANKAR	chr2-189673530-189675809	0.002058388
PCSK5	chr9-76570841-76572130	0.002058316
ANKAR	chr2-190342825-190345000	0.002057792
FAM169A	chr5-75566029-75568071	0.002057154
AK5	chr1-76153899-76155279	0.0020571
SLC17A5	chr6-73262582-73263834	0.002057069
SLC17A5	chr6-73652201-73655699	0.002056975
FAM169A	chr5-75509530-75512676	0.002056832
CROT	chr7-87933446-87935436	0.002056446
SLC17A5	chr6-73585115-73586913	0.002056374
SLC17A5	chr6-73695225-73697492	0.002056359
EHBP1	chr2-61905169-61906433	0.002056209
EHBP1	chr2-61693741-61695585	0.002056089
EHBP1	chr2-61535907-61539728	0.002056081
WRN	chr8-30103000-30106053	0.002055996
SLC17A5	chr6-73421830-73423206	0.002055979
SMIM8	chr6-87151433-87152967	0.002055968
AK5	chr1-76267444-76268498	0.002055822
SLC17A5	chr6-73746832-73748116	0.002055802
AK5	chr1-76270199-76271253	0.002055789
AK5	chr1-77412060-77414280	0.00205562
SLC17A5	chr6-72619706-72623632	0.002055538
CEBPD	chr8-47360248-47362379	0.002055462
CEBPD	chr8-47353141-47354274	0.002055261
SMIM8	chr6-87154441-87157344	0.002055048
PCSK5	chr9-76458695-76460552	0.00205502
MZT2A	chr2-130796229-130799208	0.002054753
ANKAR	chr2-190318869-190320336	0.002054573
FAM169A	chr5-74433314-74435010	0.002054449
ANKAR	chr2-189762107-189764013	0.002054432
KCNQ5	chr6-73388572-73389671	0.002054206
KCNQ5	chr6-73309390-73310769	0.00205415
TSHZ1	chr18-74994421-74996449	0.002054118
CEBPD	chr8-47627362-47628371	0.002053978
MZT2A	chr2-131092055-131094512	0.002053772
CROT	chr7-88217630-88221368	0.002053739
SLC17A5	chr6-73579389-73581336	0.002053561
FXN	chr9-68844041-68845463	0.002053426
PCSK5	chr9-76403183-76403961	0.002053417
H2AFZ	chr4-99845356-99848834	0.002053266
CEBPD	chr8-47733676-47739294	0.002053082
ANKAR	chr2-189782029-189785322	0.002052873

ABCB1	chr7-88315479-88317904	0.002052577
WRN	chr8-30721888-30724685	0.002052305
KCNQ5	chr6-73262582-73263834	0.002052115
KCNQ5	chr6-73652201-73655699	0.002052022
TSHZ1	chr18-75070386-75072017	0.002051987
TSHZ1	chr18-74631240-74632676	0.002051919
FXN	chr9-68974186-68976341	0.002051875
CEBPD	chr8-47368872-47370475	0.002051798
EHBP1	chr2-62194564-62201250	0.002051645
WRN	chr8-30067057-30102417	0.002051577
CEBPD	chr8-47600195-47603155	0.002051467
KCNQ5	chr6-73585115-73586913	0.002051423
KCNQ5	chr6-73695225-73697492	0.002051408
PCSK5	chr9-76393349-76395940	0.002051257
CEBPD	chr8-47259459-47261808	0.002051128
KCNQ5	chr6-73421830-73423206	0.002051026
SMIM8	chr6-87321921-87323222	0.002051013
RAP2A	chr13-98562881-98564806	0.002050924
SLC17A5	chr6-73449295-73453461	0.002050869
KCNQ5	chr6-73746832-73748116	0.002050853
FXN	chr9-68779060-68781168	0.002050724
KCNQ5	chr6-72619706-72623632	0.002050586
MZT2A	chr2-130726706-130730025	0.00205033
CEBPD	chr8-47959222-47962181	0.002049761
RAP2A	chr13-98541817-98544452	0.002049622
RAP2A	chr13-97109579-97110499	0.002049563
SLC17A5	chr6-73509779-73524658	0.002049386
RAP2A	chr13-98565735-98572999	0.002049234
EHBP1	chr2-62302524-62307369	0.002049182
KCNQ5	chr6-73579389-73581336	0.002048617
CROT	chr7-88278721-88279568	0.00204839
EHBP1	chr2-62704622-62707322	0.002048081
MZT2A	chr2-130390304-130392307	0.002047964
MZT2A	chr2-130354872-130357036	0.002047835
MZT2A	chr2-130369379-130373853	0.002047827
FXN	chr9-69027387-69029311	0.002047557
CEBPD	chr8-47597219-47599190	0.00204746
TSHZ1	chr18-75206914-75212917	0.002047307
AK5	chr1-77430672-77432282	0.00204728
CEBPD	chr8-47525761-47526945	0.002047261
RAP2A	chr13-98508823-98510507	0.002047196
PCSK5	chr9-76023426-76024759	0.002047013
MZT2A	chr2-131103895-131107045	0.002046956
SLC17A5	chr6-73454515-73456698	0.002046883
CEBPD	chr8-48007103-48010848	0.002046864
EHBP1	chr2-63587407-63589749	0.002046733
SLC17A5	chr6-73460713-73463264	0.002046415
CEBPD	chr8-48513963-48515336	0.002046358
TSHZ1	chr18-74596458-74599204	0.002046158
KCNQ5	chr6-73449295-73453461	0.002045928

MZT2A	chr2-130341365-130343987	0.002045872
CEBPD	chr8-46917415-46918501	0.002045773
WRN	chr8-30056178-30062519	0.002045566
CEBPD	chr8-47573336-47574954	0.0020454
RAP2A	chr13-98484362-98485630	0.002045242
FXN	chr9-68704576-68707052	0.002045157
WRN	chr8-30725479-30728586	0.002045019
H2AFZ	chr4-99815343-99818760	0.002044751
SMIM8	chr6-87407412-87409064	0.002044654
EHBP1	chr2-63839942-63843121	0.002044586
WRN	chr8-31032169-31034808	0.002044477
KCNQ5	chr6-73509779-73524658	0.002044448
RAP2A	chr13-98574155-98579227	0.002044388
MZT2A	chr2-131681775-131683342	0.002044356
RAP2A	chr13-97209940-97211381	0.002044353
FXN	chr9-69945317-69947357	0.002043806
RAP2A	chr13-98475450-98477262	0.002043734
FXN	chr9-69035230-69037047	0.002043538
H2AFZ	chr4-98927414-98930883	0.00204329
EHBP1	chr2-63855408-63856970	0.002043149
TSHZ1	chr18-75215753-75217347	0.002043081
FXN	chr9-69173550-69175924	0.002042871
FXN	chr9-69671217-69673504	0.00204247
MZT2A	chr2-131672418-131674383	0.002042424
FXN	chr9-69168386-69169602	0.002042293
FXN	chr9-69758793-69760657	0.002042034
KCNQ5	chr6-73454515-73456698	0.002041951
FXN	chr9-69120684-69123032	0.002041888
MZT2A	chr2-131490924-131494183	0.002041812
RAP2A	chr13-98444554-98447234	0.002041638
KCNQ5	chr6-73460713-73463264	0.002041485
PCSK5	chr9-75889549-75893547	0.00204144
WRN	chr8-30811779-30813375	0.002041283
TSHZ1	chr18-75245813-75250784	0.002041237
H2AFZ	chr4-98994596-98997059	0.002041088
WRN	chr8-30743299-30744993	0.002040916
AK5	chr1-77681420-77684246	0.002040708
MZT2A	chr2-131527388-131529821	0.002040589
PCSK5	chr9-74951299-74953912	0.002040506
CROT	chr7-88315479-88317904	0.002040391
FXN	chr9-68559719-68561004	0.002039933
MSRA	chr8-9002042-9003735	0.002039899
H2AFZ	chr4-99562731-99564592	0.002039506
MSRA	chr8-11199037-11202359	0.002039373
TSHZ1	chr18-74588632-74591402	0.002039219
H2AFZ	chr4-99043148-99044170	0.002039213
WRN	chr8-30050040-30051959	0.002039195
PCSK5	chr9-75026830-75029470	0.002038752
RAP2A	chr13-97221586-97227665	0.002038716
MSRA	chr8-9065619-9066416	0.002038712

RAP2A	chr13-97975216-97977695	0.002038545
SMIM8	chr6-87472246-87474041	0.002038332
H2AFZ	chr4-99087749-99089854	0.002038132
MSRA	chr8-11014900-11017401	0.002037503
FXN	chr9-68546232-68548293	0.002037487
PCSK5	chr9-75868926-75869886	0.002036915
AK5	chr1-77758745-77760343	0.00203679
MSRA	chr8-8994684-8995737	0.002036721
PCSK5	chr9-75086518-75092307	0.002036509
RAP2A	chr13-97432197-97436178	0.002035764
RAP2A	chr13-97386803-97388721	0.002035449
PCSK5	chr9-75147482-75152997	0.002035305
IRAK3	chr12-66129876-66131670	0.002035233
MSRA	chr8-10838624-10840663	0.0020348
EEF1A1	chr6-73388572-73389671	0.002034433
EEF1A1	chr6-73309390-73310769	0.002034378
IRAK3	chr12-66144746-66145723	0.002034148
SMIM8	chr6-87588582-87590865	0.002033984
AK5	chr1-77779018-77780406	0.002033836
MSRA	chr8-9087814-9089370	0.002033649
IRAK3	chr12-65881212-65882697	0.002033372
TSHZ1	chr18-74493415-74501826	0.002033109
EEF1A1	chr6-73262582-73263834	0.002032362
EEF1A1	chr6-73652201-73655699	0.002032268
EEF1A1	chr6-73585115-73586913	0.002031674
EEF1A1	chr6-73695225-73697492	0.002031658
SMIM8	chr6-87689967-87694649	0.002031608
IRAK3	chr12-66287544-66288850	0.002031601
MSRA	chr8-8918976-8920667	0.002031517
EEF1A1	chr6-73421830-73423206	0.002031284
IRAK3	chr12-66290431-66291441	0.002031232
EEF1A1	chr6-73746832-73748116	0.002031108
IRAK3	chr12-66168072-66171119	0.002030901
EEF1A1	chr6-72619706-72623632	0.002030849
TSPAN5	chr4-99855086-99857790	0.002030841
MSRA	chr8-10332517-10335392	0.002030549
IRAK3	chr12-65168504-65172475	0.002030201
AK5	chr1-77975075-77980661	0.00202981
IRAK3	chr12-66268017-66270178	0.002029773
SMIM8	chr6-87695380-87703502	0.002029644
IRAK3	chr12-67268008-67270820	0.002029571
EEF1A1	chr6-73579389-73581336	0.002028895
IRAK3	chr12-66301724-66305101	0.002028689
TSHZ1	chr18-74373017-74374560	0.002028167
IRAK3	chr12-66186907-66191995	0.002028118
MSRA	chr8-9149573-9152197	0.002027867
IRAK3	chr12-66233958-66237404	0.002027834
CYTOR	chr2-88626170-88629719	0.002027571
SMIM8	chr6-87830843-87832818	0.002027208
MSRA	chr8-8891608-8894319	0.002027163

SMIM8	chr6-87716863-87719819	0.002026752
IRAK3	chr12-66964257-66965165	0.00202637
MSRA	chr8-10053374-10055956	0.002026338
IRAK3	chr12-66776655-66777696	0.002026248
EEF1A1	chr6-73449295-73453461	0.002026233
MSRA	chr8-9555135-9557088	0.002024958
MSRA	chr8-8868574-8873109	0.002024891
EEF1A1	chr6-73509779-73524658	0.002024768
SMIM8	chr6-87824709-87826493	0.002024729
AK5	chr1-78003227-78006330	0.002024401
TSPAN5	chr4-98446260-98448056	0.002024153
TSHZ1	chr18-74290764-74293164	0.002023712
CYTOR	chr2-88599566-88600668	0.002023631
SMIM8	chr6-87730302-87733210	0.00202332
TSPAN5	chr4-98657183-98660260	0.00202296
EEF1A1	chr6-73454515-73456698	0.002022293
TSPAN5	chr4-99845356-99848834	0.002022023
SMIM8	chr6-87754597-87757740	0.002021902
EEF1A1	chr6-73460713-73463264	0.002021833
TSPAN5	chr4-98259690-98262950	0.002021682
CYTOR	chr2-88169750-88171316	0.002021496
CYTOR	chr2-86719452-86721584	0.002021259
SMIM8	chr6-87741957-87742832	0.002021221
CYTOR	chr2-88054128-88056846	0.002021022
CYTOR	chr2-86440159-86444277	0.002020607
CYTOR	chr2-88015405-88017801	0.002020568
CYTOR	chr2-86621258-86624719	0.002020567
TSHZ1	chr18-74196049-74196807	0.002019657
CYTOR	chr2-86783559-86792275	0.002019396
TSPAN5	chr4-98662620-98664166	0.002019252
MRTFB	chr16-13956938-13958227	0.002019149
TSHZ1	chr18-74082204-74083683	0.002018637
MRTFB	chr16-14070191-14072306	0.002018378
CYTOR	chr2-86562040-86564322	0.002018321
CYTOR	chr2-86552643-86553642	0.002017472
TSHZ1	chr18-74146985-74149176	0.002017327
MRTFB	chr16-13919204-13921067	0.002017017
CYTOR	chr2-86793413-86795411	0.002016857
TSPAN5	chr4-98142596-98144249	0.002016842
CYTOR	chr2-86824966-86827722	0.002016714
CYTOR	chr2-86805831-86810178	0.002015641
TSPAN5	chr4-98705968-98706981	0.002015226
MRTFB	chr16-15154098-15155549	0.002014241
TSPAN5	chr4-99815343-99818760	0.002013636
MRTFB	chr16-14282295-14283335	0.002013201
MGAT5	chr2-134261404-134263338	0.002012549
MGAT5	chr2-134118151-134123707	0.0020125
TSPAN5	chr4-98927414-98930883	0.002012199
MGAT5	chr2-134282132-134283949	0.002012056
MGAT5	chr2-134717747-134719678	0.002011832

MGAT5	chr2-134737637-134740683	0.002011463
MGAT5	chr2-134258285-134259450	0.002011387
MGAT5	chr2-134668372-134671996	0.002011157
MGAT5	chr2-134288707-134290878	0.002011156
TSPAN5	chr4-98994596-98997059	0.002010028
MGAT5	chr2-134180777-134181948	0.002009943
MGAT5	chr2-134244286-134246540	0.002008915
MGAT5	chr2-134917234-134919840	0.0020085
TSPAN5	chr4-99562731-99564592	0.002008471
TSPAN5	chr4-99043148-99044170	0.002008182
MGAT5	chr2-134216718-134219760	0.002007406
TSPAN5	chr4-99087749-99089854	0.002007118
MGAT5	chr2-134232017-134234395	0.002007003
MRTFB	chr16-14284752-14287973	0.002005059
MRTFB	chr16-15093786-15095160	0.002004881
MGAT5	chr2-135051498-135054164	0.002003284
ANKH	chr5-14705585-14708600	0.001997692
MRTFB	chr16-14300598-14303939	0.001997099
MRTFB	chr16-15053960-15056931	0.001996547
CMSS1	chr3-98762872-98764988	0.001995964
ANKH	chr5-14675525-14676866	0.001995454
ANKH	chr5-14809295-14810555	0.001995025
CMSS1	chr3-98760836-98762268	0.001994575
CMSS1	chr3-98778287-98779543	0.001993026
MRTFB	chr16-14308485-14309940	0.001991695
MRTFB	chr16-15013636-15015076	0.001991262
ANKH	chr5-14663569-14668771	0.001989526
ANKH	chr5-14868929-14873507	0.001989219
MRTFB	chr16-14354325-14355771	0.001989182
MRTFB	chr16-14974274-14976270	0.001988973
CMSS1	chr3-98732058-98734084	0.001988746
MRTFB	chr16-14628770-14634739	0.001988495
CMSS1	chr3-98900756-98902557	0.001988191
SSBP2	chr5-81006262-81008637	0.001986341
SSBP2	chr5-81299310-81302815	0.001986323
CMSS1	chr3-99816809-99819184	0.001984085
RHBDD1	chr2-227370055-227371448	0.001983982
SSBP2	chr5-80959822-80962376	0.001983579
ANKH	chr5-14992352-14994416	0.001983474
ANKH	chr5-14593316-14596481	0.001983183
RHBDD1	chr2-227324285-227326309	0.001983079
SSBP2	chr5-81506771-81507404	0.001982026
RHBDD1	chr2-227384833-227386372	0.00198183
CMSS1	chr3-99875394-99876957	0.00198151
RHBDD1	chr2-226834981-226839644	0.001980547
SSBP2	chr5-80957587-80958731	0.001980364
ANKH	chr5-14996702-14999147	0.001979795
RHBDD1	chr2-226423105-226424194	0.001979327
CMSS1	chr3-99895215-99896820	0.001979235
ANKH	chr5-14586815-14589584	0.001979053

RHBDD1	chr2-226790665-226792806	0.001978758
RHBDD1	chr2-226797983-226803024	0.001978737
ACVR1	chr2-157466757-157471565	0.001978157
MAL	chr2-95022196-95023922	0.001978093
CMSS1	chr3-100708374-100711277	0.00197804
ACVR1	chr2-157463439-157465803	0.001977997
RHBDD1	chr2-226118315-226119426	0.001977793
SSBP2	chr5-80754802-80756555	0.001977704
RHBDD1	chr2-227406096-227408241	0.001977299
ANKH	chr5-14581089-14583296	0.001977261
MAL	chr2-95024881-95027172	0.001976547
ANKH	chr5-14559363-14560928	0.001975935
CMSS1	chr3-100047030-100048542	0.001975792
ACVR1	chr2-157452242-157454506	0.001975045
SSBP2	chr5-80653980-80655800	0.001975003
CMSS1	chr3-100602491-100603941	0.001974767
SSBP2	chr5-81749028-81752447	0.00197441
ACVR1	chr2-157627783-157629583	0.001973813
ANKH	chr5-14412916-14415639	0.001973372
MAL	chr2-95073812-95075834	0.001973331
ACVR1	chr2-157331418-157332472	0.001973278
ACVR1	chr2-157327277-157328322	0.001972734
MAL	chr2-95164903-95166458	0.001972712
ACVR1	chr2-157402819-157405108	0.001972572
RHBDD1	chr2-227414789-227416885	0.001972378
ACVR1	chr2-157426512-157445564	0.001972324
MAL	chr2-95158313-95160608	0.001972169
ACVR1	chr2-157409445-157422458	0.001971664
CMSS1	chr3-100260001-100261885	0.00197128
MAL	chr2-95206932-95208102	0.001971022
MAL	chr2-95076381-95077930	0.001970949
MAL	chr2-95121060-95122508	0.001970844
CMSS1	chr3-100491971-100493008	0.001970326
ANKH	chr5-14330859-14332031	0.001969916
RIPK2	chr8-90000460-90002728	0.001969508
RIPK2	chr8-90005010-90006592	0.00196876
ANKH	chr5-14142218-14145819	0.00196874
RHBDD1	chr2-227436213-227437360	0.001968393
RIPK2	chr8-89981428-89985453	0.001968072
ANKH	chr5-14264469-14265133	0.001967779
CMSS1	chr3-100333948-100335893	0.001967738
MAL	chr2-95401682-95403736	0.001967673
RIPK2	chr8-90644533-90647080	0.001967539
CMSS1	chr3-100399756-100402521	0.001967321
RIPK2	chr8-90466902-90468302	0.001966956
SSBP2	chr5-81778255-81780437	0.001966731
ACVR1	chr2-157874574-157877736	0.00196643
RIPK2	chr8-90627524-90628621	0.001966173
ACVR1	chr2-158967925-158970186	0.001966112
RIPK2	chr8-89974271-89976401	0.001965165

RHBDD1	chr2-227450164-227451854	0.001964728
MAL	chr2-96135787-96160058	0.001964612
MAL	chr2-96115693-96118856	0.001964389
MAL	chr2-96161711-96165419	0.001962996
RIPK2	chr8-89901365-89903641	0.00196246
SSBP2	chr5-82173934-82175010	0.001962363
SSBP2	chr5-81850399-81853360	0.001962173
ATP8B4	chr15-50064248-50067748	0.001961942
SSBP2	chr5-82277158-82279373	0.001961883
SSBP2	chr5-81970699-81973287	0.001961431
ATP8B4	chr15-49619931-49622022	0.001961093
RIPK2	chr8-89887163-89888482	0.001960771
ACVR1	chr2-158455968-158458833	0.001960563
MAL	chr2-96174104-96175144	0.001960093
ACVR1	chr2-157897071-157900361	0.001959873
RHBDD1	chr2-227471497-227473424	0.001959758
ATP8B4	chr15-49045360-49047252	0.001959322
RIPK2	chr8-89836743-89837764	0.001959181
ATP8B4	chr15-50107463-50108572	0.001958998
SSBP2	chr5-82356743-82357918	0.001958768
MAL	chr2-96207208-96210289	0.001958623
ATP8B4	chr15-49169510-49171331	0.001958425
MAL	chr2-96202372-96205567	0.001958067
COX10-AS1	chr17-14427746-14429499	0.001957835
ACVR1	chr2-158142171-158143317	0.001957676
ATP8B4	chr15-49154763-49156615	0.001957253
RIPK2	chr8-89780809-89782808	0.00195616
COX10-AS1	chr17-14604514-14605818	0.001956131
COX10-AS1	chr17-14404178-14405085	0.001955599
NT5DC1	chr6-116100222-116102078	0.00195507
RSAD2	chr2-6909270-6910554	0.001954478
RSAD2	chr2-7866754-7868612	0.001954404
ATP8B4	chr15-50112271-50114883	0.001953313
RSAD2	chr2-6913141-6914394	0.00195314
RSAD2	chr2-6876123-6879209	0.001953025
VOPP1	chr7-56033517-56034814	0.001952956
VOPP1	chr7-56050204-56052637	0.001952877
RHBDD1	chr2-227782939-227784059	0.00195282
COX10-AS1	chr17-14737836-14738999	0.001952135
VOPP1	chr7-55963876-55966055	0.00195206
PATJ	chr1-61049623-61051554	0.001951625
VOPP1	chr7-55951250-55952678	0.0019516
DIP2C	chr10-1061847-1064611	0.001951586
VOPP1	chr7-55886716-55888442	0.001951583
RIPK2	chr8-89776656-89777779	0.001951327
RSAD2	chr2-6863856-6866834	0.001950981
VOPP1	chr7-56063542-56065528	0.001950582
VOPP1	chr7-55568768-55574651	0.00195055
COX10-AS1	chr17-14358956-14360309	0.001950193
PATJ	chr1-61056611-61058995	0.001950184

PATJ	chr1-61042680-61043822	0.001949637
DIP2C	chr10-1109824-1110947	0.001949456
RSAD2	chr2-6916730-6919459	0.001949258
DIP2C	chr10-1055335-1058389	0.001949252
NT5DC1	chr6-116250505-116255501	0.001948848
VOPP1	chr7-54758269-54760328	0.001948592
COX10-AS1	chr17-15237883-15239062	0.00194828
RIPK2	chr8-89725640-89726415	0.001948207
ATP8B4	chr15-50685589-50687695	0.001948182
ATP8B4	chr15-50247560-50248736	0.001948034
ATP8B4	chr15-50764162-50766613	0.001948017
RSAD2	chr2-7448846-7453802	0.001947413
RSAD2	chr2-7430060-7432913	0.001947412
VOPP1	chr7-55532917-55538150	0.001947228
RSAD2	chr2-7773024-7774356	0.0019471
RIPK2	chr8-89755871-89760003	0.001946681
ATP8B4	chr15-50423239-50425495	0.001946621
VOPP1	chr7-56073553-56074912	0.001946582
PATJ	chr1-61076371-61080358	0.001946155
ATP8B4	chr15-50885125-50886349	0.00194593
ATP8B4	chr15-50350024-50357258	0.001945793
RHBDD1	chr2-227817848-227819322	0.001945659
MTHFD2L	chr4-74157328-74159294	0.001945619
IGFBP7	chr4-56465847-56468627	0.001945476
RSAD2	chr2-7427232-7429492	0.001945443
RSAD2	chr2-7007713-7008819	0.001945406
RIPK2	chr8-89728620-89730773	0.00194513
ATP8B4	chr15-51076411-51078031	0.001945076
RSAD2	chr2-7467797-7468793	0.001944985
COX10-AS1	chr17-14351181-14353562	0.001944445
COX10-AS1	chr17-14199589-14202820	0.001944323
IGFBP7	chr4-56504954-56506897	0.001944315
IGFBP7	chr4-56434405-56438050	0.001944198
ATP8B4	chr15-50906658-50909829	0.001944106
RSAD2	chr2-7057705-7059981	0.001944058
COX10-AS1	chr17-14196303-14198267	0.001943969
MTHFD2L	chr4-74206000-74207212	0.001943589
IGFBP7	chr4-56757147-56759641	0.001943499
DIP2C	chr10-127639-128486	0.001943342
IGFBP7	chr4-56654674-56657011	0.0019432
DIP2C	chr10-1047451-1051048	0.001943126
COX10-AS1	chr17-14203328-14204891	0.001943075
VOPP1	chr7-56104560-56108120	0.001943063
MTHFD2L	chr4-74098596-74099995	0.001943019
COX10-AS1	chr17-14068786-14070159	0.001942696
IGFBP7	chr4-56529997-56531607	0.001942672
VOPP1	chr7-55308789-55310128	0.001942662
TRIO	chr5-14705585-14708600	0.001942579
RSAD2	chr2-7725055-7726334	0.001942553
NT5DC1	chr6-116267788-116269269	0.001942519

DIP2C	chr10-134020-137557	0.001942322
IGFBP7	chr4-56544280-56545263	0.001942321
RSAD2	chr2-7567192-7568460	0.001942307
VOPP1	chr7-55473912-55477092	0.001942293
NT5DC1	chr6-116392105-116393862	0.001942154
PATJ	chr1-61080931-61084125	0.001941851
COX10-AS1	chr17-13930746-13932438	0.001941781
NT5DC1	chr6-116386458-116387860	0.001941529
IGFBP7	chr4-56799629-56800610	0.001941426
COX10-AS1	chr17-13017064-13019013	0.001941424
COX10-AS1	chr17-14296685-14305495	0.001941269
COX10-AS1	chr17-14308681-14310444	0.001941221
TRIO	chr5-14675525-14676866	0.001940404
IGFBP7	chr4-56386687-56388724	0.001940325
TRIO	chr5-14809295-14810555	0.001939984
NT5DC1	chr6-116447354-116448322	0.001939607
NT5DC1	chr6-116369325-116372027	0.001939421
NT5DC1	chr6-116275290-116281915	0.001939076
PATJ	chr1-61253317-61255714	0.001938949
DIP2C	chr10-333233-334372	0.00193851
VOPP1	chr7-55468934-55470018	0.001938465
VOPP1	chr7-55364718-55367332	0.001938445
MTHFD2L	chr4-73222417-73223611	0.001937974
MTHFD2L	chr4-74230597-74231596	0.001937897
MTHFD2L	chr4-74056452-74057471	0.001937802
PATJ	chr1-61452971-61454010	0.001937223
MTHFD2L	chr4-73066901-73070894	0.001937216
MTHFD2L	chr4-73256896-73260356	0.00193712
DOCK4	chr7-111081521-111084085	0.001936965
IGFBP7	chr4-56821133-56822304	0.001936879
DOCK4	chr7-111137676-111138709	0.001936484
DIP2C	chr10-1043048-1046564	0.001936212
DOCK4	chr7-111024868-111025735	0.001936184
DOCK4	chr7-111012781-111014116	0.001935956
IGFBP7	chr4-55946711-55950287	0.001935874
NT5DC1	chr6-116461343-116463036	0.00193504
PATJ	chr1-61724280-61725916	0.001935036
TRIO	chr5-14663569-14668771	0.001934638
MTHFD2L	chr4-73704317-73705273	0.001934616
TRIO	chr5-14868929-14873507	0.001934338
DIP2C	chr10-450416-452101	0.001933794
MTHFD2L	chr4-73735063-73735901	0.001933525
DOCK4	chr7-111561053-111563144	0.001933335
NT5DC1	chr6-116615811-116617336	0.001933123
MTHFD2L	chr4-73730772-73732687	0.001932605
LANCL2	chr7-56033517-56034814	0.001932133
NT5DC1	chr6-116570418-116572939	0.001932105
LANCL2	chr7-56050204-56052637	0.001932054
IGFBP7	chr4-56906387-56910011	0.001931613
MTHFD2L	chr4-74338649-74339639	0.00193161

DIP2C	chr10-986856-989383	0.00193139
NT5DC1	chr6-116464827-116465893	0.001931385
NT5DC1	chr6-116667570-116669422	0.001931315
PATJ	chr1-61741661-61743582	0.001931281
LANCL2	chr7-55963876-55966055	0.001931245
LANCL2	chr7-55951250-55952678	0.001930791
LANCL2	chr7-55886716-55888442	0.001930774
NT5DC1	chr6-116475088-116477154	0.001930631
DIP2C	chr10-470065-471198	0.00193044
LANCL2	chr7-56063542-56065528	0.001929784
LANCL2	chr7-55568768-55574651	0.001929752
DIP2C	chr10-943734-945974	0.001929438
DIP2C	chr10-929366-933240	0.001929171
DIP2C	chr10-484326-485760	0.001929165
DOCK4	chr7-112449260-112454443	0.001929163
DIP2C	chr10-804310-806845	0.00192912
TRIO	chr5-14992352-14994416	0.00192875
TRIO	chr5-14593316-14596481	0.00192847
DOCK4	chr7-111986960-111988186	0.001928191
LANCL2	chr7-54758269-54760328	0.001927815
IGFBP7	chr4-56957924-56959055	0.001927642
DOCK4	chr7-112390703-112392641	0.001927595
DOCK4	chr7-112461935-112463091	0.001927536
MTHFD2L	chr4-74364329-74367108	0.001927293
AC053527.2	chr4-74157328-74159294	0.001926839
NT5DC1	chr6-116680275-116683159	0.001926757
PATJ	chr1-61781878-61783879	0.001926499
LANCL2	chr7-55532917-55538150	0.001926466
LANCL2	chr7-56073553-56074912	0.001925826
IGFBP7	chr4-56976023-56980163	0.001925487
MTHFD2L	chr4-74397727-74399523	0.00192528
TRIO	chr5-14996702-14999147	0.001925173
AC053527.2	chr4-74206000-74207212	0.001924828
TRIO	chr5-14586815-14589584	0.001924452
AC053527.2	chr4-74098596-74099995	0.001924265
DOCK4	chr7-112203993-112207663	0.001924258
IGFBP7	chr4-57109421-57111517	0.001924131
MTHFD2L	chr4-74444416-74446004	0.001923984
DOCK4	chr7-112789485-112791108	0.001923851
DOCK4	chr7-112080242-112082630	0.001923634
TRIO	chr5-14581089-14583296	0.001922709
PATJ	chr1-62271748-62272723	0.0019225
LANCL2	chr7-56104560-56108120	0.001922343
DOCK4	chr7-112083967-112085223	0.001922204
LANCL2	chr7-55308789-55310128	0.001921947
LANCL2	chr7-55473912-55477092	0.001921583
TRIO	chr5-14559363-14560928	0.00192142
DOCK4	chr7-112937706-112941087	0.001920557
PATJ	chr1-62434583-62438598	0.001920556
PATJ	chr1-62783197-62785101	0.001920404

PATJ	chr1-62687218-62689056	0.001920361
PATJ	chr1-62800206-62801529	0.001919744
AC053527.2	chr4-73222417-73223611	0.001919267
AC053527.2	chr4-74230597-74231596	0.00191919
AC053527.2	chr4-74056452-74057471	0.001919097
DOCK4	chr7-113116984-113119291	0.001919013
TRIO	chr5-14412916-14415639	0.001918928
AC053527.2	chr4-73066901-73070894	0.001918516
AC053527.2	chr4-73256896-73260356	0.001918422
SDHAF3	chr7-97871524-97873058	0.001917913
SDHAF3	chr7-96320513-96322826	0.001917872
LANCL2	chr7-55468934-55470018	0.001917796
LANCL2	chr7-55364718-55367332	0.001917776
SDHAF3	chr7-96285198-96286435	0.001917518
CMAS	chr12-21437056-21439100	0.001917407
SDHAF3	chr7-97116059-97118689	0.001916879
SDHAF3	chr7-97971363-97972954	0.001916783
SDHAF3	chr7-96326567-96327287	0.001916519
AC053527.2	chr4-73704317-73705273	0.001915942
SDHAF3	chr7-96709196-96710336	0.001915927
DZIP3	chr3-108123890-108129274	0.001915821
TRIO	chr5-14330859-14332031	0.001915567
CMAS	chr12-21456094-21457068	0.001915425
DZIP3	chr3-108099102-108103284	0.001915313
AC053527.2	chr4-73735063-73735901	0.001914862
TRIO	chr5-14142218-14145819	0.001914423
PIGK	chr1-77279844-77283411	0.001914261
AC053527.2	chr4-73730772-73732687	0.001913952
TRIO	chr5-14264469-14265133	0.001913489
DZIP3	chr3-108130165-108134966	0.00191317
AC053527.2	chr4-74338649-74339639	0.001912965
SDHAF3	chr7-98106144-98107863	0.001912627
PIGK	chr1-77218646-77220059	0.001912214
CMAS	chr12-21500649-21502632	0.001912093
PIGK	chr1-77312130-77314107	0.001911873
DZIP3	chr3-108087127-108094053	0.001910691
DZIP3	chr3-108219331-108225119	0.001909974
SH3RF1	chr4-169659333-169660983	0.001909905
CMAS	chr12-22543152-22545258	0.001909631
ZFAND1	chr8-80864513-80865320	0.00190953
TJP2	chr9-68844041-68845463	0.001909155
CMAS	chr12-21527289-21528354	0.001908961
ZFAND1	chr8-80872445-80875091	0.001908955
AC053527.2	chr4-74364329-74367108	0.001908689
CMAS	chr12-22624450-22627692	0.001908404
PIGK	chr1-76294343-76295489	0.001908208
SH3RF1	chr4-169756793-169758626	0.001908091
DZIP3	chr3-108588499-108590983	0.001908066
ZFAND1	chr8-81080104-81082721	0.001907751
TJP2	chr9-68974186-68976341	0.001907714

SH3RF1	chr4-169618226-169622990	0.001907294
CMAS	chr12-21614000-21615972	0.001907131
DZIP3	chr3-108602226-108604233	0.00190706
ZFAND1	chr8-81093005-81095383	0.001906871
SDHAF3	chr7-98131379-98135067	0.00190678
PIGK	chr1-76153899-76155279	0.001906769
AC053527.2	chr4-74397727-74399523	0.001906695
TJP2	chr9-68779060-68781168	0.001906643
CMAS	chr12-22538870-22539755	0.001906323
PIGK	chr1-76073982-76076615	0.001906122
CMAS	chr12-21656760-21658488	0.001906062
NUBPL	chr14-32200396-32204580	0.001905777
ZFAND1	chr8-81096614-81098869	0.001905748
PIGK	chr1-76267444-76268498	0.001905585
PIGK	chr1-76270199-76271253	0.001905556
AC053527.2	chr4-74444416-74446004	0.001905413
PIGK	chr1-77412060-77414280	0.001905396
DZIP3	chr3-108805963-108807000	0.001905135
TJP2	chr9-70257533-70261077	0.00190503
CMAS	chr12-21676611-21678097	0.001904346
WDR27	chr6-169212607-169215519	0.001904006
DZIP3	chr3-108061886-108062786	0.001903899
SH3RF1	chr4-170025503-170027967	0.001903738
TJP2	chr9-69027387-69029311	0.001903699
WDR27	chr6-168883965-168885217	0.001903311
ZFAND1	chr8-81105512-81112926	0.001902758
CMAS	chr12-22729581-22730610	0.001902715
WDR27	chr6-169654271-169655513	0.001902295
DZIP3	chr3-107429640-107432570	0.001902036
SDHAF3	chr7-98137448-98138444	0.001901506
TJP2	chr9-68704576-68707052	0.001901468
CMAS	chr12-22045881-22047206	0.001901447
CMAS	chr12-22409056-22410708	0.001901381
SH3RF1	chr4-169611666-169613825	0.001901303
NUBPL	chr14-31205652-31209084	0.001901266
DZIP3	chr3-108820606-108822984	0.001901207
SLAIN1	chr13-78658165-78660549	0.001900718
NUBPL	chr14-32075673-32079121	0.001900496
NUBPL	chr14-31419456-31421315	0.001900376
TJP2	chr9-69945317-69947357	0.001900212
TJP2	chr9-69035230-69037047	0.001899963
NUBPL	chr14-31127382-31129291	0.001899823
WDR27	chr6-169700118-169703679	0.001899617
TJP2	chr9-69173550-69175924	0.001899345
DZIP3	chr3-107522277-107526444	0.00189912
TJP2	chr9-69671217-69673504	0.00189897
TJP2	chr9-69168386-69169602	0.001898807
CMAS	chr12-22332848-22336188	0.001898677
TJP2	chr9-69758793-69760657	0.001898564
DZIP3	chr3-107927263-107928438	0.001898523

TJP2	chr9-69120684-69123032	0.001898431
CMAS	chr12-22396102-22397233	0.001898297
SDHAF3	chr7-98202136-98204049	0.001898164
WDR27	chr6-169722466-169725736	0.001897882
COQ7	chr16-19883415-19886764	0.001897735
PIGK	chr1-77430672-77432282	0.001897665
COQ7	chr16-19554377-19556966	0.00189766
NUBPL	chr14-31456669-31458162	0.001897604
NUBPL	chr14-31123969-31124924	0.001897542
ZFAND1	chr8-81279857-81282005	0.001897482
WDR27	chr6-169750791-169752884	0.001897472
ZFAND1	chr8-81129945-81131813	0.001897112
ZFAND1	chr8-81685667-81686666	0.001897042
DZIP3	chr3-107598701-107600144	0.001897039
COQ7	chr16-19695840-19697368	0.001896831
WDR27	chr6-169789709-169790977	0.00189675
COQ7	chr16-19520328-19525611	0.001896617
TJP2	chr9-68559719-68561004	0.001896611
DZIP3	chr3-108833328-108835750	0.001896434
SDHAF3	chr7-98210428-98214749	0.001896297
COQ7	chr16-19717435-19719103	0.001896278
NUBPL	chr14-31944146-31945638	0.00189624
NUBPL	chr14-31090402-31091370	0.001896142
ABLIM1	chr10-114821079-114823214	0.001895917
NUBPL	chr14-31067948-31068757	0.001895887
NUBPL	chr14-31023930-31027630	0.00189568
ABLIM1	chr10-114937714-114938898	0.001895506
NUBPL	chr14-31559978-31562720	0.001895397
ABLIM1	chr10-114542903-114545289	0.00189535
SH3RF1	chr4-169270050-169272695	0.001895252
SLAIN1	chr13-78601344-78604086	0.001894882
SDHAF3	chr7-98250355-98253671	0.00189466
ZFAND1	chr8-81720437-81721905	0.001894568
NUBPL	chr14-30873980-30875637	0.001894384
TJP2	chr9-68546232-68548293	0.001894337
ZFAND1	chr8-81234152-81235843	0.001894301
RPS6	chr9-19048195-19051504	0.001894061
SH3RF1	chr4-169008705-169011192	0.001893702
WDR27	chr6-170048170-170050415	0.001893484
SH3RF1	chr4-168880971-168882200	0.001893473
COQ7	chr16-19513388-19514319	0.001893123
SDHAF3	chr7-98275962-98276934	0.001892865
SH3RF1	chr4-168317417-168319238	0.001892645
SH3RF1	chr4-169079108-169080859	0.001892356
NUBPL	chr14-30620082-30623037	0.001892351
ABLIM1	chr10-114538163-114542279	0.001892221
SH3RF1	chr4-169210477-169211744	0.001892142
SDHAF3	chr7-98280302-98288504	0.001892098
C19orf12	chr19-29711498-29717167	0.001891582
PIGK	chr1-77681420-77684246	0.001891573

COQ7	chr16-19086038-19087265	0.001891459
NUBPL	chr14-30558438-30560151	0.001891257
SH3RF1	chr4-168877592-168879773	0.001890888
RPS6	chr9-19102032-19103833	0.001890842
SH3RF1	chr4-168479206-168483115	0.001890731
WDR27	chr6-170583720-170585562	0.001890293
ZFAND1	chr8-81132417-81133457	0.001890276
RPS6	chr9-19925872-19926618	0.001890096
COQ7	chr16-19113471-19114998	0.001890061
C19orf12	chr19-29694322-29695335	0.001889861
COQ7	chr16-19066090-19069738	0.00188973
ABLIM1	chr10-114024605-114026773	0.001889539
C19orf12	chr19-29810332-29813391	0.001889203
RPS6	chr9-19464466-19465433	0.001889065
ZFAND1	chr8-81160829-81161951	0.001888953
COQ7	chr16-19210157-19211468	0.001888919
ABLIM1	chr10-114042784-114045908	0.00188883
SLAIN1	chr13-78022038-78022823	0.001888753
ABLIM1	chr10-113965014-113966360	0.001888541
ANK3	chr10-60777818-60779450	0.001888466
RPS6	chr9-19125600-19129245	0.001888141
ANK3	chr10-60731197-60734091	0.00188803
PIGK	chr1-77758745-77760343	0.001887942
SH3RF1	chr4-168502833-168504670	0.001887781
SH3RF1	chr4-168831068-168833338	0.001887641
COQ7	chr16-19117049-19119815	0.001887479
ABLIM1	chr10-113960208-113962273	0.001887238
RPS6	chr9-20209265-20210451	0.001887182
WDR27	chr6-170086851-170090364	0.001887114
ABLIM1	chr10-114522139-114528088	0.001887029
COQ7	chr16-19167497-19169185	0.001886633
WDR27	chr6-170552207-170555136	0.00188648
ABLIM1	chr10-113853648-113855981	0.00188634
SH3RF1	chr4-168509490-168510403	0.001886275
ABLIM1	chr10-114060371-114062246	0.001885915
ANK3	chr10-60942700-60945053	0.001885832
COQ7	chr16-18925141-18927677	0.001885547
ZFAND1	chr8-81140331-81141844	0.001885385
ABLIM1	chr10-113678842-113682483	0.001885233
PIGK	chr1-77779018-77780406	0.001885205
RPS6	chr9-19153791-19154726	0.001885109
C19orf12	chr19-29690607-29691413	0.001885036
ZFAND1	chr8-81153577-81155209	0.001885007
RPS6	chr9-19407663-19410033	0.00188451
C19orf12	chr19-29830821-29832066	0.001884201
SLAIN1	chr13-77695870-77699587	0.001883911
ANK3	chr10-60727688-60728745	0.0018838
AGAP1	chr2-234463500-234465878	0.001883549
RPS6	chr9-20242172-20244870	0.001882744
ANK3	chr10-61388953-61391372	0.001882742

ABLIM1	chr10-114172255-114175331	0.001882265
ABLIM1	chr10-114498376-114500288	0.00188225
AGAP1	chr2-234482741-234484879	0.001881978
WDR27	chr6-170305673-170308261	0.001881917
COQ7	chr16-18799899-18802830	0.001881729
AGAP1	chr2-234449865-234456022	0.001881626
PIGK	chr1-77975075-77980661	0.001881473
ANK3	chr10-61486607-61487319	0.00188139
ANK3	chr10-61397033-61398235	0.001881344
C19orf12	chr19-29211659-29213920	0.00188132
RPS6	chr9-19161160-19164014	0.001881304
C19orf12	chr19-29605066-29607200	0.00188099
COQ7	chr16-18788608-18791565	0.00188079
C19orf12	chr19-29609805-29611362	0.001880767
RPS6	chr9-20321417-20322391	0.001880503
ANK3	chr10-61867269-61868653	0.001880474
ABLIM1	chr10-114303876-114305395	0.001880468
SLAIN1	chr13-77477962-77479031	0.001880104
RPS6	chr9-20314541-20316212	0.001880041
C19orf12	chr19-29679525-29682272	0.001880029
C19orf12	chr19-29620898-29622865	0.001879538
RPS6	chr9-19377248-19381267	0.001879353
C19orf12	chr19-29837139-29838153	0.001879178
AGAP1	chr2-234443715-234448875	0.001878631
AGAP1	chr2-234307512-234309597	0.001877828
RPS6	chr9-19183778-19185045	0.001877801
C19orf12	chr19-29663030-29669654	0.001877795
ANK3	chr10-60666045-60666738	0.001877708
C19orf12	chr19-29671100-29678958	0.001877438
AGAP1	chr2-234420202-234428212	0.00187737
AGAP1	chr2-234436389-234439159	0.001877061
WDR27	chr6-170288468-170297824	0.001876922
RPS6	chr9-19229689-19233160	0.001876723
AGAP1	chr2-234487469-234501036	0.001876675
PIGK	chr1-78003227-78006330	0.001876459
AGAP1	chr2-234288645-234294392	0.001876423
ANK3	chr10-61896674-61898266	0.001876356
SLAIN1	chr13-77338449-77339831	0.001876216
LINC00926	chr15-56242760-56246795	0.001876186
C19orf12	chr19-29841652-29846326	0.001875711
WDR27	chr6-170271021-170273355	0.001874419
SLAIN1	chr13-76883594-76887384	0.001874043
WDR27	chr6-170280008-170281168	0.001873647
SLAIN1	chr13-76879112-76880377	0.001873544
C19orf12	chr19-29872115-29873981	0.001873531
ANK3	chr10-60236621-60238919	0.001872651
LINC00926	chr15-56364926-56365841	0.001872228
SLAIN1	chr13-76979831-76980576	0.001871951
SLAIN1	chr13-77328508-77330673	0.001871855
TAPT1-AS1	chr4-15781347-15782504	0.00187143

C19orf12	chr19-29941665-29943269	0.0018714
SLAIN1	chr13-76799439-76800163	0.001871391
TAPT1-AS1	chr4-15777232-15780300	0.001870871
TAPT1-AS1	chr4-15961666-15964092	0.001870628
ANK3	chr10-60031489-60033243	0.001870147
SLAIN1	chr13-76744909-76746300	0.001870057
AGAP1	chr2-234951310-234953050	0.001869976
TAPT1-AS1	chr4-16224011-16228982	0.001869674
ANK3	chr10-59991447-59992317	0.001869228
ANK3	chr10-61898925-61905400	0.001869176
LINC00926	chr15-56464565-56465649	0.001868947
SLAIN1	chr13-76990671-76993536	0.001868767
TAPT1-AS1	chr4-16237543-16238685	0.001868394
SLAIN1	chr13-77323125-77327735	0.001868214
LINC00926	chr15-56877165-56878294	0.001868133
LINC00926	chr15-56885432-56888441	0.001868007
LINC00926	chr15-56732018-56734271	0.001867789
ANK3	chr10-59903892-59908895	0.001867758
TAPT1-AS1	chr4-15765411-15767079	0.001867393
SLAIN1	chr13-77025051-77028380	0.001867026
AGAP1	chr2-236567450-236570238	0.001866281
AGAP1	chr2-236550680-236553327	0.001866219
LINC00926	chr15-58329770-58333360	0.001866174
LINC00926	chr15-56917471-56920702	0.001866148
LINC00926	chr15-58247575-58249125	0.001865989
TAPT1-AS1	chr4-17336377-17337306	0.001865925
AGAP1	chr2-236544816-236547301	0.001865606
EPS8	chr12-15590057-15590938	0.00186551
EPS8	chr12-15570710-15571788	0.001865177
AGAP1	chr2-235043778-235045963	0.001865001
LINC00926	chr15-58472800-58474264	0.00186468
AGAP1	chr2-236535973-236539148	0.001864202
LINC00926	chr15-58146211-58147801	0.001863905
LINC00926	chr15-58484430-58486333	0.001863556
AGAP1	chr2-235050836-235051833	0.001863328
LINC00926	chr15-57298382-57300641	0.001863204
EPS8	chr12-15544421-15548127	0.001862975
EPS8	chr12-15788128-15790398	0.001862244
LINC00926	chr15-58139627-58140659	0.001861624
LINC00926	chr15-57705868-57707757	0.001861214
TAPT1-AS1	chr4-15753765-15757509	0.001860877
EPS8	chr12-15534066-15535737	0.001860604
EPS8	chr12-14993509-14996248	0.001858387
C11orf65	chr11-107927053-107930238	0.001856528
SMC4	chr3-160129352-160130248	0.001855705
EPS8	chr12-15881176-15883880	0.001855569
C11orf65	chr11-107857890-107860048	0.001855526
C11orf65	chr11-107956898-107957798	0.001855474
EPS8	chr12-14980812-14984939	0.001855259
C11orf65	chr11-108120871-108122689	0.001854891

SMC4	chr3-159988141-159989532	0.001854789
C11orf65	chr11-108220754-108227272	0.001854289
C11orf65	chr11-107988071-107989143	0.001854148
C11orf65	chr11-108008245-108010468	0.001854098
SMC4	chr3-160225020-160226917	0.001854074
TAPT1-AS1	chr4-15702543-15704214	0.00185341
TAPT1-AS1	chr4-15653381-15657651	0.001853021
SMC4	chr3-159928159-159930308	0.00185297
C11orf65	chr11-107840130-107841498	0.001852377
TAPT1-AS1	chr4-15659486-15660892	0.001852095
STX18	chr4-3368381-3370638	0.001852
TAPT1-AS1	chr4-15649683-15650588	0.00185188
SMC4	chr3-159762787-159765472	0.00185185
ACVR2A	chr2-148019810-148022204	0.001851292
TAPT1-AS1	chr4-15399939-15401926	0.001850783
ACVR2A	chr2-147843457-147846678	0.001850686
C11orf54	chr11-93196692-93198813	0.001850417
EPS8	chr12-14955808-14963795	0.001850413
C11orf65	chr11-108235643-108238081	0.001850376
SMC4	chr3-160398427-160402339	0.001850131
C11orf54	chr11-93523217-93527402	0.00185001
NELL2	chr12-45725439-45733443	0.001850009
NELL2	chr12-45983573-45985597	0.001849344
ACVR2A	chr2-148508911-148510078	0.001849335
TAPT1-AS1	chr4-15665482-15666704	0.001849278
C11orf54	chr11-93537359-93538454	0.001849147
C11orf65	chr11-107564831-107566333	0.001848916
NELL2	chr12-45668675-45671173	0.001848794
C11orf54	chr11-94512079-94513448	0.001848724
NELL2	chr12-45989246-45992711	0.001848623
NELL2	chr12-45986320-45987316	0.001848506
TAPT1-AS1	chr4-15689340-15692116	0.001848166
EPS8	chr12-15910695-15912059	0.001847833
ACVR2A	chr2-147689223-147690331	0.001847429
C11orf54	chr11-94543088-94546242	0.001847394
SMC4	chr3-160801222-160802568	0.00184737
SMC4	chr3-160980712-160981849	0.00184734
C11orf65	chr11-107457164-107458547	0.001847216
TAPT1-AS1	chr4-15681017-15683559	0.001847028
ACVR2A	chr2-148524069-148525437	0.001846864
IFT80	chr3-160129352-160130248	0.001846832
ACVR2A	chr2-148545254-148546863	0.001846757
ACVR2A	chr2-148538378-148540776	0.001846586
C11orf54	chr11-94492895-94494703	0.001846556
SMC4	chr3-160448681-160450778	0.001846092
ACVR2A	chr2-148526382-148529763	0.001845948
IFT80	chr3-159988141-159989532	0.00184592
C11orf54	chr11-93541180-93544078	0.001845914
SMC4	chr3-160754231-160757791	0.001845439
STX18	chr4-3372100-3374651	0.001845308

IFT80	chr3-160225020-160226917	0.00184521
NELL2	chr12-45655086-45656166	0.001845176
C11orf65	chr11-108994654-108995446	0.001844935
DPP4	chr2-161307432-161309606	0.001844547
C11orf54	chr11-94583511-94584773	0.001844521
EPS8	chr12-14948045-14954989	0.001844506
SMC4	chr3-160563993-160566731	0.001844373
ACVR2A	chr2-148552202-148554314	0.001844205
IFT80	chr3-159928159-159930308	0.001844111
NELL2	chr12-44874887-44877228	0.001843984
SMC4	chr3-161034939-161036435	0.00184397
DPP4	chr2-161242902-161245956	0.00184389
STX18	chr4-3484135-3487240	0.00184379
C11orf65	chr11-108465430-108468912	0.001843714
ACVR2A	chr2-147663091-147664492	0.001843585
NELL2	chr12-44846908-44849095	0.001843315
IFT80	chr3-159762787-159765472	0.001842996
STX18	chr4-3442888-3444882	0.001842769
DPP4	chr2-161414274-161417345	0.001842511
STX18	chr4-3528384-3533528	0.001842286
C11orf54	chr11-94638267-94639580	0.001842262
ACVR2A	chr2-147630322-147632625	0.001842021
EPS8	chr12-16347276-16349614	0.00184199
NELL2	chr12-45200127-45201221	0.001841671
STX18	chr4-4348083-4349154	0.00184166
ACVR2A	chr2-147635750-147637537	0.001841576
C11orf54	chr11-94767897-94769568	0.001841297
IFT80	chr3-160398427-160402339	0.001841285
STX18	chr4-3384599-3386247	0.0018412
C11orf54	chr11-94127820-94131663	0.001841161
DPP4	chr2-161236903-161239774	0.001840997
STX18	chr4-3387284-3390124	0.001840917
NELL2	chr12-45244268-45245360	0.001840728
STX18	chr4-4385804-4388238	0.001840639
C11orf54	chr11-93654255-93655686	0.001840303
STX18	chr4-4288877-4291247	0.001840027
GDE1	chr16-20674048-20676732	0.001839857
NELL2	chr12-44827621-44828613	0.00183983
DPY30	chr2-32062079-32064853	0.00183955
EPS8	chr12-14884262-14885094	0.001839478
DPY30	chr2-32355757-32358764	0.001839467
STX18	chr4-3954453-3956838	0.001839277
DPP4	chr2-161952564-161954609	0.00183914
NELL2	chr12-45204736-45206558	0.00183874
ACVR2A	chr2-148613876-148615098	0.001838718
DPY30	chr2-32276948-32279033	0.001838674
DPY30	chr2-32132721-32133508	0.001838634
IFT80	chr3-160801222-160802568	0.001838538
IFT80	chr3-160980712-160981849	0.001838508
C11orf65	chr11-108663514-108666598	0.00183843

SMC4	chr3-161220658-161223168	0.001838406
NELL2	chr12-45214754-45217461	0.001838092
DPY30	chr2-32484587-32485642	0.001837931
STX18	chr4-4268031-4270980	0.001837727
C11orf65	chr11-108496574-108499055	0.001837492
DPP4	chr2-161169585-161171992	0.001837454
DPY30	chr2-32039049-32040500	0.001837338
IFT80	chr3-160448681-160450778	0.001837265
STX18	chr4-4540786-4542945	0.001837256
STX18	chr4-4247425-4249424	0.001837208
EPS8	chr12-14802351-14804603	0.001836714
GDE1	chr16-19883415-19886764	0.001836663
NELL2	chr12-43758076-43760654	0.001836619
IFT80	chr3-160754231-160757791	0.001836616
GDE1	chr16-19554377-19556966	0.00183659
DPY30	chr2-32264227-32266101	0.00183659
DPY30	chr2-32165053-32166622	0.001836424
DPP4	chr2-161992287-161993616	0.001836356
NELL2	chr12-43835259-43837035	0.001836063
GDE1	chr16-19695840-19697368	0.001835787
EPS8	chr12-14773379-14776934	0.001835676
GDE1	chr16-19520328-19525611	0.001835581
IFT80	chr3-160563993-160566731	0.001835556
DPY30	chr2-31218914-31220301	0.001835481
DPY30	chr2-32261849-32262783	0.001835426
C11orf54	chr11-93783534-93785408	0.001835379
C11orf65	chr11-108592579-108594316	0.001835288
GDE1	chr16-19717435-19719103	0.001835252
IFT80	chr3-161034939-161036435	0.001835153
DPY30	chr2-32626967-32628731	0.001835022
DPP4	chr2-162011024-162014508	0.001834979
C11orf54	chr11-93660811-93663153	0.001834828
NELL2	chr12-43805268-43807822	0.001834786
DPP4	chr2-161165415-161167472	0.0018344
EPS8	chr12-14768761-14772476	0.001834113
DPP4	chr2-162072480-162074822	0.001834074
STX18	chr4-4574521-4576143	0.001833598
SMC4	chr3-161310478-161311765	0.001833292
C11orf54	chr11-93739838-93742532	0.001832721
DPY30	chr2-32008455-32012190	0.001832606
ACVR2A	chr2-148642706-148647537	0.001832563
DPY30	chr2-31233619-31234929	0.001832555
GDE1	chr16-19513388-19514319	0.001832199
DPP4	chr2-162260797-162262052	0.001831971
STX18	chr4-4943596-4944553	0.001831603
DPP4	chr2-161159740-161161913	0.001831502
SMC4	chr3-161371468-161373209	0.001830651
GDE1	chr16-19086038-19087265	0.001830586
SMC4	chr3-161394089-161395853	0.00183057
IFT80	chr3-161220658-161223168	0.001829616

GDE1	chr16-19113471-19114998	0.001829232
GDE1	chr16-19066090-19069738	0.001828914
DPY30	chr2-31318295-31320289	0.001828738
ACVR2A	chr2-148874564-148877463	0.001828648
DPP4	chr2-162316701-162320247	0.001828153
GDE1	chr16-19210157-19211468	0.00182813
DPY30	chr2-31980565-31981444	0.001828119
ACVR2A	chr2-148926145-148927688	0.001828048
DPP4	chr2-161134884-161140086	0.001827586
GDE1	chr16-19117049-19119815	0.001826734
DPY30	chr2-31955897-31958636	0.00182663
GDE1	chr16-19167497-19169185	0.001825915
GDE1	chr16-18925141-18927677	0.001824866
IFT80	chr3-161310478-161311765	0.001824526
DPP4	chr2-162342764-162345452	0.001824152
ADPRM	chr17-10149760-10152809	0.001824029
ADPRM	chr17-10126716-10128505	0.001822935
FMN1	chr15-33194167-33195609	0.001822235
DPP4	chr2-161076868-161078971	0.001822225
ADPRM	chr17-9575258-9577584	0.001822196
ADPRM	chr17-10033051-10035710	0.001821941
IFT80	chr3-161371468-161373209	0.001821898
IFT80	chr3-161394089-161395853	0.001821817
ADPRM	chr17-10156011-10157998	0.001821446
ADPRM	chr17-10017267-10022890	0.001821436
GDE1	chr16-18799899-18802830	0.001821172
FMN1	chr15-33154130-33155402	0.001821063
ADPRM	chr17-10013088-10014246	0.001820733
ADPRM	chr17-10059102-10061687	0.001820365
GDE1	chr16-18788608-18791565	0.001820262
ADPRM	chr17-10729108-10730687	0.001820145
DERA	chr12-15590057-15590938	0.001819734
FMN1	chr15-33226102-33227222	0.001819563
DERA	chr12-15570710-15571788	0.001819409
MYO9A	chr15-72116614-72119504	0.001819138
ADPRM	chr17-10118755-10121233	0.001819065
TGS1	chr8-55917258-55920151	0.00181811
SLC30A6	chr2-32062079-32064853	0.001817811
SLC30A6	chr2-32355757-32358764	0.001817729
FMN1	chr15-33152374-33153460	0.001817614
TGS1	chr8-55887981-55891956	0.001817439
DERA	chr12-15544421-15548127	0.001817261
ADPRM	chr17-10171100-10173168	0.001817218
ADPRM	chr17-10062624-10064745	0.001817155
SLC30A6	chr2-32276948-32279033	0.001816946
SLC30A6	chr2-32132721-32133508	0.001816905
TGS1	chr8-55924111-55925731	0.001816836
DERA	chr12-15788128-15790398	0.001816548
SLC30A6	chr2-32484587-32485642	0.001816211
NUCB2	chr11-17206598-17210051	0.001816053

TGS1	chr8-55878674-55886699	0.001815964
ADPRM	chr17-10696528-10700487	0.001815893
SLC30A6	chr2-32039049-32040500	0.001815625
ADPRM	chr17-10117078-10118185	0.00181548
NUCB2	chr11-17229983-17231485	0.001815226
DERA	chr12-15534066-15535737	0.001814948
SLC30A6	chr2-32264227-32266101	0.001814886
ADPRM	chr17-10113073-10116563	0.001814793
SLC30A6	chr2-32165053-32166622	0.001814722
ADPRM	chr17-10197436-10199437	0.001814681
FMN1	chr15-33150849-33151663	0.001814492
MYO9A	chr15-72198952-72200987	0.00181446
TGS1	chr8-55863605-55866433	0.001814434
FMN1	chr15-34037902-34040260	0.001814001
SLC30A6	chr2-31218914-31220301	0.001813788
SLC30A6	chr2-32261849-32262783	0.001813735
NUCB2	chr11-17097662-17099188	0.001813726
TGS1	chr8-55929235-55931181	0.001813543
EBF1	chr5-159331029-159333277	0.001813469
SLC30A6	chr2-32626967-32628731	0.001813336
FMN1	chr15-32908063-32909526	0.001813293
FMN1	chr15-32866482-32867745	0.001813254
FMN1	chr15-33125896-33127001	0.001813212
STX18-AS1	chr4-3368381-3370638	0.00181283
DERA	chr12-14993509-14996248	0.001812785
FMN1	chr15-32675165-32676026	0.001812272
TGS1	chr8-55843307-55845942	0.001812229
EBF1	chr5-159337197-159338545	0.001811435
NUCB2	chr11-17275279-17279922	0.001811203
EBF1	chr5-159262308-159264850	0.00181096
SLC30A6	chr2-32008455-32012190	0.00181095
SLC30A6	chr2-31233619-31234929	0.001810897
NUCB2	chr11-17075863-17078939	0.001810872
NUCB2	chr11-16737588-16740675	0.001810539
DERA	chr12-15881176-15883880	0.001810037
TGS1	chr8-55938371-55941027	0.001809817
TGS1	chr8-55988863-55992423	0.001809814
TGS1	chr8-56005010-56007385	0.001809804
DERA	chr12-14980812-14984939	0.001809733
NUCB2	chr11-18392356-18397112	0.001809691
TSC22D1	chr13-45464040-45466139	0.001809439
MYO9A	chr15-72226241-72232997	0.001808926
FMN1	chr15-34100589-34102774	0.001808402
TGS1	chr8-55975069-55976652	0.001808304
TGS1	chr8-55772149-55774233	0.001808184
TSC22D1	chr13-44917091-44919325	0.001808181
TSC22D1	chr13-45416477-45419258	0.001808169
TGS1	chr8-55971141-55974128	0.001807823
MYO9A	chr15-73051178-73053582	0.001807214
SLC30A6	chr2-31318295-31320289	0.001807127

NUCB2	chr11-18105485-18106575	0.001807079
TGS1	chr8-56073243-56075461	0.001806905
CPQ	chr8-96759793-96762252	0.001806598
SLC30A6	chr2-31980565-31981444	0.001806515
NUCB2	chr11-17285259-17287554	0.001806469
CPQ	chr8-96644542-96647010	0.001806395
TSC22D1	chr13-44575177-44578572	0.00180635
STX18-AS1	chr4-3372100-3374651	0.001806279
NUCB2	chr11-18277478-18278491	0.001806132
TSC22D1	chr13-44988026-44990457	0.001806128
NUCB2	chr11-18010093-18014387	0.001806097
EBF1	chr5-159340473-159344641	0.001805948
NUCB2	chr11-18382510-18385040	0.001805909
RNF144A	chr2-8090639-8091585	0.001805851
EBF1	chr5-159206337-159211020	0.001805775
TSC22D1	chr13-45366897-45368410	0.001805683
FMN1	chr15-34318208-34319669	0.00180533
FMN1	chr15-34209350-34210660	0.001805288
SLC30A6	chr2-31955897-31958636	0.001805043
DERA	chr12-14955808-14963795	0.001805006
FMN1	chr15-34223885-34225892	0.001804914
STX18-AS1	chr4-3484135-3487240	0.001804794
MYO9A	chr15-72236973-72238103	0.001804723
NUCB2	chr11-18280261-18281333	0.001804356
FMN1	chr15-34331191-34338704	0.001804222
NUCB2	chr11-17388547-17390255	0.001804214
CPQ	chr8-96820455-96821318	0.001804136
NUCB2	chr11-18320368-18324924	0.001803884
NUCB2	chr11-17351018-17354922	0.001803828
STX18-AS1	chr4-3442888-3444882	0.001803794
TSC22D1	chr13-43877529-43880681	0.001803664
STX18-AS1	chr4-3528384-3533528	0.001803322
MYO9A	chr15-72271632-72273998	0.001802944
CPQ	chr8-96570625-96571547	0.001802943
CHPT1	chr12-101875254-101879050	0.001802769
MYO9A	chr15-72305978-72307179	0.001802751
STX18-AS1	chr4-4348083-4349154	0.001802709
DERA	chr12-15910695-15912059	0.001802491
TSC22D1	chr13-44140933-44143959	0.001802367
TGS1	chr8-56209284-56213092	0.001802317
STX18-AS1	chr4-3384599-3386247	0.001802258
MYO9A	chr15-72318541-72320897	0.001802246
TMEM220	chr17-10149760-10152809	0.001802076
TSC22D1	chr13-45329216-45342771	0.001802055
STX18-AS1	chr4-3387284-3390124	0.001801981
TSC22D1	chr13-45046861-45048063	0.00180196
TSC22D1	chr13-44434476-44438430	0.00180179
CHPT1	chr12-101917416-101918161	0.001801773
STX18-AS1	chr4-4385804-4388238	0.001801709
CHPT1	chr12-101838492-101840373	0.001801289

EBF1	chr5-159197953-159199630	0.001801265
CPQ	chr8-96852071-96855278	0.001801194
FMN1	chr15-34341376-34343678	0.001801158
STX18-AS1	chr4-4288877-4291247	0.00180111
TMEM220	chr17-10126716-10128505	0.001800994
EBF1	chr5-159096840-159101334	0.001800747
STX18-AS1	chr4-3954453-3956838	0.001800378
MYO9A	chr15-72375029-72377721	0.001800273
TMEM220	chr17-9575258-9577584	0.001800264
EBF1	chr5-159362005-159362817	0.00180024
EBF1	chr5-159104347-159107268	0.001800186
TMEM220	chr17-10033051-10035710	0.001800014
MYO9A	chr15-72796388-72797700	0.001799991
RNF144A	chr2-6909270-6910554	0.001799698
RNF144A	chr2-7866754-7868612	0.001799629
CPQ	chr8-96860513-96862323	0.001799565
EBF1	chr5-159119997-159121121	0.001799565
CHPT1	chr12-102060848-102063398	0.001799544
TMEM220	chr17-10156011-10157998	0.001799524
TMEM220	chr17-10017267-10022890	0.001799515
CPQ	chr8-96926547-96927687	0.001799325
DERA	chr12-14948045-14954989	0.001799244
TGS1	chr8-56557838-56560728	0.001799206
TSC22D1	chr13-44191352-44192271	0.001799166
TSC22D1	chr13-45310783-45312191	0.001799088
CPQ	chr8-96979011-96979984	0.001798984
TSC22D1	chr13-45119076-45121747	0.001798914
STX18-AS1	chr4-4268031-4270980	0.00179886
TMEM220	chr17-10013088-10014246	0.001798818
RNF144A	chr2-6913141-6914394	0.001798465
TMEM220	chr17-10059102-10061687	0.001798456
STX18-AS1	chr4-4540786-4542945	0.001798398
RNF144A	chr2-6876123-6879209	0.00179836
STX18-AS1	chr4-4247425-4249424	0.001798351
TMEM220	chr17-10729108-10730687	0.00179824
CPQ	chr8-96337549-96338759	0.001798053
FMN1	chr15-34365117-34368781	0.001797946
TSC22D1	chr13-44271972-44276535	0.001797708
CHPT1	chr12-101828476-101831647	0.001797582
MYO9A	chr15-72461470-72462846	0.001797433
CHPT1	chr12-102119146-102121327	0.00179729
CPQ	chr8-96988847-96990053	0.001797188
TMEM220	chr17-10118755-10121233	0.001797171
EBF1	chr5-159411879-159413092	0.001797058
EBF1	chr5-159986871-159987824	0.001796992
TSC22D1	chr13-44258476-44260377	0.001796803
DERA	chr12-16347276-16349614	0.001796791
CPQ	chr8-96234166-96236626	0.001796748
EBF1	chr5-159773019-159773979	0.001796658
RNF144A	chr2-6863856-6866834	0.001796478

MYO9A	chr15-72494159-72497038	0.001796429
MYO9A	chr15-72487883-72491179	0.001796068
EBF1	chr5-160008384-160010180	0.001795862
MYO9A	chr15-72472122-72476918	0.001795407
TMEM220	chr17-10171100-10173168	0.001795348
MYO9A	chr15-72781485-72787299	0.001795335
TMEM220	chr17-10062624-10064745	0.001795285
PPM1L	chr3-160129352-160130248	0.001795218
MYO9A	chr15-72477679-72483530	0.001795171
CHPT1	chr12-101669821-101672401	0.00179494
RNF144A	chr2-6916730-6919459	0.001794891
STX18-AS1	chr4-4574521-4576143	0.001794816
CPQ	chr8-96282646-96284202	0.001794603
CPQ	chr8-96260731-96264985	0.001794355
DERA	chr12-14884262-14885094	0.001794339
PPM1L	chr3-159988141-159989532	0.001794331
CHPT1	chr12-100573149-100574681	0.001794138
CHPT1	chr12-101696556-101699170	0.001794078
TMEM220	chr17-10696528-10700487	0.001794039
CPQ	chr8-97277326-97278400	0.001793975
CHPT1	chr12-101615562-101616807	0.001793642
PPM1L	chr3-160225020-160226917	0.001793642
TMEM220	chr17-10117078-10118185	0.001793628
CHPT1	chr12-101822904-101824570	0.001793573
RNF144A	chr2-7448846-7453802	0.001793193
RNF144A	chr2-7430060-7432913	0.001793191
TMEM220	chr17-10113073-10116563	0.001792951
RNF144A	chr2-7773024-7774356	0.001792905
STX18-AS1	chr4-4943596-4944553	0.001792864
TMEM220	chr17-10197436-10199437	0.001792842
EBF1	chr5-160118144-160120528	0.001792818
PPM1L	chr3-159928159-159930308	0.001792573
CHPT1	chr12-101743533-101744653	0.001792181
GPHN	chr14-67700438-67701150	0.001792125
GPHN	chr14-65411192-65414173	0.001791915
DERA	chr12-14802351-14804603	0.001791644
CHPT1	chr12-101773493-101774282	0.001791542
PPM1L	chr3-159762787-159765472	0.001791489
GPHN	chr14-66506903-66509695	0.001791459
RNF144A	chr2-7427232-7429492	0.001791379
RNF144A	chr2-7007713-7008819	0.001791345
CHPT1	chr12-101406922-101408830	0.001791237
CHPT1	chr12-100576373-100577313	0.001791154
GPHN	chr14-65926894-65929245	0.001791088
RNF144A	chr2-7467797-7468793	0.001790956
GPHN	chr14-65846246-65848279	0.00179084
DERA	chr12-14773379-14776934	0.001790632
AL359232.1	chr14-67700438-67701150	0.001790584
CPQ	chr8-97637588-97640766	0.001790567
GPHN	chr14-65849772-65851081	0.001790458

AL359232.1	chr14-65411192-65414173	0.001790375
FAM227B	chr15-48877547-48878897	0.001790109
RNF144A	chr2-7057705-7059981	0.001790104
GPHN	chr14-67239895-67242518	0.001790037
FAM227B	chr15-48809929-48812182	0.001790007
CHPT1	chr12-101279278-101280859	0.001789953
AL359232.1	chr14-66506903-66509695	0.001789918
PPM1L	chr3-160398427-160402339	0.001789826
GPHN	chr14-67694473-67696405	0.001789774
AL359232.1	chr14-65926894-65929245	0.001789547
EBF1	chr5-160268256-160269736	0.001789483
AL359232.1	chr14-65846246-65848279	0.001789301
DERA	chr12-14768761-14772476	0.001789107
GPHN	chr14-67673884-67675612	0.001788939
AL359232.1	chr14-65849772-65851081	0.001788919
GPHN	chr14-67618221-67621394	0.001788849
RNF144A	chr2-7725055-7726334	0.001788717
AL359232.1	chr14-67239895-67242518	0.001788497
RNF144A	chr2-7567192-7568460	0.001788491
AL359232.1	chr14-67694473-67696405	0.001788235
CPQ	chr8-97642994-97646936	0.001787957
GPHN	chr14-67599289-67601659	0.00178791
AL359232.1	chr14-67673884-67675612	0.001787401
AL359232.1	chr14-67618221-67621394	0.001787311
FAM227B	chr15-48975578-48976522	0.00178716
PPM1L	chr3-160801222-160802568	0.001787156
PPM1L	chr3-160980712-160981849	0.001787127
FAM227B	chr15-48794517-48795311	0.001786821
GPHN	chr14-67358614-67361153	0.001786674
AL359232.1	chr14-67599289-67601659	0.001786373
PPM1L	chr3-160448681-160450778	0.001785919
FAM227B	chr15-50064248-50067748	0.001785723
CPQ	chr8-97774778-97777125	0.001785703
GPHN	chr14-67532668-67533932	0.001785456
PPM1L	chr3-160754231-160757791	0.001785287
AL359232.1	chr14-67358614-67361153	0.001785138
FAM227B	chr15-49619931-49622022	0.00178495
PPM1L	chr3-160563993-160566731	0.001784256
AL359232.1	chr14-67532668-67533932	0.001783922
PPM1L	chr3-161034939-161036435	0.001783866
FAM227B	chr15-49045360-49047252	0.001783337
GPHN	chr14-67411579-67412589	0.00178309
FAM227B	chr15-50107463-50108572	0.001783044
GPHN	chr14-67512915-67517213	0.001782612
FAM227B	chr15-49169510-49171331	0.00178252
AL359232.1	chr14-67411579-67412589	0.001781557
FAM227B	chr15-49154763-49156615	0.001781454
GPHN	chr14-67487710-67489382	0.001781441
AL359232.1	chr14-67512915-67517213	0.001781079
AL359232.1	chr14-67487710-67489382	0.00177991

PPM1L	chr3-161220658-161223168	0.001778484
FAM227B	chr15-50112271-50114883	0.001777869
PPM1L	chr3-161310478-161311765	0.001773536
FAM227B	chr15-50685589-50687695	0.0017732
FAM227B	chr15-50247560-50248736	0.001773064
FAM227B	chr15-50764162-50766613	0.00177305
FAM227B	chr15-50423239-50425495	0.001771779
FAM227B	chr15-50350024-50357258	0.001771023
PPM1L	chr3-161371468-161373209	0.00177098
PPM1L	chr3-161394089-161395853	0.001770902
ESR1	chr6-150865164-150867189	0.001770679
ESR1	chr6-151040629-151041543	0.001769967
ESR1	chr6-151493338-151495048	0.001769366
ESR1	chr6-150631311-150634452	0.001769199
ESR1	chr6-151451390-151453469	0.001768956
PTPRK	chr6-129709359-129710662	0.001767979
PTPRK	chr6-129683436-129687939	0.001767726
ESR1	chr6-150622557-150623848	0.001767344
ESR1	chr6-151372124-151375285	0.001767247
ESR1	chr6-150598919-150601318	0.001767154
ESR1	chr6-151497739-151498928	0.001766862
ESR1	chr6-151412742-151414393	0.001766438
PTPRK	chr6-129671494-129673262	0.00176621
ESR1	chr6-152309587-152310902	0.001764727
ESR1	chr6-151379715-151382104	0.00176464
PTPRK	chr6-128258989-128260596	0.001764632
ESR1	chr6-151388259-151394227	0.00176431
PTPRK	chr6-127971333-127972754	0.001764185
PTPRK	chr6-129498735-129499976	0.001764111
PTPRK	chr6-128518628-128521238	0.001763507
ESR1	chr6-152300562-152302764	0.001763386
ESR1	chr6-151690155-151691839	0.00176311
PTPRK	chr6-129490033-129491882	0.001762968
ESR1	chr6-152182731-152186741	0.001761243
PTPRK	chr6-127950577-127952028	0.001760948
ESR1	chr6-152170496-152171714	0.001760806
RNF144B	chr6-18386898-18388710	0.001759038
FMNL2	chr2-152729842-152731394	0.001758222
STAM2	chr2-152729842-152731394	0.001758209
RNF144B	chr6-18329770-18331439	0.001757159
RNF144B	chr6-18400610-18401608	0.00175709
PTPRK	chr6-127934159-127935188	0.00175585
PTPRK	chr6-127346343-127348560	0.001754961
PTPRK	chr6-127457249-127458094	0.001754348
USP28	chr11-114134804-114137698	0.00175433
RNF144B	chr6-18262636-18266097	0.001753963
USP28	chr11-114154158-114156839	0.001753819
AC253572.2	chr1-119987043-119989225	0.001753739
FMNL2	chr2-152715880-152719408	0.00175321
STAM2	chr2-152715880-152719408	0.001753196

PTPRK	chr6-127341420-127344729	0.001753194
AC253572.2	chr1-121097029-121098186	0.00175288
PTPRK	chr6-127514416-127517077	0.001751948
AC253572.2	chr1-121184237-121185436	0.001751857
PTPRK	chr6-127918230-127919310	0.001751564
FMNL2	chr2-152466784-152469577	0.00175151
STAM2	chr2-152466784-152469577	0.001751494
ZBTB16	chr11-114134804-114137698	0.001751467
RNF144B	chr6-18260157-18261396	0.001751458
FMNL2	chr2-152431872-152433753	0.001751396
STAM2	chr2-152431872-152433753	0.001751382
AC253572.2	chr1-120068879-120069874	0.001751337
FMNL2	chr2-151260615-151262925	0.001751272
STAM2	chr2-151260615-151262925	0.001751257
USP28	chr11-114082185-114084125	0.001751
FMNL2	chr2-152030723-152032057	0.001750998
STAM2	chr2-152030723-152032057	0.001750984
USP28	chr11-114157510-114163266	0.001750972
ZBTB16	chr11-114154158-114156839	0.001750958
LIX1-AS1	chr5-95957625-95963190	0.001750937
FMNL2	chr2-152593913-152594972	0.001750768
FMNL2	chr2-152611478-152613162	0.001750768
STAM2	chr2-152593913-152594972	0.001750754
STAM2	chr2-152611478-152613162	0.001750753
RALB	chr2-120176421-120178325	0.001750741
FMNL2	chr2-152042702-152043557	0.001750686
STAM2	chr2-152042702-152043557	0.001750672
LIX1-AS1	chr5-96537134-96538159	0.001750539
AC253572.2	chr1-120941376-120943046	0.001750305
PTPRK	chr6-127900629-127901712	0.001750286
RALB	chr2-120109060-120110951	0.001750267
FMNL2	chr2-152420658-152422275	0.001750259
STAM2	chr2-152420658-152422275	0.001750245
RNF144B	chr6-18165741-18167090	0.001749616
FMNL2	chr2-152174684-152176915	0.001749596
STAM2	chr2-152174684-152176915	0.001749581
FMNL2	chr2-151826590-151829334	0.001749405
AC253572.2	chr1-120175677-120177294	0.001749396
STAM2	chr2-151826590-151829334	0.001749391
FMNL2	chr2-152334833-152336823	0.001749323
STAM2	chr2-152334833-152336823	0.001749309
RALB	chr2-120222341-120225940	0.001748747
LIX1-AS1	chr5-96656585-96657624	0.00174863
USP28	chr11-114163818-114167613	0.001748375
ZBTB16	chr11-114082185-114084125	0.00174814
ZBTB16	chr11-114157510-114163266	0.001748115
AC253572.2	chr1-121395774-121397291	0.001747881
PALM2-AKAP2	chr9-110603983-110604864	0.001747814
USP28	chr11-114171353-114173683	0.001747353
LIX1-AS1	chr5-96714058-96716718	0.001747178

LIX1-AS1	chr5-96660811-96665075	0.001747169
AC253572.2	chr1-120414772-120416078	0.001747109
USP28	chr11-114178903-114181100	0.001747093
RALB	chr2-120312729-120315258	0.001747083
LIX1-AS1	chr5-96701117-96704269	0.001747012
RNF144B	chr6-18152594-18156867	0.001746934
RALB	chr2-120229703-120233646	0.001746705
FMNL2	chr2-151636867-151639053	0.001746615
STAM2	chr2-151636867-151639053	0.0017466
PALM2-AKAP2	chr9-110254528-110258209	0.001746549
FMNL2	chr2-151286793-151290797	0.001746533
STAM2	chr2-151286793-151290797	0.001746517
RALB	chr2-120343255-120347209	0.001746493
RALB	chr2-120012433-120013817	0.001746476
RALB	chr2-120250895-120254430	0.001746355
AC253572.2	chr1-120913851-120914547	0.001745981
LIX1-AS1	chr5-96804664-96809335	0.001745972
USP28	chr11-114182705-114184074	0.0017457
ZBTB16	chr11-114163818-114167613	0.001745523
USP28	chr11-114072228-114076352	0.001744983
FMNL2	chr2-151409292-151410920	0.001744959
STAM2	chr2-151409292-151410920	0.001744944
ZBTB16	chr11-114171353-114173683	0.001744502
AC253572.2	chr1-120723496-120724701	0.001744363
ZBTB16	chr11-114178903-114181100	0.001744241
RIC8B	chr12-107317230-107322881	0.001744175
RIC8B	chr12-107327770-107330323	0.001744039
USP28	chr11-113313891-113315828	0.001743606
USP28	chr11-113188019-113189273	0.001743585
PALM2-AKAP2	chr9-110156459-110158309	0.001743507
AC253572.2	chr1-121458342-121459187	0.001743499
RIC8B	chr12-107091720-107094735	0.001742991
ZBTB16	chr11-114182705-114184074	0.00174285
LIX1-AS1	chr5-96815987-96817844	0.001742819
AC253572.2	chr1-120849406-120851607	0.001742779
RALB	chr2-121197103-121198402	0.001742623
RNF144B	chr6-18122073-18123208	0.001742423
USP28	chr11-114398923-114402696	0.00174238
AC253572.2	chr1-120843986-120845057	0.001742365
ZBTB16	chr11-114072228-114076352	0.001742131
WDFY4	chr10-48671056-48673240	0.001742085
RIC8B	chr12-107027269-107028189	0.001742032
PALM2-AKAP2	chr9-110047594-110050261	0.001741653
USP28	chr11-113772958-113774389	0.001741628
WDFY4	chr10-48667729-48668759	0.001741473
AC253572.2	chr1-121518559-121520360	0.001741438
RIC8B	chr12-106986157-106987825	0.001741385
PALM2-AKAP2	chr9-110064441-110067098	0.001741227
RIC8B	chr12-107368392-107371052	0.001741224
AC253572.2	chr1-121511055-121512455	0.001741065

AC253572.2	chr1-121515914-121517375	0.001740946
RALB	chr2-119928977-119930258	0.001740928
PALM2-AKAP2	chr9-110148128-110149339	0.001740832
ZBTB16	chr11-113313891-113315828	0.001740757
ZBTB16	chr11-113188019-113189273	0.001740736
AC253572.2	chr1-121551214-121552022	0.001740654
PALM2-AKAP2	chr9-110142949-110144470	0.001740205
WDFY4	chr10-48683722-48686105	0.001739826
AC092944.1	chr3-156663765-156665790	0.001739728
RIC8B	chr12-106954431-106957749	0.001739726
ZBTB2	chr6-150865164-150867189	0.001739624
ZBTB16	chr11-114398923-114402696	0.001739535
USP28	chr11-114058408-114062594	0.001739442
AC092944.1	chr3-156605580-156607342	0.001739435
TMEM267	chr5-43601825-43604964	0.001739433
PALM2-AKAP2	chr9-110021755-110022575	0.001739406
LIX1-AS1	chr5-96875509-96877908	0.001739013
ZBTB2	chr6-151040629-151041543	0.001738923
ZBTB16	chr11-113772958-113774389	0.001738782
USP28	chr11-113874514-113876825	0.001738612
WDFY4	chr10-48655040-48657234	0.001738535
TMEM267	chr5-43191557-43194307	0.00173839
USP28	chr11-114438562-114440428	0.001738374
ZBTB2	chr6-151493338-151495048	0.00173833
TMEM267	chr5-43312175-43314526	0.001738279
RNF144B	chr6-17700693-17707667	0.001738213
ZBTB2	chr6-150631311-150634452	0.001738169
ZBTB2	chr6-151451390-151453469	0.001737927
LIX1-AS1	chr5-96932593-96937535	0.001737849
TMEM267	chr5-43555781-43558090	0.001737793
LIX1-AS1	chr5-96895919-96897482	0.001737573
AC092944.1	chr3-156673441-156678584	0.001737273
USP28	chr11-114035784-114036702	0.001737192
RNF144B	chr6-17883989-17885511	0.001737179
RNF144B	chr6-17993005-17994159	0.001737011
RNF144B	chr6-17599585-17602268	0.001736936
TMEM267	chr5-43396237-43397592	0.00173662
ZBTB16	chr11-114058408-114062594	0.001736598
LIX1-AS1	chr5-96879724-96882246	0.001736566
LIX1-AS1	chr5-96885128-96886772	0.001736444
ZBTB2	chr6-150622557-150623848	0.001736346
RALB	chr2-119758828-119761228	0.001736322
ZBTB2	chr6-151372124-151375285	0.00173625
AC092944.1	chr3-156553993-156555933	0.001736233
ZBTB2	chr6-150598919-150601318	0.00173616
WDFY4	chr10-48799268-48801620	0.001736116
LIX1-AS1	chr5-96957184-96959060	0.001736064
TMEM267	chr5-43120265-43122814	0.00173606
RALB	chr2-121286831-121287924	0.001736004
TMEM267	chr5-43514223-43515990	0.001735983

RIC8B	chr12-107371880-107374827	0.001735939
ZBTB2	chr6-151497739-151498928	0.001735871
ZBTB16	chr11-113874514-113876825	0.001735769
RIC8B	chr12-106773460-106775474	0.001735748
ZBTB16	chr11-114438562-114440428	0.001735535
ZBTB2	chr6-151412742-151414393	0.001735453
TMEM267	chr5-43483037-43485870	0.001735437
WDFY4	chr10-48633847-48635287	0.001735251
RNF144B	chr6-17591064-17592004	0.001734781
RALB	chr2-119355373-119356540	0.001734729
TBC1D19	chr4-26877378-26880429	0.001734665
PALM2-AKAP2	chr9-109973917-109975110	0.001734543
RNF144B	chr6-17907219-17909388	0.00173441
RALB	chr2-119365561-119368540	0.001734367
ZBTB16	chr11-114035784-114036702	0.001734352
RALB	chr2-119677829-119679812	0.001734303
RALB	chr2-119338535-119339863	0.001734254
AC092944.1	chr3-156679515-156681532	0.001734011
ZBTB2	chr6-152309587-152310902	0.001733775
TBC1D19	chr4-26872875-26874632	0.001733762
RNF144B	chr6-17580038-17581574	0.001733722
ZBTB2	chr6-151379715-151382104	0.001733688
RALB	chr2-119282215-119284528	0.001733605
ZBTB2	chr6-151388259-151394227	0.001733363
WDFY4	chr10-49176920-49178257	0.001733248
LIX1-AS1	chr5-96960648-96963650	0.001733079
WDFY4	chr10-48606618-48608530	0.001733067
IL15	chr4-141331746-141334153	0.00173305
RNF144B	chr6-17986605-17988785	0.001733025
TMEM267	chr5-43015290-43021643	0.001732788
WDFY4	chr10-49189840-49191653	0.001732765
TBC1D19	chr4-26882544-26887732	0.001732485
ZBTB2	chr6-152300562-152302764	0.001732456
WDFY4	chr10-49187986-49188922	0.001732419
TMEM267	chr5-43109316-43111439	0.001732408
RNF144B	chr6-17932006-17932980	0.001732266
LIX1-AS1	chr5-97182144-97184536	0.001732193
ZBTB2	chr6-151690155-151691839	0.001732186
IL15	chr4-141328684-141330991	0.001732182
WDFY4	chr10-49282713-49285977	0.001732134
AC092944.1	chr3-156524290-156525251	0.001732081
TMEM267	chr5-43006912-43011400	0.001732079
AC092944.1	chr3-156815385-156818224	0.001731836
WDFY4	chr10-48576782-48577813	0.001731794
TMEM267	chr5-43036356-43045777	0.001731281
LIX1-AS1	chr5-97008817-97010088	0.00173119
IL15	chr4-141347646-141349229	0.001731051
AC092944.1	chr3-156824659-156827480	0.001730925
PALM2-AKAP2	chr9-109110054-109111199	0.001730535
ZBTB2	chr6-152182731-152186741	0.00173035

TBC1D19	chr4-26856716-26862811	0.001730278
RIC8B	chr12-107684619-107686602	0.001730224
WDFY4	chr10-48305881-48308047	0.001730157
ZBTB2	chr6-152170496-152171714	0.001729921
PALM2-AKAP2	chr9-109117725-109120693	0.001729749
TMEM267	chr5-43104171-43106360	0.001729722
RIC8B	chr12-106759845-106760739	0.001729675
AC092944.1	chr3-156483062-156484992	0.00172956
TMEM267	chr5-43063602-43069165	0.00172953
PALM2-AKAP2	chr9-109971957-109972872	0.001729458
WDFY4	chr10-49309515-49312510	0.001729045
TMEM267	chr5-42989132-42996745	0.001728944
TBC1D19	chr4-26992306-26993689	0.001728942
LRMP	chr12-26124505-26126973	0.001728583
PALM2-AKAP2	chr9-109012092-109014804	0.001728494
IL15	chr4-141311183-141312756	0.001728286
IL15	chr4-141635796-141638004	0.001727968
PALM2-AKAP2	chr9-109320082-109321420	0.001727561
WDFY4	chr10-47552667-47554131	0.001727487
TBC1D19	chr4-26318137-26322886	0.00172727
CD96	chr3-111541589-111549459	0.001726941
PALM2-AKAP2	chr9-109966404-109970056	0.001726869
TBC1D19	chr4-26826369-26827690	0.001726637
CD96	chr3-111609539-111611188	0.001726274
TBC1D19	chr4-26287614-26289087	0.001726115
RIC8B	chr12-107759355-107762252	0.001726107
TBC1D19	chr4-26583233-26584897	0.001726101
ATP2C1	chr3-131502057-131503767	0.001725635
ATP2C1	chr3-131386160-131387496	0.001725548
IL15	chr4-142354759-142357246	0.001725517
TBC1D19	chr4-26821665-26824006	0.001725198
IARS2	chr1-221188254-221189022	0.001724942
PALM2-AKAP2	chr9-108933084-108935287	0.001724921
TMEM267	chr5-42948600-42954736	0.001724772
CD96	chr3-111538708-111540273	0.001724548
TBC1D19	chr4-25375096-25378207	0.001724163
IL15	chr4-142404226-142407894	0.001724024
WDFY4	chr10-49362339-49363344	0.00172387
RIC8B	chr12-106747268-106748712	0.001723438
LRMP	chr12-26121549-26123803	0.001723294
IL15	chr4-141220309-141221819	0.001723224
TBC1D19	chr4-25859016-25864828	0.001723016
PALM2-AKAP2	chr9-108469403-108470181	0.001722717
IL15	chr4-142416500-142417469	0.001722604
IARS2	chr1-221166429-221168019	0.001722602
AC092944.1	chr3-157128020-157131872	0.00172252
SPRED1	chr15-39334671-39335739	0.00172249
C9orf85	chr9-71781152-71784329	0.00172239
CD96	chr3-111615730-111617448	0.001722291
TBC1D19	chr4-26272627-26274176	0.001721994

CD96	chr3-111069996-111072928	0.001721697
SPRED1	chr15-39326231-39327968	0.001721643
CEMIP2	chr9-71781152-71784329	0.001721549
C9orf85	chr9-71761935-71770246	0.001721451
ATP2C1	chr3-131380743-131382667	0.001721205
TMEM267	chr5-42908081-42909648	0.00172116
PCNX2	chr1-232628911-232631268	0.001720714
CEMIP2	chr9-71761935-71770246	0.001720611
IL15	chr4-142565505-142568490	0.001720573
C9orf85	chr9-71794620-71797336	0.001720502
PCNX2	chr1-232620179-232621509	0.001720447
SPRED1	chr15-39375803-39377103	0.001720264
IARS2	chr1-220876295-220883526	0.001719761
CEMIP2	chr9-71794620-71797336	0.001719662
SPRED1	chr15-39312926-39313801	0.001719569
CD96	chr3-112071762-112073124	0.001719512
IL15	chr4-140807663-140808626	0.001719473
IARS2	chr1-221097402-221099922	0.001719391
SPRED1	chr15-38129451-38131591	0.00171929
TBC1D19	chr4-25912823-25915129	0.001719218
CCDC170	chr6-150865164-150867189	0.001719185
IL15	chr4-140742300-140743897	0.001719145
RIC8B	chr12-106357180-106359114	0.001719138
IARS2	chr1-220999837-221001694	0.001718846
SPRED1	chr15-38084168-38086638	0.001718794
IL15	chr4-140755504-140757588	0.001718793
CD96	chr3-112085690-112087327	0.001718759
IL15	chr4-142703638-142704591	0.001718578
PCNX2	chr1-232589742-232591271	0.001718571
CCDC170	chr6-151040629-151041543	0.001718493
AC092944.1	chr3-157136856-157138238	0.001718469
WDFY4	chr10-49537951-49540170	0.001718441
IL15	chr4-140789450-140791505	0.00171827
IARS2	chr1-219088534-219091323	0.001718238
IL15	chr4-140718077-140721633	0.001718185
CCDC170	chr6-151493338-151495048	0.001717908
SPRED1	chr15-38690078-38691194	0.001717894
C9orf85	chr9-71909649-71912471	0.001717886
AC092944.1	chr3-157436399-157438415	0.001717854
LRMP	chr12-26113644-26115165	0.001717827
CCDC170	chr6-150631311-150634452	0.001717748
CD96	chr3-111978353-111980081	0.001717702
IARS2	chr1-220786117-220787560	0.001717634
PCNX2	chr1-232804830-232806476	0.001717627
CCDC170	chr6-151451390-151453469	0.00171751
RIC8B	chr12-106300717-106307143	0.001717433
CD96	chr3-111666946-111669513	0.001717376
C9orf85	chr9-71746636-71748832	0.001717282
C9orf85	chr9-72585527-72587408	0.001717263
AC092944.1	chr3-157173442-157176676	0.001717208

RIC8B	chr12-106245150-106250402	0.001717182
IARS2	chr1-221045795-221048005	0.001717155
IARS2	chr1-221069030-221070385	0.001717124
CEMIP2	chr9-71909649-71912471	0.001717049
TBC1D19	chr4-26217695-26219395	0.001716973
HOPX	chr4-56465847-56468627	0.001716854
C9orf85	chr9-72925310-72926323	0.001716723
AC092944.1	chr3-157144409-157164230	0.001716699
SPRED1	chr15-38682890-38686740	0.001716692
RIC8B	chr12-106226752-106228310	0.001716632
NNT-AS1	chr5-43601825-43604964	0.00171657
IL15	chr4-140523215-140525007	0.001716499
CEMIP2	chr9-71746636-71748832	0.001716444
CEMIP2	chr9-72585527-72587408	0.001716425
C9orf85	chr9-72292088-72293504	0.001716307
AC092944.1	chr3-158097356-158098931	0.001716096
CCDC170	chr6-150622557-150623848	0.001715947
CEMIP2	chr9-72925310-72926323	0.001715886
SPRED1	chr15-38251048-38254966	0.001715882
CCDC170	chr6-151372124-151375285	0.001715852
HOPX	chr4-56504954-56506897	0.001715831
CD96	chr3-112126338-112130888	0.001715814
C9orf85	chr9-72294240-72295375	0.00171581
CCDC170	chr6-150598919-150601318	0.001715763
HOPX	chr4-56434405-56438050	0.001715726
NNT-AS1	chr5-43191557-43194307	0.00171554
CCDC170	chr6-151497739-151498928	0.001715477
CEMIP2	chr9-72292088-72293504	0.001715471
NNT-AS1	chr5-43312175-43314526	0.001715431
IARS2	chr1-219173290-219175266	0.001715418
TBC1D19	chr4-26074176-26076917	0.001715284
C9orf85	chr9-72304222-72307260	0.001715151
CD96	chr3-111858920-111861022	0.001715144
HOPX	chr4-56757147-56759641	0.001715111
CCDC170	chr6-151412742-151414393	0.001715065
C9orf85	chr9-72526814-72528257	0.001714992
CEMIP2	chr9-72294240-72295375	0.001714975
SPRED1	chr15-38670051-38673484	0.001714965
NNT-AS1	chr5-43555781-43558090	0.001714953
HOPX	chr4-56654674-56657011	0.001714847
ATP2C1	chr3-131360987-131362529	0.001714651
SPRED1	chr15-39387897-39388975	0.001714633
LRMP	chr12-25958083-25959938	0.001714583
CD96	chr3-111672238-111677630	0.001714578
HOPX	chr4-56529997-56531607	0.001714382
CEMIP2	chr9-72304222-72307260	0.001714317
TBC1D19	chr4-26197147-26198024	0.00171418
CEMIP2	chr9-72526814-72528257	0.001714154
SPRED1	chr15-38072054-38073460	0.001714128
HOPX	chr4-56544280-56545263	0.001714071

LRMP	chr12-25859728-25860614	0.001713845
NNT-AS1	chr5-43396237-43397592	0.001713794
LRMP	chr12-25589794-25591319	0.001713701
IARS2	chr1-219459989-219462468	0.00171349
C9orf85	chr9-72351466-72357300	0.001713429
CCDC170	chr6-152309587-152310902	0.001713408
CCDC170	chr6-151379715-151382104	0.00171332
HOPX	chr4-56799629-56800610	0.001713282
NNT-AS1	chr5-43120265-43122814	0.001713241
NNT-AS1	chr5-43514223-43515990	0.001713166
CCDC170	chr6-151388259-151394227	0.001712999
CD96	chr3-112132239-112135282	0.001712642
NNT-AS1	chr5-43483037-43485870	0.001712628
CEMIP2	chr9-72351466-72357300	0.001712596
IARS2	chr1-220732574-220733700	0.001712325
HOPX	chr4-56386687-56388724	0.001712308
SPRED1	chr15-38650378-38653900	0.001712199
PCNX2	chr1-232923560-232924677	0.001712197
PCNX2	chr1-234486742-234489041	0.001712149
CCDC170	chr6-152300562-152302764	0.001712105
C9orf85	chr9-71685096-71686444	0.00171202
C9orf85	chr9-72464648-72465411	0.001711937
SLC35F1	chr6-119076084-119079630	0.001711934
LRMP	chr12-25381893-25388706	0.001711845
CCDC170	chr6-151690155-151691839	0.001711836
SPRED1	chr15-38453667-38455453	0.001711368
C9orf85	chr9-72358377-72366566	0.001711292
AC092944.1	chr3-158102750-158107613	0.001711286
CEMIP2	chr9-71685096-71686444	0.001711184
CEMIP2	chr9-72464648-72465411	0.001711103
LRMP	chr12-24661431-24662278	0.001710929
CD96	chr3-112303402-112304925	0.001710811
IARS2	chr1-220044472-220047771	0.001710703
FCGR1B	chr1-119987043-119989225	0.001710536
SNTB1	chr8-120730818-120733426	0.001710475
CEMIP2	chr9-72358377-72366566	0.001710459
C9orf85	chr9-72443173-72444604	0.001710421
CD96	chr3-112332855-112335695	0.001710343
CD96	chr3-112463074-112464994	0.001710069
CCDC170	chr6-152182731-152186741	0.001710023
NNT-AS1	chr5-43015290-43021643	0.001710014
FCGR1B	chr1-121097029-121098186	0.001709698
PCNX2	chr1-234477727-234479943	0.001709696
SLC35F1	chr6-118933925-118936184	0.001709654
NNT-AS1	chr5-43109316-43111439	0.001709638
CCDC170	chr6-152170496-152171714	0.001709599
CEMIP2	chr9-72443173-72444604	0.001709587
SLC35F1	chr6-118767885-118769684	0.001709575
SPRED1	chr15-38610478-38611834	0.001709476
NNT-AS1	chr5-43006912-43011400	0.001709315

HOPX	chr4-56821133-56822304	0.001709268
CD96	chr3-112472742-112474151	0.001709221
SNTB1	chr8-120706155-120707315	0.001708977
ATP2C1	chr3-131025612-131027816	0.001708968
SLC35F1	chr6-118796214-118797136	0.001708962
C9orf85	chr9-71638389-71639308	0.001708856
SPRED1	chr15-38562107-38565687	0.001708822
FCGR1B	chr1-121184237-121185436	0.0017087
NNT-AS1	chr5-43036356-43045777	0.001708527
HOPX	chr4-55946711-55950287	0.00170838
FCGR1B	chr1-120068879-120069874	0.001708192
CEMIP2	chr9-71638389-71639308	0.001708023
SNTB1	chr8-120747671-120750315	0.001708014
LRMP	chr12-25376363-25378626	0.001707706
SPRED1	chr15-38068785-38070230	0.001707701
SPRED1	chr15-39426489-39428174	0.001707439
SLC35F1	chr6-118745451-118747159	0.001707256
FCGR1B	chr1-120941376-120943046	0.001707186
PCNX2	chr1-232949922-232951695	0.001707168
NNT-AS1	chr5-43104171-43106360	0.00170699
SLC35F1	chr6-118890873-118897131	0.001706975
SLC35F1	chr6-118800198-118801279	0.001706926
NNT-AS1	chr5-43063602-43069165	0.001706799
IARS2	chr1-220088553-220090800	0.001706675
HOPX	chr4-55544766-55547742	0.001706458
FCGR1B	chr1-120175677-120177294	0.001706299
IARS2	chr1-220689931-220690914	0.00170628
KIAA0825	chr5-95807745-95811026	0.001706252
NNT-AS1	chr5-42989132-42996745	0.00170622
LRMP	chr12-24900307-24904349	0.001706186
HOPX	chr4-55853020-55854731	0.001706136
ATP2C1	chr3-130892676-130895347	0.001705953
SLC35F1	chr6-118808472-118811152	0.001705822
PCNX2	chr1-234408445-234409794	0.001705658
AC092944.1	chr3-158109474-158110957	0.001705543
KIAA0825	chr5-95780592-95781687	0.001705223
FCGR1B	chr1-121395774-121397291	0.001704822
ATP2C1	chr3-130849963-130850951	0.001704752
PCNX2	chr1-233326765-233329082	0.001704716
LRMP	chr12-25050989-25053345	0.001704703
PCNX2	chr1-233112461-233115425	0.001704675
HOPX	chr4-56906387-56910011	0.001704621
PCNX2	chr1-233294816-233296040	0.001704548
LRMP	chr12-24947873-24949623	0.001704533
LRMP	chr12-25194179-25198394	0.001704427
SNTB1	chr8-120701935-120702782	0.001704401
FCGR1B	chr1-120414772-120416078	0.00170407
SNTB1	chr8-120774824-120775866	0.001703461
PCNX2	chr1-234213699-234214908	0.001703249
IARS2	chr1-220093525-220095066	0.001703161

LRMP	chr12-25333459-25334645	0.001703006
KIAA0825	chr5-95816783-95826314	0.001702976
FCGR1B	chr1-120913851-120914547	0.00170297
ATP2C1	chr3-130745728-130748692	0.001702956
IARS2	chr1-220271416-220273260	0.001702688
LRMP	chr12-25240904-25241987	0.001702639
SLC35F1	chr6-118718861-118720767	0.001702566
LYN	chr8-55917258-55920151	0.001702265
NNT-AS1	chr5-42948600-42954736	0.001702103
LYN	chr8-55887981-55891956	0.001701637
FCGR1B	chr1-120723496-120724701	0.001701392
KIAA0825	chr5-95759606-95762192	0.001701352
SNTB1	chr8-119855115-119856867	0.001701306
HOPX	chr4-56957924-56959055	0.001701116
LYN	chr8-55924111-55925731	0.001701073
PCNX2	chr1-234372285-234374582	0.001700923
ZSWIM6	chr5-62403012-62404840	0.001700711
FCGR1B	chr1-121458342-121459187	0.001700548
SNTB1	chr8-119873176-119875191	0.001700526
LRMP	chr12-25249188-25252185	0.001700496
SNTB1	chr8-120868500-120869292	0.001700397
SNTB1	chr8-119832075-119833836	0.001700289
LYN	chr8-55878674-55886699	0.001700255
LRMP	chr12-25322245-25324346	0.001700237
PCNX2	chr1-234325860-234327235	0.001700213
SNTB1	chr8-119614274-119616021	0.001700135
FCGR1B	chr1-120849406-120851607	0.001699847
SNTB1	chr8-120808383-120812937	0.001699818
SNTB1	chr8-120444106-120445843	0.00169972
SNTB1	chr8-120861099-120862500	0.001699659
PPP2CB	chr8-30141162-30142596	0.001699529
FCGR1B	chr1-120843986-120845057	0.001699443
PPP2CB	chr8-30154569-30158928	0.001699221
HOPX	chr4-56976023-56980163	0.001699215
SNTB1	chr8-119638416-119639956	0.001699143
ZSWIM6	chr5-62305096-62308639	0.001698919
ATP2C1	chr3-130178845-130180265	0.001698852
LYN	chr8-55863605-55866433	0.001698822
SNTB1	chr8-120857001-120858110	0.001698756
NNT-AS1	chr5-42908081-42909648	0.001698539
FCGR1B	chr1-121518559-121520360	0.001698537
SNTB1	chr8-119955699-119956802	0.001698532
ZSWIM6	chr5-62411687-62413573	0.001698384
FCGR1B	chr1-121511055-121512455	0.001698173
RTN1	chr14-60247260-60250830	0.001698128
FCGR1B	chr1-121515914-121517375	0.001698058
HOPX	chr4-57109421-57111517	0.001698019
LYN	chr8-55929235-55931181	0.001697988
PPP2CB	chr8-30131799-30137748	0.001697865
FCGR1B	chr1-121551214-121552022	0.001697773

PCNX2	chr1-234356401-234357420	0.001697719
PCNX2	chr1-234329920-234332770	0.00169764
SNTB1	chr8-119981919-119983258	0.001697619
SLC35F1	chr6-118708118-118711050	0.001697552
KIAA0825	chr5-95730676-95732517	0.001697505
RTN1	chr14-60327297-60328382	0.001696895
LYN	chr8-55843307-55845942	0.001696757
RTN1	chr14-60183228-60184747	0.001696565
RTN1	chr14-60980237-60982339	0.001695836
PPP2CB	chr8-30655667-30659145	0.001695802
PPP2CB	chr8-30111754-30116951	0.001695561
KIAA0825	chr5-95645792-95648148	0.001695543
ATP2C1	chr3-129690286-129691149	0.001695323
KIAA0825	chr5-95631726-95632862	0.001695038
ZSWIM6	chr5-62299149-62300322	0.001694578
LYN	chr8-55938371-55941027	0.001694498
LYN	chr8-55988863-55992423	0.001694496
LYN	chr8-56005010-56007385	0.001694486
KIAA0825	chr5-95619500-95622982	0.001694215
RTN1	chr14-60753173-60754580	0.001694103
RTN1	chr14-60330852-60332246	0.001693949
SLC35F1	chr6-118704316-118707375	0.001693941
RTN1	chr14-60164057-60166695	0.001693161
ATP2C1	chr3-130110673-130113120	0.001693107
LYN	chr8-55975069-55976652	0.001693082
LYN	chr8-55772149-55774233	0.001692969
PPP2CB	chr8-30103000-30106053	0.001692905
LYN	chr8-55971141-55974128	0.001692632
ATP2C1	chr3-129822172-129823214	0.00169243
RTN1	chr14-60734454-60735560	0.001691944
KIAA0825	chr5-95554067-95556107	0.001691872
SLC35F1	chr6-118650170-118652466	0.001691837
ATP2C1	chr3-129891738-129894470	0.001691787
LYN	chr8-56073243-56075461	0.001691773
RTN1	chr14-60721278-60724539	0.001691718
ATP2C1	chr3-129910996-129911761	0.001691502
ZSWIM6	chr5-62291542-62292482	0.001690196
SLC35F1	chr6-118558366-118559580	0.001690114
ATP2C1	chr3-129936530-129937358	0.001689972
PPP2CB	chr8-30721888-30724685	0.001689864
RTN1	chr14-60151324-60152747	0.001689818
PPP2CB	chr8-30067057-30102417	0.001689267
KIAA0825	chr5-95283693-95285453	0.001688669
ATP2C1	chr3-130093577-130095028	0.001688197
LINC02432	chr4-141331746-141334153	0.001688053
SLC35F1	chr6-117674693-117678752	0.001687855
ATP2C1	chr3-129974319-129975989	0.00168755
LYN	chr8-56209284-56213092	0.001687478
RTN1	chr14-60090982-60094814	0.001687375
SLC35F1	chr6-117481297-117484097	0.001687373

KIAA0825	chr5-94617787-94619538	0.00168731
ZSWIM6	chr5-62255978-62257203	0.001687268
LINC02432	chr4-141328684-141330991	0.001687207
KIAA0825	chr5-94432362-94434176	0.001687072
KIAA0825	chr5-95261285-95262309	0.001686454
ATP2C1	chr3-130002479-130003461	0.001686322
KIAA0825	chr5-95081164-95082488	0.001686311
LINC02432	chr4-141347646-141349229	0.001686106
ZSWIM6	chr5-60520446-60522851	0.001685692
SLC35F1	chr6-117601332-117603593	0.001685629
LYN	chr8-56992571-56994590	0.001685442
ZSWIM6	chr5-62223348-62224585	0.001685413
SLC35F1	chr6-117547384-117550015	0.001685103
RTN1	chr14-59964122-59966155	0.001685001
LYN	chr8-56557838-56560728	0.001684564
PPP2CB	chr8-30056178-30062519	0.001684318
KIF13A	chr6-18386898-18388710	0.001683981
PPP2CB	chr8-30725479-30728586	0.001683865
KIAA0825	chr5-94110725-94112475	0.001683754
ZSWIM6	chr5-60538366-60540042	0.001683669
PPP2CB	chr8-31032169-31034808	0.001683419
LINC02432	chr4-141311183-141312756	0.001683412
ZSWIM6	chr5-61407728-61409863	0.001683333
ZSWIM6	chr5-60540820-60542481	0.001683312
LINC02432	chr4-141635796-141638004	0.001683103
ZSWIM6	chr5-60699219-60701783	0.001682623
KIF13A	chr6-18329770-18331439	0.001682183
KIF13A	chr6-18400610-18401608	0.001682116
RTN1	chr14-59576136-59577283	0.001681011
PPP2CB	chr8-30811779-30813375	0.001680789
LINC02432	chr4-142354759-142357246	0.001680716
ZSWIM6	chr5-60943398-60946386	0.001680583
PPP2CB	chr8-30743299-30744993	0.001680487
ZSWIM6	chr5-61329628-61337466	0.001680473
LINC02432	chr4-142404226-142407894	0.001679261
KIF13A	chr6-18262636-18266097	0.001679123
PPP2CB	chr8-30050040-30051959	0.001679072
LINC02432	chr4-141220309-141221819	0.00167848
ZSWIM6	chr5-61161544-61163610	0.001678043
LINC02432	chr4-142416500-142417469	0.001677878
ZSWIM6	chr5-61319061-61321362	0.00167778
KIAA0825	chr5-93620394-93623304	0.001677731
PPP2CB	chr8-29737134-29738269	0.001677255
ZSWIM6	chr5-61275209-61277922	0.00167676
KIF13A	chr6-18260157-18261396	0.001676725
PPP2CB	chr8-29806236-29806957	0.001675511
PPP2CB	chr8-29773315-29775473	0.001675068
KIF13A	chr6-18165741-18167090	0.00167496
RTN1	chr14-59482224-59485721	0.001674897
LINC02432	chr4-140807663-140808626	0.001674826

LINC02432	chr4-140742300-140743897	0.001674507
LINC02432	chr4-140155768-140157290	0.001674258
LINC02432	chr4-140755504-140757588	0.001674163
LINC02432	chr4-140789450-140791505	0.001673653
LINC02432	chr4-140718077-140721633	0.001673572
ZEB2	chr2-144606498-144608598	0.001672706
KIF13A	chr6-18152594-18156867	0.001672394
LINC02432	chr4-140252156-140253614	0.001672172
ZEB2	chr2-144507361-144525092	0.001671933
LINC02432	chr4-140523215-140525007	0.00167193
KIAA0825	chr5-93583842-93585789	0.001671447
LINC02432	chr4-140372539-140374535	0.001671167
ZEB2	chr2-144659326-144661724	0.00167095
ZEB2	chr2-144458547-144460632	0.001669751
ZEB2	chr2-145692823-145693644	0.001668821
RTN1	chr14-59463986-59466163	0.001668153
KIF13A	chr6-18122073-18123208	0.001668075
KIAA0825	chr5-93578321-93582136	0.001667565
ZEB2	chr2-144456157-144457278	0.001667282
ZEB2	chr2-144671432-144672488	0.001667028
ZEB2	chr2-144703561-144704735	0.001666267
CST3	chr20-23161193-23163162	0.001665896
CST3	chr20-23225049-23226072	0.001665767
CST3	chr20-23364639-23367021	0.001664859
ZEB2	chr2-144413766-144416097	0.001664691
CST3	chr20-23419660-23422145	0.001664371
CST3	chr20-23349252-23354353	0.001664188
KIF13A	chr6-17700693-17707667	0.001664043
CST3	chr20-23360750-23363886	0.001663732
CST3	chr20-23155087-23156626	0.001663507
RTN1	chr14-59187499-59190788	0.001663337
CST3	chr20-23354880-23359602	0.001663173
ZEB2	chr2-144682189-144684448	0.001663055
KIF13A	chr6-17883989-17885511	0.001663054
ZEB2	chr2-144700964-144702519	0.001662977
KIF13A	chr6-17993005-17994159	0.001662894
KIF13A	chr6-17599585-17602268	0.001662821
RTN1	chr14-58747023-58749718	0.00166216
ZEB2	chr2-144686457-144687557	0.001661419
ZEB2	chr2-144379732-144383990	0.001661249
CST3	chr20-23654704-23656790	0.001661063
KIF13A	chr6-17591064-17592004	0.001660758
PRLR	chr5-36239654-36243314	0.001660649
KIF13A	chr6-17907219-17909388	0.001660403
KIF13A	chr6-17015366-17016587	0.001660322
PRLR	chr5-36150714-36153072	0.001659818
PRLR	chr5-35924586-35926873	0.001659804
KIF13A	chr6-17580038-17581574	0.001659743
CST3	chr20-23147242-23148761	0.001659512
PRLR	chr5-35850992-35860227	0.00165932

TXK	chr4-47173733-47174517	0.001659317
KIF13A	chr6-17986605-17988785	0.001659078
KIF13A	chr6-17932006-17932980	0.001658351
PRLR	chr5-35617199-35618681	0.001658075
PRLR	chr5-35482861-35484647	0.001657846
ZEB2	chr2-144331053-144333291	0.001656684
PRLR	chr5-35830179-35831560	0.001656604
PRLR	chr5-34838461-34839895	0.001656275
CST3	chr20-24026855-24028092	0.001656075
CST3	chr20-23142022-23143527	0.001656019
PRLR	chr5-35319210-35320366	0.001655952
PRLR	chr5-34498553-34499726	0.00165585
PRLR	chr5-34914581-34916506	0.001655284
PRLR	chr5-35672810-35674482	0.001654954
TXK	chr4-47462296-47464332	0.001654898
PRLR	chr5-34928262-34930913	0.00165479
CST3	chr20-23135487-23137257	0.001654405
CST3	chr20-23124580-23127247	0.001654228
SMIM26	chr20-17609386-17618285	0.001653806
CST3	chr20-23115578-23117146	0.001653769
SMIM26	chr20-17679797-17683356	0.001653475
ZEB2	chr2-144323989-144325770	0.001652061
CST3	chr20-23085099-23088329	0.001651753
PRLR	chr5-35821647-35824310	0.001651342
DISC1	chr1-231039001-231042019	0.001650951
SMIM26	chr20-17568086-17571777	0.001650684
DISC1	chr1-230976744-230980426	0.001650093
TXK	chr4-47483751-47486533	0.001650019
DISC1	chr1-231211097-231212009	0.001649661
ZEB2	chr2-144183459-144184399	0.001649338
PRLR	chr5-35780042-35781579	0.00164926
ZEB2	chr2-144237655-144238696	0.001649227
CST3	chr20-23047720-23051657	0.001648623
SMIM26	chr20-17938703-17939855	0.001648555
DISC1	chr1-231240233-231243195	0.001647807
EPSTI1	chr13-42047387-42049823	0.001647146
DISC1	chr1-230972364-230973757	0.001647107
DISC1	chr1-231419856-231423107	0.001647021
SMIM26	chr20-17530442-17531825	0.001646963
DISC1	chr1-231335013-231339668	0.001646938
EPSTI1	chr13-42039217-42042177	0.00164649
DISC1	chr1-231527593-231530795	0.001646421
CST3	chr20-23025396-23026083	0.001646226
PRLR	chr5-35815494-35817703	0.001645602
SMIM26	chr20-17503581-17507647	0.001644929
PRLR	chr5-35796634-35797876	0.001644071
EPSTI1	chr13-42270788-42273492	0.001644055
TXK	chr4-47509966-47511026	0.001643951
DISC1	chr1-230867619-230869355	0.001643759
DISC1	chr1-231603305-231604429	0.001643468

PRLR	chr5-35810250-35812003	0.001642542
EPSTI1	chr13-41960246-41961799	0.001642401
TXK	chr4-48829990-48832054	0.001642308
TXK	chr4-48778335-48782249	0.001642034
DISC1	chr1-230641757-230643291	0.001641916
TXK	chr4-48906008-48907447	0.00164066
SMIM26	chr20-17964647-17970515	0.001640614
TXK	chr4-48340209-48343818	0.001640411
TXK	chr4-48132724-48134770	0.00163938
EPSTI1	chr13-42274446-42276937	0.001639229
TXK	chr4-48268904-48271350	0.001639128
TXK	chr4-48137499-48140111	0.001639084
DISC1	chr1-231611420-231615183	0.001638342
TXK	chr4-48127562-48131568	0.001638053
EPSTI1	chr13-41864269-41865157	0.001637071
TXK	chr4-47531438-47532346	0.001636571
EPSTI1	chr13-43877529-43880681	0.001636511
EPSTI1	chr13-42720270-42721138	0.00163564
EPSTI1	chr13-42354242-42356002	0.001635489
EPSTI1	chr13-44140933-44143959	0.001635333
EPSTI1	chr13-43057465-43058768	0.001635253
EPSTI1	chr13-42394660-42396984	0.001635099
SMIM26	chr20-19757289-19760417	0.00163473
EPSTI1	chr13-42990138-42993730	0.00163472
EPSTI1	chr13-42377372-42378860	0.001634382
TXK	chr4-48047022-48048606	0.001634073
SMIM26	chr20-19252319-19253451	0.001633741
DISC1	chr1-231625669-231629599	0.001633169
EPSTI1	chr13-43054400-43056124	0.00163304
DISC1	chr1-232628911-232631268	0.001632954
EPSTI1	chr13-43022823-43024445	0.001632912
SMIM26	chr20-18136830-18138787	0.001632863
DISC1	chr1-232620179-232621509	0.001632701
AC098829.1	chr4-15781347-15782504	0.001632621
EPSTI1	chr13-44191352-44192271	0.001632429
AC098829.1	chr4-15777232-15780300	0.001632133
EPSTI1	chr13-43044894-43046005	0.001631987
AC098829.1	chr4-15961666-15964092	0.001631922
FGFBP2	chr4-15781347-15782504	0.00163154
AC098829.1	chr4-16224011-16228982	0.00163109
FGFBP2	chr4-15777232-15780300	0.001631053
DISC1	chr1-232589742-232591271	0.001630921
FGFBP2	chr4-15961666-15964092	0.001630842
AC046134.2	chr3-139676830-139678479	0.001630784
DISC1	chr1-231803195-231804275	0.001630474
DISC1	chr1-232804830-232806476	0.001630025
FGFBP2	chr4-16224011-16228982	0.001630009
AC098829.1	chr4-16237543-16238685	0.001629971
SMIM26	chr20-18793423-18795193	0.001629657
TXK	chr4-47836531-47838053	0.001629372

AC098829.1	chr4-15765411-15767079	0.001629097
FGFBP2	chr4-16237543-16238685	0.001628893
TXK	chr4-48036470-48037322	0.001628677
KLF4	chr9-107281151-107285032	0.00162806
FGFBP2	chr4-15765411-15767079	0.00162802
SMIM26	chr20-18287400-18289414	0.001627723
KLF4	chr9-107465376-107466418	0.001627294
AC046134.2	chr3-139388731-139390832	0.001626424
3-Mar	chr5-126600349-126601756	0.001626207
FAM200B	chr4-15781347-15782504	0.001625758
3-Mar	chr5-126449433-126451362	0.001625729
SMIM26	chr20-18412209-18413649	0.001625405
FAM200B	chr4-15777232-15780300	0.001625273
FAM200B	chr4-15961666-15964092	0.001625062
SMIM26	chr20-18587434-18589035	0.001624904
TXK	chr4-47913355-47915552	0.001624834
TXK	chr4-48016125-48017719	0.001624745
SMIM26	chr20-18466264-18468264	0.001624328
MRPL13	chr8-120730818-120733426	0.001624266
FAM200B	chr4-16224011-16228982	0.001624232
3-Mar	chr5-126747762-126748608	0.001624155
KLF4	chr9-107480158-107492721	0.00162355
AC098829.1	chr4-15753765-15757509	0.001623411
AC098829.1	chr4-14856049-14856971	0.001623361
FAM200B	chr4-16237543-16238685	0.00162312
SMIM26	chr20-18496186-18497642	0.001623063
PID1	chr2-229330617-229331730	0.00162297
MRPL13	chr8-120706155-120707315	0.001622842
3-Mar	chr5-126422657-126425068	0.001622571
AC046134.2	chr3-138347254-138349699	0.001622438
FGFBP2	chr4-15753765-15757509	0.001622338
FGFBP2	chr4-14856049-14856971	0.001622288
PCNX4	chr14-60247260-60250830	0.001622274
FAM200B	chr4-15765411-15767079	0.001622251
PID1	chr2-229313292-229314446	0.001622172
SMIM26	chr20-18566585-18568141	0.001622008
MRPL13	chr8-120747671-120750315	0.001621931
AC046134.2	chr3-138367101-138368478	0.001621817
PID1	chr2-230456988-230457994	0.001621801
SMIM26	chr20-18506619-18509145	0.001621784
3-Mar	chr5-126758024-126759406	0.001621239
AC046134.2	chr3-138328335-138330389	0.001621228
PCNX4	chr14-60327297-60328382	0.001621097
PCNX4	chr14-60183228-60184747	0.001620782
AC046134.2	chr3-139343394-139344775	0.00162078
PID1	chr2-230447238-230449005	0.001620741
PID1	chr2-229408209-229409492	0.001620247
PCNX4	chr14-60980237-60982339	0.001620085
AC046134.2	chr3-138221745-138223325	0.001620068
AC046134.2	chr3-138186383-138188798	0.001620024

PCNX4	chr14-61101505-61104791	0.001619332
AC098829.1	chr4-14877062-14878024	0.001619154
MAP3K7CL	chr21-29208057-29209391	0.001618989
MAP3K7CL	chr21-29295003-29295890	0.001618841
3-Mar	chr5-126760288-126763909	0.001618833
PID1	chr2-229300468-229301937	0.00161852
MRPL13	chr8-120701935-120702782	0.001618497
AC046134.2	chr3-138370910-138371863	0.001618463
PCNX4	chr14-60753173-60754580	0.00161843
PCNX4	chr14-60330852-60332246	0.001618283
MRPL13	chr8-119600890-119602276	0.001618195
AC046134.2	chr3-138890591-138891688	0.001618178
FGFBP2	chr4-14877062-14878024	0.001618083
PID1	chr2-230441191-230442231	0.001618027
KLF4	chr9-107512985-107514248	0.001617914
MRPL13	chr8-120774824-120775866	0.001617607
PCNX4	chr14-60164057-60166695	0.001617528
AC098829.1	chr4-15702543-15704214	0.001616897
PID1	chr2-230223922-230227897	0.00161688
AC046134.2	chr3-138833140-138835653	0.001616801
3-Mar	chr5-126775641-126780862	0.001616658
MAP3K7CL	chr21-29297433-29303043	0.001616611
FAM200B	chr4-15753765-15757509	0.00161659
AC046134.2	chr3-138914770-138916730	0.001616571
PID1	chr2-230261936-230262843	0.001616568
AC098829.1	chr4-15653381-15657651	0.001616561
FAM200B	chr4-14856049-14856971	0.001616536
CALHM6	chr6-116100222-116102078	0.001616384
PCNX4	chr14-60734454-60735560	0.001616367
RALGPS2	chr1-179034092-179035539	0.001616186
PCNX4	chr14-61279693-61281742	0.001616184
PCNX4	chr14-60721278-60724539	0.001616151
RALGPS2	chr1-179025151-179027374	0.001616001
PID1	chr2-229415764-229417033	0.001615995
MAP3K7CL	chr21-29195630-29197709	0.001615929
RALGPS2	chr1-178724388-178726924	0.001615834
FGFBP2	chr4-15702543-15704214	0.00161583
RALGPS2	chr1-178541545-178543830	0.001615797
AC098829.1	chr4-15659486-15660892	0.001615753
AC098829.1	chr4-15000965-15005549	0.001615689
TEC	chr4-47173733-47174517	0.001615666
AC098829.1	chr4-15649683-15650588	0.001615566
MRPL13	chr8-119855115-119856867	0.001615561
AC046134.2	chr3-139329111-139329810	0.001615529
FGFBP2	chr4-15653381-15657651	0.001615494
PID1	chr2-230217431-230220866	0.001615266
PID1	chr2-230407930-230417284	0.001615157
PID1	chr2-229294884-229297841	0.00161502
PID1	chr2-230317440-230320103	0.001614952
RALGPS2	chr1-179080598-179083999	0.001614932

MRPL13	chr8-119873176-119875191	0.001614821
TRIM52	chr5-180820465-180821895	0.001614749
MRPL13	chr8-120868500-120869292	0.001614698
FGFBP2	chr4-15659486-15660892	0.001614686
PID1	chr2-229282676-229283769	0.001614649
FGFBP2	chr4-15000965-15005549	0.00161462
AC098829.1	chr4-15399939-15401926	0.001614608
RALGPS2	chr1-178093206-178094808	0.001614597
MRPL13	chr8-119832075-119833836	0.001614595
FGFBP2	chr4-15649683-15650588	0.001614498
MRPL13	chr8-119614274-119616021	0.001614448
MAP3K7CL	chr21-29347020-29348966	0.00161438
ZFP64	chr20-51500063-51501621	0.001614355
TRIM52	chr5-180813462-180817415	0.001614354
PCNX4	chr14-60151324-60152747	0.001614334
MRPL13	chr8-120808383-120812937	0.001614148
MRPL13	chr8-120444106-120445843	0.001614054
ZFP64	chr20-51497219-51499230	0.001614042
MRPL13	chr8-120861099-120862500	0.001613997
PID1	chr2-230326504-230328195	0.001613995
AC046134.2	chr3-138935804-138940217	0.001613843
FGFBP2	chr4-15399939-15401926	0.00161354
MRPL13	chr8-119638416-119639956	0.001613507
AC098829.1	chr4-15665482-15666704	0.001613295
PID1	chr2-230066867-230069210	0.001613261
3-Mar	chr5-126808138-126812549	0.001613223
AC046134.2	chr3-138786559-138787417	0.00161318
PID1	chr2-229919694-229923344	0.001613175
MAP3K7CL	chr21-29449141-29450168	0.001613159
MRPL13	chr8-120857001-120858110	0.00161314
TRIM52	chr5-180828770-180833550	0.001613113
AC046134.2	chr3-138943838-138947744	0.001612957
MRPL13	chr8-119955699-119956802	0.001612927
TRIM52	chr5-180801653-180812199	0.001612919
KLF4	chr9-107547155-107549546	0.001612793
FAM200B	chr4-14877062-14878024	0.001612346
AC098829.1	chr4-15689340-15692116	0.001612323
ZFP64	chr20-51528395-51530398	0.0016123
FGFBP2	chr4-15665482-15666704	0.00161223
MRPL13	chr8-119981919-119983258	0.00161206
ZFP64	chr20-51399927-51403031	0.001612025
PCNX4	chr14-60090982-60094814	0.001612001
MAP3K7CL	chr21-29638015-29640690	0.00161193
TRIM52	chr5-180688346-180690359	0.001611699
ZFP64	chr20-51492102-51495160	0.001611462
TEC	chr4-47462296-47464332	0.001611362
AC098829.1	chr4-15681017-15683559	0.001611332
FGFBP2	chr4-15689340-15692116	0.001611258
CALHM6	chr6-116250505-116255501	0.001611241
RALGPS2	chr1-179088565-179089575	0.001611024

TRIM52	chr5-180648756-180650637	0.001610792
MAP3K7CL	chr21-29183777-29184622	0.001610753
ZFP64	chr20-51404283-51405749	0.001610399
FGFBP2	chr4-15681017-15683559	0.001610269
AC046134.2	chr3-138608043-138610166	0.001610166
AC046134.2	chr3-138780683-138781779	0.00161011
FAM200B	chr4-15702543-15704214	0.001610103
KLF4	chr9-107587093-107588265	0.001610016
TRIM52	chr5-180860223-180862137	0.001609932
KLF4	chr9-107614982-107615944	0.001609858
FAM200B	chr4-15653381-15657651	0.001609767
PCNX4	chr14-59964122-59966155	0.001609733
KLF4	chr9-107611798-107612827	0.001609567
SASH1	chr6-149316354-149321325	0.001609515
ZFP64	chr20-51540584-51543583	0.001609469
ZFP64	chr20-53387993-53388870	0.001609454
KLF4	chr9-107635723-107639790	0.001609331
TRIM52	chr5-180589694-180592069	0.001609128
FAM200B	chr4-15659486-15660892	0.001608962
FAM200B	chr4-15000965-15005549	0.001608896
CUBN	chr10-17616323-17618058	0.001608845
FAM200B	chr4-15649683-15650588	0.001608774
CUBN	chr10-17643279-17645086	0.00160871
CUBN	chr10-17205663-17207863	0.001608476
ZFP64	chr20-51423265-51425914	0.00160847
SASH1	chr6-149365182-149366894	0.001608257
ZFP64	chr20-51413887-51415601	0.001608164
ZFP64	chr20-52190648-52192727	0.001608013
SASH1	chr6-149270567-149273874	0.001607935
ZFP64	chr20-52404578-52406402	0.001607916
3-Mar	chr5-127516968-127518638	0.001607896
KLF4	chr9-107644682-107645631	0.001607882
FAM200B	chr4-15399939-15401926	0.00160782
ZFP64	chr20-51630950-51632207	0.001607689
3-Mar	chr5-126845347-126846248	0.001607688
ZFP64	chr20-53000806-53002472	0.001607611
3-Mar	chr5-127228855-127230081	0.001607611
CUBN	chr10-17199990-17202615	0.001607562
TXN	chr9-110603983-110604864	0.001607553
ZFP64	chr20-51650519-51651328	0.001607534
KLF4	chr9-107760482-107761483	0.001607235
DNAJC15	chr13-42047387-42049823	0.001607173
ZFP64	chr20-51416317-51418440	0.00160715
ZFP64	chr20-52409701-52411358	0.001607118
TRIM52	chr5-180873391-180874448	0.001607009
KLF4	chr9-107866773-107868734	0.001606962
CD38	chr4-15781347-15782504	0.001606837
ZFP64	chr20-52971902-52974673	0.00160667
KLF4	chr9-107674075-107676923	0.001606624
TEC	chr4-47483751-47486533	0.001606612

3-Mar	chr5-128082022-128085243	0.001606608
KLF4	chr9-107749672-107752225	0.001606553
DNAJC15	chr13-42039217-42042177	0.001606533
FAM200B	chr4-15665482-15666704	0.001606515
TXN	chr9-111037452-111039596	0.00160651
3-Mar	chr5-128196652-128198484	0.001606495
CUBN	chr10-17212346-17213835	0.001606464
CUBN	chr10-17453055-17455269	0.00160639
TXN	chr9-110254528-110258209	0.001606389
CUBN	chr10-17647281-17649116	0.001606383
CD38	chr4-15777232-15780300	0.001606357
CD38	chr4-15961666-15964092	0.00160615
MAP3K7CL	chr21-29162730-29164727	0.001606016
CALHM6	chr6-116267788-116269269	0.001606007
TRIM52	chr5-181159034-181162103	0.001606
PCNX4	chr14-59576136-59577283	0.001605923
CALHM6	chr6-116392105-116393862	0.001605702
TRIM52	chr5-180493623-180495565	0.001605674
FAM200B	chr4-15689340-15692116	0.001605548
CD38	chr4-16224011-16228982	0.001605331
CALHM6	chr6-116386458-116387860	0.001605186
SASH1	chr6-149259376-149260594	0.001605112
RALGPS2	chr1-179129205-179130605	0.001604989
FAM200B	chr4-15681017-15683559	0.001604561
TXN	chr9-111068026-111069388	0.001604414
SASH1	chr6-149431618-149432589	0.001604407
KLF4	chr9-107976603-107977369	0.00160438
3-Mar	chr5-127072727-127074059	0.001604355
CUBN	chr10-17031875-17034293	0.001604331
CD38	chr4-16237543-16238685	0.001604231
DNAJC15	chr13-42270788-42273492	0.001604157
CUBN	chr10-17784684-17786113	0.001603943
CALHM6	chr6-116447354-116448322	0.001603598
MAP3K7CL	chr21-29099437-29100651	0.001603593
TXN	chr9-110156459-110158309	0.001603591
CALHM6	chr6-116369325-116372027	0.001603445
CD38	chr4-15765411-15767079	0.00160337
CUBN	chr10-17214584-17218877	0.001603345
SASH1	chr6-149139991-149141426	0.001603233
CUBN	chr10-17427369-17429626	0.001603203
CALHM6	chr6-116275290-116281915	0.001603159
MAP3K7CL	chr21-29089544-29091331	0.001603145
SASH1	chr6-149246447-149247663	0.001603116
TXN	chr9-111075959-111077140	0.001603005
SASH1	chr6-149230620-149235924	0.001602802
SASH1	chr6-149128330-149130156	0.001602787
TXN	chr9-111078114-111079246	0.001602778
DNAJC15	chr13-41960246-41961799	0.001602543
CUBN	chr10-16816179-16818505	0.001602517
MAP3K7CL	chr21-29078607-29081112	0.001602437

CUBN	chr10-16787123-16788590	0.001602053
TXN	chr9-110047594-110050261	0.001601887
CUBN	chr10-17225995-17242794	0.001601835
TXN	chr9-110064441-110067098	0.001601494
3-Mar	chr5-126851398-126855728	0.001601229
CUBN	chr10-17001808-17004010	0.001601206
CUBN	chr10-17028482-17030374	0.001601164
TXN	chr9-110148128-110149339	0.00160113
TRIM52	chr5-180352296-180354973	0.001600833
SASH1	chr6-149065601-149067099	0.00160077
TEC	chr4-47509966-47511026	0.001600705
TXN	chr9-110142949-110144470	0.001600554
CUBN	chr10-17022685-17027565	0.001600116
PCNX4	chr14-59482224-59485721	0.001600081
KLF4	chr9-107989137-107990213	0.001600011
SASH1	chr6-149450374-149452055	0.00159986
CALHM6	chr6-116461343-116463036	0.001599823
TXN	chr9-110021755-110022575	0.001599819
CUBN	chr10-16436210-16438102	0.001599579
3-Mar	chr5-127028709-127032288	0.00159945
DNAJC15	chr13-42274446-42276937	0.001599447
MAP3K7CL	chr21-29076116-29078100	0.001599354
TEC	chr4-48829990-48832054	0.001599104
RALGPS2	chr1-179141009-179143764	0.001598986
TEC	chr4-48778335-48782249	0.001598838
CALHM6	chr6-116615811-116617336	0.001598238
SASH1	chr6-149060075-149061318	0.001598079
CD38	chr4-15753765-15757509	0.001597775
CD38	chr4-14856049-14856971	0.001597724
TEC	chr4-48906008-48907447	0.001597499
CALHM6	chr6-116570418-116572939	0.001597396
DNAJC15	chr13-41864269-41865157	0.001597342
TEC	chr4-48340209-48343818	0.001597258
TRDMT1	chr10-17616323-17618058	0.001597031
TRDMT1	chr10-17643279-17645086	0.001596897
CALHM6	chr6-116464827-116465893	0.001596802
DNAJC15	chr13-43877529-43880681	0.001596797
CALHM6	chr6-116667570-116669422	0.001596743
TRDMT1	chr10-17205663-17207863	0.001596663
3-Mar	chr5-126972167-126973654	0.001596581
TRIM52	chr5-180291003-180295191	0.00159643
SASH1	chr6-148682754-148683666	0.001596338
SASH1	chr6-148757948-148762553	0.001596267
TEC	chr4-48132724-48134770	0.001596252
LONRF1	chr8-13131788-13133912	0.001596217
CALHM6	chr6-116475088-116477154	0.001596178
KLF4	chr9-108271928-108272977	0.001596096
TRIM52	chr5-180084669-180086442	0.001596075
3-Mar	chr5-126987626-126988700	0.00159602
TEC	chr4-48268904-48271350	0.001596008

SASH1	chr6-148746050-148749087	0.001595993
TEC	chr4-48137499-48140111	0.001595964
DNAJC15	chr13-42720270-42721138	0.001595948
TRIM52	chr5-180079479-180081332	0.001595887
DNAJC15	chr13-42354242-42356002	0.001595798
SASH1	chr6-148677456-148678767	0.00159576
TRDMT1	chr10-17199990-17202615	0.001595756
DNAJC15	chr13-44140933-44143959	0.001595648
DNAJC15	chr13-43057465-43058768	0.001595567
DNAJC15	chr13-42394660-42396984	0.001595419
LONRF1	chr8-12764947-12767684	0.001595373
TXN	chr9-109973917-109975110	0.001595347
RALGPS2	chr1-179175038-179176124	0.001595219
DNAJC15	chr13-42990138-42993730	0.00159505
TEC	chr4-48127562-48131568	0.00159496
KLF4	chr9-108469403-108470181	0.001594955
TRIM52	chr5-180089924-180091567	0.001594818
DNAJC15	chr13-42377372-42378860	0.001594719
TRDMT1	chr10-17212346-17213835	0.001594667
TRDMT1	chr10-17453055-17455269	0.001594593
TRDMT1	chr10-17647281-17649116	0.001594586
MPP7	chr10-28177906-28179899	0.001594397
TRIM52	chr5-180208584-180209514	0.001594362
RALGPS2	chr1-179292258-179295714	0.001594124
RALGPS2	chr1-179228341-179230632	0.001594114
INTS2	chr17-61138963-61139888	0.001594041
MAP3K7CL	chr21-29072714-29074577	0.001593826
SASH1	chr6-148656666-148657762	0.001593776
PCNX4	chr14-59463986-59466163	0.001593638
CD38	chr4-14877062-14878024	0.001593583
TEC	chr4-47531438-47532346	0.001593519
TRIM52	chr5-180069269-180073134	0.00159344
DNAJC15	chr13-43054400-43056124	0.001593407
DNAJC15	chr13-43022823-43024445	0.001593285
RALGPS2	chr1-179296951-179299579	0.001593146
CALHM6	chr6-116680275-116683159	0.001592975
DNAJC15	chr13-44191352-44192271	0.001592814
MPP7	chr10-28301656-28304836	0.001592621
TRDMT1	chr10-17031875-17034293	0.001592549
DNAJC15	chr13-43044894-43046005	0.001592382
TRDMT1	chr10-17784684-17786113	0.001592164
SASH1	chr6-147201568-147206137	0.001592157
MPP7	chr10-27741256-27746794	0.001592107
INTS2	chr17-61221069-61223413	0.001592079
SASH1	chr6-147321894-147323318	0.001591803
TXN	chr9-109110054-109111199	0.001591663
TRDMT1	chr10-17214584-17218877	0.00159157
LONRF1	chr8-12752769-12759229	0.001591479
TRDMT1	chr10-17427369-17429626	0.001591428
CD38	chr4-15702543-15704214	0.001591364

TEC	chr4-48047022-48048606	0.001591085
CD38	chr4-15653381-15657651	0.001591033
TXN	chr9-109117725-109120693	0.00159094
TRDMT1	chr10-16816179-16818505	0.001590749
TXN	chr9-109971957-109972872	0.001590669
LONRF1	chr8-11557713-11559394	0.001590358
RALGPS2	chr1-179326951-179328398	0.00159034
TRDMT1	chr10-16787123-16788590	0.001590288
CD38	chr4-15659486-15660892	0.001590237
CD38	chr4-15000965-15005549	0.001590174
TRDMT1	chr10-17225995-17242794	0.001590071
CD38	chr4-15649683-15650588	0.001590054
RALGPS2	chr1-179881459-179884414	0.001589979
INTS2	chr17-61250288-61252909	0.001589493
TRDMT1	chr10-17001808-17004010	0.001589448
TRDMT1	chr10-17028482-17030374	0.001589406
CD38	chr4-15399939-15401926	0.00158911
PCNX4	chr14-59187499-59190788	0.001589036
TXN	chr9-109320082-109321420	0.001588926
CALHM6	chr6-117481297-117484097	0.001588921
TRDMT1	chr10-17022685-17027565	0.001588365
TXN	chr9-109966404-109970056	0.001588288
MAP3K7CL	chr21-29022006-29026425	0.001588005
MPP7	chr10-27503314-27505394	0.001587964
TRDMT1	chr10-16436210-16438102	0.001587832
CD38	chr4-15665482-15666704	0.001587818
MAP3K7CL	chr21-28883700-28885953	0.00158764
CALHM6	chr6-117601332-117603593	0.00158728
RALGPS2	chr1-179364855-179366849	0.001586965
MPP7	chr10-28330082-28337372	0.001586902
CD38	chr4-15689340-15692116	0.001586861
CALHM6	chr6-117547384-117550015	0.001586785
TEC	chr4-47836531-47838053	0.001586509
RALGPS2	chr1-179876270-179878340	0.001586375
MAP3K7CL	chr21-28991919-28994107	0.001586131
CD38	chr4-15681017-15683559	0.001585886
TEC	chr4-48036470-48037322	0.001585832
SIAH1	chr16-49462241-49465244	0.001585671
LONRF1	chr8-11563692-11565023	0.001585534
LONRF1	chr8-12664886-12666069	0.001585489
RALGPS2	chr1-179814290-179815957	0.001585205
INTS2	chr17-61398945-61401400	0.001584784
MAP3K7CL	chr21-29017509-29019992	0.00158451
MPP7	chr10-27464450-27465748	0.001584411
MAP3K7CL	chr21-29002161-29004112	0.001584303
AL163541.1	chr13-110644673-110645982	0.001584155
CRNKL1	chr20-19757289-19760417	0.001583961
INTS2	chr17-62724313-62725563	0.001583676
AL163541.1	chr13-110614888-110617063	0.001583497
INTS2	chr17-62964941-62967293	0.001583295

CRNKL1	chr20-19942790-19943764	0.00158316
SIAH1	chr16-49441198-49442027	0.001583081
CRNKL1	chr20-19252319-19253451	0.001583002
INTS2	chr17-62995241-62996532	0.001582778
INTS2	chr17-62703323-62704875	0.001582479
INTS2	chr17-62968331-62969093	0.001582333
MPP7	chr10-27319561-27320567	0.001582257
TEC	chr4-47913355-47915552	0.001582091
TEC	chr4-48016125-48017719	0.001582003
AC027097.2	chr18-58657132-58659785	0.001581835
CRNKL1	chr20-19973717-19975717	0.001581826
AC027097.2	chr18-58649587-58650797	0.001581788
AL163541.1	chr13-109389658-109390568	0.001581752
CRNKL1	chr20-19827874-19828783	0.001581744
AL163541.1	chr13-110137010-110139065	0.001581575
CRNKL1	chr20-19934288-19938379	0.001581431
AL163541.1	chr13-110657883-110659022	0.001581406
LONRF1	chr8-11768146-11771260	0.001581287
AL163541.1	chr13-109790930-109791759	0.001581118
SIAH1	chr16-48630073-48631024	0.001581052
MPP7	chr10-27257004-27259789	0.001580398
INTS2	chr17-62651932-62654161	0.001580185
AL163541.1	chr13-109654219-109656055	0.001580085
MAN1A1	chr6-119119738-119120817	0.001580059
LONRF1	chr8-11823616-11824762	0.001580058
AL163541.1	chr13-110559583-110562674	0.001580054
LONRF1	chr8-11819888-11821159	0.001579995
MPP7	chr10-28366995-28369606	0.001579896
AL163541.1	chr13-110160219-110161899	0.001579845
MAN1A1	chr6-119345675-119351368	0.001579775
AL163541.1	chr13-109777970-109789489	0.001579601
MAN1A1	chr6-119076084-119079630	0.00157958
LONRF1	chr8-11801546-11809384	0.001579576
LONRF1	chr8-12658544-12659927	0.001579334
MAN1A1	chr6-119312525-119316202	0.001579214
AL163541.1	chr13-109704469-109706221	0.001578987
AC027097.2	chr18-58614605-58616490	0.001578881
MAN1A1	chr6-119339698-119341462	0.001578722
CRNKL1	chr20-19867073-19868906	0.001578501
CRNKL1	chr20-19915004-19916218	0.001578464
MAN1A1	chr6-119321906-119323315	0.001578434
AC027097.2	chr18-58668921-58673020	0.001578095
SIAH1	chr16-48622673-48624344	0.001578086
CRNKL1	chr20-20002637-20004160	0.001577856
INTS2	chr17-62626656-62628771	0.001577806
INTS2	chr17-61401914-61405967	0.001577777
AL163541.1	chr13-110912610-110917128	0.001577682
MAN1A1	chr6-118933925-118936184	0.001577475
MAN1A1	chr6-118767885-118769684	0.001577401
MPP7	chr10-27251855-27253689	0.001577348

LONRF1	chr8-11844943-11849855	0.001577304
AL163541.1	chr13-110663697-110666756	0.001577062
CRNKL1	chr20-19902356-19904027	0.001577023
AL163541.1	chr13-110471123-110472440	0.001576913
MAN1A1	chr6-118796214-118797136	0.001576837
AL163541.1	chr13-110535994-110537280	0.001576426
AC027097.2	chr18-58541148-58543215	0.001575739
INTS2	chr17-62611199-62612437	0.001575709
AC027097.2	chr18-58042784-58045997	0.001575424
MAN1A1	chr6-118745451-118747159	0.001575261
MPP7	chr10-28491838-28493022	0.001575255
AL163541.1	chr13-110519951-110522461	0.001575198
AL163541.1	chr13-110711448-110716670	0.001575139
MAN1A1	chr6-118890873-118897131	0.001575002
MAN1A1	chr6-118800198-118801279	0.001574957
AC027097.2	chr18-58321778-58322647	0.001574898
MPP7	chr10-28379859-28382506	0.00157489
MPP7	chr10-28402056-28403533	0.001574534
AC027097.2	chr18-58534766-58537272	0.001574425
AC027097.2	chr18-57665645-57667487	0.001574402
MAN1A1	chr6-118808472-118811152	0.001573939
AL163541.1	chr13-110675628-110677371	0.001573857
MPP7	chr10-28530624-28534852	0.001573628
MPP7	chr10-28399079-28401380	0.001573509
SIAH1	chr16-48620985-48622013	0.001573431
AL163541.1	chr13-110704776-110707461	0.001573359
CRNKL1	chr20-20016648-20018296	0.001573353
INTS2	chr17-62477512-62480274	0.001573209
MPP7	chr10-27239598-27244038	0.001572972
SIAH1	chr16-48383633-48386955	0.001572407
AC027097.2	chr18-58687299-58688967	0.001572278
AC027097.2	chr18-57649438-57650530	0.001571964
LONRF1	chr8-11856900-11864268	0.001571488
SIAH1	chr16-48243654-48245351	0.001571316
SIAH1	chr16-48378273-48379664	0.001571251
AC027097.2	chr18-57580785-57581901	0.001571246
SIAH1	chr16-47492323-47494296	0.001571158
SIAH1	chr16-48431836-48435328	0.001571112
MAN1A1	chr6-118718861-118720767	0.001570934
AC027097.2	chr18-57585777-57587358	0.001570374
CRNKL1	chr20-20051575-20053289	0.001570282
INTS2	chr17-61407948-61413336	0.001570266
AC027097.2	chr18-56650111-56653548	0.001570078
MPP7	chr10-28676575-28679358	0.001569882
AC027097.2	chr18-57629496-57631445	0.001569744
SIAH1	chr16-48317601-48319365	0.001569744
INTS2	chr17-62423390-62424581	0.001569733
CRNKL1	chr20-21125150-21126826	0.001569373
SIAH1	chr16-48365134-48367253	0.001569294
AC027097.2	chr18-57621014-57623153	0.001569264

CRNKL1	chr20-20254864-20255771	0.001569004
MPP7	chr10-27152491-27157451	0.001568856
LRMDA	chr10-74967611-74969186	0.001568736
SIAH1	chr16-48353273-48354375	0.001568578
CRNKL1	chr20-20259299-20260179	0.001568454
SIAH1	chr16-48607059-48614062	0.001568236
SIAH1	chr16-47460066-47462409	0.001568224
SIAH1	chr16-48458038-48458866	0.001567821
LRMDA	chr10-75043376-75044284	0.001567732
AC027097.2	chr18-58754287-58756483	0.001567554
CRNKL1	chr20-20710523-20713677	0.001567553
CRNKL1	chr20-21099605-21103941	0.001567135
AC027097.2	chr18-56637327-56639632	0.001566838
LRMDA	chr10-74853723-74855417	0.001566784
CRNKL1	chr20-20733907-20734996	0.001566349
MAN1A1	chr6-118708118-118711050	0.001566309
LONRF1	chr8-11901943-11903034	0.001566182
AC027097.2	chr18-58764019-58764749	0.001566181
CRNKL1	chr20-20779839-20781309	0.001565945
MPP7	chr10-29408772-29410507	0.001565939
INTS2	chr17-62063718-62066728	0.001565887
ANGPTL1	chr1-179034092-179035539	0.001565299
LRMDA	chr10-75099524-75100571	0.001565294
ANGPTL1	chr1-179025151-179027374	0.00156512
INTS2	chr17-61862739-61864308	0.001564979
ANGPTL1	chr1-178724388-178726924	0.001564959
ANGPTL1	chr1-178541545-178543830	0.001564923
SIAH1	chr16-48558145-48559822	0.001564911
SIAH1	chr16-48602229-48604330	0.001564857
LONRF1	chr8-11864871-11869863	0.001564811
MICAL2	chr11-12221836-12226510	0.00156451
ANGPTL1	chr1-179080598-179083999	0.001564086
MICAL2	chr11-12195595-12196972	0.001564053
ANGPTL1	chr1-178093206-178094808	0.00156376
INTS2	chr17-61926904-61928667	0.001563642
LRMDA	chr10-75110223-75113472	0.001563305
MAN1A1	chr6-118704316-118707375	0.001562977
MICAL2	chr11-12235130-12236978	0.001562706
LRMDA	chr10-75187652-75188657	0.001562455
LONRF1	chr8-11896617-11898849	0.00156221
LRMDA	chr10-74841829-74843224	0.001562112
EMC4	chr15-33194167-33195609	0.001561976
TIAM1	chr21-32411054-32414217	0.001561899
TIAM1	chr21-32569070-32570559	0.001561798
LRMDA	chr10-75191841-75192847	0.001561688
MICAL2	chr11-12192552-12193501	0.001561586
BMPR1A	chr10-86535188-86537395	0.001561505
TIAM1	chr21-32611382-32613802	0.00156143
BMPR1A	chr10-86520558-86522923	0.001561263
TIAM1	chr21-32392291-32394016	0.001561074

MAN1A1	chr6-118650170-118652466	0.001561036
EMC4	chr15-33154130-33155402	0.00156097
SSR3	chr3-156663765-156665790	0.001560687
SSR3	chr3-156605580-156607342	0.001560425
TIAM1	chr21-32726568-32729296	0.001560403
LONRF1	chr8-11876981-11879161	0.001560313
ANGPTL1	chr1-179088565-179089575	0.0015603
MICAL2	chr11-12673728-12675390	0.001560115
RSL24D1	chr15-55279140-55280894	0.001559969
MICAL2	chr11-10930371-10932228	0.001559954
MICAL2	chr11-10897848-10901039	0.001559902
RSL24D1	chr15-55281723-55283668	0.001559754
EMC4	chr15-33226102-33227222	0.001559684
LONRF1	chr8-11885662-11889148	0.001559639
BMPR1A	chr10-86411170-86414003	0.001559613
BMPR1A	chr10-85612039-85613376	0.001559563
MICAL2	chr11-13275304-13280540	0.001559477
MAN1A1	chr6-118558366-118559580	0.001559447
LRMDA	chr10-75209040-75211987	0.001559267
BMPR1A	chr10-86709728-86712712	0.001559219
MICAL2	chr11-13207494-13208776	0.001558765
MICAL2	chr11-12159102-12160335	0.001558712
BMPR1A	chr10-86399546-86402767	0.001558694
SSR3	chr3-156673441-156678584	0.001558486
TIAM1	chr21-32278130-32280243	0.001558117
EMC4	chr15-33152374-33153460	0.001558013
RSL24D1	chr15-55275703-55278534	0.001557721
SSR3	chr3-156553993-156555933	0.001557552
BMPR1A	chr10-87859885-87865844	0.001557284
MICAL2	chr11-12124325-12125492	0.001556761
MICAL2	chr11-10933507-10934810	0.001556585
LRMDA	chr10-74823874-74829683	0.001556279
BMPR1A	chr10-87817034-87819445	0.001555908
DTWD2	chr5-119308227-119310466	0.001555704
RSL24D1	chr15-55266870-55269049	0.001555597
MICAL2	chr11-12109497-12112329	0.001555594
SSR3	chr3-156679515-156681532	0.00155556
BMPR1A	chr10-86755508-86757858	0.001555343
EMC4	chr15-33150849-33151663	0.001555338
DTWD2	chr5-119302592-119304739	0.001555336
RSL24D1	chr15-55288734-55291104	0.001555331
RSL24D1	chr15-55249902-55251622	0.001555088
RSL24D1	chr15-55254833-55256653	0.001554927
EMC4	chr15-34037902-34040260	0.001554915
LRMDA	chr10-75230294-75236737	0.001554695
RSL24D1	chr15-55247144-55248392	0.001554656
SSR3	chr3-155852484-155855743	0.001554633
ANGPTL1	chr1-179129205-179130605	0.001554455
EMC4	chr15-33125896-33127001	0.001554239
SSR3	chr3-155805253-155806799	0.001554035

TIAM1	chr21-31098345-31099504	0.001553867
MICAL2	chr11-12086143-12088588	0.001553866
SSR3	chr3-156524290-156525251	0.001553828
DTWD2	chr5-119314233-119317231	0.001553826
DTWD2	chr5-119299237-119300591	0.001553704
SSR3	chr3-156815385-156818224	0.00155361
DTWD2	chr5-119267631-119272194	0.001553536
SSR3	chr3-155862189-155863237	0.001553447
ZDHHC13	chr11-18705416-18708198	0.001553239
RSL24D1	chr15-55220846-55223364	0.001553167
BMPR1A	chr10-87739698-87740912	0.00155309
LRMDA	chr10-76403774-76404925	0.00155305
DTWD2	chr5-119272933-119279848	0.001552894
SSR3	chr3-156824659-156827480	0.001552791
DTWD2	chr5-119069991-119072260	0.001552746
TIAM1	chr21-31772304-31773497	0.001552692
DTWD2	chr5-119285466-119291469	0.00155264
BMPR1A	chr10-87083490-87085166	0.001552576
ZDHHC13	chr11-18720530-18722604	0.001552492
BMPR1A	chr10-86939060-86940192	0.001552115
LRMDA	chr10-75430060-75432210	0.001552084
RSL24D1	chr15-55195874-55197931	0.001552024
SSR3	chr3-155743957-155746000	0.001551974
BMPR1A	chr10-86965935-86973455	0.001551869
SSR3	chr3-155869687-155872390	0.00155177
RSL24D1	chr15-55217325-55218414	0.001551739
BMPR1A	chr10-87092154-87096363	0.0015516
SSR3	chr3-156483062-156484992	0.001551567
ZDHHC13	chr11-18697049-18701287	0.001551404
BMPR1A	chr10-86958035-86960995	0.001551096
MICAL2	chr11-11149157-11150928	0.001551022
LRMDA	chr10-74588883-74589983	0.001550802
MICAL2	chr11-12046255-12047991	0.001550701
DTWD2	chr5-119320526-119324785	0.001550441
EMC4	chr15-34100589-34102774	0.001550116
ZDHHC13	chr11-19116354-19117921	0.00154995
DTWD2	chr5-119030893-119033188	0.001549758
ATP6V1B2	chr8-19127808-19128782	0.001549561
BMPR1A	chr10-87659369-87660733	0.001549516
LRMDA	chr10-75294451-75296252	0.001549511
LRMDA	chr10-75407406-75409935	0.001549154
BMPR1A	chr10-87203166-87204362	0.001549129
ATP6V1B2	chr8-19013249-19014720	0.00154879
ANGPTL1	chr1-179141009-179143764	0.001548641
DTWD2	chr5-119353412-119360064	0.00154832
PPAT	chr4-56465847-56468627	0.001548277
EMC4	chr15-34986817-34989244	0.001548188
TIAM1	chr21-31132178-31133913	0.001548181
ATP6V1B2	chr8-19459642-19460988	0.001548152
ZDHHC13	chr11-18671764-18673052	0.001547962

DTWD2	chr5-119367444-119369087	0.001547624
DTWD2	chr5-119325386-119327689	0.00154755
ZDHHC13	chr11-19240296-19242741	0.001547512
RSL24D1	chr15-55317744-55320751	0.001547491
EMC4	chr15-34318208-34319669	0.001547484
DTWD2	chr5-119334562-119342682	0.001547463
EMC4	chr15-34968805-34970512	0.001547451
EMC4	chr15-34209350-34210660	0.001547447
PPAT	chr4-56504954-56506897	0.001547352
PPAT	chr4-56434405-56438050	0.00154726
EMC4	chr15-34223885-34225892	0.001547126
BMPR1A	chr10-87504280-87505901	0.001547012
BMPR1A	chr10-87341596-87343450	0.001546987
MICAL2	chr11-11856444-11858260	0.001546947
ZDHHC13	chr11-20156142-20157450	0.001546947
PPAT	chr4-56757147-56759641	0.001546704
DTWD2	chr5-119328726-119334048	0.001546656
ZDHHC13	chr11-19711748-19714921	0.001546596
EMC4	chr15-34331191-34338704	0.001546533
PPAT	chr4-56654674-56657011	0.001546465
BACH1	chr21-29208057-29209391	0.001546455
LRMDA	chr10-75399596-75404660	0.00154638
BACH1	chr21-29295003-29295890	0.001546314
MICAL2	chr11-11840942-11845871	0.001546277
LRMDA	chr10-75375653-75376490	0.001546242
TIAM1	chr21-31730648-31733589	0.001546052
PPAT	chr4-56529997-56531607	0.001546046
LRMDA	chr10-74585050-74587567	0.001545938
PPAT	chr4-56544280-56545263	0.001545766
DTWD2	chr5-118987621-118989335	0.001545708
DTWD2	chr5-119411604-119412496	0.001545327
SSR3	chr3-157128020-157131872	0.001545251
RGS2	chr1-193104036-193105968	0.001545148
RGS2	chr1-193120863-193123527	0.001545129
EMC4	chr15-34582301-34584667	0.001545118
PPAT	chr4-56799629-56800610	0.001545055
ANGPTL1	chr1-179175038-179176124	0.001544992
MICAL2	chr11-11848457-11849823	0.00154483
ZDHHC13	chr11-18631902-18635390	0.001544634
RGS2	chr1-193057919-193061434	0.001544316
RGS2	chr1-192936119-192938035	0.001544257
BACH1	chr21-29297433-29303043	0.001544185
PPAT	chr4-56386687-56388724	0.001544178
RGS2	chr1-192940286-192942648	0.001544175
ATP6V1B2	chr8-19473168-19473926	0.001544105
ANGPTL1	chr1-179950610-179956170	0.00154408
ANGPTL1	chr1-179292258-179295714	0.001543933
ANGPTL1	chr1-179228341-179230632	0.001543922
EMC4	chr15-34341376-34343678	0.001543908
RGS2	chr1-192954622-192955579	0.001543874

BACH1	chr21-29195630-29197709	0.001543534
RGS2	chr1-193417335-193419102	0.001543477
ANGPTL1	chr1-179296951-179299579	0.001542986
RGS2	chr1-192907571-192908617	0.001542785
DTWD2	chr5-119413847-119414744	0.001542784
ZDHHC13	chr11-18617524-18619015	0.001542384
PTAR1	chr9-70409796-70415000	0.001542284
EMC4	chr15-34514055-34515555	0.001542054
BACH1	chr21-29347020-29348966	0.001542054
PPAT	chr4-55345674-55348013	0.001541717
PTAR1	chr9-70405737-70408773	0.001541708
SSR3	chr3-157136856-157138238	0.001541618
DTWD2	chr5-119451995-119453400	0.001541458
PPAT	chr4-56821133-56822304	0.001541436
EMC4	chr15-34365117-34368781	0.001541154
TIAM1	chr21-31158751-31160030	0.001541146
SSR3	chr3-157436399-157438415	0.001541065
BACH1	chr21-29449141-29450168	0.001540887
RGS2	chr1-193478944-193479982	0.001540854
ZDHHC13	chr11-18587528-18591150	0.001540722
PPAT	chr4-55946711-55950287	0.001540636
PPAT	chr4-55394998-55399804	0.00154053
SSR3	chr3-157173442-157176676	0.001540486
TIAM1	chr21-31658888-31661054	0.001540459
EMC4	chr15-34436190-34438274	0.001540306
ANGPTL1	chr1-179326951-179328398	0.001540269
SSR3	chr3-157144409-157164230	0.00154003
ANGPTL1	chr1-179881459-179884414	0.001539921
BACH1	chr21-29638015-29640690	0.001539712
PTAR1	chr9-70417964-70423113	0.001539712
RGS2	chr1-192805355-192815423	0.001539501
ATP6V1B2	chr8-20200118-20202755	0.001539323
RSL24D1	chr15-55402642-55404063	0.001539259
ATP6V1B2	chr8-19474658-19476389	0.001539163
PPAT	chr4-55544766-55547742	0.001538901
RGS2	chr1-193537213-193538683	0.001538785
RGS2	chr1-193568457-193570296	0.001538654
PPAT	chr4-55853020-55854731	0.001538612
BACH1	chr21-29183777-29184622	0.001538589
ATP6V1B2	chr8-19754589-19759001	0.001538582
ZDHHC13	chr11-18525222-18528970	0.001538499
ATP6V1B2	chr8-20196412-20199000	0.001538463
ATP6V1B2	chr8-20302469-20304475	0.001538329
RGS2	chr1-193558360-193559328	0.001538258
ATP6V1B2	chr8-19816289-19818695	0.001537766
PTAR1	chr9-70397040-70399325	0.001537728
ATP6V1B2	chr8-20492681-20493967	0.001537503
TIAM1	chr21-31654266-31655909	0.001537478
ATP6V1B2	chr8-19695251-19700780	0.001537325
PPAT	chr4-56906387-56910011	0.001537245

ATP6V1B2	chr8-20435038-20437625	0.001537117
CCT8	chr21-29208057-29209391	0.001537043
ANGPTL1	chr1-179364855-179366849	0.001537001
CCT8	chr21-29295003-29295890	0.001536902
TIAM1	chr21-31557431-31560364	0.001536809
TIAM1	chr21-31397661-31398811	0.001536685
PTAR1	chr9-70562933-70564866	0.001536613
ANGPTL1	chr1-179876270-179878340	0.00153643
ATP6V1B2	chr8-20188819-20189949	0.001535952
ATP6V1B2	chr8-19496079-19498471	0.001535914
PTAR1	chr9-68844041-68845463	0.001535786
RGS2	chr1-192608632-192610466	0.001535768
RGS2	chr1-192516405-192517665	0.001535691
ATP6V1B2	chr8-19510655-19512210	0.001535641
TIAM1	chr21-31354223-31355429	0.001535565
TIAM1	chr21-31180447-31183067	0.001535443
ATP6V1B2	chr8-19843423-19845483	0.001535344
ANGPTL1	chr1-179814290-179815957	0.001535296
ZDHHC13	chr11-18392356-18397112	0.001535296
RGS2	chr1-192538606-192540116	0.001534981
CCT8	chr21-29297433-29303043	0.001534785
PTAR1	chr9-68974186-68976341	0.001534625
PKP4	chr2-159614735-159616893	0.001534581
RGS2	chr1-192157749-192159265	0.001534509
CCT8	chr21-29195630-29197709	0.00153414
PPAT	chr4-56957924-56959055	0.001534084
BACH1	chr21-29162730-29164727	0.001534065
TTC28-AS1	chr22-28673898-28675650	0.00153378
PTAR1	chr9-68779060-68781168	0.001533764
RSL24D1	chr15-55407175-55409149	0.001533722
TIAM1	chr21-31201007-31202012	0.001533718
ATP6V1B2	chr8-20186281-20187696	0.001533643
RGS2	chr1-192544972-192546933	0.001533588
RGS2	chr1-192575157-192576265	0.001533508
GNPAT	chr1-231039001-231042019	0.0015334
ATP6V1B2	chr8-19886295-19887284	0.001533385
ZDHHC13	chr11-18105485-18106575	0.00153308
PKP4	chr2-157466757-157471565	0.001533044
TIAM1	chr21-31184044-31188454	0.00153303
TTC28-AS1	chr22-28441940-28443851	0.001532998
PKP4	chr2-157463439-157465803	0.00153292
CCT8	chr21-29347020-29348966	0.001532668
GNPAT	chr1-230976744-230980426	0.001532602
PTAR1	chr9-70257533-70261077	0.001532466
PPAT	chr4-56976023-56980163	0.001532369
RSL24D1	chr15-55992893-55994301	0.001532306
AC009226.1	chr2-207632515-207633651	0.001532302
RSL24D1	chr15-56042739-56044145	0.00153228
ZDHHC13	chr11-18277478-18278491	0.001532276
ZDHHC13	chr11-18010093-18014387	0.001532246

GNPAT	chr1-231211097-231212009	0.001532201
ZDHHC13	chr11-18382510-18385040	0.001532086
RSL24D1	chr15-55497916-55498976	0.001531916
PKP4	chr2-159515321-159517004	0.001531913
BACH1	chr21-29099437-29100651	0.00153175
AC009226.1	chr2-207623722-207627927	0.001531668
CCT8	chr21-29449141-29450168	0.001531509
PTAR1	chr9-69027387-69029311	0.001531395
BACH1	chr21-29089544-29091331	0.001531322
AC009226.1	chr2-207661831-207662873	0.001531293
PPAT	chr4-57109421-57111517	0.001531291
TTC28-AS1	chr22-28678983-28680388	0.001531199
ZDHHC13	chr11-18280261-18281333	0.001530769
BACH1	chr21-29078607-29081112	0.001530647
PKP4	chr2-157452242-157454506	0.001530631
GNPAT	chr1-231240233-231243195	0.001530479
PKP4	chr2-159326482-159327339	0.001530429
ZDHHC13	chr11-18320368-18324924	0.001530369
RSL24D1	chr15-56242760-56246795	0.001530345
CCT8	chr21-29638015-29640690	0.001530343
AC009226.1	chr2-207710456-207713071	0.001530146
GNPAT	chr1-230972364-230973757	0.00152983
GNPAT	chr1-231419856-231423107	0.001529748
PKP4	chr2-157627783-157629583	0.001529678
GNPAT	chr1-231335013-231339668	0.001529671
PTAR1	chr9-68704576-68707052	0.001529602
TTC28-AS1	chr22-27917954-27920686	0.001529586
PKP4	chr2-159285492-159287658	0.001529491
AC009226.1	chr2-207766783-207771528	0.001529378
PKP4	chr2-157331418-157332472	0.001529264
CCT8	chr21-29183777-29184622	0.001529226
GNPAT	chr1-231527593-231530795	0.001529192
PKP4	chr2-157327277-157328322	0.001528842
PKP4	chr2-157402819-157405108	0.001528716
AC009226.1	chr2-206764575-206766425	0.001528612
PTAR1	chr9-69945317-69947357	0.001528591
PKP4	chr2-157426512-157445564	0.001528523
NME9	chr3-139388731-139390832	0.001528436
AC009226.1	chr2-207619382-207620452	0.001528398
PTAR1	chr9-69035230-69037047	0.00152839
AC009226.1	chr2-208024844-208026377	0.001528217
SAMD3	chr6-131061437-131064005	0.001528183
PKP4	chr2-157409445-157422458	0.001528012
PTAR1	chr9-69173550-69175924	0.001527893
BACH1	chr21-29076116-29078100	0.001527701
TTC28-AS1	chr22-27132939-27134971	0.001527632
SAMD3	chr6-131134946-131136824	0.001527623
PTAR1	chr9-69671217-69673504	0.001527592
AC009226.1	chr2-207135028-207136545	0.001527515
PKP4	chr2-159090667-159092557	0.001527504

PTAR1	chr9-69168386-69169602	0.00152746
TTC28-AS1	chr22-27112897-27113971	0.001527433
PTAR1	chr9-69758793-69760657	0.001527266
PTAR1	chr9-69120684-69123032	0.001527156
RSL24D1	chr15-56364926-56365841	0.001527116
TTC28-AS1	chr22-28741187-28743329	0.001527033
GNPAT	chr1-230867619-230869355	0.00152672
TTC28-AS1	chr22-28810092-28812735	0.001526711
GNPAT	chr1-231603305-231604429	0.001526448
TTC28-AS1	chr22-28822561-28823867	0.001526432
SAMD3	chr6-130575585-130577826	0.001526185
TTC28-AS1	chr22-27800755-27802653	0.0015261
TTC28-AS1	chr22-27150146-27151140	0.00152601
PTAR1	chr9-68559719-68561004	0.001525694
AC009226.1	chr2-208253742-208255942	0.001525674
AC009226.1	chr2-207141141-207145023	0.001525615
TTC28-AS1	chr22-29117740-29118744	0.001525359
TTC28-AS1	chr22-28798138-28803283	0.001525275
GNPAT	chr1-230153476-230154697	0.001525206
GNPAT	chr1-230641757-230643291	0.001525007
GNPAT	chr1-230190894-230191810	0.001525002
SAMD3	chr6-131199399-131200763	0.001524871
TTC28-AS1	chr22-27669130-27670769	0.001524859
TTC28-AS1	chr22-28829115-28830141	0.001524779
CCT8	chr21-29162730-29164727	0.001524729
NME9	chr3-138347254-138349699	0.00152469
NME9	chr3-138367101-138368478	0.001524107
AC009226.1	chr2-207155163-207158174	0.001524086
TTC28-AS1	chr22-28772190-28774316	0.001524002
PKP4	chr2-157874574-157877736	0.001523956
PKP4	chr2-158967925-158970186	0.001523709
TTC28-AS1	chr22-29071169-29074535	0.001523675
TTC28-AS1	chr22-28791640-28794233	0.001523671
GNPAT	chr1-230149074-230150887	0.001523609
NME9	chr3-138328335-138330389	0.001523552
TTC28-AS1	chr22-28882492-28884827	0.001523408
AC009226.1	chr2-207526668-207535064	0.001523235
SAMD3	chr6-130513331-130514533	0.00152314
NME9	chr3-139343394-139344775	0.001523134
NME9	chr3-138136165-138138310	0.001523071
NME9	chr3-138173703-138175534	0.001522982
AC009226.1	chr2-207162901-207168515	0.001522973
PRAG1	chr8-9002042-9003735	0.001522891
NME9	chr3-138221745-138223325	0.001522462
CCT8	chr21-29099437-29100651	0.001522428
BACH1	chr21-29072714-29074577	0.001522422
NME9	chr3-138186383-138188798	0.001522419
PRAG1	chr8-9065619-9066416	0.001522005
CCT8	chr21-29089544-29091331	0.001522002
AC009226.1	chr2-208264970-208267387	0.001521926

SAMD3	chr6-131281109-131282328	0.001521751
GNPAT	chr1-231611420-231615183	0.001521688
AC009226.1	chr2-207235541-207237078	0.001521459
CCT8	chr21-29078607-29081112	0.00152133
SAMD3	chr6-130183280-130184554	0.001521127
SAMD3	chr6-130222692-130224222	0.001521001
NME9	chr3-138370910-138371863	0.001520953
SAMD3	chr6-130131260-130133423	0.001520758
NME9	chr3-138890591-138891688	0.001520687
SAMD3	chr6-130215090-130216075	0.001520636
PRAG1	chr8-8994684-8995737	0.00152052
GNPAT	chr1-230145424-230146818	0.001519502
PKP4	chr2-158455968-158458833	0.001519408
NME9	chr3-138833140-138835653	0.001519393
NME9	chr3-138914770-138916730	0.001519177
AC009226.1	chr2-207311197-207312019	0.001519067
PKP4	chr2-157897071-157900361	0.001518874
CD302	chr2-159795656-159798733	0.001518808
SAMD3	chr6-130027771-130029957	0.001518643
AC009226.1	chr2-207523910-207525994	0.001518408
CCT8	chr21-29076116-29078100	0.001518402
PRAG1	chr8-9087814-9089370	0.001518225
NME9	chr3-139329111-139329810	0.001518201
CD302	chr2-159710847-159714994	0.001518131
PKP4	chr2-158142171-158143317	0.00151717
GNPAT	chr1-231625669-231629599	0.001516883
BACH1	chr21-29022006-29026425	0.001516862
AC009226.1	chr2-207498393-207503758	0.001516657
CD302	chr2-159903009-159905852	0.001516643
PRAG1	chr8-8918976-8920667	0.001516635
NME9	chr3-138935804-138940217	0.001516614
BACH1	chr21-28883700-28885953	0.001516511
AC009226.1	chr2-207504617-207507396	0.001516054
NME9	chr3-138786559-138787417	0.001515988
NME9	chr3-138943838-138947744	0.001515782
SAMD3	chr6-130017900-130021663	0.001515545
CD302	chr2-159614735-159616893	0.00151551
BACH1	chr21-28991919-28994107	0.00151507
LCLAT1	chr2-30334613-30338258	0.001515
LCLAT1	chr2-30208333-30209874	0.001514742
GNPAT	chr1-231803195-231804275	0.001514379
LCLAT1	chr2-30265626-30268726	0.00151436
GNPAT	chr1-230066166-230069349	0.001514178
PRAG1	chr8-9149573-9152197	0.001513909
LCLAT1	chr2-30144160-30169627	0.001513816
BACH1	chr21-29017509-29019992	0.001513523
PRAG1	chr8-8891608-8894319	0.001513385
BACH1	chr21-29002161-29004112	0.001513325
NME9	chr3-138608043-138610166	0.001513155
CCT8	chr21-29072714-29074577	0.001513155

NME9	chr3-138780683-138781779	0.001513104
SAMD3	chr6-129766942-129767841	0.001513039
LCLAT1	chr2-30222252-30227560	0.001512999
CD302	chr2-159515321-159517004	0.001512876
LCLAT1	chr2-30421121-30423240	0.001512805
CD302	chr2-160269311-160271568	0.001512795
SAMD3	chr6-129709359-129710662	0.001512131
SAMD3	chr6-129747935-129750736	0.00151206
LCLAT1	chr2-30257154-30258791	0.00151195
SAMD3	chr6-129683436-129687939	0.001511915
PRAG1	chr8-9555135-9557088	0.001511737
PRAG1	chr8-8868574-8873109	0.001511689
CD302	chr2-159326482-159327339	0.001511412
PRAG1	chr8-8847937-8849668	0.001510758
LCLAT1	chr2-30229829-30240751	0.001510717
SAMD3	chr6-129671494-129673262	0.001510619
GSR	chr8-30141162-30142596	0.001510564
CD302	chr2-159285492-159287658	0.001510485
GSR	chr8-30154569-30158928	0.001510291
LCLAT1	chr2-30252062-30256127	0.001510187
LCLAT1	chr2-30138753-30139874	0.001510108
CD302	chr2-160379014-160382280	0.001509532
PRAG1	chr8-8796735-8798434	0.001509287
GSR	chr8-30131799-30137748	0.001509085
LCLAT1	chr2-30445887-30448675	0.001508882
SAMD3	chr6-129498735-129499976	0.001508823
CD302	chr2-159090667-159092557	0.001508523
CD302	chr2-160393874-160394788	0.001508523
CD302	chr2-160387562-160389294	0.001508305
CD302	chr2-160405532-160408693	0.001508136
SAMD3	chr6-129490033-129491882	0.001507844
CCT8	chr21-29022006-29026425	0.001507629
IRS2	chr13-110644673-110645982	0.001507484
CCT8	chr21-28883700-28885953	0.001507279
GSR	chr8-30655667-30659145	0.001507252
GSR	chr8-30111754-30116951	0.001507037
PRAG1	chr8-8701340-8702980	0.00150687
IRS2	chr13-110614888-110617063	0.001506859
AVEN	chr15-33194167-33195609	0.00150641
PRAG1	chr8-8227080-8229452	0.001506143
PRAG1	chr8-8260475-8261401	0.001506036
CCT8	chr21-28991919-28994107	0.001505848
PRAG1	chr8-8244309-8245032	0.001505737
LCLAT1	chr2-29316761-29318555	0.001505549
CD302	chr2-160410838-160412787	0.001505534
LCLAT1	chr2-30502951-30504216	0.001505444
AVEN	chr15-33154130-33155402	0.001505441
PRAG1	chr8-8237888-8239242	0.001505333
PRAG1	chr8-8333227-8334716	0.001505276
IRS2	chr13-109389658-109390568	0.001505199

IRS2	chr13-110137010-110139065	0.00150503
IRS2	chr13-110657883-110659022	0.001504869
CD302	chr2-158967925-158970186	0.001504774
GSR	chr8-30103000-30106053	0.001504677
IRS2	chr13-109790930-109791759	0.001504594
PRAG1	chr8-8669638-8671006	0.001504444
CCT8	chr21-29017509-29019992	0.00150431
AVEN	chr15-33226102-33227222	0.001504201
CCT8	chr21-29002161-29004112	0.001504113
PRAG1	chr8-8344577-8348201	0.001503924
LCLAT1	chr2-30711481-30712248	0.001503829
IRS2	chr13-109654219-109656055	0.001503612
IRS2	chr13-110559583-110562674	0.001503582
LCLAT1	chr2-30712813-30715386	0.001503462
IRS2	chr13-110160219-110161899	0.001503384
PRAG1	chr8-8384058-8388641	0.001503331
IRS2	chr13-109777970-109789489	0.00150315
TAF9	chr5-68711167-68712743	0.001503134
LCLAT1	chr2-31218914-31220301	0.001502607
AVEN	chr15-33152374-33153460	0.001502589
IRS2	chr13-109704469-109706221	0.001502566
LCLAT1	chr2-29314631-29315670	0.00150251
TAF9	chr5-69042526-69043807	0.001502299
TAF9	chr5-68214470-68217960	0.001502229
TAF9	chr5-68432775-68436095	0.001502025
GSR	chr8-30721888-30724685	0.001501974
TAF9	chr5-68279089-68282494	0.001501615
TAF9	chr5-68196629-68197431	0.001501545
GSR	chr8-30067057-30102417	0.001501444
TAF9	chr5-68239187-68241937	0.0015014
TAF9	chr5-68274992-68275998	0.001501373
IRS2	chr13-110912610-110917128	0.001501324
CD302	chr2-160427628-160429842	0.001501104
TAF9	chr5-68287606-68289064	0.001501041
IRS2	chr13-110663697-110666756	0.001500733
TAF9	chr5-68255199-68257399	0.001500701
TAF9	chr5-68368863-68370171	0.001500683
IRS2	chr13-110471123-110472440	0.001500594
TAF9	chr5-68250706-68253072	0.001500589
TAF9	chr5-68330941-68332991	0.001500376
LCLAT1	chr2-31233619-31234929	0.001500211
ABAT	chr16-9101811-9104363	0.001500173
IRS2	chr13-110535994-110537280	0.001500128
TAF9	chr5-69368625-69370776	0.001500059
AVEN	chr15-33150849-33151663	0.001500008
AVEN	chr15-34037902-34040260	0.001499601
ABAT	chr16-9105184-9111272	0.001499127
AVEN	chr15-32908063-32909526	0.001499016
TAF9	chr5-69093211-69095059	0.001498989
IRS2	chr13-110519951-110522461	0.001498961

AVEN	chr15-33125896-33127001	0.001498949
IRS2	chr13-110711448-110716670	0.001498904
ABAT	chr16-9089805-9101086	0.001498214
CD302	chr2-160650639-160652903	0.001498139
TAF9	chr5-69331454-69335905	0.001497941
IRS2	chr13-110675628-110677371	0.001497683
IRS2	chr13-110704776-110707461	0.001497209
CD302	chr2-160482206-160483851	0.001497109
LCLAT1	chr2-31318295-31320289	0.001497087
GSR	chr8-30056178-30062519	0.001497045
GSR	chr8-30725479-30728586	0.00149664
GSR	chr8-31032169-31034808	0.001496245
ABAT	chr16-9112132-9116252	0.001495892
CD302	chr2-160490899-160494686	0.001495891
AVEN	chr15-34100589-34102774	0.001494971
TAF9	chr5-69165798-69167925	0.001494657
TAF9	chr5-69234144-69236429	0.001494557
ABAT	chr16-9078568-9079796	0.00149434
GSR	chr8-29528533-29532694	0.001494211
GSR	chr8-30811779-30813375	0.001493906
GSR	chr8-30743299-30744993	0.001493638
GSR	chr8-29554972-29556642	0.001493176
AVEN	chr15-34986817-34989244	0.001493111
ABAT	chr16-9127393-9128499	0.001492596
AVEN	chr15-34318208-34319669	0.001492433
AVEN	chr15-34968805-34970512	0.001492401
AVEN	chr15-34209350-34210660	0.001492397
GSR	chr8-30050040-30051959	0.001492381
AVEN	chr15-34223885-34225892	0.001492088
MAST4	chr5-67192658-67197538	0.001491895
TAF9	chr5-69216434-69218825	0.001491829
TAF9	chr5-69188518-69190830	0.00149172
AVEN	chr15-34331191-34338704	0.001491517
MAST4	chr5-67187053-67189422	0.001491257
GSR	chr8-29737134-29738269	0.001490766
ABAT	chr16-8961693-8964868	0.001490528
AVEN	chr15-34582301-34584667	0.00149015
MAST4	chr5-67202055-67205176	0.001490036
MAST4	chr5-66828259-66829497	0.001489791
MAST4	chr5-67181453-67182872	0.001489546
MAST4	chr5-67003561-67006456	0.001489239
GSR	chr8-29806236-29806957	0.001489216
AVEN	chr15-34341376-34343678	0.001488984
GSR	chr8-29773315-29775473	0.001488821
FKTN	chr9-105243581-105246125	0.001488815
MAST4	chr5-67162867-67168216	0.001488702
MAST4	chr5-66170914-66172368	0.001488389
FKTN	chr9-105191234-105192431	0.00148833
ABAT	chr16-8955460-8961140	0.001488055
FKTN	chr9-105405104-105406245	0.001487949

MAST4	chr5-67514678-67516244	0.001487872
FKTN	chr9-105693241-105695770	0.001487438
AVEN	chr15-34514055-34515555	0.001487194
SLC35D1	chr1-66300508-66301701	0.001487047
ORC5	chr7-103343464-103345317	0.001487011
ORC5	chr7-103296738-103298118	0.001486852
SLC35D1	chr1-66281820-66283072	0.00148685
FKTN	chr9-105141018-105143442	0.001486762
FKTN	chr9-105447400-105448885	0.001486698
ABAT	chr16-8943453-8945334	0.001486623
FKTN	chr9-105557598-105558703	0.00148641
AVEN	chr15-34365117-34368781	0.001486328
MAST4	chr5-67205953-67208198	0.001486234
AVEN	chr15-34436190-34438274	0.001485509
CCDC125	chr5-68711167-68712743	0.001485367
FKTN	chr9-105127463-105129418	0.001485119
ABAT	chr16-8888986-8892514	0.001485107
SLC35D1	chr1-66327516-66338593	0.001485078
ORC5	chr7-103279467-103281375	0.001485066
CCDC125	chr5-69042526-69043807	0.001484541
CCDC125	chr5-68214470-68217960	0.00148447
MAST4	chr5-66142544-66145865	0.001484342
ORC5	chr7-103346330-103348854	0.001484339
CCDC125	chr5-68432775-68436095	0.001484269
MAST4	chr5-67221236-67223651	0.001484201
AL136456.1	chr1-193104036-193105968	0.001484177
AL136456.1	chr1-193120863-193123527	0.001484159
ORC5	chr7-103073871-103076122	0.001483916
FKTN	chr9-105114148-105116083	0.001483894
CCDC125	chr5-68279089-68282494	0.001483863
CCDC125	chr5-68196629-68197431	0.001483796
AL390957.1	chr1-193104036-193105968	0.00148375
AL390957.1	chr1-193120863-193123527	0.001483731
ORC5	chr7-103148524-103150175	0.001483719
CCDC125	chr5-68239187-68241937	0.001483651
CCDC125	chr5-68274992-68275998	0.001483624
AL136456.1	chr1-193057919-193061434	0.001483378
AL136456.1	chr1-192936119-192938035	0.001483319
SLC35D1	chr1-66274549-66276830	0.001483305
CCDC125	chr5-68287606-68289064	0.001483296
AL136456.1	chr1-192940286-192942648	0.001483241
SLC35D1	chr1-66349264-66352759	0.001483157
CCDC125	chr5-68255199-68257399	0.001482961
ABAT	chr16-8880012-8880854	0.001482954
AL390957.1	chr1-193057919-193061434	0.001482952
AL136456.1	chr1-192954622-192955579	0.001482952
CCDC125	chr5-68368863-68370171	0.001482944
AL390957.1	chr1-192936119-192938035	0.001482892
ABAT	chr16-8643896-8645509	0.001482862
CCDC125	chr5-68250706-68253072	0.001482851

AL390957.1	chr1-192940286-192942648	0.001482814
MAST4	chr5-67208942-67209956	0.00148279
SLC35D1	chr1-66380004-66381793	0.001482785
CCDC125	chr5-68330941-68332991	0.001482639
AL136456.1	chr1-193417335-193419102	0.001482571
SLC35D1	chr1-66372257-66376594	0.001482553
AL390957.1	chr1-192954622-192955579	0.001482526
SLC35D1	chr1-66395032-66397678	0.001482411
ABAT	chr16-8673595-8676017	0.001482352
FKTN	chr9-105063499-105065390	0.001482346
CCDC125	chr5-69368625-69370776	0.001482328
AL390957.1	chr1-193417335-193419102	0.001482144
MAST4	chr5-67214748-67216851	0.001481961
AL136456.1	chr1-192907571-192908617	0.001481907
AL390957.1	chr1-192907571-192908617	0.001481479
TMEM38B	chr9-105243581-105246125	0.001481434
ABAT	chr16-8631703-8633845	0.00148142
CCDC125	chr5-69093211-69095059	0.00148127
TMEM38B	chr9-105191234-105192431	0.001480951
ABAT	chr16-8870650-8871656	0.001480822
ABAT	chr16-8796546-8799322	0.001480787
TMEM38B	chr9-105405104-105406245	0.001480572
SLC35D1	chr1-66438352-66439744	0.001480541
CCDC125	chr5-69331454-69335905	0.001480235
TMEM38B	chr9-105693241-105695770	0.001480063
AL136456.1	chr1-193478944-193479982	0.001480052
ABAT	chr16-8866159-8870044	0.001479948
AL390957.1	chr1-193478944-193479982	0.001479625
ORC5	chr7-104207036-104208709	0.001479488
TMEM38B	chr9-105141018-105143442	0.001479391
TMEM38B	chr9-105447400-105448885	0.001479326
FKTN	chr9-105051680-105053893	0.00147915
MAST4	chr5-65924438-65928648	0.00147909
TMEM38B	chr9-105557598-105558703	0.001479041
ABAT	chr16-8620611-8622659	0.001478764
AL136456.1	chr1-192805355-192815423	0.001478751
AL390957.1	chr1-192805355-192815423	0.001478325
AL136456.1	chr1-193537213-193538683	0.001478064
AL136456.1	chr1-193568457-193570296	0.001477938
TMEM38B	chr9-105127463-105129418	0.001477755
SLC35D1	chr1-66445217-66447003	0.001477698
AL390957.1	chr1-193537213-193538683	0.001477636
AL136456.1	chr1-193558360-193559328	0.001477557
AL390957.1	chr1-193568457-193570296	0.00147751
AL136456.1	chr1-194056002-194057331	0.001477475
AL390957.1	chr1-193558360-193559328	0.00147713
AL390957.1	chr1-194056002-194057331	0.001477047
CCDC125	chr5-69165798-69167925	0.001476988
CCDC125	chr5-69234144-69236429	0.001476892
MAST4	chr5-65480101-65484168	0.001476864

ABAT	chr16-8567398-8568310	0.001476705
MAST4	chr5-65562138-65564136	0.001476556
TMEM38B	chr9-105114148-105116083	0.001476537
CHROMR	chr2-178522493-178524142	0.001475804
CHROMR	chr2-179049145-179050366	0.00147573
CHROMR	chr2-178477783-178481709	0.001475382
CHROMR	chr2-179234680-179236086	0.001475346
CHROMR	chr2-178530097-178531778	0.001475293
CHROMR	chr2-179031522-179032721	0.001475259
SLC35D1	chr1-66923727-66926193	0.001475175
AL136456.1	chr1-192608632-192610466	0.001475166
MAST4	chr5-65845028-65846033	0.001475124
MAST4	chr5-65623492-65625669	0.001475097
AL136456.1	chr1-192516405-192517665	0.001475092
TMEM38B	chr9-105063499-105065390	0.001474994
AL136456.1	chr1-194289344-194290310	0.001474941
AL390957.1	chr1-192608632-192610466	0.001474741
AL390957.1	chr1-192516405-192517665	0.001474667
AL136456.1	chr1-192538606-192540116	0.001474409
ORC5	chr7-104922638-104923525	0.001474301
CCDC125	chr5-69216434-69218825	0.001474195
RAD17	chr5-68711167-68712743	0.001474113
CCDC125	chr5-69188518-69190830	0.001474086
AL390957.1	chr1-192538606-192540116	0.001473985
MAST4	chr5-65719115-65723523	0.00147398
FKTN	chr9-105006418-105007859	0.001473979
SLC35D1	chr1-66929190-66932143	0.001473642
AL390957.1	chr1-192157749-192159265	0.001473532
RAD17	chr5-69042526-69043807	0.001473293
RAD17	chr5-68214470-68217960	0.001473221
FKTN	chr9-104774890-104776213	0.001473185
AL136456.1	chr1-192544972-192546933	0.001473071
RAD17	chr5-68432775-68436095	0.001473023
AL136456.1	chr1-192575157-192576265	0.001472995
CHROMR	chr2-179263137-179265793	0.001472805
CHROMR	chr2-178449071-178452354	0.001472683
AL390957.1	chr1-192544972-192546933	0.001472649
RAD17	chr5-68279089-68282494	0.00147262
SLC35D1	chr1-67683371-67687139	0.001472574
AL390957.1	chr1-192575157-192576265	0.001472571
RAD17	chr5-68196629-68197431	0.00147255
SLC35D1	chr1-66933786-66935056	0.001472468
RAD17	chr5-68239187-68241937	0.001472408
RAD17	chr5-68274992-68275998	0.001472382
FKTN	chr9-104763458-104765896	0.001472113
RAD17	chr5-68287606-68289064	0.001472056
TMEM38B	chr9-105051680-105053893	0.001471814
RAD17	chr5-68255199-68257399	0.001471724
RAD17	chr5-68368863-68370171	0.001471707
RAD17	chr5-68250706-68253072	0.001471614

FKTN	chr9-104868527-104869815	0.001471427
RAD17	chr5-68330941-68332991	0.001471405
RAD17	chr5-69368625-69370776	0.001471096
SLC35D1	chr1-67052642-67055033	0.001470382
ORC5	chr7-104933040-104934113	0.001470198
RAD17	chr5-69093211-69095059	0.001470046
ORC5	chr7-105387586-105389730	0.001469658
FKTN	chr9-104746965-104749120	0.001469533
RAD17	chr5-69331454-69335905	0.001469018
FKTN	chr9-104989458-104994114	0.001468297
CHROMR	chr2-178413055-178415319	0.001467982
FKTN	chr9-104872858-104873815	0.001467691
ORC5	chr7-105351180-105356155	0.001467675
RIN2	chr20-19757289-19760417	0.001467222
ORC5	chr7-104939817-104948257	0.00146722
SLC35D1	chr1-67674102-67675318	0.001467184
SLC35D1	chr1-67170607-67172499	0.001466912
TMEM38B	chr9-105006418-105007859	0.001466669
ZHX2	chr8-123273210-123275868	0.001466597
AL589693.1	chr6-157368861-157370238	0.001466496
RIN2	chr20-19942790-19943764	0.001466479
RIN2	chr20-19252319-19253451	0.001466332
CCR6	chr6-167825590-167828126	0.001466151
ORC5	chr7-105344970-105348216	0.001466092
DAB1	chr1-59506831-59507760	0.00146598
ZHX2	chr8-123394403-123397288	0.001465941
TMEM38B	chr9-104774890-104776213	0.00146588
RAD17	chr5-69165798-69167925	0.001465796
RAD17	chr5-69234144-69236429	0.0014657
AL589693.1	chr6-157379612-157382771	0.001465471
DAB1	chr1-59295928-59297973	0.001465427
RIN2	chr20-19973717-19975717	0.001465244
RIN2	chr20-19827874-19828783	0.001465167
ZHX2	chr8-123240204-123241880	0.001465006
RIN2	chr20-19934288-19938379	0.001464877
TMEM38B	chr9-104763458-104765896	0.001464812
SIRT1	chr10-69072673-69074673	0.001464771
DAB1	chr1-59672881-59675234	0.001464768
FKTN	chr9-104967853-104969071	0.001464593
FKTN	chr9-104926180-104928813	0.001464583
ORC5	chr7-104956568-104958036	0.001464359
TMEM38B	chr9-104868527-104869815	0.00146413
ZHX2	chr8-122779351-122789599	0.00146413
ZHX2	chr8-123415782-123417904	0.001463895
CCR6	chr6-167698976-167700099	0.001463803
ORC5	chr7-105267270-105270492	0.001463593
DAB1	chr1-59291901-59293727	0.001463313
DAB1	chr1-59696466-59697814	0.001463306
CHROMR	chr2-178193869-178195311	0.001463278
DAB1	chr1-59692434-59693934	0.001463268

SLC35D1	chr1-67194050-67195333	0.001463197
RAD17	chr5-69216434-69218825	0.001463023
SIRT1	chr10-69060553-69066667	0.001462924
RAD17	chr5-69188518-69190830	0.001462915
SLC35D1	chr1-67428500-67432116	0.001462806
ZHX2	chr8-123036569-123039827	0.001462696
RIN2	chr20-18793423-18795193	0.001462666
CCR6	chr6-167350274-167351834	0.001462492
ZHX2	chr8-122792809-122795675	0.001462371
TMEM38B	chr9-104746965-104749120	0.001462244
ZHX2	chr8-122918378-122919654	0.001462196
CCR6	chr6-167118476-167123198	0.001462184
RIN2	chr20-19867073-19868906	0.001462164
SIRT1	chr10-69050869-69059939	0.001462138
RIN2	chr20-19915004-19916218	0.001462128
SIRT1	chr10-67849082-67850450	0.001461931
ZHX2	chr8-123156043-123161749	0.001461836
SIRT1	chr10-67772707-67773595	0.001461802
CCR6	chr6-167111604-167115345	0.001461797
SIRT1	chr10-69043606-69050211	0.001461797
ZHX2	chr8-123519541-123521189	0.001461763
GAS7	chr17-8997742-8998627	0.001461673
ZHX2	chr8-123041242-123043051	0.001461618
GAS7	chr17-9001526-9004762	0.001461582
RIN2	chr20-20002637-20004160	0.001461566
CHROMR	chr2-177617791-177619597	0.001461389
SLC35D1	chr1-67307098-67308489	0.001461348
DAB1	chr1-59146423-59147513	0.001461191
ZHX2	chr8-122909775-122911566	0.001461149
CCR6	chr6-166862090-166863322	0.001461146
CHROMR	chr2-177210721-177219304	0.001461083
ZHX2	chr8-122798548-122800398	0.001461049
TMEM38B	chr9-104989458-104994114	0.001461014
ORC5	chr7-104960430-104962327	0.001461001
CCR6	chr6-166625984-166629199	0.001460952
DAB1	chr1-58941246-58943291	0.00146088
SIRT1	chr10-68987839-68990611	0.001460868
DAB1	chr1-58967863-58969085	0.001460866
AL365295.1	chr14-52175558-52176977	0.001460803
RIN2	chr20-19902356-19904027	0.001460794
CHROMR	chr2-178111947-178113503	0.001460675
CHROMR	chr2-177753608-177754664	0.001460616
CHROMR	chr2-177551846-177553779	0.001460504
DAB1	chr1-59019702-59021050	0.00146044
TMEM38B	chr9-104872858-104873815	0.001460412
CCR6	chr6-167089455-167096171	0.001460393
ORC5	chr7-105209457-105211583	0.001460152
ZHX2	chr8-123534316-123535297	0.001460103
AL589693.1	chr6-157535024-157536628	0.001459914
ZHX2	chr8-123072027-123073994	0.001459511

CCR6	chr6-166949264-166960619	0.001459461
SIRT1	chr10-67761651-67765900	0.001459381
AL365295.1	chr14-51988273-51990621	0.001459376
SIRT1	chr10-67883424-67886578	0.00145932
SIRT1	chr10-68954835-68959585	0.001459244
CCR6	chr6-166337814-166343926	0.001459134
AL365295.1	chr14-52267323-52268894	0.001459054
DAB1	chr1-58922117-58923142	0.001459041
ZHX2	chr8-123095817-123096737	0.001458975
GAS7	chr17-9020588-9023618	0.001458922
SIRT1	chr10-68599415-68600923	0.001458844
CCR6	chr6-166586685-166589423	0.001458825
RPS6KA2	chr6-167825590-167828126	0.00145855
ZHX2	chr8-123536362-123539236	0.00145841
CCR6	chr6-166381313-166384391	0.001458409
RIN2	chr20-18587434-18589035	0.0014584
CCR6	chr6-167079524-167080493	0.001458351
BTBD11	chr12-107317230-107322881	0.001458297
SIRT1	chr10-68720204-68722743	0.001458261
BTBD11	chr12-107327770-107330323	0.001458183
ZHX2	chr8-123085424-123086754	0.001458146
SIRT1	chr10-68526674-68528676	0.001457907
CCR6	chr6-166333107-166335158	0.001457864
SIRT1	chr10-68899966-68902606	0.001457786
ORC5	chr7-104980904-104985335	0.001457769
CCR6	chr6-166997459-167000262	0.001457519
SIRT1	chr10-68826793-68828792	0.001457496
RIN2	chr20-20016648-20018296	0.001457396
TMEM38B	chr9-104967853-104969071	0.001457329
TMEM38B	chr9-104926180-104928813	0.001457318
BTBD11	chr12-107091720-107094735	0.001457306
ORC5	chr7-105019773-105023881	0.001457123
CCR6	chr6-167046641-167048347	0.001457026
CHROMR	chr2-177392140-177393847	0.001456922
SIRT1	chr10-67612642-67613782	0.001456534
CCR6	chr6-166416841-166418064	0.001456514
BTBD11	chr12-107027269-107028189	0.001456504
CCR6	chr6-166540746-166542337	0.001456345
DAB1	chr1-58738666-58740445	0.001456313
RPS6KA2	chr6-167698976-167700099	0.001456214
ORC5	chr7-105005994-105018426	0.001456158
ZHX2	chr8-123539801-123542335	0.00145608
BTBD11	chr12-106986157-106987825	0.001455963
BTBD11	chr12-107368392-107371052	0.001455828
RIN2	chr20-18566585-18568141	0.001455801
DAB1	chr1-58754628-58756004	0.001455766
CCR6	chr6-166258426-166260082	0.001455675
DAB1	chr1-58902954-58904554	0.00145559
SIRT1	chr10-68073894-68076210	0.001455538
SIRT1	chr10-68471212-68472434	0.001455499

CCR6	chr6-166487633-166489015	0.001455394
RPS6KA2	chr6-167350274-167351834	0.00145491
GAS7	chr17-9239025-9242483	0.001454786
CHROMR	chr2-177220692-177230055	0.001454669
RPS6KA2	chr6-167118476-167123198	0.001454604
BTBD11	chr12-106954431-106957749	0.001454576
RIN2	chr20-20051575-20053289	0.001454551
RPS6KA2	chr6-167111604-167115345	0.001454219
AL365295.1	chr14-52309701-52319278	0.00145418
DAB1	chr1-58698763-58700752	0.001453784
RPS6KA2	chr6-166862090-166863322	0.00145357
ZHX2	chr8-123767546-123771230	0.001453565
RPS6KA2	chr6-166625984-166629199	0.001453377
RIN2	chr20-20254864-20255771	0.001453367
DAB1	chr1-58777205-58787295	0.001453325
SIRT1	chr10-68406276-68408065	0.001453217
SIRT1	chr10-68330019-68337423	0.001453038
GAS7	chr17-10149760-10152809	0.00145288
RIN2	chr20-20259299-20260179	0.001452859
RPS6KA2	chr6-167089455-167096171	0.001452822
Z94721.1	chr6-167825590-167828126	0.001452529
AL589693.1	chr6-157548795-157551119	0.00145248
DAB1	chr1-58882862-58886476	0.001452428
CHROMR	chr2-177281039-177282095	0.001452121
RIN2	chr20-20710523-20713677	0.001452024
GAS7	chr17-10126716-10128505	0.001452009
RPS6KA2	chr6-166949264-166960619	0.001451893
DAB1	chr1-58812506-58817227	0.001451585
RPS6KA2	chr6-166337814-166343926	0.001451569
GAS7	chr17-9575258-9577584	0.00145142
BTBD11	chr12-107371880-107374827	0.00145141
RPS6KA2	chr6-166586685-166589423	0.001451262
BTBD11	chr12-106773460-106775474	0.00145125
GAS7	chr17-10033051-10035710	0.001451219
RIN2	chr20-20733907-20734996	0.001450908
RPS6KA2	chr6-166381313-166384391	0.001450848
GAS7	chr17-10156011-10157998	0.001450824
GAS7	chr17-10017267-10022890	0.001450816
RPS6KA2	chr6-167079524-167080493	0.001450789
RIN2	chr20-20779839-20781309	0.001450532
RPS6KA2	chr6-166333107-166335158	0.001450305
AL589693.1	chr6-157273135-157275682	0.001450283
GAS7	chr17-10013088-10014246	0.001450256
Z94721.1	chr6-167698976-167700099	0.001450203
GAS7	chr17-10059102-10061687	0.001449963
RPS6KA2	chr6-166997459-167000262	0.001449963
CHROMR	chr2-177239047-177243573	0.001449927
GAS7	chr17-10729108-10730687	0.001449789
SLC2A9	chr4-10954558-10955357	0.001449589
RPS6KA2	chr6-167046641-167048347	0.001449472

DAB1	chr1-58618150-58619013	0.001449461
CHROMR	chr2-177250105-177266949	0.001449043
RPS6KA2	chr6-166416841-166418064	0.001448963
GAS7	chr17-10118755-10121233	0.001448927
Z94721.1	chr6-167350274-167351834	0.001448905
RPS6KA2	chr6-166540746-166542337	0.001448793
Z94721.1	chr6-167118476-167123198	0.0014486
AL365295.1	chr14-52324265-52326096	0.001448497
CREB3L2	chr7-139133056-139134520	0.001448282
Z94721.1	chr6-167111604-167115345	0.001448216
RPS6KA2	chr6-166258426-166260082	0.001448126
AL365295.1	chr14-52694432-52697570	0.001447897
RPS6KA2	chr6-166487633-166489015	0.001447848
AL365295.1	chr14-52706110-52707981	0.001447594
Z94721.1	chr6-166862090-166863322	0.001447569
GAS7	chr17-10171100-10173168	0.001447456
GAS7	chr17-10062624-10064745	0.001447405
Z94721.1	chr6-166625984-166629199	0.001447376
Z94721.1	chr6-167089455-167096171	0.001446826
ANXA2R	chr5-43601825-43604964	0.00144675
BTBD11	chr12-107684619-107686602	0.001446632
CREB3L2	chr7-139131350-139132317	0.001446514
GAS7	chr17-10696528-10700487	0.001446402
SLC2A9	chr4-10671086-10672364	0.001446343
AL365295.1	chr14-52550695-52554639	0.001446328
AL589693.1	chr6-157560802-157563068	0.001446226
AL589693.1	chr6-157235404-157237514	0.001446192
BTBD11	chr12-106759845-106760739	0.001446174
GAS7	chr17-10117078-10118185	0.001446071
DAB1	chr1-58544999-58547489	0.001446033
Z94721.1	chr6-166949264-166960619	0.0014459
ANXA2R	chr5-43191557-43194307	0.001445883
ANXA2R	chr5-43312175-43314526	0.00144579
AL365295.1	chr14-52729320-52731116	0.001445738
Z94721.1	chr6-166337814-166343926	0.001445578
GAS7	chr17-10113073-10116563	0.001445524
CREB3L2	chr7-139118122-139119913	0.001445465
GAS7	chr17-10197436-10199437	0.001445437
ANXA2R	chr5-43555781-43558090	0.001445385
Z94721.1	chr6-166586685-166589423	0.00144527
AL365295.1	chr14-52949634-52952045	0.001445168
SLC2A9	chr4-10640319-10641190	0.001445113
Z94721.1	chr6-166381313-166384391	0.00144486
AL365295.1	chr14-52326971-52328631	0.001444852
Z94721.1	chr6-167079524-167080493	0.001444801
AL365295.1	chr14-52917625-52919391	0.001444596
AL365295.1	chr14-52351073-52353290	0.001444515
ANXA2R	chr5-43396237-43397592	0.001444411
SLC2A9	chr4-10460663-10462211	0.001444356
Z94721.1	chr6-166333107-166335158	0.00144432

CREB3L2	chr7-139111292-139113346	0.001444272
AL365295.1	chr14-53108094-53110298	0.001444193
AL365295.1	chr14-52741249-52743176	0.001444013
Z94721.1	chr6-166997459-167000262	0.001443977
ANXA2R	chr5-43120265-43122814	0.001443945
ANXA2R	chr5-43514223-43515990	0.00144388
AL365295.1	chr14-52790497-52792695	0.001443719
CDK6	chr7-93146567-93148872	0.001443694
CDK6	chr7-92717657-92719209	0.001443589
Z94721.1	chr6-167046641-167048347	0.001443488
ANXA2R	chr5-43483037-43485870	0.001443427
AL589693.1	chr6-157220570-157224636	0.001443372
CDK6	chr7-92730495-92733049	0.001443194
BTBD11	chr12-107759355-107762252	0.001443192
Z94721.1	chr6-166416841-166418064	0.001442981
CDK6	chr7-93231235-93233058	0.00144287
Z94721.1	chr6-166540746-166542337	0.001442813
CDK6	chr7-93117202-93118708	0.001442723
SLC2A9	chr4-10456206-10458191	0.001442434
CDK6	chr7-92446734-92449171	0.001442375
CDK6	chr7-92684848-92685913	0.001442265
Z94721.1	chr6-166258426-166260082	0.00144215
CREB3L2	chr7-139102196-139110791	0.001442134
CDK6	chr7-92809395-92810479	0.001442083
AL365295.1	chr14-53126461-53128811	0.001441886
Z94721.1	chr6-166487633-166489015	0.001441871
CDK6	chr7-92831942-92837624	0.001441775
BTBD11	chr12-108312251-108314567	0.001441453
ANXA2R	chr5-43015290-43021643	0.001441224
CDK6	chr7-92527287-92530253	0.001441138
BTBD11	chr12-106747268-106748712	0.00144096
ANXA2R	chr5-43109316-43111439	0.001440908
CDK6	chr7-92423519-92426024	0.001440668
ANXA2R	chr5-43006912-43011400	0.001440634
CREB3L2	chr7-138161430-138162191	0.001440489
AL589693.1	chr6-157099560-157100849	0.001440148
BTBD11	chr12-108332573-108334745	0.001440113
ANXA2R	chr5-43036356-43045777	0.001439972
CDK6	chr7-92633100-92636028	0.001439949
CREB3L2	chr7-138257330-138258432	0.001439931
CDK6	chr7-94003447-94005197	0.001439697
AL365295.1	chr14-53151091-53154717	0.001439659
CREB3L2	chr7-139090718-139098083	0.00143932
AL365295.1	chr14-53216911-53218266	0.001439161
SLC2A9	chr4-10180779-10181840	0.001439139
CDK6	chr7-92588510-92591207	0.001439082
AL365295.1	chr14-53161490-53162531	0.001438778
ANXA2R	chr5-43104171-43106360	0.001438675
SLC2A9	chr4-10092654-10097677	0.001438599
ANXA2R	chr5-43063602-43069165	0.001438516

SLC2A9	chr4-10044344-10045377	0.001438515
CDK6	chr7-92620902-92621943	0.001438464
ANXA2R	chr5-42989132-42996745	0.001438025
CREB3L2	chr7-137999987-138003222	0.001437999
SLC2A9	chr4-10032281-10033887	0.001437628
BTBD11	chr12-108514433-108518996	0.001437559
BTBD11	chr12-106357180-106359114	0.001437365
SLC2A9	chr4-10098556-10101581	0.001437262
SLC2A9	chr4-10018382-10021983	0.001437151
SLC2A9	chr4-10022823-10024847	0.001437072
CREB3L2	chr7-139074768-139075816	0.001437046
CREB3L2	chr7-138459113-138462205	0.001436959
SLC2A9	chr4-10005784-10007384	0.001436909
CREB3L2	chr7-138980676-138982353	0.001436668
CREB3L2	chr7-139034885-139036468	0.001436531
CREB3L2	chr7-139041330-139045838	0.001436222
BTBD11	chr12-106300717-106307143	0.001435939
SLC2A9	chr4-10122520-10124857	0.00143592
CDK6	chr7-92244785-92247266	0.001435808
BTBD11	chr12-106245150-106250402	0.001435729
AL589693.1	chr6-157053992-157055265	0.001435615
CREB3L2	chr7-138903110-138904284	0.001435602
ZNHIT6	chr1-86787654-86789390	0.001435573
SLC2A9	chr4-10105912-10111518	0.001435328
BTBD11	chr12-106226752-106228310	0.001435268
ZNHIT6	chr1-86773127-86775702	0.001435252
SLC2A9	chr4-9691065-9692401	0.001435065
ANXA2R	chr5-42948600-42954736	0.001434555
SLC2A9	chr4-10114882-10120087	0.001434495
CREB3L2	chr7-137934404-137937101	0.001433982
CREB3L2	chr7-138751929-138753958	0.00143387
CREB3L2	chr7-138886220-138887145	0.001433734
ZNHIT6	chr1-86703497-86706485	0.001433089
CREB3L2	chr7-138875635-138876372	0.001432699
INVS	chr9-99150295-99151188	0.001432335
ZNHIT6	chr1-85047405-85049064	0.001432249
INVS	chr9-100097899-100100243	0.001432036
INVS	chr9-99136819-99137792	0.00143185
ZNHIT6	chr1-84995398-84999371	0.001431714
INVS	chr9-99905831-99907771	0.001431692
ANXA2R	chr5-42908081-42909648	0.001431552
SLC2A9	chr4-9603794-9605234	0.001431389
ZNHIT6	chr1-86502043-86504288	0.001431078
CREB3L2	chr7-137903375-137904292	0.001430859
INVS	chr9-99184176-99185351	0.001430747
ZNHIT6	chr1-86155064-86157137	0.001430721
AL589693.1	chr6-157033266-157035161	0.001430532
ZNHIT6	chr1-86137267-86138070	0.001430461
ZNHIT6	chr1-86394697-86397520	0.001430456
ZNHIT6	chr1-85061604-85063166	0.001430212

INVS	chr9-99134733-99136165	0.00143013
INVS	chr9-100351059-100354511	0.001429922
CDK6	chr7-92178756-92179793	0.001429705
SEC11C	chr18-58657132-58659785	0.001429598
SEC11C	chr18-58649587-58650797	0.001429556
ANXA2R	chr5-41924339-41926666	0.001429325
SEC11C	chr18-59911271-59912211	0.001429227
INVS	chr9-99818440-99830419	0.001429197
ZNHIT6	chr1-85707430-85709077	0.001428885
INVS	chr9-98993126-98994921	0.001428808
INVS	chr9-99118668-99120633	0.001428675
ZNHIT6	chr1-84944422-84946339	0.001428627
SEC11C	chr18-59917228-59918919	0.00142861
INVS	chr9-99103457-99110167	0.001428433
SEC11C	chr18-59904035-59905935	0.001428303
INVS	chr9-99110738-99114055	0.001428272
INVS	chr9-99076303-99077278	0.001428201
INVS	chr9-99220974-99223168	0.001428
INVS	chr9-100598269-100599327	0.001427997
CCDC152	chr5-43601825-43604964	0.001427662
INVS	chr9-99056689-99060162	0.001427418
ZNHIT6	chr1-85200007-85202395	0.0014274
GTDC1	chr2-144606498-144608598	0.00142725
SLC2A9	chr4-9531997-9533061	0.001427239
ANXA2R	chr5-41903000-41905163	0.001427191
INVS	chr9-100410376-100412415	0.001427167
INVS	chr9-99039377-99041860	0.001427156
SEC11C	chr18-58614605-58616490	0.001426929
HEG1	chr3-124886190-124887945	0.001426903
ZNHIT6	chr1-85274754-85279929	0.001426815
CCDC152	chr5-43191557-43194307	0.001426807
CCDC152	chr5-43312175-43314526	0.001426717
INVS	chr9-99659928-99660913	0.001426645
GTDC1	chr2-144507361-144525092	0.00142659
AL589693.1	chr6-157018987-157022702	0.00142659
CCDC152	chr5-43555781-43558090	0.001426317
SEC11C	chr18-58668921-58673020	0.001426219
HEG1	chr3-124729802-124731344	0.001426194
SLC39A10	chr2-196639077-196640300	0.001426192
INVS	chr9-100426272-100428234	0.00142617
INVS	chr9-99595539-99596533	0.001426115
ZNHIT6	chr1-85258569-85260363	0.001425998
SEC11C	chr18-59898635-59902802	0.001425887
AC068587.4	chr8-13131788-13133912	0.001425884
GTDC1	chr2-144659326-144661724	0.001425752
CCDC152	chr5-43396237-43397592	0.001425356
HEG1	chr3-125001947-125002926	0.001425178
AC068587.4	chr8-12764947-12767684	0.001425132
ZNHIT6	chr1-84572542-84575283	0.001424989
CDK6	chr7-92133188-92135822	0.0014249

CCDC152	chr5-43120265-43122814	0.001424896
CCDC152	chr5-43514223-43515990	0.001424832
ZNHIT6	chr1-84862520-84863677	0.001424769
GTDC1	chr2-144458547-144460632	0.001424728
AL589693.1	chr6-156963554-156964439	0.001424726
SLC2A9	chr4-9151790-9154144	0.001424648
ANXA2R	chr5-41868841-41871333	0.001424644
SLC39A10	chr2-196273990-196277504	0.001424484
CCDC152	chr5-43483037-43485870	0.001424385
AL589693.1	chr6-156776270-156783260	0.00142421
SEC11C	chr18-58541148-58543215	0.001424091
ZNHIT6	chr1-84620374-84621470	0.001424086
SLC39A10	chr2-196267034-196269488	0.001423912
SEC11C	chr18-58042784-58045997	0.001423806
HEG1	chr3-124619665-124621061	0.001423509
SEC11C	chr18-59885458-59886778	0.001423363
AL589693.1	chr6-156719405-156721267	0.001423359
SEC11C	chr18-58321778-58322647	0.00142333
AC068587.4	chr8-11491635-11494865	0.001423036
SEC11C	chr18-58534766-58537272	0.001422903
ZNHIT6	chr1-84688618-84691629	0.001422846
CDK6	chr7-91880102-91881582	0.001422811
CDK6	chr7-91945190-91947201	0.001422686
GTDC1	chr2-144456157-144457278	0.001422621
CLMN	chr14-95506887-95508401	0.001422599
CDK6	chr7-91939758-91942680	0.001422506
SLC39A10	chr2-196262164-196263911	0.001422458
GTDC1	chr2-144671432-144672488	0.001422405
AC068587.4	chr8-11464613-11468224	0.001422317
ZNHIT6	chr1-84853558-84855138	0.001422223
CCDC152	chr5-43015290-43021643	0.001422209
HEG1	chr3-125051521-125056800	0.001422068
SEC11C	chr18-59881601-59882984	0.001422005
CCDC152	chr5-43109316-43111439	0.001421898
ZNHIT6	chr1-84692618-84693901	0.001421811
SEC11C	chr18-59357886-59359943	0.001421764
GTDC1	chr2-144703561-144704735	0.001421755
ACADSB	chr10-123007419-123010003	0.001421685
CLMN	chr14-95494258-95497290	0.001421675
AC068587.4	chr8-12752769-12759229	0.001421653
CCDC152	chr5-43006912-43011400	0.001421629
SEC11C	chr18-59139010-59141022	0.001421379
AL589693.1	chr6-156697105-156699501	0.001421096
CCDC152	chr5-43036356-43045777	0.001420973
SEC11C	chr18-58687299-58688967	0.001420962
AC068587.4	chr8-11557713-11559394	0.001420651
HEG1	chr3-123960489-123961778	0.00142055
ACADSB	chr10-123134438-123137836	0.001420506
GTDC1	chr2-144413766-144416097	0.00142041
ABCA1	chr9-105243581-105246125	0.001420229

ACADSB	chr10-122979522-122981586	0.001419973
SLC39A10	chr2-196199781-196202107	0.001419926
CLMN	chr14-95513314-95520921	0.001419818
AL589693.1	chr6-155313520-155315847	0.001419795
ABCA1	chr9-105191234-105192431	0.001419765
CCDC152	chr5-43104171-43106360	0.001419694
SEC11C	chr18-58886582-58888093	0.00141969
CCDC152	chr5-43063602-43069165	0.001419537
SLC39A10	chr2-196208559-196212311	0.001419536
HEG1	chr3-125593730-125596552	0.001419515
ABCA1	chr9-105405104-105406245	0.001419403
SLC39A10	chr2-196181749-196185426	0.001419137
HEG1	chr3-125119291-125122134	0.001419117
CCDC152	chr5-42989132-42996745	0.001419054
SLC39A10	chr2-196259503-196261278	0.001419044
GTDC1	chr2-144682189-144684448	0.001419016
GTDC1	chr2-144700964-144702519	0.001418948
ABCA1	chr9-105693241-105695770	0.001418914
SLC39A10	chr2-196139918-196178981	0.001418387
ABCA1	chr9-105141018-105143442	0.00141827
HEG1	chr3-125519390-125520916	0.001418257
ABCA1	chr9-105447400-105448885	0.001418209
AL589693.1	chr6-156686084-156688501	0.00141807
SLC39A10	chr2-196130604-196139222	0.001418025
ABCA1	chr9-105557598-105558703	0.001417934
GTDC1	chr2-144686457-144687557	0.001417619
CLMN	chr14-95462326-95464968	0.001417607
FGGY	chr1-59506831-59507760	0.001417592
GTDC1	chr2-144379732-144383990	0.001417474
SLC39A10	chr2-196067938-196069604	0.001417349
SLC39A10	chr2-196212820-196214287	0.001417213
HEG1	chr3-125632501-125633797	0.00141713
SEC11C	chr18-58862709-58865155	0.001417079
FGGY	chr1-59813997-59815790	0.00141707
FGGY	chr1-59295928-59297973	0.001417058
HEG1	chr3-125139532-125144820	0.001417047
ACADSB	chr10-123147586-123151866	0.001416993
ACADSB	chr10-122952824-122955346	0.001416827
AL589693.1	chr6-155417393-155419379	0.001416779
ABCA1	chr9-105127463-105129418	0.001416702
SEC11C	chr18-58754287-58756483	0.001416693
FGGY	chr1-59672881-59675234	0.001416421
AC068587.4	chr8-11563692-11565023	0.001416343
AC068587.4	chr8-12664886-12666069	0.001416302
AL589693.1	chr6-156395024-156397760	0.001416135
SLC39A10	chr2-195532104-195537363	0.001415884
SLC39A10	chr2-195655954-195660750	0.00141565
CCDC152	chr5-42948600-42954736	0.001415631
ABCA1	chr9-105114148-105116083	0.001415534
SEC11C	chr18-58764019-58764749	0.001415452

HEG1	chr3-125145740-125149453	0.001415377
CLMN	chr14-95155176-95158967	0.00141527
CLMN	chr14-95522129-95525412	0.001415158
FGGY	chr1-59291901-59293727	0.001415013
FGGY	chr1-59696466-59697814	0.001415005
ACADSB	chr10-122373494-122376105	0.001414987
FGGY	chr1-59692434-59693934	0.001414969
ACADSB	chr10-121974210-121975723	0.001414711
HEG1	chr3-125374184-125376360	0.001414594
CLMN	chr14-94394116-94395426	0.00141459
SLC39A10	chr2-196247367-196250446	0.00141458
ACADSB	chr10-122878781-122880695	0.001414482
ACADSB	chr10-122416377-122417745	0.001414162
ABCA1	chr9-105063499-105065390	0.001414056
ACADSB	chr10-124151842-124153984	0.001414019
CLMN	chr14-95185140-95186584	0.001413763
SLC39A10	chr2-196228988-196231020	0.001413692
GTDC1	chr2-144331053-144333291	0.001413578
HEG1	chr3-125154281-125156451	0.001413347
SLC39A10	chr2-195648450-195651495	0.001413287
RORA	chr15-62389871-62392191	0.001413283
CLMN	chr14-95442311-95443324	0.001413021
FGGY	chr1-59146423-59147513	0.001412961
ACADSB	chr10-123153498-123155516	0.001412935
AP003086.1	chr11-78427375-78428648	0.001412722
CCDC152	chr5-42908081-42909648	0.001412667
FGGY	chr1-58941246-58943291	0.001412661
FGGY	chr1-58967863-58969085	0.001412648
SLC39A10	chr2-195559086-195564930	0.001412644
AC068587.4	chr8-11768146-11771260	0.001412549
RORA	chr15-62066492-62068949	0.001412528
HEG1	chr3-125766158-125767400	0.001412352
CLMN	chr14-94390106-94393165	0.001412347
FGGY	chr1-59019702-59021050	0.001412236
SLC39A10	chr2-195579814-195580736	0.001411803
RORA	chr15-62059118-62061775	0.001411762
RORA	chr15-60578965-60581661	0.001411712
ACADSB	chr10-121926628-121929100	0.001411615
AC068587.4	chr8-11823616-11824762	0.00141145
CLMN	chr14-95313419-95315206	0.00141143
AC068587.4	chr8-11819888-11821159	0.001411394
SLC39A10	chr2-196238015-196243836	0.001411349
RORA	chr15-60582234-60586649	0.001411344
AP003086.1	chr11-78464413-78466099	0.001411303
CLMN	chr14-95533329-95536306	0.001411218
RORA	chr15-61857122-61858161	0.001411158
HEG1	chr3-125356706-125357941	0.001411148
HEG1	chr3-125163661-125165619	0.001411106
SLC39A10	chr2-196231874-196235253	0.001411105
GTDC1	chr2-143151986-143154129	0.001411063

AP003086.1	chr11-78424039-78426016	0.001411045
GTDC1	chr2-143255628-143259946	0.001411036
AC068587.4	chr8-11801546-11809384	0.001411021
ABCA1	chr9-105051680-105053893	0.001411008
FGGY	chr1-58922117-58923142	0.001410882
AC068587.4	chr8-12658544-12659927	0.001410803
CLMN	chr14-95318500-95320536	0.001410693
CCDC152	chr5-41924339-41926666	0.001410468
ACADSB	chr10-124144323-124146125	0.001410389
CLMN	chr14-94180562-94181483	0.001410262
RORA	chr15-60916027-60917515	0.001410052
CCNG2	chr4-77860772-77864142	0.001409938
HEG1	chr3-125310507-125311333	0.001409933
GAB2	chr11-78427375-78428648	0.001409845
ACADSB	chr10-123160287-123161192	0.001409776
GTDC1	chr2-144323989-144325770	0.001409634
CLMN	chr14-94128370-94130490	0.001409562
CLMN	chr14-94173329-94175830	0.001409454
CLMN	chr14-96126010-96127214	0.001409352
GTDC1	chr2-143149867-143150928	0.001409281
GTDC1	chr2-143261429-143263816	0.001409231
AC068587.4	chr8-11844943-11849855	0.00140899
SLC44A1	chr9-105243581-105246125	0.001408954
RORA	chr15-60572556-60575388	0.001408932
CLMN	chr14-96361574-96364643	0.001408903
AP003086.1	chr11-78573579-78575769	0.001408724
SLC44A1	chr9-105191234-105192431	0.001408495
RORA	chr15-60588971-60593566	0.001408479
GAB2	chr11-78464413-78466099	0.001408427
CCDC152	chr5-41903000-41905163	0.001408362
FGGY	chr1-58738666-58740445	0.001408244
CLMN	chr14-96390783-96392912	0.001408204
GAB2	chr11-78424039-78426016	0.001408171
SLC44A1	chr9-105405104-105406245	0.001408134
HEG1	chr3-125915003-125917475	0.001408028
RORA	chr15-60843908-60845557	0.001407957
HEG1	chr3-126081537-126084839	0.001407796
FGGY	chr1-58754628-58756004	0.001407716
SLC44A1	chr9-105693241-105695770	0.00140765
FGGY	chr1-58902954-58904554	0.001407545
ZBTB20-AS5	chr3-113838330-113839423	0.001407511
ACADSB	chr10-123240854-123242451	0.001407467
AP003086.1	chr11-79440343-79442139	0.001407429
GTDC1	chr2-143139785-143142979	0.001407347
GTDC1	chr2-144183459-144184399	0.00140731
GTDC1	chr2-144237655-144238696	0.001407214
SLC44A1	chr9-105141018-105143442	0.001407012
SLC44A1	chr9-105447400-105448885	0.00140695
SLC44A1	chr9-105557598-105558703	0.001406677
AP003086.1	chr11-78415393-78421496	0.001406538

HEG1	chr3-125989972-125991435	0.001406459
ZBTB20-AS5	chr3-113947226-113949023	0.001406336
CLMN	chr14-96500966-96503756	0.001406317
LEF1	chr4-108432271-108434572	0.001406254
ABCA1	chr9-105006418-105007859	0.001406075
GAB2	chr11-78573579-78575769	0.001405855
CCDC152	chr5-41868841-41871333	0.001405848
FGGY	chr1-58698763-58700752	0.001405798
RORA	chr15-60839646-60842738	0.001405504
CCNG2	chr4-77852357-77855525	0.001405489
SLC44A1	chr9-105127463-105129418	0.001405456
RORA	chr15-60649315-60650653	0.001405399
FGGY	chr1-58777205-58787295	0.001405356
ABCA1	chr9-104774890-104776213	0.001405317
LEF1	chr4-108359175-108360813	0.001405299
ZBTB20-AS5	chr3-113830038-113831239	0.001405176
JUN	chr1-59506831-59507760	0.001405089
ACADSB	chr10-123994165-123996391	0.001404864
RORA	chr15-60567781-60571710	0.001404628
JUN	chr1-59813997-59815790	0.001404573
GAB2	chr11-79440343-79442139	0.001404562
JUN	chr1-59295928-59297973	0.00140456
FGGY	chr1-58882862-58886476	0.001404488
ACADSB	chr10-124091527-124094853	0.00140442
SLC44A1	chr9-105114148-105116083	0.001404298
ABCA1	chr9-104763458-104765896	0.001404293
RORA	chr15-60719934-60721613	0.001404253
LEF1	chr4-108466192-108467949	0.001404167
JUN	chr1-59672881-59675234	0.001403928
AC068587.4	chr8-11856900-11864268	0.001403795
GAB2	chr11-78415393-78421496	0.001403674
FGGY	chr1-58812506-58817227	0.001403672
RORA	chr15-60392524-60399879	0.00140367
ABCA1	chr9-104868527-104869815	0.001403639
RORA	chr15-60407420-60409172	0.001403436
LEF1	chr4-108080587-108081774	0.001403405
AP003086.1	chr11-78187199-78190272	0.001402906
RORA	chr15-60127075-60128411	0.001402887
AP003086.1	chr11-78138594-78140674	0.001402854
SLC44A1	chr9-105063499-105065390	0.001402832
LEF1	chr4-108106818-108108626	0.001402734
ZBTB20-AS5	chr3-114055718-114057746	0.001402616
JUN	chr1-59291901-59293727	0.001402532
JUN	chr1-59696466-59697814	0.001402525
LEF1	chr4-108070695-108073211	0.001402496
JUN	chr1-59692434-59693934	0.00140249
RORA	chr15-60477913-60480751	0.001401936
ABCA1	chr9-104746965-104749120	0.001401831
LEF1	chr4-108355193-108356563	0.001401665
FGGY	chr1-58618150-58619013	0.001401619

AP003086.1	chr11-78349082-78351590	0.00140157
AP003086.1	chr11-78078471-78081906	0.001401526
ACADSB	chr10-123997103-123998889	0.001401349
RORA	chr15-60563396-60564425	0.001401342
LEF1	chr4-108048975-108052308	0.001401339
AP003086.1	chr11-78201106-78203903	0.001401211
AP003086.1	chr11-77819055-77822037	0.00140115
LEF1	chr4-107718368-107722031	0.001401001
AP003086.1	chr11-77636560-77638427	0.001400989
LEF1	chr4-108035746-108036993	0.001400918
LEF1	chr4-108043066-108046490	0.001400907
CCNG2	chr4-77826329-77828169	0.00140075
LEF1	chr4-108619014-108622283	0.001400726
ABCA1	chr9-104989458-104994114	0.001400653
ZBTB20-AS5	chr3-113748705-113749729	0.001400646
RORA	chr15-60507581-60508632	0.001400617
ZBTB20-AS5	chr3-114290592-114292019	0.001400592
AP003086.1	chr11-77993779-77995755	0.001400511
JUN	chr1-59146423-59147513	0.001400498
LEF1	chr4-108029046-108030807	0.001400454
AP003086.1	chr11-78045730-78046648	0.001400443
ZBTB20-AS5	chr3-114231318-114233425	0.001400326
JUN	chr1-58941246-58943291	0.001400201
JUN	chr1-58967863-58969085	0.001400188
LEF1	chr4-108112787-108117788	0.001400173
ABCA1	chr9-104872858-104873815	0.001400078
GAB2	chr11-78187199-78190272	0.001400049
GAB2	chr11-78138594-78140674	0.001399999
ABCA1	chr9-104092979-104095052	0.001399867
SLC44A1	chr9-105051680-105053893	0.001399808
JUN	chr1-59019702-59021050	0.001399779
AC004889.1	chr7-144812385-144813490	0.001399672
LINC01135	chr1-59506831-59507760	0.001399569
AP003086.1	chr11-77589108-77590709	0.00139938
LEF1	chr4-107988917-107991215	0.00139912
AP003086.1	chr11-78289498-78290975	0.001399081
LINC01135	chr1-59813997-59815790	0.001399055
AC068587.4	chr8-11901943-11903034	0.001399054
LINC01135	chr1-59295928-59297973	0.001399042
ZBTB20-AS5	chr3-114152352-114153233	0.001398972
AC004889.1	chr7-143345200-143346347	0.001398967
AC004889.1	chr7-143901245-143902861	0.001398951
ACADSB	chr10-124063595-124065153	0.001398936
LEF1	chr4-107824125-107825881	0.0013989
ZBTB20-AS5	chr3-114307211-114309487	0.001398874
AP003086.1	chr11-77408341-77412628	0.001398871
CCNG2	chr4-77193181-77194619	0.001398859
AP003086.1	chr11-78291691-78292899	0.001398767
GAB2	chr11-78349082-78351590	0.001398716
AC004889.1	chr7-143361326-143363404	0.001398672

GAB2	chr11-78078471-78081906	0.001398671
ZBTB20-AS5	chr3-114221139-114223781	0.00139863
CCNG2	chr4-77154035-77159106	0.001398447
JUN	chr1-58922117-58923142	0.001398437
LINC01135	chr1-59672881-59675234	0.001398413
GAB2	chr11-78201106-78203903	0.001398356
FGGY	chr1-58544999-58547489	0.001398304
GAB2	chr11-77819055-77822037	0.001398297
AC004889.1	chr7-144817944-144819177	0.001398254
GAB2	chr11-77636560-77638427	0.001398136
CCNG2	chr4-77817292-77823287	0.001398131
LEF1	chr4-108649363-108651838	0.001398093
CCNG2	chr4-77800655-77801983	0.001398049
ACADSB	chr10-124035388-124036715	0.00139787
AC068587.4	chr8-11864871-11869863	0.00139783
LEF1	chr4-107930659-107932996	0.001397687
LEF1	chr4-108170508-108173850	0.001397676
GAB2	chr11-77993779-77995755	0.001397658
GAB2	chr11-78045730-78046648	0.001397591
ZBTB20-AS5	chr3-114212642-114216815	0.001397589
LEF1	chr4-107837233-107839598	0.001397451
AP003086.1	chr11-77472848-77476339	0.001397271
LEF1	chr4-108159763-108161575	0.001397145
ABCA1	chr9-104967853-104969071	0.00139712
ABCA1	chr9-104926180-104928813	0.001397111
LINC01135	chr1-59291901-59293727	0.001397023
LINC01135	chr1-59696466-59697814	0.001397017
LINC01135	chr1-59692434-59693934	0.001396981
AP003086.1	chr11-77448298-77449687	0.00139666
ACADSB	chr10-124037238-124038510	0.001396574
AC004889.1	chr7-143836128-143837663	0.001396531
GAB2	chr11-77589108-77590709	0.00139653
CCNG2	chr4-76896897-76898669	0.001396378
ZBTB20-AS5	chr3-115099905-115100777	0.001396376
ZBTB20-AS5	chr3-113743235-113748148	0.001396321
CCNG2	chr4-76948283-76951399	0.001396306
AC004889.1	chr7-143374386-143376826	0.001396284
GAB2	chr11-78289498-78290975	0.001396232
AC004889.1	chr7-143286425-143289855	0.001396172
CCNG2	chr4-77125880-77128423	0.001396082
GAB2	chr11-77408341-77412628	0.001396022
GAB2	chr11-78291691-78292899	0.001395918
LEF1	chr4-108165117-108169957	0.001395874
AC004889.1	chr7-144834071-144836850	0.001395864
JUN	chr1-58738666-58740445	0.001395823
SAMSN1	chr21-15206316-15207366	0.001395596
AC068587.4	chr8-11896617-11898849	0.001395507
JUN	chr1-58754628-58756004	0.0013953
SAMSN1	chr21-15060753-15066847	0.001395201
JUN	chr1-58902954-58904554	0.00139513

LINC01135	chr1-59146423-59147513	0.001394997
CCNG2	chr4-76304851-76307490	0.001394969
SLC44A1	chr9-105006418-105007859	0.001394914
ZBTB20-AS5	chr3-115145540-115148818	0.00139491
LINC01135	chr1-58941246-58943291	0.001394701
LINC01135	chr1-58967863-58969085	0.001394688
CCNG2	chr4-76988113-76990762	0.001394468
GAB2	chr11-77472848-77476339	0.001394425
AC004889.1	chr7-143406826-143409775	0.001394344
LINC01135	chr1-59019702-59021050	0.001394281
AC004889.1	chr7-143378177-143387996	0.001394219
SLC44A1	chr9-104774890-104776213	0.001394161
AC004889.1	chr7-144841896-144843091	0.00139407
GAB2	chr11-77448298-77449687	0.001393815
AC068587.4	chr8-11876981-11879161	0.001393813
ZBTB20-AS5	chr3-113736386-113737260	0.00139376
JUN	chr1-58698763-58700752	0.001393399
CCNG2	chr4-76211795-76214920	0.00139337
PVT1	chr8-127975230-127978753	0.00139334
AC068587.4	chr8-11885662-11889148	0.00139321
CDK8	chr13-27293011-27294663	0.001393207
SLC44A1	chr9-104763458-104765896	0.001393146
PVT1	chr8-127915623-127918477	0.00139311
CCNG2	chr4-77072143-77078187	0.001393065
JUN	chr1-58777205-58787295	0.00139296
SAMSN1	chr21-15221701-15223221	0.001392948
LINC01135	chr1-58922117-58923142	0.001392944
CDK8	chr13-27270171-27271931	0.001392828
ZBTB20-AS5	chr3-113694935-113698140	0.001392658
CCNG2	chr4-76197867-76201119	0.001392567
SLC44A1	chr9-104868527-104869815	0.001392497
CCNG2	chr4-76993671-76995086	0.001392206
CCNG2	chr4-76147516-76149266	0.001392167
JUN	chr1-58882862-58886476	0.0013921
SAMSN1	chr21-14762355-14763805	0.00139167
ZBTB20-AS5	chr3-113625303-113627328	0.001391563
AC004889.1	chr7-143279039-143280681	0.001391482
CCNG2	chr4-77007091-77008357	0.001391443
JUN	chr1-58812506-58817227	0.001391292
PVT1	chr8-127979458-127983137	0.001391154
CCNG2	chr4-76089544-76090869	0.001390918
SLC44A1	chr9-104746965-104749120	0.001390704
CDK8	chr13-27423247-27428812	0.001390697
LINC01135	chr1-58738666-58740445	0.00139034
CDK8	chr13-27256573-27261677	0.001389848
LINC01135	chr1-58754628-58756004	0.001389819
SAMSN1	chr21-14541206-14544743	0.001389766
LINC01135	chr1-58902954-58904554	0.00138965
SLC44A1	chr9-104989458-104994114	0.001389536
ZBTB20-AS5	chr3-113606625-113607586	0.001389524

SAMSN1	chr21-14480526-14483734	0.001389403
SAMSN1	chr21-14545423-14546958	0.001389307
SAMSN1	chr21-15513879-15514893	0.001389262
JUN	chr1-58618150-58619013	0.001389256
PVT1	chr8-127793559-127796604	0.001389077
SLC44A1	chr9-104872858-104873815	0.001388962
PVT1	chr8-127991697-127994308	0.001388911
SLC44A1	chr9-104092979-104095052	0.001388754
SAMSN1	chr21-14382320-14384431	0.001388519
SAMSN1	chr21-14533004-14535760	0.00138848
SAMSN1	chr21-14520205-14521182	0.001388432
CCNG2	chr4-76028131-76029686	0.001388118
PVT1	chr8-128047943-128051417	0.001388039
PVT1	chr8-128041732-128043694	0.001388032
LINC01135	chr1-58698763-58700752	0.001387925
PVT1	chr8-128167370-128168791	0.00138751
LINC01135	chr1-58777205-58787295	0.001387488
AC004889.1	chr7-143262646-143265387	0.001387285
SAMSN1	chr21-14522666-14523962	0.001387144
ZBTB20-AS5	chr3-113531329-113535172	0.00138712
SAMSN1	chr21-14526402-14528971	0.001387117
SAMSN1	chr21-14685236-14688085	0.001387073
SAMSN1	chr21-14547822-14550715	0.001386946
ZBTB20-AS5	chr3-113275734-113276902	0.001386937
CDK8	chr13-27441715-27443286	0.001386715
SAMSN1	chr21-15681973-15683480	0.001386655
LINC01135	chr1-58882862-58886476	0.001386631
SAMSN1	chr21-15729208-15734358	0.001386212
SLC44A1	chr9-104967853-104969071	0.001386031
SLC44A1	chr9-104926180-104928813	0.00138602
JUN	chr1-58544999-58547489	0.00138597
CDK8	chr13-27250494-27254086	0.001385967
ZBTB20-AS5	chr3-113513975-113516789	0.001385907
SAMSN1	chr21-15686132-15687272	0.001385889
LINC01135	chr1-58812506-58817227	0.001385826
SAMSN1	chr21-14215783-14216807	0.001385819
PVT1	chr8-128173495-128174572	0.001385656
AC004889.1	chr7-143250414-143252460	0.001385136
AC004889.1	chr7-143239294-143241177	0.001384725
CCNG2	chr4-75989840-75991600	0.001384566
SAMSN1	chr21-14591346-14592980	0.001384476
AC004889.1	chr7-143222545-143223461	0.001384443
SAMSN1	chr21-14675521-14677202	0.001384213
LINC01135	chr1-58618150-58619013	0.001383798
PVT1	chr8-128204843-128206525	0.001383143
AC004889.1	chr7-143214808-143215586	0.001383096
CDK8	chr13-27446647-27447811	0.001383017
CDK8	chr13-27167871-27173317	0.001382979
SAMSN1	chr21-14027010-14027895	0.001382879
PVT1	chr8-127759275-127761304	0.001382231

PVT1	chr8-128539987-128541640	0.001381731
PVT1	chr8-128238876-128240802	0.001381445
CDK8	chr13-27130958-27131803	0.001381254
PVT1	chr8-128250289-128252175	0.001381254
AC004889.1	chr7-143205581-143206469	0.001381133
PVT1	chr8-128542168-128544216	0.001381112
LINC01135	chr1-58544999-58547489	0.001380525
CDK8	chr13-27448827-27452079	0.001380282
AC004889.1	chr7-143125867-143126594	0.001380252
HHAT	chr1-211513876-211516126	0.001379768
CDK8	chr13-26982889-26983692	0.001379663
ACYP2	chr2-54330032-54331787	0.001378722
HHAT	chr1-211228588-211229865	0.001378509
PVT1	chr8-128659133-128660265	0.001378379
ACYP2	chr2-54556476-54563464	0.001378244
HHAT	chr1-211133494-211134589	0.001377972
HHAT	chr1-211257972-211261341	0.001377946
ACYP2	chr2-54595391-54598126	0.001377871
HHAT	chr1-210328278-210329861	0.001377522
HHAT	chr1-210332077-210333358	0.001377516
LEF1-AS1	chr4-108432271-108434572	0.001377408
ACYP2	chr2-54601227-54607029	0.00137735
ACYP2	chr2-54587684-54591128	0.001377274
ACYP2	chr2-54567984-54576108	0.001377052
HHAT	chr1-209827186-209828713	0.00137696
ACYP2	chr2-54114805-54116478	0.001376897
CDK8	chr13-26785099-26786384	0.001376759
ACYP2	chr2-54579008-54582564	0.001376649
HHAT	chr1-211416134-211417445	0.001376606
LEF1-AS1	chr4-108359175-108360813	0.001376473
HHAT	chr1-211325184-211330483	0.001375755
ACYP2	chr2-54672728-54674120	0.001375719
MLLT3	chr9-21812907-21814343	0.001375587
PVT1	chr8-127733640-127740565	0.001375469
LEF1-AS1	chr4-108466192-108467949	0.001375364
ACYP2	chr2-55019243-55020826	0.00137466
PVT1	chr8-127168754-127171296	0.001374637
LEF1-AS1	chr4-108080587-108081774	0.001374617
HHAT	chr1-209805461-209806831	0.001374276
ACYP2	chr2-55009477-55011366	0.001374177
ACYP2	chr2-54708451-54709999	0.001374098
LEF1-AS1	chr4-108106818-108108626	0.00137396
ACYP2	chr2-55047358-55052732	0.00137384
BMERB1	chr16-15954234-15957729	0.001373788
LEF1-AS1	chr4-108070695-108073211	0.001373726
ZNF532	chr18-58657132-58659785	0.001373661
ZNF532	chr18-58649587-58650797	0.00137362
ACYP2	chr2-54722605-54725620	0.001373588
BMERB1	chr16-15948464-15953107	0.001373475
ZNF532	chr18-59911271-59912211	0.001373303

HHAT	chr1-211381834-211383777	0.001373159
HHAT	chr1-211334961-211336579	0.001373013
LEF1-AS1	chr4-108355193-108356563	0.001372914
PVT1	chr8-127196313-127197691	0.001372911
MLLT3	chr9-21801768-21803900	0.001372771
ZNF532	chr18-59917228-59918919	0.001372711
ACYP2	chr2-53993798-53995544	0.001372618
LEF1-AS1	chr4-108048975-108052308	0.001372594
BMERB1	chr16-15958584-15962333	0.001372542
ZNF532	chr18-59904035-59905935	0.001372416
LEF1-AS1	chr4-107718368-107722031	0.001372262
CSGALNACT1	chr8-19127808-19128782	0.001372195
LEF1-AS1	chr4-108035746-108036993	0.001372181
CDK8	chr13-26253397-26255476	0.001372175
LEF1-AS1	chr4-108043066-108046490	0.00137217
LEF1-AS1	chr4-108619014-108622283	0.001371993
ACYP2	chr2-55150562-55154819	0.001371976
MCTP1	chr5-95807745-95811026	0.00137192
ACYP2	chr2-55111485-55113413	0.001371821
CSGALNACT1	chr8-18747388-18748557	0.001371786
LEF1-AS1	chr4-108029046-108030807	0.001371726
HHAT	chr1-211352842-211354316	0.001371721
BMERB1	chr16-15887777-15889424	0.001371657
CSGALNACT1	chr8-19013249-19014720	0.001371512
PVT1	chr8-127386530-127387631	0.00137151
LEF1-AS1	chr4-108112787-108117788	0.001371451
PVT1	chr8-127209617-127211171	0.00137113
ZNF532	chr18-58614605-58616490	0.001371095
MCTP1	chr5-95780592-95781687	0.001371095
CSGALNACT1	chr8-19459642-19460988	0.001370946
BMERB1	chr16-16074371-16076923	0.001370925
CSGALNACT1	chr8-18957115-18958075	0.00137082
MTERF1	chr7-92717657-92719209	0.001370492
LEF1-AS1	chr4-107988917-107991215	0.00137042
ZNF532	chr18-58668921-58673020	0.001370414
LEF1-AS1	chr4-107824125-107825881	0.001370205
BMERB1	chr16-16232201-16232800	0.001370147
MTERF1	chr7-92730495-92733049	0.001370118
ZNF532	chr18-59898635-59902802	0.001370093
ACYP2	chr2-55133121-55135088	0.001370024
ACYP2	chr2-55136234-55138039	0.001370024
BMERB1	chr16-15671408-15674149	0.00136963
LEF1-AS1	chr4-108649363-108651838	0.001369415
MTERF1	chr7-92446734-92449171	0.00136934
MCTP1	chr5-95816783-95826314	0.001369286
MTERF1	chr7-92684848-92685913	0.001369236
MTERF1	chr7-92809395-92810479	0.001369063
LEF1-AS1	chr4-107930659-107932996	0.001369017
HHAT	chr1-209782958-209785634	0.001369014
LEF1-AS1	chr4-108170508-108173850	0.001369006

BMERB1	chr16-15649344-15651620	0.001368803
LEF1-AS1	chr4-107837233-107839598	0.001368785
MTERF1	chr7-92831942-92837624	0.00136877
BMERB1	chr16-15657995-15659894	0.001368677
BMERB1	chr16-15641935-15644562	0.001368541
LEF1-AS1	chr4-108159763-108161575	0.001368484
ZNF532	chr18-58541148-58543215	0.001368367
MTERF1	chr7-92527287-92530253	0.001368165
ZNF532	chr18-58042784-58045997	0.001368095
MCTP1	chr5-95759606-95762192	0.001367982
MTERF1	chr7-92423519-92426024	0.001367719
ZNF532	chr18-59885458-59886778	0.001367668
ZNF532	chr18-58321778-58322647	0.001367637
ACYP2	chr2-53967227-53971896	0.001367432
MLLT3	chr9-21789378-21790131	0.001367411
CSGALNACT1	chr8-19473168-19473926	0.001367364
LEF1-AS1	chr4-108165117-108169957	0.001367241
ZNF532	chr18-58534766-58537272	0.001367226
ZNF532	chr18-57665645-57667487	0.001367207
MTERF1	chr7-92633100-92636028	0.001367036
CDK8	chr13-26221214-26223391	0.00136703
EXOC6B	chr2-72825220-72826381	0.001366698
ZNF532	chr18-59881601-59882984	0.001366364
MTERF1	chr7-92588510-92591207	0.001366213
BMERB1	chr16-15588766-15593721	0.001366164
ZNF532	chr18-59357886-59359943	0.001366133
ZNF532	chr18-59139010-59141022	0.001365763
EXOC6B	chr2-74025450-74027398	0.001365638
MTERF1	chr7-92620902-92621943	0.001365627
ZNF532	chr18-58687299-58688967	0.001365362
EXOC6B	chr2-72915865-72919046	0.001365007
EXOC6B	chr2-73111453-73113871	0.001364906
MCTP1	chr5-95730676-95732517	0.001364888
EXOC6B	chr2-73068990-73072784	0.001364553
MCTP1	chr5-95834011-95836840	0.001364346
MCTP1	chr5-95957625-95963190	0.001364209
EXOC6B	chr2-73032845-73035444	0.001364205
ZNF532	chr18-58886582-58888093	0.001364141
EXOC6B	chr2-73175144-73178227	0.001363815
HHAT	chr1-209626220-209628105	0.001363771
ACYP2	chr2-53785515-53788294	0.001363516
MCTP1	chr5-95645792-95648148	0.001363311
EXOC6B	chr2-73969318-73972078	0.001363288
ATP8B1	chr18-58657132-58659785	0.001363205
EXOC6B	chr2-74000098-74003475	0.001363198
ATP8B1	chr18-58649587-58650797	0.001363165
CSGALNACT1	chr8-20200118-20202755	0.001363129
MTERF1	chr7-92244785-92247266	0.001363105
CSGALNACT1	chr8-19474658-19476389	0.001362987
MCTP1	chr5-95631726-95632862	0.001362906

CDK8	chr13-26184625-26187972	0.001362897
EXOC6B	chr2-73948370-73949403	0.00136279
EXOC6B	chr2-73978532-73988623	0.00136279
HHAT	chr1-209767396-209770408	0.001362592
HHAT	chr1-209648740-209652007	0.001362567
ZNF43	chr19-21474136-21475429	0.001362528
MCTP1	chr5-95869013-95871288	0.00136249
CSGALNACT1	chr8-19754589-19759001	0.001362472
CSGALNACT1	chr8-20196412-20199000	0.001362368
CSGALNACT1	chr8-20302469-20304475	0.001362249
MCTP1	chr5-95619500-95622982	0.001362244
ACYP2	chr2-53767248-53768666	0.001362062
CSGALNACT1	chr8-19816289-19818695	0.00136175
MLLT3	chr9-21576150-21577236	0.001361726
ZNF532	chr18-58862709-58865155	0.00136163
CSGALNACT1	chr8-20492681-20493967	0.001361518
ZNF43	chr19-21482692-21484308	0.001361368
CSGALNACT1	chr8-19695251-19700780	0.001361358
ZNF532	chr18-58754287-58756483	0.001361261
BMERB1	chr16-15154098-15155549	0.00136119
CSGALNACT1	chr8-20435038-20437625	0.001361176
HHAT	chr1-209656835-209658090	0.001361099
EXOC6B	chr2-73212375-73214910	0.001360894
ATP8B1	chr18-58614605-58616490	0.00136066
BMERB1	chr16-14282295-14283335	0.001360488
CDK8	chr13-26049636-26052637	0.001360476
MCTP1	chr5-95554067-95556107	0.00136036
EXOC6B	chr2-73924436-73929750	0.001360353
CSGALNACT1	chr8-20188819-20189949	0.001360144
CSGALNACT1	chr8-19496079-19498471	0.001360109
ZNF532	chr18-58764019-58764749	0.001360068
ZNF43	chr19-21396377-21397964	0.001360003
ATP8B1	chr18-58668921-58673020	0.001359983
MCTP1	chr5-95840649-95844429	0.001359884
CSGALNACT1	chr8-19510655-19512210	0.001359867
MCTP1	chr5-95860862-95862229	0.001359743
CSGALNACT1	chr8-19843423-19845483	0.001359606
CDK8	chr13-26011931-26013182	0.001359116
HHAT	chr1-209659254-209661701	0.00135882
MCTP1	chr5-95858443-95859573	0.001358267
CSGALNACT1	chr8-20186281-20187696	0.0013581
ATP8B1	chr18-58541148-58543215	0.001357953
CSGALNACT1	chr8-19886295-19887284	0.001357871
MCTP1	chr5-95283693-95285453	0.001357785
MLLT3	chr9-21558717-21560475	0.00135769
ATP8B1	chr18-58042784-58045997	0.001357682
CDK8	chr13-25901452-25903543	0.001357633
LYRM4	chr6-4774531-4778457	0.001357518
LINC02273	chr4-152115338-152116623	0.001357481
HHAT	chr1-209755423-209757593	0.001357446

LYRM4	chr6-5506174-5507953	0.001357347
MTERF1	chr7-92178756-92179793	0.001357311
ATP8B1	chr18-58321778-58322647	0.001357229
EXOC6B	chr2-73232342-73235939	0.001357226
LYRM4	chr6-5259889-5262303	0.001357203
AGBL3	chr7-134645480-134648042	0.001357157
ZNF43	chr19-21505054-21507037	0.001357126
LYRM4	chr6-5003084-5004794	0.001357105
LINC02273	chr4-152097695-152104024	0.001356896
ATP8B1	chr18-58534766-58537272	0.001356821
LYRM4	chr6-4407520-4408671	0.00135681
ATP8B1	chr18-57665645-57667487	0.001356801
EXOC6B	chr2-73876942-73878124	0.001356774
AGBL3	chr7-135661435-135663457	0.001356737
MCTP1	chr5-94617787-94619538	0.00135669
LINC02273	chr4-152458585-152459689	0.001356579
LYRM4	chr6-5173721-5176044	0.001356516
AGBL3	chr7-135976215-135978062	0.001356498
MCTP1	chr5-94432362-94434176	0.001356498
LYRM4	chr6-5084306-5086538	0.00135645
HHAT	chr1-209672763-209676241	0.001356353
AGBL3	chr7-134458052-134459898	0.001356173
MCTP1	chr5-95261285-95262309	0.001356002
LYRM4	chr6-5517672-5519514	0.001355928
AGBL3	chr7-135146610-135149231	0.001355891
MCTP1	chr5-95081164-95082488	0.001355888
AGBL3	chr7-135979891-135982660	0.00135586
AGBL3	chr7-134669315-134670505	0.001355766
CDK8	chr13-25872761-25875613	0.001355622
MLLT3	chr9-21443789-21445084	0.001355611
LINC02273	chr4-152533729-152538389	0.001355534
LYRM4	chr6-4134024-4136861	0.001355437
HHAT	chr1-209747318-209749010	0.00135538
AGBL3	chr7-135557297-135559248	0.00135531
ZNF43	chr19-21358422-21360177	0.001355305
LINC02273	chr4-152553725-152555232	0.001355261
LINC02273	chr4-152635716-152636615	0.001355242
AGBL3	chr7-135150113-135152534	0.001355235
BMERB1	chr16-14284752-14287973	0.001354986
ATP8B1	chr18-58687299-58688967	0.001354969
AGBL3	chr7-134995044-134996448	0.001354929
BMERB1	chr16-15093786-15095160	0.001354865
CDK8	chr13-25285318-25289340	0.001354724
ATP8B1	chr18-57649438-57650530	0.001354699
EXOC6B	chr2-73268572-73270134	0.001354402
XKR6	chr8-11491635-11494865	0.001354381
LINC02273	chr4-152079528-152080495	0.001354362
MLLT3	chr9-21381426-21382425	0.00135414
AGBL3	chr7-134710543-134711844	0.001354087
ATP8B1	chr18-57580785-57581901	0.001354081

LINC02273	chr4-152643044-152644525	0.00135397
MCTP1	chr5-94110725-94112475	0.00135383
AGBL3	chr7-134985876-134987511	0.001353822
CDK8	chr13-25846703-25848830	0.001353822
EXOC6B	chr2-73828236-73831423	0.001353769
ATP8B1	chr18-58886582-58888093	0.001353757
XKR6	chr8-11464613-11468224	0.001353696
CDK8	chr13-25300626-25303008	0.001353419
ATP8B1	chr18-57585777-57587358	0.001353329
EXOC6B	chr2-73283336-73285655	0.001353213
LYRM4	chr6-5657472-5658577	0.001353173
EXOC6B	chr2-73290167-73294212	0.001353157
EXOC6B	chr2-73384898-73387116	0.001353114
AGBL3	chr7-135160068-135162525	0.001353076
ATP8B1	chr18-56650111-56653548	0.001353074
ATP8B1	chr18-57629496-57631445	0.001352786
AGBL3	chr7-135508764-135511220	0.001352757
MTERF1	chr7-92133188-92135822	0.001352751
MLLT3	chr9-19925872-19926618	0.001352665
EXOC6B	chr2-73736535-73738408	0.001352634
EXOC6B	chr2-73779123-73780829	0.001352477
ATP8B1	chr18-57621014-57623153	0.001352372
AGBL3	chr7-134432047-134433561	0.001352166
XKR6	chr8-11557713-11559394	0.00135211
MLLT3	chr9-19464466-19465433	0.001351927
ZNF43	chr19-21568797-21569950	0.001351883
MLLT3	chr9-21334390-21335909	0.001351767
LYRM4	chr6-5698640-5699787	0.001351673
LYRM4	chr6-5723611-5725083	0.001351586
MTERF1	chr7-91164836-91165966	0.00135133
ATP8B1	chr18-58862709-58865155	0.001351265
AGBL3	chr7-135166233-135172138	0.001351051
XKR6	chr8-11453558-11459742	0.00135099
ATP8B1	chr18-58754287-58756483	0.001350898
ZNF43	chr19-21327610-21329995	0.001350888
AGBL3	chr7-135206822-135212411	0.001350861
LINC02273	chr4-151864848-151867001	0.001350813
MTERF1	chr7-91880102-91881582	0.001350766
MTERF1	chr7-91945190-91947201	0.001350648
MLLT3	chr9-20209265-20210451	0.001350579
LYRM4	chr6-5664933-5666046	0.001350569
MTERF1	chr7-91325719-91327942	0.001350553
MTERF1	chr7-91939758-91942680	0.001350477
LYRM4	chr6-5670806-5673398	0.001350425
LINC02273	chr4-152662648-152664430	0.001350377
ATP8B1	chr18-56637327-56639632	0.001350283
MTERF1	chr7-91263377-91265920	0.00135023
MTERF1	chr7-91300069-91300979	0.00135008
ATP8B1	chr18-58764019-58764749	0.001349715
LYRM4	chr6-5667470-5668476	0.001349607

BMERB1	chr16-14300598-14303939	0.001349606
CXXC1	chr18-51211210-51212292	0.001349475
LYRM4	chr6-5831043-5833154	0.001349449
BMERB1	chr16-15053960-15056931	0.001349234
ZNF43	chr19-21140749-21142790	0.001348392
MLLT3	chr9-21118839-21119756	0.001348347
MLLT3	chr9-20640146-20641161	0.001348295
XKR6	chr8-11283548-11285617	0.00134811
XKR6	chr8-11563692-11565023	0.001348009
LINC02273	chr4-151760239-151761844	0.001347784
CACNA2D3	chr3-52982789-52984483	0.001347649
ZNF43	chr19-21081794-21082921	0.001347597
ZNF43	chr19-21586140-21587530	0.001347526
MLLT3	chr9-20619709-20625201	0.001347402
MLLT3	chr9-20242172-20244870	0.001347402
MLLT3	chr9-20683095-20685660	0.001347346
ZNF43	chr19-21019966-21022148	0.001347075
GLUL	chr1-182603083-182605071	0.001346926
GLUL	chr1-183363525-183364552	0.001346593
AGBL3	chr7-134315834-134317668	0.001346582
GLUL	chr1-183469779-183473650	0.001346563
CXXC1	chr18-51194167-51199488	0.001346425
GLUL	chr1-183588716-183591500	0.001346354
XKR6	chr8-11199037-11202359	0.001346238
GLUL	chr1-182614245-182617027	0.001346131
LYRM4	chr6-5850315-5852195	0.001346092
CACNA2D3	chr3-53018005-53019087	0.001346081
LINC02273	chr4-151407399-151410317	0.001346076
CCDC138	chr2-108448037-108450413	0.001346014
CXXC1	chr18-50877656-50880580	0.001346008
BMERB1	chr16-14308485-14309940	0.001345954
GLUL	chr1-183583086-183584127	0.001345852
GLUL	chr1-183567395-183569079	0.001345825
GLUL	chr1-183592874-183594054	0.001345822
MLLT3	chr9-20321417-20322391	0.001345797
BMERB1	chr16-15013636-15015076	0.001345663
ZNF43	chr19-20999441-21000545	0.001345657
CCDC138	chr2-108533324-108535557	0.001345551
MLLT3	chr9-20314541-20316212	0.001345467
GLUL	chr1-183579873-183581097	0.001345437
GLUL	chr1-183022253-183024464	0.001345369
MLLT3	chr9-20989798-20991411	0.00134532
MLLT3	chr9-21095860-21097209	0.001345257
LINC02273	chr4-151097775-151101396	0.001345241
CXXC1	chr18-50853785-50855233	0.001345152
GLUL	chr1-182601061-182602259	0.00134512
XKR6	chr8-11014900-11017401	0.001345003
LINC02273	chr4-152678393-152681141	0.001344966
CXXC1	chr18-50967221-50968897	0.001344945
XKR6	chr8-11768146-11771260	0.001344398

ZNF43	chr19-21593423-21595308	0.001344377
BMERB1	chr16-14354325-14355771	0.001344256
LINC02273	chr4-151014249-151017068	0.001344241
GLUL	chr1-182788896-182790343	0.001344167
MLLT3	chr9-21029814-21032260	0.001344151
BMERB1	chr16-14974274-14976270	0.001344116
BMERB1	chr16-14628770-14634739	0.001343792
SMARCAL1	chr2-217610059-217611367	0.001343771
GLUL	chr1-182838465-182842164	0.001343696
CXXC1	chr18-51190978-51191834	0.00134367
ZNF43	chr19-20922694-20924249	0.001343451
XKR6	chr8-11823616-11824762	0.001343353
XKR6	chr8-11819888-11821159	0.0013433
CACNA2D3	chr3-53043706-53047387	0.001343274
XKR6	chr8-10838624-10840663	0.00134322
CXXC1	chr18-51028136-51031463	0.001343147
LYRM4	chr6-5865500-5867419	0.00134308
GLUL	chr1-182794679-182796437	0.001343049
RABGEF1	chr7-65750268-65752247	0.001342982
XKR6	chr8-11801546-11809384	0.001342944
CXXC1	chr18-50819417-50821444	0.001342788
CCDC138	chr2-108583812-108586009	0.00134262
CXXC1	chr18-50306965-50309237	0.001342484
CXXC1	chr18-51151164-51154839	0.001342421
CXXC1	chr18-50297553-50299412	0.001342197
MED19	chr11-58572858-58580527	0.001342052
AGBL3	chr7-134292835-134293867	0.001341977
CXXC1	chr18-49485454-49488487	0.001341786
CXXC1	chr18-49476200-49477553	0.001341697
ZNF43	chr19-20775740-20777241	0.00134161
ZNF43	chr19-21603594-21605448	0.001341445
MED19	chr11-57740636-57742792	0.001341312
LYRM4	chr6-5994295-6004925	0.001341297
CXXC1	chr18-50356092-50357699	0.001341259
GLUL	chr1-182587872-182590022	0.001341207
CXXC1	chr18-49459381-49461573	0.00134116
ZNF43	chr19-20660653-20662007	0.001341051
XKR6	chr8-11844943-11849855	0.001341011
AGBL3	chr7-134126875-134127946	0.001340859
MED19	chr11-57761278-57762490	0.001340767
GLUL	chr1-182143071-182150314	0.001340757
CXXC1	chr18-50805614-50806808	0.001340631
CACNA2D3	chr3-53071837-53075081	0.001340517
SMARCAL1	chr2-217222264-217224036	0.001340427
CXXC1	chr18-49489890-49493356	0.001340421
XKR6	chr8-10332517-10335392	0.001340413
LYRM4	chr6-6006205-6009544	0.001340409
GLUL	chr1-182028994-182030651	0.001340378
CXXC1	chr18-50279806-50289439	0.001340209
CXXC1	chr18-50374311-50375777	0.001340132

AGBL3	chr7-134174588-134175375	0.001340092
LINC02273	chr4-152696603-152697703	0.001339586
LYRM4	chr6-6331212-6332929	0.001339511
GLUL	chr1-182192895-182194552	0.001339263
MED19	chr11-58567834-58568741	0.001339227
MED19	chr11-57711299-57713372	0.001339154
GLUL	chr1-181395794-181398789	0.001338965
CCDC138	chr2-108587050-108589165	0.001338577
CACNA2D3	chr3-53886931-53893410	0.001338571
CACNA2D3	chr3-53102514-53105288	0.001338548
MED19	chr11-57791267-57794095	0.001338478
LYRM4	chr6-6406905-6408041	0.001338308
CXXC1	chr18-49812613-49815126	0.001338284
RABGEF1	chr7-65770110-65771962	0.001338268
SMARCAL1	chr2-216497837-216499993	0.001338211
CXXC1	chr18-50265620-50268921	0.001337904
ZNF43	chr19-21766536-21768067	0.001337829
CACNA2D3	chr3-53880639-53885577	0.001337806
XKR6	chr8-10053374-10055956	0.001337634
SMARCAL1	chr2-216485237-216487342	0.00133755
GLUL	chr1-182393939-182395113	0.001337362
SMARCAL1	chr2-216411655-216413339	0.001337266
CCDC138	chr2-108618348-108622390	0.001337259
CXXC1	chr18-49819538-49821039	0.001337084
CCDC138	chr2-108635962-108637783	0.001336956
CACNA2D3	chr3-53109692-53110731	0.00133694
MED19	chr11-58524064-58525934	0.00133692
GLUL	chr1-182258847-182260187	0.001336892
MED19	chr11-57800359-57801474	0.001336646
CCDC138	chr2-109348121-109349947	0.001336465
CCDC138	chr2-109254400-109256442	0.001336162
MYC	chr8-127975230-127978753	0.001336152
CCDC138	chr2-108610867-108614242	0.00133607
XKR6	chr8-11856900-11864268	0.001336066
LINC02273	chr4-152778110-152780963	0.001336012
MYC	chr8-127915623-127918477	0.001335932
CACNA2D3	chr3-53748304-53750855	0.001335795
SMARCAL1	chr2-216352447-216353646	0.001335794
GLUL	chr1-182388595-182392754	0.001335755
ZNF43	chr19-22633576-22634954	0.001335688
CCDC138	chr2-108589712-108592035	0.001335571
MED19	chr11-57666153-57669704	0.00133542
CCDC138	chr2-108652212-108653233	0.001335071
CCDC138	chr2-108593509-108595589	0.001334903
AC093010.2	chr3-113838330-113839423	0.00133473
CACNA2D3	chr3-53113671-53115133	0.001334684
LINC02273	chr4-153082175-153083873	0.00133463
LINC02273	chr4-153111651-153116901	0.00133437
CCDC138	chr2-109238266-109239328	0.001334286
SMARCAL1	chr2-215903219-215904786	0.001334253

MYC	chr8-127979458-127983137	0.001334055
ZNF43	chr19-21835238-21836914	0.00133402
CCDC138	chr2-109613197-109615748	0.001333988
LINC02273	chr4-153151295-153154521	0.001333896
SMARCAL1	chr2-215793458-215794545	0.001333835
SMARCAL1	chr2-215899902-215901326	0.001333719
AC093010.2	chr3-113947226-113949023	0.001333615
MED19	chr11-57565313-57568835	0.001333503
SMARCAL1	chr2-216012585-216014065	0.001333352
SMARCAL1	chr2-215811291-215812434	0.001333302
SMARCAL1	chr2-215845923-215846903	0.001333067
LINC02273	chr4-153222039-153223700	0.001333055
LINC02273	chr4-153248323-153250038	0.001333041
RABGEF1	chr7-66678987-66683410	0.001333031
SMARCAL1	chr2-215748073-215751164	0.001332988
CCDC138	chr2-108718208-108721332	0.001332813
RABGEF1	chr7-66652971-66656744	0.001332774
SMARCAL1	chr2-216113619-216115749	0.001332681
MED19	chr11-57637528-57639986	0.001332632
MED19	chr11-57529448-57531810	0.001332579
CCDC138	chr2-109212669-109215733	0.001332562
AC093010.2	chr3-113830038-113831239	0.001332515
RABGEF1	chr7-65870922-65874391	0.001332406
MED19	chr11-57656694-57658663	0.001332399
ZNF43	chr19-22051932-22053501	0.001332356
MYC	chr8-127793559-127796604	0.001332064
CACNA2D3	chr3-53346423-53348597	0.001331965
MYC	chr8-127991697-127994308	0.001331904
CCDC138	chr2-109143503-109145244	0.001331866
CCDC138	chr2-109127844-109130774	0.001331827
ZNF43	chr19-21850716-21852755	0.001331709
CCDC138	chr2-109030596-109033601	0.001331683
MED19	chr11-57644546-57650586	0.001331616
XKR6	chr8-11901943-11903034	0.001331553
CACNA2D3	chr3-53129302-53131667	0.001331497
CCDC138	chr2-108785982-108787510	0.001331481
CCDC138	chr2-108986864-108989534	0.001331329
CACNA2D3	chr3-53164827-53172868	0.001331271
RABGEF1	chr7-66737632-66743968	0.001331173
MYC	chr8-128047943-128051417	0.001331067
MYC	chr8-128041732-128043694	0.00133106
SMARCAL1	chr2-216015658-216017760	0.001330848
MYC	chr8-128167370-128168791	0.00133056
XKR6	chr8-11864871-11869863	0.001330389
CACNA2D3	chr3-53179741-53181829	0.001330168
AC093010.2	chr3-114055718-114057746	0.001330087
CACNA2D3	chr3-53155755-53164079	0.001329982
RABGEF1	chr7-66626592-66633319	0.001329914
MED19	chr11-57513459-57516362	0.001329907
MED19	chr11-57424328-57427914	0.001329729

SMARCAL1	chr2-215728219-215729365	0.001329453
CCDC138	chr2-109635879-109636868	0.001329322
SMARCAL1	chr2-216107998-216110763	0.001329225
MED19	chr11-57457049-57461481	0.001329118
RABGEF1	chr7-66758223-66763408	0.001328816
MYC	chr8-128173495-128174572	0.001328782
MED19	chr11-57334511-57336866	0.001328611
CACNA2D3	chr3-53134461-53135337	0.001328393
ARHGAP31	chr3-119702331-119703630	0.00132831
ARHGAP31	chr3-119700246-119701705	0.001328229
AC093010.2	chr3-113748705-113749729	0.001328219
SMARCAL1	chr2-216057431-216058560	0.001328214
XKR6	chr8-11896617-11898849	0.001328178
AC093010.2	chr3-114290592-114292019	0.001328169
AC093010.2	chr3-114231318-114233425	0.001327917
CACNA2D3	chr3-53142602-53143816	0.001327826
RABGEF1	chr7-65980120-65982902	0.001327723
RABGEF1	chr7-66113893-66115869	0.00132752
CACNA2D3	chr3-53269537-53270920	0.001327457
SMARCAL1	chr2-216081175-216083262	0.00132739
MED19	chr11-57473472-57475086	0.001327355
RABGEF1	chr7-66842849-66849062	0.001327214
MED19	chr11-57482223-57483651	0.001327203
CACNA2D3	chr3-53193590-53197583	0.001327192
RABGEF1	chr7-66204391-66206195	0.001327104
CACNA2D3	chr3-53140222-53141933	0.001326946
MED19	chr11-57323373-57326470	0.001326866
AC093010.2	chr3-114152352-114153233	0.001326633
XKR6	chr8-11876981-11879161	0.001326565
AC093010.2	chr3-114307211-114309487	0.00132654
RABGEF1	chr7-66099456-66100719	0.001326479
MYC	chr8-128204843-128206525	0.001326371
AC093010.2	chr3-114221139-114223781	0.001326309
MED19	chr11-57475824-57477924	0.001326249
RABGEF1	chr7-66920326-66923264	0.001326224
ARHGAP31	chr3-119809243-119810780	0.001326029
XKR6	chr8-11885662-11889148	0.001325993
RABGEF1	chr7-66591114-66593490	0.001325908
RABGEF1	chr7-66075075-66077206	0.001325874
MYC	chr8-127759275-127761304	0.001325499
ARHGAP31	chr3-119676224-119678281	0.001325392
AC093010.2	chr3-114212642-114216815	0.001325321
RABGEF1	chr7-66412990-66414412	0.001325036
MYC	chr8-128539987-128541640	0.001325018
RABGEF1	chr7-66991749-66997480	0.001324783
MYC	chr8-128238876-128240802	0.001324743
MYC	chr8-128250289-128252175	0.00132456
CACNA2D3	chr3-53249260-53253292	0.001324557
CACNA2D3	chr3-53253872-53257884	0.001324472
MYC	chr8-128542168-128544216	0.001324424

AC093010.2	chr3-115099905-115100777	0.001324172
AC093010.2	chr3-113743235-113748148	0.001324118
SMARCAL1	chr2-215680754-215682702	0.001323817
BIRC3	chr11-101914354-101916406	0.001323164
ARHGAP31	chr3-120091828-120096564	0.00132315
RABGEF1	chr7-66557318-66558928	0.001323123
RABGEF1	chr7-66491623-66495128	0.001323029
AC093010.2	chr3-115145540-115148818	0.001322782
RABGEF1	chr7-67128520-67129669	0.001322316
BIRC3	chr11-102305724-102307834	0.001322291
BIRC3	chr11-103108849-103110825	0.001322186
BIRC3	chr11-103035867-103036565	0.0013221
BIRC3	chr11-103090883-103092871	0.001322096
AOPEP	chr9-95874432-95877127	0.001322072
BIRC3	chr11-102312299-102314714	0.00132192
MYC	chr8-128659133-128660265	0.001321804
BIRC3	chr11-102413292-102415993	0.001321778
AC093010.2	chr3-113736386-113737260	0.001321689
ARHGAP31	chr3-120348062-120350753	0.001321441
BIRC3	chr11-102919684-102920815	0.001321292
ARHGAP31	chr3-120416890-120418020	0.001321215
BIRC3	chr11-102293557-102294933	0.001321161
BIRC3	chr11-102360377-102362337	0.001321056
BIRC3	chr11-102109787-102112277	0.001320987
ARHGAP31	chr3-119637555-119639742	0.001320956
BIRC3	chr11-102446989-102447993	0.001320682
AC093010.2	chr3-113694935-113698140	0.001320644
BIRC3	chr11-102316270-102321585	0.001320479
AC093010.2	chr3-113018338-113020349	0.001320312
AOPEP	chr9-95724100-95725492	0.001319746
BIRC3	chr11-102345934-102350675	0.001319741
RABGEF1	chr7-67301079-67303678	0.001319673
BIRC3	chr11-102287063-102288601	0.001319661
PSMD7	chr16-74998047-75001021	0.001319656
AC093010.2	chr3-113625303-113627328	0.001319606
BIRC3	chr11-102268294-102269459	0.001319411
BIRC3	chr11-102341474-102342478	0.0013194
BIRC3	chr11-102559609-102561334	0.001319178
MYC	chr8-127733640-127740565	0.001319014
MYC	chr8-126556098-126559168	0.001318772
PSMD7	chr16-74983564-74985898	0.001318547
SMARCAL1	chr2-215435513-215436960	0.001318385
PSMD7	chr16-75064287-75065858	0.001318382
MYC	chr8-127168754-127171296	0.001318216
BIRC3	chr11-102448509-102450318	0.001318049
AOPEP	chr9-95004035-95005583	0.001317858
AC093010.2	chr3-113051103-113051736	0.001317801
AC093010.2	chr3-113606625-113607586	0.001317672
ARHGAP31	chr3-119351985-119353879	0.001317599
AOPEP	chr9-95316549-95318312	0.001317189

GATA3	chr10-7471499-7472639	0.00131686
GATA3	chr10-7406837-7413434	0.001316801
PSMD7	chr16-75147960-75149579	0.001316715
AOPEP	chr9-94870700-94871950	0.001316581
MYC	chr8-127196313-127197691	0.00131656
ARHGAP31	chr3-119627334-119629539	0.001316535
BIRC3	chr11-102546386-102547610	0.001316528
ARHGAP31	chr3-119462830-119464535	0.00131636
AOPEP	chr9-95515458-95517396	0.001316194
ARHGAP31	chr3-119321753-119324321	0.001316132
PSMD7	chr16-74774262-74775271	0.001315791
PSMD7	chr16-75074765-75076075	0.001315726
GATA3	chr10-7269098-7270370	0.001315619
PSMD7	chr16-75108684-75113281	0.001315586
BIRC3	chr11-102450895-102457022	0.001315568
SMARCAL1	chr2-215311173-215312947	0.001315495
PSMD7	chr16-75379902-75381166	0.001315439
AC093010.2	chr3-113531329-113535172	0.001315392
TOR3A	chr1-179034092-179035539	0.001315328
DLG2	chr11-85826103-85828367	0.001315242
AC093010.2	chr3-113275734-113276902	0.001315219
MYC	chr8-127386530-127387631	0.001315218
TOR3A	chr1-179025151-179027374	0.001315178
TOR3A	chr1-178724388-178726924	0.001315042
TOR3A	chr1-178541545-178543830	0.001315012
AOPEP	chr9-95462131-95463644	0.001314994
BIRC3	chr11-102495103-102496017	0.001314936
DLG2	chr11-85854237-85855808	0.001314867
MYC	chr8-127209617-127211171	0.001314853
GATA3	chr10-7258772-7261057	0.001314825
GATA3	chr10-7192384-7194293	0.001314765
GATA3	chr10-7480658-7485936	0.001314691
MPP6	chr7-23467437-23471943	0.001314577
AOPEP	chr9-94860195-94862263	0.001314539
GATA3	chr10-7184059-7187317	0.001314365
TOR3A	chr1-179080598-179083999	0.001314307
AC093010.2	chr3-113513975-113516789	0.001314243
DLG2	chr11-85823320-85824999	0.001314094
TOR3A	chr1-178093206-178094808	0.001314035
DLG2	chr11-86041653-86043258	0.001313962
PSMD7	chr16-75105088-75106433	0.001313908
MPP6	chr7-23473290-23475252	0.001313841
PSMD7	chr16-75098069-75100503	0.001313771
ARHGAP31	chr3-119034150-119035387	0.001313755
AOPEP	chr9-95505532-95512554	0.001313499
ARHGAP31	chr3-119300694-119302190	0.001313473
AOPEP	chr9-94725971-94727696	0.001313254
AOPEP	chr9-94653874-94656952	0.001313168
AOPEP	chr9-95493893-95495804	0.001313167
AOPEP	chr9-94668725-94669949	0.001313141

PSMD7	chr16-74709160-74710041	0.001313076
ARHGAP31	chr3-119586938-119589897	0.001312973
DLG2	chr11-86062038-86070605	0.001312796
ARHGAP31	chr3-119467976-119470150	0.001312774
GATA3	chr10-7120882-7121825	0.001312269
PSMD7	chr16-73153781-73154826	0.001312122
ARHGAP31	chr3-119238305-119242092	0.001312076
PHLPP2	chr16-71726093-71728266	0.001312002
AOPEP	chr9-94648888-94649912	0.001311884
PSMD7	chr16-75432300-75435157	0.00131179
ARHGAP31	chr3-119292573-119296765	0.001311749
RASGRP1	chr15-39334671-39335739	0.001311571
PHLPP2	chr16-71722662-71724593	0.001311514
PSMD7	chr16-74699562-74702343	0.001311341
MPP6	chr7-23489933-23491487	0.001311187
TOR3A	chr1-179088565-179089575	0.001311127
RASGRP1	chr15-39326231-39327968	0.001310926
DLG2	chr11-85808899-85812011	0.001310855
GATA3	chr10-7786364-7789273	0.00131082
UTP20	chr12-100127761-100128941	0.001310781
DLG2	chr11-86138323-86139643	0.001310684
PSMD7	chr16-74695439-74696539	0.001310165
ARHGAP31	chr3-119579179-119580764	0.001310158
PHLPP2	chr16-71806341-71811961	0.001309987
AOPEP	chr9-93950904-93953422	0.00130991
RASGRP1	chr15-39375803-39377103	0.001309876
AOPEP	chr9-94029910-94031548	0.001309816
GATA3	chr10-8182985-8183921	0.001309686
GATA3	chr10-8136794-8138369	0.001309354
RASGRP1	chr15-39312926-39313801	0.001309346
RASGRP1	chr15-38129451-38131591	0.001309134
AOPEP	chr9-94642882-94644073	0.001309005
PHLPP2	chr16-71563981-71566567	0.001308942
AOPEP	chr9-93574191-93579064	0.001308868
ARHGAP31	chr3-119497717-119499842	0.001308769
RASGRP1	chr15-38084168-38086638	0.001308757
AOPEP	chr9-93565795-93568002	0.001308693
UTP20	chr12-101875254-101879050	0.001308631
PSMD7	chr16-74666144-74667907	0.001308385
GATA3	chr10-7046000-7047071	0.001308331
PSMD7	chr16-74295724-74298278	0.001308168
RASGRP1	chr15-38690078-38691194	0.00130807
KCTD7	chr7-65750268-65752247	0.001307919
UTP20	chr12-101917416-101918161	0.001307908
AOPEP	chr9-94164671-94169310	0.001307904
TTC12	chr11-112158960-112161616	0.0013079
ZFHX3	chr16-74998047-75001021	0.001307844
ARHGAP31	chr3-119569185-119571129	0.001307777
PSMD7	chr16-75462186-75465499	0.001307733
TOR3A	chr1-180278548-180279566	0.001307571

UTP20	chr12-101838492-101840373	0.001307557
GATA3	chr10-8041366-8062418	0.001307369
UTP20	chr12-100137913-100139142	0.001307308
GATA3	chr10-8244867-8246107	0.001307297
GATA3	chr10-7817644-7819639	0.001307296
RASGRP1	chr15-38682890-38686740	0.001307155
MNDA	chr1-159935482-159938859	0.001307129
DLG2	chr11-86141299-86142566	0.001306962
ZFHX3	chr16-74983564-74985898	0.001306744
PHLPP2	chr16-71844303-71847300	0.001306683
ZFHX3	chr16-75064287-75065858	0.001306581
MPP6	chr7-23523509-23533506	0.001306576
RASGRP1	chr15-38251048-38254966	0.00130654
TTC12	chr11-112174991-112175939	0.001306479
MNDA	chr1-159908299-159932674	0.001306424
ARHGAP31	chr3-119558884-119560305	0.001306324
UTP20	chr12-102060848-102063398	0.001306291
DLG2	chr11-85755233-85757016	0.001306242
TOR3A	chr1-179129205-179130605	0.001306216
ARHGAP31	chr3-119561685-119563197	0.001306196
GATA3	chr10-8033844-8038379	0.001306085
AOPEP	chr9-94638306-94640415	0.001305881
RASGRP1	chr15-38670051-38673484	0.00130584
PHLPP2	chr16-71483633-71485250	0.00130582
PSMD7	chr16-75468540-75469430	0.00130571
PSMD7	chr16-74605455-74609672	0.001305597
RASGRP1	chr15-39387897-39388975	0.001305588
AOPEP	chr9-94258806-94260527	0.001305423
PRKCE	chr2-46534457-46538851	0.001305366
TTC12	chr11-114134804-114137698	0.001305328
RASGRP1	chr15-38072054-38073460	0.001305205
UTP20	chr12-100141352-100143675	0.001305094
TOR3A	chr1-180228842-180231882	0.001305077
MNDA	chr1-159944351-159946578	0.001305043
TTC12	chr11-114154158-114156839	0.001304948
UTP20	chr12-100198951-100203433	0.001304921
UTP20	chr12-101828476-101831647	0.001304867
UTP20	chr12-100163687-100164542	0.001304676
UTP20	chr12-102119146-102121327	0.001304655
IGF1R	chr15-99472715-99474707	0.001304624
MPP6	chr7-25115157-25123154	0.001304533
MPP6	chr7-25123746-25126406	0.001304451
PRKCE	chr2-46497598-46501048	0.001304423
AOPEP	chr9-94373799-94375747	0.001304414
UTP20	chr12-100266209-100269474	0.001304299
PRKCE	chr2-46540870-46546013	0.001304265
PHLPP2	chr16-72007826-72009324	0.001304142
PHLPP2	chr16-71850663-71854190	0.001304093
PSMD7	chr16-74367126-74369352	0.00130405
ZFHX3	chr16-74774262-74775271	0.001304012

PRKCE	chr2-46239054-46240385	0.001304004
IGF1R	chr15-99450418-99453848	0.001303973
PHLPP2	chr16-71893830-71897243	0.001303965
GATA3	chr10-6926975-6929800	0.001303963
ZFHX3	chr16-75074765-75076075	0.001303948
MNDA	chr1-159897878-159901715	0.001303854
RASGRP1	chr15-38650378-38653900	0.001303735
MPP6	chr7-25093149-25094659	0.001303723
TTC12	chr11-112219462-112220388	0.001303536
PHLPP2	chr16-71880144-71885650	0.001303379
KCTD7	chr7-65770110-65771962	0.001303328
IGF1R	chr15-99475493-99479148	0.001303306
GATA3	chr10-8330735-8333379	0.00130324
PRKCE	chr2-46296198-46300138	0.001303221
PHLPP2	chr16-71461462-71462860	0.001303152
MPP6	chr7-25028206-25029510	0.001303138
RASGRP1	chr15-38453667-38455453	0.001303102
MPP6	chr7-24978487-24982463	0.001303072
PRKCE	chr2-46224646-46225607	0.001302953
UTP20	chr12-101669821-101672401	0.00130295
PSMD7	chr16-74599765-74601971	0.001302862
TTC12	chr11-114082185-114084125	0.001302848
PDP1	chr8-93879964-93880992	0.001302842
TTC12	chr11-114157510-114163266	0.00130283
PDP1	chr8-93753624-93755739	0.001302829
TOR3A	chr1-180153594-180157538	0.001302771
MPP6	chr7-25179246-25181133	0.00130271
MPP6	chr7-24962501-24963854	0.001302709
PHLPP2	chr16-72092779-72094696	0.001302573
LPCAT2	chr16-54928039-54931612	0.001302518
PRKCE	chr2-46469790-46470990	0.001302476
IGF1R	chr15-99442079-99444625	0.001302461
UTP20	chr12-100573149-100574681	0.001302366
UTP20	chr12-101696556-101699170	0.001302323
LPCAT2	chr16-55437317-55438637	0.001302196
ZFHX3	chr16-73068243-73069533	0.001302097
MYOF	chr10-92572358-92576419	0.001302057
MNDA	chr1-158147549-158151166	0.001302041
DLG2	chr11-86143271-86145326	0.001302029
UTP20	chr12-101615562-101616807	0.001302007
PSMD7	chr16-74563272-74564582	0.001302004
UTP20	chr12-101822904-101824570	0.001301957
DLG2	chr11-85752036-85753896	0.00130192
PDP1	chr8-95024104-95025954	0.001301891
PDP1	chr8-93739864-93741917	0.001301754
PRKCE	chr2-46350217-46353424	0.001301747
PDP1	chr8-93883954-93886540	0.001301694
C1orf112	chr1-168535085-168536219	0.00130168
PRKCE	chr2-46547093-46552861	0.001301671
RASGRP1	chr15-38610478-38611834	0.001301662

MNDA	chr1-157652833-157653744	0.001301641
MYOF	chr10-92290076-92292761	0.001301596
PDP1	chr8-92964608-92966603	0.001301536
IGF1R	chr15-99424140-99426293	0.001301493
IGF1R	chr15-99434865-99439259	0.00130148
MPP6	chr7-23546748-23547634	0.001301463
IGF1R	chr15-99431914-99434080	0.001301412
TOR3A	chr1-179141009-179143764	0.001301331
ZFHX3	chr16-74709160-74710041	0.001301321
PRKCE	chr2-46461215-46462565	0.001301274
ZFHX3	chr16-73062834-73065185	0.001301191
RASGRP1	chr15-38562107-38565687	0.001301164
C1orf112	chr1-169043658-169045504	0.001301094
MPP6	chr7-24910482-24911765	0.001301092
GATA3	chr10-6919744-6920622	0.001301074
MNDA	chr1-159889207-159892185	0.001301033
UTP20	chr12-101743533-101744653	0.001300947
PDP1	chr8-93699960-93701201	0.001300906
MNDA	chr1-160030658-160032859	0.001300898
TTC12	chr11-114163818-114167613	0.001300898
PDP1	chr8-93990167-93992379	0.001300748
IGF1R	chr15-99250604-99252698	0.001300702
PDP1	chr8-93935075-93936524	0.001300686
PHLPP2	chr16-71288472-71290298	0.001300588
PRKCE	chr2-45935945-45936834	0.001300513
UTP20	chr12-101773493-101774282	0.001300484
TOR3A	chr1-180130814-180132548	0.001300439
MYOF	chr10-92590403-92594512	0.00130043
PDP1	chr8-93904011-93906545	0.001300424
ZFHX3	chr16-73153781-73154826	0.001300378
MNDA	chr1-159007644-159014528	0.001300342
MNDA	chr1-158179379-158181761	0.001300342
RASGRP1	chr15-38068785-38070230	0.001300311
UTP20	chr12-101406922-101408830	0.001300261
IGF1R	chr15-99485240-99487672	0.001300256
UTP20	chr12-100576373-100577313	0.0013002
MNDA	chr1-158930302-158932620	0.001300178
TTC12	chr11-114171353-114173683	0.001300136
PDP1	chr8-93915678-93918567	0.001300129
RASGRP1	chr15-39426489-39428174	0.00130011
C1orf112	chr1-168530864-168532457	0.001300043
TTC12	chr11-112225526-112227905	0.00130001
TTC12	chr11-114178903-114181100	0.001299943
GATA3	chr10-8402317-8405578	0.001299744
ZFHX3	chr16-74699562-74702343	0.001299602
GATA3	chr10-9095886-9098270	0.001299433
PHLPP2	chr16-72171920-72173042	0.001299382
UTP20	chr12-101279278-101280859	0.001299329
LPCAT2	chr16-55444049-55445561	0.001299312
DLG2	chr11-85682126-85683385	0.001299294

PRKCE	chr2-46568399-46570506	0.001299186
IGF1R	chr15-99104689-99106932	0.001298989
PDP1	chr8-94119525-94120860	0.001298984
ZFHX3	chr16-73055520-73059550	0.001298926
TTC12	chr11-114182705-114184074	0.001298906
MNDA	chr1-159853704-159856940	0.001298804
C1orf112	chr1-169794114-169795800	0.001298768
GATA3	chr10-9009949-9010711	0.001298736
IGF1R	chr15-98599957-98601065	0.001298528
PRKCE	chr2-45728303-45730562	0.001298511
ZFHX3	chr16-74695439-74696539	0.001298437
DLG2	chr11-85663024-85665958	0.001298412
TTC12	chr11-114072228-114076352	0.001298372
MAPK8	chr10-48671056-48673240	0.001298355
DLG2	chr11-86218208-86220257	0.001298349
GATA3	chr10-8415982-8417201	0.001298324
PRKCE	chr2-45887988-45893807	0.001298271
TOR3A	chr1-179175038-179176124	0.001298265
MPP6	chr7-24833473-24834786	0.001298232
KCTD7	chr7-66678987-66683410	0.001298229
C1orf112	chr1-169890761-169891715	0.001298103
DLG2	chr11-85647100-85648732	0.001298094
PRKCE	chr2-46697855-46700738	0.001298082
PRKCE	chr2-45770083-45771959	0.001298051
PRKCE	chr2-46615789-46617953	0.001298027
KCTD7	chr7-66652971-66656744	0.001297978
MAPK8	chr10-48667729-48668759	0.001297898
C1orf112	chr1-169105108-169108077	0.001297885
IGF1R	chr15-98646802-98652538	0.001297776
MPP6	chr7-23596877-23598354	0.001297737
IGF1R	chr15-98547241-98549077	0.001297706
MNDA	chr1-158830047-158831832	0.00129768
MYOF	chr10-92688516-92692801	0.001297668
C1orf112	chr1-168519970-168522145	0.001297658
KCTD7	chr7-65870922-65874391	0.00129762
PRKCE	chr2-45649358-45654697	0.001297619
DLG2	chr11-86161494-86163675	0.001297564
C1orf112	chr1-169711055-169712481	0.001297552
MAPK8	chr10-47165106-47167920	0.001297529
PRKCE	chr2-45860177-45861409	0.001297505
TTC12	chr11-112279813-112281053	0.001297505
TOR3A	chr1-179950610-179956170	0.001297497
MNDA	chr1-159015167-159016241	0.001297496
DLG2	chr11-86205348-86206393	0.001297429
IGF1R	chr15-98865064-98867101	0.001297379
TOR3A	chr1-179292258-179295714	0.001297375
DLG2	chr11-86243520-86246319	0.00129737
TOR3A	chr1-179228341-179230632	0.001297365
TTC12	chr11-113313891-113315828	0.001297348
TTC12	chr11-113188019-113189273	0.001297333

MNDA	chr1-158247934-158249272	0.001297309
DLG2	chr11-85627129-85629412	0.001297162
IGF1R	chr15-99508983-99512743	0.001297059
ZFHX3	chr16-73047393-73048953	0.00129703
IGF1R	chr15-98850639-98853801	0.001296965
IGF1R	chr15-99702993-99704857	0.001296907
TTC12	chr11-112289715-112290824	0.001296847
ZFHX3	chr16-72787146-72788778	0.001296826
MNDA	chr1-159828782-159829706	0.001296807
PHLPP2	chr16-70800310-70801911	0.001296805
IGF1R	chr15-99630739-99631873	0.001296676
ZFHX3	chr16-74666144-74667907	0.001296673
MAPK8	chr10-48683722-48686105	0.001296672
ZFHX3	chr16-72907950-72908958	0.001296657
C1orf112	chr1-170531220-170532974	0.001296612
TOR3A	chr1-179296951-179299579	0.001296578
C1orf112	chr1-169892576-169895121	0.001296505
ZFHX3	chr16-72927201-72928218	0.001296464
PDP1	chr8-94959885-94962303	0.001296463
ZFHX3	chr16-74295724-74298278	0.001296458
MAPK8	chr10-47206479-47207783	0.001296456
TTC12	chr11-114398923-114402696	0.001296437
KCTD7	chr7-66737632-66743968	0.00129642
MPP6	chr7-24754892-24758123	0.001296413
MPP6	chr7-24571423-24574794	0.001296361
ZFHX3	chr16-72930879-72932652	0.001296339
MPP6	chr7-23679585-23681336	0.00129628
SEC22A	chr3-123960489-123961778	0.001296235
PRKCE	chr2-44777537-44779336	0.001296149
PHLPP2	chr16-72202044-72204219	0.001295945
TTC12	chr11-113772958-113774389	0.001295876
DLG2	chr11-86201285-86204719	0.001295719
MAPK8	chr10-48655040-48657234	0.001295709
C1orf112	chr1-170073914-170075610	0.001295706
IGF1R	chr15-99613865-99615386	0.001295635
C1orf112	chr1-168512761-168514969	0.001295625
PRKCE	chr2-45643076-45644431	0.001295574
IGF1R	chr15-99706442-99707915	0.001295537
MPP6	chr7-24786198-24787541	0.001295524
RASGRP1	chr15-39453439-39454912	0.001295521
C1orf112	chr1-169666808-169668389	0.001295453
PDP1	chr8-94261540-94262814	0.001295389
IGF1R	chr15-99562202-99567823	0.001295387
DLG2	chr11-86301708-86302959	0.00129535
MPP6	chr7-24831145-24832931	0.001295326
MYOF	chr10-92741798-92743110	0.001295316
DLG2	chr11-86192554-86194131	0.001295309
LPCAT2	chr16-55508211-55510644	0.00129527
MNDA	chr1-158808966-158810754	0.001295267
C1orf112	chr1-169706716-169709115	0.001295214

KCTD7	chr7-66626592-66633319	0.001295193
MAPK8	chr10-47133785-47134993	0.001295187
LINC00174	chr7-65373138-65374798	0.001295071
IGF1R	chr15-97959580-97962071	0.001295068
MNDA	chr1-158285191-158287348	0.001295056
C1orf112	chr1-169691264-169695722	0.001294775
SEC22A	chr3-123618282-123619588	0.001294645
MNDA	chr1-159824906-159828063	0.001294302
TOR3A	chr1-179326951-179328398	0.001294295
DLG2	chr11-86671635-86672730	0.001294288
SEC22A	chr3-123583985-123586632	0.001294276
C1orf112	chr1-169604399-169605730	0.001294253
TTC12	chr11-114058408-114062594	0.001294249
MPP6	chr7-24820117-24822228	0.001294207
PRKCE	chr2-45567199-45568782	0.001294148
MYOF	chr10-92747188-92748295	0.001294137
KCTD7	chr7-66758223-66763408	0.001294123
TOR3A	chr1-179881459-179884414	0.001294002
C1orf112	chr1-168503540-168505144	0.001293951
SEC22A	chr3-123200923-123203095	0.00129395
PRKCE	chr2-45610241-45612092	0.001293926
ZFHX3	chr16-74605455-74609672	0.001293911
MAPK8	chr10-48799268-48801620	0.001293908
MPP6	chr7-24825528-24827439	0.001293831
PHLPP2	chr16-72664024-72665937	0.001293633
TTC12	chr11-113874514-113876825	0.001293631
C1orf112	chr1-169697540-169700053	0.001293531
C1orf112	chr1-169700991-169702054	0.0012935
TTC12	chr11-114438562-114440428	0.001293456
MYOF	chr10-92755967-92758168	0.001293408
C1orf112	chr1-169115876-169116794	0.0012934
MAPK8	chr10-48633847-48635287	0.001293262
MNDA	chr1-159067536-159069327	0.001293201
RASGRP1	chr15-39468221-39469344	0.001293104
IGF1R	chr15-99731754-99734294	0.001293062
KCTD7	chr7-65980120-65982902	0.001293059
LINC00174	chr7-65750268-65752247	0.001292877
KCTD7	chr7-66113893-66115869	0.00129286
PHLPP2	chr16-72787146-72788778	0.001292743
TTC12	chr11-114035784-114036702	0.001292575
PHLPP2	chr16-72907950-72908958	0.001292575
KCTD7	chr7-66842849-66849062	0.001292562
RASGRP1	chr15-39579910-39582060	0.001292505
SEC22A	chr3-123066708-123068015	0.001292476
MAPK8	chr10-47253991-47258726	0.001292455
KCTD7	chr7-66204391-66206195	0.001292455
ZFHX3	chr16-74367126-74369352	0.001292377
RASGRP1	chr15-39623205-39625650	0.001292269
LPCAT2	chr16-55832916-55834567	0.001292238
PDP1	chr8-94719239-94720812	0.001292228

KCTD7	chr7-66099456-66100719	0.001291847
MAPK8	chr10-49176920-49178257	0.001291771
MAPK8	chr10-48606618-48608530	0.001291635
C1orf112	chr1-169584623-169587124	0.001291617
KCTD7	chr7-66920326-66923264	0.001291598
PDP1	chr8-94419667-94420886	0.001291578
MYOF	chr10-92788998-92791060	0.001291554
TOR3A	chr1-179364855-179366849	0.001291548
LPCAT2	chr16-56190143-56192557	0.001291527
PDP1	chr8-94552725-94554843	0.001291461
MNDA	chr1-159780219-159782493	0.001291412
MAPK8	chr10-49189840-49191653	0.001291411
PDP1	chr8-94946663-94950633	0.001291338
KCTD7	chr7-66591114-66593490	0.001291291
KCTD7	chr7-66075075-66077206	0.001291259
LPCAT2	chr16-56048393-56049397	0.001291257
RASGRP1	chr15-39629092-39630044	0.001291252
LPCAT2	chr16-56194044-56194911	0.001291212
ZFHX3	chr16-74599765-74601971	0.0012912
PDP1	chr8-94821436-94824889	0.001291174
MAPK8	chr10-49187986-49188922	0.001291152
PHLPP2	chr16-70736456-70739583	0.001291099
TOR3A	chr1-179876270-179878340	0.001291068
MAPK8	chr10-49282713-49285977	0.00129094
MAPK8	chr10-48576782-48577813	0.001290687
KCTD7	chr7-66412990-66414412	0.001290441
ZFHX3	chr16-74563272-74564582	0.001290348
KCTD7	chr7-66991749-66997480	0.001290195
TOR3A	chr1-179814290-179815957	0.001290116
SEC22A	chr3-122295006-122296118	0.001290092
C1orf112	chr1-169366998-169369025	0.001290073
MNDA	chr1-159076254-159078152	0.001289957
PDP1	chr8-94474330-94475828	0.001289885
SEC22A	chr3-123026069-123028894	0.001289777
C1orf112	chr1-169483498-169487053	0.001289565
PDP1	chr8-94436300-94437691	0.001289526
MAPK8	chr10-48305881-48308047	0.001289468
MNDA	chr1-159090855-159092750	0.001289451
PDP1	chr8-94862186-94863662	0.001289286
SEC22A	chr3-122382661-122384938	0.001289216
LPCAT2	chr16-56201947-56203549	0.001289179
BLK	chr8-11491635-11494865	0.001289025
PDP1	chr8-94893424-94897210	0.001288802
MAPK8	chr10-49309515-49312510	0.001288639
KCTD7	chr7-66557318-66558928	0.001288579
KCTD7	chr7-66491623-66495128	0.001288488
SEC22A	chr3-122245570-122248281	0.001288455
BLK	chr8-11464613-11468224	0.001288374
LINC00174	chr7-65770110-65771962	0.001288339
MAPK8	chr10-47260402-47261900	0.00128793

KCTD7	chr7-67128520-67129669	0.001287792
MYOF	chr10-92847206-92849927	0.001287764
MAPK8	chr10-47552667-47554131	0.001287477
LPCAT2	chr16-56675485-56677028	0.001287401
AZIN1-AS1	chr8-102095508-102098459	0.001287394
BLK	chr8-11557713-11559394	0.001286864
LPCAT2	chr16-56662161-56663556	0.00128685
SEC22A	chr3-122645992-122647679	0.001286309
SEC22A	chr3-122575864-122578658	0.001286247
LINC01572	chr16-71726093-71728266	0.001286087
LPCAT2	chr16-56261838-56262986	0.00128601
LPCAT2	chr16-56681657-56683855	0.001285929
BLK	chr8-11453558-11459742	0.001285799
SEC22A	chr3-122678105-122683597	0.001285659
LINC01572	chr16-71722662-71724593	0.001285609
MAPK8	chr10-47384020-47385048	0.00128553
AZIN1-AS1	chr8-103414174-103416916	0.00128543
MAPK8	chr10-47311041-47312723	0.001285347
LPCAT2	chr16-56519069-56520960	0.001285326
LPCAT2	chr16-56576782-56577657	0.001285308
SEC22A	chr3-122232363-122234248	0.001285279
KCTD7	chr7-67301079-67303678	0.001285219
SEC22A	chr3-122562991-122565747	0.001285188
AZIN1-AS1	chr8-102106439-102109009	0.00128507
LPCAT2	chr16-56657019-56658943	0.001284801
MAPK8	chr10-49362339-49363344	0.001284782
AZIN1-AS1	chr8-103298373-103299587	0.00128466
SEC22A	chr3-122497231-122498599	0.001284625
PHLPP2	chr16-70695423-70697814	0.001284413
SEC22A	chr3-122513584-122515535	0.001284194
LINC01572	chr16-71806341-71811961	0.001284114
LPCAT2	chr16-56450296-56452753	0.001284047
SPEF2	chr5-36685256-36686413	0.001284043
LPCAT2	chr16-56606298-56611939	0.001283982
SPEF2	chr5-36657318-36658409	0.001283909
SEC22A	chr3-122066134-122069064	0.001283694
AZIN1-AS1	chr8-103019817-103022055	0.00128364
LPCAT2	chr16-56701365-56702683	0.001283565
LPCAT2	chr16-56356193-56358402	0.001283476
LINC00174	chr7-66678987-66683410	0.001283296
LINC01572	chr16-71563981-71566567	0.001283087
BLK	chr8-11283548-11285617	0.001283057
LINC00174	chr7-66652971-66656744	0.001283048
SEC22A	chr3-122074201-122078289	0.001283015
LPCAT2	chr16-56642286-56644096	0.001282962
BLK	chr8-11563692-11565023	0.001282962
SPEF2	chr5-36239654-36243314	0.001282946
BLK	chr8-12664886-12666069	0.001282924
LPCAT2	chr16-56423508-56426585	0.001282903
MYOF	chr10-92901613-92902477	0.001282895

LPCAT2	chr16-56624902-56626511	0.001282722
SPEF2	chr5-36689042-36690938	0.001282707
LINC00174	chr7-65870922-65874391	0.001282696
SEC22A	chr3-122100805-122102581	0.001282663
SEC22A	chr3-122063782-122064818	0.001282441
DAAM1	chr14-60247260-60250830	0.001282407
SPEF2	chr5-36150714-36153072	0.001282304
SPEF2	chr5-35924586-35926873	0.001282293
SEC22A	chr3-122091406-122093010	0.001282044
SPEF2	chr5-35850992-35860227	0.001281918
AZIN1-AS1	chr8-102122343-102124997	0.001281763
LINC00174	chr7-66737632-66743968	0.001281507
DAAM1	chr14-60327297-60328382	0.001281476
CASS4	chr20-57472784-57475094	0.00128138
BLK	chr8-11199037-11202359	0.001281276
DAAM1	chr14-60183228-60184747	0.001281226
AZIN1-AS1	chr8-102978131-102980383	0.001281128
BCL11A	chr2-60755572-60757437	0.001281123
SPEF2	chr5-35617199-35618681	0.001280957
CASS4	chr20-57480418-57482588	0.001280943
LINC01572	chr16-71844303-71847300	0.001280873
SPEF2	chr5-35482861-35484647	0.001280781
SPEF2	chr5-36722854-36725714	0.001280605
CASS4	chr20-57463572-57465493	0.001280391
LINC00174	chr7-66626592-66633319	0.001280296
BLK	chr8-11014900-11017401	0.0012801
LINC01572	chr16-71483633-71485250	0.001280027
SPEF2	chr5-35830179-35831560	0.00127982
BCL11A	chr2-60581360-60582255	0.001279807
BCL11A	chr2-60796170-60797442	0.001279688
MYOF	chr10-93427089-93428338	0.001279651
SPEF2	chr5-34838461-34839895	0.001279567
BLK	chr8-11768146-11771260	0.001279525
MYOF	chr10-93435977-93437959	0.001279522
CASS4	chr20-57449319-57452844	0.001279521
CASS4	chr20-57428662-57436451	0.001279446
CASS4	chr20-57425073-57427152	0.001279411
SPEF2	chr5-35319210-35320366	0.001279317
SEC22A	chr3-122055066-122056604	0.001279283
SPEF2	chr5-36744340-36746034	0.001279254
DAAM1	chr14-60330852-60332246	0.001279251
LINC00174	chr7-66758223-66763408	0.001279238
SPEF2	chr5-34498553-34499726	0.001279237
KCTD9	chr8-26588703-26590108	0.001279119
PHLPP2	chr16-70685204-70687686	0.001278961
MYOF	chr10-93363807-93365297	0.001278952
AZIN1-AS1	chr8-102236564-102240591	0.001278835
SPEF2	chr5-34914581-34916506	0.001278801
MYOF	chr10-94362148-94363581	0.0012788
DAAM1	chr14-60164057-60166695	0.001278654

MYOF	chr10-94402072-94404269	0.001278561
SPEF2	chr5-35672810-35674482	0.001278546
BLK	chr8-11823616-11824762	0.001278532
KCTD9	chr8-26575672-26580271	0.001278496
BLK	chr8-11819888-11821159	0.001278481
SPEF2	chr5-34928262-34930913	0.00127842
BLK	chr8-10838624-10840663	0.001278402
LINC01572	chr16-72007826-72009324	0.001278382
MYOF	chr10-93412431-93414056	0.001278349
LINC01572	chr16-71850663-71854190	0.001278334
CASS4	chr20-57596691-57597660	0.001278301
LINC01572	chr16-71893830-71897243	0.001278208
LINC00174	chr7-65980120-65982902	0.001278187
BLK	chr8-11801546-11809384	0.001278142
LINC00174	chr7-66113893-66115869	0.001277991
CASS4	chr20-57388751-57412930	0.001277944
BLK	chr8-12658544-12659927	0.001277943
LINC00174	chr7-66842849-66849062	0.001277695
LINC01572	chr16-71880144-71885650	0.001277634
CASS4	chr20-56391078-56393808	0.001277631
CASS4	chr20-56373644-56374567	0.001277613
LINC00174	chr7-66204391-66206195	0.00127759
MYOF	chr10-93481767-93483365	0.001277541
MYOF	chr10-93386495-93387713	0.001277513
MYOF	chr10-94544622-94546904	0.00127739
MYOF	chr10-93893493-93895043	0.001277368
KCTD9	chr8-26595436-26596611	0.001277303
KCTD9	chr8-26570948-26572849	0.001277121
LINC00174	chr7-66099456-66100719	0.001276989
LINC01572	chr16-72092779-72094696	0.001276844
BCL11A	chr2-60549132-60557896	0.0012768
KCTD9	chr8-26448106-26450585	0.001276795
LINC00174	chr7-66920326-66923264	0.001276743
AZIN1-AS1	chr8-102286898-102287983	0.001276709
KCTD9	chr8-26445723-26446770	0.001276707
KCTD9	chr8-26467083-26468043	0.001276513
PHLPP2	chr16-70522234-70526455	0.001276499
LINC00174	chr7-66591114-66593490	0.001276439
LINC00174	chr7-66075075-66077206	0.001276407
BLK	chr8-11844943-11849855	0.001276302
BCL11A	chr2-60817804-60820982	0.00127627
AZIN1-AS1	chr8-102861612-102866293	0.001276188
DAAM1	chr14-60151324-60152747	0.00127613
CASS4	chr20-56409283-56410196	0.001276103
SEC22A	chr3-122002582-122005566	0.001276037
CASS4	chr20-56358222-56359887	0.00127596
KCTD9	chr8-24271584-24272597	0.001275922
SPEF2	chr5-35821647-35824310	0.001275755
BLK	chr8-10332517-10335392	0.001275731
LINC00174	chr7-66412990-66414412	0.001275599

LINC00174	chr7-66991749-66997480	0.001275356
MYOF	chr10-93742519-93743364	0.001275206
MYOF	chr10-93495465-93498407	0.001275112
KCTD9	chr8-24293685-24294383	0.001275098
KCTD9	chr8-26440034-26441730	0.001275016
SYTL2	chr11-85826103-85828367	0.001274797
AZIN1-AS1	chr8-102410206-102413720	0.00127461
CASS4	chr20-57374333-57375997	0.001274456
SYTL2	chr11-85854237-85855808	0.001274433
CASS4	chr20-57604840-57606324	0.001274389
DAAM1	chr14-60090982-60094814	0.001274285
SPEF2	chr5-35780042-35781579	0.001274146
CASS4	chr20-56411315-56413860	0.001274145
MYOF	chr10-93701535-93703704	0.001274107
BCL11A	chr2-60515522-60516791	0.001274037
LINC00174	chr7-66557318-66558928	0.001273759
LINC01572	chr16-72171920-72173042	0.001273715
SYTL2	chr11-85823320-85824999	0.001273683
LINC00174	chr7-66491623-66495128	0.001273668
SYTL2	chr11-86041653-86043258	0.001273556
LINC00174	chr7-67128520-67129669	0.001272981
KCTD9	chr8-26608532-26612025	0.001272933
CASS4	chr20-56414639-56415762	0.001272862
BCL11A	chr2-60867311-60868783	0.001272816
BCL11A	chr2-60483937-60485403	0.001272703
SEL1L3	chr4-26877378-26880429	0.001272657
DAAM1	chr14-59964122-59966155	0.001272492
KCTD9	chr8-24349155-24350936	0.001272449
SYTL2	chr11-86062038-86070605	0.001272425
LINC01572	chr16-73068243-73069533	0.001272359
BCL11A	chr2-60438973-60440273	0.001272344
CASS4	chr20-56417098-56419905	0.001272335
SEL1L3	chr4-26872875-26874632	0.001271996
KCTD9	chr8-26436086-26438975	0.001271928
CASS4	chr20-56427626-56430817	0.001271635
AZIN1-AS1	chr8-102528219-102529352	0.001271611
BLK	chr8-11856900-11864268	0.001271596
LINC01572	chr16-73062834-73065185	0.001271473
BCL11A	chr2-60350793-60354358	0.001271424
SPEF2	chr5-35815494-35817703	0.001271321
SEL1L3	chr4-26882544-26887732	0.001271057
BCL11A	chr2-60965004-60967178	0.001271013
BCL11A	chr2-60880103-60889441	0.001270908
BCL11A	chr2-60971348-60973677	0.001270782
LINC01572	chr16-73153781-73154826	0.001270679
BCL11A	chr2-60926245-60927813	0.001270667
SYTL2	chr11-85808899-85812011	0.001270544
LINC00174	chr7-67301079-67303678	0.001270437
SYTL2	chr11-86138323-86139643	0.001270378
LINC01572	chr16-72202044-72204219	0.001270345

SPEF2	chr5-35796634-35797876	0.001270137
CASS4	chr20-57350434-57351992	0.001270003
CASS4	chr20-56439600-56443156	0.001269894
BCL11A	chr2-60992515-60993586	0.001269777
KCTD9	chr8-26265659-26267654	0.001269706
AZIN1-AS1	chr8-102814327-102815081	0.001269532
DAAM1	chr14-59576136-59577283	0.00126948
KCTD9	chr8-25457202-25459813	0.001269445
SEL1L3	chr4-26856716-26862811	0.00126944
LINC01572	chr16-73055520-73059550	0.00126926
KCTD9	chr8-26382144-26384508	0.001268983
SPEF2	chr5-35810250-35812003	0.001268956
BCL11A	chr2-59887723-59888547	0.001268939
BCL11A	chr2-61016611-61018609	0.001268693
KCTD9	chr8-26290336-26293747	0.001268536
SGMS2	chr4-108432271-108434572	0.001268508
SEL1L3	chr4-26992306-26993689	0.001268458
KCTD9	chr8-24386291-24388290	0.001268308
BCL11A	chr2-61064796-61066850	0.001268086
LINC01572	chr16-72664024-72665937	0.001268079
KCTD9	chr8-26302994-26305659	0.001267789
AZIN1-AS1	chr8-102567670-102568572	0.001267775
SGMS2	chr4-108359175-108360813	0.001267646
BCL11A	chr2-61144252-61146006	0.001267641
CASS4	chr20-56460244-56461187	0.001267453
LINC01572	chr16-73047393-73048953	0.001267408
BLK	chr8-11901943-11903034	0.001267301
SEL1L3	chr4-26318137-26322886	0.001267232
LINC01572	chr16-72787146-72788778	0.001267206
KCTD9	chr8-25242704-25244534	0.001267179
LINC01572	chr16-72907950-72908958	0.001267042
LINC01572	chr16-72927201-72928218	0.001266853
SYTL2	chr11-86141299-86142566	0.00126677
SEL1L3	chr4-26826369-26827690	0.001266767
LINC01572	chr16-72930879-72932652	0.001266732
CASS4	chr20-56467832-56470946	0.001266648
SGMS2	chr4-108466192-108467949	0.001266626
SEL1L3	chr4-26287614-26289087	0.001266385
BCL11A	chr2-61176043-61179494	0.001266382
SEL1L3	chr4-26583233-26584897	0.001266374
BLK	chr8-11864871-11869863	0.001266193
SYTL2	chr11-85755233-85757016	0.001266073
SGMS2	chr4-108080587-108081774	0.001265938
CASS4	chr20-56465755-56466838	0.001265863
SEL1L3	chr4-26821665-26824006	0.001265713
SGMS2	chr4-108106818-108108626	0.001265333
SGMS2	chr4-108070695-108073211	0.001265118
SEL1L3	chr4-25375096-25378207	0.001264953
DAAM1	chr14-59482224-59485721	0.001264863
SH3RF3	chr2-108448037-108450413	0.0012647

SGMS2	chr4-106707723-106709027	0.001264521
KCTD9	chr8-25183607-25186698	0.001264468
AZIN1-AS1	chr8-102583164-102584255	0.001264377
SGMS2	chr4-108355193-108356563	0.001264369
AZIN1-AS1	chr8-102651329-102652442	0.001264332
AZIN1-AS1	chr8-102653280-102659153	0.001264277
SH3RF3	chr2-108533324-108535557	0.001264266
KCTD9	chr8-25237271-25239876	0.001264261
SEL1L3	chr4-25859016-25864828	0.001264111
BLK	chr8-11896617-11898849	0.001264088
SGMS2	chr4-108048975-108052308	0.001264075
SGMS2	chr4-107718368-107722031	0.001263769
MIR646HG	chr20-59157931-59168100	0.001263769
BCL11A	chr2-61424899-61427098	0.001263754
MIR646HG	chr20-59173850-59175866	0.001263732
MIR646HG	chr20-59120186-59125168	0.001263714
SGMS2	chr4-108035746-108036993	0.001263694
MIR646HG	chr20-59041430-59044480	0.001263686
SGMS2	chr4-108043066-108046490	0.001263684
SEL1L3	chr4-25312178-25313553	0.001263648
MIR646HG	chr20-59144231-59154205	0.001263568
SGMS2	chr4-108619014-108622283	0.001263521
AZIN1-AS1	chr8-102803014-102811610	0.001263429
SEL1L3	chr4-26272627-26274176	0.00126336
SGMS2	chr4-108029046-108030807	0.001263275
AZIN1-AS1	chr8-102600140-102601831	0.001263243
SGMS2	chr4-108112787-108117788	0.001263022
SGMS2	chr4-106676179-106677035	0.001262973
KCTD9	chr8-25198314-25199777	0.001262921
AZIN1-AS1	chr8-102584801-102586588	0.001262807
MIR646HG	chr20-59177185-59179513	0.001262697
BLK	chr8-11876981-11879161	0.001262554
AZIN1-AS1	chr8-102660686-102663475	0.001262504
MIAT	chr22-26502690-26503758	0.001262275
MIR646HG	chr20-59031020-59033234	0.001262123
SGMS2	chr4-107988917-107991215	0.001262071
BLK	chr8-11885662-11889148	0.001262008
SYTL2	chr11-86143271-86145326	0.00126199
SYTL2	chr11-85752036-85753896	0.001261885
SGMS2	chr4-107824125-107825881	0.001261874
SH3RF3	chr2-108583812-108586009	0.001261511
SEL1L3	chr4-25233012-25235863	0.001261407
SEL1L3	chr4-25912823-25915129	0.001261324
MIAT	chr22-26511723-26513147	0.001261235
SGMS2	chr4-108649363-108651838	0.001261147
DAAM1	chr14-58330590-58331740	0.001260803
SGMS2	chr4-107930659-107932996	0.001260779
SGMS2	chr4-108170508-108173850	0.001260769
MIAT	chr22-26481495-26486052	0.001260664
MIR646HG	chr20-59207338-59208503	0.001260645

SGMS2	chr4-107837233-107839598	0.001260567
DAAM1	chr14-58395271-58397038	0.001260553
BCL11A	chr2-61469591-61472952	0.001260467
DAAM1	chr14-58296529-58300336	0.001260451
AZIN1-AS1	chr8-102787779-102791692	0.001260353
SGMS2	chr4-108159763-108161575	0.00126029
DAAM1	chr14-58280952-58282471	0.001260264
DAAM1	chr14-58243713-58245955	0.001260213
AZIN1-AS1	chr8-102794151-102796103	0.001260176
DAAM1	chr14-59463986-59466163	0.00125977
SEL1L3	chr4-26217695-26219395	0.001259677
DAAM1	chr14-58238151-58241483	0.001259562
SEL1L3	chr4-25172837-25174091	0.001259524
SYTL2	chr11-85682126-85683385	0.00125934
SGMS2	chr4-108165117-108169957	0.001259144
DAAM1	chr14-58426263-58428743	0.001259046
MIR646HG	chr20-59221864-59223331	0.001258539
SYTL2	chr11-85663024-85665958	0.001258487
MIAT	chr22-26572959-26574122	0.001258463
SEL1L3	chr4-26074176-26076917	0.001258436
SYTL2	chr11-86218208-86220257	0.001258423
SEL1L3	chr4-25159298-25161852	0.001258195
BCL11A	chr2-61693741-61695585	0.001258179
SYTL2	chr11-85647100-85648732	0.001258179
BCL11A	chr2-61535907-61539728	0.001258174
MIR646HG	chr20-59003629-59009123	0.001258108
OSBPL3	chr7-25115157-25123154	0.001257877
OSBPL3	chr7-25123746-25126406	0.001257797
DAAM1	chr14-58199065-58201280	0.001257788
SH3RF3	chr2-108587050-108589165	0.001257711
SYTL2	chr11-86161494-86163675	0.001257663
SEL1L3	chr4-26197147-26198024	0.001257626
SYTL2	chr11-86205348-86206393	0.001257533
SYTL2	chr11-86243520-86246319	0.001257475
MIR646HG	chr20-59253049-59254493	0.001257389
SYTL2	chr11-85627129-85629412	0.001257275
MIR646HG	chr20-59402635-59404188	0.001257227
MIAT	chr22-26479032-26480838	0.001257122
OSBPL3	chr7-25093149-25094659	0.001257096
MIR646HG	chr20-59722282-59723764	0.0012569
DAAM1	chr14-58637049-58638966	0.001256747
SEL1L3	chr4-24978648-24980503	0.001256571
OSBPL3	chr7-25028206-25029510	0.001256532
SH3RF3	chr2-108618348-108622390	0.001256473
OSBPL3	chr7-24978487-24982463	0.001256469
SH3RF3	chr2-108635962-108637783	0.00125619
DAAM1	chr14-59187499-59190788	0.001256133
OSBPL3	chr7-24962501-24963854	0.001256119
OSBPL3	chr7-25179246-25181133	0.001256118
SYTL2	chr11-86201285-86204719	0.001255874

SH3RF3	chr2-109348121-109349947	0.001255727
MIAT	chr22-26578955-26580369	0.001255611
SYTL2	chr11-86301708-86302959	0.001255517
SYTL2	chr11-86192554-86194131	0.001255477
SH3RF3	chr2-109254400-109256442	0.001255442
DAAM1	chr14-58151573-58152691	0.001255432
SH3RF3	chr2-108610867-108614242	0.001255357
DAAM1	chr14-58747023-58749718	0.001255244
MIR646HG	chr20-59932892-59934616	0.001255171
SH3RF3	chr2-108589712-108592035	0.001254887
OSBPL3	chr7-24910482-24911765	0.001254559
SYTL2	chr11-86671635-86672730	0.001254487
SH3RF3	chr2-108652212-108653233	0.001254418
SH3RF3	chr2-108593509-108595589	0.00125426
SEL1L3	chr4-24973693-24975842	0.001253917
SH3RF3	chr2-109238266-109239328	0.001253679
MIAT	chr22-26586436-26593257	0.001253666
AC104389.5	chr11-6200371-6202164	0.001253511
MIAT	chr22-26432348-26433274	0.001253491
SH3RF3	chr2-109613197-109615748	0.001253399
OSBPL3	chr7-25856447-25857787	0.001253346
AC104389.5	chr11-6233589-6235907	0.001252972
MIR646HG	chr20-60136615-60139012	0.001252901
AC104389.5	chr11-5808359-5809626	0.001252808
MIAT	chr22-26596717-26597580	0.001252329
SH3RF3	chr2-108718208-108721332	0.001252295
MIR646HG	chr20-58980437-58983385	0.001252189
AC104389.5	chr11-5683090-5685698	0.001252162
SH3RF3	chr2-109212669-109215733	0.001252059
AC104389.5	chr11-5624010-5626509	0.001252012
MIR646HG	chr20-59938589-59941346	0.001252009
AC104389.5	chr11-5700196-5701675	0.001251942
MIAT	chr22-27132939-27134971	0.001251881
OSBPL3	chr7-24833473-24834786	0.001251802
AC104389.5	chr11-5688623-5692793	0.001251786
MIAT	chr22-27112897-27113971	0.001251719
SH3RF3	chr2-109143503-109145244	0.001251405
SH3RF3	chr2-109127844-109130774	0.001251369
MIAT	chr22-26427870-26431833	0.001251346
SH3RF3	chr2-109030596-109033601	0.001251233
MIAT	chr22-25447558-25449027	0.001251222
OSBPL3	chr7-26078623-26079596	0.001251097
AC104389.5	chr11-6377927-6379859	0.001251089
SH3RF3	chr2-108785982-108787510	0.001251043
OSBPL3	chr7-25860309-25863066	0.001250998
MIAT	chr22-25561832-25567760	0.001250903
SH3RF3	chr2-108986864-108989534	0.001250902
OSBPL3	chr7-25949745-25952183	0.001250846
MIAT	chr22-27800755-27802653	0.001250628
MIAT	chr22-26641793-26643997	0.00125062

MIAT	chr22-27150146-27151140	0.001250553
AC104389.5	chr11-4606705-4608892	0.001250546
OSBPL3	chr7-25892757-25896004	0.001250264
OSBPL3	chr7-24754892-24758123	0.001250047
OSBPL3	chr7-24571423-24574794	0.001249997
MIAT	chr22-27669130-27670769	0.001249611
MIAT	chr22-26670843-26675044	0.001249602
MIR646HG	chr20-60054513-60059140	0.00124948
OSBPL3	chr7-26096863-26098660	0.001249309
CPPED1	chr16-12078784-12081505	0.001249249
OSBPL3	chr7-24786198-24787541	0.00124919
CPPED1	chr16-12033748-12035682	0.001249088
SH3RF3	chr2-109635879-109636868	0.001249016
OSBPL3	chr7-24831145-24832931	0.001249
AC104389.5	chr11-6417923-6420117	0.00124895
AC104389.5	chr11-6389457-6392507	0.001248931
MIR646HG	chr20-59948229-59949549	0.001248909
AC104389.5	chr11-6437784-6439451	0.001248429
ALG9	chr11-110711492-110713377	0.001248294
AC104389.5	chr11-6413054-6416358	0.001248225
MIAT	chr22-26644839-26645900	0.001248214
OSBPL3	chr7-24820117-24822228	0.00124792
AC104389.5	chr11-6401386-6405934	0.001247867
CEMIP	chr15-81322660-81325051	0.001247815
MIR646HG	chr20-60045773-60046814	0.001247796
OSBPL3	chr7-24825528-24827439	0.001247558
CPPED1	chr16-12087812-12090433	0.001247264
CEMIP	chr15-79960473-79963079	0.001246904
CEMIP	chr15-79965812-79974030	0.001246851
MIAT	chr22-26665139-26666106	0.001246816
CPPED1	chr16-12009272-12011330	0.0012466
CPPED1	chr16-13956938-13958227	0.00124649
MIR646HG	chr20-58906118-58908702	0.001246267
CPPED1	chr16-11611814-11615178	0.001245975
MIAT	chr22-26647496-26649739	0.001245962
AC104389.5	chr11-6472446-6476295	0.001245897
ALG9	chr11-110797266-110798822	0.001245542
OSBPL3	chr7-26099329-26102646	0.001245389
CPPED1	chr16-11638919-11643007	0.001245388
MIAT	chr22-26656848-26659493	0.001245282
CPPED1	chr16-11663245-11683246	0.001245219
CPPED1	chr16-13919204-13921067	0.001245174
CPPED1	chr16-12458196-12460146	0.001244952
ALG9	chr11-111936514-111939321	0.001244712
SH3RF3	chr2-110204096-110205624	0.001244701
CEMIP	chr15-81310727-81313732	0.001244674
CEMIP	chr15-80159735-80161313	0.001244571
CEMIP	chr15-79953572-79955217	0.001244467
ALG9	chr11-111931391-111932431	0.00124446
CPPED1	chr16-11628827-11630700	0.001244163

CPPED1	chr16-12800649-12804550	0.001244152
CEMIP	chr15-79977103-79979750	0.001244143
CEMIP	chr15-80151842-80153883	0.001244076
CPPED1	chr16-11618249-11623214	0.001243803
ALG9	chr11-112024058-112026147	0.001243674
CPPED1	chr16-11975842-11978008	0.001243369
CPPED1	chr16-11688845-11690695	0.001243284
CPPED1	chr16-11624227-11625825	0.001243173
CEMIP	chr15-80694185-80696799	0.001243161
CPPED1	chr16-11809600-11810860	0.001242874
SH3RF3	chr2-110211815-110213022	0.001242681
ALG9	chr11-112073448-112075106	0.00124264
ICOS	chr2-202870583-202872307	0.001242617
ICOS	chr2-202911064-202912975	0.001242578
CPPED1	chr16-11813432-11815708	0.001242484
MIR646HG	chr20-58901097-58904020	0.001242262
ALG9	chr11-111925782-111927948	0.001242256
ALG9	chr11-112085897-112087600	0.001242244
AC104389.5	chr11-6480257-6482937	0.001242119
ALG9	chr11-112158960-112161616	0.001241916
CPPED1	chr16-11790845-11800894	0.00124183
CEMIP	chr15-80072033-80073019	0.001241682
ALG9	chr11-111018437-111020487	0.001241522
CPPED1	chr16-11850588-11852898	0.001241417
CPPED1	chr16-11913195-11916876	0.001241388
CEMIP	chr15-79922396-79924746	0.001241271
CEMIP	chr15-80984386-80985648	0.00124124
OSBPL3	chr7-26151103-26157408	0.001241038
ICOS	chr2-202996883-202998030	0.001240895
CPPED1	chr16-11727755-11729383	0.001240798
MIR646HG	chr20-58888078-58900423	0.001240759
ALG9	chr11-112174991-112175939	0.001240566
CEMIP	chr15-79981452-79982752	0.001240518
CEMIP	chr15-81301374-81304987	0.001240189
CPPED1	chr16-11781866-11788396	0.001240161
CEMIP	chr15-80988717-80992671	0.001240159
CEMIP	chr15-80998207-81003237	0.001240006
CEMIP	chr15-81023028-81025345	0.001239714
CPPED1	chr16-11739203-11745167	0.001239517
CEMIP	chr15-80058465-80062103	0.001239077
CEMIP	chr15-79895821-79901208	0.001239036
ALG9	chr11-111877607-111880559	0.001238802
AC104389.5	chr11-6745566-6747523	0.001238796
AC104389.5	chr11-6602657-6607748	0.001238712
OSBPL3	chr7-26182360-26203181	0.001238501
CEMIP	chr15-79993124-79994005	0.001238417
CEMIP	chr15-81265255-81266858	0.001238359
ALG9	chr11-112219462-112220388	0.001237771
NDUFAB1	chr16-23518147-23519679	0.001237735
ALG9	chr11-111176732-111178144	0.001237632

ICOS	chr2-203012180-203016139	0.001237404
NDUFAB1	chr16-23520264-23521693	0.001236822
AC104389.5	chr11-6609429-6613509	0.001236799
NDUFAB1	chr16-23508116-23511129	0.001236542
CEMIP	chr15-81291307-81300419	0.001236469
CEMIP	chr15-81274768-81275681	0.001236304
AC104389.5	chr11-6616382-6622552	0.001236247
AC104389.5	chr11-6681999-6684338	0.00123618
AC104389.5	chr11-6635065-6636153	0.001236074
ALG9	chr11-111870535-111872213	0.001235717
AC104389.5	chr11-6645841-6649554	0.00123561
AC104389.5	chr11-6652979-6657116	0.001235294
ALG9	chr11-111378516-111380364	0.00123526
NDUFAB1	chr16-23556102-23558571	0.00123522
CEMIP	chr15-81284356-81287520	0.001235174
ICOS	chr2-203656398-203657315	0.001235104
ALG9	chr11-111446040-111447223	0.001234902
ALG9	chr11-111539267-111542030	0.001234837
NDUFAB1	chr16-23678252-23679811	0.00123469
ALG9	chr11-111436453-111437225	0.001234663
ICOS	chr2-203704358-203712298	0.001234643
NDUFAB1	chr16-23640377-23642712	0.001234434
ALG9	chr11-112225526-112227905	0.001234423
NDUFAB1	chr16-23595533-23597426	0.001234328
ALG9	chr11-111601381-111605196	0.001234308
ALG9	chr11-111765176-111767399	0.001234231
NDUFAB1	chr16-23825896-23827695	0.001234132
ICOS	chr2-203649675-203651712	0.001233694
ICOS	chr2-203237922-203240438	0.001233597
NDUFAB1	chr16-23504004-23507119	0.001233154
ICOS	chr2-203717869-203718804	0.001232652
NDUFAB1	chr16-23835239-23838958	0.001232647
ICOS	chr2-204967096-204968233	0.001232519
ALG9	chr11-112279813-112281053	0.001232045
ICOS	chr2-203533596-203536050	0.001231803
ICOS	chr2-203950280-203952110	0.001231634
IL7R	chr5-36685256-36686413	0.001231472
ICOS	chr2-203327541-203329291	0.001231446
ALG9	chr11-112289715-112290824	0.00123142
IL7R	chr5-36657318-36658409	0.001231344
NDUFAB1	chr16-23847725-23849307	0.001231043
ICOS	chr2-204970678-204971893	0.001230839
ICOS	chr2-203719521-203721884	0.001230666
ICOS	chr2-203814966-203815923	0.001230566
IL7R	chr5-36239654-36243314	0.001230419

gene	p_val	avg_log2FC	pct.1	pct.2	p_val_adj	cluster
ABLIM1	0	-1.663796467	0.066	0.485	0	CD14 Mono
CD96	0	-2.089552061	0.071	0.582	0	CD14 Mono
EEF1A1	0	-1.772367173	0.974	0.996	0	CD14 Mono
RPS13	0	-1.30648549	0.768	0.931	0	CD14 Mono
RPS6	0	-1.750811574	0.826	0.971	0	CD14 Mono
RASGRP1	3.95E-273	-1.385540091	0.068	0.432	9.73E-269	CD14 Mono
NR3C2	1.05E-253	-1.529634148	0.067	0.415	2.59E-249	CD14 Mono
PATJ	4.51E-209	-1.260055777	0.048	0.347	1.11E-204	CD14 Mono
LINC01934	8.15E-202	-1.507847834	0.053	0.345	2.01E-197	CD14 Mono
FAM49A	2.84E-189	-1.764955191	0.016	0.43	7.00E-185	CD8 Naive
TAFA1	5.34E-185	-1.432801928	0.039	0.312	1.31E-180	CD14 Mono
KCNQ5	2.42E-179	-1.440579837	0.03	0.293	5.95E-175	CD14 Mono
SAMD3	2.51E-179	-1.269776182	0.035	0.3	6.17E-175	CD14 Mono
FAM49A	6.00E-167	-1.660192113	0.035	0.425	1.48E-162	CD4 Naive
MCTP1	1.14E-163	-1.886137351	0.03	0.405	2.80E-159	CD8 Naive
GAB2	7.61E-163	-1.830213801	0.038	0.414	1.87E-158	CD8 Naive
IRAK3	1.26E-158	-1.863088285	0.015	0.38	3.09E-154	CD8 Naive
PTPRE	4.73E-158	-1.577053894	0.081	0.456	1.16E-153	CD8 Naive
KYNU	3.47E-152	-1.43677845	0.011	0.365	8.55E-148	CD8 Naive
MAL	4.62E-152	-1.07062038	0.052	0.292	1.14E-147	CD14 Mono
FAM49A	5.16E-150	-1.744871745	0.016	0.418	1.27E-145	CD4 TCM
GAB2	5.75E-150	-1.763409631	0.047	0.411	1.41E-145	CD4 Naive
TRIO	7.54E-150	-1.29234881	0.022	0.377	1.85E-145	CD8 Naive
GAS7	5.40E-146	-1.506927907	0.022	0.368	1.33E-141	CD8 Naive
IRAK3	4.76E-141	-1.757166032	0.031	0.376	1.17E-136	CD4 Naive
TRIO	4.82E-140	-1.281110992	0.029	0.375	1.19E-135	CD4 Naive
PTPRE	1.88E-139	-1.480576961	0.097	0.452	4.64E-135	CD4 Naive
CPPED1	2.42E-139	-1.448064827	0.042	0.383	5.95E-135	CD8 Naive
DOCK5	1.45E-136	-1.339095857	0.008	0.332	3.56E-132	CD8 Naive
MZT2B	6.07E-136	-0.835688411	0.188	0.425	1.49E-131	CD14 Mono
MCTP1	7.63E-134	-1.678779871	0.06	0.399	1.88E-129	CD4 Naive
GAS7	5.17E-133	-1.431849523	0.031	0.365	1.27E-128	CD4 Naive
KYNU	1.45E-129	-1.325800114	0.032	0.36	3.56E-125	CD4 Naive
CPPED1	7.70E-129	-1.385630395	0.048	0.381	1.90E-124	CD4 Naive
ZHX2	1.61E-125	-0.944627198	0.193	0.421	3.97E-121	CD14 Mono
DOCK5	3.24E-124	-1.283611964	0.018	0.329	7.97E-120	CD4 Naive
KLF4	1.08E-123	-1.307890782	0.016	0.321	2.67E-119	CD8 Naive
MNDA	2.20E-123	-1.39065108	0.131	0.452	5.41E-119	CD8 Naive
IRAK3	4.18E-123	-1.828268581	0.02	0.369	1.03E-118	CD4 TCM
TNS3	1.36E-122	-1.099374337	0.003	0.301	3.35E-118	CD8 Naive
KYNU	4.03E-122	-1.420196438	0.009	0.355	9.91E-118	CD4 TCM
IL15	6.66E-119	-1.194122727	0.015	0.311	1.64E-114	CD8 Naive
MCTP1	4.96E-118	-1.79521642	0.051	0.392	1.22E-113	CD4 TCM
KIF13A	2.47E-114	-1.225673188	0.013	0.298	6.07E-110	CD8 Naive
EPSTI1	4.93E-113	-1.164971253	0.186	0.504	1.21E-108	CD8 Naive
TRIO	6.16E-112	-1.22197094	0.031	0.365	1.52E-107	CD4 TCM
GAS7	4.05E-111	-1.447246574	0.028	0.356	9.97E-107	CD4 TCM
AP003086.1	9.28E-111	-1.115804207	0.035	0.325	2.28E-106	CD8 Naive
RTN1	2.00E-109	-1.348010471	0.011	0.286	4.93E-105	CD8 Naive

ANKH	2.31E-109	-0.830928362	0.08	0.276	5.68E-105	CD14 Mono
KLF4	4.97E-109	-1.219043073	0.028	0.317	1.22E-104	CD4 Naive
TNS3	2.68E-108	-1.047400527	0.017	0.298	6.58E-104	CD4 Naive
SATB1-AS1	7.59E-106	-0.876439618	0.042	0.223	1.87E-101	CD14 Mono
IL15	9.61E-106	-1.140827408	0.028	0.308	2.37E-101	CD4 Naive
DOCK5	1.43E-105	-1.304436228	0.012	0.322	3.52E-101	CD4 TCM
RGS2	1.94E-105	-1.196801014	0.068	0.357	4.76E-101	CD8 Naive
MYOF	3.32E-104	-1.138613174	0.017	0.286	8.18E-100	CD8 Naive
PID1	1.26E-103	-1.76019086	0.015	0.28	3.10E-99	CD8 Naive
AP003086.1	1.27E-103	-1.08051785	0.04	0.323	3.12E-99	CD4 Naive
KIF13A	9.84E-101	-1.145325166	0.025	0.295	2.42E-96	CD4 Naive
AHR	2.37E-100	-1.119826498	0.074	0.358	5.82E-96	CD8 Naive
GAB2	4.64E-100	-1.571743832	0.084	0.397	1.14E-95	CD4 TCM
GATA3	1.04E-99	-0.697478636	0.022	0.185	2.57E-95	CD14 Mono
SYNE1	4.61E-99	-0.915890502	0.29	0.483	1.13E-94	CD14 Mono
RIPK2	5.30E-99	-1.131614963	0.051	0.329	1.31E-94	CD4 Naive
RTN1	7.05E-99	-1.293618806	0.021	0.283	1.73E-94	CD4 Naive
STK3	6.23E-98	-0.948977794	0.033	0.298	1.53E-93	CD8 Naive
CPPED1	2.20E-97	-1.356954091	0.065	0.37	5.40E-93	CD4 TCM
MYOF	2.60E-97	-1.118161878	0.023	0.284	6.40E-93	CD4 Naive
KLF4	3.19E-97	-1.284547388	0.017	0.311	7.84E-93	CD4 TCM
TRAT1	2.87E-96	-0.718731842	0.031	0.194	7.07E-92	CD14 Mono
XKR6	6.73E-96	-0.775823913	0.092	0.278	1.66E-91	CD14 Mono
BACH1	1.01E-95	-1.153654723	0.222	0.515	2.49E-91	CD4 Naive
PTPRE	3.04E-95	-1.359905561	0.134	0.438	7.48E-91	CD4 TCM
MNDA	1.14E-94	-1.344514922	0.14	0.441	2.81E-90	CD4 TCM
PRKCE	7.42E-94	-0.97498	0.165	0.462	1.83E-89	CD8 Naive
BACH1	1.64E-93	-1.21733287	0.25	0.512	4.03E-89	CD8 Naive
ZSWIM6	1.76E-93	-1.074586932	0.254	0.551	4.34E-89	CD4 Naive
C9orf72	5.00E-93	-1.176097351	0.158	0.464	1.23E-88	CD4 TCM
ZSWIM6	6.75E-93	-1.153073337	0.281	0.548	1.66E-88	CD8 Naive
EPSTI1	8.38E-93	-0.987604766	0.197	0.501	2.06E-88	CD4 Naive
PID1	1.30E-92	-1.677083738	0.026	0.277	3.19E-88	CD4 Naive
TNS3	1.53E-92	-1.053346398	0.01	0.291	3.76E-88	CD4 TCM
NUCB2	1.62E-92	-0.763297858	0.094	0.274	4.00E-88	CD14 Mono
RGS2	4.15E-92	-1.106498802	0.079	0.354	1.02E-87	CD4 Naive
ICOS	1.32E-90	-0.693256444	0.017	0.166	3.25E-86	CD14 Mono
MZT2A	2.05E-90	-0.619531379	0.075	0.248	5.05E-86	CD14 Mono
CEBPD	2.31E-88	-0.919351762	0.05	0.304	5.68E-84	CD8 Naive
WDFY4	1.59E-87	-0.943257151	0.007	0.236	3.91E-83	CD8 Naive
ZNF827	6.20E-87	-0.609897521	0.017	0.161	1.53E-82	CD14 Mono
KIF13A	7.10E-87	-1.157151651	0.017	0.289	1.75E-82	CD4 TCM
USP53	2.91E-86	-0.618956299	0.029	0.179	7.17E-82	CD14 Mono
C9orf72	2.97E-86	-1.014849457	0.2	0.467	7.32E-82	CD8 Naive
PRKN	1.28E-85	-0.945547246	0.114	0.287	3.15E-81	CD14 Mono
EPB41L3	2.89E-85	-0.836982559	0.015	0.244	7.12E-81	CD8 Naive
VOPP1	2.94E-85	-0.782017269	0.312	0.484	7.23E-81	CD14 Mono
RTN1	4.02E-85	-1.316240904	0.014	0.278	9.90E-81	CD4 TCM
WDFY4	6.86E-85	-0.935802228	0.007	0.235	1.69E-80	CD4 Naive
PID1	3.03E-84	-1.754420174	0.012	0.272	7.47E-80	CD4 TCM

PRKCE	7.90E-84	-0.87259378	0.17	0.46	1.94E-79	CD4 Naive
MICAL2	7.25E-83	-0.812130984	0.011	0.232	1.78E-78	CD8 Naive
BACH1	2.03E-81	-1.231295584	0.235	0.506	4.99E-77	CD4 TCM
CEBPD	1.45E-80	-0.970905728	0.033	0.299	3.57E-76	CD4 TCM
MNDA	1.82E-80	-1.077655558	0.177	0.443	4.49E-76	CD4 Naive
STK3	4.39E-80	-0.833548767	0.05	0.294	1.08E-75	CD4 Naive
CEBPD	5.16E-80	-0.860455258	0.056	0.302	1.27E-75	CD4 Naive
RIPK2	8.29E-80	-1.01174383	0.081	0.325	2.04E-75	CD8 Naive
MYOF	2.23E-79	-1.09469828	0.022	0.278	5.48E-75	CD4 TCM
FRMD4B	1.11E-78	-0.876845546	0.012	0.223	2.73E-74	CD8 Naive
EPHA4	2.05E-77	-0.648092676	0.014	0.143	5.05E-73	CD14 Mono
RNF144A	5.58E-77	-0.671865947	0.064	0.214	1.37E-72	CD14 Mono
AHR	3.31E-76	-0.928243772	0.1	0.353	8.15E-72	CD4 Naive
DPP4	3.54E-76	-0.575012049	0.017	0.145	8.72E-72	CD14 Mono
MAST4	3.57E-76	-0.625150252	0.026	0.16	8.79E-72	CD14 Mono
BMP2K	4.60E-76	-0.957545029	0.052	0.311	1.13E-71	CD4 TCM
ZFHX3	7.40E-76	-0.805666537	0.027	0.246	1.82E-71	CD4 Naive
RIN2	8.93E-76	-0.823007347	0.013	0.221	2.20E-71	CD8 Naive
CYTOR	1.45E-75	-0.713130738	0.023	0.235	3.56E-71	CD8 Naive
SAMSN1	1.61E-75	-0.875339767	0.143	0.395	3.97E-71	CD8 Naive
ENOSF1	4.21E-75	-0.663636817	0.084	0.239	1.04E-70	CD14 Mono
ZFHX3	4.24E-75	-0.788700885	0.031	0.247	1.04E-70	CD8 Naive
RABGEF1	5.29E-75	-0.754020078	0.224	0.514	1.30E-70	CD4 Naive
TSPAN5	8.99E-75	-0.645308596	0.035	0.172	2.21E-70	CD14 Mono
PPM1L	4.12E-74	-0.943392322	0.038	0.255	1.01E-69	CD8 Naive
MAN1A1	1.64E-73	-0.801754865	0.09	0.336	4.04E-69	CD4 Naive
RIPK2	4.49E-73	-1.048871772	0.063	0.32	1.11E-68	CD4 TCM
CDK14	9.22E-73	-0.871747873	0.01	0.208	2.27E-68	CD8 Naive
RIN2	9.23E-73	-0.837459713	0.016	0.22	2.27E-68	CD4 Naive
TRPS1	2.59E-72	-1.020353146	0.247	0.489	6.39E-68	CD8 Naive
EPB41L3	1.98E-71	-0.765673367	0.029	0.24	4.86E-67	CD4 Naive
RGS2	3.17E-71	-1.114792885	0.093	0.345	7.81E-67	CD4 TCM
LPCAT2	9.28E-71	-0.734607973	0.011	0.206	2.28E-66	CD8 Naive
MICAL2	1.34E-70	-0.750630037	0.024	0.23	3.29E-66	CD4 Naive
EPB41L3	2.36E-70	-0.837400534	0.01	0.237	5.80E-66	CD4 TCM
WDFY4	4.82E-70	-0.929143952	0.006	0.229	1.19E-65	CD4 TCM
GLUL	1.67E-69	-0.648583734	0.013	0.206	4.12E-65	CD8 Naive
MAN1A1	1.97E-68	-0.777814646	0.101	0.336	4.85E-64	CD8 Naive
SETBP1	5.42E-68	-0.967157199	0.006	0.19	1.33E-63	CD8 Naive
SYTL2	5.57E-68	-0.600454656	0.024	0.146	1.37E-63	CD14 Mono
ZSWIM6	1.01E-67	-1.08902667	0.302	0.538	2.48E-63	CD4 TCM
MYC	1.03E-67	-0.541458194	0.041	0.171	2.53E-63	CD14 Mono
C9orf72	4.66E-67	-0.874646454	0.219	0.462	1.15E-62	CD4 Naive
SETBP1	2.80E-66	-0.972805398	0.006	0.189	6.90E-62	CD4 Naive
CLMN	3.04E-66	-0.715435013	0.003	0.182	7.49E-62	CD8 Naive
CENPK	3.08E-66	-0.497003696	0.023	0.142	7.59E-62	CD14 Mono
PTPRK	1.91E-65	-0.651474046	0.018	0.132	4.71E-61	CD14 Mono
LPCAT2	2.15E-65	-0.711628646	0.016	0.204	5.30E-61	CD4 Naive
FRMD4B	5.52E-65	-0.798177291	0.026	0.22	1.36E-60	CD4 Naive
PPM1L	6.03E-64	-0.841715499	0.048	0.253	1.48E-59	CD4 Naive

RIN2	1.73E-63	-0.839193743	0.009	0.216	4.26E-59	CD4 TCM
CDK14	2.16E-63	-0.832977493	0.02	0.206	5.31E-59	CD4 Naive
BMP2K	7.81E-63	-0.724844905	0.089	0.312	1.92E-58	CD4 Naive
CD96	4.98E-62	-1.77640324	0.103	0.466	1.23E-57	CD16 Mono
IL15	9.58E-61	-0.918209872	0.068	0.295	2.36E-56	CD4 TCM
ZFHX3	2.15E-60	-0.782748096	0.03	0.24	5.28E-56	CD4 TCM
SAMD12	2.36E-60	-0.58582194	0.017	0.124	5.80E-56	CD14 Mono
RETREG1	2.50E-60	-0.681324148	0.131	0.272	6.15E-56	CD14 Mono
EXT1	5.40E-60	-0.908165356	0.202	0.426	1.33E-55	CD8 Naive
JUN	2.13E-59	-0.860255422	0.506	0.708	5.25E-55	CD4 Naive
AP003086.1	7.58E-59	-0.865333619	0.082	0.311	1.87E-54	CD4 TCM
MPP6	8.95E-59	-0.479151112	0.015	0.117	2.20E-54	CD14 Mono
GAB1	2.78E-58	-0.59699034	0.005	0.166	6.84E-54	CD8 Naive
BIRC3	3.10E-58	-0.667679473	0.137	0.275	7.63E-54	CD14 Mono
AC010275.1	3.18E-57	-0.439161585	0.012	0.111	7.82E-53	CD14 Mono
CDK14	4.12E-57	-0.855527572	0.011	0.202	1.01E-52	CD4 TCM
ANXA2R	7.61E-57	-0.465989805	0.038	0.151	1.87E-52	CD14 Mono
CMSS1	2.58E-56	-0.629858186	0.143	0.284	6.36E-52	CD14 Mono
LPCAT2	2.80E-56	-0.721079776	0.011	0.2	6.90E-52	CD4 TCM
PPM1L	7.13E-56	-0.916238705	0.044	0.248	1.75E-51	CD4 TCM
RAB30	1.54E-55	-0.442804332	0.015	0.114	3.80E-51	CD14 Mono
LEF1-AS1	1.93E-55	-0.526445399	0.022	0.125	4.76E-51	CD14 Mono
EXT1	2.54E-55	-0.991065009	0.19	0.42	6.25E-51	CD4 TCM
STK3	2.54E-55	-0.780332661	0.07	0.285	6.26E-51	CD4 TCM
CLMN	2.58E-55	-0.663743373	0.016	0.179	6.34E-51	CD4 Naive
RPS6	2.97E-55	-0.99908536	0.951	0.932	7.32E-51	CD16 Mono
GAB1	3.07E-55	-0.598895097	0.007	0.165	7.56E-51	CD4 Naive
RGS18	5.19E-55	-0.514505503	0.016	0.178	1.28E-50	CD8 Naive
EXT1	7.04E-54	-0.808178826	0.203	0.425	1.73E-49	CD4 Naive
SETBP1	7.18E-54	-0.735887406	0.073	0.196	1.77E-49	CD14 Mono
SAMSN1	2.20E-53	-0.688351764	0.169	0.39	5.40E-49	CD4 Naive
TIAM1	2.53E-53	-0.660667362	0.113	0.316	6.24E-49	CD8 Naive
RNF144B	3.18E-53	-0.627676929	0.022	0.184	7.83E-49	CD8 Naive
LMO4	7.58E-53	-0.45460758	0.059	0.251	1.87E-48	CD4 Naive
CEP78	1.05E-52	-0.490965871	0.035	0.14	2.57E-48	CD14 Mono
SETBP1	1.40E-51	-0.917498842	0.009	0.184	3.44E-47	CD4 TCM
ATP6V1B2	1.53E-51	-0.472699731	0.066	0.255	3.77E-47	CD4 Naive
CYTOR	2.08E-51	-0.543724776	0.051	0.23	5.11E-47	CD4 Naive
RNF144B	2.63E-51	-0.617025007	0.023	0.183	6.47E-47	CD4 Naive
RABGEF1	4.38E-51	-0.704835987	0.29	0.504	1.08E-46	CD8 Naive
PRAG1	5.93E-51	-0.516576466	0.049	0.157	1.46E-46	CD14 Mono
CLMN	8.23E-51	-0.682998349	0.006	0.176	2.03E-46	CD4 TCM
FAM198B-AS1	2.18E-50	-0.548910929	0.001	0.14	5.37E-46	CD8 Naive
AC253572.2	2.37E-50	-0.573964671	0.063	0.245	5.83E-46	CD4 Naive
PRKCE	5.04E-50	-0.775296897	0.216	0.447	1.24E-45	CD4 TCM
HOPX	6.46E-50	-0.45376474	0.016	0.106	1.59E-45	CD14 Mono
CCDC141	1.58E-49	-0.430759849	0.017	0.107	3.90E-45	CD14 Mono
GLUL	2.46E-49	-0.540021437	0.037	0.201	6.06E-45	CD4 Naive
LINC02432	2.48E-49	-0.518916485	0.005	0.144	6.12E-45	CD8 Naive
MCTP2	3.30E-49	-0.750764929	0.088	0.275	8.12E-45	CD4 Naive

LINC01184	4.31E-49	-0.506427406	0.08	0.199	1.06E-44	CD14 Mono
BMP2K	7.93E-49	-0.688072948	0.12	0.308	1.95E-44	CD8 Naive
ABLIM1	8.13E-49	-1.456984895	0.082	0.39	2.00E-44	CD16 Mono
PALM2-AKAP2	4.77E-48	-0.521916011	0.035	0.133	1.18E-43	CD14 Mono
SNTB1	9.04E-48	-0.715068507	0.276	0.5	2.23E-43	CD4 TCM
PIP5K1B	1.76E-47	-0.573175278	0.008	0.144	4.33E-43	CD8 Naive
GAB1	3.28E-47	-0.593383082	0.003	0.161	8.08E-43	CD4 TCM
ARHGAP31	3.35E-47	-0.464786681	0.02	0.167	8.24E-43	CD8 Naive
ATP6V1B2	8.98E-47	-0.458453879	0.076	0.254	2.21E-42	CD8 Naive
TRPS1	3.00E-46	-0.884436799	0.27	0.478	7.38E-42	CD4 TCM
TRPS1	3.95E-46	-0.745721098	0.271	0.484	9.71E-42	CD4 Naive
MCTP2	1.22E-45	-0.790831655	0.101	0.274	3.00E-41	CD8 Naive
RNF144B	1.25E-45	-0.646669216	0.017	0.179	3.08E-41	CD4 TCM
LINC02432	5.79E-45	-0.50779706	0.009	0.143	1.42E-40	CD4 Naive
GTDC1	7.79E-45	-0.527483258	0.068	0.241	1.92E-40	CD4 Naive
MOB3B	3.39E-44	-0.583082814	0.015	0.172	8.33E-40	CD4 TCM
MAF	4.10E-44	-0.437817147	0.025	0.112	1.01E-39	CD14 Mono
RGS18	4.55E-44	-0.4469542	0.029	0.176	1.12E-39	CD4 Naive
RGS18	1.28E-43	-0.500139533	0.016	0.174	3.16E-39	CD4 TCM
MOB3B	3.75E-43	-0.525928266	0.032	0.174	9.24E-39	CD8 Naive
MOB3B	2.22E-42	-0.49116176	0.031	0.174	5.46E-38	CD4 Naive
FAM198B-AS1	7.52E-42	-0.519622331	0.011	0.138	1.85E-37	CD4 Naive
GALNT10	1.17E-41	-0.496290189	0.12	0.305	2.87E-37	CD4 Naive
DISC1	4.85E-41	-1.124067272	0.16	0.477	1.19E-36	NK
ARHGAP31	8.32E-41	-0.414861075	0.028	0.165	2.05E-36	CD4 Naive
PIP5K1B	1.37E-40	-0.524515221	0.015	0.142	3.37E-36	CD4 Naive
FILIP1L	1.60E-40	-0.554015209	0.088	0.191	3.95E-36	CD14 Mono
MIR646HG	6.30E-40	-0.609329534	0.057	0.204	1.55E-35	CD8 Naive
AC253572.2	7.92E-40	-0.531332471	0.084	0.242	1.95E-35	CD8 Naive
TBC1D19	1.47E-39	-0.398753073	0.038	0.125	3.62E-35	CD14 Mono
GAB2	1.58E-39	-1.453538367	0.075	0.375	3.88E-35	NK
FAM198B-AS1	2.28E-39	-0.5330785	0.003	0.136	5.62E-35	CD4 TCM
NBPF15	6.38E-39	-0.377094598	0.034	0.117	1.57E-34	CD14 Mono
IMMP2L	6.50E-39	-0.550040388	0.407	0.52	1.60E-34	CD14 Mono
LINC02432	4.68E-38	-0.498718889	0.007	0.14	1.15E-33	CD4 TCM
COX10-AS1	6.48E-38	-0.412185239	0.062	0.156	1.60E-33	CD14 Mono
LCLAT1	6.66E-38	-0.486564237	0.096	0.198	1.64E-33	CD14 Mono
OSBPL3	7.54E-38	-0.416489921	0.089	0.253	1.86E-33	CD4 Naive
ATP6V1B2	8.86E-38	-0.489291118	0.079	0.249	2.18E-33	CD4 TCM
C9orf72	1.04E-37	-1.29456424	0.085	0.442	2.57E-33	CD8 TEM_2
PIP5K1B	1.18E-37	-0.55112192	0.008	0.14	2.90E-33	CD4 TCM
NR3C2	1.74E-37	-1.328545749	0.078	0.336	4.28E-33	CD16 Mono
SH3RF1	2.16E-37	-0.405509109	0.005	0.113	5.32E-33	CD8 Naive
PCNX2	3.25E-37	-0.503323416	0.156	0.262	7.99E-33	CD14 Mono
BASP1	4.18E-37	-0.404978682	0.009	0.12	1.03E-32	CD8 Naive
CPQ	1.03E-36	-0.422378722	0.254	0.466	2.54E-32	CD4 Naive
L3MBTL4	1.18E-36	-0.46558835	0.005	0.111	2.90E-32	CD8 Naive
OSBPL3	1.36E-36	-0.475330024	0.097	0.252	3.34E-32	CD8 Naive
RASGRP1	1.38E-36	-1.178077811	0.101	0.348	3.39E-32	CD16 Mono
FRMD4B	2.03E-36	-0.671545616	0.057	0.211	5.01E-32	CD4 TCM

DISC1	3.43E-36	-1.27278304	0.134	0.474	8.45E-32	CD8 TEM_2
L3MBTL4	3.61E-36	-0.468696677	0.004	0.111	8.89E-32	CD4 Naive
SGMS2	5.26E-36	-0.379692936	0.003	0.107	1.30E-31	CD8 Naive
ACPP	1.13E-35	-0.313028846	0.001	0.102	2.79E-31	CD8 Naive
DZIP3	1.31E-35	-0.365160814	0.044	0.128	3.23E-31	CD14 Mono
C9orf72	1.38E-35	-1.080047573	0.156	0.442	3.39E-31	NK
PALM2-AKAP2	2.00E-35	-0.512682428	0.013	0.123	4.91E-31	CD8 Naive
SH3RF1	2.22E-35	-0.40381495	0.006	0.112	5.46E-31	CD4 Naive
AC120193.1	2.38E-35	-0.527960767	0.028	0.103	5.87E-31	CD14 Mono
MIR646HG	3.01E-35	-0.511695757	0.061	0.203	7.40E-31	CD4 Naive
MCTP1	3.17E-35	-1.772354942	0.022	0.364	7.81E-31	CD8 TEM_1
IRAK3	4.79E-35	-1.451934146	0.068	0.342	1.18E-30	NK
UGGT2	4.80E-35	-0.355092133	0.009	0.115	1.18E-30	CD8 Naive
CACNA2D3	7.34E-35	-0.395242276	0.008	0.113	1.81E-30	CD8 Naive
AC253572.2	1.61E-34	-0.549413688	0.079	0.238	3.97E-30	CD4 TCM
DISC1	1.91E-34	-1.19648382	0.154	0.474	4.69E-30	Memory B
HS3ST3B1	1.91E-34	-0.381698606	0.036	0.112	4.69E-30	CD14 Mono
PCSK5	2.34E-34	-0.601670107	0.14	0.313	5.76E-30	CD4 TCM
DOCK4	2.46E-34	-0.417359495	0.01	0.114	6.05E-30	CD8 Naive
RALB	2.60E-34	-0.307954601	0.021	0.137	6.40E-30	CD4 Naive
BACH1	3.28E-34	-1.436196918	0.165	0.486	8.08E-30	CD8 TEM_2
LONRF1	4.31E-34	-0.37143366	0.036	0.159	1.06E-29	CD4 Naive
BIRC3	6.97E-34	-0.545200662	0.111	0.259	1.72E-29	CD8 Naive
KYNU	7.35E-34	-1.119939257	0.06	0.328	1.81E-29	NK
IRAK3	7.72E-34	-1.701642107	0.028	0.34	1.90E-29	CD8 TEM_2
MICAL2	1.01E-33	-0.571468897	0.068	0.219	2.48E-29	CD4 TCM
DISC1	1.09E-33	-1.210316546	0.154	0.473	2.69E-29	Intermediate B
MAN1A1	1.19E-33	-0.59227991	0.147	0.323	2.94E-29	CD4 TCM
MCTP1	1.34E-33	-1.685279514	0.051	0.364	3.30E-29	CD8 TEM_2
SGMS2	1.59E-33	-0.374137901	0.006	0.106	3.90E-29	CD4 Naive
ABCA1	1.59E-33	-0.367794737	0.004	0.101	3.91E-29	CD8 Naive
TEC	5.06E-33	-0.380946832	0.027	0.143	1.25E-28	CD4 Naive
SLC2A9	8.44E-33	-0.39361969	0.025	0.138	2.08E-28	CD4 Naive
GAB2	8.87E-33	-1.688050233	0.046	0.372	2.18E-28	CD8 TEM_1
SPATA5	1.00E-32	-0.496896239	0.193	0.296	2.47E-28	CD14 Mono
ACPP	1.24E-32	-0.305159209	0.004	0.101	3.04E-28	CD4 Naive
IRAK3	1.37E-32	-1.712715531	0.019	0.339	3.37E-28	CD8 TEM_1
MCTP1	1.75E-32	-1.440809444	0.109	0.365	4.32E-28	NK
MRTFB	2.03E-32	-0.399876167	0.068	0.154	4.99E-28	CD14 Mono
KYNU	2.52E-32	-1.274820569	0.023	0.326	6.20E-28	CD8 TEM_2
C11orf65	2.64E-32	-0.417222258	0.085	0.172	6.49E-28	CD14 Mono
BACH1	2.82E-32	-1.443637342	0.16	0.485	6.93E-28	CD8 TEM_1
MPP7	3.49E-32	-1.0015719	0.16	0.387	8.60E-28	CD16 Mono
GAB2	5.36E-32	-1.563864578	0.065	0.372	1.32E-27	CD8 TEM_2
CALHM6	5.40E-32	-0.383778784	0.055	0.185	1.33E-27	CD4 Naive
KYNU	1.22E-31	-1.320929164	0.012	0.326	3.01E-27	CD8 TEM_1
MCTP2	1.32E-31	-0.716521345	0.114	0.267	3.24E-27	CD4 TCM
IGF1R	1.36E-31	-0.552354184	0.173	0.352	3.35E-27	CD4 TCM
RABGEF1	1.43E-31	-0.640986105	0.327	0.494	3.52E-27	CD4 TCM
BASP1	1.64E-31	-0.377090622	0.016	0.119	4.04E-27	CD4 Naive

FAM49A	1.67E-31	-1.560398315	0.042	0.382	4.12E-27	CD4 TEM
FMN1	1.80E-31	-0.401693294	0.008	0.102	4.43E-27	CD8 Naive
MAF	2.24E-31	-0.436961687	0.008	0.102	5.50E-27	CD8 Naive
DOCK4	2.86E-31	-0.408445589	0.013	0.113	7.05E-27	CD4 Naive
CACNA2D3	3.00E-31	-0.410631096	0.003	0.11	7.38E-27	CD4 TCM
UGGT2	3.50E-31	-0.339938945	0.014	0.114	8.63E-27	CD4 Naive
GCNT1	5.54E-31	-0.319948089	0.012	0.109	1.36E-26	CD8 Naive
CALHM6	6.93E-31	-0.412498908	0.06	0.184	1.70E-26	CD8 Naive
SNTB1	7.19E-31	-1.057221994	0.178	0.486	1.77E-26	Memory B
SLC2A9	8.26E-31	-0.380655945	0.029	0.138	2.03E-26	CD8 Naive
EPHA4	1.10E-30	-0.484671677	0.022	0.123	2.70E-26	CD8 Naive
L3MBTL4	1.32E-30	-0.462412964	0.003	0.108	3.25E-26	CD4 TCM
SNTB1	1.49E-30	-0.436600069	0.315	0.5	3.66E-26	CD4 Naive
EEF1A1	2.34E-30	-0.727944827	0.996	0.99	5.76E-26	NK
SH3RF1	3.05E-30	-0.404412253	0.004	0.11	7.50E-26	CD4 TCM
RALB	6.15E-30	-0.283313173	0.029	0.136	1.51E-25	CD8 Naive
CACNA2D3	6.34E-30	-0.366131475	0.014	0.111	1.56E-25	CD4 Naive
PIGK	6.39E-30	-0.344799104	0.061	0.14	1.57E-25	CD14 Mono
MCTP1	1.35E-29	-1.746052602	0.038	0.362	3.31E-25	CD4 TEM
SGMS2	1.05E-28	-0.368013637	0.003	0.103	2.58E-24	CD4 TCM
DOCK4	1.23E-28	-0.432425321	0.008	0.111	3.03E-24	CD4 TCM
IRS2	2.35E-28	-0.482573662	0.105	0.241	5.79E-24	CD4 Naive
PATJ	2.38E-28	-1.035797097	0.068	0.279	5.85E-24	CD16 Mono
MNDA	3.95E-28	-1.061829202	0.175	0.418	9.71E-24	NK
BASP1	4.14E-28	-0.395353246	0.012	0.116	1.02E-23	CD4 TCM
PTPRE	4.15E-28	-1.186069806	0.13	0.414	1.02E-23	Memory B
PTPRE	4.63E-28	-1.307530148	0.134	0.413	1.14E-23	Intermediate B
LRMP	5.74E-28	-0.373060365	0.16	0.321	1.41E-23	CD4 Naive
NR3C2	8.14E-28	-1.101827183	0.098	0.334	2.00E-23	NK
EMC4	8.88E-28	-0.296026903	0.059	0.133	2.18E-23	CD14 Mono
CPQ	9.38E-28	-0.503015717	0.289	0.456	2.31E-23	CD4 TCM
KYNU	1.00E-27	-1.320477471	0.017	0.324	2.47E-23	CD4 TEM
ZSWIM6	1.06E-27	-1.015166077	0.286	0.522	2.60E-23	NK
RSL24D1	1.19E-27	-0.360612305	0.157	0.246	2.92E-23	CD14 Mono
FMN1	1.23E-27	-0.386492664	0.012	0.101	3.03E-23	CD4 Naive
LRMP	1.48E-27	-0.45506487	0.157	0.317	3.63E-23	CD4 TCM
CD302	2.00E-27	-0.297885694	0.075	0.202	4.91E-23	CD4 Naive
CPPED1	2.07E-27	-1.040216684	0.107	0.347	5.10E-23	NK
CHPT1	2.39E-27	-0.31027178	0.083	0.211	5.88E-23	CD4 Naive
TNS3	2.45E-27	-0.875596249	0.047	0.27	6.02E-23	NK
TNS3	3.52E-27	-0.995339984	0.009	0.268	8.67E-23	CD8 TEM_2
CD302	3.82E-27	-0.312360629	0.078	0.202	9.41E-23	CD8 Naive
AC103591.3	4.67E-27	-0.381775299	0.04	0.142	1.15E-22	CD8 Naive
GCNT1	4.81E-27	-0.290929767	0.016	0.108	1.18E-22	CD4 Naive
NDUFAB1	4.96E-27	-0.317546902	0.091	0.172	1.22E-22	CD14 Mono
AC027097.2	7.04E-27	-0.349139897	0.118	0.26	1.73E-22	CD4 Naive
UGGT2	7.94E-27	-0.3440063	0.011	0.111	1.95E-22	CD4 TCM
CPQ	8.98E-27	-1.042711923	0.177	0.446	2.21E-22	Intermediate B
IRAK3	9.73E-27	-1.686255775	0.038	0.337	2.39E-22	CD4 TEM
CSGALNACT1	1.12E-26	-1.073933727	0.07	0.293	2.76E-22	NK

MCTP1	1.14E-26	-1.464946072	0.091	0.362	2.80E-22	Intermediate B
KLF4	1.16E-26	-0.999993418	0.062	0.288	2.86E-22	NK
KLF4	2.04E-26	-1.158849621	0.026	0.287	5.03E-22	CD8 TEM_2
CHPT1	2.14E-26	-0.319617876	0.087	0.211	5.26E-22	CD8 Naive
PVT1	2.45E-26	-0.868551773	0.092	0.321	6.03E-22	NK
CALHM6	2.63E-26	-0.408246757	0.057	0.181	6.48E-22	CD4 TCM
SLC2A9	2.88E-26	-0.401635704	0.027	0.135	7.09E-22	CD4 TCM
BBS9	3.54E-26	-0.471528564	0.223	0.31	8.71E-22	CD14 Mono
SAMD3	4.08E-26	-1.070117245	0.045	0.24	1.00E-21	CD16 Mono
TRMT11	5.60E-26	-0.358034523	0.077	0.153	1.38E-21	CD14 Mono
TEC	5.83E-26	-0.3450289	0.029	0.14	1.43E-21	CD4 TCM
TIAM1	5.86E-26	-1.02735625	0.04	0.296	1.44E-21	CD8 TEM_2
H2AFZ	6.65E-26	-0.3518541	0.142	0.226	1.64E-21	CD14 Mono
SMIM26	6.76E-26	-0.315910074	0.077	0.152	1.66E-21	CD14 Mono
MPP7	7.02E-26	-1.026777955	0.12	0.385	1.73E-21	Intermediate B
ZSWIM6	8.59E-26	-1.163451048	0.253	0.52	2.12E-21	CD8 TEM_2
BACH1	8.77E-26	-1.04855293	0.262	0.485	2.16E-21	NK
KIF13A	1.12E-25	-1.130066488	0.017	0.267	2.75E-21	CD8 TEM_2
GLUL	1.25E-25	-0.424509573	0.067	0.193	3.09E-21	CD4 TCM
KCNQ5	1.40E-25	-1.206767438	0.041	0.234	3.46E-21	CD16 Mono
CSGALNACT1	1.52E-25	-1.200801139	0.037	0.292	3.75E-21	CD8 TEM_2
CPPED1	1.92E-25	-1.263568552	0.068	0.344	4.72E-21	CD8 TEM_1
DOCK5	2.07E-25	-1.161286319	0.028	0.296	5.10E-21	CD8 TEM_1
HEG1	2.15E-25	-0.323512638	0.05	0.155	5.30E-21	CD8 Naive
AP003086.1	2.30E-25	-0.872096304	0.072	0.295	5.67E-21	NK
CPQ	2.50E-25	-0.419969233	0.302	0.459	6.14E-21	CD8 Naive
KLF4	3.03E-25	-1.192072021	0.022	0.286	7.45E-21	CD8 TEM_1
SSBP2	4.70E-25	-0.97285255	0.159	0.441	1.16E-20	CD8 TEM_2
GAS7	5.72E-25	-1.336327008	0.038	0.327	1.41E-20	CD4 TEM
ZNHIT6	6.14E-25	-0.347445703	0.095	0.172	1.51E-20	CD14 Mono
CSGALNACT1	6.62E-25	-1.094485476	0.099	0.293	1.63E-20	CD16 Mono
CD96	7.70E-25	-1.813413012	0.102	0.454	1.90E-20	cDC
RPS13	8.06E-25	-0.652583653	0.928	0.886	1.99E-20	CD16 Mono
MYOF	1.04E-24	-0.957254824	0.051	0.258	2.57E-20	NK
BACH1	1.21E-24	-1.388516152	0.215	0.483	2.97E-20	CD4 TEM
DPY30	1.21E-24	-0.283377191	0.066	0.137	2.99E-20	CD14 Mono
PCSK5	1.29E-24	-1.008490787	0.054	0.302	3.18E-20	Intermediate B
UBE3D	1.30E-24	-0.369992685	0.06	0.128	3.19E-20	CD14 Mono
LMO4	1.44E-24	-0.31082655	0.113	0.243	3.54E-20	CD8 Naive
GABPB1-AS1	1.46E-24	-0.445508309	0.154	0.235	3.59E-20	CD14 Mono
PID1	1.48E-24	-1.408000201	0.047	0.252	3.63E-20	NK
DISC1	1.49E-24	-0.534941803	0.33	0.479	3.67E-20	CD4 TCM
MNDA	2.19E-24	-1.260948727	0.145	0.416	5.38E-20	CD8 TEM_1
RTN1	2.72E-24	-1.064866326	0.051	0.257	6.69E-20	NK
SLC12A2	3.37E-24	-0.300379538	0.051	0.117	8.29E-20	CD14 Mono
DOCK5	3.43E-24	-1.198219464	0.017	0.295	8.45E-20	CD4 TEM
MPP7	4.06E-24	-0.899782277	0.13	0.385	9.98E-20	Memory B
C9orf72	4.12E-24	-1.110902989	0.17	0.438	1.01E-19	CD8 TEM_1
TRIO	5.34E-24	-1.112745258	0.052	0.336	1.31E-19	CD4 TEM
AHR	6.20E-24	-0.92217522	0.113	0.328	1.53E-19	NK

TNS3	7.02E-24	-0.968378196	0.015	0.267	1.73E-19	CD8 TEM_1
IRAK3	8.90E-24	-1.434636037	0.091	0.338	2.19E-19	Intermediate B
DLG2	8.95E-24	-0.475593653	0.04	0.101	2.20E-19	CD14 Mono
TAFA1	1.15E-23	-1.205810572	0.066	0.249	2.83E-19	CD16 Mono
MYOF	1.29E-23	-1.041038658	0.02	0.257	3.18E-19	CD8 TEM_2
IMMP2L	1.65E-23	-0.809070113	0.269	0.5	4.07E-19	NK
JUN	1.92E-23	-0.557580255	0.589	0.695	4.73E-19	CD8 Naive
ITGB3BP	2.53E-23	-0.341623205	0.08	0.152	6.24E-19	CD14 Mono
MYOF	2.84E-23	-1.086439377	0.012	0.257	6.98E-19	CD8 TEM_1
AP003086.1	2.84E-23	-0.978870952	0.048	0.293	6.99E-19	CD8 TEM_2
DOCK5	3.11E-23	-1.070818876	0.054	0.295	7.66E-19	Intermediate B
MCTP1	4.05E-23	-1.218376103	0.114	0.362	9.97E-19	Memory B
RTN1	7.88E-23	-1.202917898	0.026	0.256	1.94E-18	CD8 TEM_2
PCNX2	1.48E-22	-0.781450238	0.066	0.243	3.63E-18	CD16 Mono
RTN1	1.69E-22	-1.212540385	0.015	0.255	4.15E-18	CD8 TEM_1
PTPRE	1.77E-22	-1.321691358	0.146	0.411	4.36E-18	CD4 TEM
C9orf72	1.86E-22	-1.147455532	0.167	0.437	4.58E-18	CD4 TEM
JUN	1.90E-22	-0.911034439	0.615	0.684	4.68E-18	CD16 Mono
ARHGAP10	2.10E-22	-0.299098637	0.059	0.162	5.17E-18	CD4 Naive
AC103591.3	2.25E-22	-0.331850111	0.046	0.141	5.53E-18	CD4 Naive
EEF1A1	2.34E-22	-0.619632329	0.998	0.99	5.77E-18	CD16 Mono
KIF13A	2.50E-22	-1.023335875	0.022	0.266	6.17E-18	CD8 TEM_1
STK3	2.55E-22	-0.916751322	0.025	0.269	6.28E-18	CD8 TEM_1
BMP2K	2.66E-22	-0.914109676	0.04	0.29	6.55E-18	CD8 TEM_1
TIAM1	2.78E-22	-0.93804911	0.06	0.295	6.83E-18	Intermediate B
TXN	2.88E-22	-0.311013	0.096	0.21	7.10E-18	CD8 Naive
PVT1	3.55E-22	-0.794062751	0.071	0.319	8.75E-18	CD8 TEM_2
NT5DC1	3.62E-22	-0.389460593	0.142	0.219	8.90E-18	CD14 Mono
AC009226.1	4.92E-22	-0.28297721	0.032	0.117	1.21E-17	CD8 Naive
AP003086.1	5.83E-22	-0.967734378	0.043	0.293	1.43E-17	CD8 TEM_1
CEBPD	6.08E-22	-0.875087564	0.043	0.277	1.50E-17	CD8 TEM_2
PCSK5	6.35E-22	-0.86516806	0.126	0.302	1.56E-17	CD16 Mono
SSBP2	6.90E-22	-0.723282643	0.224	0.441	1.70E-17	NK
SMC4	8.23E-22	-0.280054517	0.087	0.196	2.02E-17	CD8 Naive
KLF4	1.51E-21	-1.154455046	0.028	0.285	3.72E-17	CD4 TEM
CPPED1	1.96E-21	-1.043082904	0.099	0.344	4.81E-17	CD8 TEM_2
PID1	2.13E-21	-1.581051412	0.031	0.25	5.24E-17	CD8 TEM_2
CSGALNACT1	2.20E-21	-0.4998975	0.177	0.3	5.42E-17	CD8 Naive
EPB41L3	2.67E-21	-0.731283424	0.041	0.22	6.57E-17	NK
CSGALNACT1	3.07E-21	-1.11057826	0.049	0.29	7.55E-17	CD8 TEM_1
IL15	3.07E-21	-0.834335253	0.083	0.279	7.56E-17	NK
SLC35F1	3.73E-21	-0.273648445	0.077	0.143	9.17E-17	CD14 Mono
RTN1	3.80E-21	-1.206491297	0.007	0.255	9.35E-17	CD4 TEM
AHR	4.37E-21	-0.993333215	0.091	0.326	1.08E-16	CD8 TEM_2
WDFY4	4.62E-21	-0.801506809	0.034	0.212	1.14E-16	NK
PCSK5	4.62E-21	-0.861562967	0.068	0.302	1.14E-16	CD8 TEM_2
LINC01934	5.18E-21	-1.141886006	0.105	0.277	1.27E-16	CD16 Mono
IGF1R	5.79E-21	-0.962647684	0.097	0.34	1.43E-16	CD8 TEM_2
KIF13A	5.85E-21	-1.099474002	0.017	0.266	1.44E-16	CD4 TEM
MGAT5	6.62E-21	-0.514768846	0.395	0.448	1.63E-16	CD14 Mono

BMP2K	8.11E-21	-0.707715951	0.092	0.291	2.00E-16	NK
MNDA	9.31E-21	-1.089747723	0.182	0.415	2.29E-16	CD8 TEM_2
DIMT1	1.06E-20	-0.315868746	0.092	0.16	2.61E-16	CD14 Mono
PTPRK	1.09E-20	-0.438966543	0.032	0.113	2.67E-16	CD4 Naive
AC027097.2	1.19E-20	-0.395751259	0.128	0.255	2.93E-16	CD4 TCM
PTPRK	1.23E-20	-0.482066835	0.025	0.112	3.03E-16	CD4 TCM
MAL	1.57E-20	-0.893787898	0.072	0.237	3.86E-16	CD16 Mono
TIAM1	1.70E-20	-0.882685557	0.078	0.295	4.18E-16	Memory B
RGS2	1.75E-20	-0.909874908	0.13	0.325	4.32E-16	NK
CD38	3.01E-20	-0.327136266	0.024	0.111	7.40E-16	CD4 TCM
DOCK5	3.45E-20	-0.958554515	0.078	0.295	8.48E-16	Memory B
FAM49A	3.94E-20	-1.070085199	0.13	0.38	9.69E-16	CD8 TEM_1
EPB41L3	4.55E-20	-0.774322293	0.014	0.219	1.12E-15	CD8 TEM_2
CHPT1	4.65E-20	-0.357025407	0.092	0.207	1.14E-15	CD4 TCM
SAMSN1	5.28E-20	-0.835141342	0.138	0.369	1.30E-15	Memory B
BMP2K	7.35E-20	-0.841058582	0.065	0.289	1.81E-15	CD8 TEM_2
TNS3	8.09E-20	-0.944567019	0.028	0.266	1.99E-15	CD4 TEM
EXT1	8.32E-20	-1.05150812	0.17	0.402	2.05E-15	CD8 TEM_2
MNDA	8.86E-20	-1.242763157	0.177	0.414	2.18E-15	CD4 TEM
LRMP	9.04E-20	-0.285468497	0.186	0.317	2.22E-15	CD8 Naive
GAB2	9.97E-20	-1.422559129	0.125	0.368	2.45E-15	CD4 TEM
TRIO	1.03E-19	-0.865623752	0.09	0.335	2.55E-15	CD8 TEM_1
LINC01934	1.04E-19	-1.112272484	0.065	0.276	2.55E-15	Memory B
WDFY4	1.09E-19	-0.85974237	0.011	0.211	2.68E-15	CD8 TEM_2
PID1	1.11E-19	-1.556737062	0.031	0.25	2.72E-15	CD8 TEM_1
RETREG1	1.13E-19	-0.782924434	0.066	0.243	2.79E-15	NK
RGS2	1.21E-19	-0.95829377	0.086	0.324	2.99E-15	CD8 TEM_1
WDFY4	1.48E-19	-0.884285443	0.003	0.211	3.65E-15	CD8 TEM_1
CEBPD	1.66E-19	-0.852814308	0.046	0.276	4.09E-15	CD8 TEM_1
PTPRE	1.71E-19	-1.109041292	0.176	0.411	4.21E-15	CD8 TEM_1
CDK14	2.47E-19	-0.77382232	0.026	0.188	6.08E-15	NK
EPB41L3	2.57E-19	-0.780738438	0.009	0.218	6.32E-15	CD8 TEM_1
PCSK5	2.60E-19	-0.860257836	0.092	0.301	6.40E-15	Memory B
EPSTI1	2.73E-19	-0.897118391	0.233	0.468	6.71E-15	CD8 TEM_2
EXT1	3.41E-19	-1.102821444	0.179	0.402	8.39E-15	Intermediate B
IRS2	3.76E-19	-0.83130768	0.028	0.229	9.27E-15	CD8 TEM_2
CD302	4.87E-19	-0.283662319	0.086	0.198	1.20E-14	CD4 TCM
RIPK2	7.12E-19	-0.976288684	0.071	0.298	1.75E-14	CD8 TEM_1
RIN2	7.16E-19	-0.793881709	0.009	0.199	1.76E-14	CD8 TEM_2
CREB3L2	8.79E-19	-0.275376064	0.063	0.151	2.16E-14	CD8 Naive
MAL	9.94E-19	-0.810736992	0.066	0.237	2.45E-14	NK
CD38	1.05E-18	-0.297683388	0.035	0.112	2.58E-14	CD8 Naive
GNPTAB	1.14E-18	-0.314459483	0.175	0.304	2.80E-14	CD4 Naive
DOCK5	1.31E-18	-0.892169942	0.077	0.295	3.22E-14	CD8 TEM_2
CPPED1	1.42E-18	-0.961684212	0.127	0.343	3.50E-14	Memory B
KCNQ5	1.56E-18	-0.582154544	0.126	0.237	3.84E-14	CD4 TCM
RTN1	1.56E-18	-1.068323259	0.051	0.255	3.85E-14	Intermediate B
PID1	1.68E-18	-1.360227364	0.046	0.25	4.14E-14	Intermediate B
SLC39A10	1.99E-18	-0.386049816	0.191	0.256	4.89E-14	CD14 Mono
EPB41L2	2.04E-18	-0.265354333	0.043	0.125	5.02E-14	CD4 Naive

EXT1	2.04E-18	-0.927866684	0.178	0.402	5.02E-14	Memory B
ZSWIM6	2.76E-18	-1.193228529	0.323	0.516	6.78E-14	CD4 TEM
PCSK5	2.83E-18	-0.771719384	0.074	0.301	6.95E-14	CD8 TEM_1
GAS7	2.91E-18	-0.934394548	0.114	0.327	7.15E-14	Memory B
ZFHX3	2.98E-18	-0.750600491	0.028	0.223	7.33E-14	CD8 TEM_2
PALM2-AKAP2	3.18E-18	-0.360293112	0.04	0.118	7.82E-14	CD4 Naive
PID1	3.19E-18	-1.606106297	0.028	0.249	7.86E-14	CD4 TEM
GAS7	3.73E-18	-0.98024671	0.111	0.327	9.18E-14	Intermediate B
CUBN	4.45E-18	-0.302653399	0.057	0.112	1.09E-13	CD14 Mono
FAM49A	5.63E-18	-1.48292463	0.043	0.377	1.39E-13	Treg
EXT1	5.84E-18	-1.06029622	0.17	0.402	1.44E-13	CD8 TEM_1
MYOF	7.22E-18	-1.023663933	0.035	0.255	1.78E-13	CD4 TEM
RIN2	1.11E-17	-0.785365268	0.006	0.198	2.73E-13	CD8 TEM_1
CD96	1.23E-17	-0.995083689	0.248	0.455	3.03E-13	Intermediate B
CDK14	1.53E-17	-0.818963565	0.009	0.186	3.76E-13	CD8 TEM_2
RPS6KA2	1.88E-17	-0.270868911	0.067	0.153	4.62E-13	CD8 Naive
CEP128	1.99E-17	-0.345094724	0.084	0.144	4.91E-13	CD14 Mono
EPB41L3	2.81E-17	-0.797495857	0.01	0.217	6.91E-13	CD4 TEM
AC027097.2	2.94E-17	-0.327042874	0.147	0.256	7.23E-13	CD8 Naive
SAMSN1	3.29E-17	-0.677173371	0.179	0.369	8.10E-13	NK
MYOF	3.34E-17	-0.814441573	0.065	0.256	8.23E-13	Memory B
RGS2	3.42E-17	-0.970416358	0.119	0.323	8.42E-13	CD8 TEM_2
ZFHX3	3.72E-17	-0.739444031	0.025	0.223	9.16E-13	CD8 TEM_1
TRIO	4.49E-17	-0.709499197	0.116	0.335	1.11E-12	CD8 TEM_2
IRS2	4.71E-17	-0.393405275	0.136	0.237	1.16E-12	CD8 Naive
PVT1	4.72E-17	-0.688866356	0.111	0.318	1.16E-12	Memory B
CPPED1	4.81E-17	-1.120803889	0.118	0.342	1.18E-12	CD4 TEM
ATP8B1	4.97E-17	-0.258758468	0.133	0.24	1.22E-12	CD8 Naive
SH3RF3	5.20E-17	-0.40013615	0.042	0.125	1.28E-12	CD4 TCM
GNPTAB	5.24E-17	-0.363920982	0.179	0.3	1.29E-12	CD4 TCM
ACYP2	5.45E-17	-0.417016012	0.23	0.297	1.34E-12	CD14 Mono
WDFY4	5.63E-17	-0.872668482	0.007	0.21	1.39E-12	CD4 TEM
SAMD3	6.98E-17	-0.957332947	0.054	0.237	1.72E-12	Memory B
MYOF	7.25E-17	-0.915810879	0.066	0.255	1.78E-12	Intermediate B
RIPK2	9.25E-17	-0.88027064	0.094	0.298	2.28E-12	CD8 TEM_2
SNTB1	9.81E-17	-0.391229727	0.379	0.49	2.41E-12	CD8 Naive
TAFA1	1.01E-16	-1.131906471	0.06	0.246	2.48E-12	Intermediate B
GATA3	1.02E-16	-0.590800599	0.019	0.148	2.52E-12	CD16 Mono
GAS7	1.10E-16	-0.983441994	0.111	0.326	2.70E-12	CD8 TEM_1
RIN2	1.17E-16	-0.672812407	0.047	0.199	2.89E-12	NK
EPB41L2	1.23E-16	-0.250630767	0.049	0.124	3.02E-12	CD8 Naive
CPPED1	1.25E-16	-1.027845526	0.148	0.342	3.06E-12	Intermediate B
RGS2	1.26E-16	-0.890034907	0.127	0.324	3.11E-12	Memory B
MCTP1	1.45E-16	-1.783446403	0.027	0.358	3.57E-12	gdT
FRMD4B	1.53E-16	-0.796889314	0.015	0.2	3.77E-12	CD8 TEM_1
KLF4	1.74E-16	-0.878195473	0.088	0.285	4.29E-12	Intermediate B
ABLIM1	1.87E-16	-1.383587944	0.122	0.38	4.60E-12	cDC
IL15	1.90E-16	-0.835358028	0.08	0.276	4.68E-12	CD8 TEM_2
LPCAT2	2.57E-16	-0.690601254	0.006	0.184	6.33E-12	CD8 TEM_1
SNTB1	2.60E-16	-0.849317532	0.253	0.481	6.41E-12	CD4 TEM

STK3	2.68E-16	-0.733193883	0.071	0.268	6.58E-12	CD8 TEM_2
LINC01934	3.03E-16	-1.106750208	0.085	0.275	7.46E-12	Intermediate B
BMP2K	3.13E-16	-0.849954792	0.069	0.288	7.69E-12	CD4 TEM
RNF144A	3.43E-16	-0.639782074	0.047	0.181	8.43E-12	CD16 Mono
IRAK3	3.43E-16	-1.677375447	0.03	0.334	8.44E-12	Treg
GALNT10	3.87E-16	-0.296426452	0.183	0.295	9.52E-12	CD8 Naive
LPCAT2	3.90E-16	-0.580951461	0.038	0.186	9.61E-12	NK
MAST4	4.47E-16	-0.557153444	0.01	0.13	1.10E-11	CD16 Mono
C1orf112	5.64E-16	-0.273564329	0.069	0.122	1.39E-11	CD14 Mono
MPP7	6.49E-16	-0.655268633	0.211	0.384	1.60E-11	NK
ZSWIM6	7.20E-16	-0.896446528	0.306	0.518	1.77E-11	CD8 TEM_1
TRPS1	9.98E-16	-1.494750331	0.117	0.459	2.46E-11	MAIT
EPSTI1	1.02E-15	-0.491048819	0.366	0.472	2.50E-11	CD4 TCM
DISC1	1.04E-15	-1.202287318	0.128	0.467	2.57E-11	gdT
RNF144B	1.05E-15	-0.59773834	0.03	0.167	2.59E-11	NK
KYNU	1.20E-15	-1.347472465	0.007	0.32	2.96E-11	gdT
TAFA1	1.22E-15	-0.906954678	0.092	0.247	3.00E-11	NK
RTN1	1.37E-15	-0.839473165	0.07	0.254	3.36E-11	Memory B
MGAT5	1.43E-15	-0.303026834	0.313	0.453	3.53E-11	CD4 Naive
BACH1	1.57E-15	-1.408075858	0.149	0.48	3.85E-11	gdT
MAL	1.59E-15	-0.850355004	0.06	0.235	3.92E-11	CD8 TEM_2
CEBPD	1.73E-15	-0.683818544	0.089	0.275	4.27E-11	Memory B
IRAK3	1.75E-15	-1.719084985	0.02	0.334	4.31E-11	gdT
RIPK2	2.21E-15	-0.753735863	0.139	0.298	5.43E-11	NK
KLF4	2.32E-15	-0.883928853	0.103	0.285	5.71E-11	Memory B
GAB2	2.56E-15	-1.335499366	0.055	0.367	6.30E-11	Treg
ACVR1	2.89E-15	-0.2509783	0.065	0.143	7.11E-11	CD8 Naive
CLMN	3.09E-15	-0.634147085	0.006	0.162	7.59E-11	CD8 TEM_2
RIN2	3.38E-15	-0.765720294	0.01	0.198	8.31E-11	CD4 TEM
IGF1R	3.67E-15	-0.779205762	0.127	0.339	9.02E-11	CD8 TEM_1
CPQ	3.74E-15	-0.653981053	0.232	0.444	9.20E-11	Memory B
TRIO	4.26E-15	-1.207448566	0.02	0.332	1.05E-10	gdT
KIAA0825	4.49E-15	-0.265286398	0.053	0.127	1.10E-10	CD4 Naive
KYNU	4.58E-15	-1.243084284	0.03	0.32	1.13E-10	Treg
EPSTI1	4.67E-15	-1.238886741	0.135	0.464	1.15E-10	gdT
EPSTI1	5.30E-15	-0.732023338	0.244	0.467	1.30E-10	CD8 TEM_1
SATB1-AS1	5.74E-15	-0.731233825	0.054	0.182	1.41E-10	CD16 Mono
TRPS1	6.02E-15	-0.836434303	0.303	0.462	1.48E-10	NK
BIRC3	6.80E-15	-0.631548447	0.092	0.246	1.67E-10	NK
GAS7	7.81E-15	-1.330971368	0.037	0.324	1.92E-10	Treg
TAFA1	8.43E-15	-1.05219457	0.078	0.246	2.07E-10	Memory B
KIAA0825	8.59E-15	-0.255498859	0.055	0.127	2.11E-10	CD8 Naive
AC103591.3	9.35E-15	-0.310509011	0.057	0.137	2.30E-10	CD4 TCM
LPCAT2	9.41E-15	-0.61819931	0.023	0.184	2.32E-10	CD8 TEM_2
CEBPD	9.98E-15	-0.747952455	0.094	0.275	2.46E-10	Intermediate B
AHR	1.13E-14	-0.815702154	0.141	0.325	2.79E-10	Memory B
CLMN	1.21E-14	-0.654064332	0.003	0.162	2.98E-10	CD8 TEM_1
AHR	1.49E-14	-0.548782618	0.229	0.33	3.66E-10	CD4 TCM
TIAM1	1.75E-14	-0.58932482	0.09	0.294	4.32E-10	CD8 TEM_1
XKR6	1.76E-14	-0.682640225	0.103	0.236	4.34E-10	CD16 Mono

MGAT5	2.39E-14	-0.342057793	0.332	0.451	5.88E-10	CD8 Naive
ZBTB20-AS5	2.48E-14	-0.267472027	0.063	0.111	6.09E-10	CD14 Mono
IL15	2.60E-14	-0.785794898	0.083	0.276	6.39E-10	CD8 TEM_1
SNTB1	3.07E-14	-0.933871329	0.152	0.48	7.57E-10	Treg
PTPRE	3.18E-14	-1.326156333	0.091	0.408	7.82E-10	Naive B
MOB3B	3.66E-14	-0.52263598	0.032	0.16	9.00E-10	NK
GAB2	3.88E-14	-1.629367547	0.051	0.366	9.56E-10	MAIT
RIN2	3.96E-14	-0.668564531	0.037	0.198	9.75E-10	Intermediate B
BTBD11	4.00E-14	-0.738385547	0.043	0.204	9.85E-10	CD8 TEM_2
MOB3B	4.07E-14	-0.574467522	0.011	0.16	1.00E-09	CD8 TEM_2
CDK14	4.14E-14	-0.763791266	0.022	0.186	1.02E-09	CD8 TEM_1
AP003086.1	4.58E-14	-0.796394601	0.087	0.291	1.13E-09	CD4 TEM
BACH1	5.00E-14	-1.426139075	0.168	0.479	1.23E-09	MAIT
DISC1	6.27E-14	-0.817596555	0.267	0.468	1.54E-09	CD4 TEM
DOCK5	6.59E-14	-1.236575147	0.007	0.291	1.62E-09	gdT
VOPP1	6.75E-14	-0.325945598	0.337	0.455	1.66E-09	CD8 Naive
LINC02432	7.04E-14	-0.447361801	0.013	0.13	1.73E-09	NK
SSBP2	7.41E-14	-1.083968942	0.122	0.436	1.82E-09	gdT
RASGRP1	7.72E-14	-0.739225216	0.165	0.342	1.90E-09	Memory B
MCTP1	7.80E-14	-1.45482021	0.079	0.358	1.92E-09	Treg
PRKN	7.89E-14	-0.736600247	0.102	0.248	1.94E-09	NK
RABGEF1	8.40E-14	-0.849599646	0.292	0.48	2.07E-09	CD4 TEM
RNF144B	8.64E-14	-0.605869957	0.017	0.166	2.13E-09	CD8 TEM_2
RGS18	9.10E-14	-0.493080485	0.014	0.161	2.24E-09	CD8 TEM_2
SSBP2	1.03E-13	-0.617662347	0.216	0.438	2.52E-09	CD8 TEM_1
RIPK2	1.08E-13	-1.113722574	0.024	0.295	2.66E-09	Treg
TSPAN5	1.20E-13	-0.56248599	0.029	0.141	2.96E-09	CD16 Mono
TRAT1	1.22E-13	-0.571701626	0.034	0.157	3.01E-09	NK
TIAM1	1.23E-13	-0.543479969	0.134	0.295	3.04E-09	NK
PRKCE	1.42E-13	-0.604067709	0.264	0.428	3.49E-09	NK
CPQ	1.42E-13	-1.104103871	0.133	0.441	3.49E-09	Naive B
PRKCE	1.78E-13	-0.685441118	0.233	0.427	4.37E-09	CD8 TEM_2
RGS2	2.16E-13	-0.90280075	0.128	0.322	5.33E-09	CD4 TEM
PPM1L	2.17E-13	-0.745312869	0.056	0.231	5.35E-09	CD8 TEM_1
TRPS1	2.18E-13	-1.321547215	0.161	0.459	5.36E-09	Naive B
TIAM1	2.18E-13	-1.078289213	0.014	0.291	5.37E-09	gdT
PVT1	2.29E-13	-0.92196837	0.027	0.314	5.64E-09	gdT
EPB41L2	2.60E-13	-0.251275636	0.049	0.122	6.39E-09	CD4 TCM
DOCK5	2.62E-13	-1.15276473	0.03	0.291	6.44E-09	Treg
RASGRP1	2.63E-13	-1.097449651	0.117	0.34	6.48E-09	cDC
SYNE1	2.68E-13	-0.938521954	0.368	0.435	6.60E-09	CD16 Mono
MOB3B	2.72E-13	-0.578696814	0.009	0.159	6.69E-09	CD8 TEM_1
FRMD4B	2.86E-13	-0.704119231	0.046	0.199	7.05E-09	Intermediate B
EPB41L3	2.93E-13	-0.622525435	0.062	0.217	7.22E-09	Memory B
PPM1L	3.06E-13	-0.681008121	0.068	0.231	7.54E-09	Memory B
CPPED1	3.31E-13	-1.284411371	0.054	0.34	8.15E-09	gdT
MARCH3	3.41E-13	-0.388514564	0.075	0.122	8.38E-09	CD14 Mono
MICAL2	3.51E-13	-0.627723218	0.057	0.207	8.65E-09	Memory B
RIPK2	3.55E-13	-0.742496107	0.124	0.297	8.75E-09	Memory B
GAB2	3.57E-13	-1.473217058	0.074	0.366	8.79E-09	gdT

FAM49A	3.66E-13	-1.374054578	0.073	0.376	9.01E-09	MAIT
EPB41L3	3.78E-13	-0.656864787	0.06	0.217	9.31E-09	Intermediate B
EXT1	3.78E-13	-0.66613779	0.243	0.402	9.31E-09	NK
PRKCE	3.86E-13	-0.999826572	0.128	0.426	9.50E-09	Treg
TRAT1	4.45E-13	-0.57532511	0.045	0.157	1.09E-08	CD16 Mono
GAB2	4.54E-13	-0.946960302	0.197	0.368	1.12E-08	Memory B
C9orf72	4.85E-13	-1.146439536	0.142	0.434	1.19E-08	gdT
DISC1	4.93E-13	-0.355880891	0.384	0.475	1.21E-08	CD8 Naive
MAL	5.08E-13	-0.743273747	0.074	0.234	1.25E-08	Intermediate B
EXT1	5.70E-13	-0.967820632	0.205	0.4	1.40E-08	CD4 TEM
CSGALNACT1	6.39E-13	-1.233473734	0.014	0.287	1.57E-08	gdT
CCT8	6.81E-13	-0.270848096	0.164	0.216	1.68E-08	CD14 Mono
MPP7	7.22E-13	-1.268124055	0.038	0.38	1.78E-08	pDC
CDK14	7.79E-13	-0.771998495	0.021	0.185	1.92E-08	CD4 TEM
STK3	8.72E-13	-0.594029801	0.1	0.267	2.15E-08	Memory B
BTBD11	8.86E-13	-0.742693557	0.054	0.204	2.18E-08	Intermediate B
MNDA	9.50E-13	-1.372403497	0.124	0.411	2.34E-08	MAIT
RETREG1	9.60E-13	-0.678378231	0.08	0.241	2.36E-08	CD8 TEM_2
KCNQ5	1.06E-12	-1.078617814	0.056	0.229	2.61E-08	CD4 TEM
PCSK5	1.32E-12	-0.920281045	0.037	0.298	3.26E-08	Treg
FRMD4B	1.34E-12	-0.632592367	0.051	0.199	3.30E-08	Memory B
C9orf72	1.44E-12	-0.80451836	0.288	0.434	3.53E-08	Intermediate B
PCNX2	1.45E-12	-0.542204514	0.102	0.241	3.56E-08	NK
AC253572.2	1.51E-12	-0.571011755	0.068	0.226	3.71E-08	CD8 TEM_2
MICAL2	1.51E-12	-0.620335038	0.057	0.207	3.73E-08	Intermediate B
PATJ	1.57E-12	-0.748202785	0.111	0.274	3.87E-08	CD8 TEM_2
GAB1	1.66E-12	-0.531142623	0.014	0.148	4.08E-08	CD8 TEM_2
EPHA4	1.66E-12	-0.532937578	0.016	0.114	4.08E-08	CD16 Mono
FAM198B-AS1	1.75E-12	-0.504915517	0	0.125	4.31E-08	CD8 TEM_2
RNF144A	1.90E-12	-0.603348415	0.041	0.18	4.66E-08	Memory B
NUCB2	1.91E-12	-0.649825216	0.077	0.232	4.71E-08	Intermediate B
MNDA	2.05E-12	-1.25599311	0.128	0.411	5.04E-08	gdT
KLF4	2.14E-12	-1.106949996	0.03	0.282	5.26E-08	Treg
TRPS1	2.18E-12	-0.910139405	0.281	0.46	5.37E-08	CD4 TEM
TGS1	2.20E-12	-0.285211244	0.193	0.244	5.41E-08	CD14 Mono
IL15	2.25E-12	-1.005879134	0.024	0.274	5.53E-08	Treg
IRAK3	2.33E-12	-1.578851257	0.051	0.333	5.73E-08	MAIT
LPCAT2	2.67E-12	-0.650294923	0.024	0.183	6.58E-08	CD4 TEM
ENOSF1	2.68E-12	-0.599482464	0.059	0.203	6.60E-08	Memory B
CD96	2.84E-12	-1.539018536	0.113	0.451	7.00E-08	pDC
BMP2K	3.04E-12	-0.984808763	0.02	0.286	7.47E-08	gdT
IMMP2L	3.17E-12	-0.671302201	0.315	0.496	7.80E-08	CD8 TEM_2
ATP6V1B2	3.18E-12	-0.454849496	0.074	0.235	7.83E-08	CD8 TEM_2
PRKN	3.25E-12	-0.714467217	0.085	0.247	7.99E-08	CD8 TEM_2
CLMN	3.37E-12	-0.529518578	0.045	0.162	8.30E-08	NK
TNS3	3.43E-12	-0.981773424	0.007	0.263	8.45E-08	gdT
IRAK3	3.46E-12	-1.545334336	0.063	0.333	8.52E-08	Naive B
STK3	3.66E-12	-0.713052908	0.087	0.266	9.01E-08	CD4 TEM
ATP8B1	4.06E-12	-0.308616264	0.143	0.235	9.99E-08	CD4 TCM
MCTP1	4.10E-12	-1.496757473	0.073	0.357	1.01E-07	MAIT

PPM1L	4.25E-12	-0.743392621	0.056	0.23	1.05E-07	CD4 TEM
ARHGAP10	4.33E-12	-0.500374773	0.047	0.153	1.07E-07	CD16 Mono
DPP4	4.43E-12	-0.47058014	0.019	0.116	1.09E-07	CD16 Mono
RGS18	4.78E-12	-0.4717547	0.019	0.16	1.18E-07	CD8 TEM_1
ICOS	4.87E-12	-0.559368108	0.031	0.132	1.20E-07	CD16 Mono
CPPED1	4.89E-12	-1.044340344	0.079	0.34	1.20E-07	Treg
SAMD3	4.96E-12	-0.85156671	0.085	0.235	1.22E-07	Intermediate B
MIR646HG	5.11E-12	-0.338155344	0.11	0.193	1.26E-07	CD4 TCM
USP53	5.39E-12	-0.514173941	0.041	0.144	1.33E-07	CD16 Mono
AC068587.4	5.71E-12	-0.664293946	0.148	0.312	1.41E-07	Intermediate B
RNF144B	6.04E-12	-0.55048747	0.022	0.166	1.49E-07	CD8 TEM_1
LINC02432	6.05E-12	-0.490510766	0	0.129	1.49E-07	CD8 TEM_1
GPHN	6.21E-12	-0.580912425	0.147	0.291	1.53E-07	NK
LPCAT2	6.27E-12	-0.552036445	0.046	0.184	1.54E-07	Memory B
TRIO	6.28E-12	-1.125436918	0.058	0.331	1.55E-07	MAIT
LPCAT2	6.29E-12	-0.595135485	0.043	0.184	1.55E-07	Intermediate B
AHR	6.32E-12	-0.840169579	0.171	0.323	1.56E-07	Intermediate B
STK3	7.06E-12	-0.541904057	0.128	0.267	1.74E-07	NK
MOB3B	7.11E-12	-0.578566528	0.01	0.159	1.75E-07	CD4 TEM
MCTP1	7.48E-12	-1.520674655	0.091	0.357	1.84E-07	Naive B
C9orf72	7.95E-12	-1.010581149	0.177	0.434	1.96E-07	Treg
ZNF827	8.04E-12	-0.485768419	0.029	0.128	1.98E-07	CD16 Mono
TRPS1	8.28E-12	-0.856449982	0.316	0.46	2.04E-07	Intermediate B
RIN2	8.58E-12	-0.598044817	0.057	0.197	2.11E-07	Memory B
MAL	9.11E-12	-0.695122523	0.086	0.234	2.24E-07	Memory B
DISC1	9.19E-12	-1.070160068	0.196	0.466	2.26E-07	Naive B
LRMP	1.08E-11	-0.594649662	0.118	0.304	2.65E-07	CD4 TEM
RNF144B	1.15E-11	-0.626884522	0.017	0.165	2.82E-07	CD4 TEM
GAB1	1.15E-11	-0.492928585	0.012	0.147	2.84E-07	CD8 TEM_1
RTN1	1.23E-11	-1.152601545	0.018	0.251	3.03E-07	Treg
PPM1L	1.29E-11	-0.807855124	0.085	0.23	3.17E-07	Intermediate B
CLMN	1.31E-11	-0.601671281	0.014	0.161	3.22E-07	CD4 TEM
RTN1	1.31E-11	-1.249736058	0.007	0.251	3.22E-07	gdT
RGS18	1.39E-11	-0.400103175	0.047	0.161	3.41E-07	NK
NFIA	1.39E-11	-0.548862734	0.024	0.151	3.43E-07	Memory B
BACH1	1.43E-11	-0.784622526	0.319	0.481	3.52E-07	Memory B
ZSWIM6	1.51E-11	-0.828560172	0.39	0.515	3.71E-07	Intermediate B
KIF13A	1.51E-11	-1.077566716	0.014	0.262	3.73E-07	gdT
PRKCE	1.52E-11	-0.825162524	0.257	0.426	3.75E-07	CD4 TEM
TRPS1	1.58E-11	-0.786246075	0.298	0.46	3.88E-07	CD8 TEM_2
KLF4	1.59E-11	-1.109720921	0.027	0.282	3.90E-07	gdT
KIF13A	1.64E-11	-1.068614286	0.03	0.262	4.04E-07	Treg
PID1	1.80E-11	-0.952798628	0.097	0.248	4.43E-07	Memory B
LINC02432	1.94E-11	-0.468331904	0.009	0.129	4.77E-07	CD8 TEM_2
TNS3	1.96E-11	-0.904472868	0.03	0.263	4.81E-07	Treg
MGAT5	2.04E-11	-0.77510467	0.385	0.437	5.03E-07	CD16 Mono
RPS6	2.12E-11	-0.494835288	0.932	0.933	5.21E-07	NK
IGF1R	2.16E-11	-1.11300176	0.137	0.336	5.31E-07	cDC
GLUL	2.17E-11	-0.494516836	0.037	0.183	5.33E-07	CD8 TEM_1
AHR	2.19E-11	-0.713971809	0.148	0.324	5.39E-07	CD8 TEM_1

SAMD3	2.21E-11	-1.064103146	0.036	0.234	5.45E-07	cDC
TFB1M	2.24E-11	-0.251593076	0.112	0.16	5.52E-07	CD14 Mono
PATJ	2.43E-11	-0.594695044	0.143	0.275	5.98E-07	NK
SETBP1	2.45E-11	-0.825042471	0.021	0.168	6.02E-07	CD4 TEM
CEBPD	2.65E-11	-0.68873882	0.101	0.273	6.52E-07	CD4 TEM
ATP6V1B2	2.78E-11	-0.4396727	0.071	0.235	6.83E-07	CD8 TEM_1
ABLIM1	3.09E-11	-1.442055905	0.075	0.378	7.61E-07	pDC
SAMSN1	4.02E-11	-1.184575275	0.057	0.363	9.90E-07	pDC
MYOF	4.16E-11	-1.054690589	0.014	0.252	1.03E-06	gdT
AP003086.1	4.51E-11	-0.626244297	0.135	0.29	1.11E-06	Memory B
STK3	4.64E-11	-0.583060067	0.108	0.266	1.14E-06	Intermediate B
PIP5K1B	4.74E-11	-0.51994218	0.011	0.129	1.17E-06	CD8 TEM_2
MCTP2	4.84E-11	-1.038545889	0.056	0.254	1.19E-06	cDC
GTDC1	4.94E-11	-0.26966093	0.14	0.227	1.22E-06	CD4 TCM
NR3C2	5.20E-11	-1.162382916	0.132	0.327	1.28E-06	cDC
MARCH3	5.20E-11	-0.326154658	0.053	0.117	1.28E-06	CD4 TCM
KYNU	6.15E-11	-1.077285978	0.051	0.319	1.51E-06	MAIT
PID1	6.44E-11	-1.647525915	0.014	0.246	1.58E-06	gdT
MPP7	6.49E-11	-0.503281548	0.202	0.382	1.60E-06	CD8 TEM_2
MPP7	6.67E-11	-0.960884939	0.119	0.38	1.64E-06	Naive B
NUCB2	6.92E-11	-0.642859111	0.13	0.232	1.70E-06	CD16 Mono
MYOF	7.44E-11	-0.999385302	0.03	0.252	1.83E-06	Treg
DOCK5	7.77E-11	-1.170486027	0.044	0.29	1.91E-06	MAIT
PRAG1	8.87E-11	-0.473181989	0.039	0.134	2.18E-06	CD16 Mono
RGS18	9.57E-11	-0.444589262	0.034	0.16	2.36E-06	Intermediate B
SLC2A9	9.67E-11	-0.441178023	0.011	0.127	2.38E-06	CD8 TEM_2
TRAT1	9.90E-11	-0.559113665	0.031	0.156	2.44E-06	Intermediate B
AP003086.1	9.90E-11	-0.91687493	0.041	0.288	2.44E-06	gdT
PCSK5	1.01E-10	-1.004543776	0.056	0.297	2.49E-06	Naive B
TRPS1	1.09E-10	-0.641042558	0.289	0.461	2.68E-06	Memory B
SLC2A9	1.11E-10	-0.393988247	0.028	0.127	2.73E-06	NK
MNDA	1.12E-10	-1.122594998	0.177	0.411	2.77E-06	Treg
RETREG1	1.22E-10	-0.663016011	0.1	0.24	3.01E-06	Intermediate B
PCSK5	1.26E-10	-0.941239069	0.054	0.297	3.09E-06	gdT
DISC1	1.27E-10	-0.86945019	0.207	0.466	3.14E-06	Treg
FAM198B-AS1	1.28E-10	-0.438118899	0.028	0.125	3.14E-06	NK
CEBPD	1.29E-10	-0.877326538	0.049	0.272	3.18E-06	Treg
FAM198B-AS1	1.52E-10	-0.475955281	0.006	0.124	3.74E-06	CD8 TEM_1
C9orf72	1.59E-10	-1.098109317	0.182	0.433	3.92E-06	MAIT
KIF13A	1.60E-10	-1.121925837	0.022	0.262	3.95E-06	MAIT
RIPK2	1.64E-10	-0.844717808	0.135	0.295	4.03E-06	CD4 TEM
BTBD11	1.66E-10	-0.622134506	0.073	0.203	4.08E-06	Memory B
LINC02432	1.79E-10	-0.448808496	0.014	0.128	4.40E-06	Intermediate B
RIPK2	1.83E-10	-1.058895658	0.044	0.294	4.50E-06	MAIT
CEMIP2	1.85E-10	-0.387462962	0.38	0.4	4.56E-06	CD14 Mono
TRPS1	1.90E-10	-0.898227816	0.195	0.459	4.67E-06	Treg
SATB1-AS1	2.00E-10	-0.641624299	0.051	0.18	4.92E-06	CD8 TEM_2
ZFHX3	2.11E-10	-0.43827143	0.098	0.222	5.20E-06	NK
FAM49A	2.25E-10	-0.711606998	0.224	0.378	5.54E-06	Memory B
BMP2K	2.37E-10	-0.934232998	0.036	0.285	5.83E-06	MAIT

RABGEF1	2.58E-10	-0.55048781	0.312	0.48	6.34E-06	CD8 TEM_2
SNTB1	2.62E-10	-1.216264136	0.198	0.478	6.46E-06	pDC
RGS2	2.76E-10	-0.759240761	0.177	0.321	6.80E-06	Intermediate B
ARHGAP31	2.79E-10	-0.344013754	0.045	0.151	6.86E-06	NK
SLC2A9	2.93E-10	-0.441180913	0.009	0.127	7.20E-06	CD8 TEM_1
PIP5K1B	3.03E-10	-0.450931261	0.032	0.129	7.47E-06	NK
EPB41L3	3.16E-10	-0.814644271	0	0.215	7.77E-06	gdT
EPSTI1	3.20E-10	-1.011121361	0.204	0.463	7.87E-06	MAIT
GAS7	3.20E-10	-1.134767259	0.073	0.323	7.88E-06	MAIT
NUCB2	3.35E-10	-0.526046607	0.095	0.232	8.25E-06	Memory B
IL15	3.36E-10	-0.776790544	0.118	0.274	8.28E-06	CD4 TEM
ZSWIM6	3.72E-10	-0.674771185	0.362	0.517	9.16E-06	Memory B
GAS7	4.21E-10	-0.752193962	0.176	0.324	1.04E-05	CD8 TEM_2
LEF1-AS1	4.23E-10	-0.444598492	0.019	0.102	1.04E-05	CD16 Mono
EPB41L3	4.32E-10	-0.764986337	0.012	0.215	1.06E-05	Treg
PIP5K1B	5.01E-10	-0.502025953	0.012	0.129	1.23E-05	CD8 TEM_1
RGS18	5.02E-10	-0.45337143	0.024	0.16	1.23E-05	CD4 TEM
GATA3	5.36E-10	-0.492269114	0.032	0.146	1.32E-05	Memory B
SH3RF1	5.85E-10	-0.340611763	0.015	0.102	1.44E-05	NK
EPSTI1	5.92E-10	-0.811641786	0.33	0.463	1.46E-05	CD4 TEM
ZHX2	6.21E-10	-0.957685959	0.193	0.364	1.53E-05	cDC
RPS6	6.26E-10	-0.871981931	0.962	0.933	1.54E-05	pDC
PID1	6.92E-10	-1.391791645	0.037	0.246	1.70E-05	Treg
MICAL2	7.10E-10	-0.507734339	0.065	0.206	1.75E-05	CD8 TEM_1
WDFY4	7.61E-10	-0.885256572	0	0.207	1.87E-05	gdT
CYTOR	7.78E-10	-0.447316114	0.078	0.211	1.92E-05	Memory B
LMO4	7.94E-10	-0.633744391	0.024	0.228	1.95E-05	Treg
RTN1	8.77E-10	-1.173736246	0.022	0.251	2.16E-05	MAIT
ZFHX3	8.79E-10	-0.745175597	0.018	0.22	2.16E-05	Treg
KLF4	9.04E-10	-1.04524992	0.044	0.281	2.22E-05	MAIT
ARHGAP10	9.47E-10	-0.505197167	0.037	0.152	2.33E-05	Intermediate B
BMP2K	1.04E-09	-0.807468635	0.067	0.285	2.57E-05	Treg
ABLIM1	1.06E-09	-0.700891526	0.236	0.38	2.61E-05	CD8 TEM_2
PATJ	1.16E-09	-0.981778527	0.096	0.272	2.84E-05	cDC
GAS7	1.20E-09	-1.131076478	0.091	0.323	2.96E-05	Naive B
CEMIP2	1.34E-09	-1.036328382	0.113	0.398	3.29E-05	pDC
CD302	1.42E-09	-0.351570888	0.079	0.19	3.50E-05	NK
MICAL2	1.44E-09	-0.552775581	0.111	0.206	3.55E-05	CD16 Mono
AHR	1.49E-09	-0.961335817	0.088	0.322	3.66E-05	gdT
MAL	1.60E-09	-0.930236358	0.061	0.232	3.94E-05	cDC
EPB41L2	1.63E-09	-0.431193138	0.014	0.117	4.01E-05	CD8 TEM_2
PTPRK	1.83E-09	-0.523679654	0.021	0.106	4.52E-05	NK
EXT1	1.86E-09	-1.083318427	0.162	0.398	4.59E-05	gdT
EEF1A1	1.87E-09	-0.815019772	1	0.99	4.60E-05	pDC
TNS3	1.87E-09	-0.891413974	0.036	0.262	4.61E-05	MAIT
MSRA	1.89E-09	-0.462037299	0.093	0.24	4.65E-05	CD8 TEM_1
IGF1R	1.93E-09	-0.929315798	0.095	0.335	4.75E-05	gdT
ZSWIM6	2.04E-09	-1.009245165	0.277	0.514	5.03E-05	gdT
DOCK4	2.27E-09	-0.411777702	0.006	0.103	5.59E-05	CD8 TEM_2
MIR646HG	2.27E-09	-0.625624005	0.066	0.188	5.59E-05	Intermediate B

IRS2	2.37E-09	-0.497850997	0.083	0.227	5.84E-05	CD8 TEM_1
ACVR2A	2.48E-09	-0.447701018	0.034	0.146	6.10E-05	Intermediate B
PIP5K1B	2.51E-09	-0.513238491	0.01	0.128	6.19E-05	CD4 TEM
FAM198B-AS1	2.73E-09	-0.443595192	0.02	0.124	6.72E-05	Intermediate B
RIPK2	2.73E-09	-0.948687558	0.068	0.294	6.72E-05	gdT
SSBP2	2.81E-09	-0.901898085	0.182	0.435	6.92E-05	MAIT
ICOS	3.16E-09	-0.492129621	0.041	0.131	7.78E-05	NK
MYOF	3.23E-09	-0.96591961	0.029	0.252	7.95E-05	MAIT
ACVR1	3.33E-09	-0.352794459	0.043	0.136	8.20E-05	NK
CACNA2D3	3.45E-09	-0.365496216	0.006	0.101	8.48E-05	CD8 TEM_2
PTPRE	3.61E-09	-0.993787527	0.195	0.407	8.89E-05	Treg
FAM241A	3.62E-09	-0.378692848	0.062	0.161	8.91E-05	NK
SATB1-AS1	3.89E-09	-0.632957781	0.063	0.179	9.57E-05	Intermediate B
RIN2	3.99E-09	-0.769239419	0.012	0.195	9.82E-05	Treg
IRS2	4.29E-09	-0.498195363	0.115	0.228	0.00010553	NK
LONRF1	4.48E-09	-0.350085551	0.034	0.146	0.000110286	CD8 TEM_2
AC068587.4	4.51E-09	-0.471552535	0.168	0.312	0.00011111	Memory B
EPB41L3	4.69E-09	-0.791958896	0.007	0.214	0.000115532	MAIT
DOCK5	4.71E-09	-1.038210888	0.077	0.29	0.000116038	Naive B
FAM198B-AS1	5.05E-09	-0.423481353	0.024	0.124	0.000124182	Memory B
AP003086.1	5.08E-09	-0.871337531	0.085	0.288	0.000125081	Treg
TAFA1	5.43E-09	-1.144608927	0.076	0.243	0.000133607	cDC
TEC	5.70E-09	-0.383521807	0.026	0.131	0.000140228	CD8 TEM_2
PPM1L	5.85E-09	-0.865665438	0.037	0.228	0.000143937	Treg
SH3RF3	6.10E-09	-0.534329966	0.02	0.119	0.000150017	CD8 TEM_2
TRIO	6.15E-09	-0.769271665	0.11	0.331	0.000151457	Treg
NFIA	6.28E-09	-0.435663511	0.055	0.151	0.000154535	NK
CPQ	6.28E-09	-0.502046482	0.295	0.442	0.000154611	CD8 TEM_2
WRN	6.41E-09	-0.278937358	0.15	0.191	0.000157778	CD14 Mono
RTN1	7.17E-09	-1.104252211	0.042	0.251	0.000176538	Naive B
LINC02432	7.24E-09	-0.449399876	0.014	0.128	0.000178287	CD4 TEM
CPPED1	7.32E-09	-1.080481347	0.119	0.339	0.000180169	Naive B
IRAK3	7.48E-09	-1.511067553	0.075	0.332	0.000184061	pDC
RGS2	8.25E-09	-0.912072543	0.11	0.32	0.000203026	Treg
CACNA2D3	8.31E-09	-0.329691487	0.021	0.102	0.000204497	NK
KLF4	8.61E-09	-1.040605332	0.07	0.281	0.000211905	Naive B
PALM2-AKAP2	8.79E-09	-0.45488603	0.033	0.111	0.000216246	CD16 Mono
MNDA	8.91E-09	-0.650338146	0.281	0.412	0.000219263	Memory B
FAM49A	9.41E-09	-0.670009071	0.239	0.377	0.000231591	CD8 TEM_2
TAFA1	1.00E-08	-1.069562782	0.035	0.243	0.000246458	Naive B
RIN2	1.02E-08	-0.779272653	0.007	0.195	0.000250878	gdT
ANXA2R	1.02E-08	-0.385688491	0.045	0.125	0.000251433	CD16 Mono
DISC1	1.06E-08	-0.941650287	0.248	0.465	0.00025995	MAIT
SH3RF1	1.08E-08	-0.347693695	0.009	0.101	0.000266462	CD8 TEM_2
ARHGAP10	1.16E-08	-0.479699552	0.049	0.152	0.000285176	Memory B
NR3C2	1.23E-08	-1.257830538	0.075	0.326	0.000302176	pDC
AC253572.2	1.25E-08	-0.409007872	0.115	0.225	0.000307389	NK
RETREG1	1.25E-08	-0.596944684	0.15	0.24	0.000308291	CD16 Mono
STK3	1.49E-08	-0.764673289	0.054	0.264	0.000366359	gdT
MIR646HG	1.51E-08	-0.593213672	0.076	0.187	0.000371072	Memory B

GLUL	1.70E-08	-0.418401318	0.07	0.183	0.000417848	Memory B
CEBPD	1.76E-08	-0.770211221	0.061	0.272	0.000432439	gdT
TRAT1	1.78E-08	-0.495198505	0.051	0.155	0.000437559	Memory B
CSGALNACT1	1.78E-08	-0.987871629	0.073	0.286	0.000438974	MAIT
FAM241A	1.90E-08	-0.384395117	0.043	0.161	0.000467965	CD8 TEM_1
PVT1	2.01E-08	-0.981199707	0.066	0.313	0.000494192	pDC
EPSTI1	2.03E-08	-0.499524286	0.348	0.465	0.000500217	NK
JUN	2.09E-08	-0.565190523	0.608	0.684	0.000515169	NK
CHPT1	2.18E-08	-0.323318049	0.094	0.198	0.000536501	NK
JUN	2.23E-08	-1.199698525	0.557	0.682	0.000549365	pDC
LINC01934	2.24E-08	-1.182466914	0.117	0.271	0.000550981	cDC
FRMD4B	2.25E-08	-0.472989191	0.077	0.198	0.000554855	CD8 TEM_2
AC009226.1	2.27E-08	-0.304389111	0.028	0.109	0.000558174	NK
GLUL	2.32E-08	-0.590954269	0.012	0.181	0.00057168	Treg
CD302	2.35E-08	-0.362350208	0.071	0.189	0.000578301	CD8 TEM_2
EPS8	2.36E-08	-0.261974237	0.023	0.102	0.000580904	NK
WDFY4	2.38E-08	-0.769173568	0.03	0.207	0.000584818	Treg
IRS2	2.42E-08	-0.562336399	0.105	0.227	0.000596824	Intermediate B
SYTL2	2.44E-08	-0.472953944	0.024	0.117	0.000600639	Memory B
TNS3	2.52E-08	-0.463424979	0.138	0.264	0.000620189	Memory B
ICOS	2.63E-08	-0.502122995	0.031	0.13	0.00064683	Intermediate B
SLC2A9	2.73E-08	-0.348346032	0.03	0.126	0.000672067	Memory B
AP003086.1	2.79E-08	-0.856319854	0.073	0.288	0.000687527	MAIT
DOCK4	2.81E-08	-0.370310138	0.026	0.103	0.000692116	NK
AC027097.2	2.99E-08	-0.39963799	0.105	0.245	0.00073481	CD8 TEM_1
SYTL2	3.22E-08	-0.484949396	0.023	0.117	0.000793467	Intermediate B
FAM241A	3.25E-08	-0.37664859	0.051	0.161	0.000800362	CD8 TEM_2
WWOX	3.43E-08	-0.364437822	0.474	0.507	0.000843371	CD14 Mono
PRKCE	3.84E-08	-0.874932706	0.197	0.424	0.000946247	MAIT
RGS2	3.87E-08	-0.922763725	0.108	0.319	0.000952137	gdT
BASP1	3.89E-08	-0.382554954	0.007	0.107	0.000956793	CD4 TEM
UGGT2	3.94E-08	-0.271149426	0.026	0.104	0.000969258	NK
CDK14	3.99E-08	-0.825576736	0	0.183	0.000982061	MAIT
SAMSN1	4.11E-08	-0.806495772	0.139	0.363	0.001011504	MAIT
BASP1	4.12E-08	-0.319832304	0.03	0.108	0.001013627	NK
DOCK4	4.12E-08	-0.386402618	0.009	0.102	0.00101464	CD8 TEM_1
UGGT2	4.60E-08	-0.317132208	0.014	0.103	0.001131	CD8 TEM_2
CHPT1	4.63E-08	-0.478731359	0.069	0.197	0.001140283	CD4 TEM
SH3RF1	4.68E-08	-0.374505874	0.003	0.101	0.001151745	CD4 TEM
CPPED1	4.68E-08	-0.993762975	0.124	0.339	0.001152039	MAIT
BTBD11	4.80E-08	-0.470107976	0.102	0.203	0.001180565	NK
SH3RF1	4.89E-08	-0.357397008	0.009	0.101	0.001204695	CD8 TEM_1
RIC8B	4.99E-08	-0.381297408	0.03	0.12	0.001228679	Memory B
TIAM1	5.10E-08	-0.79482745	0.084	0.29	0.001256381	Naive B
LPCAT2	5.13E-08	-0.660098897	0.007	0.181	0.001263555	gdT
TRAT1	5.17E-08	-0.626574207	0.015	0.154	0.001272675	cDC
SYNE1	5.43E-08	-1.097006303	0.189	0.434	0.001337232	pDC
LINC02432	5.44E-08	-0.405206107	0.035	0.128	0.001338711	Memory B
MNDA	5.50E-08	-1.277453133	0.189	0.409	0.001352494	pDC
CPQ	5.57E-08	-0.409328783	0.278	0.442	0.001369717	CD8 TEM_1

GAB1	5.66E-08	-0.453776745	0.035	0.146	0.001393438	CD4 TEM
MICAL2	5.82E-08	-0.711090694	0.021	0.204	0.001432764	Naive B
STK3	6.48E-08	-0.744459863	0.063	0.264	0.00159476	Naive B
BTBD11	6.52E-08	-0.818723242	0.021	0.201	0.001604253	Naive B
SYTL2	6.53E-08	-0.447150031	0.043	0.117	0.00160729	CD16 Mono
KIF13A	6.98E-08	-0.542262898	0.141	0.263	0.001718881	Memory B
RGS2	7.05E-08	-0.956462229	0.119	0.319	0.001735774	Naive B
WDFY4	7.37E-08	-0.842047906	0.022	0.207	0.001812943	MAIT
PCSK5	7.41E-08	-0.981599014	0.066	0.296	0.001823627	pDC
MNDA	7.47E-08	-0.995278667	0.21	0.41	0.001839365	Naive B
UGGT2	7.96E-08	-0.348302356	0.007	0.103	0.001960266	CD4 TEM
MGAT5	8.32E-08	-0.501192252	0.293	0.439	0.002048789	CD8 TEM_2
ZFHX3	8.39E-08	-0.686730474	0.034	0.219	0.002064761	gdT
GATA3	9.13E-08	-0.492795109	0.048	0.145	0.00224834	Intermediate B
RNF144B	9.16E-08	-0.656098546	0	0.163	0.002253558	gdT
FAM198B-AS1	9.24E-08	-0.455092941	0.021	0.124	0.002274035	CD4 TEM
JUN	9.33E-08	-0.435759088	0.557	0.685	0.002297064	CD8 TEM_2
LEF1-AS1	9.55E-08	-0.404586819	0.028	0.101	0.002350239	NK
BACH1	9.58E-08	-1.289227878	0.292	0.477	0.002359033	pDC
UGGT2	9.89E-08	-0.313071132	0.012	0.103	0.002433621	CD8 TEM_1
ZFHX3	1.01E-07	-0.54469267	0.094	0.22	0.002497174	CD4 TEM
MCTP2	1.02E-07	-0.342787271	0.221	0.26	0.002501895	CD14 Mono
CLMN	1.02E-07	-0.625690964	0.006	0.159	0.002513771	Treg
TRPS1	1.06E-07	-0.63959709	0.324	0.459	0.00260022	CD8 TEM_1
MARCH3	1.07E-07	-0.463028359	0.023	0.113	0.002630585	CD8 TEM_2
SH3RF3	1.08E-07	-0.505665868	0.017	0.118	0.002662927	CD4 TEM
PID1	1.08E-07	-1.337393673	0.056	0.246	0.002663082	Naive B
SETBP1	1.10E-07	-0.867087558	0.012	0.166	0.002710782	Treg
IRS2	1.22E-07	-0.740599617	0.042	0.225	0.002994431	Naive B
SYNE1	1.29E-07	-1.008687403	0.34	0.434	0.003186447	cDC
CD302	1.44E-07	-0.325636105	0.077	0.189	0.003556157	Intermediate B
IGF1R	1.51E-07	-0.42895486	0.318	0.337	0.003704894	CD14 Mono
AC027097.2	1.52E-07	-0.373903728	0.139	0.245	0.003745443	NK
RPS6KA2	1.62E-07	-0.361819678	0.04	0.144	0.003982019	CD8 TEM_1
RNF144A	1.62E-07	-0.514757063	0.077	0.178	0.003985632	Intermediate B
ARHGAP31	1.69E-07	-0.314184248	0.043	0.15	0.004171673	CD8 TEM_1
CACNA2D3	1.75E-07	-0.31220908	0.012	0.101	0.004316009	CD8 TEM_1
FAM241A	1.77E-07	-0.396746662	0.06	0.16	0.004352226	Intermediate B
RIPK2	1.79E-07	-0.690305809	0.185	0.295	0.004405136	Intermediate B
CSGALNACT1	1.80E-07	-0.557799533	0.165	0.287	0.004423752	Memory B
SLC2A9	1.94E-07	-0.37932229	0.034	0.126	0.004778067	Intermediate B
RNF144B	2.07E-07	-0.598402613	0.012	0.164	0.005098935	Treg
GATA3	2.08E-07	-0.567634696	0.015	0.144	0.005116268	cDC
LPCAT2	2.13E-07	-0.594350969	0.024	0.181	0.005246639	Treg
GLUL	2.15E-07	-0.40494326	0.077	0.182	0.005299659	Intermediate B
GAB1	2.19E-07	-0.332327892	0.062	0.147	0.005395058	NK
ARHGAP31	2.21E-07	-0.306582439	0.048	0.15	0.005439175	CD8 TEM_2
MYC	2.40E-07	-0.367590491	0.06	0.14	0.005896944	NK
AC027097.2	2.41E-07	-0.405805041	0.122	0.245	0.005936702	CD8 TEM_2
DPP4	2.43E-07	-0.402776011	0.03	0.115	0.005971167	Memory B

EXT1	2.47E-07	-0.93579376	0.196	0.397	0.006090527	Naive B
DPP4	2.51E-07	-0.385685229	0.041	0.115	0.006165735	NK
LRMP	2.52E-07	-0.39690883	0.17	0.303	0.006208685	CD8 TEM_2
GABPB1-AS1	2.56E-07	-0.525009635	0.142	0.218	0.006306453	CD16 Mono
ZFHX3	2.58E-07	-0.712909105	0.036	0.219	0.006356318	MAIT
AC009226.1	2.83E-07	-0.323824208	0.023	0.108	0.006953929	CD8 TEM_2
MCTP2	2.85E-07	-1.050265916	0.038	0.252	0.007023588	pDC
IL15	2.95E-07	-0.581713996	0.162	0.273	0.007271027	Intermediate B
RASGRP1	3.00E-07	-1.00703009	0.123	0.338	0.007385401	pDC
DISC1	3.01E-07	-0.504757543	0.327	0.467	0.00740529	CD8 TEM_1
MSRA	3.26E-07	-0.373808114	0.119	0.239	0.008020541	CD8 TEM_2
PTPRK	3.36E-07	-0.501712877	0.039	0.106	0.00826436	CD16 Mono
TXN	3.48E-07	-0.382556063	0.095	0.198	0.008573491	Memory B
BASP1	3.76E-07	-0.295259794	0.019	0.107	0.009261715	CD8 TEM_1
CDK14	3.92E-07	-0.743808114	0.02	0.183	0.009638403	gdT
PTPRK	3.93E-07	-0.539798528	0.014	0.105	0.009668917	CD4 TEM
CEBPD	3.98E-07	-0.719325986	0.084	0.271	0.00980494	Naive B
AC004889.1	NA	0	NA	NA	1	NA
AC026341.1	NA	0	NA	NA	1	NA
AC027018.1	NA	0	NA	NA	1	NA
AC046134.2	NA	0	NA	NA	1	NA
AC053527.2	NA	0	NA	NA	1	NA
AC073332.1	NA	0	NA	NA	1	NA
AC104389.5	NA	0	NA	NA	1	NA
ACAD11	NA	0	NA	NA	1	NA
ACADSB	NA	0	NA	NA	1	NA
ADPRM	NA	0	NA	NA	1	NA
AL356599.1	NA	0	NA	NA	1	NA
AL359232.1	NA	0	NA	NA	1	NA
AL513188.1	NA	0	NA	NA	1	NA
ALG9	NA	0	NA	NA	1	NA
AOPEP	NA	0	NA	NA	1	NA
AP1AR	NA	0	NA	NA	1	NA
ATG10	NA	0	NA	NA	1	NA
BMERB1	NA	0	NA	NA	1	NA
BMT2	NA	0	NA	NA	1	NA
C11orf54	NA	0	NA	NA	1	NA
C9orf85	NA	0	NA	NA	1	NA
CAAP1	NA	0	NA	NA	1	NA
CASS4	NA	0	NA	NA	1	NA
CCDC112	NA	0	NA	NA	1	NA
CDK8	NA	0	NA	NA	1	NA
CEMIP	NA	0	NA	NA	1	NA
CHROMR	NA	0	NA	NA	1	NA
CMAS	NA	0	NA	NA	1	NA
COQ7	NA	0	NA	NA	1	NA
CRNKL1	NA	0	NA	NA	1	NA
CROT	NA	0	NA	NA	1	NA
CXXC1	NA	0	NA	NA	1	NA
DNAJC15	NA	0	NA	NA	1	NA

EHBP1	NA	0	NA	NA	1	NA
EPM2A	NA	0	NA	NA	1	NA
EXOC6B	NA	0	NA	NA	1	NA
FAM114A2	NA	0	NA	NA	1	NA
FAM200B	NA	0	NA	NA	1	NA
FAM227B	NA	0	NA	NA	1	NA
FBXL3	NA	0	NA	NA	1	NA
FGGY	NA	0	NA	NA	1	NA
FKTN	NA	0	NA	NA	1	NA
FXN	NA	0	NA	NA	1	NA
GABPB1	NA	0	NA	NA	1	NA
GDE1	NA	0	NA	NA	1	NA
GNPAT	NA	0	NA	NA	1	NA
GSR	NA	0	NA	NA	1	NA
GSTCD	NA	0	NA	NA	1	NA
IARS2	NA	0	NA	NA	1	NA
IFT80	NA	0	NA	NA	1	NA
IKBIP	NA	0	NA	NA	1	NA
INTS2	NA	0	NA	NA	1	NA
INVS	NA	0	NA	NA	1	NA
IPO11	NA	0	NA	NA	1	NA
KCTD7	NA	0	NA	NA	1	NA
KCTD9	NA	0	NA	NA	1	NA
LANCL2	NA	0	NA	NA	1	NA
LINC00174	NA	0	NA	NA	1	NA
LINC00667	NA	0	NA	NA	1	NA
LINC01135	NA	0	NA	NA	1	NA
LINC01376	NA	0	NA	NA	1	NA
LYRM4	NA	0	NA	NA	1	NA
MAP3K7CL	NA	0	NA	NA	1	NA
MAPK8	NA	0	NA	NA	1	NA
MARCH6	NA	0	NA	NA	1	NA
MED19	NA	0	NA	NA	1	NA
MMADHC	NA	0	NA	NA	1	NA
MRPL13	NA	0	NA	NA	1	NA
MTERF1	NA	0	NA	NA	1	NA
NDUFAF2	NA	0	NA	NA	1	NA
NME9	NA	0	NA	NA	1	NA
NNT-AS1	NA	0	NA	NA	1	NA
NUBPL	NA	0	NA	NA	1	NA
ORC5	NA	0	NA	NA	1	NA
PDE5A	NA	0	NA	NA	1	NA
PPAT	NA	0	NA	NA	1	NA
PPP2CB	NA	0	NA	NA	1	NA
PSMD7	NA	0	NA	NA	1	NA
PTAR1	NA	0	NA	NA	1	NA
RAD17	NA	0	NA	NA	1	NA
RBM43	NA	0	NA	NA	1	NA
RRAGA	NA	0	NA	NA	1	NA
SEC22A	NA	0	NA	NA	1	NA

SIAH1	NA	0	NA	NA	1	NA
SIRT1	NA	0	NA	NA	1	NA
SLAIN1	NA	0	NA	NA	1	NA
SLC17A5	NA	0	NA	NA	1	NA
SLC30A6	NA	0	NA	NA	1	NA
SLC35D1	NA	0	NA	NA	1	NA
SMARCAL1	NA	0	NA	NA	1	NA
SMIM8	NA	0	NA	NA	1	NA
STX18-AS1	NA	0	NA	NA	1	NA
TAF9	NA	0	NA	NA	1	NA
THAP5	NA	0	NA	NA	1	NA
THYN1	NA	0	NA	NA	1	NA
TIAM2	NA	0	NA	NA	1	NA
TMEM267	NA	0	NA	NA	1	NA
TMEM38B	NA	0	NA	NA	1	NA
TRDMT1	NA	0	NA	NA	1	NA
TRIM52	NA	0	NA	NA	1	NA
TSTD3	NA	0	NA	NA	1	NA
TTC28-AS1	NA	0	NA	NA	1	NA
TXNL1	NA	0	NA	NA	1	NA
UTP20	NA	0	NA	NA	1	NA
UTP25	NA	0	NA	NA	1	NA
UTP3	NA	0	NA	NA	1	NA
VPS37A	NA	0	NA	NA	1	NA
WDR27	NA	0	NA	NA	1	NA
ZBTB2	NA	0	NA	NA	1	NA
ZDHHC13	NA	0	NA	NA	1	NA
ZFAND1	NA	0	NA	NA	1	NA
ZFP64	NA	0	NA	NA	1	NA
ANK3	0	-2.213038603	0.061	0.503	0	CD14 Mono
ANK3	0	1.669277251	0.899	0.321	0	CD4 TCM
ANK3	0	1.4913854	0.799	0.322	0	CD4 Naive
ANXA1	0	2.305306748	0.904	0.447	0	CD14 Mono
ATP8B4	0	1.040888075	0.441	0.072	0	CD14 Mono
BANK1	0	3.260678751	0.995	0.108	0	Memory B
BANK1	0	3.652664841	1	0.11	0	Intermediate B
BLK	0	2.475920744	0.895	0.069	0	Intermediate B
BLK	0	2.077618706	0.711	0.074	0	Memory B
CAMK4	0	-2.473911307	0.083	0.621	0	CD14 Mono
CAMK4	0	1.408504328	0.885	0.415	0	CD4 Naive
CAMK4	0	1.249190783	0.914	0.409	0	CD8 Naive
CST3	0	2.276886757	0.994	0.42	0	CD16 Mono
EBF1	0	3.113126642	0.958	0.065	0	Naive B
EBF1	0	2.755007402	0.872	0.049	0	Intermediate B
EBF1	0	2.517387959	0.768	0.052	0	Memory B
FGFBP2	0	1.914897091	0.659	0.051	0	CD8 TEM_2
FGFBP2	0	2.332954168	0.665	0.043	0	NK
HDAC9	0	1.71070409	0.807	0.24	0	CD14 Mono
INPP4B	0	-2.446204415	0.094	0.592	0	CD14 Mono
INPP4B	0	1.833524348	0.967	0.397	0	CD4 TCM

JCHAIN	0	2.489733953	0.896	0.046	0	pDC
LEF1	0	1.714334558	0.964	0.364	0	CD8 Naive
LEF1	0	1.533120221	0.929	0.372	0	CD4 Naive
LEF1	0	-2.483716473	0.085	0.577	0	CD14 Mono
LINC00926	0	2.961879625	0.895	0.051	0	Memory B
LINC00926	0	2.107381557	0.806	0.056	0	Intermediate B
LIX1-AS1	0	2.864730134	0.697	0.04	0	Memory B
LRMDA	0	3.125894573	0.977	0.123	0	CD14 Mono
LYN	0	1.808158963	0.989	0.323	0	CD14 Mono
MARCH1	0	1.867995119	0.942	0.247	0	CD14 Mono
NELL2	0	2.209387314	0.801	0.154	0	CD8 Naive
NRCAM	0	1.636259343	0.411	0.021	0	CD8 Naive
RALGPS2	0	3.41381238	0.994	0.169	0	Intermediate B
RALGPS2	0	2.606346853	0.954	0.169	0	Memory B
RORA	0	-2.059164723	0.076	0.547	0	CD14 Mono
THEMIS	0	1.745272613	0.906	0.332	0	CD8 Naive
THEMIS	0	-2.160167008	0.071	0.534	0	CD14 Mono
TXK	0	-1.982426497	0.118	0.536	0	CD14 Mono
TXK	0	1.466909088	0.852	0.357	0	CD8 Naive
ZBTB16	0	2.223606856	0.768	0.117	0	NK
ZEB2	0	2.440908077	0.994	0.347	0	CD14 Mono
CST3	2.15E-306	0.59738876	0.771	0.334	5.28E-302	CD14 Mono
AC139720.1	9.57E-298	1.660830248	0.502	0.12	2.35E-293	CD4 Naive
ZEB2	6.85E-297	-3.36062818	0.047	0.594	1.69E-292	CD8 Naive
CCSER1	1.46E-292	2.157860078	0.858	0.083	3.60E-288	cDC
CCSER1	6.25E-283	2.214346594	0.627	0.078	1.54E-278	Memory B
LYN	5.96E-271	-2.937070926	0.056	0.569	1.47E-266	CD8 Naive
ZEB2	1.63E-265	-3.01916158	0.068	0.588	4.00E-261	CD4 Naive
INPP4B	9.02E-263	1.208873301	0.855	0.399	2.22E-258	CD4 Naive
AL136456.1	2.44E-258	2.00037619	0.497	0.04	6.01E-254	CD4 TEM
LINC00926	2.76E-255	2.019686635	0.86	0.07	6.79E-251	Naive B
LYN	1.44E-250	1.906501649	0.998	0.472	3.55E-246	CD16 Mono
LYN	9.16E-243	-2.69324912	0.075	0.564	2.25E-238	CD4 Naive
BLK	8.84E-242	2.590221839	0.888	0.086	2.18E-237	Naive B
BCL11A	7.04E-240	2.079899417	0.759	0.15	1.73E-235	Memory B
SEL1L3	1.53E-230	2.074085715	0.816	0.186	3.76E-226	Memory B
ZFAT	4.28E-230	3.616582557	0.991	0.098	1.05E-225	pDC
ZEB2	5.30E-229	-3.304166882	0.058	0.576	1.30E-224	CD4 TCM
AK5	1.61E-227	1.352664265	0.451	0.124	3.97E-223	CD4 Naive
MLLT3	5.49E-223	1.385724829	0.711	0.348	1.35E-218	CD4 Naive
BANK1	2.57E-220	3.167972209	1	0.128	6.33E-216	Naive B
MLLT3	8.42E-219	-1.438064311	0.16	0.482	2.07E-214	CD14 Mono
LYN	2.75E-216	-2.926201556	0.053	0.554	6.76E-212	CD4 TCM
AC139720.1	4.23E-212	1.21527873	0.495	0.131	1.04E-207	CD4 TCM
CCSER1	5.25E-211	1.956793607	0.57	0.081	1.29E-206	Intermediate B
MARCH1	1.12E-210	-2.227161191	0.049	0.491	2.76E-206	CD8 Naive
TXK	5.46E-202	1.052431491	0.763	0.373	1.34E-197	CD4 Naive
ANXA1	1.49E-195	-2.253625324	0.229	0.622	3.67E-191	CD8 Naive
BCL11A	2.99E-195	1.607610164	0.738	0.152	7.36E-191	Intermediate B
HDAC9	7.03E-195	-2.26243152	0.026	0.448	1.73E-190	CD8 Naive

SEL1L3	4.30E-194	1.686472642	0.815	0.187	1.06E-189	Intermediate B
MARCH1	3.47E-182	-2.054147225	0.075	0.485	8.54E-178	CD4 Naive
NELL2	3.24E-179	-1.574864862	0.046	0.315	7.98E-175	CD14 Mono
HDAC9	3.99E-176	-2.125839379	0.039	0.444	9.81E-172	CD4 Naive
LRMDA	5.57E-172	-2.585664126	0.017	0.401	1.37E-167	CD8 Naive
MARCH1	1.01E-167	-2.201060956	0.047	0.478	2.48E-163	CD4 TCM
MLLT3	4.31E-159	0.878188784	0.7	0.348	1.06E-154	CD8 Naive
RORA	6.82E-158	1.093444685	0.751	0.381	1.68E-153	CD4 TCM
RALGPS2	3.50E-153	2.638274258	0.986	0.186	8.63E-149	Naive B
LRMDA	1.77E-147	-2.376882913	0.041	0.396	4.36E-143	CD4 Naive
HDAC9	2.33E-146	-2.13252816	0.036	0.434	5.73E-142	CD4 TCM
BCL11A	4.53E-146	3.18631414	0.991	0.163	1.12E-141	pDC
SEL1L3	5.46E-136	-1.148827451	0.047	0.266	1.34E-131	CD14 Mono
LRMDA	3.54E-135	-2.54561483	0.018	0.39	8.71E-131	CD4 TCM
TOX	4.12E-135	1.358145997	0.574	0.144	1.01E-130	NK
GLCCI1	5.42E-130	-1.019808112	0.106	0.338	1.34E-125	CD14 Mono
CST3	8.59E-130	2.916071397	0.99	0.438	2.11E-125	cDC
CAMK4	5.04E-127	0.725574079	0.845	0.432	1.24E-122	CD4 TCM
RORA	8.02E-124	1.739944311	0.948	0.408	1.97E-119	CD4 TEM
SEL1L3	1.18E-123	2.363524623	0.93	0.198	2.90E-119	Naive B
AC139720.1	6.01E-117	-1.245008768	0.031	0.222	1.48E-112	CD14 Mono
ZEB2	5.28E-114	0.9597878	0.998	0.492	1.30E-109	CD16 Mono
TOX	1.45E-112	1.351910017	0.594	0.149	3.58E-108	CD8 TEM_2
ANXA1	2.30E-108	-1.700357144	0.319	0.607	5.65E-104	CD4 Naive
HDAC9	3.04E-106	1.998523964	0.99	0.377	7.48E-102	cDC
MARCH1	1.56E-105	1.474946825	0.905	0.412	3.84E-101	Memory B
TTN	6.73E-103	-0.841905384	0.049	0.229	1.66E-98	CD14 Mono
AK5	8.69E-103	-0.980336766	0.039	0.215	2.14E-98	CD14 Mono
TXK	2.81E-102	1.041510587	0.885	0.404	6.91E-98	NK
ZBTB16	5.83E-99	2.034389608	0.745	0.138	1.43E-94	MAIT
AL589693.1	8.06E-99	-1.351813215	0.025	0.19	1.98E-94	CD14 Mono
ATP8B4	2.20E-98	1.115573903	0.746	0.158	5.42E-94	cDC
TOX	3.19E-98	-1.072571273	0.039	0.209	7.84E-94	CD14 Mono
TTN	2.17E-97	0.714296355	0.374	0.151	5.34E-93	CD8 Naive
AL136456.1	6.18E-93	1.816565592	0.438	0.048	1.52E-88	MAIT
LIX1-AS1	6.70E-90	1.246955532	0.319	0.054	1.65E-85	Intermediate B
JCHAIN	1.49E-89	0.649635695	0.293	0.046	3.68E-85	Intermediate B
SEL1L3	7.73E-89	1.947676135	0.934	0.201	1.90E-84	pDC
CST3	5.73E-87	-1.365683726	0.229	0.484	1.41E-82	CD8 Naive
MARCH1	3.37E-85	1.078371306	0.937	0.412	8.29E-81	Intermediate B
AK5	2.89E-83	0.607699113	0.348	0.14	7.12E-79	CD8 Naive
PDE4D	2.10E-79	0.734264044	0.447	0.263	5.17E-75	CD14 Mono
BLK	2.31E-73	-0.849947299	0.009	0.128	5.69E-69	CD14 Mono
LEF1	1.29E-70	0.340581244	0.777	0.406	3.18E-66	CD4 TCM
CDK6	2.40E-70	-0.836967431	0.184	0.347	5.91E-66	CD14 Mono
ATP8B4	7.53E-70	-0.753000098	0.006	0.195	1.85E-65	CD8 Naive
JCHAIN	3.71E-69	6.411562138	0.944	0.053	9.13E-65	Plasma
BCL11A	5.52E-69	-0.969755759	0.009	0.198	1.36E-64	CD8 Naive
CAMK4	1.48E-67	-2.080367586	0.115	0.498	3.64E-63	CD16 Mono
INPP4B	4.16E-65	-2.212753238	0.107	0.48	1.02E-60	CD16 Mono

CST3	1.87E-64	-1.324556372	0.242	0.475	4.59E-60	CD4 TCM
BCL11A	1.02E-63	1.574036628	0.671	0.165	2.52E-59	Naive B
ATP8B4	1.47E-63	-0.726850085	0.012	0.194	3.61E-59	CD4 Naive
AL136456.1	6.28E-63	2.089966482	0.335	0.048	1.55E-58	Treg
PDE4D	8.47E-63	2.524650759	0.818	0.304	2.09E-58	Naive B
RORA	3.98E-62	0.977733651	0.824	0.409	9.80E-58	CD8 TEM_2
CST3	5.01E-62	-1.114487185	0.244	0.481	1.23E-57	CD4 Naive
BCL11A	2.39E-61	-0.943468598	0.017	0.196	5.89E-57	CD4 Naive
TOX	7.75E-61	1.883250242	0.604	0.157	1.91E-56	Treg
AL589693.1	7.17E-60	0.883323321	0.286	0.124	1.76E-55	CD4 Naive
LRMDA	9.23E-60	1.031422153	0.964	0.336	2.27E-55	cDC
CAMK4	1.32E-59	-1.957641282	0.113	0.496	3.24E-55	NK
LEF1	1.91E-59	-2.129909854	0.109	0.466	4.70E-55	CD16 Mono
EBF1	3.66E-59	-1.021047176	0.006	0.103	9.00E-55	CD14 Mono
PDE4D	1.32E-58	1.430799876	0.632	0.299	3.25E-54	Memory B
THEMIS	2.25E-57	0.408738006	0.67	0.379	5.53E-53	CD4 TCM
USP28	2.58E-57	0.729364445	0.299	0.085	6.35E-53	NK
LINC00926	1.99E-56	-0.814255611	0.011	0.106	4.90E-52	CD14 Mono
ATP8B4	2.65E-56	-0.744928255	0.005	0.19	6.52E-52	CD4 TCM
BCL11A	3.91E-56	-0.962185662	0.007	0.193	9.62E-52	CD4 TCM
RORA	1.68E-55	1.600941501	0.956	0.416	4.13E-51	MAIT
RALGPS2	1.83E-55	-1.41313665	0.104	0.23	4.49E-51	CD14 Mono
RORA	4.47E-55	0.737142451	0.772	0.406	1.10E-50	NK
INPP4B	9.29E-55	0.957326359	0.924	0.448	2.29E-50	CD4 TEM
HDAC9	1.71E-53	0.952063718	0.751	0.376	4.21E-49	Memory B
AL589693.1	2.33E-53	0.655306507	0.297	0.127	5.74E-49	CD4 TCM
ANK3	8.57E-53	0.823108124	0.854	0.373	2.11E-48	CD4 TEM
MARCH1	9.25E-53	-1.829717062	0.09	0.446	2.28E-48	NK
RORA	1.03E-52	-1.755935786	0.123	0.439	2.55E-48	CD16 Mono
BCL11A	1.65E-52	0.663329562	0.609	0.163	4.07E-48	cDC
LYN	6.80E-52	-2.642123868	0.08	0.511	1.67E-47	CD8 TEM_1
TXK	6.90E-52	-1.695766492	0.115	0.442	1.70E-47	CD16 Mono
THEMIS	1.29E-51	-1.786537515	0.103	0.428	3.18E-47	CD16 Mono
ANK3	1.68E-51	-1.944618352	0.078	0.403	4.14E-47	CD16 Mono
ATP8B4	9.80E-51	0.932934168	0.405	0.158	2.41E-46	NK
INPP4B	2.78E-50	-1.772400725	0.126	0.477	6.85E-46	NK
BANK1	3.07E-50	-1.86803211	0.012	0.16	7.55E-46	CD4 Naive
ANXA1	3.80E-50	-1.898074655	0.171	0.582	9.36E-46	Intermediate B
MARCH1	6.30E-50	0.317892235	0.879	0.406	1.55E-45	CD16 Mono
BANK1	1.91E-49	-1.867062251	0.015	0.16	4.71E-45	CD8 Naive
LYN	2.13E-48	-2.777528416	0.069	0.51	5.25E-44	CD4 TEM
ZEB2	8.66E-48	-2.954512493	0.097	0.529	2.13E-43	CD4 TEM
TTN	2.49E-47	0.631810164	0.307	0.162	6.12E-43	CD4 Naive
FGFBP2	3.48E-47	0.978071876	0.378	0.067	8.57E-43	gdT
MARCH1	1.98E-46	-2.037235558	0.051	0.443	4.87E-42	CD8 TEM_2
MLLT3	2.16E-45	-1.493043689	0.115	0.412	5.32E-41	CD16 Mono
ZBTB16	8.23E-45	0.970713357	0.389	0.137	2.03E-40	Memory B
LYN	1.65E-44	0.676698852	0.905	0.483	4.07E-40	Memory B
THEMIS	6.12E-44	-1.647687994	0.109	0.427	1.51E-39	NK
PDE4D	8.72E-44	-1.128621508	0.167	0.335	2.15E-39	CD8 Naive

MARCH1	1.45E-43	-2.064385204	0.046	0.442	3.56E-39	CD8 TEM_1
ANXA1	3.67E-43	-1.779551599	0.216	0.581	9.04E-39	Memory B
GLCCI1	6.89E-43	0.809450399	0.542	0.265	1.70E-38	NK
INPP4B	3.95E-42	-2.096689479	0.114	0.473	9.71E-38	Intermediate B
LEF1	8.78E-42	-1.793749297	0.164	0.461	2.16E-37	NK
THEMIS	8.98E-41	0.298423663	0.604	0.382	2.21E-36	CD4 Naive
INPP4B	2.10E-40	-1.852018561	0.122	0.474	5.17E-36	Memory B
CAMK4	2.59E-40	-1.799581169	0.14	0.491	6.37E-36	Intermediate B
HDAC9	1.32E-39	1.364241645	0.953	0.383	3.26E-35	pDC
PDE4D	2.00E-39	0.472480158	0.691	0.3	4.92E-35	CD4 TEM
LEF1	1.87E-38	-1.958749241	0.114	0.46	4.61E-34	Intermediate B
LRMDA	2.35E-38	-2.423920162	0.02	0.359	5.80E-34	CD8 TEM_2
BANK1	3.82E-38	-1.789228229	0.017	0.156	9.40E-34	CD4 TCM
AK5	9.99E-38	0.86169273	0.38	0.159	2.46E-33	NK
CAMK4	1.46E-37	-1.528449902	0.138	0.492	3.58E-33	Memory B
TXK	1.90E-37	-1.712291663	0.103	0.437	4.69E-33	Intermediate B
LEF1	3.31E-37	-1.85884567	0.132	0.46	8.16E-33	Memory B
TXK	1.74E-35	-1.517636656	0.111	0.437	4.29E-31	Memory B
CCSER1	1.84E-35	-0.754653667	0.008	0.112	4.52E-31	CD8 Naive
LRMDA	3.54E-35	-2.347935347	0.019	0.358	8.71E-31	CD8 TEM_1
THEMIS	4.12E-35	-1.588908839	0.097	0.424	1.01E-30	Memory B
HDAC9	5.59E-35	0.534248684	0.749	0.376	1.38E-30	Intermediate B
MARCH1	1.59E-34	-1.967315947	0.083	0.44	3.91E-30	CD4 TEM
LYN	2.81E-33	0.435420615	0.926	0.483	6.92E-29	Intermediate B
USP28	3.35E-33	0.71554865	0.273	0.089	8.25E-29	CD8 TEM_2
TOX	4.06E-33	1.080494064	0.395	0.156	1.00E-28	CD8 TEM_1
THEMIS	4.17E-33	-1.661570239	0.111	0.423	1.03E-28	Intermediate B
ZBTB16	7.73E-33	-0.648955428	0.043	0.163	1.90E-28	CD8 Naive
RORA	1.18E-32	1.121848364	0.851	0.417	2.91E-28	gdT
TOX	4.10E-32	-0.764418912	0.058	0.181	1.01E-27	CD8 Naive
TOX	8.37E-32	1.39816857	0.5	0.159	2.06E-27	gdT
HDAC9	2.03E-31	-1.981678015	0.059	0.398	4.99E-27	CD4 TEM
ANK3	2.53E-31	-1.437161011	0.139	0.398	6.23E-27	NK
LRMDA	4.03E-31	-1.733531131	0.107	0.359	9.92E-27	NK
SEC11C	4.29E-31	2.978705937	0.889	0.11	1.06E-26	Plasma
CCSER1	6.13E-31	-0.715119475	0.013	0.111	1.51E-26	CD4 Naive
ZBTB16	7.02E-31	-0.611688211	0.045	0.162	1.73E-26	CD4 Naive
SPEF2	1.11E-30	0.355773263	0.161	0.072	2.73E-26	CD8 Naive
ANXA1	2.19E-29	0.440329197	0.98	0.56	5.39E-25	cDC
BANK1	2.29E-29	-0.673573411	0.462	0.134	5.63E-25	cDC
LRMDA	4.55E-29	-2.316435524	0.038	0.357	1.12E-24	CD4 TEM
RORA	1.72E-28	-1.323976909	0.151	0.433	4.23E-24	Memory B
BLK	2.07E-28	0.479271031	0.434	0.093	5.08E-24	pDC
MARCH1	3.17E-28	0.468411845	0.949	0.42	7.80E-24	cDC
CAMK4	3.18E-28	-2.194914399	0.096	0.486	7.82E-24	cDC
THEMIS	3.86E-28	0.767839066	0.698	0.403	9.49E-24	CD8 TEM_1
CCSER1	5.00E-28	1.301628419	0.769	0.096	1.23E-23	HSPC
MARCH1	7.81E-28	1.02787552	0.846	0.424	1.92E-23	Naive B
LIX1-AS1	1.07E-27	0.80777343	0.287	0.06	2.63E-23	Naive B
LYN	1.34E-27	-2.575885831	0.061	0.505	3.31E-23	Treg

MLLT3	2.38E-27	-1.348678774	0.131	0.406	5.85E-23	Intermediate B
THEMIS	1.39E-26	0.777625175	0.659	0.404	3.41E-22	CD8 TEM_2
LRMDA	1.64E-26	-1.760352216	0.083	0.357	4.03E-22	Intermediate B
TTN	3.31E-26	1.214281626	0.47	0.177	8.14E-22	Treg
JCHAIN	7.95E-26	0.611027093	0.252	0.052	1.96E-21	Naive B
LYN	8.58E-26	-1.741447874	0.295	0.505	2.11E-21	CD8 TEM_2
AL589693.1	8.64E-26	2.716208043	0.944	0.145	2.13E-21	Plasma
NELL2	9.96E-26	0.347735336	0.357	0.226	2.45E-21	CD4 Naive
NELL2	1.53E-25	-1.371954378	0.06	0.254	3.77E-21	CD16 Mono
NELL2	3.58E-25	0.740271873	0.491	0.236	8.81E-21	CD8 TEM_1
HDAC9	3.96E-25	-1.511140554	0.111	0.398	9.75E-21	CD8 TEM_1
ZEB2	3.99E-25	-2.657175441	0.122	0.524	9.83E-21	Treg
GLCCI1	4.57E-25	0.738934085	0.5	0.269	1.12E-20	CD8 TEM_2
MLLT3	5.61E-25	0.336900035	0.542	0.379	1.38E-20	CD4 TCM
PDE4D	6.07E-25	-0.615352439	0.186	0.331	1.49E-20	CD4 Naive
AL136456.1	6.70E-25	0.329086675	0.116	0.045	1.65E-20	CD4 TCM
CDK6	7.25E-25	0.707488302	0.576	0.296	1.78E-20	CD4 TEM
LEF1	9.80E-25	-1.533547289	0.207	0.456	2.41E-20	CD8 TEM_2
CST3	1.20E-24	0.878636356	0.887	0.444	2.95E-20	pDC
RORA	1.41E-24	-1.355047664	0.185	0.431	3.47E-20	Intermediate B
BLK	1.50E-24	-0.66391833	0.024	0.109	3.68E-20	CD8 Naive
PDE4D	1.79E-24	-0.834734406	0.189	0.327	4.40E-20	CD4 TCM
THEMIS	2.95E-24	1.044473366	0.777	0.407	7.25E-20	gdT
LEF1	6.21E-24	-2.160649128	0.102	0.455	1.53E-19	cDC
LRMDA	7.07E-24	-1.642574275	0.111	0.357	1.74E-19	Memory B
INPP4B	9.16E-24	-2.167871205	0.132	0.468	2.25E-19	cDC
NELL2	1.21E-23	-1.222333569	0.051	0.253	2.97E-19	NK
ANXA1	3.17E-23	-2.13912456	0.183	0.574	7.79E-19	Treg
SEC11C	3.58E-23	-0.283537077	0.062	0.129	8.81E-19	CD14 Mono
CDK6	8.07E-23	2.499608597	1	0.303	1.99E-18	HSPC
AC139720.1	1.23E-22	-0.628185145	0.082	0.187	3.03E-18	CD8 Naive
BLK	3.08E-22	-0.633437143	0.026	0.108	7.58E-18	CD4 Naive
AL589693.1	8.66E-22	0.670176424	0.351	0.141	2.13E-17	CD4 TEM
TSC22D1	2.01E-21	1.519630052	0.769	0.131	4.95E-17	HSPC
MARCH1	3.37E-21	-1.994774074	0.067	0.435	8.30E-17	Treg
MARCH1	3.54E-21	-2.128410526	0.041	0.435	8.72E-17	gdT
SEL1L3	8.93E-21	2.291186963	1	0.207	2.20E-16	Plasma
THEMIS	1.00E-20	-1.834307049	0.102	0.418	2.47E-16	cDC
GLCCI1	1.06E-20	1.2179732	0.555	0.273	2.61E-16	Treg
SEL1L3	2.54E-20	-0.57391374	0.116	0.223	6.25E-16	CD4 Naive
CST3	6.50E-20	-1.422570422	0.226	0.455	1.60E-15	CD4 TEM
TXK	7.00E-20	-1.138370829	0.202	0.434	1.72E-15	CD8 TEM_2
THEMIS	9.01E-20	0.399430964	0.736	0.403	2.22E-15	CD4 TEM
ANXA1	1.78E-19	-2.034644249	0.21	0.573	4.39E-15	Naive B
CDK6	2.29E-19	0.426738688	0.407	0.291	5.63E-15	CD4 TCM
RALGPS2	5.14E-19	-1.095753967	0.104	0.209	1.26E-14	CD4 TCM
CAMK4	1.35E-18	0.289751568	0.84	0.469	3.31E-14	CD4 TEM
BCL11A	1.67E-18	-0.870948558	0.023	0.179	4.11E-14	NK
TTN	1.83E-18	0.437974148	0.368	0.175	4.51E-14	Intermediate B
TOX	2.32E-18	-0.969981479	0.027	0.171	5.70E-14	CD16 Mono

LYN	3.31E-18	-2.304141238	0.189	0.502	8.15E-14	gdT
GLCCI1	3.92E-18	0.367370727	0.38	0.264	9.65E-14	CD4 TCM
RORA	5.12E-18	-1.669694523	0.162	0.428	1.26E-13	cDC
CST3	8.53E-18	-1.220233285	0.228	0.456	2.10E-13	CD8 TEM_1
INPP4B	9.12E-18	-1.979468393	0.112	0.466	2.24E-13	Naive B
LRMDA	1.03E-17	-2.229642394	0.024	0.353	2.54E-13	Treg
ZEB2	1.24E-17	0.339002923	0.959	0.509	3.06E-13	cDC
LEF1	1.45E-17	-2.083633988	0.098	0.453	3.58E-13	Naive B
ATP8B4	1.49E-17	2.018082404	0.731	0.168	3.67E-13	HSPC
GLCCI1	2.01E-17	-0.896944765	0.134	0.285	4.94E-13	CD16 Mono
LRMDA	3.29E-17	-2.43895582	0.014	0.353	8.11E-13	gdT
AK5	3.42E-17	-0.846172734	0.037	0.175	8.43E-13	CD16 Mono
AC139720.1	5.36E-17	-1.058313785	0.032	0.179	1.32E-12	NK
USP28	5.51E-17	0.340454154	0.225	0.09	1.36E-12	Intermediate B
SEL1L3	5.54E-17	-0.922696333	0.072	0.215	1.36E-12	CD16 Mono
CCSER1	5.60E-17	-0.561560103	0.059	0.112	1.38E-12	CD14 Mono
ANK3	6.32E-17	-1.857575622	0.127	0.392	1.56E-12	cDC
AC139720.1	8.32E-17	-1.042439335	0.041	0.179	2.05E-12	CD16 Mono
NELL2	8.90E-17	-1.192694672	0.06	0.251	2.19E-12	Intermediate B
MARCH1	1.25E-16	-1.847764095	0.08	0.434	3.09E-12	MAIT
HDAC9	2.31E-16	-1.584250926	0.073	0.394	5.70E-12	Treg
NELL2	2.85E-16	-1.167006635	0.068	0.251	7.01E-12	Memory B
THEMIS	4.37E-16	-1.782005557	0.084	0.417	1.08E-11	Naive B
BCL11A	4.65E-16	-0.900521923	0.011	0.177	1.14E-11	CD8 TEM_2
TOX	6.70E-16	0.881406914	0.808	0.162	1.65E-11	HSPC
ANXA1	6.73E-16	-2.113078569	0.198	0.571	1.66E-11	pDC
BCL11A	1.21E-15	-0.912040957	0.006	0.177	2.97E-11	CD8 TEM_1
USP28	2.09E-15	0.601446912	0.277	0.092	5.14E-11	gdT
BANK1	2.14E-15	-1.712903911	0.023	0.146	5.26E-11	CD16 Mono
ZEB2	3.53E-15	-1.579698003	0.407	0.521	8.68E-11	CD8 TEM_1
CAMK4	4.71E-15	-2.074417357	0.104	0.483	1.16E-10	pDC
TOX	5.69E-15	0.367998493	0.241	0.154	1.40E-10	CD4 TCM
LEF1	5.82E-15	-1.844049024	0.124	0.452	1.43E-10	MAIT
ANK3	1.06E-14	-1.11243951	0.216	0.393	2.62E-10	Memory B
TXK	1.37E-14	-1.524986097	0.208	0.43	3.36E-10	cDC
BLK	1.83E-14	0.398985638	0.299	0.094	4.50E-10	MAIT
LEF1	1.95E-14	-1.835854417	0.155	0.452	4.79E-10	gdT
BANK1	2.06E-14	-1.651215224	0.021	0.146	5.08E-10	NK
AL589693.1	2.25E-14	1.553969523	0.357	0.143	5.54E-10	Naive B
ZEB2	2.27E-14	-2.133986351	0.248	0.521	5.58E-10	MAIT
AL589693.1	2.30E-14	-1.077148354	0.026	0.152	5.65E-10	NK
LYN	3.03E-14	0.491990125	0.991	0.493	7.46E-10	pDC
CDK6	3.21E-14	-0.850528185	0.128	0.31	7.90E-10	Intermediate B
ATP8B4	3.28E-14	-0.705468787	0.003	0.174	8.07E-10	CD4 TEM
CST3	3.39E-14	-1.017888454	0.318	0.455	8.35E-10	NK
LEF1	5.00E-14	-2.014507733	0.075	0.452	1.23E-09	pDC
ATP8B4	5.40E-14	-0.660885939	0.023	0.174	1.33E-09	Intermediate B
TTN	6.07E-14	0.666451263	0.42	0.178	1.49E-09	Naive B
LYN	6.56E-14	-1.960943126	0.234	0.501	1.61E-09	MAIT
BCL11A	6.88E-14	-0.901366703	0.007	0.176	1.69E-09	CD4 TEM

RALGPS2	8.86E-14	-1.369343825	0.045	0.202	2.18E-09	CD8 TEM_2
AL589693.1	9.67E-14	-1.14393138	0.037	0.152	2.38E-09	CD16 Mono
NELL2	1.14E-13	-1.051067884	0.08	0.25	2.80E-09	CD8 TEM_2
PDE4D	1.17E-13	-1.057821252	0.187	0.318	2.88E-09	CD16 Mono
BANK1	1.64E-13	-1.75233421	0.006	0.145	4.04E-09	CD8 TEM_2
ATP8B4	1.85E-13	-0.620251884	0.03	0.174	4.55E-09	Memory B
TOX	2.06E-13	0.681371752	0.392	0.161	5.07E-09	Naive B
THEMIS	2.86E-13	0.604956146	0.752	0.408	7.05E-09	MAIT
THEMIS	3.03E-13	-1.899941426	0.066	0.416	7.45E-09	pDC
MLLT3	3.41E-13	-1.418183664	0.112	0.401	8.39E-09	Naive B
TTN	3.49E-13	-0.679489529	0.07	0.187	8.60E-09	CD16 Mono
CAMK4	4.36E-13	-1.660285944	0.224	0.483	1.07E-08	Naive B
TXK	5.63E-13	-1.595797487	0.161	0.429	1.39E-08	Naive B
BCL11A	5.81E-13	0.572494888	0.769	0.17	1.43E-08	HSPC
GLCCI1	6.33E-13	2.310558076	0.944	0.276	1.56E-08	Plasma
CST3	6.47E-13	-1.079512365	0.293	0.454	1.59E-08	CD8 TEM_2
CAMK4	9.48E-13	-1.062134572	0.352	0.484	2.33E-08	CD8 TEM_2
AL589693.1	9.61E-13	1.117421197	0.265	0.142	2.37E-08	Memory B
RORA	1.02E-12	0.658223337	0.677	0.419	2.52E-08	Treg
CCSER1	1.16E-12	-0.447374468	0.039	0.105	2.86E-08	CD4 TCM
AK5	1.19E-12	-0.817194791	0.031	0.173	2.93E-08	Intermediate B
PDE4D	1.45E-12	-1.102551487	0.151	0.317	3.58E-08	CD8 TEM_2
TTN	1.50E-12	-0.637061073	0.064	0.187	3.68E-08	NK
LRMDA	1.70E-12	-2.178303653	0.073	0.351	4.18E-08	MAIT
INPP4B	1.72E-12	0.654276351	0.72	0.457	4.23E-08	Treg
ATP8B4	1.79E-12	-0.606712991	0.025	0.174	4.40E-08	CD8 TEM_1
RORA	2.07E-12	-1.433947935	0.154	0.427	5.10E-08	Naive B
CST3	2.08E-12	-1.110361411	0.313	0.454	5.11E-08	Intermediate B
MLLT3	2.34E-12	-0.837440013	0.235	0.403	5.77E-08	Memory B
CAMK4	2.40E-12	0.296251123	0.716	0.471	5.90E-08	CD8 TEM_1
LRMDA	2.40E-12	-1.861240526	0.07	0.352	5.91E-08	Naive B
INPP4B	2.53E-12	-2.016765126	0.151	0.464	6.23E-08	pDC
ANK3	2.88E-12	-1.08241047	0.236	0.392	7.10E-08	CD8 TEM_2
TOX	4.15E-12	-0.880709935	0.035	0.169	1.02E-07	Memory B
THEMIS	4.22E-12	-1.236897491	0.146	0.417	1.04E-07	Treg
LEF1	4.76E-12	0.547239203	0.75	0.443	1.17E-07	Treg
AC139720.1	7.93E-12	-0.989257354	0.043	0.177	1.95E-07	Memory B
PDE4D	1.27E-11	-1.002530087	0.142	0.317	3.13E-07	CD8 TEM_1
PDE4D	1.37E-11	0.725097306	0.569	0.308	3.37E-07	MAIT
TXK	2.22E-11	-1.220478629	0.155	0.43	5.47E-07	gdT
AC139720.1	2.27E-11	0.268380748	0.333	0.167	5.58E-07	CD4 TEM
AL589693.1	2.32E-11	2.233160574	0.577	0.145	5.72E-07	HSPC
TXK	3.18E-11	-1.570400967	0.113	0.429	7.83E-07	pDC
ZBTB16	3.50E-11	-0.724690072	0.019	0.15	8.62E-07	CD8 TEM_1
AC139720.1	3.85E-11	-1.003201484	0.045	0.176	9.48E-07	CD8 TEM_2
ZBTB16	3.98E-11	-0.445163112	0.083	0.154	9.80E-07	CD4 TCM
AL589693.1	4.33E-11	-1.086975209	0.026	0.151	1.06E-06	CD8 TEM_2
RORA	5.50E-11	0.366242267	0.62	0.416	1.35E-06	CD8 TEM_1
AK5	6.17E-11	-0.780155287	0.037	0.173	1.52E-06	CD8 TEM_1
ANK3	6.19E-11	-1.90532704	0.094	0.39	1.52E-06	pDC

CST3	6.38E-11	-1.326820998	0.22	0.452	1.57E-06	Treg
NELL2	9.29E-11	-1.361170443	0.056	0.248	2.29E-06	cDC
CDK6	1.16E-10	0.398352123	0.356	0.296	2.86E-06	CD8 Naive
BLK	1.27E-10	-0.538287408	0.046	0.103	3.13E-06	CD4 TCM
AC139720.1	1.33E-10	-0.95967804	0.048	0.176	3.28E-06	Intermediate B
BANK1	2.50E-10	-0.693689569	0.387	0.138	6.15E-06	pDC
HDAC9	2.84E-10	-1.025062854	0.247	0.394	7.00E-06	CD8 TEM_2
BANK1	3.23E-10	-1.700939944	0.022	0.144	7.94E-06	CD8 TEM_1
BLK	5.73E-10	-0.678357724	0.019	0.101	1.41E-05	CD16 Mono
CAMK4	6.49E-10	0.491012821	0.75	0.475	1.60E-05	Treg
GLCCI1	6.78E-10	0.593312524	0.504	0.274	1.67E-05	MAIT
ZBTB16	9.70E-10	0.930259991	0.577	0.145	2.39E-05	HSPC
CST3	1.26E-09	-0.912962746	0.316	0.454	3.10E-05	Memory B
NELL2	1.35E-09	-1.194609003	0.043	0.247	3.31E-05	Treg
ATP8B4	1.58E-09	-0.488068316	0.051	0.173	3.88E-05	CD8 TEM_2
BLK	1.58E-09	-0.65598366	0.017	0.101	3.89E-05	NK
AK5	1.84E-09	-0.684394883	0.057	0.173	4.52E-05	Memory B
GLCCI1	1.91E-09	0.501400961	0.41	0.273	4.69E-05	CD8 TEM_1
CST3	2.25E-09	-1.377563324	0.248	0.451	5.54E-05	MAIT
SEC11C	3.81E-09	0.431130451	0.292	0.109	9.37E-05	pDC
BANK1	5.12E-09	-1.734112361	0.024	0.143	0.000125897	CD4 TEM
ANK3	5.35E-09	-0.996789286	0.159	0.39	0.000131586	Treg
ANXA1	5.76E-09	-1.03311122	0.46	0.571	0.000141842	CD8 TEM_1
ANK3	6.32E-09	-0.580610754	0.343	0.394	0.000155465	CD8 Naive
GLCCI1	6.85E-09	0.410059903	0.4	0.273	0.000168689	Memory B
AL589693.1	9.58E-09	-1.005351824	0.037	0.15	0.000235788	CD8 TEM_1
ANK3	9.83E-09	0.33057143	0.671	0.383	0.000241849	Naive B
RORA	1.41E-08	-1.52459684	0.208	0.425	0.000346066	pDC
BCL11A	1.43E-08	-0.910844828	0.006	0.174	0.000352534	Treg
CCSER1	1.45E-08	0.701402735	0.238	0.096	0.000355663	Naive B
ATP8B4	2.37E-08	-0.679431288	0.006	0.172	0.000582729	Treg
RALGPS2	2.61E-08	-1.313647677	0.024	0.2	0.000643593	Treg
BCL11A	2.90E-08	-0.918686693	0	0.174	0.000713938	gdT
USP28	3.15E-08	0.320132236	0.231	0.093	0.000776123	Naive B
RALGPS2	3.30E-08	-1.165701647	0.08	0.201	0.000812998	CD8 TEM_1
RALGPS2	3.95E-08	-0.863520781	0.146	0.205	0.000971456	CD4 Naive
RALGPS2	3.95E-08	-1.077179237	0.104	0.201	0.000972716	NK
AC139720.1	5.31E-08	-1.081025565	0.03	0.175	0.001306497	cDC
NELL2	8.51E-08	-1.242847547	0.056	0.247	0.00209473	Naive B
USP28	9.44E-08	0.302323782	0.5	0.094	0.002322568	Plasma
CST3	1.08E-07	-1.177679639	0.257	0.452	0.002668678	gdT
SEL1L3	2.41E-07	-0.418260111	0.161	0.216	0.005932334	CD8 Naive
PDE4D	3.02E-07	-0.584780326	0.122	0.314	0.007432133	Treg
MLLT3	3.33E-07	-0.589537616	0.303	0.402	0.008205964	NK
GLCCI1	3.37E-07	0.575565265	0.446	0.275	0.008284825	gdT
SPEF2	3.70E-07	0.255101413	0.385	0.083	0.009115182	HSPC
ABLIM1	0	1.315524293	0.778	0.31	0	CD8 Naive
AC120193.1	0	2.396455675	0.724	0.059	0	Memory B
AC253572.2	0	1.140627426	0.512	0.116	0	CD14 Mono
AHR	0	1.661695169	0.698	0.183	0	CD14 Mono

ANGPTL1	0	1.813201404	0.624	0.043	0	Intermediate B
AP003086.1	0	1.658499459	0.718	0.13	0	CD14 Mono
ATP6V1B2	0	0.845785268	0.489	0.137	0	CD14 Mono
BACH1	0	1.954734685	0.935	0.311	0	CD14 Mono
BMP2K	0	1.110231849	0.592	0.171	0	CD14 Mono
C9orf72	0	1.7317684	0.888	0.266	0	CD14 Mono
CALHM6	0	1.595915194	0.77	0.136	0	CD16 Mono
CCDC149	0	0.655960787	0.223	0.012	0	CD14 Mono
CEBPD	0	1.366338801	0.635	0.138	0	CD14 Mono
CLMN	0	1.295097966	0.463	0.047	0	CD14 Mono
CPPED1	0	1.902144929	0.83	0.159	0	CD14 Mono
CPQ	0	1.028651816	0.734	0.331	0	CD14 Mono
DISC1	0	1.259760589	0.806	0.339	0	CD14 Mono
DOCK4	0	0.960765799	0.293	0.03	0	CD14 Mono
DOCK5	0	1.601576638	0.748	0.122	0	CD14 Mono
EPB41L3	0	1.346233493	0.573	0.083	0	CD14 Mono
EPSTI1	0	1.46227082	0.817	0.332	0	CD14 Mono
EXT1	0	1.723292344	0.807	0.247	0	CD14 Mono
FAM198B-AS1	0	1.189578898	0.387	0.026	0	CD14 Mono
FAM49A	0	1.904405498	0.87	0.194	0	CD14 Mono
FMNL2	0	1.824160726	0.586	0.054	0	CD16 Mono
FRMD4B	0	1.414160353	0.513	0.08	0	CD14 Mono
GAB2	0	2.39844792	0.915	0.164	0	CD14 Mono
GAS7	0	2.060591841	0.84	0.133	0	CD14 Mono
GLUL	0	1.022985389	0.43	0.089	0	CD14 Mono
HOPX	0	1.702487459	0.578	0.059	0	NK
IL15	0	1.708518883	0.696	0.117	0	CD14 Mono
IRAK3	0	2.444202734	0.904	0.124	0	CD14 Mono
IRS2	0	1.188221727	0.489	0.127	0	CD14 Mono
KIF13A	0	1.957772476	0.738	0.087	0	CD14 Mono
KLF4	0	1.991826032	0.769	0.103	0	CD14 Mono
KYNU	0	1.703631355	0.766	0.155	0	CD14 Mono
LPCAT2	0	1.219986833	0.471	0.075	0	CD14 Mono
MCTP1	0	2.23028245	0.917	0.152	0	CD14 Mono
MCTP2	0	2.158821109	0.9	0.219	0	NK
MICAL2	0	1.355773096	0.547	0.078	0	CD14 Mono
MNDA	0	1.969228976	0.887	0.236	0	CD14 Mono
MYOF	0	1.545690113	0.641	0.109	0	CD14 Mono
PDGFD	0	1.905817207	0.55	0.035	0	NK
PID1	0	2.572244248	0.7	0.08	0	CD14 Mono
PRLR	0	0.870059359	0.237	0.012	0	CD14 Mono
PTPRE	0	2.09139768	0.916	0.221	0	CD14 Mono
PTPRK	0	1.218887368	0.383	0.057	0	CD8 Naive
RABGEF1	0	1.291714368	0.813	0.354	0	CD14 Mono
RGS2	0	1.804849009	0.754	0.16	0	CD14 Mono
RIN2	0	1.59529202	0.573	0.057	0	CD14 Mono
RIPK2	0	1.834511726	0.723	0.137	0	CD14 Mono
RTN1	0	1.912999262	0.709	0.083	0	CD14 Mono
SGMS2	0	0.874263049	0.292	0.021	0	CD14 Mono
SH3RF1	0	1.29397075	0.607	0.071	0	CD16 Mono

SLC2A9	0	0.975677675	0.351	0.041	0	CD14 Mono
SNTB1	0	1.190589443	0.8	0.359	0	CD14 Mono
SPRED1	0	1.259537734	0.576	0.055	0	CD16 Mono
STK3	0	1.513520723	0.663	0.117	0	CD14 Mono
TNS3	0	1.625572731	0.7	0.102	0	CD14 Mono
TRIO	0	1.073533314	0.683	0.201	0	CD14 Mono
TRPS1	0	1.798054622	0.859	0.311	0	CD14 Mono
ZFHX3	0	1.251706043	0.531	0.104	0	CD14 Mono
ZSWIM6	0	1.890094028	0.939	0.358	0	CD14 Mono
LINC02432	1.16E-301	0.815304795	0.326	0.053	2.84E-297	CD14 Mono
RGS18	6.34E-297	0.8212126	0.373	0.078	1.56E-292	CD14 Mono
SETBP1	3.23E-285	1.346038126	0.753	0.133	7.94E-281	CD16 Mono
LINC02432	4.15E-279	1.230179341	0.634	0.098	1.02E-274	CD16 Mono
RNF144B	1.17E-264	0.800465466	0.372	0.086	2.87E-260	CD14 Mono
CACNA2D3	9.10E-263	0.727363308	0.267	0.038	2.24E-258	CD14 Mono
RNF144B	1.09E-260	1.52995195	0.695	0.133	2.68E-256	CD16 Mono
MYOF	4.21E-259	1.392047877	0.885	0.216	1.04E-254	CD16 Mono
PRKCE	3.30E-253	0.815463167	0.703	0.32	8.13E-249	CD14 Mono
NR3C2	3.27E-252	1.213496615	0.69	0.264	8.05E-248	CD8 Naive
AC120193.1	6.58E-250	1.486487359	0.57	0.066	1.62E-245	Intermediate B
SETBP1	2.02E-244	1.876612322	0.792	0.142	4.97E-240	Intermediate B
SAMD3	3.04E-234	1.818375255	0.804	0.203	7.48E-230	NK
WDFY4	1.40E-233	1.87914206	0.835	0.182	3.46E-229	Intermediate B
YES1	7.29E-233	1.448594704	0.454	0.056	1.79E-228	NK
DOCK5	3.12E-231	1.38921736	0.899	0.255	7.68E-227	CD16 Mono
MITF	3.30E-231	0.646086362	0.188	0.016	8.13E-227	CD14 Mono
ACPP	4.11E-231	0.622955576	0.237	0.035	1.01E-226	CD14 Mono
PDGFD	1.19E-230	1.560925416	0.455	0.044	2.93E-226	CD8 TEM_2
CEP78	2.94E-227	1.445826886	0.559	0.091	7.24E-223	NK
AL163541.1	1.95E-226	1.035922759	0.18	0.014	4.80E-222	CD14 Mono
GAB1	3.59E-226	0.714339796	0.327	0.077	8.84E-222	CD14 Mono
IRF4	1.16E-223	2.224271832	0.726	0.045	2.84E-219	pDC
CACNA2D3	1.42E-223	1.489742609	0.766	0.085	3.50E-219	cDC
SASH1	4.86E-223	1.060486989	0.463	0.062	1.20E-218	CD16 Mono
IGFBP7	7.58E-218	1.375332725	0.422	0.053	1.87E-213	NK
MOB3B	1.55E-216	0.758056037	0.341	0.088	3.82E-212	CD14 Mono
EPS8	3.32E-212	0.965452439	0.502	0.077	8.16E-208	CD16 Mono
KCNQ5	1.05E-210	1.231113401	0.53	0.174	2.59E-206	CD8 Naive
MARCH3	2.33E-209	1.926911827	0.578	0.092	5.74E-205	Memory B
CD96	1.05E-207	1.102820618	0.783	0.393	2.59E-203	CD8 Naive
CDK14	3.42E-202	1.908439948	0.749	0.16	8.43E-198	Intermediate B
EPB41L3	5.58E-202	1.071264331	0.763	0.183	1.37E-197	CD16 Mono
UGGT2	6.46E-202	0.653913881	0.248	0.047	1.59E-197	CD14 Mono
PPM1L	2.96E-200	1.329813916	0.768	0.197	7.29E-196	CD16 Mono
JUN	3.33E-200	1.02199529	0.844	0.622	8.18E-196	CD14 Mono
MCTP1	4.69E-196	1.320224489	0.967	0.321	1.15E-191	CD16 Mono
EPHA4	2.40E-195	1.043239921	0.361	0.077	5.90E-191	CD4 TCM
ANGPTL1	1.15E-193	1.385360819	0.424	0.049	2.83E-189	Memory B
PALM2-AKAP2	2.38E-192	1.78959874	0.551	0.091	5.85E-188	Memory B
FMN1	9.05E-185	0.654954178	0.225	0.041	2.23E-180	CD14 Mono

SYNE1	4.08E-184	1.725207533	0.91	0.409	1.00E-179	NK
FMN1	9.79E-184	1.387211444	0.675	0.078	2.41E-179	cDC
SAMD12	7.79E-183	2.225899141	0.896	0.087	1.92E-178	pDC
RPS6	4.64E-181	0.751174459	0.98	0.925	1.14E-176	CD8 Naive
CPPED1	7.92E-180	1.138057396	0.932	0.305	1.95E-175	CD16 Mono
PALM2-AKAP2	1.69E-171	1.316848102	0.556	0.092	4.15E-167	Intermediate B
RAB30	1.45E-170	1.380464101	0.478	0.073	3.57E-166	Memory B
EEF1A1	3.91E-167	0.679182515	0.995	0.989	9.61E-163	CD8 Naive
CD38	1.83E-164	1.374742127	0.456	0.084	4.51E-160	NK
CD302	8.72E-164	0.608817316	0.359	0.123	2.15E-159	CD14 Mono
SAMD3	5.93E-163	1.71266382	0.784	0.21	1.46E-158	CD8 TEM_2
FAM49A	1.08E-162	1.094298369	0.946	0.342	2.67E-158	CD16 Mono
AGAP1	1.87E-162	1.393083581	0.386	0.046	4.61E-158	CD8 TEM_2
MAL	9.23E-161	1.14606552	0.481	0.189	2.27E-156	CD4 Naive
LPCAT2	7.62E-160	0.880030204	0.644	0.155	1.87E-155	CD16 Mono
TIAM1	3.13E-159	0.695237514	0.489	0.215	7.70E-155	CD14 Mono
IRAK3	4.50E-159	0.960512439	0.936	0.297	1.11E-154	CD16 Mono
LEF1-AS1	8.62E-159	0.806836325	0.291	0.067	2.12E-154	CD8 Naive
EEF1A1	1.53E-158	0.621597965	0.999	0.989	3.76E-154	CD4 Naive
CDK14	4.43E-158	1.635612198	0.665	0.162	1.09E-153	Memory B
ARHGAP31	6.55E-156	0.600851167	0.3	0.092	1.61E-151	CD14 Mono
CCDC170	8.00E-156	0.461521104	0.158	0.021	1.97E-151	CD14 Mono
SYNE1	2.56E-155	1.814056222	0.938	0.414	6.29E-151	CD8 TEM_2
MAF	1.02E-154	0.885943967	0.294	0.063	2.52E-150	CD4 TCM
NUCB2	2.99E-153	0.927519192	0.476	0.186	7.36E-149	CD8 Naive
TEC	1.23E-151	0.557470957	0.27	0.076	3.03E-147	CD14 Mono
PIP5K1B	1.63E-148	1.510832716	0.556	0.11	4.02E-144	Intermediate B
ABLIM1	3.35E-146	0.965362665	0.64	0.333	8.23E-142	CD4 Naive
SATB1-AS1	3.66E-144	0.858041915	0.408	0.138	9.01E-140	CD8 Naive
PPM1L	2.89E-140	1.6942891	0.652	0.205	7.12E-136	NK
AC103591.3	2.95E-140	0.661264049	0.264	0.079	7.27E-136	CD14 Mono
RPS6	5.84E-140	0.665397836	0.981	0.925	1.44E-135	CD4 Naive
RETREG1	5.90E-139	0.890660701	0.479	0.196	1.45E-134	CD8 Naive
CCR6	6.82E-139	1.009006094	0.427	0.056	1.68E-134	CD4 TEM
SASH1	8.88E-139	0.687952145	0.194	0.042	2.19E-134	CD14 Mono
IGF1R	6.79E-138	1.164232698	0.568	0.295	1.67E-133	CD4 Naive
TMTC1	1.04E-135	0.509057744	0.143	0.02	2.57E-131	CD14 Mono
WDFY4	1.22E-135	1.618861519	0.665	0.187	3.00E-131	Memory B
HEG1	7.93E-135	0.717124856	0.529	0.12	1.95E-130	CD16 Mono
JAKMIP2	1.53E-134	1.034434672	0.347	0.044	3.77E-130	CD8 TEM_2
MAN1A1	6.70E-134	0.645795606	0.49	0.236	1.65E-129	CD14 Mono
GATA3	3.59E-130	0.846780303	0.371	0.113	8.84E-126	CD4 TCM
PPM1L	1.40E-128	0.506688685	0.398	0.163	3.44E-124	CD14 Mono
ANGPTL1	3.27E-128	1.430841276	0.545	0.056	8.05E-124	Naive B
SH3RF3	2.87E-127	0.860776828	0.299	0.086	7.07E-123	CD8 Naive
FCGR1B	1.46E-126	0.376410203	0.15	0.026	3.59E-122	CD14 Mono
EEF1A1	9.83E-124	0.574109619	0.987	0.99	2.42E-119	CD4 TCM
GNPTAB	6.17E-122	1.482260635	0.693	0.267	1.52E-117	NK
MAF	4.51E-121	1.135769714	0.476	0.078	1.11E-116	CD4 TEM
RALB	1.11E-120	0.589883089	0.463	0.103	2.74E-116	CD16 Mono

TRAT1	1.26E-120	0.74157396	0.381	0.122	3.11E-116	CD4 TCM
LINC01934	5.95E-120	0.881129492	0.511	0.229	1.46E-115	CD8 Naive
AC009226.1	6.19E-118	0.481512082	0.222	0.063	1.52E-113	CD14 Mono
AGAP1	5.54E-117	0.992568244	0.299	0.046	1.36E-112	NK
SAMSN1	1.66E-116	0.686392309	0.541	0.296	4.08E-112	CD14 Mono
RGS18	1.79E-114	0.737809266	0.523	0.137	4.39E-110	CD16 Mono
RPS13	4.54E-114	0.65278575	0.936	0.881	1.12E-109	CD4 Naive
SETBP1	7.45E-113	1.45273565	0.57	0.149	1.83E-108	Memory B
SLC44A1	1.07E-112	0.488545477	0.265	0.09	2.63E-108	CD14 Mono
RTN1	2.13E-111	1.697993639	0.888	0.235	5.25E-107	cDC
WDFY4	5.38E-111	2.155686108	0.981	0.196	1.32E-106	pDC
MIR646HG	5.08E-110	0.635964962	0.325	0.133	1.25E-105	CD14 Mono
BIRC3	1.92E-109	1.500109601	0.662	0.223	4.73E-105	Memory B
PTPRK	4.65E-108	1.380558296	0.435	0.09	1.15E-103	Memory B
TAFA1	1.90E-107	1.095559124	0.453	0.206	4.67E-103	CD4 Naive
CDK14	1.03E-106	2.099700933	0.818	0.171	2.54E-102	Naive B
SYTL2	1.09E-106	1.030182244	0.424	0.099	2.69E-102	NK
JAKMIP2	7.30E-106	0.887574455	0.277	0.044	1.80E-101	NK
RPS13	3.68E-105	0.583460427	0.946	0.879	9.06E-101	CD8 Naive
ICOS	7.35E-105	0.767574569	0.324	0.102	1.81E-100	CD4 TCM
AC120193.1	1.62E-103	1.469454096	0.573	0.076	3.98E-99	Naive B
HOPX	3.53E-102	0.979580261	0.389	0.071	8.69E-98	CD8 TEM_2
HHAT	3.03E-101	1.410713996	0.604	0.066	7.46E-97	pDC
C9orf72	6.46E-101	0.805588173	0.916	0.404	1.59E-96	CD16 Mono
SETBP1	9.56E-100	2.039847778	0.887	0.156	2.35E-95	pDC
EEF1A1	3.90E-99	1.078176371	1	0.99	9.61E-95	CD4 TEM
SATB1-AS1	5.86E-99	0.950788067	0.358	0.147	1.44E-94	CD4 Naive
CDK14	5.06E-98	0.304444465	0.32	0.13	1.25E-93	CD14 Mono
ADAMTS6	5.34E-98	0.940118288	0.342	0.057	1.31E-93	Intermediate B
DPP4	1.03E-97	1.051126441	0.497	0.101	2.53E-93	CD4 TEM
PCSK5	1.53E-97	0.771950567	0.502	0.26	3.76E-93	CD8 Naive
PRKN	4.42E-97	0.813197345	0.448	0.209	1.09E-92	CD8 Naive
IGF1R	7.57E-97	0.76999365	0.548	0.297	1.86E-92	CD8 Naive
L3MBTL4	3.93E-94	1.067409661	0.362	0.083	9.67E-90	NK
KYNU	4.91E-94	0.718060452	0.778	0.292	1.21E-89	CD16 Mono
AGAP1	5.46E-94	1.548479007	0.46	0.052	1.34E-89	MAIT
TAFA1	2.07E-93	0.807063453	0.482	0.209	5.09E-89	CD4 TCM
LY96	3.14E-93	0.413608795	0.195	0.061	7.72E-89	CD14 Mono
MGAT5	3.79E-93	1.597216289	0.808	0.421	9.32E-89	Memory B
PATJ	4.53E-93	0.785488021	0.514	0.237	1.11E-88	CD4 TCM
RASGRP1	4.82E-93	0.696264165	0.598	0.303	1.19E-88	CD4 TCM
ABAT	2.78E-92	0.439981757	0.228	0.08	6.84E-88	CD14 Mono
IL15	5.90E-92	0.664719838	0.702	0.247	1.45E-87	CD16 Mono
ABCA1	9.23E-92	0.443431099	0.182	0.054	2.27E-87	CD14 Mono
PALM2-AKAP2	4.58E-91	1.528886667	0.615	0.1	1.13E-86	Naive B
RGS2	6.56E-91	0.699730757	0.77	0.293	1.61E-86	CD16 Mono
KLF4	1.71E-90	0.546746164	0.732	0.254	4.20E-86	CD16 Mono
RPS6	6.32E-90	0.49863565	0.97	0.928	1.56E-85	CD4 TCM
IGFBP7	1.05E-89	0.610807352	0.447	0.063	2.60E-85	cDC
PATJ	1.40E-89	0.733807169	0.475	0.235	3.44E-85	CD8 Naive

BIRC3	1.59E-89	1.189299934	0.661	0.224	3.90E-85	Intermediate B
TJP2	3.14E-89	0.429137811	0.309	0.063	7.72E-85	CD16 Mono
MCTP2	3.63E-89	1.232078698	0.673	0.235	8.93E-85	CD8 TEM_2
BACH1	1.16E-88	0.759623327	0.92	0.452	2.86E-84	CD16 Mono
MAL	1.63E-87	0.674757377	0.429	0.197	4.02E-83	CD8 Naive
RAB30	2.39E-86	0.844948009	0.382	0.077	5.88E-82	Intermediate B
TMTC1	5.91E-86	0.585218986	0.243	0.043	1.46E-81	CD16 Mono
PIP5K1B	1.12E-85	0.345846127	0.234	0.086	2.75E-81	CD14 Mono
WDFY4	1.13E-84	1.618113277	0.811	0.196	2.77E-80	Naive B
ICOS	1.85E-84	0.754472449	0.28	0.103	4.55E-80	CD4 Naive
CCR6	2.57E-84	0.813594227	0.319	0.057	6.33E-80	Intermediate B
CSGALNACT1	9.14E-84	0.947959744	0.465	0.254	2.25E-79	CD4 Naive
LEF1-AS1	1.64E-82	0.751419394	0.234	0.076	4.04E-78	CD4 Naive
WDFY4	1.78E-82	1.137401772	0.782	0.193	4.38E-78	cDC
BASP1	1.38E-81	1.099380312	0.518	0.097	3.41E-77	cDC
L3MBTL4	2.08E-81	0.359727741	0.19	0.063	5.13E-77	CD14 Mono
PRKCE	3.65E-81	1.456995819	0.781	0.407	8.98E-77	Memory B
CCR6	5.58E-81	0.888811308	0.303	0.057	1.37E-76	Memory B
SH3RF1	9.80E-81	0.426330834	0.19	0.065	2.41E-76	CD14 Mono
EPB41L2	1.57E-80	0.787711984	0.558	0.105	3.88E-76	cDC
LONRF1	6.55E-80	0.404874921	0.251	0.103	1.61E-75	CD14 Mono
MPP7	7.05E-80	0.495139117	0.539	0.318	1.73E-75	CD14 Mono
MIAT	2.62E-79	0.492850094	0.179	0.042	6.45E-75	CD4 TCM
SYTL2	1.35E-78	1.074908588	0.418	0.103	3.32E-74	CD8 TEM_2
TRAT1	3.06E-78	0.805727898	0.304	0.127	7.54E-74	CD4 Naive
SAMSN1	4.89E-78	0.900281761	0.755	0.34	1.20E-73	CD16 Mono
BASP1	1.06E-77	0.912385655	0.399	0.094	2.61E-73	Intermediate B
AC078845.1	1.67E-77	0.4059019	0.251	0.048	4.11E-73	CD16 Mono
ATP6V1B2	7.30E-76	0.583362092	0.597	0.21	1.80E-71	CD16 Mono
AC002460.2	1.91E-75	0.687586942	0.183	0.051	4.70E-71	CD8 Naive
ABCB1	3.14E-75	0.740281229	0.288	0.063	7.72E-71	NK
PCNX2	5.90E-75	0.720452452	0.436	0.209	1.45E-70	CD4 TCM
STAM2	1.25E-74	0.359361859	0.277	0.123	3.08E-70	CD14 Mono
GAB2	2.17E-74	0.447547124	0.84	0.337	5.35E-70	CD16 Mono
PRAG1	2.73E-74	0.625808377	0.273	0.106	6.71E-70	CD8 Naive
ZFHX3	3.74E-74	0.575580921	0.572	0.198	9.21E-70	CD16 Mono
GAB1	5.42E-72	1.455497506	0.726	0.137	1.33E-67	pDC
SETBP1	1.29E-71	1.593437873	0.699	0.156	3.17E-67	Naive B
UGGT2	2.20E-71	0.496534861	0.339	0.088	5.42E-67	CD16 Mono
NR3C2	3.56E-71	0.716107138	0.509	0.294	8.76E-67	CD4 Naive
MIR646HG	1.67E-70	1.155908684	0.473	0.17	4.11E-66	NK
RNF144A	5.89E-70	0.600711569	0.334	0.149	1.45E-65	CD8 Naive
AC120193.1	1.47E-69	0.294315444	0.452	0.076	3.61E-65	cDC
CD96	2.17E-69	0.561008063	0.704	0.415	5.34E-65	CD4 TCM
HS3ST3B1	8.28E-69	1.19643068	0.585	0.087	2.04E-64	pDC
TRIO	9.10E-69	1.205216959	0.689	0.314	2.24E-64	Memory B
MSRA	1.59E-68	0.402646642	0.362	0.19	3.91E-64	CD14 Mono
RALB	2.05E-68	0.315966462	0.215	0.088	5.04E-64	CD14 Mono
RETREG1	5.42E-68	0.786453771	0.393	0.21	1.33E-63	CD4 Naive
ABCB1	7.16E-68	1.147619209	0.453	0.068	1.76E-63	MAIT

HOPX	2.20E-65	1.150280609	0.459	0.077	5.42E-61	gdT
SAMD3	2.36E-65	1.146133065	0.608	0.218	5.82E-61	CD8 TEM_1
TSPAN5	4.00E-65	0.590813555	0.295	0.116	9.85E-61	CD4 TCM
RNF144A	1.01E-64	0.709244795	0.318	0.152	2.49E-60	CD4 Naive
CSGALNACT1	1.22E-64	1.285597194	0.655	0.27	2.99E-60	Intermediate B
EPSTI1	4.51E-64	0.615284795	0.864	0.439	1.11E-59	CD16 Mono
SPRED1	2.04E-63	0.32933948	0.156	0.054	5.02E-59	CD14 Mono
GCNT1	3.78E-63	0.415859838	0.315	0.084	9.29E-59	CD16 Mono
DOCK5	5.09E-63	0.928466467	0.868	0.276	1.25E-58	cDC
VOPP1	1.02E-62	1.222565374	0.766	0.427	2.50E-58	Intermediate B
PVT1	1.54E-62	0.714228513	0.5	0.286	3.79E-58	CD4 TCM
AC068587.4	1.59E-62	0.7119701	0.499	0.282	3.90E-58	CD4 TCM
MGAT5	2.02E-62	1.176225535	0.789	0.422	4.98E-58	Intermediate B
RPS6	8.31E-61	0.793044357	1	0.931	2.05E-56	CD4 TEM
LRMP	5.30E-60	1.209340895	0.644	0.287	1.31E-55	Intermediate B
DAAM1	7.03E-60	1.139708785	0.679	0.13	1.73E-55	pDC
BASP1	1.37E-59	0.312771552	0.187	0.075	3.38E-55	CD14 Mono
MIR646HG	5.90E-59	0.822563364	0.66	0.174	1.45E-54	cDC
AC027097.2	7.42E-59	0.379848208	0.358	0.198	1.83E-54	CD14 Mono
TXN	1.56E-58	1.852234906	0.745	0.188	3.84E-54	pDC
TRIO	2.16E-58	0.946880303	0.888	0.317	5.32E-54	cDC
MNDA	3.79E-58	0.507349734	0.825	0.385	9.32E-54	CD16 Mono
MIAT	2.89E-57	0.800371133	0.25	0.051	7.11E-53	CD8 TEM_2
NBPF15	4.86E-57	0.463856872	0.223	0.079	1.20E-52	CD4 TCM
AGBL3	7.74E-57	0.434722726	0.154	0.047	1.91E-52	CD8 Naive
AP003086.1	9.13E-57	0.468194761	0.64	0.266	2.25E-52	CD16 Mono
NFIA	1.60E-56	0.438361275	0.239	0.113	3.94E-52	CD14 Mono
AC098829.1	4.31E-56	0.364888637	0.173	0.069	1.06E-51	CD14 Mono
CREB3L2	1.17E-55	1.187479673	0.651	0.133	2.87E-51	pDC
SYTL2	1.72E-55	1.380842673	0.526	0.108	4.25E-51	MAIT
FCGR1B	2.25E-55	0.261066217	0.222	0.05	5.54E-51	CD16 Mono
CEP128	3.33E-55	1.258701619	0.632	0.123	8.19E-51	pDC
RGS18	5.95E-55	0.620682803	0.584	0.148	1.46E-50	cDC
RPS6KA2	7.88E-55	0.380352606	0.393	0.128	1.94E-50	CD16 Mono
CCDC141	1.22E-54	0.469350236	0.186	0.066	3.00E-50	CD8 Naive
CEP78	1.44E-54	0.838280296	0.366	0.103	3.53E-50	CD8 TEM_2
DERA	3.69E-54	0.323342547	0.24	0.117	9.08E-50	CD14 Mono
ZBTB20-AS5	4.48E-54	0.796073698	0.336	0.09	1.10E-49	Intermediate B
ACPP	7.61E-54	0.474096364	0.411	0.082	1.87E-49	cDC
MAL	2.49E-53	0.49414514	0.412	0.206	6.14E-49	CD4 TCM
LRMP	6.93E-53	0.48306881	0.646	0.281	1.70E-48	CD16 Mono
ARHGAP31	1.35E-52	0.600574468	0.558	0.138	3.33E-48	cDC
GLUL	6.11E-52	0.432720555	0.451	0.164	1.51E-47	CD16 Mono
PCSK5	7.71E-52	0.276478692	0.424	0.247	1.90E-47	CD14 Mono
DPP4	8.79E-52	1.126998032	0.511	0.106	2.16E-47	MAIT
CHPT1	1.16E-51	0.271733536	0.297	0.157	2.86E-47	CD14 Mono
CEBPD	1.63E-51	0.82226142	0.782	0.259	4.02E-47	cDC
RASGRP1	3.46E-51	0.65351468	0.48	0.313	8.53E-47	CD4 Naive
MOB3B	4.25E-51	0.680544105	0.563	0.147	1.05E-46	cDC
IRF4	5.70E-51	0.474788224	0.294	0.048	1.40E-46	cDC

MAST4	8.26E-51	0.937810799	0.379	0.116	2.03E-46	Intermediate B
ADAMTS6	1.07E-50	0.495946921	0.156	0.052	2.63E-46	CD8 Naive
PIP5K1B	2.07E-50	1.137014609	0.362	0.116	5.10E-46	Memory B
VOPP1	2.97E-50	0.63044131	0.603	0.418	7.31E-46	CD4 TCM
H2AFZ	1.06E-49	0.397954407	0.498	0.189	2.61E-45	CD16 Mono
RTN1	2.50E-49	0.432886152	0.564	0.231	6.16E-45	CD16 Mono
CHPT1	2.83E-49	1.062032504	0.457	0.184	6.97E-45	Memory B
TRIO	3.70E-49	0.924935825	0.672	0.316	9.10E-45	Intermediate B
PRKCE	7.56E-49	1.031976636	0.746	0.409	1.86E-44	Intermediate B
AHR	8.81E-49	0.7355544	0.863	0.308	2.17E-44	cDC
PDGFD	1.83E-48	1.143039717	0.338	0.054	4.51E-44	gdT
KCNQ5	2.18E-48	0.901358918	0.553	0.212	5.38E-44	Intermediate B
SAMD3	2.33E-48	1.363799641	0.689	0.223	5.74E-44	gdT
TRIO	3.23E-48	0.402740028	0.696	0.308	7.94E-44	CD16 Mono
GAB1	3.43E-48	0.412362042	0.375	0.131	8.44E-44	CD16 Mono
CHPT1	1.53E-47	0.794485254	0.484	0.183	3.77E-43	Intermediate B
FAM241A	2.27E-47	0.289105273	0.245	0.125	5.58E-43	CD14 Mono
SH3RF1	5.78E-47	0.602856185	0.411	0.092	1.42E-42	cDC
AC010275.1	1.80E-46	0.498108218	0.18	0.07	4.43E-42	CD4 Naive
ZNF532	1.86E-46	0.698657891	0.245	0.058	4.58E-42	Intermediate B
ACVR2A	1.83E-45	0.293196946	0.225	0.112	4.50E-41	CD14 Mono
DLG2	2.67E-45	0.616905181	0.18	0.07	6.58E-41	CD8 Naive
RPS13	3.33E-45	0.354387348	0.935	0.882	8.20E-41	CD4 TCM
AVEN	6.49E-45	0.830705398	0.425	0.068	1.60E-40	pDC
SYNE1	1.10E-44	1.376509268	0.865	0.426	2.70E-40	gdT
ABCA1	1.10E-44	0.389068109	0.265	0.079	2.71E-40	CD16 Mono
IRF4	1.29E-44	2.096065955	0.778	0.051	3.17E-40	Plasma
FMN1	1.36E-44	0.956673015	0.481	0.085	3.36E-40	pDC
RSAD2	4.54E-44	0.293195868	0.168	0.075	1.12E-39	CD14 Mono
GAS7	7.26E-44	0.635879635	0.863	0.309	1.79E-39	cDC
MNDA	7.79E-44	0.807168462	0.939	0.397	1.92E-39	cDC
PRKN	8.26E-44	0.748727575	0.37	0.222	2.03E-39	CD4 Naive
RASGRP1	1.10E-43	0.467553856	0.491	0.311	2.71E-39	CD8 Naive
ARHGAP10	1.85E-43	0.964686934	0.386	0.14	4.54E-39	CD8 TEM_2
PTPRK	1.99E-43	0.738178148	0.325	0.094	4.90E-39	Intermediate B
CCR6	2.88E-43	0.808562253	0.35	0.062	7.08E-39	Naive B
ZNF827	4.62E-43	0.471517728	0.231	0.105	1.14E-38	CD8 Naive
ANKH	1.48E-42	0.489667274	0.378	0.205	3.64E-38	CD4 TCM
EXT1	1.58E-42	0.472017641	0.739	0.376	3.89E-38	CD16 Mono
CSGALNACT1	5.88E-42	0.542616629	0.441	0.263	1.45E-37	CD4 TCM
LMO4	1.43E-41	0.255765909	0.323	0.19	3.51E-37	CD14 Mono
TEC	4.48E-41	0.652874314	0.457	0.121	1.10E-36	cDC
PRKCE	9.63E-41	0.366227474	0.794	0.401	2.37E-36	CD16 Mono
ACVR1	1.46E-40	0.48534508	0.251	0.117	3.60E-36	CD4 TCM
TAPT1-AS1	2.07E-40	0.868550939	0.311	0.103	5.11E-36	Memory B
MCTP2	2.55E-40	0.791070857	0.532	0.239	6.29E-36	Memory B
ABCB1	2.73E-40	0.381429959	0.156	0.059	6.72E-36	CD8 Naive
TNS3	3.75E-40	0.579165301	0.736	0.25	9.22E-36	cDC
FRMD4B	5.82E-40	0.774798723	0.584	0.186	1.43E-35	cDC
CCNG2	7.54E-40	0.265563241	0.208	0.057	1.86E-35	CD16 Mono

DPP4	1.80E-39	0.454563509	0.224	0.097	4.43E-35	CD4 TCM
ESR1	2.99E-39	1.383737783	0.889	0.071	7.36E-35	Plasma
MCTP1	4.68E-39	0.557082811	0.914	0.342	1.15E-34	cDC
CD38	4.79E-39	2.122707938	1	0.099	1.18E-34	Plasma
ZSWIM6	6.75E-39	0.369451611	0.877	0.492	1.66E-34	CD16 Mono
AC010275.1	8.56E-39	0.363868273	0.174	0.071	2.11E-34	CD8 Naive
MYOF	9.24E-39	0.537071521	0.716	0.24	2.27E-34	cDC
BIRC3	1.77E-38	0.482877361	0.384	0.22	4.35E-34	CD4 TCM
KLF4	1.93E-38	0.572719661	0.751	0.269	4.76E-34	cDC
ARHGAP31	4.48E-38	1.062858321	0.575	0.142	1.10E-33	pDC
LINC02273	1.20E-37	0.282429732	0.137	0.045	2.95E-33	CD4 TCM
GNPTAB	1.23E-37	0.903860108	0.543	0.277	3.04E-33	CD8 TEM_2
L3MBTL4	2.17E-37	0.928861909	0.281	0.089	5.34E-33	Memory B
ACPP	5.04E-37	0.295463597	0.249	0.079	1.24E-32	CD16 Mono
FAM169A	5.53E-37	0.393378793	0.155	0.061	1.36E-32	CD8 Naive
WWOX	5.79E-37	1.127360496	0.724	0.49	1.42E-32	Memory B
ATP8B1	7.36E-37	0.847308462	0.493	0.216	1.81E-32	Intermediate B
CD96	8.00E-37	0.474789634	0.593	0.425	1.97E-32	CD4 Naive
TAFA1	9.62E-37	0.442471581	0.374	0.218	2.37E-32	CD8 Naive
LMO4	1.46E-36	0.306628345	0.492	0.211	3.60E-32	CD16 Mono
LY96	1.55E-36	0.285241971	0.267	0.088	3.80E-32	CD16 Mono
ENOSF1	2.21E-36	0.479257245	0.312	0.18	5.43E-32	CD8 Naive
CD302	2.27E-36	0.349724562	0.418	0.173	5.59E-32	CD16 Mono
RPS6KA2	2.79E-36	0.894197791	0.566	0.137	6.87E-32	pDC
YES1	5.15E-36	0.604796572	0.247	0.068	1.27E-31	CD8 TEM_2
RAP2A	8.84E-36	0.716077344	0.307	0.106	2.18E-31	CD8 TEM_2
PTPRE	1.07E-35	0.629604003	0.914	0.394	2.62E-31	cDC
ATP8B1	2.79E-35	0.950798248	0.459	0.216	6.86E-31	Memory B
LINC01572	3.53E-35	0.849659818	0.254	0.075	8.69E-31	Intermediate B
IMMP2L	4.53E-35	0.499429573	0.612	0.47	1.11E-30	CD8 Naive
XKR6	6.28E-35	0.668645518	0.51	0.22	1.55E-30	Intermediate B
GCNT1	7.15E-35	0.848564749	0.268	0.089	1.76E-30	Memory B
RPS13	7.35E-35	0.587669225	0.983	0.886	1.81E-30	CD4 TEM
ANKH	1.71E-34	0.571356203	0.33	0.208	4.21E-30	CD4 Naive
RIPK2	1.85E-34	0.261761575	0.588	0.276	4.56E-30	CD16 Mono
CYTOR	2.14E-34	0.846343863	0.435	0.198	5.28E-30	CD8 TEM_2
BASP1	2.27E-34	0.924554591	0.406	0.1	5.58E-30	Naive B
STK3	2.81E-34	0.320276451	0.535	0.247	6.91E-30	CD16 Mono
TIAM1	8.79E-34	0.592481096	0.421	0.27	2.16E-29	CD4 TCM
PTPRE	1.15E-33	1.141073892	0.925	0.398	2.84E-29	pDC
LINC01934	1.64E-33	0.99373578	0.54	0.259	4.04E-29	CD8 TEM_1
PDP1	2.02E-33	0.255932217	0.241	0.08	4.97E-29	CD16 Mono
NIPAL2	2.12E-33	0.387551975	0.315	0.072	5.21E-29	cDC
MICAL2	2.55E-33	0.514864891	0.584	0.194	6.28E-29	cDC
CPQ	2.73E-33	0.364190271	0.77	0.419	6.71E-29	CD16 Mono
CPPED1	3.06E-33	0.448077552	0.838	0.326	7.54E-29	cDC
EPHA4	3.14E-33	0.633904579	0.33	0.103	7.73E-29	CD4 TEM
TRIO	3.18E-33	1.253527577	0.734	0.322	7.83E-29	Naive B
MAN1A1	3.51E-33	0.785349206	0.52	0.293	8.64E-29	NK
AGAP1	4.00E-33	0.518562136	0.222	0.053	9.86E-29	CD4 TEM

GATA3	7.92E-33	0.714825569	0.385	0.135	1.95E-28	CD4 TEM
HEG1	8.10E-33	0.677152677	0.318	0.132	1.99E-28	NK
TRAT1	1.22E-32	0.520925998	0.417	0.144	3.01E-28	CD4 TEM
CD96	2.04E-32	0.775557704	0.744	0.438	5.01E-28	CD8 TEM_1
NR3C2	4.70E-32	0.462601361	0.473	0.304	1.16E-27	CD4 TCM
JAKMIP2	4.83E-32	0.878689897	0.27	0.052	1.19E-27	gdT
CD302	5.67E-32	0.479166118	0.543	0.178	1.40E-27	cDC
KANK1	2.07E-31	0.510683136	0.139	0.058	5.09E-27	CD4 Naive
PHLPP2	2.60E-31	0.688761171	0.33	0.058	6.40E-27	pDC
ZFHX3	4.03E-31	0.462463493	0.599	0.209	9.92E-27	cDC
PATJ	4.18E-31	0.540458613	0.376	0.252	1.03E-26	CD4 Naive
MAST4	5.52E-31	0.363894873	0.23	0.111	1.36E-26	CD4 TCM
CALHM6	1.48E-30	0.454734096	0.503	0.161	3.65E-26	cDC
CDK14	1.52E-30	0.299788216	0.538	0.173	3.74E-26	cDC
BMPR1A	1.88E-30	0.36656409	0.129	0.048	4.62E-26	CD4 TCM
MICU3	2.02E-30	0.339201108	0.149	0.064	4.96E-26	CD8 Naive
MYC	2.19E-30	0.503935264	0.224	0.123	5.38E-26	CD4 Naive
LCLAT1	3.06E-30	0.477114868	0.268	0.155	7.54E-26	CD8 Naive
RAB30	4.07E-30	1.041651599	0.889	0.086	1.00E-25	Plasma
ZHX2	4.38E-30	0.687310873	0.641	0.351	1.08E-25	Intermediate B
EPB41L2	4.44E-30	0.872811913	0.286	0.107	1.09E-25	Memory B
PID1	4.93E-30	0.712065232	0.614	0.236	1.21E-25	cDC
JAKMIP2	1.71E-29	0.712939747	0.577	0.053	4.22E-25	HSPC
BTBD11	1.74E-29	0.505558389	0.299	0.182	4.28E-25	CD8 Naive
SYNE1	3.19E-29	0.919913045	0.651	0.425	7.86E-25	CD8 TEM_1
USP53	3.45E-29	0.398651018	0.231	0.124	8.49E-25	CD8 Naive
AC027097.2	5.10E-29	0.720501211	0.49	0.232	1.25E-24	Intermediate B
SAMD3	5.14E-29	0.876618088	0.635	0.225	1.26E-24	MAIT
TSPAN5	8.70E-29	0.658798135	0.358	0.13	2.14E-24	CD4 TEM
AC027097.2	8.75E-29	0.863701936	0.454	0.233	2.15E-24	Memory B
BMP2K	1.47E-28	0.704270408	0.524	0.273	3.62E-24	Intermediate B
MZT2B	1.60E-28	0.669228359	0.653	0.354	3.93E-24	CD4 TEM
CEBPD	1.82E-28	0.255543307	0.531	0.255	4.47E-24	CD16 Mono
EPB41L2	2.10E-28	0.340197566	0.27	0.106	5.18E-24	CD16 Mono
RHBDD1	3.31E-28	0.81273248	0.473	0.253	8.14E-24	Memory B
PALM2-AKAP2	5.00E-28	1.192594601	0.944	0.106	1.23E-23	Plasma
MIAT	1.18E-27	0.58808718	0.194	0.053	2.90E-23	CD8 TEM_1
SSR3	1.27E-27	1.600069834	1	0.136	3.11E-23	Plasma
BMP2K	3.47E-27	0.757693673	0.503	0.274	8.53E-23	Memory B
XKR6	3.60E-27	0.885388957	0.594	0.224	8.86E-23	Naive B
MAN1A1	6.55E-27	1.153247246	0.774	0.298	1.61E-22	pDC
RSAD2	8.24E-27	0.255042399	0.245	0.092	2.03E-22	CD16 Mono
STK3	8.96E-27	0.316337192	0.675	0.253	2.20E-22	cDC
LINC01934	8.97E-27	0.544319939	0.373	0.252	2.21E-22	CD4 Naive
MAF	1.17E-26	0.674707216	0.35	0.086	2.88E-22	MAIT
SSBP2	1.44E-26	0.796167889	0.638	0.424	3.54E-22	Memory B
IGFBP7	1.76E-26	1.175656828	0.615	0.069	4.34E-22	HSPC
AC103591.3	2.29E-26	1.060408146	0.462	0.125	5.63E-22	pDC
NUCB2	2.52E-26	0.528262752	0.317	0.212	6.19E-22	CD4 Naive
MCTP2	2.81E-26	0.970618776	0.601	0.245	6.91E-22	gdT

MAST4	7.29E-26	0.496636925	0.204	0.112	1.79E-21	CD4 Naive
DISC1	8.05E-26	0.56479395	0.893	0.454	1.98E-21	cDC
CENPK	9.18E-26	0.478029666	0.186	0.099	2.26E-21	CD4 Naive
SSBP2	1.02E-25	0.581309633	0.501	0.42	2.51E-21	CD4 Naive
USP53	1.40E-25	0.347600978	0.238	0.127	3.43E-21	CD4 TCM
KYNU	1.95E-25	0.381682819	0.746	0.307	4.80E-21	cDC
BIRC3	3.76E-25	0.854050494	0.601	0.234	9.24E-21	Naive B
GTDC1	4.56E-25	0.72428166	0.42	0.21	1.12E-20	CD8 TEM_2
PRAG1	7.89E-25	0.585917337	0.311	0.122	1.94E-20	Intermediate B
MGAT5	8.24E-25	0.377469845	0.551	0.42	2.03E-20	CD4 TCM
SH3RF3	8.78E-25	0.495514584	0.192	0.104	2.16E-20	CD4 Naive
LMO4	9.95E-25	0.394680534	0.579	0.218	2.45E-20	cDC
LINC01934	1.10E-24	0.691436486	0.506	0.26	2.70E-20	CD8 TEM_2
SSR3	1.50E-24	0.688705293	0.481	0.134	3.70E-20	pDC
GALNT10	1.62E-24	0.991237879	0.642	0.275	3.99E-20	MAIT
MGAT5	2.35E-24	0.59204514	0.705	0.427	5.78E-20	CD4 TEM
TEC	3.05E-24	1.081232243	0.808	0.125	7.51E-20	HSPC
CCDC141	3.26E-24	0.384312625	0.15	0.072	8.02E-20	CD4 Naive
CCDC141	4.37E-24	0.989844368	0.293	0.08	1.07E-19	Treg
TOR3A	4.98E-24	0.270750974	0.294	0.08	1.23E-19	cDC
C11orf65	8.91E-24	0.36549679	0.231	0.136	2.19E-19	CD8 Naive
OSBPL3	1.11E-23	0.57835987	0.409	0.222	2.73E-19	NK
SLC12A2	1.15E-23	0.70947114	0.396	0.096	2.84E-19	pDC
LARP1B	1.33E-23	1.206286741	0.889	0.115	3.28E-19	Plasma
OSBPL3	2.03E-23	0.402704549	0.336	0.217	5.00E-19	CD4 TCM
GTDC1	2.11E-23	0.861392058	0.414	0.211	5.20E-19	CD8 TEM_1
LONRF1	2.60E-23	0.25142011	0.305	0.134	6.41E-19	CD16 Mono
KYNU	3.60E-23	0.827184824	0.692	0.31	8.85E-19	Naive B
CEP128	3.65E-23	0.388406322	0.217	0.117	8.98E-19	CD4 TCM
NIPAL2	5.20E-23	0.438334877	0.214	0.072	1.28E-18	Intermediate B
TXN	5.77E-23	0.424050895	0.513	0.188	1.42E-18	cDC
MAL	6.07E-23	0.446359491	0.497	0.221	1.50E-18	CD4 TEM
MARCH3	6.21E-23	0.556030584	0.271	0.104	1.53E-18	Intermediate B
GAB1	6.61E-23	1.562930818	0.944	0.142	1.63E-18	Plasma
MGAT5	1.24E-22	1.022038839	0.762	0.43	3.05E-18	Naive B
GALNT10	1.34E-22	0.774519669	0.48	0.273	3.30E-18	CD8 TEM_2
CCDC138	1.43E-22	0.632628202	0.311	0.067	3.51E-18	pDC
SETBP1	1.46E-22	0.542934349	0.358	0.157	3.60E-18	CD8 TEM_2
AC093010.2	1.53E-22	0.256609354	0.122	0.05	3.77E-18	CD4 TCM
PIP5K1B	1.75E-22	1.043505508	0.378	0.121	4.30E-18	Naive B
CEP128	2.45E-22	1.563455264	0.889	0.127	6.03E-18	Plasma
RASGRP1	2.67E-22	0.530714547	0.628	0.328	6.57E-18	CD4 TEM
KANK1	5.41E-22	0.355594853	0.135	0.06	1.33E-17	CD4 TCM
JAKMIP2	6.37E-22	0.52645783	0.173	0.051	1.57E-17	CD8 TEM_1
BASP1	6.89E-22	0.569241858	0.249	0.099	1.70E-17	Memory B
SAMD12	7.30E-22	0.461223397	0.264	0.091	1.80E-17	CD4 TEM
HOPX	7.94E-22	0.703199217	0.307	0.079	1.95E-17	MAIT
GABPB1-AS1	8.76E-22	0.401526094	0.299	0.2	2.16E-17	CD8 Naive
MPP6	1.60E-21	0.279934447	0.156	0.08	3.94E-17	CD8 Naive
STX18	3.02E-21	0.294405749	0.482	0.176	7.43E-17	cDC

MARCH3	3.51E-21	0.926933338	0.35	0.106	8.64E-17	Naive B
PDGFD	4.18E-21	0.646946401	0.179	0.055	1.03E-16	CD8 TEM_1
GCNT1	4.94E-21	0.278228033	0.305	0.091	1.21E-16	cDC
BIRC3	5.65E-21	0.917644752	0.524	0.234	1.39E-16	Treg
SYTL2	5.71E-21	0.953371118	0.345	0.11	1.41E-16	gdT
MZT2B	6.70E-21	0.338328664	0.467	0.349	1.65E-16	CD4 TCM
MIAT	7.06E-21	0.603516167	0.226	0.055	1.74E-16	Treg
SETBP1	8.87E-21	0.491372261	0.322	0.156	2.18E-16	NK
TSPAN5	9.20E-21	0.94378966	0.372	0.132	2.26E-16	Treg
TMEM220	1.22E-20	0.2523636	0.101	0.044	3.01E-16	CD8 Naive
ANXA2R	2.33E-20	0.281395202	0.193	0.11	5.74E-16	CD8 Naive
BASP1	2.39E-20	0.645371549	0.833	0.103	5.88E-16	Plasma
RAB30	2.69E-20	0.584423396	0.308	0.085	6.63E-16	Naive B
IMMP2L	3.34E-20	0.492979812	0.553	0.48	8.21E-16	CD4 Naive
CENPK	3.99E-20	0.284285714	0.179	0.099	9.82E-16	CD8 Naive
CCDC152	4.09E-20	0.381827005	0.274	0.056	1.01E-15	pDC
BMP2K	5.52E-20	0.933530287	0.601	0.277	1.36E-15	Naive B
MYC	6.64E-20	0.295836405	0.211	0.124	1.63E-15	CD8 Naive
GALNT10	7.61E-20	0.620243636	0.439	0.272	1.87E-15	NK
ENOSF1	7.63E-20	0.491150315	0.271	0.187	1.88E-15	CD4 Naive
ACVR2A	8.13E-20	0.410246253	0.223	0.132	2.00E-15	CD4 TCM
GAB1	8.46E-20	0.913936303	0.289	0.138	2.08E-15	Memory B
CEP128	8.66E-20	1.082398189	0.354	0.124	2.13E-15	Treg
PCSK5	9.56E-20	0.527012153	0.366	0.282	2.35E-15	CD4 Naive
ZHX2	1.00E-19	0.798023017	0.538	0.354	2.47E-15	Memory B
SAMSN1	1.03E-19	0.32415955	0.469	0.346	2.54E-15	CD4 TCM
LPCAT2	1.06E-19	0.322268777	0.457	0.173	2.61E-15	cDC
CCR6	2.31E-19	0.580558108	0.255	0.064	5.68E-15	MAIT
ZNF532	3.71E-19	0.496984845	0.173	0.06	9.14E-15	Memory B
KCNQ5	4.55E-19	0.496366366	0.308	0.211	1.12E-14	CD4 Naive
CMSS1	5.10E-19	0.42875589	0.325	0.234	1.26E-14	CD8 Naive
AZIN1-AS1	5.35E-19	0.698934352	0.245	0.052	1.32E-14	pDC
MIAT	5.39E-19	0.697362137	0.223	0.055	1.33E-14	gdT
OSBPL3	9.15E-19	0.633574158	0.401	0.224	2.25E-14	CD8 TEM_2
PALM2-AKAP2	9.81E-19	0.752230413	0.317	0.104	2.41E-14	Treg
CENPK	1.26E-18	0.261466667	0.187	0.101	3.10E-14	CD4 TCM
CEP128	1.40E-18	0.309407071	0.355	0.124	3.45E-14	cDC
MZT2A	1.48E-18	0.481744488	0.413	0.196	3.63E-14	CD4 TEM
ANKH	1.63E-18	0.333862986	0.305	0.211	4.02E-14	CD8 Naive
NUCB2	1.75E-18	0.622716175	0.623	0.223	4.30E-14	pDC
EEF1A1	2.07E-18	0.564448831	1	0.99	5.09E-14	MAIT
NBPF15	2.30E-18	0.406795188	0.154	0.086	5.67E-14	CD4 Naive
DIP2C	2.62E-18	0.285514113	0.134	0.065	6.45E-14	CD4 TCM
HHAT	3.38E-18	0.273354273	0.133	0.064	8.31E-14	CD4 TCM
LINC01184	3.70E-18	0.321305567	0.241	0.156	9.11E-14	CD8 Naive
CEBPD	3.87E-18	0.428619621	0.437	0.261	9.53E-14	NK
PPM1L	5.63E-18	0.264125	0.528	0.219	1.39E-13	cDC
C1orf112	1.03E-17	0.32501007	0.17	0.098	2.53E-13	CD8 Naive
MTHFD2L	1.04E-17	0.570991484	0.302	0.077	2.55E-13	pDC
CD38	1.12E-17	0.523207906	0.358	0.098	2.75E-13	pDC

SSR3	1.14E-17	0.255155612	0.371	0.133	2.80E-13	cDC
CD96	1.15E-17	0.902020306	0.774	0.443	2.83E-13	MAIT
GATA3	1.19E-17	0.858681595	0.366	0.138	2.94E-13	Treg
OSBPL3	1.68E-17	0.5421407	0.438	0.225	4.12E-13	CD4 TEM
SLC44A1	1.85E-17	1.49753044	0.833	0.135	4.56E-13	Plasma
MYC	2.35E-17	0.271102713	0.216	0.126	5.78E-13	CD4 TCM
LRMP	2.86E-17	0.693845432	0.608	0.295	7.05E-13	Naive B
MAST4	2.94E-17	0.44392389	0.292	0.12	7.25E-13	CD4 TEM
RP9	3.35E-17	0.454119189	0.186	0.072	8.25E-13	Memory B
HS3ST3B1	3.38E-17	0.708792999	0.274	0.089	8.31E-13	Treg
SETBP1	3.73E-17	1.214658663	0.944	0.163	9.19E-13	Plasma
ABCA1	4.32E-17	0.483967429	0.205	0.084	1.06E-12	Memory B
GABPB1-AS1	5.47E-17	0.620116911	0.37	0.208	1.35E-12	Memory B
MTHFD2L	8.52E-17	0.618280779	0.538	0.078	2.10E-12	HSPC
RASGRP1	1.36E-16	0.504862982	0.534	0.329	3.34E-12	CD8 TEM_2
CPLANE1	1.46E-16	0.594935216	0.387	0.118	3.58E-12	pDC
BTBD11	2.05E-16	0.440680445	0.266	0.188	5.04E-12	CD4 Naive
HS3ST3B1	2.17E-16	0.295116012	0.158	0.084	5.34E-12	CD4 TCM
AL365295.1	2.35E-16	0.643099652	0.283	0.073	5.77E-12	pDC
MOB3B	2.50E-16	0.631448918	0.292	0.149	6.15E-12	Memory B
SYTL2	2.68E-16	0.42902033	0.271	0.109	6.59E-12	CD4 TEM
SYTL2	3.91E-16	0.619324451	0.25	0.109	9.62E-12	CD8 TEM_1
MAST4	5.42E-16	0.759767721	0.692	0.123	1.33E-11	HSPC
MAN1A1	5.46E-16	2.190134336	1	0.302	1.34E-11	Plasma
KYNU	5.98E-16	0.535431279	0.736	0.311	1.47E-11	pDC
NTPCR	1.08E-15	0.444651464	0.462	0.061	2.66E-11	HSPC
EPS8	1.14E-15	0.314376711	0.279	0.095	2.80E-11	cDC
AZIN1-AS1	1.21E-15	0.392174318	0.423	0.053	2.98E-11	HSPC
TSPAN5	1.28E-15	0.565120395	0.372	0.133	3.16E-11	MAIT
DPP4	1.30E-15	0.332260354	0.17	0.102	3.21E-11	CD4 Naive
CD96	1.41E-15	0.412547147	0.646	0.438	3.46E-11	NK
BTBD11	1.46E-15	0.853984343	0.453	0.195	3.60E-11	MAIT
PRKCE	1.55E-15	0.308815866	0.797	0.414	3.81E-11	cDC
MCTP2	1.78E-15	0.370151761	0.442	0.243	4.39E-11	Intermediate B
KCNQ5	2.03E-15	0.580463479	0.392	0.218	4.99E-11	Memory B
GAB2	2.13E-15	0.594093277	0.792	0.357	5.25E-11	pDC
ATP6V1B2	2.74E-15	0.292888443	0.523	0.224	6.75E-11	cDC
CHPT1	2.88E-15	0.618983838	0.441	0.19	7.10E-11	Naive B
ANKAR	5.23E-15	0.552063319	0.265	0.13	1.29E-10	Memory B
ABCB1	5.49E-15	0.343417585	0.194	0.069	1.35E-10	CD4 TEM
ACVR1	7.07E-15	0.752241205	0.323	0.129	1.74E-10	Treg
LARP1B	7.09E-15	0.289687681	0.305	0.112	1.74E-10	cDC
STX18	7.17E-15	0.630474597	0.481	0.178	1.76E-10	pDC
GPHN	7.86E-15	0.394900976	0.368	0.273	1.94E-10	CD4 TCM
AC093010.2	8.52E-15	0.416085285	0.153	0.055	2.10E-10	CD8 TEM_2
SAMD12	9.02E-15	0.804033822	0.667	0.095	2.22E-10	Plasma
DAB1	9.47E-15	0.380047221	0.17	0.059	2.33E-10	CD4 TEM
UBE3D	1.35E-14	0.323534798	0.166	0.101	3.33E-10	CD8 Naive
AC068587.4	1.52E-14	0.581703058	0.503	0.301	3.73E-10	CD4 TEM
MPP7	1.83E-14	0.29327532	0.448	0.364	4.51E-10	CD8 Naive

CYTOR	2.10E-14	0.487349012	0.33	0.2	5.17E-10	NK
GPHN	2.48E-14	0.503877343	0.476	0.279	6.10E-10	CD4 TEM
ZNF532	2.78E-14	0.49057441	0.217	0.062	6.85E-10	Naive B
ESR1	3.11E-14	0.260618659	0.125	0.065	7.65E-10	CD4 TCM
CREB3L2	4.00E-14	0.353592588	0.254	0.133	9.84E-10	NK
MZT2A	6.21E-14	0.260510194	0.283	0.192	1.53E-09	CD4 TCM
ERCC8	6.99E-14	0.293797669	0.289	0.108	1.72E-09	cDC
DLG2	7.56E-14	0.659774424	0.189	0.081	1.86E-09	Memory B
NR3C2	8.70E-14	0.321788319	0.559	0.316	2.14E-09	CD4 TEM
SAMD12	9.02E-14	0.269339277	0.149	0.087	2.22E-09	CD8 Naive
MPP6	9.18E-14	0.586746498	0.202	0.087	2.26E-09	Intermediate B
HDDC2	1.11E-13	0.315552162	0.141	0.058	2.73E-09	NK
LMO4	1.19E-13	0.461456601	0.345	0.219	2.92E-09	NK
AC093010.2	1.22E-13	0.310956478	0.136	0.055	3.00E-09	NK
DAAM1	1.29E-13	0.778665967	0.336	0.133	3.18E-09	Naive B
MOB3B	1.46E-13	0.462670117	0.291	0.15	3.59E-09	Intermediate B
ZHX2	1.69E-13	0.68526519	0.643	0.357	4.16E-09	Naive B
AC068587.4	1.74E-13	1.001171444	0.547	0.303	4.27E-09	MAIT
XKR6	1.79E-13	0.587192482	0.37	0.224	4.41E-09	Memory B
USP53	1.93E-13	0.361295637	0.195	0.13	4.74E-09	CD4 Naive
LARP1B	2.01E-13	0.575477025	0.615	0.115	4.94E-09	HSPC
BMPR1A	2.08E-13	0.352770716	0.145	0.054	5.11E-09	CD8 TEM_2
LONRF1	2.30E-13	0.433320407	0.274	0.138	5.65E-09	Intermediate B
AGAP1	2.68E-13	0.598878781	0.196	0.056	6.59E-09	gdT
AC120193.1	2.84E-13	0.486627304	0.5	0.082	6.99E-09	HSPC
ZHX2	3.38E-13	0.312076058	0.446	0.35	8.32E-09	CD4 TCM
ENOSF1	3.45E-13	0.285764762	0.275	0.188	8.49E-09	CD4 TCM
PCNX2	3.63E-13	0.419713523	0.42	0.229	8.94E-09	CD4 TEM
PCNX2	4.09E-13	0.581482911	0.383	0.229	1.01E-08	CD8 TEM_1
ICOS	5.56E-13	0.616791339	0.311	0.124	1.37E-08	Treg
SAMD12	6.25E-13	0.773214845	0.266	0.093	1.54E-08	Naive B
HOPX	6.29E-13	0.288294535	0.201	0.079	1.55E-08	CD4 TEM
MYC	7.34E-13	0.372575025	0.285	0.132	1.81E-08	CD4 TEM
WDR60	7.43E-13	0.492142045	0.396	0.141	1.83E-08	pDC
TAPT1-AS1	7.47E-13	0.667585829	0.577	0.109	1.84E-08	HSPC
ATP2C1	1.02E-12	1.266268322	0.846	0.245	2.50E-08	HSPC
MYO9A	1.14E-12	0.766138501	0.613	0.302	2.81E-08	pDC
OSBPL3	1.24E-12	0.723272892	0.446	0.227	3.05E-08	gdT
LINC01934	1.31E-12	0.453485771	0.348	0.258	3.24E-08	CD4 TCM
EEF1A1	1.39E-12	0.254347432	1	0.99	3.41E-08	CD8 TEM_1
ATP8B1	1.42E-12	0.725805143	0.509	0.222	3.50E-08	pDC
XKR6	1.54E-12	0.314896422	0.308	0.219	3.80E-08	CD4 TCM
PIP5K1B	1.59E-12	1.275691238	0.667	0.124	3.90E-08	Plasma
EEF1A1	1.65E-12	0.470519379	0.982	0.99	4.06E-08	Treg
TNS3	1.88E-12	0.310033994	0.43	0.254	4.64E-08	Intermediate B
TSPAN5	2.26E-12	0.383379085	0.245	0.131	5.56E-08	NK
AP003086.1	2.28E-12	0.700876522	0.594	0.282	5.62E-08	pDC
RHBDD1	2.35E-12	0.439112996	0.422	0.255	5.79E-08	Intermediate B
SGMS2	2.80E-12	0.257539629	0.244	0.089	6.89E-08	cDC
VOPP1	3.21E-12	0.821778215	0.692	0.435	7.90E-08	Naive B

EPHA4	3.42E-12	0.523382774	0.225	0.105	8.41E-08	CD8 TEM_1
AC093010.2	3.83E-12	0.432050595	0.145	0.056	9.43E-08	CD8 TEM_1
ANKH	3.95E-12	0.759432234	0.427	0.221	9.73E-08	Treg
WWOX	4.12E-12	0.781520403	0.802	0.495	1.01E-07	pDC
BIRC3	4.31E-12	0.359329667	0.413	0.234	1.06E-07	CD4 TEM
C1orf112	5.52E-12	0.337430458	0.155	0.101	1.36E-07	CD4 Naive
RASGRP1	5.85E-12	0.472540764	0.5	0.331	1.44E-07	CD8 TEM_1
PCNX4	6.11E-12	0.596081509	0.509	0.223	1.50E-07	pDC
RPS6	6.25E-12	0.282595525	0.972	0.932	1.54E-07	CD8 TEM_1
MAN1A1	6.28E-12	0.663848497	0.555	0.3	1.55E-07	MAIT
SSBP2	7.64E-12	1.412279781	0.962	0.43	1.88E-07	HSPC
TRMT11	1.02E-11	0.28367283	0.186	0.125	2.51E-07	CD8 Naive
KIAA0825	1.10E-11	0.470226589	0.172	0.11	2.70E-07	CD4 TCM
HS2ST1	1.17E-11	0.636893158	0.297	0.175	2.89E-07	Memory B
CD96	1.20E-11	0.258950279	0.701	0.44	2.96E-07	CD4 TEM
HHAT	1.44E-11	0.405751997	0.217	0.07	3.54E-07	Naive B
ACYP2	1.59E-11	0.362957322	0.442	0.274	3.93E-07	Intermediate B
FILIP1L	1.84E-11	0.323373853	0.218	0.155	4.54E-07	CD8 Naive
MAST4	2.02E-11	0.629740082	0.293	0.122	4.98E-07	Treg
SLC35F1	2.24E-11	0.349679696	0.253	0.122	5.51E-07	CD4 TEM
MAN1A1	2.28E-11	0.479542685	0.443	0.298	5.62E-07	CD8 TEM_2
CD96	2.79E-11	0.270314191	0.665	0.44	6.87E-07	CD8 TEM_2
CCDC138	3.02E-11	0.53155411	0.151	0.066	7.42E-07	Memory B
ZNF827	3.03E-11	0.809809904	0.577	0.122	7.47E-07	HSPC
EPHA4	3.32E-11	0.673949037	0.28	0.107	8.17E-07	Naive B
LRMP	3.33E-11	0.639033439	0.416	0.295	8.19E-07	Memory B
USP45	4.90E-11	0.4505532	0.264	0.083	1.21E-06	pDC
ITGB3BP	5.68E-11	0.315035032	0.254	0.129	1.40E-06	Intermediate B
TAFA1	5.99E-11	0.370445651	0.413	0.235	1.47E-06	CD4 TEM
GATA3	6.37E-11	0.482373067	0.343	0.139	1.57E-06	MAIT
AC092944.1	6.61E-11	0.296686463	0.136	0.085	1.63E-06	CD8 Naive
PATJ	7.51E-11	0.272930516	0.469	0.263	1.85E-06	CD4 TEM
TBC1D19	8.07E-11	0.372045105	0.19	0.098	1.99E-06	NK
SATB1-AS1	8.84E-11	0.585597786	0.731	0.174	2.18E-06	HSPC
TXN	9.95E-11	0.752124586	0.731	0.192	2.45E-06	HSPC
ACVR1	1.08E-10	0.399487251	0.257	0.129	2.66E-06	CD4 TEM
ESR1	1.11E-10	0.397107229	0.11	0.066	2.74E-06	CD4 Naive
LINC01184	1.19E-10	0.371878683	0.425	0.165	2.93E-06	pDC
CCDC141	1.22E-10	0.274618941	0.179	0.079	3.01E-06	Intermediate B
ABCA1	1.40E-10	0.318421475	0.182	0.085	3.45E-06	Intermediate B
RPS6	1.46E-10	0.469975099	0.963	0.932	3.58E-06	Treg
AC027097.2	1.66E-10	0.574352184	0.519	0.238	4.09E-06	pDC
CEMIP2	2.26E-10	0.49554113	0.522	0.39	5.57E-06	Memory B
Z94721.1	2.38E-10	0.288744979	0.157	0.099	5.85E-06	CD4 TCM
TBC1D19	2.55E-10	0.440627142	0.199	0.099	6.26E-06	CD8 TEM_2
IRF4	2.78E-10	0.360833967	0.122	0.05	6.85E-06	Memory B
FILIP1L	2.82E-10	0.31904795	0.291	0.159	6.93E-06	Intermediate B
CYTOR	3.15E-10	0.725523332	0.385	0.203	7.74E-06	gdT
CCDC125	3.76E-10	0.574960707	0.423	0.077	9.26E-06	HSPC
GALNT10	3.97E-10	0.831098662	0.49	0.277	9.78E-06	Naive B

RPS6	4.18E-10	0.383649434	0.985	0.932	1.03E-05	MAIT
NUCB2	4.27E-10	0.714687499	0.808	0.225	1.05E-05	HSPC
SDHAF3	4.46E-10	0.267600824	0.208	0.059	1.10E-05	pDC
ZNF43	5.20E-10	0.378014902	0.538	0.111	1.28E-05	HSPC
ZNF827	5.67E-10	0.413867366	0.228	0.119	1.40E-05	CD8 TEM_1
CEP128	7.11E-10	0.551221818	0.231	0.125	1.75E-05	CD8 TEM_1
CUBN	7.39E-10	0.258648965	0.14	0.091	1.82E-05	CD8 Naive
HHAT	8.06E-10	0.468180752	0.154	0.069	1.98E-05	CD8 TEM_1
CPLANE1	8.27E-10	0.357066113	0.207	0.117	2.04E-05	NK
BTBD11	1.07E-09	0.443328501	0.337	0.195	2.64E-05	CD4 TEM
PIP5K1B	1.09E-09	0.372950529	0.33	0.123	2.69E-05	pDC
CPLANE1	1.26E-09	0.581316229	0.538	0.12	3.09E-05	HSPC
C19orf12	1.33E-09	0.272699313	0.19	0.103	3.28E-05	NK
LINC01934	1.38E-09	0.554381707	0.48	0.265	3.39E-05	gdT
FMNL2	1.41E-09	0.264229479	0.245	0.079	3.47E-05	pDC
NUCB2	1.46E-09	0.957536433	0.889	0.225	3.59E-05	Plasma
ZNF827	1.51E-09	0.359873399	0.224	0.119	3.71E-05	CD8 TEM_2
CLMN	1.56E-09	0.27786364	0.274	0.153	3.83E-05	Intermediate B
STAM2	1.67E-09	0.410623044	0.387	0.161	4.11E-05	pDC
TNS3	2.10E-09	0.519795685	0.538	0.257	5.18E-05	pDC
L3MBTL4	2.14E-09	0.53204922	0.462	0.095	5.27E-05	HSPC
TOR3A	2.18E-09	0.433609055	0.5	0.083	5.37E-05	Plasma
HPF1	2.39E-09	0.451637717	0.462	0.094	5.87E-05	HSPC
CCDC152	2.42E-09	0.330541633	0.346	0.058	5.96E-05	HSPC
ADAMTS6	2.68E-09	0.45089135	0.189	0.065	6.60E-05	Naive B
SRPRB	2.81E-09	0.425486989	0.5	0.083	6.91E-05	Plasma
CREB3L2	3.23E-09	0.579554237	0.667	0.137	7.95E-05	Plasma
SMC4	3.95E-09	0.434399802	0.415	0.179	9.73E-05	pDC
LINC01184	4.00E-09	0.50008032	0.35	0.165	9.85E-05	Naive B
MZT2A	4.16E-09	0.325431928	0.245	0.196	0.000102476	CD4 Naive
MICU3	4.36E-09	0.33884686	0.154	0.073	0.00010735	Memory B
NT5DC1	4.57E-09	0.336940008	0.322	0.195	0.000112388	Intermediate B
ZHX2	4.80E-09	0.319618799	0.4	0.355	0.000118148	CD4 Naive
MCTP2	4.98E-09	0.697715802	0.808	0.248	0.000122671	HSPC
MICU3	5.16E-09	0.263944188	0.112	0.07	0.000127062	CD4 Naive
PRAG1	5.51E-09	0.286050967	0.172	0.122	0.00013573	CD4 Naive
ANXA2R	5.55E-09	0.297221872	0.162	0.115	0.000136501	CD4 Naive
AL390957.1	5.56E-09	0.289429979	0.217	0.069	0.000136951	pDC
FRMD4B	5.70E-09	0.978170648	0.654	0.193	0.000140362	HSPC
GALNT10	6.53E-09	0.339303011	0.416	0.275	0.00016062	Intermediate B
CD302	7.60E-09	0.359343706	0.415	0.183	0.000186942	pDC
CCNG2	7.76E-09	0.367314739	0.132	0.062	0.000191044	Memory B
KCNQ5	7.78E-09	0.341219101	0.337	0.219	0.000191456	NK
SLC39A10	8.01E-09	0.460322739	0.491	0.236	0.000197224	pDC
DAAM1	8.34E-09	0.426643513	0.236	0.132	0.000205373	Intermediate B
GABPB1-AS1	8.42E-09	0.366874602	0.257	0.207	0.000207342	CD4 Naive
ABCB1	8.57E-09	0.397626343	0.385	0.072	0.000210955	HSPC
ARHGAP10	8.83E-09	0.38946914	0.235	0.144	0.000217295	NK
TAFA1	9.52E-09	0.427146044	0.373	0.236	0.000234312	CD8 TEM_1
MAF	1.01E-08	0.56682776	0.213	0.087	0.000249328	Treg

EEF1A1	1.03E-08	0.311254934	1	0.99	0.000252806	Naive B
CREB3L2	1.26E-08	0.370624388	0.577	0.137	0.000309907	HSPC
AGAP1	1.39E-08	0.381349607	0.13	0.056	0.000342074	CD8 TEM_1
ZNF827	1.41E-08	0.505410586	0.28	0.121	0.000345822	Naive B
CD96	1.60E-08	0.402825611	0.689	0.444	0.000394447	gdT
PKP4	2.04E-08	0.342749956	0.236	0.127	0.00050166	CD4 TEM
VOPP1	2.09E-08	0.506470545	0.604	0.436	0.000513907	Treg
CDK14	2.41E-08	1.089349993	0.722	0.179	0.00059354	Plasma
TTC12	2.55E-08	0.253700041	0.103	0.064	0.000626667	CD4 Naive
ZBTB20-AS5	2.59E-08	0.533090195	0.231	0.096	0.000638	Naive B
LINC01184	2.95E-08	0.347617842	0.279	0.164	0.000725754	Intermediate B
KCNQ5	3.20E-08	0.784767984	0.731	0.223	0.000786932	HSPC
GPHN	3.39E-08	0.289042542	0.425	0.279	0.000835515	Intermediate B
SRPRB	3.43E-08	0.287362704	0.236	0.082	0.000844929	pDC
CCR6	3.58E-08	0.409875686	0.171	0.064	0.000881602	Treg
EPB41L2	3.61E-08	0.416358093	0.259	0.112	0.000887304	Naive B
NIPAL2	3.61E-08	0.50114439	0.196	0.075	0.00088795	Naive B
YES1	3.82E-08	0.538533915	0.189	0.073	0.000941373	gdT
PVT1	4.30E-08	0.612963653	0.503	0.308	0.001057228	Naive B
JUN	4.48E-08	0.364426529	0.802	0.677	0.001103474	CD4 TEM
LINC01572	4.58E-08	0.435972997	0.154	0.078	0.001126402	Memory B
AC253572.2	4.74E-08	0.400702382	0.462	0.218	0.001166092	pDC
AZIN1-AS1	5.99E-08	0.367038028	0.154	0.052	0.001473237	Naive B
IMMP2L	7.15E-08	0.371500348	0.618	0.485	0.001759222	Intermediate B
SMC4	9.08E-08	0.692560815	0.722	0.18	0.002235075	Plasma
MARCH3	9.45E-08	0.745317161	0.462	0.109	0.002326085	HSPC
TRIO	9.87E-08	0.967195728	0.944	0.327	0.002430064	Plasma
RNF144B	1.03E-07	0.440409318	0.358	0.159	0.002540331	pDC
ZHX2	1.07E-07	0.385743948	0.651	0.358	0.002634233	pDC
AC002460.2	1.14E-07	0.354213312	0.101	0.064	0.002799352	CD4 Naive
RASGRP1	1.30E-07	0.512093724	0.555	0.333	0.003200963	MAIT
MZT2A	1.38E-07	0.481602004	0.354	0.2	0.003403122	Treg
TBC1D19	1.44E-07	0.378324185	0.23	0.1	0.003546626	gdT
WWOX	1.66E-07	0.655201311	0.58	0.495	0.004076766	CD8 TEM_1
PVT1	1.67E-07	0.64249102	0.463	0.308	0.004114665	Treg
TXN	1.68E-07	0.285137	0.312	0.19	0.004136677	CD4 TEM
CD38	1.77E-07	0.532501621	0.423	0.1	0.004344224	HSPC
WRN	1.83E-07	0.55074014	0.615	0.179	0.004506502	HSPC
BBS9	2.19E-07	0.270405072	0.343	0.28	0.005387578	CD4 TCM
PGM1	2.26E-07	0.25899296	0.156	0.088	0.005567729	NK
BMP2K	2.29E-07	0.740538112	0.808	0.28	0.005634984	HSPC
ZNF254	2.34E-07	0.412608227	0.538	0.14	0.005762986	HSPC
NDUFAB1	2.52E-07	0.267188703	0.257	0.147	0.006213052	CD4 TEM
RPS13	2.64E-07	0.327003057	0.964	0.887	0.006495284	MAIT
CYTOR	2.64E-07	0.724351727	0.341	0.204	0.006506917	Treg
TSHZ1	2.87E-07	0.302241376	0.215	0.136	0.007063732	NK
ABCA1	3.12E-07	0.500364257	0.385	0.087	0.007681118	HSPC
DZIP3	3.17E-07	0.335307097	0.188	0.103	0.007792227	CD8 TEM_1
MZT2B	3.17E-07	0.313967364	0.38	0.36	0.007808037	CD4 Naive
YES1	3.33E-07	0.33503617	0.19	0.073	0.008198479	MAIT

ENOSF1	3.47E-07	0.520793716	0.654	0.197	0.008540392	HSPC
SEMA6A-AS1	3.47E-07	0.250827914	0.217	0.077	0.008550204	pDC
DTWD2	3.97E-07	0.268764731	0.132	0.066	0.009765545	Memory B

**type**
Down-Regulated
Down-Regulated
Down-Regulated
Down-Regulated
Down-Regulated
Down-Regulated
Down-Regulated
Down-Regulated
Down-Regulated
Down-Regulated
Down-Regulated
Down-Regulated
Down-Regulated
Down-Regulated
Down-Regulated
Down-Regulated
Down-Regulated
Down-Regulated
Down-Regulated
Down-Regulated
Down-Regulated
Down-Regulated
Down-Regulated
Down-Regulated
Down-Regulated
Down-Regulated
Down-Regulated
Down-Regulated
Down-Regulated
Down-Regulated
Down-Regulated
Down-Regulated
Down-Regulated
Down-Regulated
Down-Regulated
Down-Regulated
Down-Regulated
Down-Regulated
Down-Regulated
Down-Regulated
Down-Regulated
Down-Regulated
Down-Regulated
Down-Regulated
Down-Regulated
Down-Regulated
Down-Regulated
Down-Regulated
Down-Regulated

Down-Regulated
Down-Regulated
Down-Regulated
Down-Regulated
Down-Regulated
Down-Regulated
Down-Regulated
Down-Regulated
Down-Regulated
Down-Regulated
Down-Regulated
Down-Regulated
Down-Regulated
Down-Regulated
Down-Regulated
Down-Regulated
Down-Regulated
Down-Regulated
Down-Regulated
Down-Regulated
Down-Regulated
Down-Regulated
Down-Regulated
Down-Regulated
Down-Regulated
Down-Regulated
Down-Regulated
Down-Regulated
Down-Regulated
Down-Regulated
Down-Regulated
Down-Regulated
Down-Regulated
Down-Regulated
Down-Regulated
Down-Regulated
Down-Regulated
Down-Regulated
Down-Regulated
Down-Regulated
Down-Regulated
Down-Regulated
Down-Regulated
Down-Regulated
Down-Regulated
Down-Regulated
Down-Regulated
Down-Regulated
Down-Regulated
Down-Regulated

Down-Regulated
Down-Regulated
Down-Regulated
Down-Regulated
Down-Regulated
Down-Regulated
Down-Regulated
Down-Regulated
Down-Regulated
Down-Regulated
Down-Regulated
Down-Regulated
Down-Regulated
Down-Regulated
Down-Regulated
Down-Regulated
Down-Regulated
Down-Regulated
Down-Regulated
Down-Regulated
Down-Regulated
Down-Regulated
Down-Regulated
Down-Regulated
Down-Regulated
Down-Regulated
Down-Regulated
Down-Regulated
Down-Regulated
Down-Regulated
Down-Regulated
Down-Regulated
Down-Regulated
Down-Regulated
Down-Regulated
Down-Regulated
Down-Regulated
Down-Regulated
Down-Regulated
Down-Regulated
Down-Regulated
Down-Regulated
Down-Regulated
Down-Regulated
Down-Regulated
Down-Regulated
Down-Regulated
Down-Regulated
Down-Regulated
Down-Regulated

Down-Regulated
Down-Regulated
Down-Regulated
Down-Regulated
Down-Regulated
Down-Regulated
Down-Regulated
Down-Regulated
Down-Regulated
Down-Regulated
Down-Regulated
Down-Regulated
Down-Regulated
Down-Regulated
Down-Regulated
Down-Regulated
Down-Regulated
Down-Regulated
Down-Regulated
Down-Regulated
Down-Regulated
Down-Regulated
Down-Regulated
Down-Regulated
Down-Regulated
Down-Regulated
Down-Regulated
Down-Regulated
Down-Regulated
Down-Regulated
Down-Regulated
Down-Regulated
Down-Regulated
Down-Regulated
Down-Regulated
Down-Regulated
Down-Regulated
Down-Regulated
Down-Regulated
Down-Regulated
Down-Regulated
Down-Regulated
Down-Regulated
Down-Regulated
Down-Regulated
Down-Regulated
Down-Regulated
Down-Regulated
Down-Regulated
Down-Regulated

Down-Regulated
Down-Regulated
Down-Regulated
Down-Regulated
Down-Regulated
Down-Regulated
Down-Regulated
Down-Regulated
Down-Regulated
Down-Regulated
Down-Regulated
Down-Regulated
Down-Regulated
Down-Regulated
Down-Regulated
Down-Regulated
Down-Regulated
Down-Regulated
Down-Regulated
Down-Regulated
Down-Regulated
Down-Regulated
Down-Regulated
Down-Regulated
Down-Regulated
Down-Regulated
Down-Regulated
Down-Regulated
Down-Regulated
Down-Regulated
Down-Regulated
Down-Regulated
Down-Regulated
Down-Regulated
Down-Regulated
Down-Regulated
Down-Regulated
Down-Regulated
Down-Regulated
Down-Regulated
Down-Regulated
Down-Regulated
Down-Regulated
Down-Regulated
Down-Regulated
Down-Regulated
Down-Regulated
Down-Regulated
Down-Regulated
Down-Regulated

Down-Regulated
Down-Regulated
Down-Regulated
Down-Regulated
Down-Regulated
Down-Regulated
Down-Regulated
Down-Regulated
Down-Regulated
Down-Regulated
Down-Regulated
Down-Regulated
Down-Regulated
Down-Regulated
Down-Regulated
Down-Regulated
Down-Regulated
Down-Regulated
Down-Regulated
Down-Regulated
Down-Regulated
Down-Regulated
Down-Regulated
Down-Regulated
Down-Regulated
Down-Regulated
Down-Regulated
Down-Regulated
Down-Regulated
Down-Regulated
Down-Regulated
Down-Regulated
Down-Regulated
Down-Regulated
Down-Regulated
Down-Regulated
Down-Regulated
Down-Regulated
Down-Regulated
Down-Regulated
Down-Regulated
Down-Regulated
Down-Regulated
Down-Regulated
Down-Regulated
Down-Regulated
Down-Regulated
Down-Regulated
Down-Regulated
Down-Regulated

Down-Regulated
Down-Regulated
Down-Regulated
Down-Regulated
Down-Regulated
Down-Regulated
Down-Regulated
Down-Regulated
Down-Regulated
Down-Regulated
Down-Regulated
Down-Regulated
Down-Regulated
Down-Regulated
Down-Regulated
Down-Regulated
Down-Regulated
Down-Regulated
Down-Regulated
Down-Regulated
Down-Regulated
Down-Regulated
Down-Regulated
Down-Regulated
Down-Regulated
Down-Regulated
Down-Regulated
Down-Regulated
Down-Regulated
Down-Regulated
Down-Regulated
Down-Regulated
Down-Regulated
Down-Regulated
Down-Regulated
Down-Regulated
Down-Regulated
Down-Regulated
Down-Regulated
Down-Regulated
Down-Regulated
Down-Regulated
Down-Regulated
Down-Regulated
Down-Regulated
Down-Regulated
Down-Regulated
Down-Regulated
Down-Regulated
Down-Regulated

Down-Regulated
Down-Regulated
Down-Regulated
Down-Regulated
Down-Regulated
Down-Regulated
Down-Regulated
Down-Regulated
Down-Regulated
Down-Regulated
Down-Regulated
Down-Regulated
Down-Regulated
Down-Regulated
Down-Regulated
Down-Regulated
Down-Regulated
Down-Regulated
Down-Regulated
Down-Regulated
Down-Regulated
Down-Regulated
Down-Regulated
Down-Regulated
Down-Regulated
Down-Regulated
Down-Regulated
Down-Regulated
Down-Regulated
Down-Regulated
Down-Regulated
Down-Regulated
Down-Regulated
Down-Regulated
Down-Regulated
Down-Regulated
Down-Regulated
Down-Regulated
Down-Regulated
Down-Regulated
Down-Regulated
Down-Regulated
Down-Regulated
Down-Regulated
Down-Regulated
Down-Regulated
Down-Regulated
Down-Regulated
Down-Regulated
Down-Regulated

Down-Regulated
Down-Regulated
Down-Regulated
Down-Regulated
Down-Regulated
Down-Regulated
Down-Regulated
Down-Regulated
Down-Regulated
Down-Regulated
Down-Regulated
Down-Regulated
Down-Regulated
Down-Regulated
Down-Regulated
Down-Regulated
Down-Regulated
Down-Regulated
Down-Regulated
Down-Regulated
Down-Regulated
Down-Regulated
Down-Regulated
Down-Regulated
Down-Regulated
Down-Regulated
Down-Regulated
Down-Regulated
Down-Regulated
Down-Regulated
Down-Regulated
Down-Regulated
Down-Regulated
Down-Regulated
Down-Regulated
Down-Regulated
Down-Regulated
Down-Regulated
Down-Regulated
Down-Regulated
Down-Regulated
Down-Regulated
Down-Regulated
Down-Regulated
Down-Regulated
Down-Regulated
Down-Regulated
Down-Regulated
Down-Regulated
Down-Regulated

Down-Regulated
Down-Regulated
Down-Regulated
Down-Regulated
Down-Regulated
Down-Regulated
Down-Regulated
Down-Regulated
Down-Regulated
Down-Regulated
Down-Regulated
Down-Regulated
Down-Regulated
Down-Regulated
Down-Regulated
Down-Regulated
Down-Regulated
Down-Regulated
Down-Regulated
Down-Regulated
Down-Regulated
Down-Regulated
Down-Regulated
Down-Regulated
Down-Regulated
Down-Regulated
Down-Regulated
Down-Regulated
Down-Regulated
Down-Regulated
Down-Regulated
Down-Regulated
Down-Regulated
Down-Regulated
Down-Regulated
Down-Regulated
Down-Regulated
Down-Regulated
Down-Regulated
Down-Regulated
Down-Regulated
Down-Regulated
Down-Regulated
Down-Regulated
Down-Regulated
Down-Regulated
Down-Regulated
Down-Regulated
Down-Regulated
Down-Regulated

Down-Regulated
Down-Regulated
Down-Regulated
Down-Regulated
Down-Regulated
Down-Regulated
Down-Regulated
Down-Regulated
Down-Regulated
Down-Regulated
Down-Regulated
Down-Regulated
Down-Regulated
Down-Regulated
Down-Regulated
Down-Regulated
Down-Regulated
Down-Regulated
Down-Regulated
Down-Regulated
Down-Regulated
Down-Regulated
Down-Regulated
Down-Regulated
Down-Regulated
Down-Regulated
Down-Regulated
Down-Regulated
Down-Regulated
Down-Regulated
Down-Regulated
Down-Regulated
Down-Regulated
Down-Regulated
Down-Regulated
Down-Regulated
Down-Regulated
Down-Regulated
Down-Regulated
Down-Regulated
Down-Regulated
Down-Regulated
Down-Regulated
Down-Regulated
Down-Regulated
Down-Regulated
Down-Regulated
Down-Regulated
Down-Regulated
Down-Regulated

Down-Regulated
Down-Regulated
Down-Regulated
Down-Regulated
Down-Regulated
Down-Regulated
Down-Regulated
Down-Regulated
Down-Regulated
Down-Regulated
Down-Regulated
Down-Regulated
Down-Regulated
Down-Regulated
Down-Regulated
Down-Regulated
Down-Regulated
Down-Regulated
Down-Regulated
Down-Regulated
Down-Regulated
Down-Regulated
Down-Regulated
Down-Regulated
Down-Regulated
Down-Regulated
Down-Regulated
Down-Regulated
Down-Regulated
Down-Regulated
Down-Regulated
Down-Regulated
Down-Regulated
Down-Regulated
Down-Regulated
Down-Regulated
Down-Regulated
Down-Regulated
Down-Regulated
Down-Regulated
Down-Regulated
Down-Regulated
Down-Regulated
Down-Regulated
Down-Regulated
Down-Regulated
Down-Regulated
Down-Regulated
Down-Regulated
Down-Regulated

Down-Regulated
Down-Regulated
Down-Regulated
Down-Regulated
Down-Regulated
Down-Regulated
Down-Regulated
Down-Regulated
Down-Regulated
Down-Regulated
Down-Regulated
Down-Regulated
Down-Regulated
Down-Regulated
Down-Regulated
Down-Regulated
Down-Regulated
Down-Regulated
Down-Regulated
Down-Regulated
Down-Regulated
Down-Regulated
Down-Regulated
Down-Regulated
Down-Regulated
Down-Regulated
Down-Regulated
Down-Regulated
Down-Regulated
Down-Regulated
Down-Regulated
Down-Regulated
Down-Regulated
Down-Regulated
Down-Regulated
Down-Regulated
Down-Regulated
Down-Regulated
Down-Regulated
Down-Regulated
Down-Regulated
Down-Regulated
Down-Regulated
Down-Regulated
Down-Regulated
Down-Regulated
Down-Regulated
Down-Regulated
Down-Regulated
Down-Regulated

Down-Regulated
Down-Regulated
Down-Regulated
Down-Regulated
Down-Regulated
Down-Regulated
Down-Regulated
Down-Regulated
Down-Regulated
Down-Regulated
Down-Regulated
Down-Regulated
Down-Regulated
Down-Regulated
Down-Regulated
Down-Regulated
Down-Regulated
Down-Regulated
Down-Regulated
Down-Regulated
Down-Regulated
Down-Regulated
Down-Regulated
Down-Regulated
Down-Regulated
Down-Regulated
Down-Regulated
Down-Regulated
Down-Regulated
Down-Regulated
Down-Regulated
Down-Regulated
Down-Regulated
Down-Regulated
Down-Regulated
Down-Regulated
Down-Regulated
Down-Regulated
Down-Regulated
Down-Regulated
Down-Regulated
Down-Regulated
Down-Regulated
Down-Regulated
Down-Regulated
Down-Regulated
Down-Regulated
Down-Regulated
Down-Regulated
Down-Regulated

Down-Regulated
Down-Regulated
Down-Regulated
Down-Regulated
Down-Regulated
Down-Regulated
Down-Regulated
Down-Regulated
Down-Regulated
Down-Regulated
Down-Regulated
Down-Regulated
Down-Regulated
Down-Regulated
Down-Regulated
Down-Regulated
Down-Regulated
Down-Regulated
Down-Regulated
Down-Regulated
Down-Regulated
Down-Regulated
Down-Regulated
Down-Regulated
Down-Regulated
Down-Regulated
Down-Regulated
Down-Regulated
Down-Regulated
Down-Regulated
Down-Regulated
Down-Regulated
Down-Regulated
Down-Regulated
Down-Regulated
Down-Regulated
Down-Regulated
Down-Regulated
Down-Regulated
Down-Regulated
Down-Regulated
Down-Regulated
Down-Regulated
Down-Regulated
Down-Regulated
Down-Regulated
Down-Regulated
Down-Regulated
Down-Regulated
Down-Regulated

Down-Regulated
Down-Regulated
Down-Regulated
Down-Regulated
Down-Regulated
Down-Regulated
Down-Regulated
Down-Regulated
Down-Regulated
Down-Regulated
Down-Regulated
Down-Regulated
Down-Regulated
Down-Regulated
Down-Regulated
Down-Regulated
Down-Regulated
Down-Regulated
Down-Regulated
Down-Regulated
Down-Regulated
Down-Regulated
Down-Regulated
Down-Regulated
Down-Regulated
Down-Regulated
Down-Regulated
Down-Regulated
Down-Regulated
Down-Regulated
Down-Regulated
Down-Regulated
Down-Regulated
Down-Regulated
Down-Regulated
Down-Regulated
Down-Regulated
Down-Regulated
Down-Regulated
Down-Regulated
Down-Regulated
Down-Regulated
Down-Regulated
Down-Regulated
Down-Regulated
Down-Regulated
Down-Regulated
Down-Regulated
Down-Regulated
Down-Regulated

Down-Regulated
Down-Regulated
Down-Regulated
Down-Regulated
Down-Regulated
Down-Regulated
Down-Regulated
Down-Regulated
Down-Regulated
Down-Regulated
Down-Regulated
Down-Regulated
Down-Regulated
Down-Regulated
Down-Regulated
Down-Regulated
Down-Regulated
Down-Regulated
Down-Regulated
Down-Regulated
Down-Regulated
Down-Regulated
Down-Regulated
Down-Regulated
Down-Regulated
Down-Regulated
Down-Regulated
Down-Regulated
Down-Regulated
Down-Regulated
Down-Regulated
Down-Regulated
Down-Regulated
Down-Regulated
Down-Regulated
Down-Regulated
Down-Regulated
Down-Regulated
Down-Regulated
Down-Regulated
Down-Regulated
Down-Regulated
Down-Regulated
Down-Regulated
Down-Regulated
Down-Regulated
Down-Regulated
Down-Regulated
Down-Regulated
Down-Regulated

Down-Regulated
Down-Regulated
Down-Regulated
Down-Regulated
Down-Regulated
Down-Regulated
Down-Regulated
Down-Regulated
Down-Regulated
Down-Regulated
Down-Regulated
Down-Regulated
Down-Regulated
Down-Regulated
Down-Regulated
Down-Regulated
Down-Regulated
Down-Regulated
Down-Regulated
Down-Regulated
Down-Regulated
Down-Regulated
Down-Regulated
Down-Regulated
Down-Regulated
Down-Regulated
Down-Regulated
Down-Regulated
Down-Regulated
Down-Regulated
Down-Regulated
Down-Regulated
Down-Regulated
Down-Regulated
Down-Regulated
Down-Regulated
Down-Regulated
Down-Regulated
Down-Regulated
Down-Regulated
Down-Regulated
Down-Regulated
Down-Regulated
Down-Regulated
Down-Regulated
Down-Regulated
Down-Regulated
Down-Regulated
Down-Regulated
Down-Regulated

Down-Regulated
Down-Regulated
Down-Regulated
Down-Regulated
Down-Regulated
Down-Regulated
Down-Regulated
Down-Regulated
Down-Regulated
Down-Regulated
Down-Regulated
Down-Regulated
Down-Regulated
Down-Regulated
Down-Regulated
Down-Regulated
Down-Regulated
Down-Regulated
Down-Regulated
Down-Regulated
Down-Regulated
Down-Regulated
Down-Regulated
Down-Regulated
Down-Regulated
Down-Regulated
Down-Regulated
Down-Regulated
Down-Regulated
Down-Regulated
Down-Regulated
Down-Regulated
Down-Regulated
Down-Regulated
Down-Regulated
Down-Regulated
Down-Regulated
Down-Regulated
Down-Regulated
Down-Regulated
Down-Regulated
Down-Regulated
Down-Regulated
Down-Regulated
Down-Regulated
Down-Regulated
Down-Regulated
Down-Regulated
Down-Regulated
Down-Regulated

Down-Regulated
Down-Regulated
Down-Regulated
Down-Regulated
Down-Regulated
Down-Regulated
Down-Regulated
Down-Regulated
Down-Regulated
Down-Regulated
Down-Regulated
Down-Regulated
Down-Regulated
Down-Regulated
Down-Regulated
Down-Regulated
Down-Regulated
Down-Regulated
Down-Regulated
Down-Regulated
Down-Regulated
Down-Regulated
Down-Regulated
Down-Regulated
Down-Regulated
Down-Regulated
Down-Regulated
Down-Regulated
Down-Regulated
Down-Regulated
Down-Regulated
Down-Regulated
Down-Regulated
Down-Regulated
Down-Regulated
Down-Regulated
Down-Regulated
Down-Regulated
Down-Regulated
Down-Regulated
Down-Regulated
Down-Regulated
Down-Regulated
Down-Regulated
Down-Regulated
Down-Regulated
Down-Regulated
Down-Regulated
Down-Regulated
Down-Regulated

Down-Regulated
Down-Regulated
Down-Regulated
Down-Regulated
Down-Regulated
Down-Regulated
Down-Regulated
Down-Regulated
Down-Regulated
Down-Regulated
Down-Regulated
Down-Regulated
Down-Regulated
Down-Regulated
Down-Regulated
Down-Regulated
Down-Regulated
Not Significant
Not Significant
Not Significant
Not Significant
Not Significant
Not Significant
Not Significant
Not Significant
Not Significant
Not Significant
Not Significant
Not Significant
Not Significant
Not Significant
Not Significant
Not Significant
Not Significant
Not Significant
Not Significant
Not Significant
Not Significant
Not Significant
Not Significant
Not Significant
Not Significant
Not Significant
Not Significant
Not Significant
Not Significant
Not Significant
Not Significant
Not Significant
Not Significant
Not Significant

Not Significant
Not Significant
Not Significant
Not Significant
Not Significant
Not Significant
Not Significant
Not Significant
Not Significant
Not Significant
Not Significant
Not Significant
Not Significant
Not Significant
Not Significant
Not Significant
Not Significant
Not Significant
Not Significant
Not Significant
Not Significant
Not Significant
Not Significant
Not Significant
Not Significant
Not Significant
Not Significant
Not Significant
Not Significant
Not Significant
Not Significant
Not Significant
Not Significant
Not Significant
Not Significant
Not Significant
Not Significant
Not Significant
Not Significant
Not Significant
Not Significant
Not Significant
Not Significant
Not Significant
Not Significant
Not Significant
Not Significant
Not Significant
Not Significant
Not Significant

Not Significant
Not Significant
Not Significant
Not Significant
Not Significant
Not Significant
Not Significant
Not Significant
Not Significant
Not Significant
Not Significant
Not Significant
Not Significant
Not Significant
Not Significant
Not Significant
Not Significant
Not Significant
Not Significant
Not Significant
Not Significant
Not Significant
Not Significant
Not Significant
Not Significant
Not Significant
Not Significant
Not Significant
Not Significant
Top DEGs
Top DEGs
Top DEGs
Top DEGs
Top DEGs
Top DEGs
Top DEGs
Top DEGs
Top DEGs
Top DEGs
Top DEGs
Top DEGs
Top DEGs
Top DEGs
Top DEGs
Top DEGs
Top DEGs
Top DEGs
Top DEGs
Top DEGs
Top DEGs

Top DEGs
Top DEGs
Top DEGs
Top DEGs
Top DEGs
Top DEGs
Top DEGs
Top DEGs
Top DEGs
Top DEGs
Top DEGs
Top DEGs
Top DEGs
Top DEGs
Top DEGs
Top DEGs
Top DEGs
Top DEGs
Top DEGs
Top DEGs
Top DEGs
Top DEGs
Top DEGs
Top DEGs
Top DEGs
Top DEGs
Top DEGs
Top DEGs
Top DEGs
Top DEGs
Top DEGs
Top DEGs
Top DEGs
Top DEGs
Top DEGs
Top DEGs
Top DEGs
Top DEGs
Top DEGs
Top DEGs
Top DEGs
Top DEGs
Top DEGs
Top DEGs
Top DEGs
Top DEGs
Top DEGs
Top DEGs
Top DEGs
Top DEGs

Top DEGs
Top DEGs
Top DEGs
Top DEGs
Top DEGs
Top DEGs
Top DEGs
Top DEGs
Top DEGs
Top DEGs
Top DEGs
Top DEGs
Top DEGs
Top DEGs
Top DEGs
Top DEGs
Top DEGs
Top DEGs
Top DEGs
Top DEGs
Top DEGs
Top DEGs
Top DEGs
Top DEGs
Top DEGs
Top DEGs
Top DEGs
Top DEGs
Top DEGs
Top DEGs
Top DEGs
Top DEGs
Top DEGs
Top DEGs
Top DEGs
Top DEGs
Top DEGs
Top DEGs
Top DEGs
Top DEGs
Top DEGs
Top DEGs
Top DEGs
Top DEGs
Top DEGs
Top DEGs
Top DEGs
Top DEGs
Top DEGs
Top DEGs

Top DEGs
Top DEGs
Top DEGs
Top DEGs
Top DEGs
Top DEGs
Top DEGs
Top DEGs
Top DEGs
Top DEGs
Top DEGs
Top DEGs
Top DEGs
Top DEGs
Top DEGs
Top DEGs
Top DEGs
Top DEGs
Top DEGs
Top DEGs
Top DEGs
Top DEGs
Top DEGs
Top DEGs
Top DEGs
Top DEGs
Top DEGs
Top DEGs
Top DEGs
Top DEGs
Top DEGs
Top DEGs
Top DEGs
Top DEGs
Top DEGs
Top DEGs
Top DEGs
Top DEGs
Top DEGs
Top DEGs
Top DEGs
Top DEGs
Top DEGs
Top DEGs
Top DEGs
Top DEGs
Top DEGs
Top DEGs
Top DEGs
Top DEGs

Top DEGs
Top DEGs
Top DEGs
Top DEGs
Top DEGs
Top DEGs
Top DEGs
Top DEGs
Top DEGs
Top DEGs
Top DEGs
Top DEGs
Top DEGs
Top DEGs
Top DEGs
Top DEGs
Top DEGs
Top DEGs
Top DEGs
Top DEGs
Top DEGs
Top DEGs
Top DEGs
Top DEGs
Top DEGs
Top DEGs
Top DEGs
Top DEGs
Top DEGs
Top DEGs
Top DEGs
Top DEGs
Top DEGs
Top DEGs
Top DEGs
Top DEGs
Top DEGs
Top DEGs
Top DEGs
Top DEGs
Top DEGs
Top DEGs
Top DEGs
Top DEGs
Top DEGs
Top DEGs
Top DEGs
Top DEGs
Top DEGs
Top DEGs

Top DEGs
Top DEGs
Top DEGs
Top DEGs
Top DEGs
Top DEGs
Top DEGs
Top DEGs
Top DEGs
Top DEGs
Top DEGs
Top DEGs
Top DEGs
Top DEGs
Top DEGs
Top DEGs
Top DEGs
Top DEGs
Top DEGs
Top DEGs
Top DEGs
Top DEGs
Top DEGs
Top DEGs
Top DEGs
Top DEGs
Top DEGs
Top DEGs
Top DEGs
Top DEGs
Top DEGs
Top DEGs
Top DEGs
Top DEGs
Top DEGs
Top DEGs
Top DEGs
Top DEGs
Top DEGs
Top DEGs
Top DEGs
Top DEGs
Top DEGs
Top DEGs
Top DEGs
Top DEGs
Top DEGs
Top DEGs
Top DEGs
Top DEGs

Top DEGs
Top DEGs
Top DEGs
Top DEGs
Top DEGs
Top DEGs
Top DEGs
Top DEGs
Top DEGs
Top DEGs
Top DEGs
Top DEGs
Top DEGs
Top DEGs
Top DEGs
Top DEGs
Top DEGs
Top DEGs
Top DEGs
Top DEGs
Top DEGs
Top DEGs
Top DEGs
Top DEGs
Top DEGs
Top DEGs
Top DEGs
Top DEGs
Top DEGs
Top DEGs
Top DEGs
Top DEGs
Top DEGs
Top DEGs
Top DEGs
Top DEGs
Top DEGs
Top DEGs
Top DEGs
Top DEGs
Top DEGs
Top DEGs
Top DEGs
Top DEGs
Top DEGs
Top DEGs
Top DEGs
Top DEGs
Top DEGs
Top DEGs

Top DEGs
Top DEGs
Top DEGs
Top DEGs
Top DEGs
Top DEGs
Top DEGs
Top DEGs
Top DEGs
Top DEGs
Top DEGs
Top DEGs
Top DEGs
Top DEGs
Top DEGs
Top DEGs
Top DEGs
Top DEGs
Top DEGs
Top DEGs
Top DEGs
Top DEGs
Top DEGs
Top DEGs
Top DEGs
Top DEGs
Top DEGs
Top DEGs
Top DEGs
Top DEGs
Top DEGs
Top DEGs
Top DEGs
Top DEGs
Top DEGs
Top DEGs
Top DEGs
Top DEGs
Top DEGs
Top DEGs
Top DEGs
Top DEGs
Top DEGs
Top DEGs
Top DEGs
Top DEGs
Top DEGs
Top DEGs
Top DEGs
Top DEGs

Top DEGs
Top DEGs
Top DEGs
Top DEGs
Top DEGs
Top DEGs
Top DEGs
Top DEGs
Top DEGs
Top DEGs
Top DEGs
Top DEGs
Top DEGs
Top DEGs
Top DEGs
Top DEGs
Top DEGs
Top DEGs
Top DEGs
Top DEGs
Top DEGs
Top DEGs
Top DEGs
Top DEGs
Top DEGs
Top DEGs
Top DEGs
Top DEGs
Top DEGs
Top DEGs
Top DEGs
Top DEGs
Top DEGs
Top DEGs
Top DEGs
Top DEGs
Top DEGs
Top DEGs
Top DEGs
Top DEGs
Top DEGs
Top DEGs
Top DEGs
Top DEGs
Top DEGs
Top DEGs
Up-Regulated
Up-Regulated
Up-Regulated
Up-Regulated

Up-Regulated
Up-Regulated
Up-Regulated
Up-Regulated
Up-Regulated
Up-Regulated
Up-Regulated
Up-Regulated
Up-Regulated
Up-Regulated
Up-Regulated
Up-Regulated
Up-Regulated
Up-Regulated
Up-Regulated
Up-Regulated
Up-Regulated
Up-Regulated
Up-Regulated
Up-Regulated
Up-Regulated
Up-Regulated
Up-Regulated
Up-Regulated
Up-Regulated
Up-Regulated
Up-Regulated
Up-Regulated
Up-Regulated
Up-Regulated
Up-Regulated
Up-Regulated
Up-Regulated
Up-Regulated
Up-Regulated
Up-Regulated
Up-Regulated
Up-Regulated
Up-Regulated
Up-Regulated
Up-Regulated
Up-Regulated
Up-Regulated
Up-Regulated
Up-Regulated
Up-Regulated
Up-Regulated
Up-Regulated
Up-Regulated
Up-Regulated

Up-Regulated
Up-Regulated
Up-Regulated
Up-Regulated
Up-Regulated
Up-Regulated
Up-Regulated
Up-Regulated
Up-Regulated
Up-Regulated
Up-Regulated
Up-Regulated
Up-Regulated
Up-Regulated
Up-Regulated
Up-Regulated
Up-Regulated
Up-Regulated
Up-Regulated
Up-Regulated
Up-Regulated
Up-Regulated
Up-Regulated
Up-Regulated
Up-Regulated
Up-Regulated
Up-Regulated
Up-Regulated
Up-Regulated
Up-Regulated
Up-Regulated
Up-Regulated
Up-Regulated
Up-Regulated
Up-Regulated
Up-Regulated
Up-Regulated
Up-Regulated
Up-Regulated
Up-Regulated
Up-Regulated
Up-Regulated
Up-Regulated
Up-Regulated
Up-Regulated
Up-Regulated
Up-Regulated
Up-Regulated
Up-Regulated
Up-Regulated

Up-Regulated
Up-Regulated
Up-Regulated
Up-Regulated
Up-Regulated
Up-Regulated
Up-Regulated
Up-Regulated
Up-Regulated
Up-Regulated
Up-Regulated
Up-Regulated
Up-Regulated
Up-Regulated
Up-Regulated
Up-Regulated
Up-Regulated
Up-Regulated
Up-Regulated
Up-Regulated
Up-Regulated
Up-Regulated
Up-Regulated
Up-Regulated
Up-Regulated
Up-Regulated
Up-Regulated
Up-Regulated
Up-Regulated
Up-Regulated
Up-Regulated
Up-Regulated
Up-Regulated
Up-Regulated
Up-Regulated
Up-Regulated
Up-Regulated
Up-Regulated
Up-Regulated
Up-Regulated
Up-Regulated
Up-Regulated
Up-Regulated
Up-Regulated
Up-Regulated
Up-Regulated
Up-Regulated
Up-Regulated
Up-Regulated
Up-Regulated

Up-Regulated
Up-Regulated
Up-Regulated
Up-Regulated
Up-Regulated
Up-Regulated
Up-Regulated
Up-Regulated
Up-Regulated
Up-Regulated
Up-Regulated
Up-Regulated
Up-Regulated
Up-Regulated
Up-Regulated
Up-Regulated
Up-Regulated
Up-Regulated
Up-Regulated
Up-Regulated
Up-Regulated
Up-Regulated
Up-Regulated
Up-Regulated
Up-Regulated
Up-Regulated
Up-Regulated
Up-Regulated
Up-Regulated
Up-Regulated
Up-Regulated
Up-Regulated
Up-Regulated
Up-Regulated
Up-Regulated
Up-Regulated
Up-Regulated
Up-Regulated
Up-Regulated
Up-Regulated
Up-Regulated
Up-Regulated
Up-Regulated
Up-Regulated
Up-Regulated
Up-Regulated
Up-Regulated
Up-Regulated
Up-Regulated
Up-Regulated

Up-Regulated
Up-Regulated
Up-Regulated
Up-Regulated
Up-Regulated
Up-Regulated
Up-Regulated
Up-Regulated
Up-Regulated
Up-Regulated
Up-Regulated
Up-Regulated
Up-Regulated
Up-Regulated
Up-Regulated
Up-Regulated
Up-Regulated
Up-Regulated
Up-Regulated
Up-Regulated
Up-Regulated
Up-Regulated
Up-Regulated
Up-Regulated
Up-Regulated
Up-Regulated
Up-Regulated
Up-Regulated
Up-Regulated
Up-Regulated
Up-Regulated
Up-Regulated
Up-Regulated
Up-Regulated
Up-Regulated
Up-Regulated
Up-Regulated
Up-Regulated
Up-Regulated
Up-Regulated
Up-Regulated
Up-Regulated
Up-Regulated
Up-Regulated
Up-Regulated
Up-Regulated
Up-Regulated
Up-Regulated
Up-Regulated
Up-Regulated

Up-Regulated
Up-Regulated
Up-Regulated
Up-Regulated
Up-Regulated
Up-Regulated
Up-Regulated
Up-Regulated
Up-Regulated
Up-Regulated
Up-Regulated
Up-Regulated
Up-Regulated
Up-Regulated
Up-Regulated
Up-Regulated
Up-Regulated
Up-Regulated
Up-Regulated
Up-Regulated
Up-Regulated
Up-Regulated
Up-Regulated
Up-Regulated
Up-Regulated
Up-Regulated
Up-Regulated
Up-Regulated
Up-Regulated
Up-Regulated
Up-Regulated
Up-Regulated
Up-Regulated
Up-Regulated
Up-Regulated
Up-Regulated
Up-Regulated
Up-Regulated
Up-Regulated
Up-Regulated
Up-Regulated
Up-Regulated
Up-Regulated
Up-Regulated
Up-Regulated
Up-Regulated
Up-Regulated
Up-Regulated
Up-Regulated
Up-Regulated

Up-Regulated
Up-Regulated
Up-Regulated
Up-Regulated
Up-Regulated
Up-Regulated
Up-Regulated
Up-Regulated
Up-Regulated
Up-Regulated
Up-Regulated
Up-Regulated
Up-Regulated
Up-Regulated
Up-Regulated
Up-Regulated
Up-Regulated
Up-Regulated
Up-Regulated
Up-Regulated
Up-Regulated
Up-Regulated
Up-Regulated
Up-Regulated
Up-Regulated
Up-Regulated
Up-Regulated
Up-Regulated
Up-Regulated
Up-Regulated
Up-Regulated
Up-Regulated
Up-Regulated
Up-Regulated
Up-Regulated
Up-Regulated
Up-Regulated
Up-Regulated
Up-Regulated
Up-Regulated
Up-Regulated
Up-Regulated
Up-Regulated
Up-Regulated
Up-Regulated
Up-Regulated
Up-Regulated
Up-Regulated
Up-Regulated
Up-Regulated

Up-Regulated
Up-Regulated
Up-Regulated
Up-Regulated
Up-Regulated
Up-Regulated
Up-Regulated
Up-Regulated
Up-Regulated
Up-Regulated
Up-Regulated
Up-Regulated
Up-Regulated
Up-Regulated
Up-Regulated
Up-Regulated
Up-Regulated
Up-Regulated
Up-Regulated
Up-Regulated
Up-Regulated
Up-Regulated
Up-Regulated
Up-Regulated
Up-Regulated
Up-Regulated
Up-Regulated
Up-Regulated
Up-Regulated
Up-Regulated
Up-Regulated
Up-Regulated
Up-Regulated
Up-Regulated
Up-Regulated
Up-Regulated
Up-Regulated
Up-Regulated
Up-Regulated
Up-Regulated
Up-Regulated
Up-Regulated
Up-Regulated
Up-Regulated
Up-Regulated
Up-Regulated
Up-Regulated
Up-Regulated
Up-Regulated
Up-Regulated

Up-Regulated
Up-Regulated
Up-Regulated
Up-Regulated
Up-Regulated
Up-Regulated
Up-Regulated
Up-Regulated
Up-Regulated
Up-Regulated
Up-Regulated
Up-Regulated
Up-Regulated
Up-Regulated
Up-Regulated
Up-Regulated
Up-Regulated
Up-Regulated
Up-Regulated
Up-Regulated
Up-Regulated
Up-Regulated
Up-Regulated
Up-Regulated
Up-Regulated
Up-Regulated
Up-Regulated
Up-Regulated
Up-Regulated
Up-Regulated
Up-Regulated
Up-Regulated
Up-Regulated
Up-Regulated
Up-Regulated
Up-Regulated
Up-Regulated
Up-Regulated
Up-Regulated
Up-Regulated
Up-Regulated
Up-Regulated
Up-Regulated
Up-Regulated
Up-Regulated
Up-Regulated
Up-Regulated
Up-Regulated
Up-Regulated
Up-Regulated

Up-Regulated
Up-Regulated
Up-Regulated
Up-Regulated
Up-Regulated
Up-Regulated
Up-Regulated
Up-Regulated
Up-Regulated
Up-Regulated
Up-Regulated
Up-Regulated
Up-Regulated
Up-Regulated
Up-Regulated
Up-Regulated
Up-Regulated
Up-Regulated
Up-Regulated
Up-Regulated
Up-Regulated
Up-Regulated
Up-Regulated
Up-Regulated
Up-Regulated
Up-Regulated
Up-Regulated
Up-Regulated
Up-Regulated
Up-Regulated
Up-Regulated
Up-Regulated
Up-Regulated
Up-Regulated
Up-Regulated
Up-Regulated
Up-Regulated
Up-Regulated
Up-Regulated
Up-Regulated
Up-Regulated
Up-Regulated
Up-Regulated
Up-Regulated
Up-Regulated
Up-Regulated
Up-Regulated
Up-Regulated
Up-Regulated
Up-Regulated

Up-Regulated
Up-Regulated
Up-Regulated
Up-Regulated
Up-Regulated
Up-Regulated
Up-Regulated
Up-Regulated
Up-Regulated
Up-Regulated
Up-Regulated
Up-Regulated
Up-Regulated
Up-Regulated
Up-Regulated
Up-Regulated
Up-Regulated
Up-Regulated
Up-Regulated
Up-Regulated
Up-Regulated
Up-Regulated
Up-Regulated
Up-Regulated
Up-Regulated
Up-Regulated
Up-Regulated
Up-Regulated
Up-Regulated
Up-Regulated
Up-Regulated
Up-Regulated
Up-Regulated
Up-Regulated
Up-Regulated
Up-Regulated
Up-Regulated
Up-Regulated
Up-Regulated
Up-Regulated
Up-Regulated
Up-Regulated
Up-Regulated
Up-Regulated
Up-Regulated
Up-Regulated
Up-Regulated
Up-Regulated
Up-Regulated
Up-Regulated

Up-Regulated
Up-Regulated
Up-Regulated
Up-Regulated
Up-Regulated
Up-Regulated
Up-Regulated
Up-Regulated
Up-Regulated
Up-Regulated
Up-Regulated
Up-Regulated
Up-Regulated
Up-Regulated
Up-Regulated
Up-Regulated
Up-Regulated
Up-Regulated
Up-Regulated
Up-Regulated
Up-Regulated
Up-Regulated
Up-Regulated
Up-Regulated
Up-Regulated
Up-Regulated
Up-Regulated
Up-Regulated
Up-Regulated
Up-Regulated
Up-Regulated
Up-Regulated
Up-Regulated
Up-Regulated
Up-Regulated
Up-Regulated
Up-Regulated
Up-Regulated
Up-Regulated
Up-Regulated
Up-Regulated
Up-Regulated
Up-Regulated
Up-Regulated
Up-Regulated
Up-Regulated
Up-Regulated
Up-Regulated
Up-Regulated
Up-Regulated

Up-Regulated
Up-Regulated
Up-Regulated
Up-Regulated
Up-Regulated
Up-Regulated
Up-Regulated
Up-Regulated
Up-Regulated
Up-Regulated
Up-Regulated
Up-Regulated
Up-Regulated
Up-Regulated
Up-Regulated
Up-Regulated
Up-Regulated
Up-Regulated
Up-Regulated
Up-Regulated
Up-Regulated
Up-Regulated
Up-Regulated
Up-Regulated
Up-Regulated
Up-Regulated
Up-Regulated
Up-Regulated
Up-Regulated
Up-Regulated
Up-Regulated
Up-Regulated
Up-Regulated
Up-Regulated
Up-Regulated
Up-Regulated
Up-Regulated
Up-Regulated
Up-Regulated
Up-Regulated
Up-Regulated
Up-Regulated
Up-Regulated
Up-Regulated
Up-Regulated
Up-Regulated
Up-Regulated
Up-Regulated
Up-Regulated
Up-Regulated

Up-Regulated
Up-Regulated
Up-Regulated
Up-Regulated
Up-Regulated
Up-Regulated
Up-Regulated
Up-Regulated
Up-Regulated
Up-Regulated
Up-Regulated
Up-Regulated
Up-Regulated
Up-Regulated
Up-Regulated
Up-Regulated
Up-Regulated
Up-Regulated
Up-Regulated
Up-Regulated
Up-Regulated
Up-Regulated
Up-Regulated
Up-Regulated
Up-Regulated
Up-Regulated
Up-Regulated
Up-Regulated
Up-Regulated
Up-Regulated
Up-Regulated
Up-Regulated
Up-Regulated
Up-Regulated
Up-Regulated
Up-Regulated
Up-Regulated
Up-Regulated
Up-Regulated
Up-Regulated
Up-Regulated
Up-Regulated
Up-Regulated
Up-Regulated
Up-Regulated
Up-Regulated
Up-Regulated
Up-Regulated
Up-Regulated
Up-Regulated

Up-Regulated
Up-Regulated
Up-Regulated
Up-Regulated
Up-Regulated
Up-Regulated
Up-Regulated
Up-Regulated
Up-Regulated
Up-Regulated
Up-Regulated
Up-Regulated
Up-Regulated
Up-Regulated
Up-Regulated
Up-Regulated
Up-Regulated
Up-Regulated
Up-Regulated
Up-Regulated
Up-Regulated
Up-Regulated
Up-Regulated
Up-Regulated
Up-Regulated
Up-Regulated
Up-Regulated
Up-Regulated
Up-Regulated
Up-Regulated
Up-Regulated
Up-Regulated
Up-Regulated
Up-Regulated
Up-Regulated
Up-Regulated
Up-Regulated
Up-Regulated
Up-Regulated
Up-Regulated
Up-Regulated
Up-Regulated
Up-Regulated
Up-Regulated
Up-Regulated
Up-Regulated
Up-Regulated
Up-Regulated
Up-Regulated
Up-Regulated

Up-Regulated
Up-Regulated
Up-Regulated
Up-Regulated
Up-Regulated
Up-Regulated
Up-Regulated
Up-Regulated
Up-Regulated
Up-Regulated
Up-Regulated
Up-Regulated
Up-Regulated
Up-Regulated
Up-Regulated
Up-Regulated
Up-Regulated
Up-Regulated
Up-Regulated
Up-Regulated
Up-Regulated
Up-Regulated
Up-Regulated
Up-Regulated
Up-Regulated
Up-Regulated
Up-Regulated
Up-Regulated
Up-Regulated
Up-Regulated
Up-Regulated
Up-Regulated
Up-Regulated
Up-Regulated
Up-Regulated
Up-Regulated
Up-Regulated
Up-Regulated
Up-Regulated
Up-Regulated
Up-Regulated
Up-Regulated
Up-Regulated
Up-Regulated
Up-Regulated
Up-Regulated
Up-Regulated
Up-Regulated
Up-Regulated
Up-Regulated

Up-Regulated
Up-Regulated
Up-Regulated

TF	target	importance
Gli3	Basp1	14.03042284
Gli3	Sema4a	5.205243529
Gli3	Myh14	3.359403832
Gli3	Arhgef2	2.484805327
Gli3	Chd3	2.160000118
Gli3	Prkce	1.804799006
Gli3	Bcar1	1.707942738
Gli3	Mapk10	1.468358799
Gli3	Nrarp	1.374711034
Gli3	Igsf9	1.077138176
Gli3	Rap2b	0.853889497
Gli3	St18	0.777731404
Gli3	Arhgap23	0.709926552
Gli3	Sema4c	0.595973292
Gli3	Tmem19	0.490278605
Gli3	Elfn1	0.345066867
Gli3	Atoh8	0.28599868

peak	cluster	membership value
chr13-15479576-15479876	1	0.324835225
chr13-16011825-16012125	1	0.322937603
chr13-15045454-15045754	1	0.338675049
chr13-16012188-16012488	1	0.321358357
chr13-15462519-15462819	1	0.356189171
chr13-15480806-15481106	1	0.363072458
chr13-15411424-15411724	1	0.370874211
chr13-15389733-15390033	1	0.343934496
chr13-15426430-15426730	1	0.307450703
chr13-14630132-14630432	1	0.33486246
chr13-15207213-15207513	1	0.32720577
chr13-14612997-14613297	1	0.341281826
chr13-14618861-14619161	1	0.313395927
chr13-15741721-15742021	2	0.631223855
chr13-15513178-15513478	2	0.420404105
chr13-15710707-15711007	2	0.376911075
chr13-15463049-15463349	2	0.383527345
chr13-15588152-15588452	2	0.349446758
chr13-15424016-15424316	2	0.452762812
chr13-15677139-15677439	2	0.552651254
chr13-15623951-15624251	2	0.514136845
chr13-15710340-15710640	2	0.621366713
chr13-15580633-15580933	2	0.719525148
chr13-15533922-15534222	2	0.537739752
chr13-15427008-15427308	2	0.540226678
chr13-16475025-16475325	2	0.576949844
chr13-15481138-15481438	2	0.607033354
chr13-15466169-15466469	2	0.60568285
chr13-15022573-15022873	2	0.613069604
chr13-15514108-15514408	2	0.552401683
chr13-15468442-15468742	3	0.541840017
chr13-15179030-15179330	3	0.912154373
chr13-15209813-15210113	3	0.852258877
chr13-15588474-15588774	3	0.357232936
chr13-15194824-15195124	3	0.853054989
chr13-15421476-15421776	3	0.550011025
chr13-15395260-15395560	3	0.80372699
chr13-15510896-15511196	3	0.880873263
chr13-15464678-15464978	3	0.308939729
chr13-15444468-15444768	3	0.674052867
chr13-15390072-15390372	3	0.631164803
chr13-15419554-15419854	3	0.863418592
chr13-16490761-16491061	3	0.288823287
chr13-14613451-14613751	3	0.45370303
chr13-15207519-15207819	4	0.144901378
chr13-15465082-15465382	4	0.077779895
chr13-15464180-15464480	4	0.09492904
chr13-15759211-15759511	4	0.067843722
chr13-15463647-15463947	4	0.079570835

cluster	gene	Spearman correlation
2	Eda	0.042266485
2	Nfib	0.040278682
2	Sox5	0.039617735
2	Ntn1	0.037786414
2	Tspan18	0.036239888
2	CK137956	0.03569496
2	5830411N06Rik	0.035276878
2	Cdk6	0.034641351
2	Dst	0.034582122
2	2600006K01Rik	0.033750223
2	Acer3	0.033704349
2	Creb5	0.033491731
2	4930465M20Rik	0.033434961
2	Prickle2	0.033381351
2	Robo1	0.033158176
2	Gm13564	0.033005995
2	Cyba	0.032878417
2	Snx5	0.032779221
2	Slc24a3	0.032775267
2	Pdzd2	0.032624768
2	Timp3	0.03198593
2	Smoc2	0.031891344
2	Gm10282	0.031445877
2	Trp53inp1	0.031331721
2	Gm13264	0.031274237
2	2410018L13Rik	0.031214365
2	Tmprss12	0.031190583
2	Tpm1	0.030806486
2	Sox5os3	0.030777628
2	Setbp1	0.030734328
2	Prl	0.030697706
2	Gnaq	0.030660654
2	Cldn34d	0.030600585
2	Kansl3	0.030549218
2	Gm16181	0.030486215
2	Il27ra	0.030472972
2	Cald1	0.030211136
2	Rbms3	0.030139378
2	Mroh4	0.030092756
2	Mpdz	0.029942058
2	Frem2	0.029600178
2	Ppp1r14c	0.029549664
2	F7	0.029542893
2	Mroh8	0.029534321
2	Ggta1	0.029314496
2	Lgr5	0.028981137
2	Fbxw26	0.028805395
2	Gm20636	0.028805395
2	Pou2f1	0.028755609

2	Hmgn2	0.028707559
3	Prr5l	0.072471904
3	Lef1	0.072422817
3	Bmper	0.066449885
3	Foxp1	0.066369475
3	Dach1	0.061474814
3	Psd3	0.06088716
3	Slc7a8	0.059955691
3	Prkd1	0.059830963
3	Gli3	0.057903404
3	Kcnh1	0.057385234
3	Nrip1	0.055912478
3	Trps1	0.05536427
3	Gpnmb	0.054024058
3	Tiam2	0.053924321
3	Pmepa1	0.053358827
3	Frmd4b	0.053016746
3	Pxylp1	0.052959714
3	Ammecr1	0.05291089
3	Vav3	0.052743176
3	Lypd6b	0.052592012
3	Ptpre	0.052531675
3	Atxn1	0.052489152
3	Smad7	0.051088726
3	Kitl	0.050421806
3	Aldh1a3	0.050392746
3	Ces5a	0.049797881
3	Tle4	0.04974423
3	Lama3	0.049176157
3	Padi4	0.049116073
3	Runx2	0.048513462
3	Prdm1	0.047427994
3	Zfhx3	0.047315743
3	Celsr1	0.046814719
3	Pdzd8	0.045854041
3	Odc1	0.045778218
3	Syt7	0.045741185
3	Gtf2ird1	0.045456449
3	Trim29	0.045073002
3	Mid1	0.045050095
3	Mvb12b	0.044723436
3	Oaf	0.044696646
3	Col23a1	0.044369796
3	Ms4a13	0.044296084
3	Fhit	0.044229216
3	Grip1	0.044021675
3	Jag1	0.043999387
3	Stx1a	0.043335072
3	S100a9	0.043294034
3	Dct	0.042972219

3	Nrp1	0.042961503

Figure1

Click here to access/download;Figure;Figure1.png

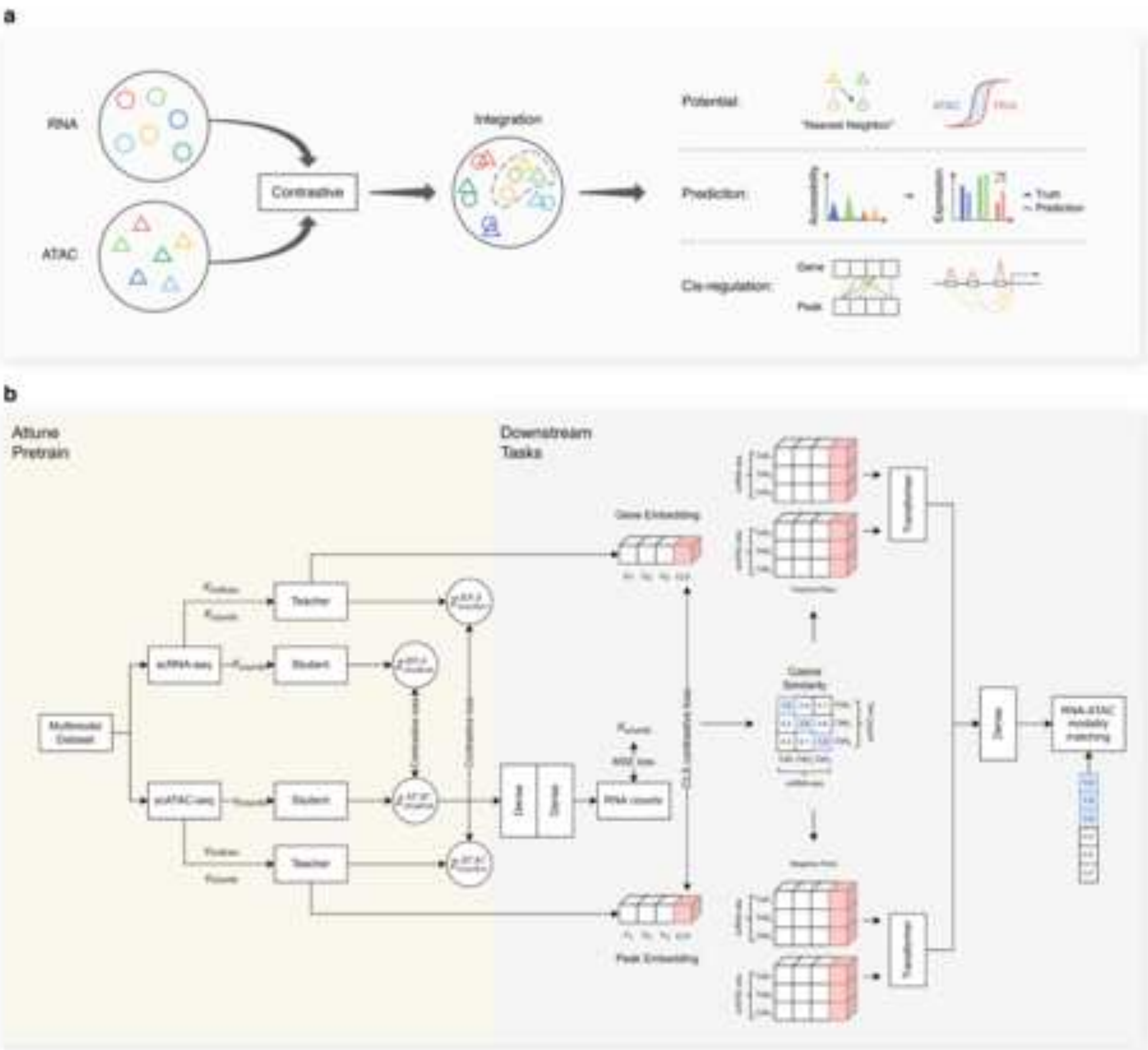

Figure2
Click here to access/download;Figure;Figure2.png

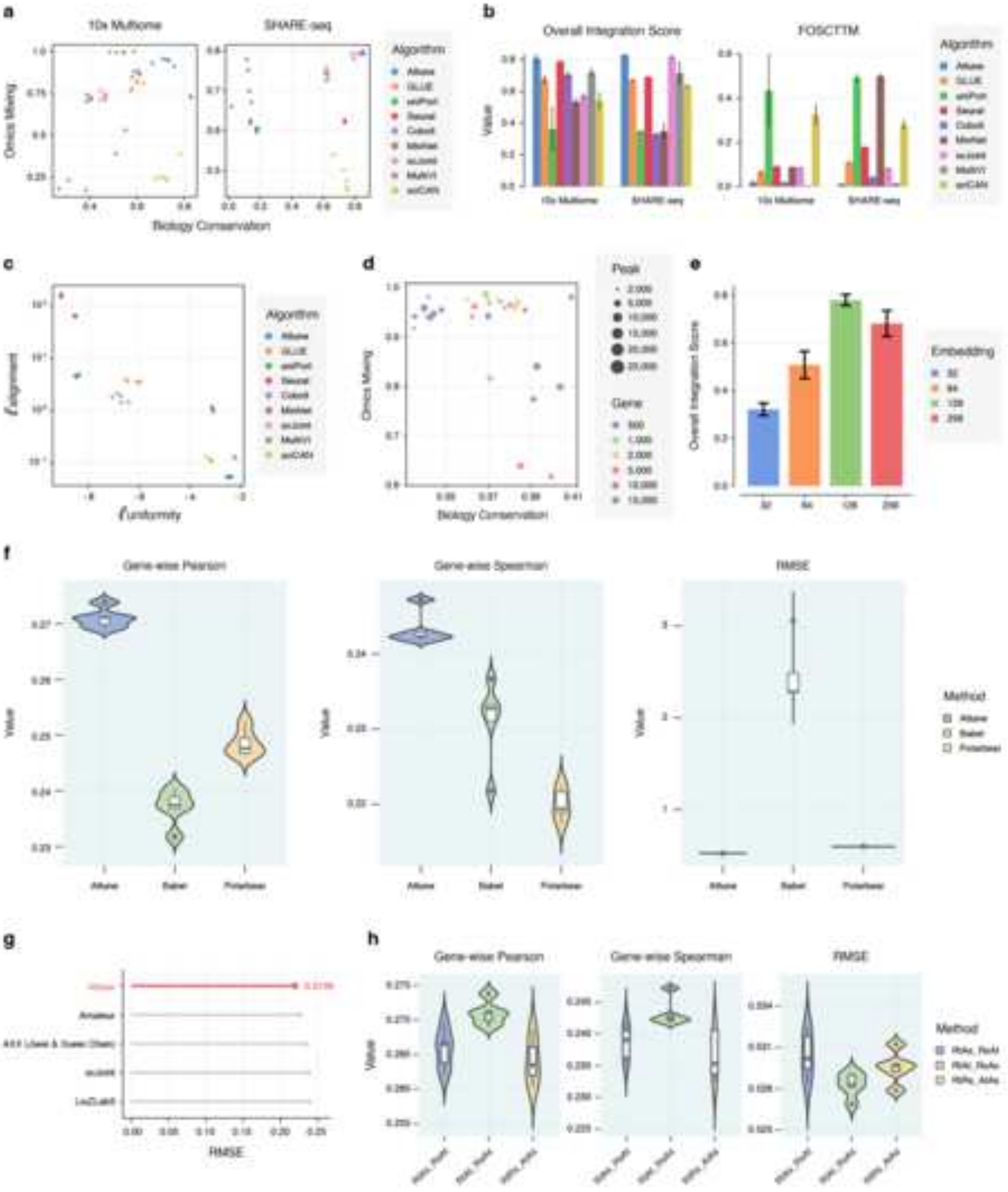

Figure3

Click here to access/download;Figure;Figure3.png

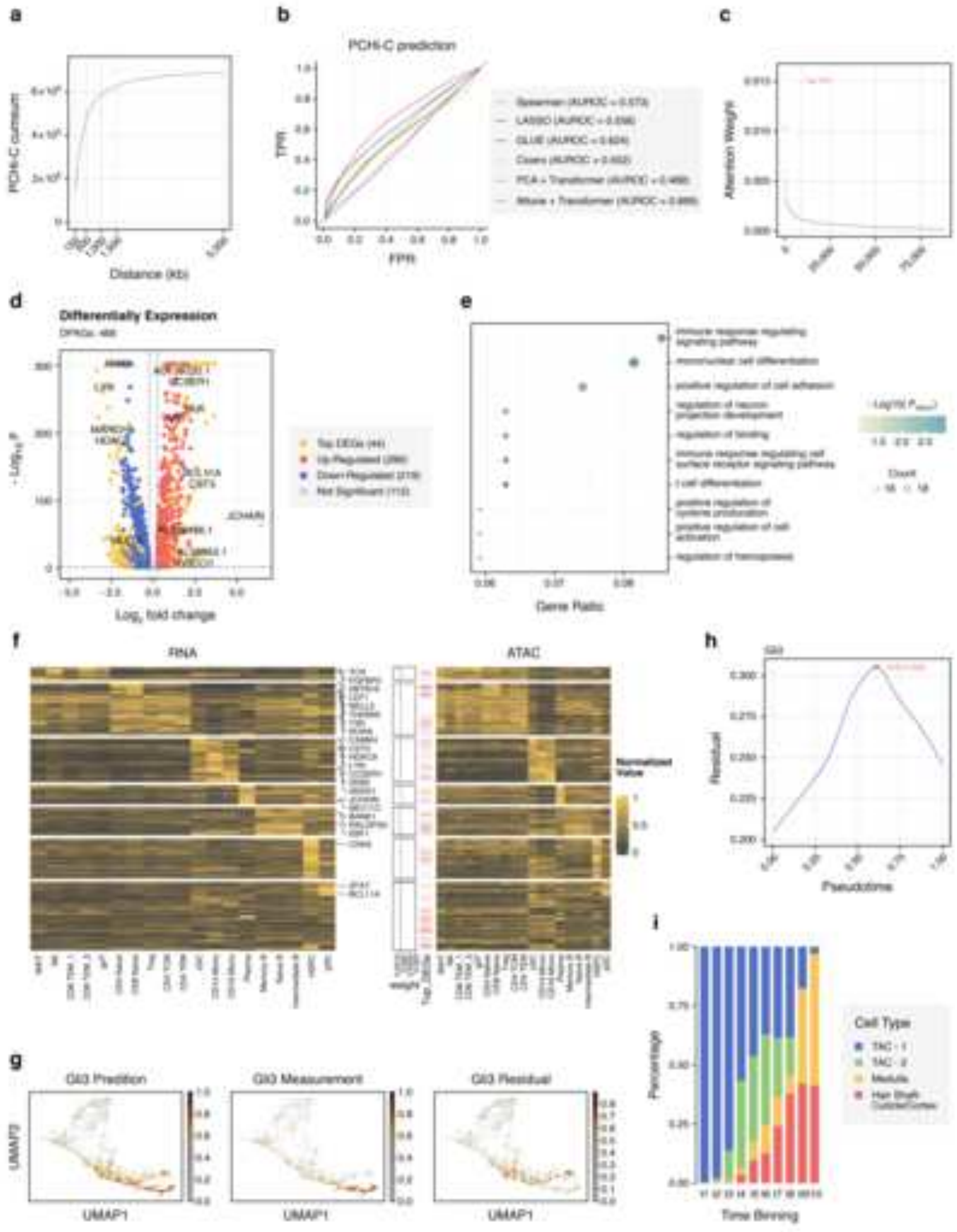

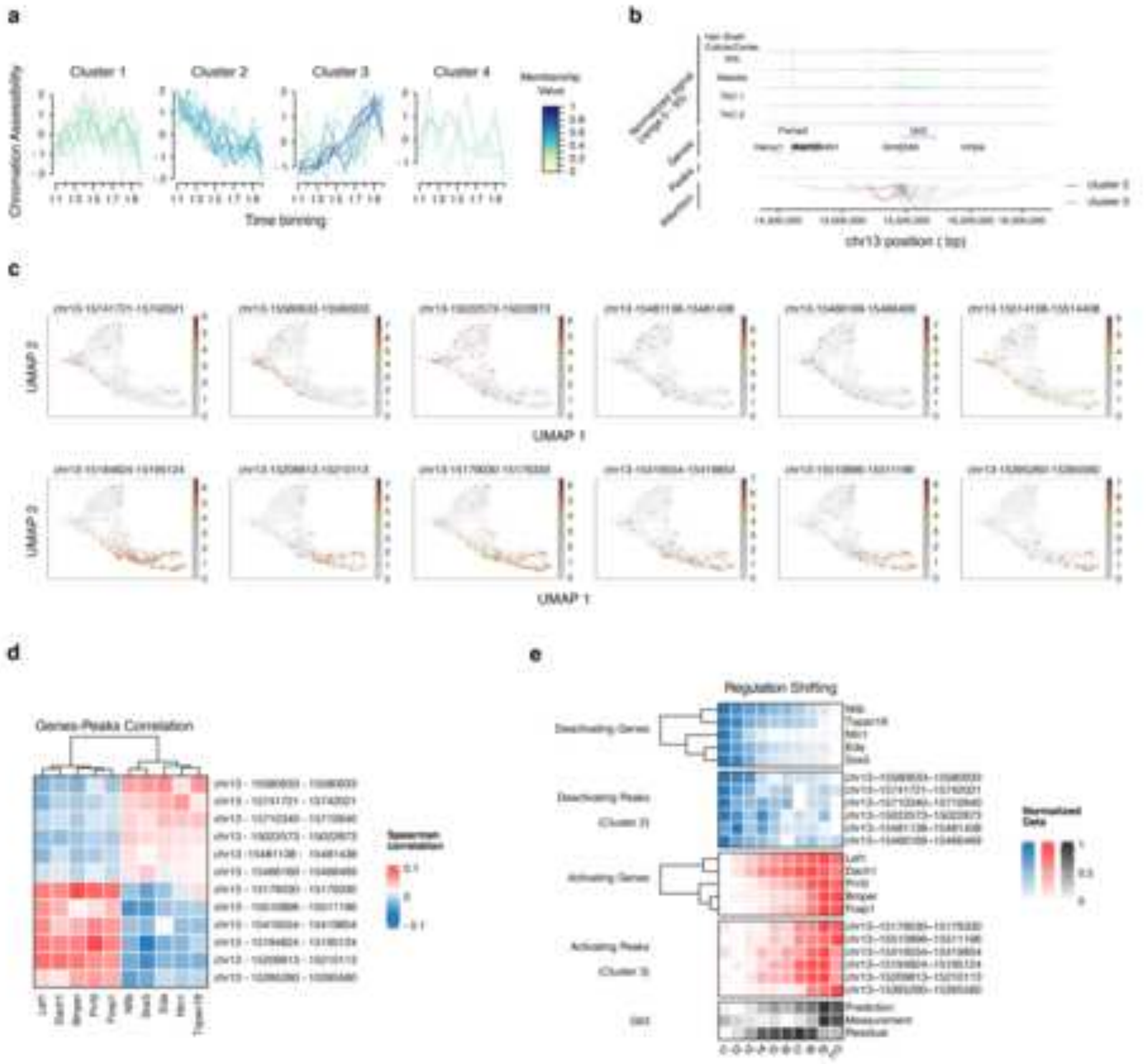
a
Cluster 1
Cluster 2
Cluster 3
Cluster 4
Chromatin Accessibility
Time binning
b
chr13 position (bp)
c
UMAP 2
UMAP 1
d
Genes-Peaks Correlation
e
Regulation Shifting
Deactivating Genes
Deactivating Peaks
Activating Genes
Activating Peaks

Figure5

Click here to access/download;Figure;Figure5_new.png

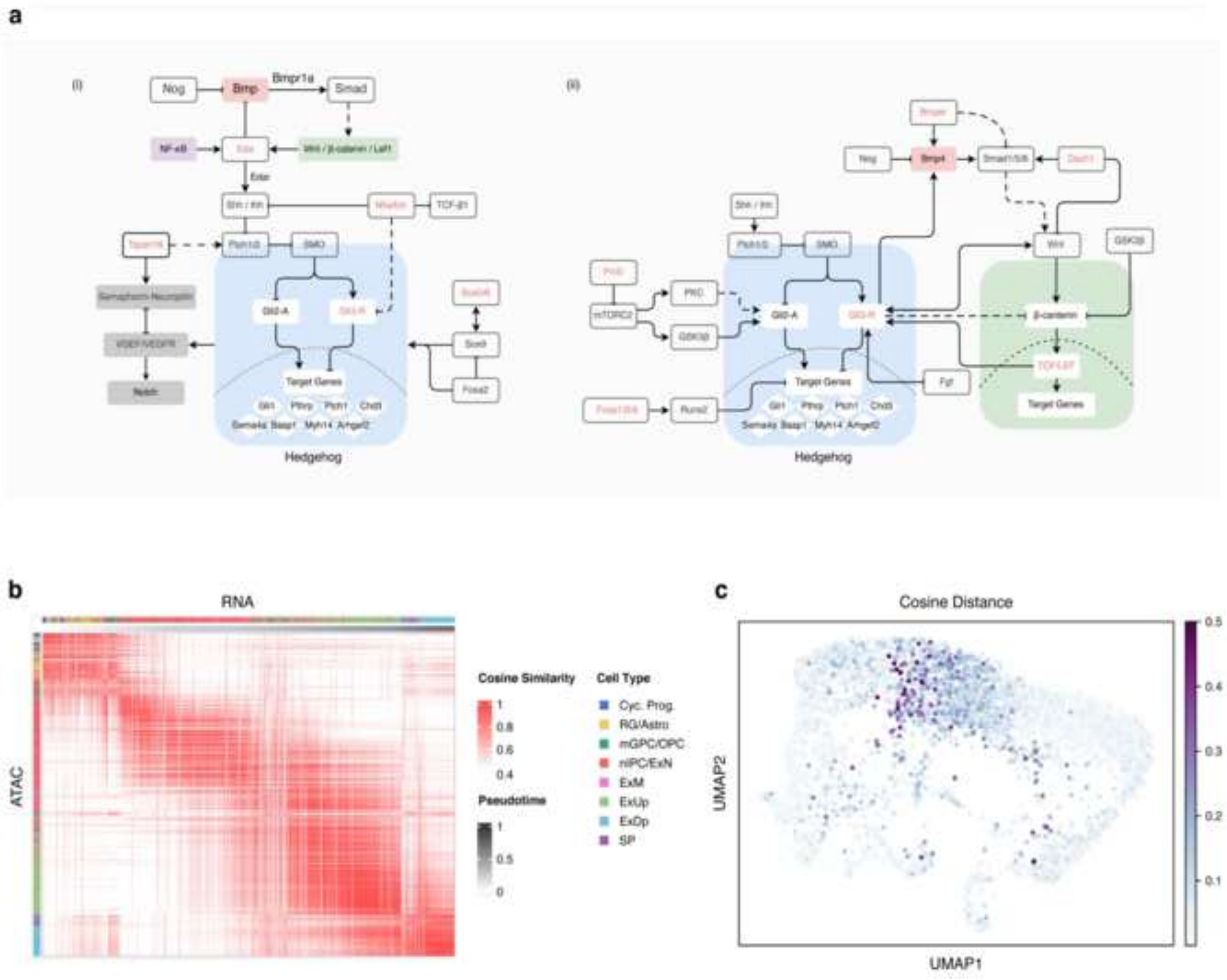

Supplementary Figure

Click here to access/download
Supplementary Material
Supplementary_File.pdf

Cover letter

Click here to access/download;Personal Cover;cover_letter_240522.docx

Dear Editor:

We are delighted to submit our manuscript, entitled "Cross-modal contrastive learning discovers chromatin potential regulating gene expression of single cell atlas" along with Supplementary Files containing 8 Supplementary Figures and 14 Supplementary Tables. We believe that our work has great potential as a candidate for publication in your esteemed journal.

Our manuscript introduces a cutting-edge computational framework called Attune, which utilizes cross-modal contrastive learning to advance the field of multi-modal single-cell data modeling and regulatory network discovery.

Our key innovations include as below,

1. Attune is a self-supervised learning framework that aligns paired gene expression and accessibility information through multi-view contrastive operations. It is fully data-driven, without requiring any prior knowledge or labels. Our framework's multi-view teacher-student configuration balances the advantages of complex and simple networks, capturing semantically rich features while maintaining generalizability. This configuration is well-suited for single-cell data analysis since each cell is unique, yet similarities are also shared between cells.

2. Attune's cell embeddings comprise information from different measurements, which can be flexibly adapted to various downstream tasks via pre-training and fine-tuning. Our framework outperforms competing methods in multiple tasks, such as data integration, cross-modal prediction, and cis-regulation discovery.

3. Moreover, Attune presents a novel strategy for inferring regulation in multi-modal single-cell data by implementing an attention mechanism. Current methods for regulation inference can be classified into four categories depending on the approach to deducing regulation: correlation (DORCs), co-expression (SCENIC+), distance (GLUE), and topic model (MIRA). The attention operation equips Attune with robust interpretability, effectively addressing the limitations of current deep learning methods in single-cell analysis. Leveraging cross-attention, Attune uncovers the cis-regulatory elements of genes and cell-type-specific factors that determine the cellular state.

4. We have also observed that certain cells' different modalities do not correspond, particularly cells in the differentiation process. Attune can capture this inconsistency and uncover chromatin potential along lineage priming. With the cross-attention mechanism, Attune unveils connections between peaks and genes and reconstructs a regulatory network that confirms the biological mechanism of chromatin potential. In the context of the mouse hair follicle dataset, Attune portrays a multi-pathway developmental landscape, beginning with the time delay of a dual-form transcription factor Gli3. Furthermore, by leveraging fine-tuned embeddings, Attune depicts the transition states between neonatal

and maturing neurons in the human cortex.

Our team specializes in integrating AI techniques into single-cell analysis, evidenced by our established publication record. We have recently contributed to Nature Machine Intelligence (doi.org/10.1038/s42256-022-00518-z) and iScience (10.1016/j.isci.2024.109635). These publications highlight our innovative approaches using contrastive learning for single-cell analysis and applying deep learning to investigate RNA velocity in single cells. We believe that our manuscript fits perfectly with the scope of your journal and adds significant value to the recent surge in using AI in multi-modal single-cell data discovery.

We eagerly look forward to your response and the opportunity to share our findings with your readership.

Best wishes,
Yang Meng
On behalf of all authors
MGI, BGI-Shenzhen

 Click here to access/download;Personal Cover;Response.docx

We sincerely appreciate the time and effort the reviewers have dedicated to reviewing our manuscript titled “”. We acknowledge the significance of addressing your concerns to ensure the clarity and comprehensibility of our work.

We are grateful for the valuable insights provided by Reviewer #1 in the feedback. And we will provide detailed explanations and clarifications for the raised comments, as we strive to address them effectively.

Reviewer#1 comments 1. As for model architecture presented in Figure 1b, the author included a one-way translation from ATAC counts to RNA counts. MSE loss was applied to minimize the reconstruction of translation from ATAC to RNA. What is the rationale behind such one-way translation? Why not translation from RNA counts to ATAC counts, or explicitly having bidirectional translation between these two modalities?

Response to Reviewer#1 comments 1:

Our approach was inspired by the multimodal single-cell data integration task presented at the NeurIPS 2021 competition, specifically Task 1: Modality Prediction [1]. This task focuses on predicting one modality from another, with performance evaluated using root mean squared error (RMSE). The biological rationale underlying this task is that genetic information flows from DNA to RNA to proteins. Chromatin accessibility (ATAC data) governs gene expression (RNA data), which in turn drives protein synthesis. By aligning with this biological hierarchy, our focus was to predict RNA (GEX) data from ATAC data, reflecting how chromatin accessibility regulates transcription.
In the context of modality prediction, previous works such as BABEL [2], MultiVI [3], and Polarbear [4] have demonstrated the feasibility of both RNA-to-ATAC and ATAC-to-RNA predictions. Building on this foundation, we chose to prioritize the ATAC-to-RNA direction for its biological relevance. However, to extend the model’s capability, we incorporated a reverse flow (RNA to ATAC) into Attune’s cross-modal prediction task (see Response Figure 1). We then benchmarked Attune against nine state-of-the-art algorithms, including LS_Lab, MultiVI, scVAEIT, LIGER, Seurat, BABEL, scMOG, scMoGNN, and CMAE, as described in a recent study on single-cell multi-omics prediction and integration [5].
To evaluate Attune, we utilized 11 single-cell RNA + ATAC datasets from a range of sequencing platforms, including SNARE-seq, SHARE-seq, ISSAAC-seq, 10x Multiome, and DOGMA-seq. We tested the model under two scenarios: intra-dataset (training and testing on the same dataset, see Response Figures 2A-2C) and inter-dataset (training and testing on different datasets, see Response Figures 2D-2F). Evaluation metrics included cell-cell PCC, peak-peak PCC, cell-cell CMD, peak-peak CMD, RMSE, AUROC, and Ranking Index (RI), providing a comprehensive assessment of prediction accuracy and generalizability. Specifically:

- **RMSE** quantifies the deviation between predicted and actual values.

- **CMD** measures differences in correlation matrices, indicating how well the model captures relationships between features.
- **PCC** assesses chromatin accessibility abundance correlation.
- **AUROC** evaluates the effectiveness of predicting chromatin accessibility.
- **RI** summarizes overall algorithm performance.
- A lower CMD and RMSE indicate better performance, while higher PCC and AUROC suggest more accurate predictions.

In the intra-dataset scenario, although Attune did not outperform LS_Lab or scVAEIT in all metrics, it achieved results that were consistently above or near the median across most benchmarks. Overall, Attune ranked in the second tier among the algorithms, comparable to MultiVI and slightly outperforming Seurat (see Response Figure 3). In the inter-dataset scenario, Attune demonstrated strong generalizability alongside LS_Lab. Both models achieved cell-cell PCC, peak-peak PCC, and AUROC values above the median, while their CMD and RMSE values were below the median, indicating robust cross-dataset performance. As a result, Attune ranked in the top tier, comparable to LS_Lab (see Response Figure 4). When considering performance across both intra- and inter-dataset scenarios (see Response Figure 5), Attune exhibited robustness and adaptability. While LS_Lab achieved the best overall performance, Attune was comparable to MultiVI and scVAEIT, highlighting its effectiveness and versatility.

The datasets and evaluation pipeline used in this study are consistent with the benchmarking article by Hu et al. [5]. The datasets were obtained from https://mailustceducn-my.sharepoint.com/:f:/g/personal/hyl2016_mail_ustc_edu_cn/EgYFP7tlTKBBuAhkdtrlOg4B1Eyo-_iBx1VKBWSK0r-9rA?e=gmhocx, and the evaluation pipeline was implemented as described in the benchmarking repository https://github.com/QuKunLab/MultiomeBenchmarking/blob/main/code/Prediction/RNA_ATAC/Matrics.py.

We hope this explanation clarifies the rationale for our design and the comprehensive steps we took to evaluate Attune.

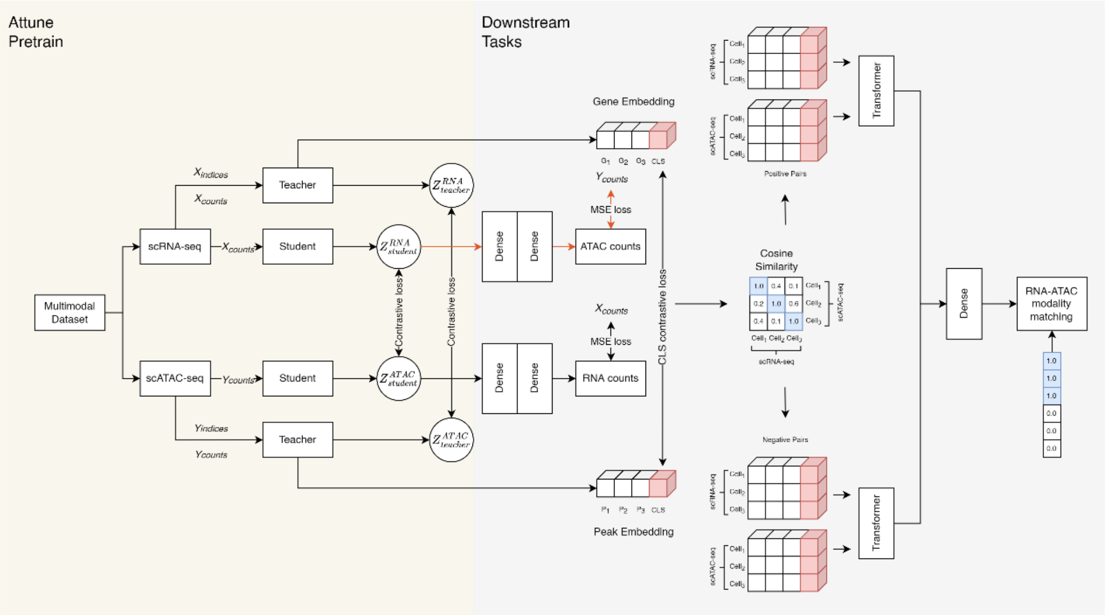

Response Figure 1. Overview of Attune model and transformer-based decoder architecture.

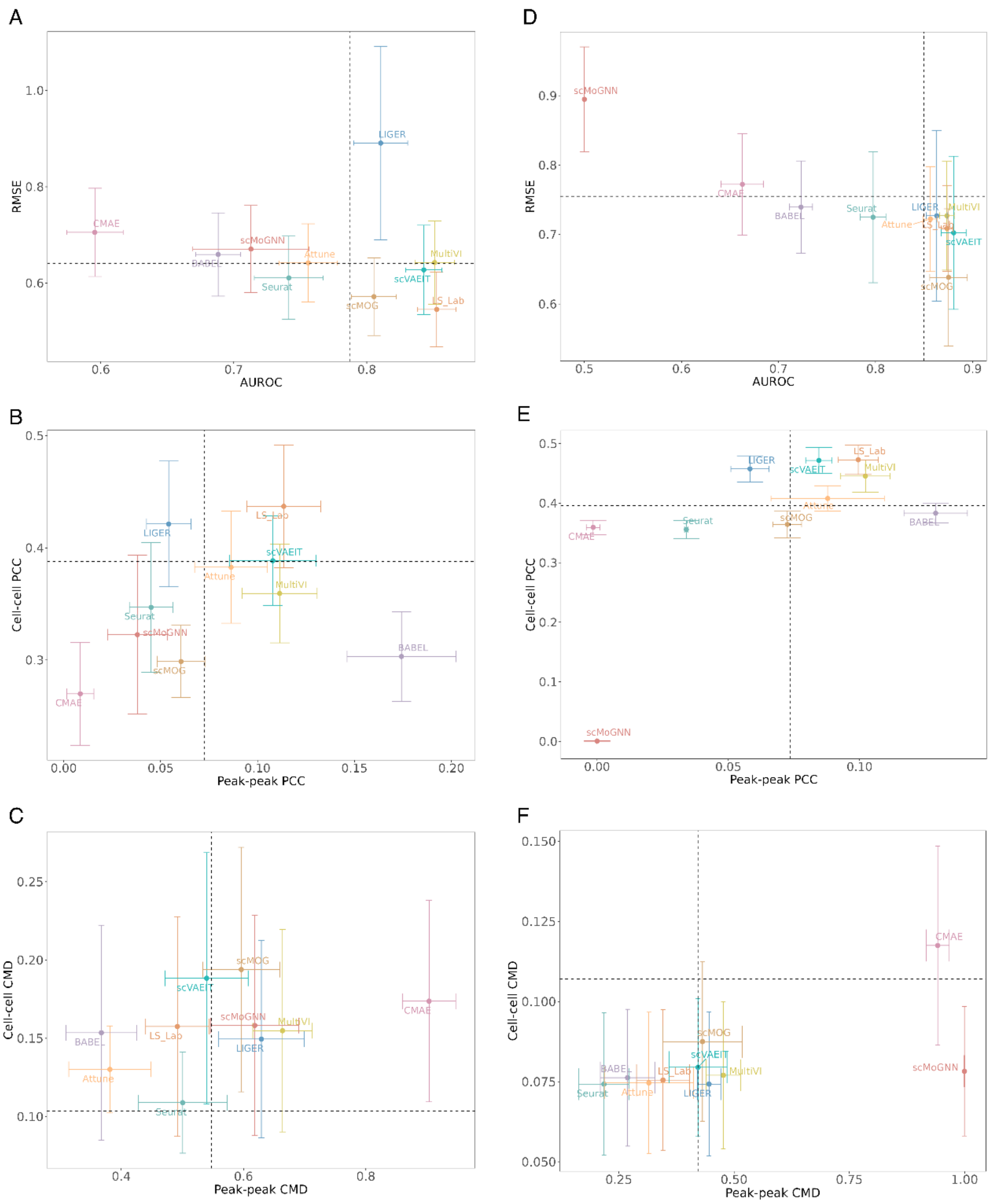

Response Figure 2. Performance Benchmarking of Cross-Modal Prediction

(A) Average RMSE versus average AUROC in the intra-dataset scenario. (B) Average cell-cell PCC versus average peak-peak PCC in the intra-dataset scenario. (C) Average cell-cell CMD versus average peak-peak CMD in the intra-dataset scenario. (D) Average RMSE versus average AUROC in the inter-dataset scenario. (E) Average cell-cell PCC versus average peak-peak PCC in the inter-dataset scenario. (F) Average cell-cell CMD versus average peak-peak CMD in the inter-dataset scenario. The dashed line represents the

median of all algorithm results. Error bars indicate the standard deviation across the 11 datasets. Data are presented as mean ± 0.5 times the standard deviation.

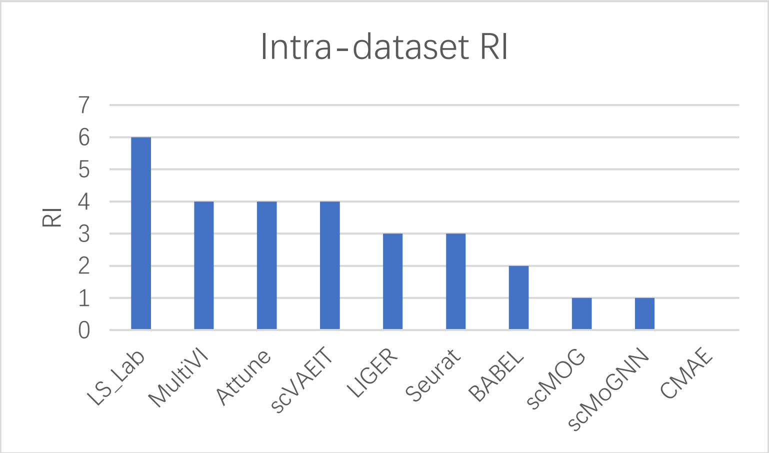

Response Figure 3. Ranking Index (RI) Values of 10 Algorithms in the Intra-Dataset Scenario

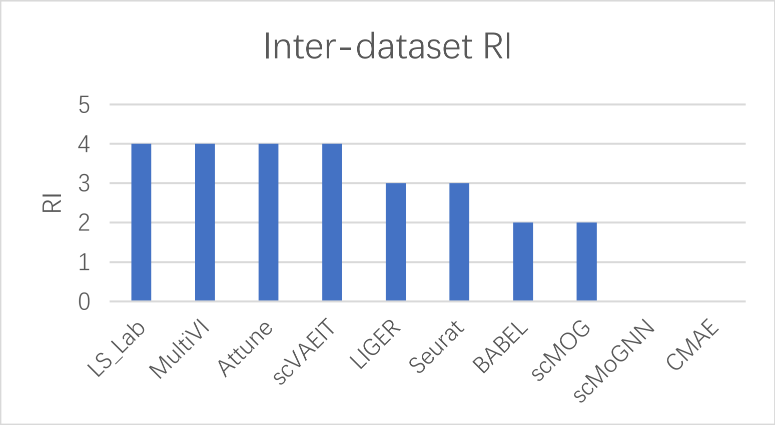

Response Figure 4. Ranking Index (RI) Values of 10 Algorithms in the Inter-Dataset Scenario

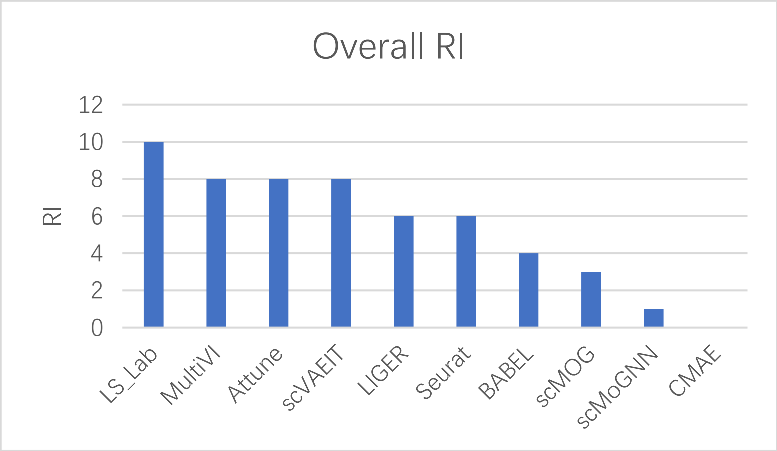

Response Figure 5. Overall Ranking Index (RI) Values of 10 Algorithms in Both Intra-Dataset and Inter-Dataset Scenarios

[1] Luecken, M.D., Burkhardt, D.B., Cannoodt, R., Lance, C., Agrawal, A., Aliee, H., Chen, A.T., Deconinck, L., Detweiler, A.M., and Granados, A.A. A sandbox for prediction and integration of dna, rna, and proteins in single cells. 2021.
[2] Wu K E, Yost K E, Chang H Y, et al. BABEL enables cross-modality translation between multiomic profiles at single-cell resolution[J]. Proceedings of the National Academy of Sciences, 2021, 118(15): e2023070118.
[3] Ashuach T, Gabitto M I, Koodli R V, et al. MultiVI: deep generative model for the integration of multimodal data[J]. Nature Methods, 2023, 20(8): 1222-1231.
[4] Zhang R, Meng-Papaxanthos L, Vert J, et al. Multimodal single-cell translation and alignment with semi-supervised learning[J]. Journal of Computational Biology, 2022, 29(11): 1198-1212.
[5] Hu Y, Wan S, Luo Y, et al. Benchmarking algorithms for single-cell multi-omics prediction and integration[J]. Nature Methods, 2024: 1-13.

Reviewer#1 comments 2. The gene-peak cross-attention seems to be a very big computation, not to mention the computation needed for the peak-peak self-attention (It is not clear to me if peak-peak self-attention is included in the model. If not, why peak-peak attention is unnecessary?). It is unclear what kind of computational power is enough to do the transformer fine-tuning. Meanwhile, it is also not clear why the author didn't combine transformer fine-tuning with pretraining as one end-to-end training? What are the benefits of dividing the model into two parts?

Response to Reviewer#1 comments 2:

Thank you for your insightful comments. We greatly appreciate the opportunity to further clarify the design and computational approach of our model.
Our methodology consists of two main components: pre-training the Attune model and performing downstream tasks. During the pre-training phase, the expression matrices of both ATAC-seq and RNA-seq data are input into the pre-training component. This phase employs unsupervised cross-modal contrastive learning, which is critical for setting up the model for subsequent tasks. The goal is to establish shared high-dimensional representations for cells across the two modalities (ATAC and RNA).
For tasks involving gene-peak interactions, the model generates embedding matrices for genes and peaks (with dimensions $N \times G \times d$ for genes and $N \times P \times d$ for peaks, where N represents the number of cells, G represents the number of genes, P represents the number of peaks, and d is the embedding dimension). These matrices are then passed through a Transformer model that uses self-attention and cross-attention mechanisms to derive a global cross-attention weight matrix ($G \times P$), which captures the gene-peak relationships. The self-attention mechanism operates on the gene embedding matrices ($N \times G \times d$) to model interactions between genes. The cross-attention mechanism operates between the peak embedding matrices ($N \times P \times d$) and the output of the gene self-attention mechanism ($N \times G \times d$) to capture the interaction between genes and peaks.
(1) Why not perform self-attention on peaks?

Due to hardware limitations (we are using a Quadro RTX 6000 with 24GB of memory), we are unable to perform self-attention on the peak embedding matrices. The number of peaks in typical datasets ranges from tens of thousands to hundreds of thousands, and the corresponding embedding matrices are typically very sparse. Performing self-attention on such sparse matrices would be computationally expensive and inefficient. Our testing has shown that performing self-attention on genes (with approximately 2,000 genes) and cross-attention between genes and peaks (with around 28,708 peaks) consumes approximately 24GB of memory on an RTX 6000 (batch size = 2) and 76GB on an A100 (batch size = 12). These memory requirements indicate that, with our current hardware setup, performing self-attention on peaks is infeasible due to their large number and sparsity.

In our cross-attention mechanism, we use the gene embedding matrices, after self-attention, as the Query and directly use the peak embedding matrices as the Key and Value. When investigating regulatory interactions between genes, we aim to identify which peaks regulate each gene. It is therefore reasonable to use the gene embeddings as the Query, as they already capture associations between genes. Each gene in the Query computes the similarity (via vector dot product) with each peak in the Key. If a gene is highly correlated with a peak, the corresponding peak in the Value will be selected. This peak is then weighted by the similarity score, and the gene embedding matrix is updated accordingly, based on the relationship between the gene and the peak.

(2) Why not combine pre-training and fine-tuning into a single end-to-end process?

The decision to separate pre-training and fine-tuning is a deliberate design choice to address computational constraints while ensuring effective model training. By pre-training Attune using cross-modal contrastive learning, we ensure that the gene and peak representations share a high-dimensional latent space. During the downstream task, we freeze the weights of the Attune model and only fine-tune the Transformer. This separation prevents the contrastive learning process from disrupting the learned gene-peak associations during fine-tuning, thus ensuring stability in the shared space and improving the accuracy of downstream predictions. If we were to combine pre-training and fine-tuning into a single end-to-end training process, the backpropagation through both Attune and the Transformer might cause the learned gene-peak associations to shift, leading to potential instability. The contrastive learning process in Attune would update the distances between cell representations in the shared latent space, which could inadvertently affect the pre-established gene-peak associations. By separating these stages, we maintain a more stable learning process for the downstream task.

In summary, the decision to exclude self-attention on peaks and to split the pre-training and fine-tuning stages was driven by both computational constraints and the need for a stable and efficient learning process. We hope this explanation clarifies the rationale behind our model architecture and design choices.

Reviewer#1 comments 3. It is not clear how benchmarking was done. Both 10X multiome and SHARE-seq are joint profiling data. It is necessary for the author to provide more details about how other methods were run under author's hands, especially how the author handled data preprocessing for each method. Meanwhile,

each cell has profiles of two modalities. Attune clearly takes advantage of knowing such correspondence. However, other methods primarily use shared features for integration, which will not take a full advantage of joint profiling. Seurat V3 was included in benchmarking. This version of Seurat also uses shared features. The author will need to consider adding Seurat V5 in benchmarking, which uses information of joint profiling for integration. Another existing contrastive learning SMILE was also proposed to integrate RNA-seq and ATAC-seq with joint-profiling data. Meanwhile, the author previously developed another contrastive learning integration method Concerto. It should also be included in benchmarking. The author may need to include more clear justifications on how Attune is improved from their previous work Concerto.

Response to Reviewer#1 comments 3:

Thank you for your insightful comments regarding the benchmarking methodology and the comparison with existing methods. We agree that it is important to clarify the distinction between multimodal integration and cross-modal alignment, as these are fundamentally different tasks with distinct objectives.

(1) Multimodal Integration: In our approach, Attune is designed for multimodal integration, where the goal is to combine data from paired modalities into a shared feature space. The model takes joint profiling data (where each cell contains profiles of both RNA and ATAC modalities) and learns a joint embedding that represents both modalities together. This approach aligns with the concept of joint embedding as outlined in the NIPS 2021 competition [1], which focuses on integrating features from different modalities into a unified representation. Attune leverages the correspondence between modalities within paired cells to optimize the integration, ensuring that both modalities are represented in a coherent and shared space.

(2) Cross-modal Alignment: In contrast, cross-modal alignment methods aim to establish correspondences between unpaired cells from different modalities. Historically, most single-cell methods were designed to analyze one modality at a time, but recent advances have introduced methods for measuring multiple modalities within the same cells. Methods such as GLUE, uniPort, Seurat V3, Cobolt, MinNet, scJoint, MultiVI, and sciCAN focus on aligning unpaired cells by mapping data from different modalities into a shared space using similarity metrics. These methods can also be applied to joint profiling data, but they do not fully exploit the pairing information. For example, scJoint is designed for unpaired data, but it can still be used with paired data, treating the RNA and ATAC components as separate datasets [2]. Similarly, GLUE has been benchmarked against unpaired methods like MMD-MA, LIGER, and Seurat V3 using paired data such as 10X Multiome and SHARE-seq [3].

Data Processing. In our benchmarking experiments, we followed the standard preprocessing procedures for multimodal data integration. Although the data is joint profiling, we treated the RNA and ATAC parts as separate datasets for the purpose of comparison with other algorithms. For scRNA-seq data, we removed genes expressed in fewer than 5% of cells and normalized the counts to 10,000 per cell using SCANPY.

Additionally, sex chromosome genes were excluded, and the top 2000 highly variable genes (HVGs) were selected. For scATAC-seq data, we filtered out peaks detected in fewer than 5% of cells and removed sex chromosome peaks. These preprocessing steps ensured that the datasets were comparable while balancing computational efficiency and model performance. Methods like MultiVI and GLUE do not require the same number of features between modalities and can directly handle RNA and ATAC data. However, methods such as scJoint and sciCAN require common features between the modalities, so we first identified overlapping genes between RNA and ATAC datasets before applying the algorithms. After training, we computed integration metrics such as neighbor consistency, Seurat alignment score, and Fraction of Samples Closer Than the True Match (FOSCTTM).

Benchmarking with Multimodal Integration Methods. The key advantage of Attune lies in its ability to explicitly use the pairing information between modalities. This allows the model to treat the two modalities of the same cell as positive pairs, which results in a more precise alignment. Through contrastive learning, the model brings RNA and ATAC data closer in a high-dimensional space, yielding better performance in metrics like FOSCTTM compared to methods that rely solely on shared features. Unlike other methods that aim to create a single unified representation, Attune aligns RNA and ATAC data in a shared feature space while maintaining the identity of each modality, allowing for more accurate comparisons and analyses of the interactions between the modalities. Concerto [4] supports multimodal integration. It is a simple element-wise summation of the output of the teacher network or student network for each modality (the embedding of cell in each modality). The contrastive loss (NT-Xent loss [5]) is calculated on the summed cell embeddings (from teacher network and student network, respectively). Concert can generate unified cell embeddings. In the case of two modalities (RNA and ATAC), we illustrate the corresponding operation according to equation (1) and (2) and (3), where the term $z_{teacher}^{RNA} \in \mathbb{R}^d$ denotes cell embedding of RNA output by teacher network and $z_{teacher}^{ATAC} \in \mathbb{R}^d$ denotes cell embedding of ATAC output by teacher network and $z_{student}^{RNA} \in \mathbb{R}^d$ denotes cell embedding of RNA output by student network and $z_{student}^{ATAC} \in \mathbb{R}^d$ denotes cell embedding of ATAC output by student network. Add denotes add along the dimension of embedding and Contrastive denotes NT-Xent loss and d denotes the dimension of cell embeddings.

$$z_{teacher}^{multi} = Add\left(z_{teacher}^{RNA}, z_{teacher}^{ATAC}\right) \qquad z_{teacher}^{multi} \in \mathbb{R}^d \tag{1}$$

$$z_{student}^{multi} = Add\left(z_{student}^{RNA}, z_{student}^{ATAC}\right) \qquad z_{student}^{multi} \in \mathbb{R}^d \tag{2}$$

$$\mathcal{L}_{contrastive} = Contrastive\left(z_{teacher}^{multi}, z_{student}^{multi}\right) \tag{3}$$

The improvement of Attune over Concerto is that it does not need to sum the cell embeddings of the two modalities before calculating the contrastive loss, but directly calculates the contrastive loss for the cell embeddings of each modality (from the teacher network and the student network respectively). The above calculation process in shown in equation (4) and (5) and (6).

$$\mathcal{L}_{teacher} = Contrastive(z_{teacher}^{RNA}, z_{teacher}^{ATAC}) \quad (4)$$

$$\mathcal{L}_{student} = Contrastive(z_{student}^{RNA}, z_{student}^{ATAC}) \quad (5)$$

$$\mathcal{L}_{contrastive} = \frac{\mathcal{L}_{teacher} + \mathcal{L}_{student}}{2} \quad (6)$$

When using UMAP to visualize cell embeddings, Concerto performs UMAP on $z_{teacher}^{multi}$ to obtain unified cell embeddings （N×d） that combines the two modalities (RNA and ATAC), while Attune concatenates $z_{teacher}^{RNA}$ and $z_{teacher}^{ATAC}$ to get joint cell embeddings （2N×d）, which maps the cell embeddings of two modalities onto the same two-dimensional space. N represents the number of cells.

In response to the reviewer's suggestion, we have included SMILE [6], Seurat V5 [7], and Concerto in the benchmarking process. These methods use joint profiling data for integration and are compared with Attune using several metrics: mean average precision (MAP), cell type adjusted silhouette width (ASW), neighbor consistency (NC), Seurat alignment score (SAS), batch adjusted silhouette width (Batch ASW), graph connectivity (GC), biology conservation, omics mixing, overall integration score, and FOSCTTM (see Methods for details). Since Seurat V5 maps scATAC-seq datasets onto scRNA-seq datasets when integrating different modalities, it only outputs cell representations of single modality and Concerto generates unified cell embeddings. Therefore, it is impossible to calculate the metrics that require the cell representation of each modality, such as Batch ASW, NC, SAS and FOSCTTM.

In the 10X Multiome dataset (Response Table 1), SMILE outperforms Attune in GC, but Attune shows superior results in most other metrics. Seurat V5 excels in MAP and cell type ASW but is outperformed by Attune in GC. Concerto performs similarly to Attune in MAP but lags behind in terms of robustness in cell type ASW and GC. UMAP visualizations for SMILE, Seurat V5, and Concerto are shown in Response Figures 6-9.

In the SHARE-seq dataset (Response Table 2), SMILE surpasses Attune in Batch ASW and GC but falls short in MAP and cell type ASW. Therefore, it is close to Attune in Omics mixing, but inferior to Attune in Biology conservation. Seurat V5 and Concerto also perform worse than Attune in MAP, cell type ASW, and GC. The UMAP visualizations for SMILE, Seurat V5, and Concerto are shown in Response Figures 10-13. These results were repeated five times with different random seeds to ensure robustness.

Response Table 1. Metrics for evaluating integration performance in 10X Multiome dataset

fold	algorithm	map	Batch ASW	Cell type ASW	GC	NC	SAS	FOSCTTM	Biology conservation	Omics mixing	Overall score
1	SMILE	0.695	0.858	0.515	0.989	0.366	0.589	0.008	0.525	0.812	0.640
2	SMILE	0.692	0.849	0.520	0.987	0.367	0.581	0.007	0.526	0.806	0.638
3	SMILE	0.688	0.855	0.510	0.980	0.361	0.582	0.008	0.520	0.806	0.634

4	SMILE	0.689	0.850	0.513	0.985	0.365	0.580	0.007	0.522	0.805	0.635
5	SMILE	0.691	0.852	0.515	0.981	0.365	0.584	0.008	0.524	0.806	0.636
1	Attune	0.716	0.951	0.540	0.934	0.211	0.915	0.018	0.664	0.929	0.770
2	Attune	0.729	0.954	0.545	0.937	0.212	0.975	0.023	0.708	0.955	0.807
3	Attune	0.740	0.943	0.552	0.928	0.213	0.892	0.012	0.756	0.911	0.818
4	Attune	0.730	0.953	0.545	0.935	0.217	0.966	0.025	0.725	0.950	0.815
5	Attune	0.733	0.958	0.545	0.935	0.218	0.947	0.011	0.734	0.947	0.819
1	Seurat V5	0.811		0.550	0.854						
2	Seurat V5	0.809		0.548	0.850						
3	Seurat V5	0.810		0.549	0.852						
4	Seurat V5	0.810		0.550	0.853						
5	Seurat V5	0.809		0.549	0.851						
1	Concerto	0.742		0.522	0.905						
2	Concerto	0.734		0.512	0.893						
3	Concerto	0.731		0.511	0.892						
4	Concerto	0.725		0.510	0.883						
5	Concerto	0.721		0.507	0.881						

Response Table 2. Metrics for evaluating integration performance in 10X SHARE-seq dataset

fold	algorithm	map	Batch ASW	Cell type ASW	GC	NC	SAS	FOSCTTM	Biology conservation	Omics mixing	Overall score
1	SMILE	0.630	0.915	0.510	0.881	0.389	0.562	0.015	0.510	0.786	0.620
2	SMILE	0.631	0.920	0.511	0.880	0.388	0.560	0.014	0.51	0.787	0.621
3	SMILE	0.629	0.916	0.509	0.882	0.390	0.561	0.015	0.509	0.786	0.620
4	SMILE	0.630	0.915	0.512	0.881	0.390	0.563	0.014	0.511	0.786	0.621
5	SMILE	0.629	0.914	0.509	0.879	0.388	0.560	0.015	0.509	0.784	0.619
1	Attune	0.701	0.813	0.540	0.851	0.107	0.810	0.012	0.847	0.797	0.827
2	Attune	0.702	0.813	0.540	0.858	0.107	0.809	0.011	0.849	0.799	0.829
3	Attune	0.703	0.812	0.541	0.853	0.107	0.807	0.011	0.851	0.795	0.828
4	Attune	0.704	0.811	0.541	0.853	0.107	0.806	0.010	0.853	0.793	0.829
5	Attune	0.705	0.810	0.542	0.846	0.107	0.813	0.010	0.855	0.792	0.830
1	Seurat V5	0.602		0.483	0.570						
2	Seurat V5	0.602		0.484	0.572						
3	Seurat V5	0.603		0.482	0.572						
4	Seurat V5	0.601		0.481	0.571						
5	Seurat V5	0.602		0.482	0.573						
1	Concerto	0.467		0.497	0.703						
2	Concerto	0.447		0.494	0.684						
3	Concerto	0.483		0.499	0.714						
4	Concerto	0.497		0.502	0.733						

5	Concerto	0.437		0.491	0.660						

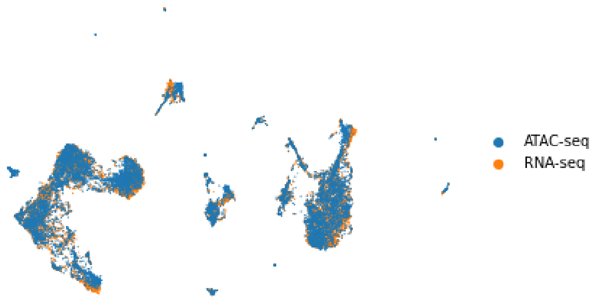

Response Figure 6. UMAP visualization of the cell embeddings of SMILE in the 10X Multiome dataset (colored by modalities).

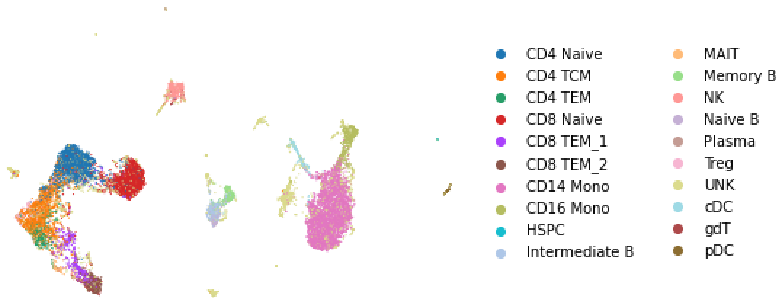

Response Figure 7. UMAP visualization of the cell embeddings of SMILE in the 10X Multiome dataset (colored by cell types).

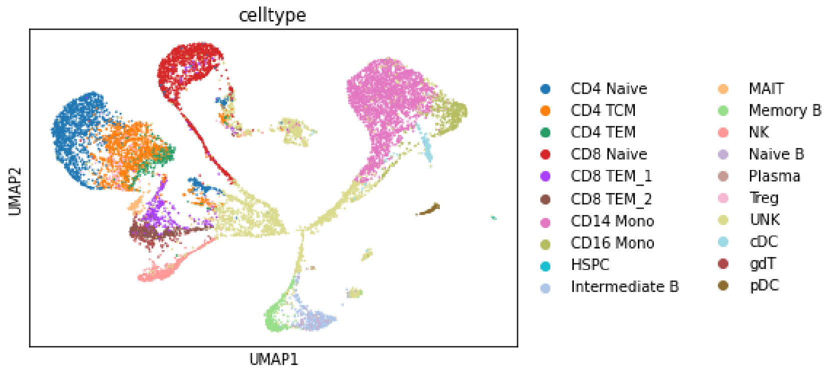

Response Figure 8. UMAP visualization of the cell embeddings of Seurat V5 in the 10X Multiome dataset (colored by cell types).

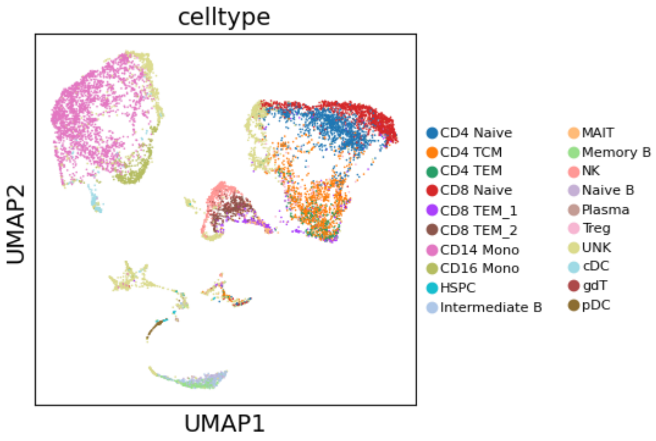

Response Figure 9. UMAP visualization of the cell embeddings of Concerto in the 10X Multiome dataset (colored by cell types).

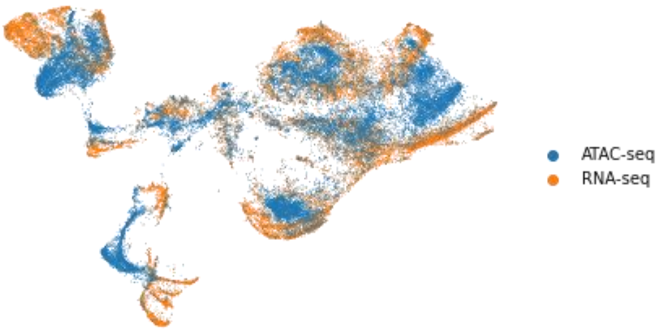

Response Figure 10. UMAP visualization of the cell embeddings of SMILE in the SHARE-seq dataset (colored by modalities).

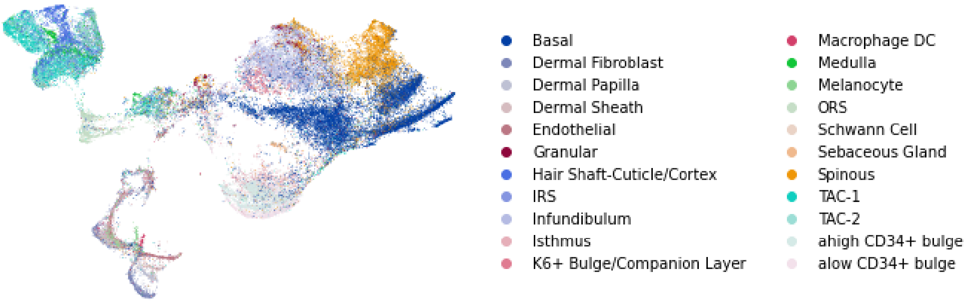

Response Figure 11. UMAP visualization of the cell embeddings of SMILE in the SHARE-seq dataset (colored by cell types).

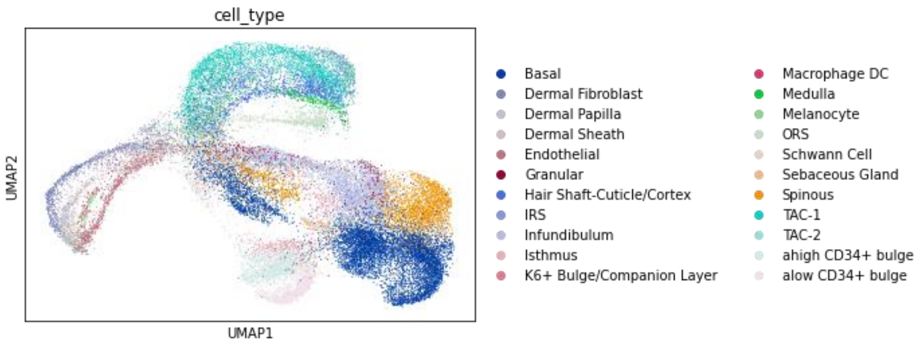

Response Figure 12. UMAP visualization of the cell embeddings of Seurat V5 in the SHARE-seq dataset (colored by cell types).

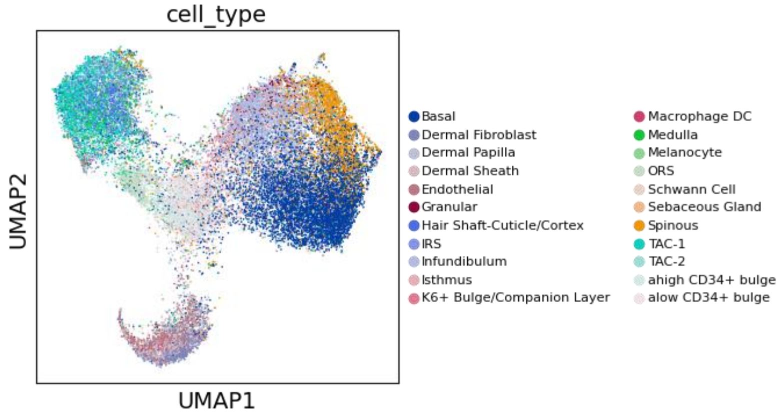

Response Figure 13. UMAP visualization of the cell embeddings of Concerto in the SHARE-seq dataset (colored by cell types).

[1] Luecken, M.D., Burkhardt, D.B., Cannoodt, R., Lance, C., Agrawal, A., Aliee, H., Chen, A.T., Deconinck, L., Detweiler, A.M., and Granados, A.A. A sandbox for prediction and integration of dna, rna, and proteins in single cells. 2021.

[2] Lin Y, Wu T Y, Wan S, et al. scJoint integrates atlas-scale single-cell RNA-seq and ATAC-seq data with transfer learning[J]. Nature biotechnology, 2022, 40(5): 703-710.

[3] Cao Z J, Gao G. Multi-omics single-cell data integration and regulatory inference with graph-linked embedding[J]. Nature Biotechnology, 2022, 40(10): 1458-1466.

[4] Yang M, Yang Y, Xie C, et al. Contrastive learning enables rapid mapping to multimodal single-cell atlas of multimillion scale[J]. Nature Machine Intelligence, 2022, 4(8): 696-709.

[5] Chen T, Kornblith S, Norouzi M, et al. A simple framework for contrastive learning of visual representations[C]//International conference on machine learning. PMLR, 2020: 1597-1607.

[6] Xu Y, Das P, McCord R P. SMILE: mutual information learning for integration of single-cell omics data[J]. Bioinformatics, 2022, 38(2): 476-486.

[7] Hao Y, Stuart T, Kowalski M H, et al. Dictionary learning for integrative, multimodal and scalable single-cell analysis[J]. Nature biotechnology, 2024, 42(2): 293-304.

Reviewer#1 comments 4. The author included a PCA + Transformer as one of

comparisons in the task of regulatory prediction. It is questionable if PCA is a proper way to learn the peak embedding. Second, only the top 10 PCs don't justify if all major variations are captured in 10 PCs. I am interested in knowing if NMF-based approach would perform much better than PCA. For example, the author can combine the cisTopic and Transformer.

Response to Reviewer#1 comments 4:

Thank you for your insightful feedback and thoughtful suggestions. We greatly appreciate your comments regarding the use of PCA for learning peak embeddings and the potential advantages of alternative methods such as NMF.

In response to your concern, we performed additional experiments to evaluate the performance of NMF-based methods. Specifically, we compared PCA with 10 components to NMF with both 10 and 20 components. However, as shown in Response Figure 14, the results did not demonstrate a significant improvement over the PCA approach. This suggests that, while NMF may have some potential advantages, the performance gain over PCA was not particularly substantial in our study. We acknowledge that further exploration, including experimentation with different configurations and larger datasets, could provide a more comprehensive assessment of NMF's capabilities in this context.

Regarding cisTopic, we recognize that it is primarily designed for scATAC-seq data and may not be directly applicable to scRNA-seq. In our study, we opted to use PCA as the dimensionality reduction technique for both scRNA-seq and scATAC-seq data to maintain consistency across the two modalities. However, we are uncertain whether the reviewer would prefer us to apply PCA specifically to scRNA-seq and use cisTopic for the scATAC-seq data. If this alternative approach is preferred, we are happy to explore it further and present the corresponding results.

We hope this clarifies our approach and addresses your concerns. Should you require additional information or further analysis, we are more than willing to provide it. Thank

you once again for your valuable input.

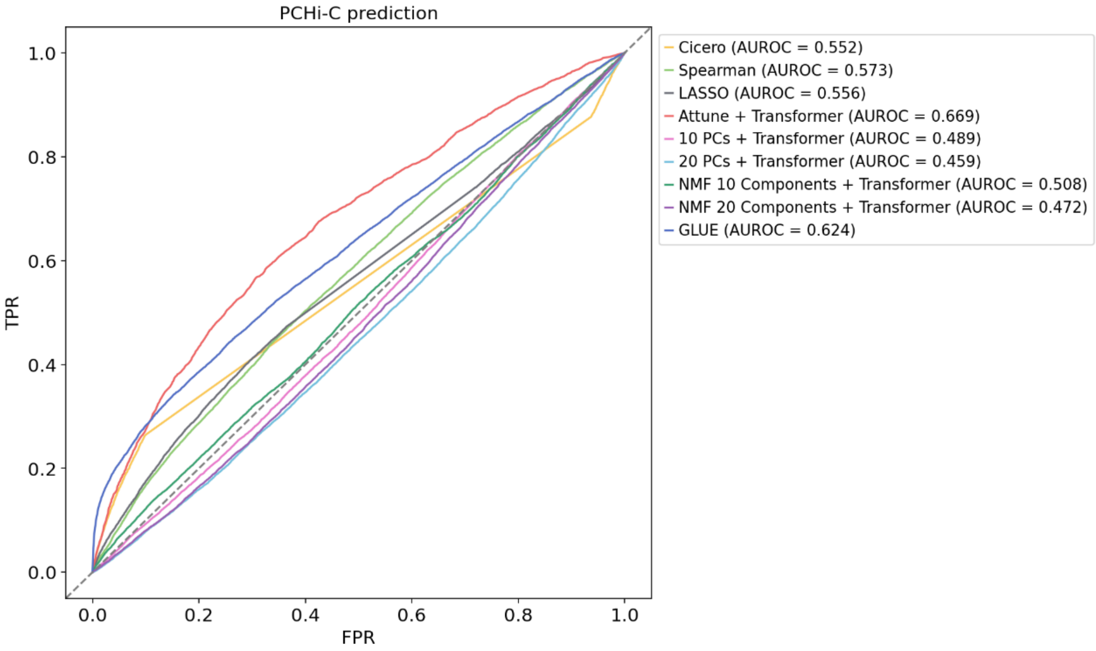

Response Figure 14. Comparison of Regulatory Prediction Performance

Reviewer#1 comments 5. It is surprising that all methods perform poorly in predicting distal interactions (Figure 3b). Even though the author showed that Attune outperforms other existing methods, the prediction results in Figure 3b doesn't justify that Attune can accurately identify distal promoter interactions (The false positive rate is so high). The author needs a clear explanation on such issue and adds additional benchmark data for this task.

Response to Reviewer#1 comments 5:

Thank you for your insightful comments regarding the performance of our model and the challenges associated with predicting distal promoter interactions. We agree that Figure 3b highlights notable limitations, particularly in accurately identifying distal interactions, and we provide additional context and detailed interpretations below.

The importance of distal interactions in benchmarking. Distal promoter interactions are biologically significant and essential to understanding gene regulation, even though they are challenging to predict. In our study, we included interactions up to 1.2 Mbps based on the statistical distribution of interaction distances in the dataset from Javierre et al. (2016) [1]. Studies such as Laverré et al. (2022) further emphasize the critical role of long-range interactions in gene expression and chromatin organization [2]. However, most existing methods primarily focus on short-range interactions (e.g., GLUE ≤150 kb, Cicero ≤500 kb). By intentionally including distal interactions in our benchmarks, we aim to highlight their biological importance and encourage improvements in predictive models for these relationships. Ignoring distal interactions could risk overlooking key regulatory mechanisms and limit the development of predictive methods for studying long-range regulation.

Performance trends and challenges of distal predictions. From the PCHi-C dataset results, we observe that true regulatory interactions decrease in frequency as distance increases (see Supplementary Figure 4a), which likely reflects a combination of biological and dataset-specific constraints. Nonetheless, most methods demonstrate higher differentiation capacity for short-range interactions (e.g., 25-50 kb and 50-75 kb bins), as shown in Response Figure 15. The boxplots illustrate that differentiation between true positive (TP) and true negative (TN) interactions diminishes at longer distances for most methods, reflecting the added complexity of long-range regulation. Nonetheless, Attune demonstrates relatively stronger differentiation capability in distal bins compared to other methods, indicating it may capture certain features of long-range regulation. Additionally, Calculating FDR uniformly across methods presents challenges due to the differing scoring systems used by each method (e.g., Spearman's correlation for association strength, Cicero's co-accessibility score for chromatin accessibility relationships). As these scores vary in scale and interpretation, defining fair and consistent thresholds across methods is challenging due to differing scoring systems. Instead of relying on thresholds, we visualized score distributions across distance bins to provide a more intuitive and method-agnostic comparison.

Inclusion of the eQTL v10 dataset. In response to your suggestion, we expanded our analysis to include the eQTL v10 dataset, which evaluates significant gene-peak pairs from whole blood samples. This dataset complements the PCHi-C data and allows us to assess Attune's generalizability across broader biological contexts. Since Attune was trained on PBMC data (primarily T cells, B cells, and monocytes), the inclusion of whole blood data helps evaluate its performance in a bulk context. However, Attune does not perform as well on the eQTL dataset, where GLUE achieves higher AUROC scores, likely due to its use of prior knowledge-based guidance graphs that incorporate additional regulatory context. For Attune, we hypothesize that incorporating such biological priors (e.g., enhancer-promoter interaction maps or tissue-specific chromatin features) might improve its performance in tasks involving distal interactions. Additionally, we hypothesize that Attune's predictions may be sensitive to cell-type composition, which could impact its robustness in datasets without cell-type annotations, such as eQTL v10. This remains an aspect requiring further investigation.

Our analysis underscores the importance of including distal interactions in benchmarking studies despite the challenges associated with their prediction. While these challenges are shared across all methods, our results show that Attune demonstrates stronger differentiation capacity in distal bins compared to other approaches. However, this study also highlights the need for future improvements, including integrating biological priors and leveraging single-cell datasets with detailed cell-type annotations to enhance robustness and performance.

We appreciate the reviewer's comments, which prompted us to expand our analysis and address the challenges of distal interaction prediction more comprehensively. If additional details or analyses are required, we would be happy to provide them. Thank you again for your thoughtful feedback.

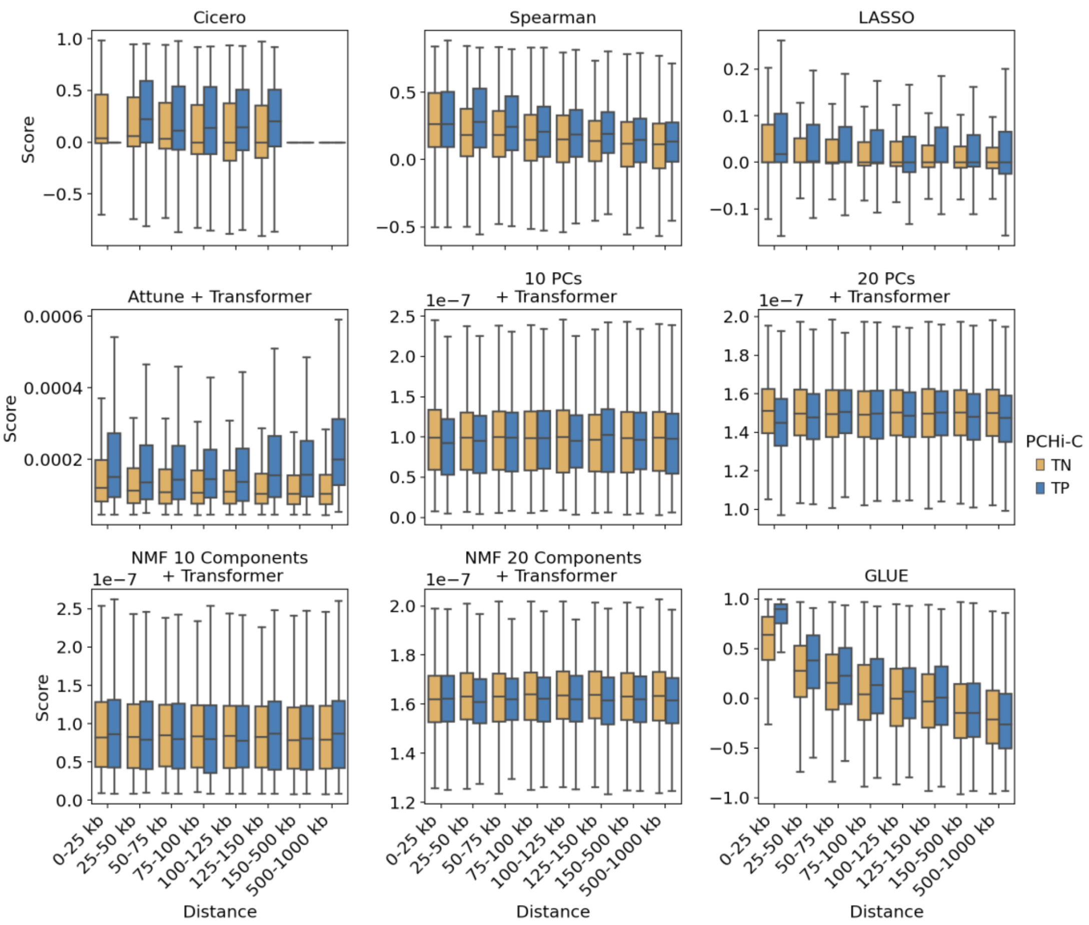

Response Figure 15. Score Distributions Across Distance Bins. The y-axis represents method-specific scores, reflecting different biological interpretations (e.g., Spearman's correlation, Cicero's co-accessibility).

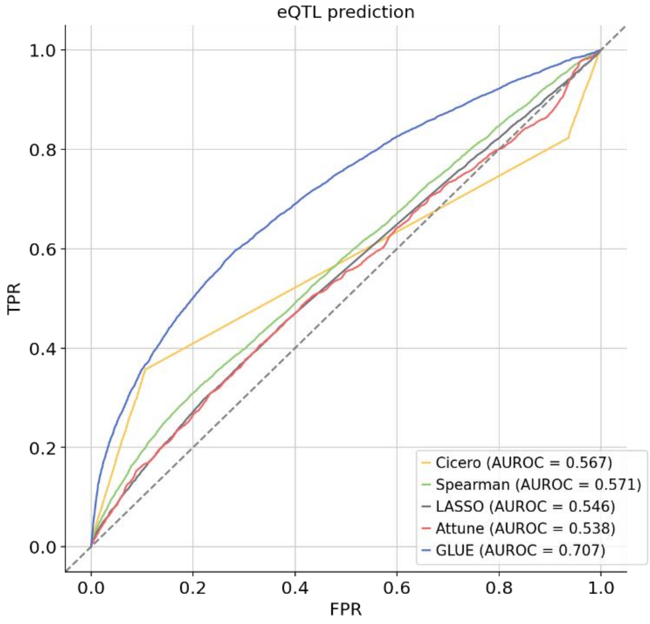

Response Figure 16. Predictive Performance of Regulatory Interaction on eQTL Dataset.

[1] Javierre, Biola M., et al. "Lineage-specific genome architecture links enhancers and non-coding disease variants to target gene promoters." Cell 167.5 (2016): 1369-1384.
[2] Laverré, Alexandre, Eric Tannier, and Anamaria Necsulea. "Long-range promoter–enhancer contacts are conserved during evolution and contribute to gene expression robustness." Genome Research 32.2 (2022): 280-296.

Reviewer#1 comments 6. It is not obvious that the chromatin regions presented in Figure 4c explain well the bifurcation of lineage commitment in mouse skin data. First, what are key peaks that drive the lineage commitment into the upper branch (IRS and TAC2). Second, these peaks seem to be not differentiable in the lower branch. Say, what drives the bifurcation into Medulla and Hair shaft-cuticle.

Response to Reviewer#1 comments 6:

Thank you for your insightful comments. To clarify the analysis presented in Figure 4c, our initial hypothesis was that Gli3 plays an important role in lineage commitment, based on the inconsistencies observed between two modalities. We then investigated the chromatin accessibility peaks associated with Gli3. Since differentiation is a time-dependent process, we performed pseudotime analysis to explore the relationship between peak accessibility and cell maturation. During this analysis, IRS cells were excluded because, as noted in both Ma's study and our results, IRS consistently occupies an intermediate position along the pseudotime trajectory, whereas hair shaft-cuticle/cortex and medulla cells are found at the differentiation endpoint [1, 2]. Given that IRS, medulla, and hair shaft-cuticle/cortex represent distinct differentiation fates, we focused our pseudotime analysis on the outer sheath differentiation pattern and excluded IRS cells to avoid confounding the results. This decision was documented in the GitHub code provided with the manuscript. However, as you rightly pointed out, this rationale was not sufficiently explained in the main text, leading to potential misinterpretation.
The primary objective of this experiment was to investigate how Gli3 contributes to overall hair follicle development (on a broader scale) rather than its specific role in determining the bifurcation into medulla or hair shaft. At the current stage, our analysis does not resolve how individual Gli3-associated peaks might guide differentiation toward specific lineages. This limitation likely requires more intricate network analyses, integrating multi-gene and multi-pathway interactions, to comprehensively address the underlying mechanisms.
We will revise the manuscript to explicitly clarify these points and provide additional context to avoid similar ambiguities. If additional clarifications or analyses are needed, we are happy to provide further details. Thank you again for your thoughtful comments and for helping us improve the clarity and rigor of our work.

[1] Ma, Sai, et al. "Chromatin potential identified by shared single-cell profiling of RNA and chromatin." Cell 183.4 (2020): 1103-1116.
[2] Abe, Yoshinori, and Nobuyuki Tanaka. "Roles of the hedgehog signaling pathway in epidermal and hair follicle development, homeostasis, and cancer." Journal of

developmental biology 5.4 (2017): 12.

Reviewer#1 comments 7. The author made a claim that "The overall upward shift of blocks indicates the lag of RNA modality", backed up by presentation of Figure 5c and Supplementary Figure 8c. These two figures are not straightforward to make such conclusion. It is suggested for the author to provide a more quantitative visualization or statistical examination to illustrate such transition.

Response to Reviewer#1 comments 7:

We greatly appreciate the reviewer's insightful suggestion to enhance the quantitative rigor of our analysis supporting the claim that "the overall upward shift of blocks indicates the lag of RNA modality." In the original analysis, we used embedding techniques to explore the alignment between RNA and ATAC modalities, primarily to assess whether any cell types exhibit poor modality alignment. This misalignment could suggest a lag in RNA modality. Although the upward shift in the heatmap (Figure 5b), the clustering patterns in the UMAP (Figure 5c), and the distribution of cosine distances (Supplementary Figure 8c) indicate a potential RNA modality lag in certain cell types, we acknowledge that these visual representations alone may not provide sufficient statistical validation.

To address this, we conducted additional statistical analyses to provide a more robust quantitative comparison of modality mismatch across cell types. Specifically, we calculated the distribution of cosine distances (1 - cosine similarity) for each cell type and visualized these results using a boxplot (Response Figure 17). Statistical comparisons using the Wilcoxon rank-sum test revealed significant differences in modality mismatch between nIPC/ExN and other cell types ($p = 1.68e^{-117}$), as well as between ExM and other cell types ($p = 7.85e^{-65}$). These findings quantitatively confirm that nIPC/ExN and ExM exhibit elevated modality mismatch compared to other cell types, thus directly supporting the claim of RNA modality lag in these regions.

We sincerely thank the reviewer for their valuable feedback, which has contributed significantly to improving the clarity and rigor of our analysis. Should further clarifications or additional analyses be required, we would be happy to provide them.

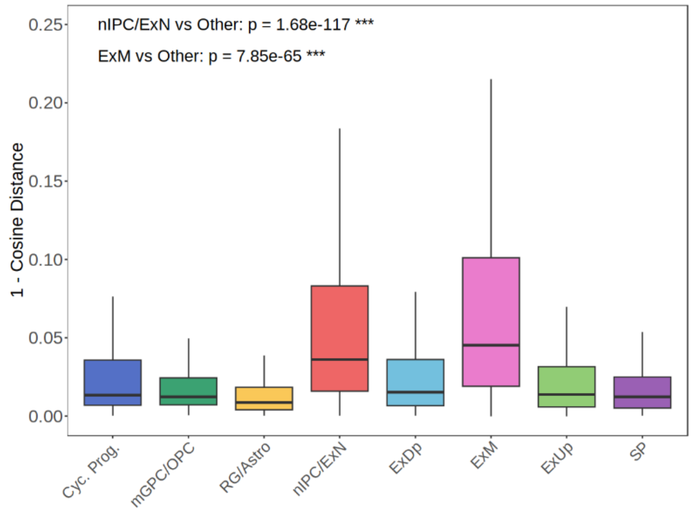

Response Figure 17. Modality Mismatch Across Cell Types.

We sincerely thank Reviewer#2 for the thoughtful and constructive feedback on our manuscript. Your suggestions have been invaluable, and we have revised the manuscript to address your concerns. Below, we respond to each comment in detail.

Reviewer#2 comments 1. The notations and descriptions in the Methods section in the whole manuscript are quite confusing, making it difficult to follow. A lot of current presentations do not adhere to standard mathematical notation practices. For example:
a. x_indices and x_counts are defined multiple times with different dimensions.
b. In the line "outputs the weighted hidden vector h, h ∈ R (equation 2)." it is unclear how an observed gene expression variable like x_counts can "output" a hidden vector.
c. Equations 1-8 would benefit from clearer mathematical notations to denote gene embedding, gene hidden states, and use math notations to denote Embedding() as a function, ensuring clarity.
d. The meaning of certain operations, such as those in equations 2 and 4, is unclear—are these referring to element-wise multiplication or vector multiplications?
e. Additionally, variables such as m and m+ are defined only after they are first introduced, which disrupts the logical flow.
f. l() in 26 is not defined.
These are some of the examples. Completely rewriting of the whole method section is probably needed to improve clarity.

Response to Reviewer#2 comments 1:

We would like to sincerely thank Reviewer#2 for the detailed and constructive feedback. Your valuable comments have highlighted several areas that required attention, and we have thoroughly reviewed and revised the Methods section accordingly. Below, we respond to each of the specific points you raised:

a. On the multiple definitions of $X_{indices}$ and X_{counts} with inconsistent dimensions:

Our model uses two input modalities: single-cell ATAC-seq (scATAC-seq) and single-cell RNA-seq (scRNA-seq). For scRNA-seq, the data is organized into a matrix with 10,000 rows (representing individual cells) and 2,000 columns (representing genes). Each column corresponds to a unique gene index ranging from 1 to 2,000, where "gene 1" is in the first column with an index of 1. In the manuscript, "i" denotes the index of a gene within the matrix, and "G" represents the total number of genes, which in this case is 2,000. The matrix "$X_{indices}$" contains the gene indices and has dimensions of (N x G), where N is the number of cells and G is the number of genes. Each row of "$X_{indices}$" contains the

sequence [1, 2, 3, ..., 2000]. "X_{counts}" refers to the gene expression matrix, which has the same dimensions as "$X_{indices}$" (N x G). We have now unified the definitions and descriptions of "$X_{indices}$" and "X_{counts}" for consistency and clarity.

b-d.

Modify "Teacher network" section in Methods.

For two kinds of single-cell data (scRNA-seq and scATAC-seq), we designed two teacher networks (RNA teacher network and ATAC teacher network) to learn fine-grained representations respectively. The RNA teacher network accepts $X_{indices} \epsilon \mathbb{R}^G$ and $X_{counts} \epsilon \mathbb{R}^G$ as input, where G denotes the number of genes. $X_{indices}$ represents gene indices, which dimension is (N×G) and X_{counts} represents the value of gene expression, which dimension is (N×G). N is the number of cells. For ATAC teacher network accepts input of $Y_{indices} \epsilon \mathbb{R}^P$ and $Y_{counts} \epsilon \mathbb{R}^P$, where P denotes the number of peaks. $Y_{indices}$ represents peak indices, which dimension is (N×P) and Y_{counts} represents the value of peak counts, which dimension is (N×P). Each gene within a cell is represented by $i \epsilon \mathbb{Z}^G$ and each peak is represented by $j \epsilon \mathbb{Z}^P$.

In the teacher network, the embedding layer maps discrete inputs such as genes, peaks, etc. to continuous vector space. The input of embedding layer of RNA teacher network is each row of $X_{indices}$, that is, an integer sequence x_1, x_2, ⋯, x_G, where each x_i represents the index of a gene. The purpose of the embedding layer is to map these integers into a dense vector $gene\ \ embed_i \in \mathbb{R}^d$ (a $\mathbf{d}$-dimensional embedding vector corresponding to x_i). The embedding layer can be represented by a matrix $E_{RNA} \in \mathbb{R}^{G \times d}$, where G is the number of genes, and $\mathbf{d}$ is the dimension of the embedding. $E_{RNA}[x_i]$ represents the extraction of the x_i row (i.e. the embedding vector corresponding to the gene $\mathbf{i}$) from the matrix E_{RNA} through a table lookup operation (equation 1). Then the RNA expression of gene $\mathbf{i}$ ($count_i \in \mathbb{R}$) from X_{counts} is element-wise cross-multiplied with its embedding vector ($gene\ \ embed_i$) to obtain the weighted embedding vector $gene\ \ hidden_i \in \mathbb{R}^d$ (equation 2). For scATAC-seq data, we use y_j to represent the index of a peak in $Y_{indices}$ and the embedding layer of ATAC teacher network is represented by a matrix $E_{ATAC} \in \mathbb{R}^{P \times d}$, where P is the number of peaks. The embedding vector $peak\ \ embed_j$ of peak $\mathbf{j}$ is obtained by table lookup operation (equation 3). The peak counts of peak $\mathbf{j}$ ($count_j \in \mathbb{R}$) from Y_{counts} is element-wise cross-multiplied with its embedding vector ($peak\ \ embed_j$) to obtain the weighted embedding vector $peak\ \ hidden_j \in \mathbb{R}^d$ (equation 4).

$$gene\ \ embed_i = E_{RNA}[x_i] \tag{1}$$
$$gene\ \ hidden_i = gene\ \ embed_i \times count_i \tag{2}$$
$$peak\ \ embed_j = E_{ATAC}[y_j] \tag{3}$$
$$peak\ \ hidden_j = peak\ \ embed_j \times count_j \tag{4}$$

e. Definition of variables such as m and m+ introduced only after they are first used:

Modify "Overview of model architecture" section in Methods.

As illustrated in Figure 1b, the input of Attune is a multimodal dataset, also called joint profiling data, which contains information from two modalities, and the cells of the two modalities are paired. Attune is designed based on joint profiling data, which considers the pairing information. We consider the cells in paired scRNA-seq and scATAC-seq as positive pairs (m and m^+ is a pair of positive samples).

The overarching model architecture encompasses the Attune pre-training model, which comprises two asymmetric teacher-student networks, along with two modules dedicated to downstream tasks: the cross-modal prediction module and the transformer-based peak-gene interaction module. Attune leverages separate teacher-student networks to learn cell embeddings from scRNA-seq and scATAC-seq respectively, through cross-modal contrastive learning. The teacher network, designed to be more complex, employs a hierarchical attention mechanism [67], while the student network uses a simpler dense operation.

Learn the representation of cells in two modalities (scRNA-seq and scATAC-seq) by maximizing the consistency between positive pairs in the embedding space. The representations from both modalities are then projected into a common space.

f. Definition of the function l() in equation 26:

Modify "Training objectives" section in Methods.

We train transformer with two objectives function: contrastive loss between $gene\ \ hidden_{CLS}$ and $peak\ \ hidden_{CLS}$, RNA-ATAC modality matching loss. $gene\ \ hidden_{CLS}$ and $peak\ \ hidden_{CLS}$ are CLS tokens in different modalities with N*1*d dimension. They learn the weighted average embedding representing the entire genes or peaks. The purpose of comparing the two embeddings is to shorten the distance between the matched RNA-ATAC pairs globally. The calculation process of contrastive loss of CLS tokens, is described as equation (24-26).

$$s_{\alpha,\beta} = sim\left(gene\ \ hidden_{CLS_\alpha}, peak\ \ hidden_{CLS_\beta}\right) \tag{24}$$

$$s^+_{\alpha,\beta} = sim\left(peak\ \ hidden_{CLS_\beta}, gene\ \ hidden_{CLS_\alpha}\right) \tag{25}$$

where $sim(h_1, h_2)$ is defined as:

$$sim(h_1, h_2) = \frac{h_1^T h_2}{\tau \|h_1\| \|h_2\|}$$

$$\mathcal{L}_{CLS} = \frac{1}{2N} \sum_{m=1}^{N} [\ell(m, m^+) + \ell(m^+, m)] \tag{26}$$

where $\ell(m, m^+)$ is defined as:

$$\ell(m, m^+) = -log \frac{\exp(s_{m,m^+})}{\sum_{k=1}^{2N} \mathbb{1}_{[k \neq m]} [\exp(s_{k,m}) + \exp(s_{k,m^+})]}$$

where $\ell(m^+, m)$ is defined as:

$$\ell(m^+, m) = -log \frac{\exp(s^+_{m,m^+})}{\sum_{k=1}^{2N} \mathbb{1}_{[k \neq m^+]} [\exp(s^+_{k,m^+}) + \exp(s^+_{k,m})]}$$

where τ is the adjustable temperature coefficient, which can be used to scale the degree of pushing apart negative samples.

We sincerely hope that the revisions we have made effectively address your concerns. Regarding your suggestion to rewrite the entire Methods section, we have carefully reviewed and reorganized it to enhance clarity. We deeply appreciate your insightful feedback, which has significantly contributed to improving the overall quality of the manuscript.

Reviewer#2 comments 2. Given that Attune uses paired information in the loss function, is it fair to compare this with other unpaired methods in the data integration performance, such as scJoint? A note should be made for the unpaired methods to clarify.

Response to Reviewer#2 comments 2:

We thank Reviewer#2 for raising this important point. Initially, we selected scJoint and other unpaired methods for comparison due to the limited availability of methods specifically designed for paired data integration at the time. However, we acknowledge that comparing a paired method like Attune with unpaired methods such as scJoint and GLUE may introduce fairness concerns, as these unpaired methods, while capable of handling paired data, do not fully leverage pairing labels.
In response to this concern, and as detailed in our reply to Reviewer#1's Comment 3, we have now included additional benchmarking with other paired data methods such as Seurat V5, SMILE, and Concerto. The updated results show that even when compared to these paired methods, Attune maintains competitive performance, further supporting its efficacy in multimodal integration. Since the detailed benchmarking results are already provided in the response to Reviewer#1's Comment 3, we do not repeat the results here, but we encourage the reviewer to refer to that section for further details. We hope this addresses the fairness concern raised by Reviewer#2.

Reviewer#2 comments 3. Methods and evaluation metrics that are mentioned in the recent benchmarking paper in single-cell multi-omics prediction and integration (Hu et al.) should be considered in this manuscript to improve the evaluation and benchmarking.
Hu, Y., Wan, S., Luo, Y. et al. Benchmarking algorithms for single-cell multi-omics prediction and integration. Nat Methods (2024).

Response to Reviewer#2 comments 3:

We thank Reviewer#2 for the valuable suggestion. In response, we have indeed benchmarked our method against the recent benchmarking work by Hu et al. (2024) on single-cell multi-omics prediction and integration [1]. A detailed discussion on predicting chromatin accessibility from scRNA-seq data is provided in our response to Reviewer#1's Comment 1.

Regarding the integration of RNA expression and chromatin accessibility (referred to as vertical integration), we benchmarked Attune alongside several algorithms highlighted in the benchmarking paper by Hu et al., including scAI, MOJITOO, MultiVI, Seurat, scVAEIT, MOFA+, Multigrate, scMVP, MIRA, DeepMAPS, SCOIT, and Schema. These comparisons were performed across 11 RNA + ATAC datasets (see Response Figure 18-20), which are joint profiling data derived from various technologies such as SNARE-seq, SHARE-seq, ISSAAC-seq, 10x Multiome, and DOGMA-seq (Response Figure 21). For the evaluation of vertical integration, we used multiple metrics, including ARI (Adjusted Rand Index), NMI (Normalized Mutual Information), cASW (cell-type labels average silhouette width), cLISI (cell-type separation LISI), and BVC (biological variation conservation). ARI and NMI were used to assess the consistency between cell-type labels and clustering results obtained through the Leiden algorithm. A higher cASW value indicates better accuracy in cell-type separation, while higher cLISI values suggest more effective cell-type separation, indicating better preservation of biological variation. The BVC metric provides a comprehensive performance evaluation, aggregating several metrics (ARI, NMI, cASW, and cLISI) into a single score.

In terms of NMI and ARI, MOJITOO achieved the highest scores, followed by Attune and scAI (Response Figure 18). For the cASW and cLISI metrics, Attune outperformed the other algorithms (Response Figure 19). These results indicate that, while Attune's performance in cell clustering was slightly lower than that of MOJITOO, it demonstrated superior cell-cell similarity representation, which is a critical factor for accurate integration. Overall, Attune, scAI, and MOJITOO performed the best in terms of vertical integration, as shown in Response Figure 19. We would like to emphasize that the datasets and evaluation pipeline used in our vertical integration benchmarks are consistent with those described in the benchmarking paper by Hu et al. (2024). The data can be accessed at https://mailustceducn-my.sharepoint.com/:f:/g/personal/hyl2016_mail_ustc_edu_cn/EgYFP7tlTKBBuAhkdtrIOg4B1Eyo-_iBx1VKBWSK0r-9rA?e=gmhocx, and the evaluation pipeline is available at https://github.com/QuKunLab/MultiomeBenchmarking/blob/main/code/Integration/compare/count_metrics_ATAC.ipynb.

We hope these additional details address your concerns and strengthen our manuscript.

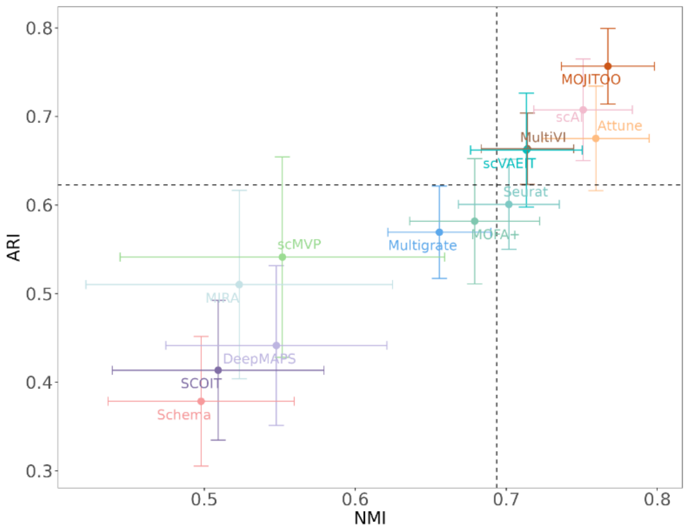

Response Figure 18. Average ARI vs average NMI. The dashed line is the median of all algorithm results. The error bars represent the standard deviation of the 11 data sets. Data are presented as mean ± 0.5 times the standard deviation. Same below.

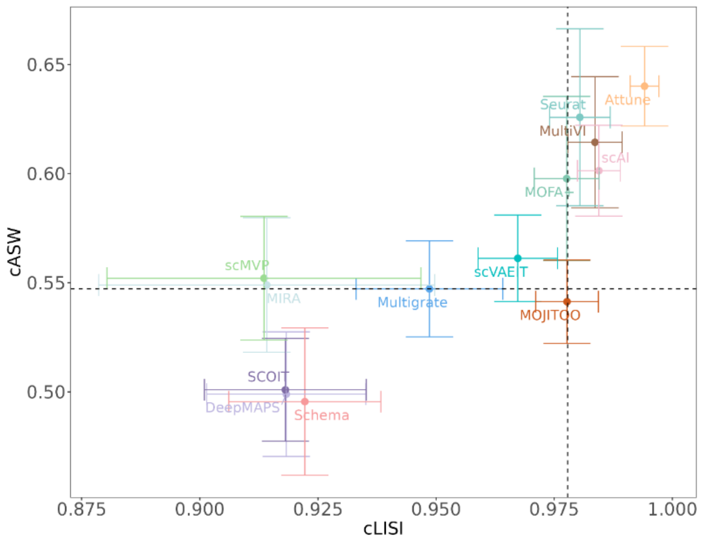

Response Figure 19. Average cASW vs average cLISI.

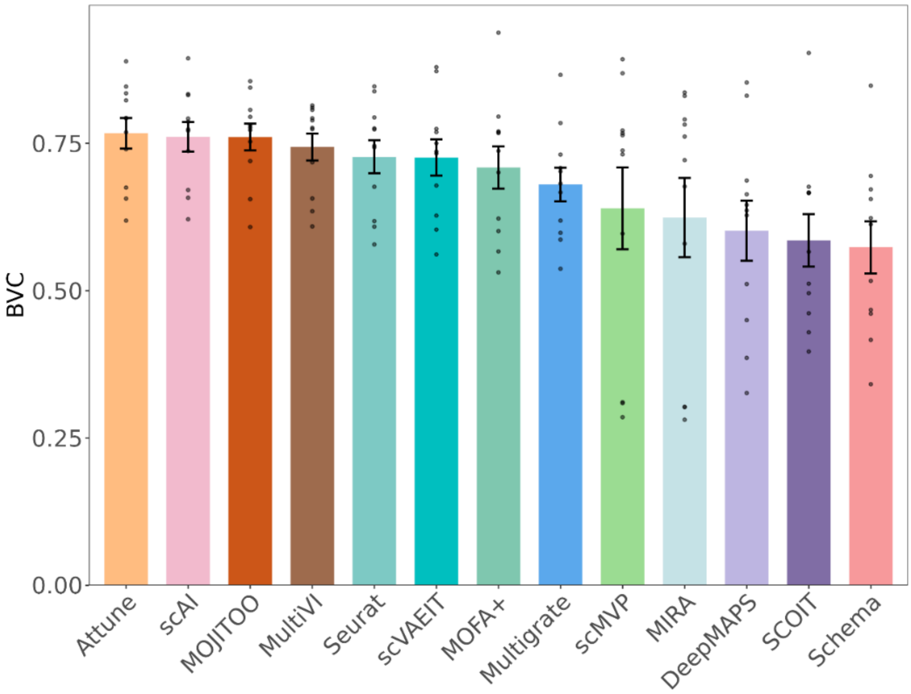

Response Figure 20. Bar plots illustrate the overall performance of these algorithms, evaluated by BVC scores on 11 RNA+ATAC datasets. Data are presented as mean and 95% confidence interval. Each point represents the BVC score of an algorithm on a dataset.

Technique	Type/Tissue	Species	ID	Dataset ID	Used batch	#Cells	#RNA	#Peak
SNARE-seq	Adult Brain	Mouse	GSE126074	Dataset 31	All	8055	12775	90358
ISSAAC-seq	Adult Brain	Mouse	E-MTAB-11264	Dataset 32	All	10361	15342	169134
10x Multiome	Adult Brain	Human	NULL	Dataset 33	All	2855	16910	132465
	PBMCs	Human	NULL	Dataset 34	All	10137	15408	139470
	PBMCs	Human	NULL	Dataset 35	All	2592	11755	80445
	PBMCs	Human	NULL	Dataset 36	All	8105	14184	102360
	PBMCs	Human	NULL	Dataset 37	All	2413	10860	60639
	Retina	Mouse	GSE201402	Dataset 38	All	9383	6275	59353
	BMMCs	Human	GSE194122	Dataset 39	s4d8	9876	13431	112089
DOGMA-seq	PBMCs	Human	GSE156478	Dataset 40	Control	7468	28310	68825
				Dataset 41	Stim	5915	28310	68825

Response Figure 21. 11 single-cell RNA+ATAC datasets.

[1] Hu Y, Wan S, Luo Y, et al. Benchmarking algorithms for single-cell multi-omics prediction and integration[J]. Nature Methods, 2024: 1-13.

Reviewer#2 comments 4. Another very recent method that should be considered to benchmark in terms of gene regulatory identification is SCENT.
+ Sakaue, S., Weinand, K., Isaac, S. et al. Tissue-specific enhancer-gene maps from multimodal single-cell data identify causal disease alleles. Nat Genet 56, 615-626 (2024).

Response to Reviewer#2 comments 4:

We sincerely thank Reviewer#2 for suggesting the inclusion of SCENT as a benchmark for gene regulatory identification. In response to this recommendation, we attempted to run SCENT on our PBMC dataset, categorizing CD4, CD8, and Treg cells as T cells, and testing a total of 87,436 gene-peak pairs, consistent with the benchmarks we conducted for Attune, GLUE, and other methods.

Despite allocating up to 100 threads for the analysis, the computation did not complete after two days of continuous running. Due to resource constraints, we adjusted the number of threads and allowed the task to continue running. However, as of now, results are still pending. We will continue monitoring the process and, if feasible, aim to include the results in our next response. We appreciate your understanding and will make every effort to provide a comprehensive evaluation as soon as possible.

Reviewer#2 comments 5. In addition to the PCHiC database, other resources can be considered for validating the identified gene regulatory regions, such as the GTEx eQTL database and the ABC enhancer database. It would be good to incorporate these resources in benchmarking.

Response to Reviewer#2 Comment 5:

We thank Reviewer#2 for the valuable suggestion to incorporate additional resources for validating gene regulatory regions. In response, we have expanded our analysis to include the eQTL v10 dataset, as detailed in our response to Reviewer#1's Comment 5. The eQTL v10 dataset evaluates significant gene-peak pairs from whole blood samples, providing a complementary perspective to the PCHi-C data.

Our analysis showed that Attune performs better on the PCHi-C dataset, likely due to its closer match with the PBMC-specific cell types used for training. However, on the eQTL v10 dataset, Attune's performance was less competitive, suggesting that its predictions may be influenced by cell-type composition and the absence of detailed cell-type annotations in this dataset. This finding highlights the potential for future improvements, such as incorporating biological priors (e.g., tissue-specific chromatin features) or training on datasets with broader cell-type diversity to enhance its robustness.

Regarding the ABC enhancer database, as it provides model-derived predictions rather than experimentally validated interactions, we prioritized the eQTL v10 dataset as a supplementary resource to the PCHi-C data. While incorporating additional datasets is always valuable, we focused on experimentally validated data for this benchmarking.

We appreciate the importance of evaluating model performance across diverse datasets and believe these insights will guide future improvements to Attune, enhancing its generalizability and applicability.

Reviewer#2 comments 6. The definition of this manuscript of DPAGs (dense peak-associated genes) has the same definition of DORC. This overlap should be discussed within the paper. Additionally, many of the analyses performed on the SHARE-seq data in this manuscript, such as DORC identification and residual analysis, are very similar to those in the original publication by Ma et al. The authors should elaborate

on the similarities and differences in findings between the two studies. For example, are the identified DORCs consistent between the studies? Are the residuals highly correlated? At present, it is unclear what new biological insights are gained exclusively from the application of the propsoed method.

Ma, S., Zhang, B., LaFave, L. M., Earl, A. S., Chiang, Z., Hu, Y., ... & Buenrostro, J. D. (2020). Chromatin potential identified by shared single-cell profiling of RNA and chromatin. Cell, 183(4), 1103-1116.

Response to Reviewer#2 Comment 6:

We thank Reviewer#2 for raising this important point about the overlap between the definitions of DPAGs (dense peak-associated genes) and DORCs (domains of regulatory chromatin) [1]. We acknowledge that both approaches share a similar conceptual basis in identifying genes based on their strong associations with regulatory peaks. However, we believe that the differences in methodology between the two analyses are substantial and merit clarification.

（1）Differences in Input Data: The input RNA and ATAC feature dimensions differ between DPAGs and DORCs. Due to the computational demands of Transformer-based models, we reduced the number of genes and peaks used in our analysis. In contrast, DORC analyses typically include a much larger set of features, which affects the associations that can be identified and their scope.

（2）Differences in Window Size: DORC analysis uses a 50 kb window to capture local regulatory associations, while DPAGs in Attune employ a much larger window (>1 Mb) to ensure that distal regulatory interactions, which play a critical role in gene expression and chromatin organization, are also considered.

As detailed in our response to Reviewer#1 Comment 2, the computational demands of Transformer-based models necessitated reducing input feature dimensions to process the large number of peaks and their potential associations with genes efficiently. These constraints significantly impacted our ability to maintain consistency in input features between DPAGs and DORCs, making direct comparisons difficult. In particular, the differences in input size and window range (Points 1 and 2) lead to results that are inherently distinct, which is why we did not perform a direct overlap analysis.

With regard to residual analysis, we recognize that Ma et al. (2020) also utilized residuals based on DORCs, and the gene selection process can impact comparability. For example, in Ma et al., Wnt3 was highlighted as an example of lineage priming, but this gene was excluded from our analysis due to filtering for highly variable genes (HVGs). This difference in gene selection further complicates direct comparisons of residuals between the two studies. While our study was inspired by the framework established by Ma et al., we aimed to explore whether similar biological phenomena could be identified using our Attune + Transformer approach, which incorporates a distinct computational methodology. We also appreciate Reviewer#2's suggestion to discuss the similarities and differences between our study and the work of Ma et al. (2020). Their study provides a comprehensive framework for identifying regulatory elements with an impressive depth of analysis, which we highly respect. Our work seeks to build on this foundation by

introducing a Transformer-based approach, though we acknowledge that scaling our method to datasets with hundreds of thousands or millions of features (such as peaks) presents challenges.

In future work, we plan to optimize our model by leveraging the attention mechanism to better capture modality relationships, aiming to provide a more robust, efficient, and convincing analytical framework. This approach will also address current challenges related to feature selection and computational resource demands, enabling more effective and scalable analysis.

[1] Ma, Sai, et al. "Chromatin potential identified by shared single-cell profiling of RNA and chromatin." Cell 183.4 (2020): 1103-1116.

Reviewer#2 comments 7 (minor). The details of the method should be described and highlighted the innovation in the first section of Results, and reference Figure 1b, which is currently not referred to in the main text.

Response to Reviewer#2 Comment 7:

Thanks for your suggestion.

Modify “Attune achieves exceptional overall performance of integration” in main text.

Attune employs a cross-modal contrastive learning approach to integrate scRNA-seq and scATAC-seq data, effectively preserving biological consistency across both modalities. The architecture consists of the Attune pre-trained model and two downstream modules: the cross-modal prediction module and the Transformer-based peak-gene interaction module. To learn cell embeddings from scRNA-seq and scATAC-seq data, Attune utilizes two asymmetric teacher-student networks, which are trained through cross-modal contrastive learning. These learned cell embeddings can be fine-tuned for various downstream tasks, including cross-modal prediction, peak-gene interaction recovery, and differentiation analysis, as depicted in Figures 1a and 1b.

Reviewer#2 comments 8 (minor). In Method Section, Sentence "Positive pairs of embeddings from the two modalities of a cell are considered as positive pairs" is confusing as Positive pairs of embedding is not defined beforehand.

Response to Reviewer#2 Comment 8:

We appreciate Reviewer#2 for pointing out the lack of clarity in the Methods section. To address this, we have revised the explanation to provide better context for the definition of "positive pairs" and to ensure clarity for readers.

Modify “*Overview of model architecture*” section in Methods.

As illustrated in Figure 1b, the input of Attune is a multimodal dataset, also called joint profiling data, which contains information from two modalities, and the cells of the two modalities are paired. Attune is designed based on joint profiling data, which considers the pairing information. We consider the cells in paired scRNA-seq and scATAC-seq as positive pairs (m and m^+ is a pair of positive samples).

The overarching model architecture encompasses the Attune pre-training model, which comprises two asymmetric teacher-student networks, along with two modules dedicated to downstream tasks: the cross-modal prediction module and the transformer-based peak-gene interaction module. Attune leverages separate teacher-student networks to learn cell embeddings from scRNA-seq and scATAC-seq respectively, through cross-modal contrastive learning. The teacher network, designed to be more complex, employs a hierarchical attention mechanism [67], while the student network uses a simpler dense operation.

Learn the representation of cells in two modalities (scRNA-seq and scATAC-seq) by maximizing the consistency between positive pairs in the embedding space. The representations from both modalities are then projected into a common space.
