# Supplementary material for: Cross-modal contrastive learning decodes developmental regulatory features through chromatin potential analysis
Source: Gigascience. 2025 Oct 17;14:giaf053. doi: 10.1093/gigascience/giaf053 (PMC12532322; doi:10.1093/gigascience/giaf053)
Supplement: giaf053_Authors_Response_To_Reviewer_Comments_Original_Submission [file giaf053_authors_response_to_reviewer_comments_original_submission.pdf]

Author's Response To Reviewer Comments

We have included the figures, tables, and formulas in a separate document titled "Response.docx" (attached to the Personal cover) to address the reviewer's comments. The reason for using a separate file is that the website's input box does not support the inclusion of figures. By providing a standalone document, we aim to make it easier for the reviewer to access and evaluate our responses. While we have conducted several additional experiments based on the reviewer's suggestions, we are unsure if they fully meet the intended requirements. Therefore, we have refrained from making extensive changes to the original manuscript to avoid potential confusion if further revisions are necessary. Should we receive additional feedback, we are prepared to integrate and update the results accordingly to ensure the manuscript's quality.

We sincerely appreciate the time and effort the reviewers have dedicated to reviewing our manuscript titled "". We acknowledge the significance of addressing your concerns to ensure the clarity and comprehensibility of our work.

We are grateful for the valuable insights provided by Reviewer #1 in the feedback. And we will provide detailed explanations and clarifications for the raised comments, as we strive to address them effectively.

Reviewer#1 comments 1. As for model architecture presented in Figure 1b, the author included a one-way translation from ATAC counts to RNA counts. MSE loss was applied to minimize the reconstruction of translation from ATAC to RNA. What is the rationale behind such one-way translation? Why not translation from RNA counts to ATAC counts, or explicitly having bidirectional translation between these two modalities?

Response to Reviewer#1 comments 1:

Our approach was inspired by the multimodal single-cell data integration task presented at the NeurIPS 2021 competition, specifically Task 1: Modality Prediction [1]. This task focuses on predicting one modality from another, with performance evaluated using root mean squared error (RMSE). The biological rationale underlying this task is that genetic information flows from DNA to RNA to proteins. Chromatin accessibility (ATAC data) governs gene expression (RNA data), which in turn drives protein synthesis. By aligning with this biological hierarchy, our focus was to predict RNA (GEX) data from ATAC data, reflecting how chromatin accessibility regulates transcription.
In the context of modality prediction, previous works such as BABEL [2], MultiVI [3], and Polarbear [4] have demonstrated the feasibility of both RNA-to-ATAC and ATAC-to-RNA predictions. Building on this foundation, we chose to prioritize the ATAC-to-RNA direction for its biological relevance. However, to extend the model's capability, we incorporated a reverse flow (RNA to ATAC) into Attune's cross-modal prediction task (see Response Figure 1). We then benchmarked Attune against nine state-of-the-art algorithms, including LS_Lab, MultiVI, scVAEIT, LIGER, Seurat, BABEL, scMOG, scMoGNN, and CMAE, as described in a recent study on single-cell multi-omics prediction and integration [5].
To evaluate Attune, we utilized 11 single-cell RNA + ATAC datasets from a range of sequencing platforms, including SNARE-seq, SHARE-seq, ISSAAC-seq, 10x Multiome, and DOGMA-seq. We tested the model under two scenarios: intra-dataset (training and testing on the same dataset, see Response Figures 2A-2C) and inter-dataset (training and testing on different datasets, see Response Figures 2D-2F). Evaluation metrics included cell-cell PCC, peak-peak PCC, cell-cell CMD, peak-peak CMD, RMSE, AUROC, and Ranking Index (RI), providing a comprehensive assessment of prediction accuracy and generalizability. Specifically:
RMSE quantifies the deviation between predicted and actual values.
CMD measures differences in correlation matrices, indicating how well the model captures relationships between features.
PCC assesses chromatin accessibility abundance correlation.
AUROC evaluates the effectiveness of predicting chromatin accessibility.
RI summarizes overall algorithm performance.
A lower CMD and RMSE indicate better performance, while higher PCC and AUROC suggest more accurate predictions.
In the intra-dataset scenario, although Attune did not outperform LS_Lab or scVAEIT in all metrics, it achieved results

that were consistently above or near the median across most benchmarks. Overall, Attune ranked in the second tier among the algorithms, comparable to MultiVI and slightly outperforming Seurat (see Response Figure 3). In the inter-dataset scenario, Attune demonstrated strong generalizability alongside LS_Lab. Both models achieved cell-cell PCC, peak-peak PCC, and AUROC values above the median, while their CMD and RMSE values were below the median, indicating robust cross-dataset performance. As a result, Attune ranked in the top tier, comparable to LS_Lab (see Response Figure 4). When considering performance across both intra- and inter-dataset scenarios (see Response Figure 5), Attune exhibited robustness and adaptability. While LS_Lab achieved the best overall performance, Attune was comparable to MultiVI and scVAEIT, highlighting its effectiveness and versatility.
The datasets and evaluation pipeline used in this study are consistent with the benchmarking article by Hu et al. [5]. The datasets were obtained from https://mailustceducn-my.sharepoint.com/:f:/g/personal/hyl2016_mail_ustc_edu_cn/EgYFP7tlTKBBuAhkdtrIOg4B1Eyo-_iBx1VKBWSK0r-9rA?e=gmhocx, and the evaluation pipeline was implemented as described in the benchmarking repository https://github.com/QuKunLab/MultiomeBenchmarking/blob/main/code/Prediction/RNA_ATAC/Matrics.py.
We hope this explanation clarifies the rationale for our design and the comprehensive steps we took to evaluate Attune.

[1] Luecken, M.D., Burkhardt, D.B., Cannoodt, R., Lance, C., Agrawal, A., Aliee, H., Chen, A.T., Deconinck, L., Detweiler, A.M., and Granados, A.A. A sandbox for prediction and integration of dna, rna, and proteins in single cells. 2021.
[2] Wu K E, Yost K E, Chang H Y, et al. BABEL enables cross-modality translation between multiomic profiles at single-cell resolution[J]. Proceedings of the National Academy of Sciences, 2021, 118(15): e2023070118.
[3] Ashuach T, Gabitto M I, Koodli R V, et al. MultiVI: deep generative model for the integration of multimodal data[J]. Nature Methods, 2023, 20(8): 1222-1231.
[4] Zhang R, Meng-Papaxanthos L, Vert J, et al. Multimodal single-cell translation and alignment with semi-supervised learning[J]. Journal of Computational Biology, 2022, 29(11): 1198-1212.
[5] Hu Y, Wan S, Luo Y, et al. Benchmarking algorithms for single-cell multi-omics prediction and integration[J]. Nature Methods, 2024: 1-13.

Reviewer#1 comments 2. The gene-peak cross-attention seems to be a very big computation, not to mention the computation needed for the peak-peak self-attention (It is not clear to me if peak-peak self-attention is included in the model. If not, why peak-peak attention is unnecessary?). It is unclear what kind of computational power is enough to do the transformer fine-tuning. Meanwhile, it is also not clear why the author didn't combine transformer fine-tuning with pretraining as one end-to-end training? What are the benefits of dividing the model into two parts?

Response to Reviewer#1 comments 2:

Thank you for your insightful comments. We greatly appreciate the opportunity to further clarify the design and computational approach of our model.
Our methodology consists of two main components: pre-training the Attune model and performing downstream tasks. During the pre-training phase, the expression matrices of both ATAC-seq and RNA-seq data are input into the pre-training component. This phase employs unsupervised cross-modal contrastive learning, which is critical for setting up the model for subsequent tasks. The goal is to establish shared high-dimensional representations for cells across the two modalities (ATAC and RNA).
For tasks involving gene-peak interactions, the model generates embedding matrices for genes and peaks (with dimensions $N\times G\times d$ for genes and $N\times P\times d$ for peaks, where N represents the number of cells, G represents the number of genes, P represents the number of peaks, and d is the embedding dimension). These matrices are then passed through a Transformer model that uses self-attention and cross-attention mechanisms to derive a global cross-attention weight matrix ($G\times P$), which captures the gene-peak relationships. The self-attention mechanism operates on the gene embedding matrices ($N\times G\times d$) to model interactions between genes. The cross-attention mechanism operates between the peak embedding matrices ($N\times P\times d$) and the output of the gene self-attention mechanism ($N\times G\times d$) to capture the interaction between genes and peaks.
(1) Why not perform self-attention on peaks?
Due to hardware limitations (we are using a Quadro RTX 6000 with 24GB of memory), we are unable to perform self-attention on the peak embedding matrices. The number of peaks in typical datasets ranges from tens of thousands to hundreds of thousands, and the corresponding embedding matrices are typically very sparse. Performing self-attention on such sparse matrices would be computationally expensive and inefficient. Our testing has shown that performing self-attention on genes (with approximately 2,000 genes) and cross-attention between genes and peaks (with around 28,708 peaks) consumes approximately 24GB of memory on an RTX 6000 (batch size = 2) and 76GB on an A100 (batch

size = 12). These memory requirements indicate that, with our current hardware setup, performing self-attention on peaks is infeasible due to their large number and sparsity.
In our cross-attention mechanism, we use the gene embedding matrices, after self-attention, as the Query and directly use the peak embedding matrices as the Key and Value. When investigating regulatory interactions between genes, we aim to identify which peaks regulate each gene. It is therefore reasonable to use the gene embeddings as the Query, as they already capture associations between genes. Each gene in the Query computes the similarity (via vector dot product) with each peak in the Key. If a gene is highly correlated with a peak, the corresponding peak in the Value will be selected. This peak is then weighted by the similarity score, and the gene embedding matrix is updated accordingly, based on the relationship between the gene and the peak.
(2) Why not combine pre-training and fine-tuning into a single end-to-end process?
The decision to separate pre-training and fine-tuning is a deliberate design choice to address computational constraints while ensuring effective model training. By pre-training Attune using cross-modal contrastive learning, we ensure that the gene and peak representations share a high-dimensional latent space. During the downstream task, we freeze the weights of the Attune model and only fine-tune the Transformer. This separation prevents the contrastive learning process from disrupting the learned gene-peak associations during fine-tuning, thus ensuring stability in the shared space and improving the accuracy of downstream predictions. If we were to combine pre-training and fine-tuning into a single end-to-end training process, the backpropagation through both Attune and the Transformer might cause the learned gene-peak associations to shift, leading to potential instability. The contrastive learning process in Attune would update the distances between cell representations in the shared latent space, which could inadvertently affect the pre-established gene-peak associations. By separating these stages, we maintain a more stable learning process for the downstream task.
In summary, the decision to exclude self-attention on peaks and to split the pre-training and fine-tuning stages was driven by both computational constraints and the need for a stable and efficient learning process. We hope this explanation clarifies the rationale behind our model architecture and design choices.

Reviewer#1 comments 3. It is not clear how benchmarking was done. Both 10X multiome and SHARE-seq are joint profiling data. It is necessary for the author to provide more details about how other methods were run under author's hands, especially how the author handled data preprocessing for each method. Meanwhile, each cell has profiles of two modalities. Attune clearly takes advantage of knowing such correspondence. However, other methods primarily use shared features for integration, which will not take a full advantage of joint profiling. Seurat V3 was included in benchmarking. This version of Seurat also uses shared features. The author will need to consider adding Seurat V5 in benchmarking, which uses information of joint profiling for integration. Another existing contrastive learning SMILE was also proposed to integrate RNA-seq and ATAC-seq with joint-profiling data. Meanwhile, the author previously developed another contrastive learning integration method Concerto. It should also be included in benchmarking. The author may need to include more clear justifications on how Attune is improved from their previous work Concerto.

Response to Reviewer#1 comments 3:

Thank you for your insightful comments regarding the benchmarking methodology and the comparison with existing methods. We agree that it is important to clarify the distinction between multimodal integration and cross-modal alignment, as these are fundamentally different tasks with distinct objectives.
(1) Multimodal Integration: In our approach, Attune is designed for multimodal integration, where the goal is to combine data from paired modalities into a shared feature space. The model takes joint profiling data (where each cell contains profiles of both RNA and ATAC modalities) and learns a joint embedding that represents both modalities together. This approach aligns with the concept of joint embedding as outlined in the NIPS 2021 competition [1], which focuses on integrating features from different modalities into a unified representation. Attune leverages the correspondence between modalities within paired cells to optimize the integration, ensuring that both modalities are represented in a coherent and shared space.
(2) Cross-modal Alignment: In contrast, cross-modal alignment methods aim to establish correspondences between unpaired cells from different modalities. Historically, most single-cell methods were designed to analyze one modality at a time, but recent advances have introduced methods for measuring multiple modalities within the same cells. Methods such as GLUE, uniPort, Seurat V3, Cobolt, MinNet, scJoint, MultiVI, and sciCAN focus on aligning unpaired cells by mapping data from different modalities into a shared space using similarity metrics. These methods can also be applied to joint profiling data, but they do not fully exploit the pairing information. For example, scJoint is designed for unpaired data, but it can still be used with paired data, treating the RNA and ATAC components as separate datasets [2]. Similarly, GLUE has been benchmarked against unpaired methods like MMD-MA, LIGER, and Seurat V3 using paired data such as 10X Multiome and SHARE-seq [3].

Data Processing. In our benchmarking experiments, we followed the standard preprocessing procedures for multimodal data integration. Although the data is joint profiling, we treated the RNA and ATAC parts as separate datasets for the purpose of comparison with other algorithms. For scRNA-seq data, we removed genes expressed in fewer than 5% of cells and normalized the counts to 10,000 per cell using SCANPY. Additionally, sex chromosome genes were excluded, and the top 2000 highly variable genes (HVGs) were selected. For scATAC-seq data, we filtered out peaks detected in fewer than 5% of cells and removed sex chromosome peaks. These preprocessing steps ensured that the datasets were comparable while balancing computational efficiency and model performance. Methods like MultiVI and GLUE do not require the same number of features between modalities and can directly handle RNA and ATAC data. However, methods such as scJoint and sciCAN require common features between the modalities, so we first identified overlapping genes between RNA and ATAC datasets before applying the algorithms. After training, we computed integration metrics such as neighbor consistency, Seurat alignment score, and Fraction of Samples Closer Than the True Match (FOSCTTM).
Benchmarking with Multimodal Integration Methods. The key advantage of Attune lies in its ability to explicitly use the pairing information between modalities. This allows the model to treat the two modalities of the same cell as positive pairs, which results in a more precise alignment. Through contrastive learning, the model brings RNA and ATAC data closer in a high-dimensional space, yielding better performance in metrics like FOSCTTM compared to methods that rely solely on shared features. Unlike other methods that aim to create a single unified representation, Attune aligns RNA and ATAC data in a shared feature space while maintaining the identity of each modality, allowing for more accurate comparisons and analyses of the interactions between the modalities.
Concerto [4] supports multimodal integration. It is a simple element-wise summation of the output of the teacher network or student network for each modality (the embedding of cell in each modality). The contrastive loss (NT-Xent loss [5]) is calculated on the summed cell embeddings (from teacher network and student network, respectively). Concert can generate unified cell embeddings. In the case of two modalities (RNA and ATAC), we illustrate the corresponding operation according to equation (1) and (2) and (3), where the term $z_{teacher}^{RNA} \in R^d$ denotes cell embedding of RNA output by teacher network and $z_{teacher}^{ATAC} \in R^d$ denotes cell embedding of ATAC output by teacher network and $z_{student}^{RNA} \in R^d$ denotes cell embedding of RNA output by student network and $z_{student}^{ATAC} \in R^d$ denotes cell embedding of ATAC output by student network. Add denotes add along the dimension of embedding and Contrastive denotes NT-Xent loss and d denotes the dimension of cell embeddings.
The improvement of Attune over Concerto is that it does not need to sum the cell embeddings of the two modalities before calculating the contrastive loss, but directly calculates the contrastive loss for the cell embeddings of each modality (from the teacher network and the student network respectively). The above calculation process in shown in equation (4) and (5) and (6).
When using UMAP to visualize cell embeddings, Concerto performs UMAP on $z_{teacher}^{multi}$ to obtain unified cell embeddings （$N \times d$） that combines the two modalities (RNA and ATAC), while Attune concatenates $z_{teacher}^{RNA}$ and $z_{teacher}^{ATAC}$ to get joint cell embeddings （$2N \times d$）, which maps the cell embeddings of two modalities onto the same two-dimensional space. N represents the number of cells.
In response to the reviewer’s suggestion, we have included SMILE [6], Seurat V5 [7], and Concerto in the benchmarking process. These methods use joint profiling data for integration and are compared with Attune using several metrics: mean average precision (MAP), cell type adjusted silhouette width (ASW), neighbor consistency (NC), Seurat alignment score (SAS), batch adjusted silhouette width (Batch ASW), graph connectivity (GC), biology conservation, omics mixing, overall integration score, and FOSCTTM (see Methods for details). Since Seurat V5 maps scATAC-seq datasets onto scRNA-seq datasets when integrating different modalities, it only outputs cell representations of single modality and Concerto generates unified cell embeddings. Therefore, it is impossible to calculate the metrics that require the cell representation of each modality, such as Batch ASW, NC, SAS and FOSCTTM.
In the 10X Multiome dataset (Response Table 1), SMILE outperforms Attune in GC, but Attune shows superior results in most other metrics. Seurat V5 excels in MAP and cell type ASW but is outperformed by Attune in GC. Concerto performs similarly to Attune in MAP but lags behind in terms of robustness in cell type ASW and GC. UMAP visualizations for SMILE, Seurat V5, and Concerto are shown in Response Figures 6-9.
In the SHARE-seq dataset (Response Table 2), SMILE surpasses Attune in Batch ASW and GC but falls short in MAP and cell type ASW. Therefore, it is close to Attune in Omics mixing, but inferior to Attune in Biology conservation. Seurat V5 and Concerto also perform worse than Attune in MAP, cell type ASW, and GC. The UMAP visualizations for SMILE, Seurat V5, and Concerto are shown in Response Figures 10-13. These results were repeated five times with different random seeds to ensure robustness.

Response Table 1. Metrics for evaluating integration performance in 10X Multiome dataset

fold	algorithm	map	Batch ASW	Cell type ASW	GC	NC	SAS	FOSCTTM	Biology conservation	Omics mixing	Overall score
1	SMILE	0.695	0.858	0.515	0.989	0.366	0.589	0.008	0.525	0.812	0.640
2	SMILE	0.692	0.849	0.520	0.987	0.367	0.581	0.007	0.526	0.806	0.638

3 SMILE 0.688 0.855 0.510 0.980 0.361 0.582 0.008 0.520 0.806 0.634
4 SMILE 0.689 0.850 0.513 0.985 0.365 0.580 0.007 0.522 0.805 0.635
5 SMILE 0.691 0.852 0.515 0.981 0.365 0.584 0.008 0.524 0.806 0.636
1 Attune 0.716 0.951 0.540 0.934 0.211 0.915 0.018 0.664 0.929 0.770
2 Attune 0.729 0.954 0.545 0.937 0.212 0.975 0.023 0.708 0.955 0.807
3 Attune 0.740 0.943 0.552 0.928 0.213 0.892 0.012 0.756 0.911 0.818
4 Attune 0.730 0.953 0.545 0.935 0.217 0.966 0.025 0.725 0.950 0.815
5 Attune 0.733 0.958 0.545 0.935 0.218 0.947 0.011 0.734 0.947 0.819
1 Seurat V5 0.811 0.550 0.854
2 Seurat V5 0.809 0.548 0.850
3 Seurat V5 0.810 0.549 0.852
4 Seurat V5 0.810 0.550 0.853
5 Seurat V5 0.809 0.549 0.851
1 Concerto 0.742 0.522 0.905
2 Concerto 0.734 0.512 0.893
3 Concerto 0.731 0.511 0.892
4 Concerto 0.725 0.510 0.883
5 Concerto 0.721 0.507 0.881

Response Table 2. Metrics for evaluating integration performance in 10X SHARE-seq dataset
fold algorithm map Batch ASW Cell type ASW GC NC SAS FOSCTTM Biology conservation Omics mixing Overall score
1 SMILE 0.630 0.915 0.510 0.881 0.389 0.562 0.015 0.510 0.786 0.620
2 SMILE 0.631 0.920 0.511 0.880 0.388 0.560 0.014 0.51 0.787 0.621
3 SMILE 0.629 0.916 0.509 0.882 0.390 0.561 0.015 0.509 0.786 0.620
4 SMILE 0.630 0.915 0.512 0.881 0.390 0.563 0.014 0.511 0.786 0.621
5 SMILE 0.629 0.914 0.509 0.879 0.388 0.560 0.015 0.509 0.784 0.619
1 Attune 0.701 0.813 0.540 0.851 0.107 0.810 0.012 0.847 0.797 0.827
2 Attune 0.702 0.813 0.540 0.858 0.107 0.809 0.011 0.849 0.799 0.829
3 Attune 0.703 0.812 0.541 0.853 0.107 0.807 0.011 0.851 0.795 0.828
4 Attune 0.704 0.811 0.541 0.853 0.107 0.806 0.010 0.853 0.793 0.829
5 Attune 0.705 0.810 0.542 0.846 0.107 0.813 0.010 0.855 0.792 0.830
1 Seurat V5 0.602 0.483 0.570
2 Seurat V5 0.602 0.484 0.572
3 Seurat V5 0.603 0.482 0.572
4 Seurat V5 0.601 0.481 0.571
5 Seurat V5 0.602 0.482 0.573
1 Concerto 0.467 0.497 0.703
2 Concerto 0.447 0.494 0.684
3 Concerto 0.483 0.499 0.714
4 Concerto 0.497 0.502 0.733
5 Concerto 0.437 0.491 0.660

[1] Luecken, M.D., Burkhardt, D.B., Cannoodt, R., Lance, C., Agrawal, A., Aliee, H., Chen, A.T., Deconinck, L., Detweiler, A.M., and Granados, A.A. A sandbox for prediction and integration of dna, rna, and proteins in single cells. 2021.
[2] Lin Y, Wu T Y, Wan S, et al. scJoint integrates atlas-scale single-cell RNA-seq and ATAC-seq data with transfer learning[J]. Nature biotechnology, 2022, 40(5): 703-710.
[3] Cao Z J, Gao G. Multi-omics single-cell data integration and regulatory inference with graph-linked embedding[J]. Nature Biotechnology, 2022, 40(10): 1458-1466.
[4] Yang M, Yang Y, Xie C, et al. Contrastive learning enables rapid mapping to multimodal single-cell atlas of multimillion scale[J]. Nature Machine Intelligence, 2022, 4(8): 696-709.
[5] Chen T, Kornblith S, Norouzi M, et al. A simple framework for contrastive learning of visual representations[C]//International conference on machine learning. PMLR, 2020: 1597-1607.
[6] Xu Y, Das P, McCord R P. SMILE: mutual information learning for integration of single-cell omics data[J]. Bioinformatics, 2022, 38(2): 476-486.
[7] Hao Y, Stuart T, Kowalski M H, et al. Dictionary learning for integrative, multimodal and scalable single-cell analysis[J]. Nature biotechnology, 2024, 42(2): 293-304.

Reviewer#1 comments 4. The author included a PCA + Transformer as one of comparisons in the task of regulatory prediction. It is questionable if PCA is a proper way to learn the peak embedding. Second, only the top 10 PCs don't justify if all major variations are captured in 10 PCs. I am interested in knowing if NMF-based approach would perform much better than PCA. For example, the author can combine the cisTopic and Transformer.

Response to Reviewer#1 comments 4:

Thank you for your insightful feedback and thoughtful suggestions. We greatly appreciate your comments regarding the use of PCA for learning peak embeddings and the potential advantages of alternative methods such as NMF.
In response to your concern, we performed additional experiments to evaluate the performance of NMF-based methods. Specifically, we compared PCA with 10 components to NMF with both 10 and 20 components. However, as shown in Response Figure 14, the results did not demonstrate a significant improvement over the PCA approach. This suggests that, while NMF may have some potential advantages, the performance gain over PCA was not particularly substantial in our study. We acknowledge that further exploration, including experimentation with different configurations and larger datasets, could provide a more comprehensive assessment of NMF's capabilities in this context.
Regarding cisTopic, we recognize that it is primarily designed for scATAC-seq data and may not be directly applicable to scRNA-seq. In our study, we opted to use PCA as the dimensionality reduction technique for both scRNA-seq and scATAC-seq data to maintain consistency across the two modalities. However, we are uncertain whether the reviewer would prefer us to apply PCA specifically to scRNA-seq and use cisTopic for the scATAC-seq data. If this alternative approach is preferred, we are happy to explore it further and present the corresponding results.
We hope this clarifies our approach and addresses your concerns. Should you require additional information or further analysis, we are more than willing to provide it. Thank you once again for your valuable input.

Reviewer#1 comments 5. It is surprising that all methods perform poorly in predicting distal interactions (Figure 3b). Even though the author showed that Attune outperforms other existing methods, the prediction results in Figure 3b doesn't justify that Attune can accurately identify distal promoter interactions (The false positive rate is so high). The author needs a clear explanation on such issue and adds additional benchmark data for this task.

Response to Reviewer#1 comments 5:

Thank you for your insightful comments regarding the performance of our model and the challenges associated with predicting distal promoter interactions. We agree that Figure 3b highlights notable limitations, particularly in accurately identifying distal interactions, and we provide additional context and detailed interpretations below.
The importance of distal interactions in benchmarking. Distal promoter interactions are biologically significant and essential to understanding gene regulation, even though they are challenging to predict. In our study, we included interactions up to 1.2 Mbps based on the statistical distribution of interaction distances in the dataset from Javierre et al. (2016) [1]. Studies such as Laverré et al. (2022) further emphasize the critical role of long-range interactions in gene expression and chromatin organization [2]. However, most existing methods primarily focus on short-range interactions (e.g., GLUE ≤150 kb, Cicero ≤500 kb). By intentionally including distal interactions in our benchmarks, we aim to highlight their biological importance and encourage improvements in predictive models for these relationships. Ignoring distal interactions could risk overlooking key regulatory mechanisms and limit the development of predictive methods for studying long-range regulation.
Performance trends and challenges of distal predictions. From the PCHi-C dataset results, we observe that true regulatory interactions decrease in frequency as distance increases (see Supplementary Figure 4a), which likely reflects a combination of biological and dataset-specific constraints. Nonetheless, most methods demonstrate higher differentiation capacity for short-range interactions (e.g., 25-50 kb and 50-75 kb bins), as shown in Response Figure 15. The boxplots illustrate that differentiation between true positive (TP) and true negative (TN) interactions diminishes at longer distances for most methods, reflecting the added complexity of long-range regulation. Nonetheless, Attune demonstrates relatively stronger differentiation capability in distal bins compared to other methods, indicating it may capture certain features of long-range regulation. Additionally, Calculating FDR uniformly across methods presents challenges due to the differing scoring systems used by each method (e.g., Spearman’s correlation for association strength, Cicero’s co-accessibility score for chromatin accessibility relationships). As these scores vary in scale and interpretation, defining fair and consistent thresholds across methods is challenging due to differing scoring systems. Instead of relying on thresholds, we visualized score distributions across distance bins to provide a more intuitive and method-agnostic comparison.
Inclusion of the eQTL v10 dataset. In response to your suggestion, we expanded our analysis to include the eQTL v10

dataset, which evaluates significant gene-peak pairs from whole blood samples. This dataset complements the PCHi-C data and allows us to assess Attune's generalizability across broader biological contexts. Since Attune was trained on PBMC data (primarily T cells, B cells, and monocytes), the inclusion of whole blood data helps evaluate its performance in a bulk context. However, Attune does not perform as well on the eQTL dataset, where GLUE achieves higher AUROC scores, likely due to its use of prior knowledge-based guidance graphs that incorporate additional regulatory context. For Attune, we hypothesize that incorporating such biological priors (e.g., enhancer-promoter interaction maps or tissue-specific chromatin features) might improve its performance in tasks involving distal interactions. Additionally, we hypothesize that Attune's predictions may be sensitive to cell-type composition, which could impact its robustness in datasets without cell-type annotations, such as eQTL v10. This remains an aspect requiring further investigation.
Our analysis underscores the importance of including distal interactions in benchmarking studies despite the challenges associated with their prediction. While these challenges are shared across all methods, our results show that Attune demonstrates stronger differentiation capacity in distal bins compared to other approaches. However, this study also highlights the need for future improvements, including integrating biological priors and leveraging single-cell datasets with detailed cell-type annotations to enhance robustness and performance.
We appreciate the reviewer's comments, which prompted us to expand our analysis and address the challenges of distal interaction prediction more comprehensively. If additional details or analyses are required, we would be happy to provide them. Thank you again for your thoughtful feedback.

[1] Javierre, Biola M., et al. "Lineage-specific genome architecture links enhancers and non-coding disease variants to target gene promoters." Cell 167.5 (2016): 1369-1384.
[2] Laverré, Alexandre, Eric Tannier, and Anamaria Necsulea. "Long-range promoter–enhancer contacts are conserved during evolution and contribute to gene expression robustness." Genome Research 32.2 (2022): 280-296.

Reviewer#1 comments 6. It is not obvious that the chromatin regions presented in Figure 4c explain well the bifurcation of lineage commitment in mouse skin data. First, what are key peaks that drive the lineage commitment into the upper branch (IRS and TAC2). Second, these peaks seem to be not differentiable in the lower branch. Say, what drives the bifurcation into Medulla and Hair shaft-cuticle.

Response to Reviewer#1 comments 6:

Thank you for your insightful comments. To clarify the analysis presented in Figure 4c, our initial hypothesis was that Gli3 plays an important role in lineage commitment, based on the inconsistencies observed between two modalities. We then investigated the chromatin accessibility peaks associated with Gli3. Since differentiation is a time-dependent process, we performed pseudotime analysis to explore the relationship between peak accessibility and cell maturation. During this analysis, IRS cells were excluded because, as noted in both Ma's study and our results, IRS consistently occupies an intermediate position along the pseudotime trajectory, whereas hair shaft-cuticle/cortex and medulla cells are found at the differentiation endpoint [1, 2]. Given that IRS, medulla, and hair shaft-cuticle/cortex represent distinct differentiation fates, we focused our pseudotime analysis on the outer sheath differentiation pattern and excluded IRS cells to avoid confounding the results. This decision was documented in the GitHub code provided with the manuscript. However, as you rightly pointed out, this rationale was not sufficiently explained in the main text, leading to potential misinterpretation.
The primary objective of this experiment was to investigate how Gli3 contributes to overall hair follicle development (on a broader scale) rather than its specific role in determining the bifurcation into medulla or hair shaft. At the current stage, our analysis does not resolve how individual Gli3-associated peaks might guide differentiation toward specific lineages. This limitation likely requires more intricate network analyses, integrating multi-gene and multi-pathway interactions, to comprehensively address the underlying mechanisms.
We will revise the manuscript to explicitly clarify these points and provide additional context to avoid similar ambiguities. If additional clarifications or analyses are needed, we are happy to provide further details. Thank you again for your thoughtful comments and for helping us improve the clarity and rigor of our work.

[1] Ma, Sai, et al. "Chromatin potential identified by shared single-cell profiling of RNA and chromatin." Cell 183.4 (2020): 1103-1116.
[2] Abe, Yoshinori, and Nobuyuki Tanaka. "Roles of the hedgehog signaling pathway in epidermal and hair follicle development, homeostasis, and cancer." Journal of developmental biology 5.4 (2017): 12.

Reviewer#1 comments 7. The author made a claim that "The overall upward shift of blocks indicates the lag of RNA modality", backed up by presentation of Figure 5c and Supplementary Figure 8c. These two figures are not

straightforward to make such conclusion. It is suggested for the author to provide a more quantitative visualization or statistical examination to illustrate such transition.

Response to Reviewer#1 comments 7:

We greatly appreciate the reviewer's insightful suggestion to enhance the quantitative rigor of our analysis supporting the claim that "the overall upward shift of blocks indicates the lag of RNA modality." In the original analysis, we used embedding techniques to explore the alignment between RNA and ATAC modalities, primarily to assess whether any cell types exhibit poor modality alignment. This misalignment could suggest a lag in RNA modality. Although the upward shift in the heatmap (Figure 5b), the clustering patterns in the UMAP (Figure 5c), and the distribution of cosine distances (Supplementary Figure 8c) indicate a potential RNA modality lag in certain cell types, we acknowledge that these visual representations alone may not provide sufficient statistical validation.
To address this, we conducted additional statistical analyses to provide a more robust quantitative comparison of modality mismatch across cell types. Specifically, we calculated the distribution of cosine distances (1 - cosine similarity) for each cell type and visualized these results using a boxplot (Response Figure 17). Statistical comparisons using the Wilcoxon rank-sum test revealed significant differences in modality mismatch between nIPC/ExN and other cell types ($p = 1.68e-117$), as well as between ExM and other cell types ($p=7.85e-65$). These findings quantitatively confirm that nIPC/ExN and ExM exhibit elevated modality mismatch compared to other cell types, thus directly supporting the claim of RNA modality lag in these regions.
We sincerely thank the reviewer for their valuable feedback, which has contributed significantly to improving the clarity and rigor of our analysis. Should further clarifications or additional analyses be required, we would be happy to provide them.

We sincerely thank Reviewer#2 for the thoughtful and constructive feedback on our manuscript. Your suggestions have been invaluable, and we have revised the manuscript to address your concerns. Below, we respond to each comment in detail.

Reviewer#2 comments 1. The notations and descriptions in the Methods section in the whole manuscript are quite confusing, making it difficult to follow. A lot of current presentations do not adhere to standard mathematical notation practices. For example:
a. x_indices and x_counts are defined multiple times with different dimensions.
b. In the line "outputs the weighted hidden vector h, $h \in R$ (equation 2)." it is unclear how an observed gene expression variable like x_counts can "output" a hidden vector.
c. Equations 1-8 would benefit from clearer mathematical notations to denote gene embedding, gene hidden states, and use math notations to denote Embedding() as a function, ensuring clarity.
d. The meaning of certain operations, such as those in equations 2 and 4, is unclear—are these referring to element-wise multiplication or vector multiplications?
e. Additionally, variables such as m and m+ are defined only after they are first introduced, which disrupts the logical flow.
f. l() in 26 is not defined.
These are some of the examples. Completely rewriting of the whole method section is probably needed to improve clarity.

Response to Reviewer#2 comments 1:

We would like to sincerely thank Reviewer#2 for the detailed and constructive feedback. Your valuable comments have highlighted several areas that required attention, and we have thoroughly reviewed and revised the Methods section accordingly. Below, we respond to each of the specific points you raised:

On the multiple definitions of X_indices and X_counts with inconsistent dimensions:
Our model uses two input modalities: single-cell ATAC-seq (scATAC-seq) and single-cell RNA-seq (scRNA-seq). For scRNA-seq, the data is organized into a matrix with 10,000 rows (representing individual cells) and 2,000 columns (representing genes). Each column corresponds to a unique gene index ranging from 1 to 2,000, where "gene 1" is in the first column with an index of 1. In the manuscript, "i" denotes the index of a gene within the matrix, and "G" represents the total number of genes, which in this case is 2,000. The matrix "X_indices" contains the gene indices and has dimensions of (N x G), where N is the number of cells and G is the number of genes. Each row of "X_indices"

contains the sequence [1, 2, 3, ..., 2000]. "X_counts" refers to the gene expression matrix, which has the same dimensions as "X_indices" (N x G). We have now unified the definitions and descriptions of "X_indices" and "X_counts" for consistency and clarity.

b-d.

Modify "Teacher network" section in Methods.

For two kinds of single-cell data (scRNA-seq and scATAC-seq), we designed two teacher networks (RNA teacher network and ATAC teacher network) to learn fine-grained representations respectively. The RNA teacher network accepts $X\_indices \in R^G$ and $X\_counts \in R^G$ as input, where G denotes the number of genes. X_indices represents gene indices, which dimension is $(N \times G)$ and X_counts represents the value of gene expression, which dimension is $(N \times G)$. N is the number of cells. For ATAC teacher network accepts input of $Y\_indices \in R^P$ and $Y\_counts \in R^P$, where P denotes the number of peaks. Y_indices represents peak indices, which dimension is $(N \times P)$ and Y_counts represents the value of peak counts, which dimension is $(N \times P)$. Each gene within a cell is represented by $i \in Z^G$ and each peak is represented by $j \in Z^P$.
In the teacher network, the embedding layer maps discrete inputs such as genes, peaks, etc. to continuous vector space. The input of embedding layer of RNA teacher network is each row of X_indices, that is, an integer sequence $x_1, x_2, \ldots, x_G$, where each x_i represents the index of a gene. The purpose of the embedding layer is to map these integers into a dense vector $\text{gene embed}_i \in R^d$ (a d-dimensional embedding vector corresponding to x_i). The embedding layer can be represented by a matrix $E\_RNA \in R^{(G \times d)}$, where G is the number of genes, and d is the dimension of the embedding. $E\_RNA [x_i]$ represents the extraction of the x_i row (i.e. the embedding vector corresponding to the gene i) from the matrix E_RNA through a table lookup operation (equation 1). Then the RNA expression of gene i ($\text{count}_i \in R$) from X_counts is element-wise cross-multiplied with its embedding vector (gene embed_i) to obtain the weighted embedding vector $\text{gene hidden}_i \in R^d$ (equation 2). For scATAC-seq data, we use y_j to represent the index of a peak in Y_indices and the embedding layer of ATAC teacher network is represented by a matrix $E\_ATAC \in R^{(P \times d)}$, where P is the number of peaks. The embedding vector peak embed_j of peak j is obtained by table lookup operation (equation 3). The peak counts of peak j ($\text{count}_j \in R$) from Y_counts is element-wise cross-multiplied with its embedding vector (peak embed_j) to obtain the weighted embedding vector $\text{peak hidden}_j \in R^d$ (equation 4).

e. Definition of variables such as m and m+ introduced only after they are first used:

Modify "Overview of model architecture" section in Methods.

As illustrated in Figure 1b, the input of Attune is a multimodal dataset, also called joint profiling data, which contains information from two modalities, and the cells of the two modalities are paired. Attune is designed based on joint profiling data, which considers the pairing information. We consider the cells in paired scRNA-seq and scATAC-seq as positive pairs (m and m^+ is a pair of positive samples).
The overarching model architecture encompasses the Attune pre-training model, which comprises two asymmetric teacher-student networks, along with two modules dedicated to downstream tasks: the cross-modal prediction module and the transformer-based peak-gene interaction module. Attune leverages separate teacher-student networks to learn cell embeddings from scRNA-seq and scATAC-seq respectively, through cross-modal contrastive learning. The teacher network, designed to be more complex, employs a hierarchical attention mechanism 67, while the student network uses a simpler dense operation.
Learn the representation of cells in two modalities (scRNA-seq and scATAC-seq) by maximizing the consistency between positive pairs in the embedding space. The representations from both modalities are then projected into a common space.

f. Definition of the function l() in equation 26:

Modify "Training objectives" section in Methods.

We train transformer with two objectives function: contrastive loss between gene hidden_{CLS} and peak hidden_{CLS}, RNA-ATAC modality matching loss. gene hidden_{CLS} and peak hidden_{CLS} are CLS tokens in different modalities with N*1*d dimension. They learn the weighted average embedding representing the entire genes or peaks.

The purpose of comparing the two embeddings is to shorten the distance between the matched RNA-ATAC pairs globally. The calculation process of contrastive loss of CLS tokens, is described as equation (24-26).

We sincerely hope that the revisions we have made effectively address your concerns. Regarding your suggestion to rewrite the entire Methods section, we have carefully reviewed and reorganized it to enhance clarity. We deeply appreciate your insightful feedback, which has significantly contributed to improving the overall quality of the manuscript.

Reviewer#2 comments 2. Given that Attune uses paired information in the loss function, is it fair to compare this with other unpaired methods in the data integration performance, such as scJoint? A note should be made for the unpaired methods to clarify.

Response to Reviewer#2 comments 2:

We thank Reviewer#2 for raising this important point. Initially, we selected scJoint and other unpaired methods for comparison due to the limited availability of methods specifically designed for paired data integration at the time. However, we acknowledge that comparing a paired method like Attune with unpaired methods such as scJoint and GLUE may introduce fairness concerns, as these unpaired methods, while capable of handling paired data, do not fully leverage pairing labels.
In response to this concern, and as detailed in our reply to Reviewer#1's Comment 3, we have now included additional benchmarking with other paired data methods such as Seurat V5, SMILE, and Concerto. The updated results show that even when compared to these paired methods, Attune maintains competitive performance, further supporting its efficacy in multimodal integration. Since the detailed benchmarking results are already provided in the response to Reviewer#1’s Comment 3, we do not repeat the results here, but we encourage the reviewer to refer to that section for further details. We hope this addresses the fairness concern raised by Reviewer#2.

Reviewer#2 comments 3. Methods and evaluation metrics that are mentioned in the recent benchmarking paper in single-cell multi-omics prediction and integration (Hu et al.) should be considered in this manuscript to improve the evaluation and benchmarking.
Hu, Y., Wan, S., Luo, Y. et al. Benchmarking algorithms for single-cell multi-omics prediction and integration. Nat Methods (2024).

Response to Reviewer#2 comments 3:

We thank Reviewer#2 for the valuable suggestion. In response, we have indeed benchmarked our method against the recent benchmarking work by Hu et al. (2024) on single-cell multi-omics prediction and integration [1]. A detailed discussion on predicting chromatin accessibility from scRNA-seq data is provided in our response to Reviewer#1’s Comment 1.
Regarding the integration of RNA expression and chromatin accessibility (referred to as vertical integration), we benchmarked Attune alongside several algorithms highlighted in the benchmarking paper by Hu et al., including scAI, MOJITOO, MultiVI, Seurat, scVAEIT, MOFA+, Multigrate, scMVP, MIRA, DeepMAPS, SCOIT, and Schema. These comparisons were performed across 11 RNA + ATAC datasets (see Response Figure 18-20), which are joint profiling data derived from various technologies such as SNARE-seq, SHARE-seq, ISSAAC-seq, 10x Multiome, and DOGMA-seq (Response Figure 21). For the evaluation of vertical integration, we used multiple metrics, including ARI (Adjusted Rand Index), NMI (Normalized Mutual Information), cASW (cell-type labels average silhouette width), cLISI (cell-type separation LISI), and BVC (biological variation conservation). ARI and NMI were used to assess the consistency between cell-type labels and clustering results obtained through the Leiden algorithm. A higher cASW value indicates better accuracy in cell-type separation, while higher cLISI values suggest more effective cell-type separation, indicating better preservation of biological variation. The BVC metric provides a comprehensive performance evaluation, aggregating several metrics (ARI, NMI, cASW, and cLISI) into a single score.
In terms of NMI and ARI, MOJITOO achieved the highest scores, followed by Attune and scAI (Response Figure 18). For the cASW and cLISI metrics, Attune outperformed the other algorithms (Response Figure 19). These results indicate that, while Attune’s performance in cell clustering was slightly lower than that of MOJITOO, it demonstrated superior cell-cell similarity representation, which is a critical factor for accurate integration. Overall, Attune, scAI, and MOJITOO performed the best in terms of vertical integration, as shown in Response Figure 19. We would like to emphasize that the datasets and evaluation pipeline used in our vertical integration benchmarks are consistent with those described in the benchmarking paper by Hu et al. (2024). The data can be accessed at https://mailustceducn-

my.sharepoint.com/:f:/g/personal/hyl2016_mail_ustc_edu_cn/EgYFP7tlTKBBuAhkdtrIOg4B1Eyo-_iBx1VKBWSK0r-9rA?e=gmhocx, and the evaluation pipeline is available at https://github.com/QuKunLab/MultiomeBenchmarking/blob/main/code/Integration/compare/count_metrics_ATAC.ipynb. We hope these additional details address your concerns and strengthen our manuscript.

[1] Hu Y, Wan S, Luo Y, et al. Benchmarking algorithms for single-cell multi-omics prediction and integration[J]. Nature Methods, 2024: 1-13.

Reviewer#2 comments 4. Another very recent method that should be considered to benchmark in terms of gene regulatory identification is SCENT.
+ Sakaue, S., Weinand, K., Isaac, S. et al. Tissue-specific enhancer-gene maps from multimodal single-cell data identify causal disease alleles. Nat Genet 56, 615-626 (2024).

Response to Reviewer#2 comments 4:

We sincerely thank Reviewer#2 for suggesting the inclusion of SCENT as a benchmark for gene regulatory identification. In response to this recommendation, we attempted to run SCENT on our PBMC dataset, categorizing CD4, CD8, and Treg cells as T cells, and testing a total of 87,436 gene-peak pairs, consistent with the benchmarks we conducted for Attune, GLUE, and other methods.
Despite allocating up to 100 threads for the analysis, the computation did not complete after two days of continuous running. Due to resource constraints, we adjusted the number of threads and allowed the task to continue running. However, as of now, results are still pending. We will continue monitoring the process and, if feasible, aim to include the results in our next response. We appreciate your understanding and will make every effort to provide a comprehensive evaluation as soon as possible.

Reviewer#2 comments 5. In addition to the PCHiC database, other resources can be considered for validating the identified gene regulatory regions, such as the GTEx eQTL database and the ABC enhancer database. It would be good to incorporate these resources in benchmarking.

Response to Reviewer#2 Comment 5:

We thank Reviewer#2 for the valuable suggestion to incorporate additional resources for validating gene regulatory regions. In response, we have expanded our analysis to include the eQTL v10 dataset, as detailed in our response to Reviewer#1's Comment 5. The eQTL v10 dataset evaluates significant gene-peak pairs from whole blood samples, providing a complementary perspective to the PCHi-C data.
Our analysis showed that Attune performs better on the PCHi-C dataset, likely due to its closer match with the PBMC-specific cell types used for training. However, on the eQTL v10 dataset, Attune's performance was less competitive, suggesting that its predictions may be influenced by cell-type composition and the absence of detailed cell-type annotations in this dataset. This finding highlights the potential for future improvements, such as incorporating biological priors (e.g., tissue-specific chromatin features) or training on datasets with broader cell-type diversity to enhance its robustness.
Regarding the ABC enhancer database, as it provides model-derived predictions rather than experimentally validated interactions, we prioritized the eQTL v10 dataset as a supplementary resource to the PCHi-C data. While incorporating additional datasets is always valuable, we focused on experimentally validated data for this benchmarking.
We appreciate the importance of evaluating model performance across diverse datasets and believe these insights will guide future improvements to Attune, enhancing its generalizability and applicability.

Reviewer#2 comments 6. The definition of this manuscript of DPAGs (dense peak-associated genes) has the same definition of DORC. This overlap should be discussed within the paper. Additionally, many of the analyses performed on the SHARE-seq data in this manuscript, such as DORC identification and residual analysis, are very similar to those in the original publication by Ma et al. The authors should elaborate on the similarities and differences in findings between the two studies. For example, are the identified DORCs consistent between the studies? Are the residuals highly correlated? At present, it is unclear what new biological insights are gained exclusively from the application of the propsoed method.
Ma, S., Zhang, B., LaFave, L. M., Earl, A. S., Chiang, Z., Hu, Y., ... & Buenrostro, J. D. (2020). Chromatin potential identified by shared single-cell profiling of RNA and chromatin. Cell, 183(4), 1103-1116.

Response to Reviewer#2 Comment 6:

We thank Reviewer#2 for raising this important point about the overlap between the definitions of DPAGs (dense peak-associated genes) and DORCs (domains of regulatory chromatin) [1]. We acknowledge that both approaches share a similar conceptual basis in identifying genes based on their strong associations with regulatory peaks. However, we believe that the differences in methodology between the two analyses are substantial and merit clarification.
（1）Differences in Input Data: The input RNA and ATAC feature dimensions differ between DPAGs and DORCs. Due to the computational demands of Transformer-based models, we reduced the number of genes and peaks used in our analysis. In contrast, DORC analyses typically include a much larger set of features, which affects the associations that can be identified and their scope.
（2）Differences in Window Size: DORC analysis uses a 50 kb window to capture local regulatory associations, while DPAGs in Attune employ a much larger window (>1 Mb) to ensure that distal regulatory interactions, which play a critical role in gene expression and chromatin organization, are also considered.
As detailed in our response to Reviewer#1 Comment 2, the computational demands of Transformer-based models necessitated reducing input feature dimensions to process the large number of peaks and their potential associations with genes efficiently. These constraints significantly impacted our ability to maintain consistency in input features between DPAGs and DORCs, making direct comparisons difficult. In particular, the differences in input size and window range (Points 1 and 2) lead to results that are inherently distinct, which is why we did not perform a direct overlap analysis.
With regard to residual analysis, we recognize that Ma et al. (2020) also utilized residuals based on DORCs, and the gene selection process can impact comparability. For example, in Ma et al., Wnt3 was highlighted as an example of lineage priming, but this gene was excluded from our analysis due to filtering for highly variable genes (HVGs). This difference in gene selection further complicates direct comparisons of residuals between the two studies. While our study was inspired by the framework established by Ma et al., we aimed to explore whether similar biological phenomena could be identified using our Attune + Transformer approach, which incorporates a distinct computational methodology. We also appreciate Reviewer#2's suggestion to discuss the similarities and differences between our study and the work of Ma et al. (2020). Their study provides a comprehensive framework for identifying regulatory elements with an impressive depth of analysis, which we highly respect. Our work seeks to build on this foundation by introducing a Transformer-based approach, though we acknowledge that scaling our method to datasets with hundreds of thousands or millions of features (such as peaks) presents challenges.
In future work, we plan to optimize our model by leveraging the attention mechanism to better capture modality relationships, aiming to provide a more robust, efficient, and convincing analytical framework. This approach will also address current challenges related to feature selection and computational resource demands, enabling more effective and scalable analysis.

[1] Ma, Sai, et al. "Chromatin potential identified by shared single-cell profiling of RNA and chromatin." Cell 183.4 (2020): 1103-1116.

Reviewer#2 comments 7 (minor). The details of the method should be described and highlighted the innovation in the first section of Results, and reference Figure 1b, which is currently not referred to in the main text.

Response to Reviewer#2 Comment 7:

Thanks for your suggestion.

Modify "Attune achieves exceptional overall performance of integration" in main text.

Attune employs a cross-modal contrastive learning approach to integrate scRNA-seq and scATAC-seq data, effectively preserving biological consistency across both modalities. The architecture consists of the Attune pre-trained model and two downstream modules: the cross-modal prediction module and the Transformer-based peak-gene interaction module. To learn cell embeddings from scRNA-seq and scATAC-seq data, Attune utilizes two asymmetric teacher-student networks, which are trained through cross-modal contrastive learning. These learned cell embeddings can be fine-tuned for various downstream tasks, including cross-modal prediction, peak-gene interaction recovery, and differentiation analysis, as depicted in Figures 1a and 1b.

Reviewer#2 comments 8 (minor). In Method Section, Sentence "Positive pairs of embeddings from the two modalities of a cell are considered as positive pairs" is confusing as Positive pairs of embedding is not defined beforehand.

Response to Reviewer#2 Comment 8:

We appreciate Reviewer#2 for pointing out the lack of clarity in the Methods section. To address this, we have revised the explanation to provide better context for the definition of "positive pairs" and to ensure clarity for readers.

Modify “Overview of model architecture” section in Methods.

As illustrated in Figure 1b, the input of Attune is a multimodal dataset, also called joint profiling data, which contains information from two modalities, and the cells of the two modalities are paired. Attune is designed based on joint profiling data, which considers the pairing information. We consider the cells in paired scRNA-seq and scATAC-seq as positive pairs (m and m^+ is a pair of positive samples).
The overarching model architecture encompasses the Attune pre-training model, which comprises two asymmetric teacher-student networks, along with two modules dedicated to downstream tasks: the cross-modal prediction module and the transformer-based peak-gene interaction module. Attune leverages separate teacher-student networks to learn cell embeddings from scRNA-seq and scATAC-seq respectively, through cross-modal contrastive learning. The teacher network, designed to be more complex, employs a hierarchical attention mechanism 67, while the student network uses a simpler dense operation.
Learn the representation of cells in two modalities (scRNA-seq and scATAC-seq) by maximizing the consistency between positive pairs in the embedding space. The representations from both modalities are then projected into a common space.

Editorial Manager®

Cookie Preference Center

We use cookies which are necessary to make our site work. We may also use additional cookies to analyze, improve and personalize our content and your digital experience. For more information, see our Cookie Policy.

You may choose not to allow some types of cookies. However, blocking some types may impact your experience of our site and the services we are able to offer. See the different category headings below to find out more or change your settings.

Allow all

Manage Consent Preferences

Strictly Necessary Cookies

Always active

These cookies are necessary for the website to function and cannot be switched off in our systems. They are usually only set in response to actions made by you which amount to a request for services, such as setting your privacy preferences, logging in or filling in forms. You can set your browser to block or alert you about these cookies, but some parts of the site will not then work.

Functional Cookies

These cookies enable the website to provide enhanced functionality and personalisation. They may be set by us or by third party providers whose services we have added to our pages. If you do not allow these cookies then some or all of these services may not function properly.

Performance Cookies

These cookies allow us to count visits and traffic sources so we can measure and improve the performance of our site. They help us to know which pages are the most and least popular and see how visitors move around the site.

Targeting Cookies

These cookies may be set through our site by our advertising partners. They may be used by those companies to build a profile of your interests and show you relevant adverts on other sites. If you do not allow these cookies, you will experience less targeted advertising.

Cookie List

Clear

Apply Cancel
Consent Leg.Interest

Confirm my choices

Powered by onetrust
