# Supplementary material for: Cross-modal contrastive learning decodes developmental regulatory features through chromatin potential analysis
Source: Gigascience. 2025 Oct 17;14:giaf053. doi: 10.1093/gigascience/giaf053 (PMC12532322; doi:10.1093/gigascience/giaf053)
Supplement: giaf053_Authors_Response_To_Reviewer_Comments_Revision_1 [file giaf053_authors_response_to_reviewer_comments_revision_1.pdf]

Author's Response To Reviewer Comments

We extend our gratitude to the editor for their valuable input and to the reviewers for their careful assessment of our work. Their detailed comments have led to meaningful improvements in the manuscript, and we are thankful for their time and expertise.
We are pleased that the revisions we implemented have successfully resolved the issues previously raised. In what follows, we address the additional points highlighted by the editorial team, providing clear explanations and necessary modifications for each item. We attached a word document of the response to the personal cover letter.

1) Please include the comparison suggested by reviewer #1 (see below).
Reviewer #1:
There is one thing left due to ambiguity in my comment 4. I did intend to see some results on "PCA specifically to scRNA-seq and use cisTopic or NMF for the scATAC-seq data". I understand that PCA is the most common practice for scRNA-seq, but it is discussable which is more advantageous for scATAC-seq, PCA or NMF. Additional results on comparison of applying PCA and NMF to scATAC-seq would be appreciated (Keep the practice of using PCA to scRNA-seq).

We are grateful for Reviewer #1's clarification regarding comment 4. Our initial approach involved applying PCA uniformly to both scRNA-seq and scATAC-seq data, which was a misinterpretation of the requested comparison. Recognizing this, we have revised our analysis to address the methodological comparison with greater precision.
To resolve this, we implemented a dual-strategy dimensionality reduction framework tailored to each data modality: PCA for scRNA-seq, and two specialized methods for scATAC-seq —cisTopic [1] and non-negative matrix factorization (NMF). For cisTopic, we constructed Latent Dirichlet Allocation (LDA) models using the runWarpLDAModels() function with topic numbers spanning 2–40 (specifically: 2, 5–15, 20, 25, 30, 35, 40), 150 iterations, and a fixed random seed (123). Model selection via the selectModel() function with derivative-based optimization identified 11 as the optimal number of topics. For NMF, we applied 20 components. Dimensional alignment between modalities was ensured by harmonizing scRNA-seq principal components (11 or 20) with scATAC-seq latent spaces.
This revised approach led to significant improvements. It achieved AUROCs of 0.542 (cisTopic + PCA + Transformer) and 0.536 (NMF + PCA + Transformer), outperforming our initial uniform PCA strategy (10 PCs: 0.489; 20 PCs: 0.459). The performance disparity underscores PCA's fundamental incompatibility with scATAC-seq data, which exhibits binary, sparse features poorly modeled by linear variance decomposition. In contrast, cisTopic and NMF explicitly accommodate these characteristics through topic modeling and non-negative constraints, respectively. These results align with commonly used methods (such as Cicero, Spearman), validating the necessity of modality-tailored processing. However, these approaches remain slightly inferior to Attune and GLUE. We are grateful to the reviewer for highlighting this important methodological detail, which has made the benchmarking more reasonable. Further details or validation experiments can be provided upon request.

Response Figure 1. Comparison of Regulatory Prediction Performance

[1] Bravo González-Blas, C., Minnoye, L., Papasokrati, D. et al. cisTopic: cis-regulatory topic modeling on single-cell ATAC-seq data. Nat Methods 16, 397–400 (2019).

2) In addition, please register your software application in the bio.tools and SciCrunch.org databases to receive RRID (Research Resource Identification Initiative ID) and biotoolsID identifiers, and include these in your manuscript, in the "software availability" section. These will facilitate tracking, reproducibility and re-use of your tool.

We appreciate for the constructive suggestion regarding the registration of our software application. We have successfully registered it in the bio.tools and SciCrunch.org databases and have obtained the corresponding RRID and biotoolsID identifiers. These identifiers have been incorporated into the "Availability of Source Code and Requirements" section of our manuscript, ensuring that our tool is now

properly cataloged and traceable.

3) I feel your title is a bit hard too parse - please consider to make it a bit easier to understand.

We are grateful for your insightful comments on the title of our manuscript. We have carefully considered your suggestion to enhance clarity and readability. Based on your comments, we have revised the title to “Cross-modal contrastive learning decodes developmental regulatory features through chromatin potential analysis.” We would like to express our sincere gratitude once again for your valuable feedback and hope that the new title meets the requirements.

4) In the bibliography, reference [44] says "invalid citation" - please check if this needs to be corrected

Thank you for bringing this to our attention. We have carefully reviewed reference [44] and have corrected the invalid citation. Additionally, we have thoroughly checked the entire bibliography to ensure that all references adhere to the formatting requirements of GigaScience.

5) We treat internet addresses (URLs, DOIs, etc) as citable items. Please include an individual reference in the bibliography for each internet source that's mentioned in the text, and cite it by reference number, rather than inserting the URL directly. Please refer to our citation guidelines in our instructions for authors.

We have addressed the issue regarding the citation of URLs. Each internet source referenced in the text has now been properly cited using a corresponding reference number, consistent with the journal's requirements. Thank you for your guidance.

6) As mentioned at submission, as a methodology article your article will be published in the "Technical Note" section of the journal. Please choose "Technical Note" as article type when submitting your revised version.

We appreciate your prompt reminder about the article type selection. We acknowledge your guidance and will meticulously ensure that the article type is designated as "Technical Note" upon submission of our revised manuscript.

7) Please also ensure that your revised manuscript conforms to the journal style, which can be found in the Instructions for Authors on the journal homepage.

We appreciate your guidance regarding the journal style requirements. We have carefully reviewed the Instructions for Authors on the journal homepage and have ensured that our revised manuscript fully conforms to the specified journal style.

Editorial Manager®

Cookie Preference Center

We use cookies which are necessary to make our site work. We may also use additional cookies to analyze, improve and personalize our content and your digital experience. For more information, see our Cookie Policy.

You may choose not to allow some types of cookies. However, blocking some types may impact your experience of our site and the services we are able to offer. See the different category headings below to find out more or change your settings.

Allow all

Manage Consent Preferences

Strictly Necessary Cookies

Always active

These cookies are necessary for the website to function and cannot be switched off in our systems. They are usually only set in response to actions made by you which amount to a request for services, such as setting your privacy preferences, logging in or filling in forms. You can set your browser to block or alert you about these cookies, but some parts of the site will not then work.

Functional Cookies

These cookies enable the website to provide enhanced functionality and personalisation. They may be set by us or by third party providers whose services we have added to our pages. If you do not allow these cookies then some or all of these services may not function properly.

Performance Cookies

These cookies allow us to count visits and traffic sources so we can measure and improve the performance of our site. They help us to know which pages are the most and least popular and see how visitors move around the site.

Targeting Cookies

These cookies may be set through our site by our advertising partners. They may be used by those companies to build a profile of your interests and show you relevant adverts on other sites. If you do not allow these cookies, you will experience less targeted advertising.

Cookie List

Clear

Apply Cancel

Consent Leg.Interest

Confirm my choices

Powered by onetrust
