# Supplementary material for: Cross-modal contrastive learning decodes developmental regulatory features through chromatin potential analysis
Source: Gigascience. 2025 Oct 17;14:giaf053. doi: 10.1093/gigascience/giaf053 (PMC12532322; doi:10.1093/gigascience/giaf053)
Supplement: giaf053_Reviewer_1_Report_Original_Submission — Yang Xu -- 10/15/2024 [file giaf053_reviewer_1_report_original_submission.pdf]

Reviewer Report

Title: Cross-modal contrastive learning decodes developmental regulatory features through chromatin potential analysis

Version: Original Submission **Date:** 10/15/2024

Reviewer name: Yang Xu

Reviewer Comments to Author:

Yang et al. proposed a framework of cross-modal integration that involves of pretraining with contrastive learning and fine-tuning with transformer. They demonstrated that this framework Attune outperforms existing methods and showed that Attune identified potential chromatin regulation factor participating in state transition in cortical neuron differentiation. Demonstration of Attune by Yang et al. could capture a great attention in such research domain and would also have great interest for cross-modal integration at atlas scale.Major comments:1. As for model architecture presented in Figure 1b, the author included a one-way translation from ATAC counts to RNA counts. MSE loss was applied to minimize the reconstruction of translation from ATAC to RNA. What is the rationale behind such one-way translation? Why not translation from RNA counts to ATAC counts, or explicitly having bidirectional translation between these two modalities?2. The gene-peak cross-attention seems to be a very big computation, not to mention the computation needed for the peak-peak self-attention (It is not clear to me if peak-peak self-attention is included in the model. If not, why peak-peak attention is unnecessary?). It is unclearwhat kind of computational power is enough to do the transformer fine-tuning. Meanwhile, it is also not clear why the author didn't combine transformer fine-tuning with pretraining as one end-to-end training? What are the benefits of dividing the model into two parts?3. It is not clear how benchmarking was done. Both 10X multiome and SHARE-seq are joint profiling data. It is necessary for the author to provide more details about how other methods were run under author's hands, especially how the author handled data preprocessing for each method. Meanwhile, each cell has profiles of two modalities. Attune clearly takes advantage of knowing such correspondence. However, other methods primarily use shared features for integration, which will not take a full advantage of joint profiling. Seurat V3 was included in benchmarking. This version of Seurat also uses shared features. The author will need to consider adding Seurat V5 in benchmarking, which uses information of joint profiling for integration. Another existing contrastive learning SMILE (https://doi.org/10.1093/bioinformatics/btab706) was also proposed to integrate RNA-seq and ATAC-seq with joint-profiling data. Meanwhile, the author previously developed another contrastive learning integration method Concerto (https://www.nature.com/articles/s42256-022-00518-z). It should also be included in benchmarking. The author may need to include more clear justifications on how Attune is improved from their previous work Concerto.4. The author included a PCA + Transformer as one of comparisons in the task of regulatory prediction. It is questionable if PCA is a proper way to learn the peak embedding. Second, only the top 10 PCs don't justify if all major variations are captured in 10 PCs. I am interested in knowing if NMF-based approach would perform much better than PCA. For example, the author can combine the cisTopic (https://www.nature.com/articles/s41592-019-0367-1) and

Transformer.5. It is surprising that all methods perform poorly in predicting distal interactions (Figure 3b). Even though the author showed that Attune outperforms other existing methods, the prediction results in Figure 3b doesn't justify that Attune can accurately identify distal promoter interactions (The false positive rate is so high). The author needs a clear explanation on such issue and adds additional benchmark data for this task.6. It is not obvious that the chromatin regions presented in Figure 4c explain well the bifurcation of lineage commitment in mouse skin data. First, what are key peaks that drive the lineage commitment into the upper branch (IRS and TAC2). Second, these peaks seem to be not differentiable in the lower branch. Say, what drives the bifurcation into Medulla and Hair shaft-cuticle.7. The author made a claim that "The overall upward shift of blocks indicates the lag of RNA modality", backed up by presentation of Figure 5c and Supplementary Figure 8c. These two figures are not straightforward to make such conclusion. It is suggested for the author to provide a more quantitative visualization or statistical examination to illustrate such transition.

Level of Interest

Please indicate how interesting you found the manuscript: Choose an item.

Quality of Written English

Please indicate the quality of language in the manuscript: Choose an item.

Declaration of Competing Interests

Please complete a declaration of competing interests, considering the following questions:

- Have you in the past five years received reimbursements, fees, funding, or salary from an organisation that may in any way gain or lose financially from the publication of this manuscript, either now or in the future?
- Do you hold any stocks or shares in an organisation that may in any way gain or lose financially from the publication of this manuscript, either now or in the future?
- Do you hold or are you currently applying for any patents relating to the content of the manuscript?
- Have you received reimbursements, fees, funding, or salary from an organization that holds or has applied for patents relating to the content of the manuscript?
- Do you have any other financial competing interests?
- Do you have any non-financial competing interests in relation to this paper?

If you can answer no to all of the above, write 'I declare that I have no competing interests' below. If your reply is yes to any, please give details below.

I declare that I have no competing interests

I agree to the open peer review policy of the journal. I understand that my name will be included on my report to the authors and, if the manuscript is accepted for publication, my named report including any attachments I upload will be posted on the website along with the authors' responses. I agree for my report to be made available under an Open Access Creative Commons CC-BY license (http://creativecommons.org/licenses/by/4.0/). I understand that any comments which I do not wish to be included in my named report can be included as confidential comments to the editors, which will not be published.

Choose an item.

To further support our reviewers, we have joined with Publons, where you can gain additional credit to further highlight your hard work (see: https://publons.com/journal/530/gigascience). On publication of this paper, your review will be automatically added to Publons, you can then choose whether or not to claim your Publons credit. I understand this statement.

Yes Choose an item.
