# Supplementary material for: Cross-modal contrastive learning decodes developmental regulatory features through chromatin potential analysis
Source: Gigascience. 2025 Oct 17;14:giaf053. doi: 10.1093/gigascience/giaf053 (PMC12532322; doi:10.1093/gigascience/giaf053)
Supplement: giaf053_Reviewer_2_Report_Original_Submission — Yingxin Lin -- 10/18/2024 [file giaf053_reviewer_2_report_original_submission.pdf]

Reviewer Report

Title: Cross-modal contrastive learning decodes developmental regulatory features through chromatin potential analysis

Version: Original Submission **Date:** 10/18/2024

Reviewer name: Yingxin Lin

Reviewer Comments to Author:

Yang et al. introduce Attune, a cross-modal contrastive learning framework designed to align gene expression and chromatin accessibility data. Systematic benchmarking demonstrates Attune's superior performance in omics integration and gene expression prediction. Leveraging a Transformer-based cross-modal attention mechanism, Attune infers regulatory interactions and identifies differential signals across cell subtypes. However, the methods section of this paper lacks clarity, and the benchmarking is limited to only two datasets, leading to the performance of the proposed method remains unclear. Additionally, it is unclear the extent that the reported biological insights are directly attributable to the proposed method.Major:1. The notations and descriptions in the Methods section in the whole manuscript are quite confusing, making it difficult to follow. A lot of current presentations do not adhere to standard mathematical notation practices. For example:a. x_indices and x_counts are defined multiple times with different dimensions.b. In the line "ð'¥ð'ð'œð'¢ð'›ð'¡ð' outputs the weighted hidden vector ð'"ð''ð'›ð'' hð'–ð''ð''ð''ð'›ð'–, ð'"ð''ð'›ð'' hð'– ð''ð''ð''ð'›ð'– â^^ Rð'' (equation 2)." it is unclear how an observed gene expression variable like x_counts can "output" a hidden vector.c. Equations 1-8 would benefit from clearer mathematical notations to denote gene embedding, gene hidden states, and use math notations to denote Embedding() as a function, ensuring clarity.d. The meaning of certain operations, such as those in equations 2 and 4, is unclearâ€"are these referring to element-wise multiplication or vector multiplications?e. Additionally, variables such as m and m+ are defined only after they are first introduced, which disrupts the logical flow.f. l() in 26 is not defined.These are some of the examples. Completely rewriting of the whole method section is probably needed to improve clarity.2. Given that Attune uses paired information in the loss function, is it fair to compare this with other unpaired methods in the data integration performance, such as scJoint? A note should be made for the unpaired methods to clarify.3. Methods and evaluation metrics that are mentioned in the recent benchmarking paper in single-cell multi-omics prediction and integration (Hu et al.) should be considered in this manuscript to improve the evaluation and benchmarking.+ Hu, Y., Wan, S., Luo, Y. et al. Benchmarking algorithms for single-cell multi-omics prediction and integration. Nat Methods (2024). https://doi.org/10.1038/s41592-024-02429-w4. Another very recent method that should be considered to benchmark in terms of gene regulatory identification is SCENT.+ Sakaue, S., Weinand, K., Isaac, S. et al. Tissue-specific enhancer-gene maps from multimodal single-cell data identify causal disease alleles. Nat Genet 56, 615-626 (2024). https://doi.org/10.1038/s41588-024-01682-15. In addition to the PCHiC database, other resources can be considered for validating the identified gene regulatory regions, such as the GTEx eQTL database and the ABC enhancer database. It would be good to incorporate these

resources in benchmarking.6. The definition of this manuscript of DPAGs (dense peak-associated genes) has the same definition of DORC with FigR. This overlap should be discussed within the paper. Additionally, many of the analyses performed on the SHARE-seq data in this manuscript, such as DORC identification and residual analysis, are very similar to those in the original publication by Ma et al. The authors should elaborate on the similarities and differences in findings between the two studies. For example, are the identified DORCs consistent between the studies? Are the residuals highly correlated? At present, it is unclear what new biological insights are gained exclusively from the application of the propsoed method.+ Ma, S., Zhang, B., LaFave, L. M., Earl, A. S., Chiang, Z., Hu, Y., ... & Buenrostro, J. D. (2020). Chromatin potential identified by shared single-cell profiling of RNA and chromatin. Cell, 183(4), 1103-1116.Minor:1. The details of the method should be described and highlighted the innovation in the first section of Results, and reference Figure 1b, which is currently not referred to in the main text.2. In Method Section, Sentence "Positive pairs of embeddings from the two modalities of a cell are considered as positive pairs" is confusing as Positive pairs of embedding is not defined beforehand.

Level of Interest

Please indicate how interesting you found the manuscript: Choose an item.

Quality of Written English

Please indicate the quality of language in the manuscript: Choose an item.

Declaration of Competing Interests

Please complete a declaration of competing interests, considering the following questions:

- Have you in the past five years received reimbursements, fees, funding, or salary from an organisation that may in any way gain or lose financially from the publication of this manuscript, either now or in the future?
- Do you hold any stocks or shares in an organisation that may in any way gain or lose financially from the publication of this manuscript, either now or in the future?
- Do you hold or are you currently applying for any patents relating to the content of the manuscript?
- Have you received reimbursements, fees, funding, or salary from an organization that holds or has applied for patents relating to the content of the manuscript?
- Do you have any other financial competing interests?
- Do you have any non-financial competing interests in relation to this paper?

If you can answer no to all of the above, write 'I declare that I have no competing interests' below. If your reply is yes to any, please give details below.

I declare that I have no competing interests.

I agree to the open peer review policy of the journal. I understand that my name will be included on my report to the authors and, if the manuscript is accepted for publication, my named report including any attachments I upload will be posted on the website along with the authors' responses. I agree for my report to be made available under an Open Access Creative Commons CC-BY license (http://creativecommons.org/licenses/by/4.0/). I understand that any comments which I do not wish to be included in my named report can be included as confidential comments to the editors, which will not be published.

Choose an item.

To further support our reviewers, we have joined with Publons, where you can gain additional credit to further highlight your hard work (see: https://publons.com/journal/530/gigascience). On publication of this paper, your review will be automatically added to Publons, you can then choose whether or not to claim your Publons credit. I understand this statement.

Yes Choose an item.
